# 42nd International Symposium on Intensive Care & Emergency Medicine

**DOI:** 10.1186/s13054-023-04377-x

**Published:** 2023-03-21

**Authors:** 

## P001

### Effective FiO_2_ delivered by a new frugal CPAP system with low oxygen needs: from bench to clinical observations

#### E. De Beaufort^1^, G. Carteaux^2^, F. Morin^3^, F. Beloncle^4^, A. Lesimple^5^, D. Savary^3^, A. Mercat^4^, L. Brochard^6^, J. C. Richard^4^, A. Mekontso-Dessap^2^

##### ^1^Université Paris-Est Créteil, Laboratoire CARMAS, IMRB, Créteil, France, ^2^Assistance Publique-Hôpitaux de Paris, CHU Henri Mondor-Albert Chenevier, Service de Médecine Intensive Réanimation, Créteil, France, ^3^Faculté de Santé, Centre Hospitalier Universitaire d’Angers, Université d’Angers, Département de Médecine d’Urgence, Angers, France, ^4^Faculté de Santé, Centre Hospitalier Universitaire d’Angers, Vent’ Lab, Université d’Angers, Département de Médecine Intensive-Réanimation et Médecine Hyperbare, Angers, France, ^5^Université d’Angers, CNRS, INSERM 1083, MITOVASC, Angers, France, ^6^St. Michael’s Hospital, Interdepartmental Division of Critical Care Medicine, Toronto, Canada

*Critical Care* 2023, **27(S1)**: P001

**Introduction:** In the context of a pandemic with a massive influx of hypoxemic patients, the high oxygen consumption usually required to achieve optimal inspired fraction of oxygen (FiO_2_) with non-invasive respiratory supports may jeopardize healthcare organization and oxygen delivery capabilities in hospitals.

**Methods:** A new CPAP device aimed at optimizing oxygen delivery with minimal oxygen needs and named “Bag-CPAP”, was designed and evaluated:A bench study compared performances of Bag-CPAP and four other non-invasive devices to those of an ICU ventilator. With the objective of delivering a PEEP level between 5 and 10 cmH_2_O, FiO_2_ actually delivered at two target ranges (40–60% and 80–100%) and oxygen consumption were measured for two different simulated patients. An observational clinical study evaluating the Bag-CPAP was conducted in France, including 20 adult patients with de novo acute respiratory failure. Actual FiO_2_ and PEEP as well as oxygen saturation (SpO_2_), respiratory rate (RR) and dyspnea score were assessed.

**Results:** 1. Bench study: All systems tested reached the minimal FiO_2_ target range (40–60%) and four were able to reach the higher target range. The ratio between FiO_2_ and oxygen consumption was the highest with the Bag-CPAP and the lowest with high-flow oxygen device whatever the FiO_2_ target.

2. Clinical study: The Bag-CPAP was well tolerated and reached the two FiO_2_ target ranges, 53% [52–53%] for moderate FiO_2_ and 94% [93–96%] for high FiO_2_ with an oxygen flow rate of at least 5 L/min and 15 L/min, respectively (Fig. 1). SpO_2_ significantly increased and dyspnea significantly decreased within the first hour of treatment (93% [90–97%] vs. 97% [95–98%], *p* = 0.001 and 4 [2–7] vs. 3 [2–4], *p* = 0.017, respectively). There was no significant effect on RR.

**Conclusions:** This study shows that the Bag-CPAP has the highest oxygen saving properties. The clinical observation confirmed bench experiments suggesting good clinical tolerance and the capacity to alleviate respiratory distress.

**Figure Figa:**
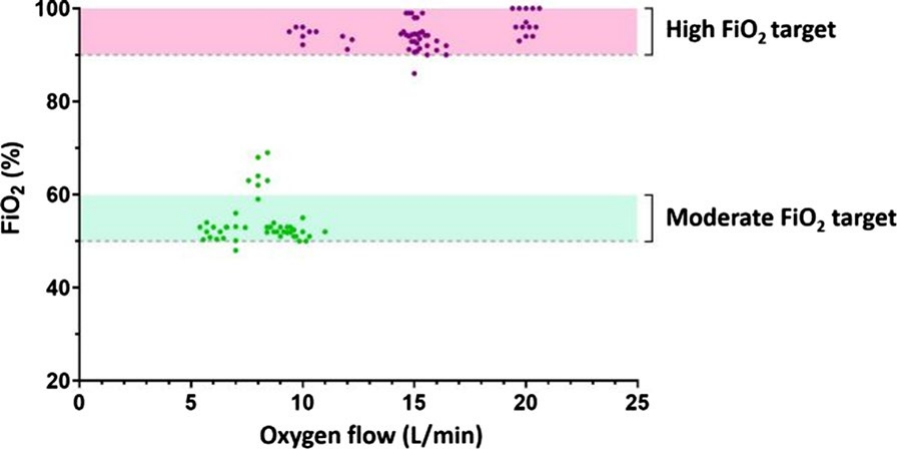
**Fig. 1 (abstract P001)**. FiO_2_ measurements according to oxygen flow rate during Bag-CPAP treatment and FiO_2_ target range (moderate: green or high: pink)

## P002

### Influence of intraoperative liberal versus conservative O_2_ fraction on pulmonary function in adults undergoing spine surgery

#### SC Meena, K Jain, A Ghimere, R Chauhan, A Luthra, V Kumar

##### Pgimer Chandigarh, Department of Anesthesia and Intensive Care, Chandugarh, India

*Critical Care* 2023, **27(S1)**: P002

**Introduction:** Spine surgery patients are very prone to development of postoperative pulmonary complications. We aimed to compare the impact of different intraoperative fractional inspiratory concentration of oxygen on postoperative pulmonary outcomes including oxygenation index and lactate values after thoracolumbar spine instrumentation.

**Methods:** Total 75 American Society of Anesthesiologists I–II adult patients aged 18–60 years who were scheduled to undergo thoraco-lumbar spine instrumentation were randomly allocated into group A: (conservative oxygen) FiO_2_ = 0.30 (30% oxygen with air) and group B: (Liberal Oxygen) FiO_2_ = 0.80 (80% oxygen with air). We aimed to compare the changes in PaO_2_/FiO_2_ ratio, lactate values, C-reactive protein, interleukin-6, PONV and SSI at the different study time intervals.

**Results:** At the end of surgery after extubation, the mean (SD) of PaO_2_/FiO_2_ ratio in 0.3 FiO_2_ and 0.8 FiO_2_ groups were 424.69 (53.69) and 315.57 (48.62) respectively. The values of lactate in both the groups increased at the end of surgery from a mean of 0.93 pg/ml in both the groups preoperatively to 1.18 pg/ml and 1.74 pg/ml in 0.3 and 0.8 FiO_2_ groups postoperatively respectively. No significant changes in the levels of interleukin 6 and CRP were found in our study when compared between baseline values and the values after intervention.

**Conclusions:** This study demonstrated the superior efficacy of conservative oxygen inspiratory fraction (0.3% FiO_2_) as compared to liberal FiO_2_ (0.8% FiO_2_), in terms of better PaO_2_/FiO_2_ ratio and low lactate values in patients undergoing surgery for thoraco-lumbar spine.

## P003

### Effect of non-invasive respiratory support on interstitial lung disease with acute respiratory failure: a systematic review and meta-analysis

#### N Sanguanwong^1^, N Jantarangsi^2^, J Ngeyvijit^3^, N Owattanapanich^4^, V Phoophiboon^5^

##### ^1^Chulalongkorn University, Department of Physiology, Bangkok, Thailand, ^2^Buddhachinaraj hospital, Department of Internal Medicine, Phitsanulok, Thailand, ^3^Chulalongkorn University, Division of Pulmonary and Critical Care Medicine, Bangkok, Thailand, ^4^Mahidol University, Division of Trauma Surgery, Bangkok, Thailand, ^5^Chulalongkorn University, Critical Care Medicine, Bangkok, Thailand

*Critical Care* 2023, **27(S1)**: P003

**Introduction:** The data of non-invasive respiratory support (non-invasive positive pressure ventilation; NIPPV and high flow nasal cannula; HFNC) in interstitial lung diseases (ILDs) with acute respiratory failure (ARF) are scarce [1, 2]. Our study aimed to identify the benefits of non-invasive respiratory support on this patient subgroup.

**Methods:** MEDLINE, EMBASE and the Cochrane Library searches were conducted from 1946 to June 2022. An additional search of relevant primary literature and review articles was also performed. A random effects model was used to estimate the PF ratio, PaCO_2_, mortality, intubation rate and hospital length of stay.

**Results:** Of 380 studies, 9 articles were included in the meta-analysis. The non-invasive respiratory support demonstrated a significant improvement in PF ratio compared to conventional oxygen therapy (COT), the mean difference was 54.58 (95% CI [9.48–9.69]) (Fig. 1). Compared to HFNC, there was a significant increase in PF ratio in NIPPV (mean difference 0.45; 95% CI [0.12–0.79]). There were no mortality and intubation rate benefits when comparing between NIPPV and HFNC, mean difference were 1.1; 95% CI [0.83–1.44] and 1.86; 95% CI [0.42–8.33], respectively. In addition, there was a significant decrease in hospital length of stay in HFNC compared to NIPPV (mean difference 9.27; 95% Cl [1.45–17.1]).

**Conclusions:** Non-invasive respiratory support might be an alternative modality in ILDs with ARF. NIPPV demonstrated a potential to improve the PF ratio compared to HFNC. There was no evidence to support a benefit of NIPPV and HFNC in terms of mortality and intubation rate.


**References**
Rochwerg B et al. Intensive Care Med. 2020;46:2226–37.Rochwerg B et al. Eur Respir J. 2017;50:1602426.

**Figure Figb:**
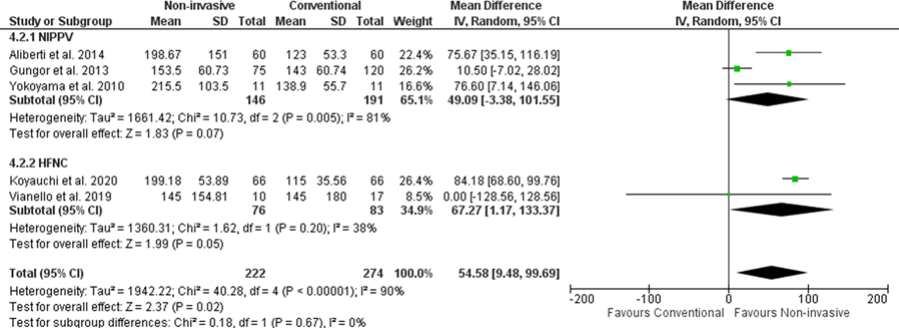
**Fig. 1 (abstract P003)**. Effect of non-invasive respiratory support on PF ratio

## P004

### Causes of mortality of severe COVID-19 patients receiving high-flow oxygen therapy

#### Z Göçerler, R Akkurt, E Paksoy Şenol, E Karakoç, B Yelken

##### Eskişehir Osmangazi University Medical Faculty, Anesthesiology and Reanimation Intensive Care Unit, Eskişehir, Turkey

*Critical Care* 2023, **27(S1)**: P004

**Introduction:** High-flow oxygen therapy (HFOT) is used to provide oxygenation and reduce the need for intubation in severe pneumonia cases caused by SARS-CoV-2 virus. In this study, causes of mortality during hospitalization in the intensive care unit (ICU) in patients receiving HFOT therapy were investigated.

**Methods:** The data of 215 adult patients, who were admitted to ICU of a university hospital between April 2020 and October 2021, with severe COVID pneumonia and received HFOT were enrolled retrospectively in our study.

**Results:** Total mortality among patients was 158 (73.4%). The overall mean age was 72 years, 61 in the survivor group and 73 in the mortality group (*p* < 0.001). It was determined that mortality rates decreased as the length of stay (LoS) in ICU and HFOT duration of the patients increased (*p* = 0.008 and < 0.001, respectively). The increase in respiratory rate on arrival was found to be significantly associated with increased mortality. Although the goal of HFOT is to improve oxygenation, no significant mortality-related difference was found in terms of pO_2_, pCO_2_ and P/F values calculated at the time of admission. The ROC curve was applied to examine the differential effect of Apache-II, SOFA, ROX and Procalcitonin measurements according to the survival. The area under the curve (AUC) and cut-off values were calculated as follows: APACHE-II (63.9%, 5,) SOFA (62.8%, 2), ROX index (66.8%, 4.72), Procalcitonin (65.7%, 0.23) (Fig. 1).

**Conclusions:** Unlike reports in literature on mortality in ICU, LoS was found to have a decreasing effect on mortality rate [1]. In addition to the well-known scoring systems such as APACHE-II and SOFA, the ROX index, which is used to predict the success of HFOT, has emerged as a predictive value for mortality at admission to the intensive care unit [2].


**References**
Moitra VK et al. Crit Care Med 2016;44:655–662.Chandel A et al. Respir Care 2021;66:909–919.

**Figure Figc:**
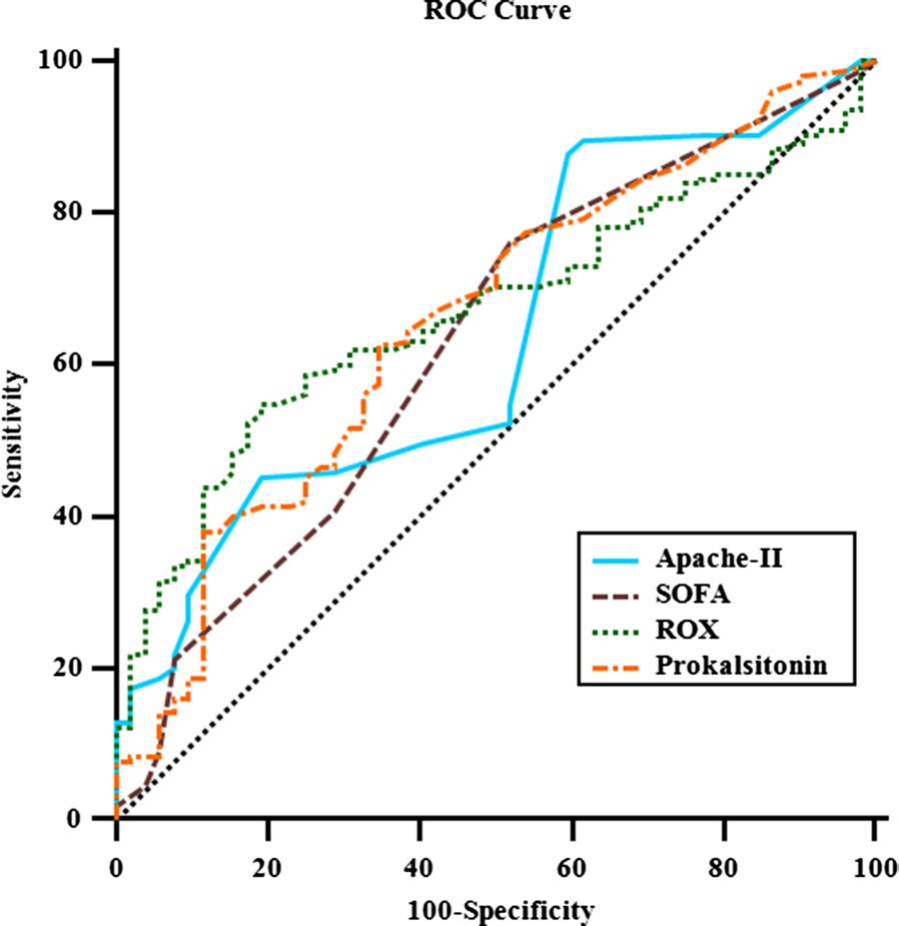
**Fig. 1 (abstract P004)**. ROC curve of APACHE-II, SOFA, ROX and procalcitonin relation with mortality

## P005

### Patient-ventilator interaction during non invasive ventilation with helmet: a comparison between pressure support ventilation and the new neural pressure support (NPS) software

#### A Costa^1^, S Zorzi^2^, A Rivolta^2^, G Cammarota^3^, R Vaschetto^4^, A Pagni^5^, DC Davide Colombo^5^

##### ^1^Ospedale SS. Trinità Borgomanero, Borgomanero, Italy, ^2^Eastern Piedmont University, Novara, Italy, ^3^Perugia University, Anesthesia and Intensive Care, Perugia, Italy, ^4^Eastern Piedmont University, Anesthesia and Intensive Care, Novara, Italy, ^5^Ospedale SS. Trinità Borgomanero, Anesthesia and Intensive Care, Borgomanero, Italy

*Critical Care* 2023, **27(S1)**: P005

**Introduction:** The NIV success is highly affected by patient comfort and patient-ventilator synchronism. The helmet is one of the most comfortable interfaces, even if synchronism is lower due to dead space. The use of neurally adjusted ventilatory assist, in which the electrical activity of the diaphragm (EAdi) drives the ventilator, has shown improvement in comfort and synchronism but still some limitation with helmet, due to the long pressurization time. pressure-support ventilation (PSV) is still the most used and diffused assisted mode, due to its simplicity and effectiveness in helmet-NIV. We tested a new ventilation software called neural pressure support (NPS), which merges the rapid pressurization of PSV along with the EAdi trigger, on a population of 24 critical care patients ventilated via helmet-NIV.

**Methods:** PSV and NPS 30-min trials were started in random order. A physician expert in mechanical ventilation optimized ventilator setting blinded for EAdi and 2 new 30-min trials were started. Tracings were recorded and the last 2 min of each analyzed. As primary endpoint we evaluated TimeSync, defined as the time during which both EAdi and Flow tracings were in the inspiratory phase. Trigger delay and TimeSynch to neural inspiratory time (TS/TN) was calculated.

**Results:** Mean TimeSync was statistically higher in NPS compared to PSV (0.632 s vs 0.469 s, *p* < 0.01). Inspiratory trigger delay was lower in NPS as compared to PSV (0.124 s vs 0.278 s, *p* < 0.01) (Fig. 1). Optimization by the expert appears to have no statistical significance for both TimeSync and Delay (0.56 vs 0.54, *p* = 0.55; 0.21 s vs 0.18 s, *p* = 0.12). TS/TN was higher in NPS versus PSV (0.76 vs 0.57, *p* < 0.01) with no difference after the optimization (0.67 vs 0.66, *p* = 0.6693).

**Conclusions:** Based on this preliminary data NPS seems to increase patient-ventilator synchronism during helmet-NIV both improving TimeSynch and shortening trigger delay. These improvements seem to be not affected by the expertise of the physician that set the ventilator.

**Figure Figd:**
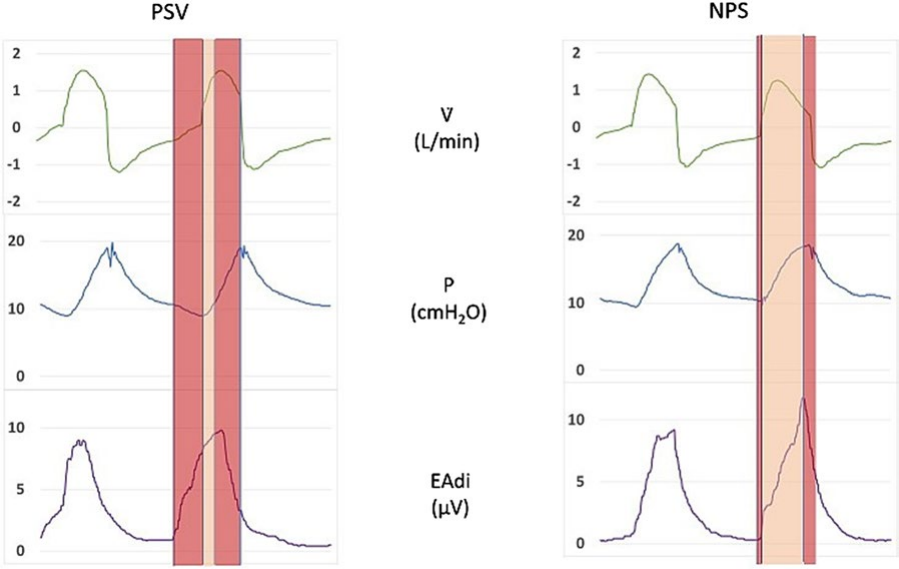
**Fig. 1 (abstract P005)**. Representative flow (V), airways pressure (P) and electrical activity of the diaphragm (EAdi) tracings from one patient both in PSV and in NPS. In light transparency is highlighted the time during which both patient and ventilator are in inspiratory phase. In dark transparency is highlighted the trigger and cycling off delay. A longer inspiratory synchrony time is appreciable in NPS versus PSV

## P006

### Tidal volume measurement during non-invasive respiratory support by helmet continuous-flow CPAP is feasible and accurate in a bench model

#### A Coppadoro^1^, G Bellani^2^, G Foti^2^

##### ^1^San Gerardo Hospital, Department of Anesthesia and Intensive Care, Monza, Italy, ^2^University of Milan-Bicocca, Department of Medicine and Surgery, Monza, Italy

*Critical Care* 2023, **27(S1)**: P006

**Introduction:** We investigated a novel technique designed to measure tidal volume during non-invasive helmet continuous-flow CPAP, a device for non-invasive respiratory support largely used during the recent COVID-19 pandemic to treat acutely ill hypoxic respiratory failure patients.

**Methods:** An active lung simulator coupled with a helmet CPAP was used to compare measured and reference tidal volumes at PEEP 5, 10 and 15 cmH_2_O and different levels of distress (pMusc 10, 15 20 and 25 cmH_2_O; respiratory rate 15, 20, 25 breaths per minutes). Tidal volume measurement was based on helmet outflow-trace analysis. Helmet inflow was increased from 60 to 75 and 90 L/min to match patients’ inspiratory flow; an additional subset of tests was conducted in condition of purposely insufficient inflow (i.e.: high respiratory distress and 60 L/min inflow).

**Results:** Explored tidal volumes ranged from 250 to 910 mL. The Bland-Altman analysis showed a bias of −3.2 ± 29.3 mL for measured tidal volumes as compared to reference, corresponding to an average relative error of −1 ± 4.4% (see Fig. 1). At univariate analyses, tidal volume underestimation correlated with respiratory rate (rho = .411, *p* = .004) but not with peak inspiratory flow, distress, or PEEP. When the helmet inflow was purposely maintained insufficient as compared to the simulated inspiratory flow, the Bland-Altman analysis showed a significant tidal volume underestimation (bias −93.3 ± 83.9 mL), corresponding to an error of −14.8 ± 6.3%.

**Conclusions:** We showed that tidal volume measurement is feasible and accurate in a model of bench continuous-flow helmet CPAP therapy by the analysis of the outflow signal, provided that helmet inflow is maintained adequate to match patient’s inspiratory efforts. Insufficient inflow resulted in tidal volume underestimation.

**Figure Fige:**
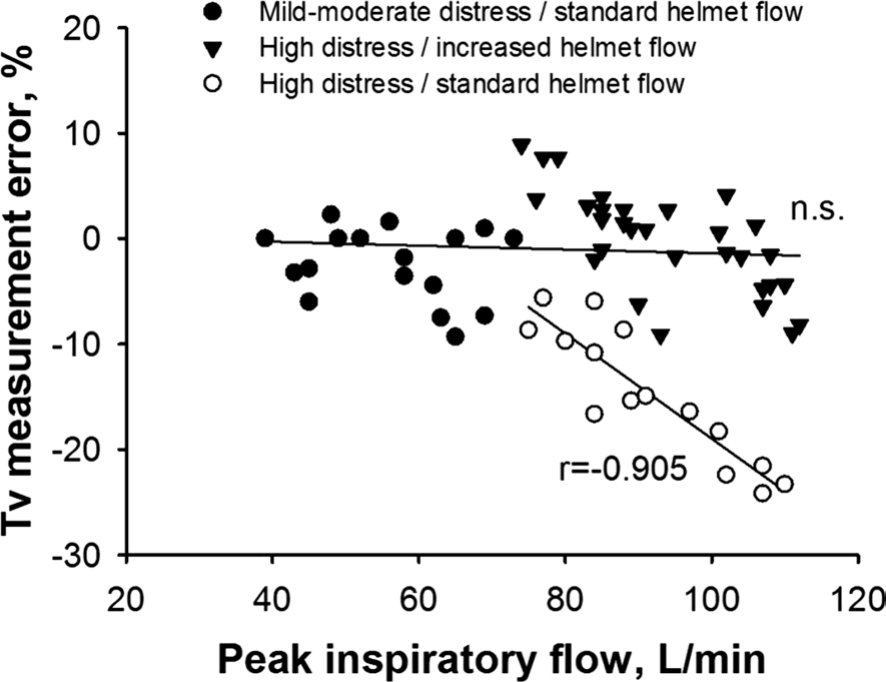
**Fig. 1 (abstract P006)**. Tidal volume measurement error was lower than ± 10% during helmet CPAP therapy, provided that the inflow was maintained adequate to patient’s respiratory distress (filled symbols). A standard helmet inflow (60 L/min), a value lower than peak inspiratory flow in case of high respiratory distress (pMusc 20 and 25 cmH_2_O, empty circles), lead to underestimation of tidal volumes; the higher the peak inspiratory flow, the higher the underestimation error (r = − 0.905 *p* < .001)

## P007

### Non-invasive ventilation in ≥ 70 year old COVID-19 patients in a level two unit: retrospective cohort analysis, comparison with COVIP substudy and defense of a multifaceted work of breathing control driven strategy

#### J Rua, P Costa, AR Nogueira, JM Mateus

##### Centro Hospitalar Universitário de Coimbra, Serviço de Medicina Intensiva, Coimbra, Portugal

*Critical Care* 2023, **27(S1)**: P007

**Introduction:** The debate about optimal management of patients with COVID-19 ARDS remains, including medical treatment, ventilatory strategies, awake proning and others. COVIP is a multicentric observational study with over 3000 patients under NIV. A substudy by Polok and al. evaluated patients (PTS) ≥ 70 years old. At our intermediate care unit (IU) we used a strategy of high dose corticosteroid started when the work of breathing (WOB) increased, prolonged awake prone positioning (> 12 h) and high CPAP ventilatory strategy. We describe our cohort of ≥ 70 years old NIV PTS and compare it to COVIP substudy results.

**Methods:** Descriptive retrospective study. Data were collected from electronic medical records of 95 COVID-19 PTS aged 70 years old or above under NIV at the IU between September/20 and March/21. Categorical data are presented as frequency (percentage) and were compared using χ2-test. Continuous variables were compared using Mann-Whitney U test. Cohort results were compared with those from Polok et al. COVIP substudy (COVIPss).

**Results:** 95 of PTS were submitted to NIV. Median age was 76 years and 49.5% were male, versus 75.7 and 71.4% in COVIPss. Median admission SOFA score was 4 and CFS was 3 with 14% considered frail (CFS > 5). In COVIPss median SOFA was 5 and 17% of PTS were frail. The preferred mode was CPAP with median maximum pressure of 13. Mean PaO2/fiO2 ratio after start of NIV was 125, 30% < 100. NIV failure occurred in 46.3% versus 74,7% in COVIPss. Our intra-unit mortality was 31.6%. 14 PTS (14.7%) were submitted to invasive mechanical ventilation and 57% of those died. In COVIPss mortality at 30d was 52.9% in NIV and 47.7 in IMV groups.

**Conclusions:** We argue that NIV is a valid option for COVID ARDS management if supported by a multifaceted strategy such as ours, using prone and CPAP for WOB control. We agree with COVIPss authors as NIV trial should be short and intubation promptly if WOB not controlled. Comparison with COVIP substudy NIV failure and mortality results, support our belief.

## P008

### Delayed intubation with high flow nasal cannula in COVID: a comparison between a first and second pandemic wave

#### J Ringoir, A Van Hoorn, J Poelaert

##### UZ Brussel, Intensive Care Unit, Jette, Belgium

*Critical Care* 2023, **27(S1)**: P008

**Introduction:** High flow nasal cannula (HFNC) treatment is an efficient treatment for hypoxemia in acute respiratory distress syndrome (ARDS). Before the COVID pandemic, non-invasive ventilation was associated with higher mortality in ARDS, and early intubation was advocated. We hypothesized that HFNC treatment was more restrictive in the first wave of the COVID pandemic compared to the second wave respecting the pre-COVID conceptual consensus of early intubation in ARDS.

**Methods:** We conducted this retrospective observational single-center study in a tertiary ICU in Brussels during the COVID pandemic. The first flare-up ranged from March to May 2020, and the second flare-up from September to January 2021. All patients with COVID pneumonia and HFNC before intubation were included. We considered a delayed intubation a ROXi < 3.85 at the start of HFNC. ROXi is the ratio of SpO_2_/FiO_2_ to the respiratory rate. The primary outcome was delayed intubation based on the ROXi in the number of days. The secondary outcome was mortality.

**Results:** We included 60 patients in the first wave and 70 in the second wave. The duration of HFNC treatment before intubation was longer during the second wave, based on ROXi < 3.85: 1.6 days versus 2.8 days, *p* < 0.05 (Fig. 1). There was no significant difference in mortality, 18% versus 29%. The length of intubation was similar in both groups. The CCI, SOFA, APACHE III and SAPS II scores were similar in both groups.

**Conclusions:** The duration of HFNC treatment in COVID-19-related ARDS before intubation has significantly been extended in the second pandemic wave. The delayed intubation based on the ROXi was in this study without significantly increased mortality. However, a trend toward higher mortality after prolonged HFNC was seen in the second pandemic wave.

**Figure Figf:**
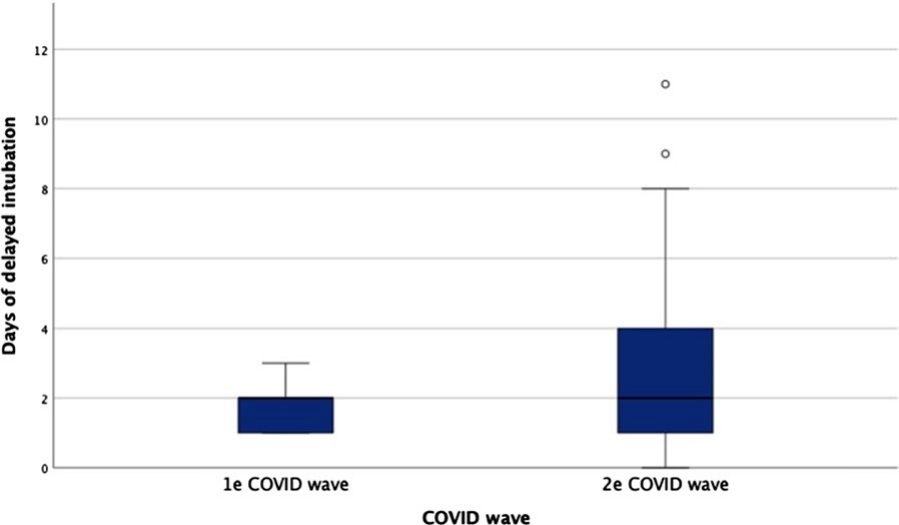
**Fig. 1 (abstract P008)**. Comparison duration HFNC between first and second COVID wave

## P009

### Outcomes following application of high flow nasal cannula and non-invasive ventilation during the second COVID-19 wave in Singapore

#### AS Nazem, ZY Tan, F Khan, D Bruce-Hickman

##### Ng Teng Fong General Hospital, Intensive Care Medicine, Singapore, Singapore

*Critical Care* 2023, **27(S1)**: P009

**Introduction:** COVID-19 pneumonia can result in significant morbidity and mortality in affected individuals. Our audit aims to study the respiratory outcomes of patients with COVID-19 pneumonia following the use of HFNC and NIV during the second wave of the pandemic.

**Methods:** We analysed the outcomes of 94 patients admitted to a tertiary combined HDU/ICU with severe COVID-19 pneumonia requiring non-invasive support between July and December 2021.

**Results:** 94 patients were admitted during the study period. ICU mortality rate was 22% (21/94), total in-hospital mortality was 38% (36/94). HFNC was used as first line respiratory support in 58/94 cases, of which 39.6% required intubation. Of those patients who failed HFNC, time to intubation was significantly higher in those patients who passed away than those who were intubated and survived (mean 6.08 days vs 2.86 days, *p* < 0.05 one sided T-test). In all patients, very late intubation defined as intubation > 5d post admission to ICU, occurred in 6/41 patients, of which the mortality rate was 100%. ROX score performed at 12 h post intubation was unable to discriminate those who succeeded with HFNC and those who required intubation (mean ROX 7.24 vs 7.9, *p* > 0.05). NIV was used in combination with HFNC pre-intubation in 5/23 HFNC cases with 100% mortality rate. Extubation failure rates were low (5/94) and use of tracheostomy was uncommon (4/94; all 4 survived ICU stay, 3 eventually died in hospital).

**Conclusions:** HFNC failure with prolonged use of HFNC and use of multiple non-invasive device strategies before intubation was associated with a high risk of mortality. Conventional measurements of HFNC failure in the form of a 12 h ROX score could not assist the clinician in predicting those patients at risk of HFNC failure.

## P010

### Mortality rate, intensive care unit length of stay and time to orotracheal intubation of COVID-19 patients under different non-invasive ventilatory therapies: retrospective cohort study

#### GM Martins^1^, PLS Leme Silva^2^, APC Pereira Cruz^2^, VM Marques^2^, SM Christovam^2^, IP Prado^2^, CS Dos Santos Samary^2^, PR Rocco^2^, FC Cruz^2^

##### ^1^Barra D’ Or Hospital, Intensive Care Unit, Rio De Janeiro, Brazil, ^2^Barra D’ Or Hospital, Rio de Janeiro Federal University, Rio De Janeiro, Brazil

*Critical Care* 2023, **27(S1)**: P010

**Introduction:** COVID-19 may lead to heterogeneous needs for ventilator therapy, whether oxygen therapy (OT), noninvasive ventilation (NIV), high-flow nasal catheter (HFNC) or their combination (NIV + HFNC). The purpose of the study was to describe, retrospectively, the mortality rate, intensive care unit length of stay (ICU-LOS) and time to orotracheal intubation of COVID-19 patients under OT, NIV, HFNC or combined (NIV + HFNC). A retrospective cohort study was done analyzing official medical data from March 2020 up to July 2021. (CAAE: 52534221.5.0000.5249).

**Methods:** The inclusion criteria were age > 18 years-old, and positive swab test for COVID-19 or computed tomography consistent of COVID-19. The exclusion criteria were hospital LOS less than 3 days, patients whose therapy (OT, NIV, HFNC or NIV + HFNC) lasted less than 48 h, and missing data about the outcome variables. The primary outcome was mortality rate, while secondary outcomes were ICU-LOS and time to orotracheal intubation. Chi-Square test was used to assess mortality rate. The Mann-Whitney U test was applied to assess differences in ICU-LOS and time to orotracheal intubation (*p* < 0.05).

**Results:** Overall, 1371 patients were enrolled. 880, 120, 35, and 148 patients were submitted to OT, NIV, HFNC or NIV + HFNC, respectively. The mortality rates were 8.4%, 29.6%, 22.2%, and 33.2% for OT, NIV, HFNC or NIV + HFNC, respectively (*p* < 0.001). The ICU-LOS was higher in NIV + HFNC (median [IQR] 15 days [16]) than NIV (9 days [10]) and OT (4 days [5], *p* < 0.001). The time to orotracheal intubation was higher in NIV (6 days [6]), HFNC (6 days [4.5]), and NIV + HFNC (6 days [6]) than OT (2 days [4]), *p* < 0.001. Mortality rate and ICU-LOS were higher in those patients requiring the combination of NIV and HFNC.

**Conclusions:** Although the type of ventilator therapy may be associated to increased mortality rate and ICU-LOS, we cannot assure causality due to exploratory nature of the retrospective study, but a marker of severity.

## P011

### Ventilator avoidance among critically ill COVID-19 patients with acute respiratory distress syndrome

#### J Fletcher, P Hahn

##### University of Michigan Health West, Critical Care, Wyoming, USA

*Critical Care* 2023, **27(S1)**: P011

**Introduction:** Misinformation citing mechanical ventilation, not the virus, as causing death in COVID-19 patients with respiratory failure has led to ventilator avoidance (“initial refusal of intubation”) during the pandemic.

**Methods:** Prospective observational cohort study (March 2020–June 2021) evaluating the incidence and significance of “initial refusal of intubation” in patients with critical COVID-19 defined as ARDS requiring > 55% sustained FiO_2_ on high flow nasal canula (HFNC), non-invasive positive-pressure ventilation (NIPPV) or requiring intubation. Outcomes included in-hospital mortality and 1-year modified Rankin Scale (mRS) score. Logistic regression was used to estimate the age and Charlson Comorbidity Index adjusted odds ratio (OR) of in-hospital death. The Wilcoxon rank-sum test was used to evaluate differences in the mRs.

**Results:** The cohort was predominantly non-Latino white (76%), male (65%), unvaccinated (99.4%), mean age of 66, and good pre-COVID-19 functional status (median mRs score of 0). Overall, 315 patients were critically ill due to COVID-19 with an in-hospital mortality of 41.9% (132/315; 95% CI 36–47%). In patients in whom intubation was recommended 39% initially refused (40/102; 95% CI 30–49%). Utilization of HFNC (90%) and NIPPV (72%) were similar between groups, however actual use of mechanical ventilation differed (98.4% in those that did not initially refuse compared to 20% in those that initially refused (*p* = 0.001)). In-hospital mortality was 79.3% (49/62) in those who initially did *not* refuse intubation compared to 77.5% (31/40) in those who refused (adjusted OR 1.3; 95% CI 0.5–.5). The distribution of 1-year mRS was not significantly different between groups (*p* = 1.0) (Fig. 1).

**Conclusions:** Among critically ill patients with COVID-19 associated ARDS, ventilator avoidance was common however, it was not associated with increased in-hospital mortality or a difference in 1-year functional outcome.

**Figure Figg:**
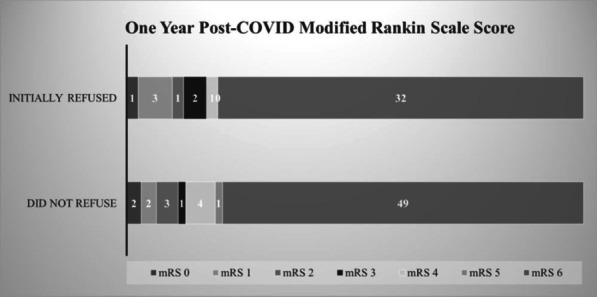
**Fig. 1 (abstract P011)**. Distribution of 1-year mRS scores in participants dichotomized by initial refusal of intubation

## P012

### Diaphragmatic sonographic evaluation in critical illness patient to predict invasive mechanical ventilation use

#### K Suttapanit, P Supatanakij, S Wongkrasunt, S Savatmongkorngul

##### Faculty of Medicine Ramathibodi Hospital, Department of Emergency Medicine, Bangkok, Thailand

*Critical Care* 2023, **27(S1)**: P012

**Introduction:** Diaphragmatic dysfunction is one of the incidents in critically ill patients and associated worsening outcomes. The diaphragmatic function can be evaluated with bedside ultrasound. The diaphragmatic excursion is one of the methods in diaphragmatic ultrasound for detecting diaphragm dysfunction [1–3]. This study aimed to evaluate the performance of the diaphragmatic excursion to predict invasive mechanical ventilation (IMV).

**Methods**: In this prospective study, patients 18 years and older who visited the emergency department (ED) with critical illness were enrolled to measure the right-side diaphragmatic excursion within 10 min after arrival. Multivariable logistic regression and the area under the receiver operating characteristic (AUROC) curve was used to assess the performance of the diaphragmatic excursion to predict IMV.

**Results:** 314 patients were enrolled in this study, and 113 (35.9%) patients required IMV. The diaphragmatic excursion was an independent risk of receiving IMV with adjusted odd ratios 0.08 [95% confidence interval (CI), 0.04–0.17, *p* value < 0.001] and had the area under the receiver operating characteristic (AUROC) 0.850 (95% CI 0.807–0.894). The cut-off of the diaphragmatic excursion was 1.2 cm with a sensitivity of 82.3% (95% CI 74.0–88.8%) and specificity of 78.1% (95% CI 71.7–83.6%) (Table 1). Ventilator days in the diaphragmatic excursion < 1.2 cm (cm) was significantly longer [13 days (interquartile range (IQR); 5.27) versus 5 (IQR; 3, 8), *p* value 0.006].

**Conclusions:** The right-side diaphragmatic excursion had a good performance in predicting IMV in critical illness. The optimal cut-off of the diaphragmatic excursion was 1.2 cm. The benefit of implementing patient management should be assessed in the future.


**References**
Supinski GS et al. Chest. 2018;153:1040–51.Dres M et al. Intensive Care Med. 2017;43:1441–52.Tuinman PR et al. Intensive Care Med. 2020;46:594–605.

**Table Taba:** **Table 1 (abstract P012)**. Prediction performance of diaphragmatic excursion for predicting intubation in critical illness in ED

Diaphragmatic excursion (cm)	Sensitivity	Specificity	Predicted probability
AUROC = 0.850 (95% CI 0.807–0.894)			
≥ 2.00	–	–	3.4% − 0.4 to 7.3%)
1.60–1.99	97.3% (92.4–99.4%)	41.8% (34.9–48.9%)	12.8% (3.2–22.3%)
1.21–1.59	92.2% (85.4–96.3%)	62.2% (55.1–68.9%)	25.6% (12.5–38.6%)
0.91–1.20	82.3% (74.0–88.8%)	78.1% (71.7–83.6%)	58.5% (45.2–71.8%)
≤ 0.90	54.9% (45.2–64.2%)	89.1% (83.9–93.0%)	73.8% (64.4–83.2%)

## P013

### A new simple method to identify the activation of the expiratory muscles. Index based on the determination of expiratory time constant

#### JF Martínez Carmona^1^, C Joya Montosa^1^, J Luna Castro^1^, MJ Delgado Amaya^1^, A Rodríguez Carmona^2^, J Castaño Pérez^3^, G Besso Centeno^4^, M Rodríguez Delgado^5^, JA Benítez Lozano, in memoriam^1^, JM Serrano Simón^6^

##### ^1^Hospital Regional Universitario de Málaga, Intensive Care Unit, Málaga, Spain, ^2^Hospital El Carmen, Intensive Care Unit, Mendoza, Argentina, ^3^Hospital Virgen de las Nieves, Intensive Care Unit, Granada, Spain, ^4^Clínica del Aconcagua, Intensive Care Unit, Villa Mercedes San Luis, Argentina, ^5^Hospital La Merced, Intensive Care Unit, Osuna, Sevilla, Spain, ^6^Hospital La Merced, Osuna, Sevilla and Hospital Reina Sofía, Córdoba, Intensive Care Unit, Cordoba, Spain

*Critical Care* 2023, **27(S1)**: P013

**Introduction:** We hypothesized the expiratory time constant (t_E_) may be used to provide determinate expiratory muscle activation (EMA) in mechanically ventilated patients. Determination of t_E_ by the quotient between exhaled tidal volume (Vt) and flow rate values between 0.10 and 0.5 s, during passive lung emptying, are linear fit. However, in EMA, t_E_ obtained from exhaled Vt at 66% is dissociated from t_E_ determined by 25–75% of the expiratory flow-volume curves (t_E_ 25–75%). EMA increases the pleural pressure, with partial airway pressure (Paw) collapse and expiratory flow limiting. Our purpose was to evaluate the index t_E_25–75%/t_E_66%, to identify EMA.

**Methods:** A retrospective study, made by Córdoba University Hospital (January-May 2020), using available recordings of flow, Paw, esophageal (Pes) and gastric pressure (Pgas) of consecutive patients admitted to ICU, during partially assisted mechanical ventilation. Index t_E_ was obtained cycle by cycle, during 60 min, by monitoring device and personal software. Sensitivity, specificity and ROC curve was carried out versus Pgas (gold std).

**Results:** 41 patients were included. 19 presented EMA: 64.16 (± 11.07) years. 80% male. 6 Pneumonia, 2 Single lung transplant, 3 Neurological, 2 Pancreatitis, 1 Trauma, 3 Cardiac and 2 abdominal surgery. Days on ventilator: 7.5 (1.5–9.5). Ers 19.06 (± 6.97) cmH_2_O/L. Rrs 13.50 (± 9.93) cmH_2_O/L/s. Peepi 7.66 (± 1.58) cmH_2_O. ΔPgas exp 9.67 (± 4.94) cmH_2_O. Settings: 6 A/C (flow 40–60 L/min), 13 PSV (10 ± 4)/PEEP5 cmH_2_O. Index t_E_: Passive exhalation 1.07 (± 0.11), EMA 2.18 (± 0.84)sec, (*p* < 0.001). Sensitivity and specificity (95% CI), threshold > 1.55: 0.84 (0.72–0.92), 0.95 (0.86–0.99), respectively; PPV 0.94, VPN 0.87. ROC curve: AUC = 0.98. A cut-off ≥ 1.4 was associated with the best index for success, sensivity of 0.95 (95% CI 0.85–0.99), specificity of 0.90 (95% CI 0.79–0.95), PPV 0.89, NPV 0.95.

**Conclusions:** Our index t_E_ allows us to identify with accuracy the expiratory muscle activity, only by the flow signal, regardless of underlying disease.

## P014

### Early diaphragm dysfunction in critically ill patients under invasive mechanical ventilation: preliminary data from a prospective observational study

#### V Silva^1^, C Pação^2^, D Palacios^3^, M Alves^2^, G Nobre de Jesus^3^, J Santos Silva^3^

##### ^1^Hospital de Santa Maria, Centro Hospitalar Universitário Lisboa Norte, EPE, Intensive Care Department, Lisboa, Portugal, ^2^Faculdade de Medicina de Lisboa, Lisboa, Portugal, ^3^Hospital de Santa Maria, Centro Hospitalar Universitário Lisboa Norte, EPE, Lisboa, Portugal

*Critical Care* 2023, **27(S1)**: P014

**Introduction:** Diaphragmatic dysfunction (DD) is a keystone factor in difficult weaning from invasive mechanical ventilation (IMV). Diaphragmatic ultrasound (DUS) is the preferred method for the evaluation of diaphragm function in the ICU setting, namely through the diaphragm thickening fraction (DTF). However, its potential role in the decision-making process of mechanical ventilation weaning is yet to be established. We aimed to assess the incidence of early DD and its role as a predictor of prolonged IMV.

**Methods:** We conducted a prospective, non-interventional study in a universitary hospital ICU. Adult patients subject to at least 48 h of IMV were consecutively enrolled. Exclusion criteria was a prior period of IMV in the past 3 months. DUS was performed at 48 h of IMV. End-inspiratory and end-expiratory diaphragm thickness were measured using M-mode, with a high-frequency linear probe placed at the zone of apposition of the diaphragm. The mean values of 3 measurements were used to calculate DTF. Interobserver measurement variability was not evaluated.

**Results:** 32 patients were included. 34.4% were female, average age was 59.9 years. Mean SAPS II and SOFA at admission were 52.87 and 7.84, respectively. Mean DTF was 21.10% (± 14.59). Average IMV duration was 10.13 days (± 8.43). 75% of patients had DD at 48 h of IMV. A weak negative correlation (r < − 0.1) was observed between DTF, days of endotracheal intubation (p value 0.609) and days of IMV (p value 0.756). Using DTF cut-off values of 20% and 30%, DTF at 48 h of IMV did not correlate with prolonged IMV (p value of 0.427 and 0.704, respectively).

**Conclusions:** In our preliminary data, there was a high prevalence of DD at 48 h of IMV, as suggested in previous literature. DD at 48 h when measured through DTF did not seem to predict prolonged IMV. DUS is well-established for diaphragm functional assessment, but further research is needed to clarify its application in clinical practice.

## P015

### The effect of differing loading conditions on ultrasound assessment of diaphragm function

#### IS Morris, EC Goligher

##### University Health Network, Department of Adult Critical Care Medicine, Toronto, Canada

*Critical Care* 2023, **27(S1)**: P015

**Introduction:** Diaphragm dysfunction is a common cause of failure to wean from invasive mechanical ventilation [1]. Ultrasound assessment of diaphragm activity has been proposed as a potential noninvasive method to assess diaphragm function and force generation [2]. The influence of varying physiological conditions on these measurements has not been systematically evaluated.

**Methods:** Healthy adult volunteers underwent esophageal and gastric balloon placement to measure transdiaphragmatic pressure (Pdi). Diaphragm thickening fraction (TFdi) and diaphragm excursion (EXdi) were measured under varying resistive load (none, marked, complete airway occlusion), varying postural load (semi-recumbent vs. supine) and varying thoracoabdominal motion (abdominal- vs. rib cage-predominant inspiration). The influence of physiological conditions on these measurements was analyzed by linear mixed effect regression.

**Results:** Nine participants were included. Pdi swing increased with resistive load (*p* < 0.001) and decreased in the supine position (*p* < 0.001) (Table 1). By contrast, TFdi did not change significantly when marked resistance was applied (*p* = 0.44) but decreased with complete airway resistance (*p* = 0.055). TFdi also decreased in the supine position (*p* = 0.004) and with rib cage-predominant inspiration (*p* < 0.001). EXdi decreased with increasing resistance (*p* < 0.001) and supine position (*p* = 0.013).

**Conclusions:** Changing physiological conditions has differential effects on Pdi, TFdi, and EXdi. Position, mechanical load, and thoracoabdominal motion should be considered when interpreting these parameters to assess diaphragm function. TFdi is relatively less affected by physiological conditions than EXdi.


**References**
Dres M et al. Intensive Care Med 2017;43:1441–1452.Goligher EC et al. Am J Respir Crit Care Med 2020;202:950–961.

**Table Tabb:** **Table 1 (abstract P015)**. The effect of differing loading conditions on measures of diaphragm function

	Pdi cmH_2_O: mean (SEM); *p* value	TFdi %: mean (SEM); *p* value	EXdi cm: mean (SEM); *p* value
Resistive load: none (reference)	53 (2.0)	52 (6.0)	2.9 (0.21)
Resistive load: marked	84 (2.8); < 0.001	57 (6.4); 0.44	1.4 (0.12); < 0.001
Resistive load: complete	87 (3.2); < 0.001	42 (4.8); 0.055	1.2 (0.10); < 0.001
Position: semi-recumbent (reference)	82 (1.54)	56 (5.7)	2.0 (0.13)
Position: supine	67 (2.18); < 0.001	45 (3.5); 0.004	1.5 (0.18); 0.013
Thoracoabdominal motion: abdomen predominant (ref)	n/a	115 (15.1)	n/a
Thoracoabdominal motion: rib cage-predominant	n/a	60 (7.5); < 0.001	n/a

Pdi, transdiaphragmatic pressure; TFdi, diaphragm thickening fraction; EXdi, diaphragm excursion; SEM, standard error of the mean); reference, reference category; n/a, not applicable (measured)

## P016

### Temporary diaphragm pacing electrodes to decrease mechanical ventilation and improve diaphragm function during cardiac procedures

#### RPO Onders, M Elmo, N Carl

##### University Hospitals Cleveland Medical Center, Department of Surgery, Cleveland, USA

*Critical Care* 2023, **27(S1)**: P016

**Introduction:** Studies have shown diaphragm pacing (DP) prevents ventilator induced diaphragm dysfunction (VIDD) secondary to mechanical ventilation (MV) improving extubation and facilitates injured phrenic nerves recovery. We report our experience utilizing a temporary DP system during cardiac procedures.

**Methods:** This is a retrospective analysis of prospective IRB approved databases of non-randomized interventional experiences at a single institution. Subgroup analysis was then limited to patients implanted with the temporary DP system (TransAeris, Synapse Biomedical) during cardiac procedures. Prior to sternotomy closure, the pleural space is opened, two electrodes are placed in each hemi- diaphragm. The electrodes also record diaphragm burst electromyography (dEMG). Once in the intensive care unit DP ensued using a disposable multi-channel stimulator generating a fused diaphragm contraction preventing VIDD.

**Results:** From 1/1/2020 to 6/30/22 252 patients were implanted with either the chronic long term DP (NeuRx) or the temporary DP system. 21 patients were implanted with the temporary DP system during a cardiac procedure. All had risk factors that predict prolonged MV including: prior open cardiac surgery, LVEF less than 30%, COPD, history of CVA, or pre-operative intra-aortic balloon pump In 3 patients, there was rapid weaning with bilateral dEMG and no stimulation occurred. The remaining 18 patients underwent diaphragm stimulation to facilitate weaning and maintain diaphragm strength. In patients with pre-existing unilateral dysfunction, DP prevented paradoxical diaphragm movement and associated sleep dysfunction. There were no adverse events related to electrode implantation, stimulation or subsequent removal.

**Conclusions:** Prolonged MV after cardiac surgery leads to significant morbidity, mortality and cost. DP was safely used to wean from MV, prevent VIDD and the electrodes can identify and improve recovery of phrenic nerve injuries.

## P017

### Comparison of ultrasound assessment for diaphragmatic workload during spontaneous breathing trial between tube compensation and pressure support ventilation

#### N Kulkanokwan^1^, S Morakul^2^, C Pisitsak^2^, W Mongkolpun^3^, P Theerawit^1^

##### ^1^Faculty of Medicine, Ramathibodi Hospital, Mahidol University, Division Critical Care Medicine, Department of Medicine, Bangkok, Thailand, ^2^Faculty of Medicine, Ramathibodi Hospital, Mahidol University, Department of Anesthesiology, Bangkok, Thailand, ^3^Siriraj Piyamaharajkarun Hospital, Siriraj Hospital, Mahidol University, Department of Critical Care Medicine, Bangkok, Thailand

*Critical Care* 2023, **27(S1)**: **P017**

**Introduction:** Spontaneous breathing trail (SBT) with pressure support ventilation (PSV) might provide over assistant while SBT with T-pieces may increase workload. Therefore, tube compensation (TC) might be an optional SBT mode but its effect on patients’ effort has not been well shown. This study aimed to compare the patient’s effort, measured by diaphragm ultrasonography, between TC and PSV and PEEP during SBT.

**Methods:** This was a crossover study. Patients requiring mechanical ventilation ≥ 48 h with the weaning requirements were included. TC (30 min) was performed and PSV 5 cmH_2_O (30 min) was done after TC in one group. In the other group, PSV was performed before TC. During the washout period, baseline ventilation was resumed until tidal volume, respiratory rate, and heart rate were return to baseline. Before and immediate after 30 min of PSV and TC, diaphragmatic workload parameters were assessed by relative change (%) of diaphragm thickness fraction (∆DTF), diaphragm excursion (∆DE) and tissue Doppler imaging (TDI) parameters of peak contraction velocity (∆PCV), peak relaxation velocity (∆PRV), velocity–time integral (∆VTI) and TDI-derived maximal relaxation rate (∆TDI-MRR).

**Results:** For our results, the interaction between period and treatment was significant. So, we used only the first period in our interpretation. Among 48 patients who met the inclusion criteria, nobody failed SBT before 30 min. ∆DTF was significant greater with TC than with PSV ([65.84 (17.52, 83.38)] % vs [0.49 (−40.85, 30.47)] %, *p* < 0.01, respectively). ∆DE was significant greater with TC [14.75(0.00,36.45)] than with PSV [−9.47(−33.14, −1.01)] % (*p* < 0.01). Change of TDI variables, except ∆TDI-MRR, did not differ between TC and PSV (Table 1).

**Conclusions:** SBT with TC was associated with higher diaphragm effort than with PSV with the same PEEP.

**Table Tabc:** **Table 1 (abstract P017)**. Ultrasound-measured diaphragmatic parameters in the tube compensation and the low-pressure support periods during spontaneous breathing trial

Variable	PSV	TC	Difference between treatments (95% CI)	*p* value
∆DTF (%)	0.51 (−40.85, 30.46)	54.24 (10.59, 70.25)	61.28 (23.27, 99.29)	0.002
∆DE (%)	−9.47 (−33.14, to 1.01)	14.75 (0.00, 36.45)	37.42 (19.32, 55.53)	< 0.001
∆PCV (%)	0.67 (−8.63, 14.21)	2.56 (−9.38, 16.02)	5.31 (−6.54, 17.17)	0.380
∆PRV (%)	2.15 (−21.93, 15.75)	16.43 (−7.83, 31.26)	12.89 (−9.10, 34.87)	0.250
∆VTI (%)	11.47 (−8.23, 23.36)	−4.92 (−24.58, 12.04)	−17.81 (−37.38, 1.75)	0.074
∆TDI-MRR (%)	−9.48 (−37.70, 31.67)	24.09 (−24.23, 58.77)	44.13 (0.11, 88.15)	0.049

## P018

### A hemodynamic echocardiographic evaluation predicts prolonged mechanical ventilation in septic patients

#### T Giraldi^1^, JR Matos-Souza^2^, D Cecilio-Fernandes ^3^, T Santos^3^

##### ^1^University of Campinas, Emergency Medicine, Campinas, Brazil, ^2^University of Campinas, Cardiology, Campinas, Brazil, ^3^University of Campinas, School of Medical Sciences, Campinas, Brazil

*Critical Care* 2023, **27(S1)**: P018

**Introduction:** Prolonged mechanical ventilation (PMV) is common among critically ill septic patients and leads to serious adverse effects. Transthoracic echocardiography (TTE) is an efficient tool for the assessment of septic shock. Our study investigated the relationship between TTE parameters and PMV in mechanically ventilated septic shock patients.

**Methods:** TTE was performed in the first 24 h of intensive care unit admission, acquiring data on cardiac output (CO), cardiac index (CI), s′ wave (s′), E wave (E), e′ wave (e′) and E/e′ ratio. We compared data on patients who met the criteria for PMV with data on patients who did not.

**Results:** Sixty-four patients were included, 26 of whom met the criteria for PMV. CO, CI and s′ were higher in PMV patients (5.49 vs. 4.20, *p* = 0.02; 2.95 vs. 2.34, *p* = 0.04; and 12.56 vs. 9.81, *p* = 0.01, respectively). Patients with PMV had greater mean CO, CI and s′ (Fig. 1). The area under the ROC curve for CO in predicting PMV was 0.707 (*p* < 0.01). A cutoff value of 4.82 L/min was associated with the highest diagnostic accuracy and predicted PMV with a sensitivity of 61.5% and a specificity of 78.9%; with positive likelihood ratio (PLR) value of 2.91 and a negative likelihood ratio (NLR) of 0.49. CI had an area under the ROC curve of 0.69 (*p* = 0.01). A cutoff value of 2.54 L/min/m^2^ was associated with the highest diagnostic accuracy and predicted PMV with a sensitivity of 69.2%, a specificity of 71.1%; PLR of 2.39 and NLR of 0.43. The s′ wave had an area under the ROC curve of 0.679 (*p* = 0.03). A cutoff value of 13.85 cm/s was associated with the highest diagnostic accuracy and predicted PMV with a sensitivity of 42.1% and a specificity of 90.6%; it provided a PLR of 4.48 and a NLR of 0.36.

**Conclusions:** Increased values of CO, CI and s′ wave on ICU admission were related to PMV. The s′ correlated with CI and could estimate systemic vascular resistance. This echocardiographic hemodynamic protocol could be added to the initial assessment of mechanically ventilated septic patients.

**Figure Figh:**
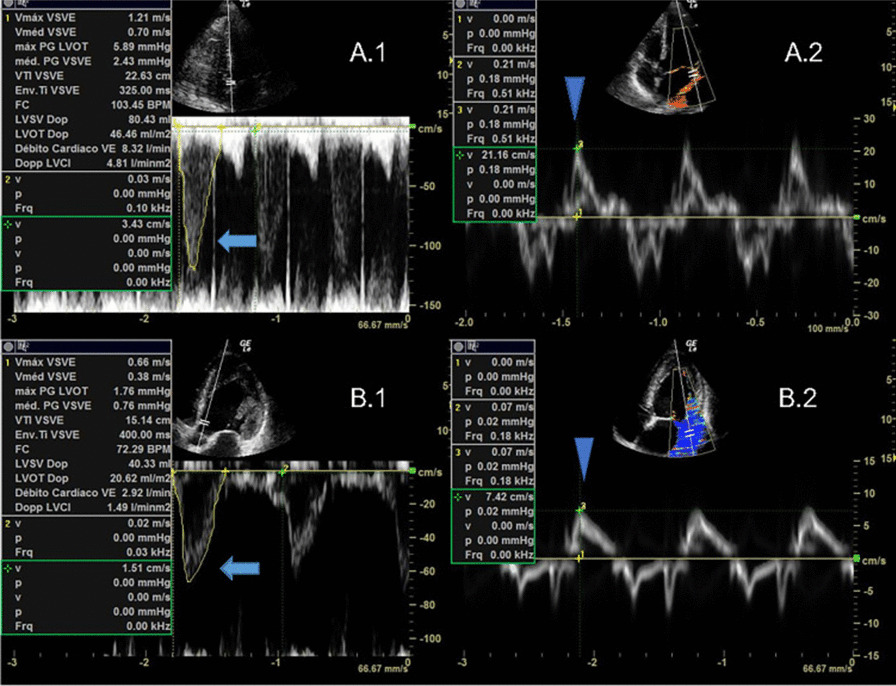
**Fig. 1 (abstract P018)**. Examples of CI and s′-wave measurements in a patient with PMV (**A**) and in one without PMV (**B**). The left ventricular outflow tract ventricular-time integral (LVOT VTI) (arrows) is illustrated in (**A.1**) and **(B.1**), and tissue Doppler peak systolic velocity (s′ wave) (arrowheads) in (**A.2**) and (**B.2**). Cardiac index values for patients A and B were, respectively, 4.81 and 1.49 L/min/m^2^, and s′ wave values for patients A and B were 21.16 and 7.42 cm/s, respectively

## PO19

WITHDRAWN

## P020

### Metabolic acidosis in pre-intubation predicts post-intubation hypotension during emergency airway management

#### M Suga, T Nishimura, S Maemura, T Ochi, S Ishihara.

##### Hyogo Emergency Medical Center, Department of Emergency and Critical Care Medicine, Kobe, Japan.

*Critical Care* 2023, **27(S1)**: P020

**Introduction:** Post-intubation hypotension (PIH) in the emergency department (ED) endotracheal intubation is an adverse event that is associated with poor outcomes. Metabolic acidosis in a pre-intubated scenario represents a significant challenging situation and a risk for patient deterioration. However, little is known about the relationship between metabolic acidosis and PIH. We aimed to evaluate the association between pre-intubation metabolic acidosis and PIH during emergency airway management.

**Methods:** We conducted a single-center, retrospective observational study at an urban ED from Nov 2016 to Mar 2022. Patients who required emergent intubation at ED were included. Patients younger than 18 years, with systolic blood pressure(sBP) < 90 mmHg, received vasopressors or mechanical circulatory support before intubation were excluded. The primary endpoint was the incidence of PIH, defined as sB*P* < 90 mmHg or the administration of any vasopressors within 30 min after intubation. Multivariable logistic regression models were used to evaluate the association between PIH and metabolic acidosis, adjusted with known risk factors (shock index (SI) before intubation, age, gender, use of non-depolarizing neuromuscular blocking agents, and intubation for respiratory failure).

**Results:** In total, 257 patients were enrolled in the current study, and 87 patients (33.9%) developed PIH. The median age was 67 years [interquartile range (IQR): 49–76], and 119 (46.3%) of 257 patients had metabolic acidosis. Compared to patients without metabolic acidosis in pre-intubation, those with metabolic acidosis showed a significantly higher incidence of PIH (51.2% vs. 18.8%, *p* < 0.001). In the patients overall, multivariable logistic regression demonstrated that age (odds ratio [OR] 1.03; 95%confidence interval [CI] 1.0–1.1), metabolic acidosis (OR, 3.4; 95% CI 1.9–6.3), shock index (OR 8.1; 95% CI 2.4–27.6) was significantly related to PIH.

**Conclusions:** Metabolic acidosis in pre-intubation could be a predictor of PIH at the ED.

## P021

### Intubation times with the bougie versus stylet in the immobilized cervical spine: a randomized trial

#### A Hazarika^1^, D Namratha^1^, D Jain^1^, N Bhatia^1^, A Ray^1^, S Singh Dhatt^2^, M Karthigeyan^3^, C Ahuja^4^

##### ^1^Postgraduate Institute of Medical Education and Research, Department of Anaesthesia & Intensive Care, Chandigarh, India, ^2^Postgraduate Institute of Medical Education and Research, Dept of Orthopaedics, Chandigarh, India, ^3^Postgraduate Institute of Medical Education and Research, Dept. of Neurosurgery, Chandigarh, India, ^4^Postgraduate Institute of Medical Education and Research, Dept. of Radiology, Chandigarh, India

*Critical Care* 2023, **27(S1)**: P021

**Introduction:** In cervical spine injury patients, the neck is stabilized by neck collar and traction, limiting neck movements. This leads to failure in aligning the laryngeal, pharyngeal and oral axes, making the visualization of the larynx difficult. Endotracheal intubation (ETI) in patients with an immobilized cervical spine is often challenging, urging the use of airway adjuncts like bougie and stylet and a video-laryngoscope (VL). Owing to the scarcity of comparative literature, we aimed to compare the intubation characteristics when using either a bougie or a stylet with a VL in these patients.

**Methods:** This randomized controlled study involved eighty-six adult ASA I/II patients with cervical spine immobilized with a collar or traction, scheduled for cervical spine surgery. ETI was performed with the C-MAC VL, assisted with bougie (Group ETB) or stylet (Group ETS). The primary outcome was time to successful ETI. First-attempt success (FAS) rate, overall successful ETI, cervical spine motion detected using fluoroscopy and complications were secondary outcomes. Any movement of the atlanto-occipital joint during ETI was noted by fluoroscopy image at two time points; T1-at the neutral position (during bag and mask ventilation after induction) and T2-point of insertion of the ETI through the glottis aperture. Fluoroscopy data was analysed only for successful ETI.

**Results:** The time for ETI in group ETB was 52.38 ± 20.85 s (n = 43), and in group ETS was 52.39 ± 32.85 s (n = 43), *p* = 0.958. There was no significant difference in FAS rate, overall success of intubation or cervical spine movement between the groups (Fig. 1). No complications were encountered.

**Conclusions:** In patients with an immobilized cervical spine, there was no significant difference in the intubation times when comparing the bougie and the stylet. The FAS rate was also similar in both groups with minimal motion at C1, C2. Both bougie and stylet are equally useful adjuncts when used with a VL, while intubating patients in whom neck movements are restricted.

**Figure Figi:**
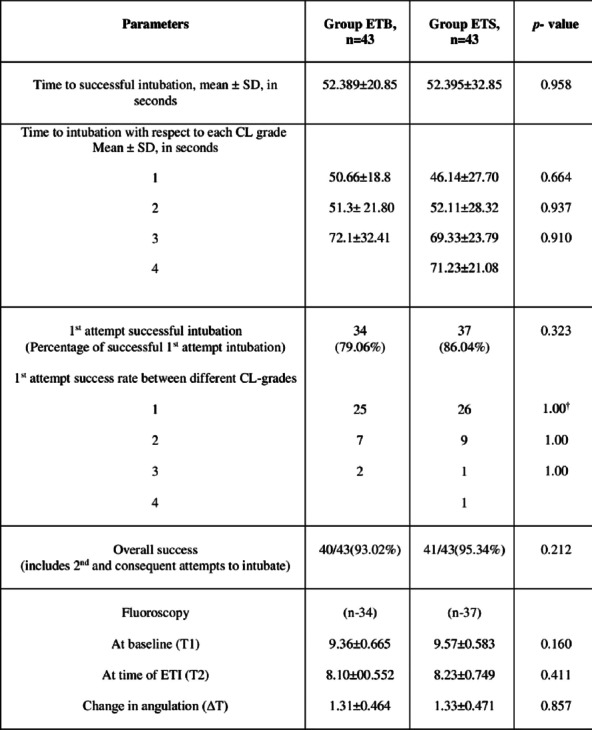
** Fig. 1 (abstract P021)**. The overall time to successful intubation, time to successful 1st attempt intubation, overall success rate and change in angulation between cervical vertebra C1 and C2 as measured during intubation by fluoroscopy

## P022

### Sealing performance of taper shaped cuff for pediatric use

#### P Lichtenthal^1^, D Wade^2^, U Borg^2^

##### ^1^University of Arizona, Department of Anesthesiology, Tucson, USA, ^2^Medtronic, Respiratory Interventions, Boulder, USA

*Critical Care* 2023, **27(S1)**: P022

**Introduction:** Studies have demonstrated that current barrel-shaped high volume, low-pressure endotracheal tube (ETT) cuffs allow aspiration of fluids through channels formed in the cuff [1]. Leakage around the ETT cuff is known to contribute to microaspiration [2]. A tapered shaped ETT cuff, in the adult population, has demonstrated less fluid leakage [3] and possibly a decrease in tracheal damage [1]. A taper shaped ETT cuff, intended for the pediatric population, was recently introduced. The object of this study was to compare fluid sealing performance of a taper shaped ETT cuff (Falcon II, Medtronic, Boulder, CO, USA) to that of barrel shaped ETT cuffs (Shiley™ Hi-Lo, Medtronic, Boulder CO USA; MicroCuff, Avanos) using excised animal tracheas.

**Methods:** Excised rabbit tracheas, including larynx, were utilized. Tracheas were wrapped in saline soaked gauze to prevent drying and suspended by a laboratory clamp stand. Prior to intubation, the cuffs were inflated to 50 cmH2O to eliminate any wrinkles and confirm integrity. After placement in the trachea, the cuffs were inflated to 25 cmH_2_O and monitored continuously. Saline (0.5–1.0 ml) was placed on top of the ETT cuff and leak volume was collected at 1, 5 and 10 min using a beaker placed on a digital scale. The smallest ETT size available for each device tested was employed for testing (2.5 mm for taper shaped ETT cuff and 3.0 for barrel shaped ETT cuffs).

**Results:** No statistically significant differences in leaked saline amount were observed between the taper shaped ETT cuff and barrel shaped ETTs.

**Conclusions:** The findings of the study suggest comparable performance between the taper shaped ETT cuff and barrel shaped ETT cuff, when assessing leak volume using a bench top study design. Further studies are required to elucidate the possibility of decreased injuries to vocal folds and trachea as a result of cuff shape.


**References**
Chang JE et al. Anesth Analg. 2017;125:1240–1245.Pozuelo-Carrascosa DP et al. Eur Respir Rev 2020;29:190,107.D’haese J et al. Acta Anaesthesiol Scand. 2013;57:873–80.


## P023

### Benefits of fully closed loop ventilation modes in patients with body mass index > 35 kg/m^2^

#### R Komnov, A Eremenko, A Alferova, D Riabova, P Titov, A Urbanov, M Fominukh, S Gerasimenko

##### Petrovsky Russian Research Center of Surgery, Post Cardiac Surgery Intensive Care unit, Moscow, Russian Federation

*Critical Care* 2023, **27(S1)**: P023

**Introduction:** In recent years, there is an active automation of processes in medicine and robotic (Intellectual) ventilation modes are being actively introduced into the daily practice of intensive care units. These modes allow for automated respiratory support, minimizing clinician involvement.

**Methods:** In this randomized trial 32 adult patients with BMI > 35 were included. Half of them were ventilated with INTELLiVENT-ASV® mode and 16 with conventional ventilation modes after uncomplicated cardiac surgery. Hamilton G5 ventilators were used and 8 physicians were involved into the study. Care of both groups was standardized, except modes of postoperative ventilation. We compared:The physician’s workload, through accounting number of manual ventilator settings and time they spent near the ventilator in every group;Safety of respiratory support by considering driving pressure and mechanical power during mechanical ventilation, positive end expiratory pressure, FiO_2_ and tidal volume level during all phases of respiratory support;Duration of tracheal intubation in the ICU.

**Results:** In the Intellivent group the number of manual ventilator settings and physician’s time spent near the ventilator before tracheal extubation were significant lower: 1 (0–2) versus 6 ± 2, and 64 ± 17 s versus 36 ± 90 s respectively (*p* < 0.0001 in both cases). Intellivent-ASV mode was more protective compared to conventional mode through significant reduction in the driving pressure, mechanical power, tidal volume, FiO_2_ and PEEP levels, but without difference between PaO_2_/FiO_2_ ratio (Table 1). There were no significant differences in the duration of respiratory support in the ICU: 276 ± 103 min (Intellivent group) versus 300 (225–175) min (control) (*p* = 0.2427).

**Conclusions:** Intellivent-ASV® mode in patients with BMI > 35 after uncomplicated cardiac surgery allows to personalize respiratory support, provides more protective mechanical ventilation and reduces the physician’s workload.

**Table Tabd:** **Table 1 (abstract P023)**. Main outcomes

Variable	Intellivent—ASV group (n = 16)	Control group (n = 16)	*p*
BMI (Body mass index); (CI mean 95%)	39 ± 2 (37–40)	38 ± 2 (37–39)	0.5591
Ratio PaO_2_/FiO_2_; (CI mean 95%)	320 ± 37 (300–339)	323 ± 52 (295–351)	0.8317
FiO_2_	29 (28–30)	35 (30–40)	0.0001
Tidal volume (Vt), ml/kg/PBW (CI mean 95%)	6.4 ± 0.5 (6.1–6.6)	9 ± 1.3 (8.3–9.7)	0.0001
∆P (driving pressure), cmH_2_O (CI mean 95%)	7 ± 1 (7–8)	9 ± 2 (8–10)	0.0039
PEEP, cmH_2_O; (CI mean 95%)	9 ± 2 (8–10)	11 ± 2 (10–12)	0.0078
Mechanical power, J/min; (CI mean 95%)	10 ± 3 (8–12)	15 ± 5 (12–18)	0.0052

## P024

### Automatic close-loop ventilation for patients with traumatic brain injuries

#### S Vosylius^1^, A Ramaskaite^2^, J Semenkovaite^2^, E Januskeviciute^3^, J Meskauskiene^1^, N Aliukoniene^1^, A Balakirev^4^

##### ^1^Republican Vilnius University Hospital, ICU, Vilnius, Lithuania, ^2^Vilnius University, Faculty of Medicine, Vilnius, Lithuania, ^3^Vilnius University, Clinic of Anesthesiology and Intensive Care, Vilnius, Lithuania, ^4^Acrux Cyber Services, Acrux Cyber Services, Vilnius, Lithuania

*Critical Care* 2023, **27(S1)**: P024

**Introduction:** Automatic close-loop mode of mechanical ventilation compared to traditional modes provides ventilated patients with monitoring parameters within safety limits and reduces the risk of ventilator induced lung injury. The aim of this study was to assess the ventilator parameters for the patients with traumatic brain injury under adaptive support ventilation (ASV).

**Methods:** This prospective observational study was conducted in the intensive care unit (ICU) of Republican Vilnius University Hospital. We included 11 patients ventilated at least 48 h with ventilators Hamilton S1 using INTELLiVENT®-ASV mode applying automatic control of minute volume, positive end expiratory pressure, and FiO_2_. IntelliSync + tool was applied for better synchronization between the inspiratory and expiratory times of patient respiratory system and ventilator. The patients were continuously monitored using Acrux DeepBreath software. The study protocol was approved by the regional Vilnius bioethics committee (2021/9-1380-851).

**Results:** A total duration of mechanical ventilation for all patients was 1541 h (from 62 to 289). A total of 1,847,197 breath cycles were analysed after exclusion of 2.3% of cycles defined as artefacts related to nursing, and therapeutic interventions. 95.2% of all breath cycles (for single patient: min. 86.8–max. 98.8%) meet criteria for lung protection (Pmax ≤ 30cmH_2_O, VT/IBW ≤ 12 ml/kg). Median of monitoring parameters: MVe = 9.8 l/min, VT = 572 ml, respiratory rate −17 breaths/min, VT/IBW = 7.5 ml/kg, Pmax = 19 cmH_2_O, PEE*P* = 5 cmH_2_O). Gas exchange was optimal: PetCO_2_ = 36 cmH_2_O, SpO_2_ = 98% with FiO_2_ = 35%. Monitoring parameters from ventilators during time scale were stable over 48 h (Table 1).

**Conclusions:** The patients with traumatic brain injury ventilated using automatic ventilation mode (INTELLiVENT®-ASV and IntelliSync + tool) during the first 48 h had optimal gas exchange and monitoring parameters within safety limits for lung protection.

**Table Tabe:** **Table 1 (abstract P024)**. Monitoring ventilator parameters during time scale over 48 h

Hours	Pmax	Pinsp	PEEP	VT/IBW
1–8	18	12	5	7.2
9–16	19	13	5	7.4
17–24	18	12	5	7.4
25–32	20	13	5	7.6
33–40	20	14	5	7.7
41–48	20	13	5	7.9

## P025

### Pressure-controlled versus volume-controlled ventilation: is there a difference in ventilation homogeneity?

#### T Sebrechts^1^, P Vets^1^, T Bleeser.^2^

##### ^1^ZNA Middelheim, Intensive care unit, Antwerp, Belgium, ^2^UZ Leuven, Department of Anesthesiology, Leuven, Belgium

*Critical Care* 2023, **27(S1)**: P025

**Introduction:** During mechanical ventilation, volume-controlled ventilation (VCV), which performs a constant inspiratory flow, may create a more homogenous ventilation compared to pressure-controlled ventilation (PCV), which performs a decelerating inspiratory flow. Ventilator associated lung injury can be reduced by a more homogenous ventilation. Two parameters concerning ventilation homogeneity can be conducted by using electric impedance tomography (EIT). Regional ventilation delay (RVD) displays regional varying time constants [1]. Intratidal variation (ITV) describes impedance changes [2].

**Methods:** In this observational prospective study, included patients were intubated and sedated because of a respiratory disease, and had no signs of spontaneous breathing. EIT measurements were performed during PCV first, followed by VCV during five minutes each. Respiratory frequency, inspiratory time and PEEP were held constant. In RVD, five regions of interests were investigated (global, ventral, midventral, middorsal and dorsal). In ITV, there were four regions (ventral, midventral, middorsal and dorsal). Wilcoxon signed-rank test was used.

**Results:** Five patients were included. ITV measurements did not show any statistically significant or clinically relevant differences between PCV and VCV. RVD measurements showed a trend to a higher RVD in the middorsal part in every patient when VCV was applied [median(IQR) + 0.95% (+ 0.84; + 1.04), *p* = 0.0625]. Other results were neither statistically significant, nor clinically relevant.

**Conclusions:** There was no evidence for relevant differences in ventilation homogeneity between PCV and VCV, as measured by ITV. For RVD there was a trend that only the middorsal part, against expectations, experiences a less homogenous ventilation during VCV, with no relevant differences in other regions. Additional research with a larger patient group is required.


**References**
Muders T et al. Yearbook of Intensive Care and Emergency Medicine 2009, pp 405–412.Lowhagen K et al. Minerva Anestesiol 2010;76:1024–35.


## P026

### Ventilator management and risk of air leak syndrome for patients with COVID-19 pneumonia

#### N Miyake, Y Igarashi, R Nakae, T Mizobuchi, T Masuno, S Yokobori

##### Nippon Medical School Hospital, Emergency and Critical Care Medicine, Tokyo, Japan

*Critical Care* 2023, **27(S1)**: P026

**Introduction:** Coronavirus disease 2019 (COVID-19) pneumonia is reportedly associated with air leak syndrome (ALS), including mediastinal emphysema and pneumothorax, and has a high mortality rate. In this study, we compared values obtained every minute from the ventilator to clarify the relationship between ventilatory management and the risk of developing ALS.

**Methods:** This was a single-center, retrospective, observational study for a 21-months period. Patient background, ventilator data, and outcomes were collected from adult patients with COVID-19 pneumonia on ventilator-assisted respiratory management. The primary outcome was the development of ALS within 30 days of starting ventilator management.

**Results:** Of the 105 patients, 14 (13%) developed ALS. The mean positive-end expiratory pressure (PEEP) difference was 0.33 cmH_2_O (95% confidence interval (CI) 0.31–0.33), and it was higher in the ALS than in the non-ALS group (9.18 ± 2.20 versus 8.85 ± 2.63, respectively). For peak pressure, the mean difference was −0.18 cmH_2_O (95% CI −0.20 to −0.15), (20.70 ± 5.30 vs. 20.87 ± 5.65) and the mean pressure difference of −0.05 cmH_2_O (95% CI −0.04 to −0.07) (12.80 ± 3.13 vs. 12.85 ± 3.55, respectively) was also higher in the non-ALS group. The difference in the single ventilation volume per ideal body weight was 0.65 ml/kg (95% CI 0.63–0.66) (7.83 ± 3.16 vs. 7.18 ± 2.96, respectively), and the difference in dynamic lung compliance was 8.57 mL/cmH_2_O (95% CI 8.43–8.70) (50.32 ± 31.68 vs. 49.68 ± 15.16, respectively), and both were higher in the ALS group. The percentage of times that the ventilation volume per body weight exceeded 8 was higher in the ALS group (53.7% vs. 38.6%, *p* < 0.001).

**Conclusions:** There was no association between higher ventilator pressures and the development of ALS. The ALS group had higher dynamic lung compliance and higher tidal volumes, which may indicate a pulmonary contribution to ALS, and ventilatory management that limits tidal volume may prevent the development of ALS.

## P027

### VentConnect: telemetry system that enables remote live transmission of ventilator screen: qualitative study of clinicians’ expectations and experience

#### V Zvonicek^1^, M Macik^2^, J Jirman^2^, M Nemy^2^, L Vyslouzilova^2^, F Duska^3^

##### ^1^University Hospital Kralovské Vinohrady, ICU, Prague, Czech Republic, ^2^Czech Technical University in Prague, Prague, Czech Republic, ^3^University Hospital Kralovské Vinohrady, Prague, Czech Republic

*Critical Care* 2023, **27(S1)**: P027

**Introduction:** To maximise the input of intensivists onto the management of ventilated patients during the COVID pandemic, we have developed and implemented telemetry system VentConnect [1]. The aim of this study is to identify stakeholder’s expectations and experience from this technology.

**Methods:** The telemetry device VentConnect (scheme at Fig. 1) enabled transmission of HDMI signal from mechanical ventilators to a password protected interface on any web browser. We implemented it between December 2020 and March 2021 on a total of 31 beds where patients were treated during COVID Pandemic. Afterwards, we performed “Structured User Interviews with ICU doctors”. Questionnaire responses we clustered and calculated.

**Results:** Eight doctors were interviewed, 4 fully qualified intensivists, and 4 in training. By far the most demanded was the ability to see flow curve or flow pattern (100%), followed by inspiratory pressures (75%) and check tidal volume (63%). Other parameters were mentioned less frequently such as driving pressure (25%) and interferences (38%). With regards users experience, answers were overwhelmingly positive, highlighting mostly the ability to continuously monitor the progress of patients without the need to donning personal protective equipment. In some, however, curiosity was the only motivator for use. Three juniors expressed apprehension that their supervisors might criticise their ventilator setting which would otherwise had gone unnoticed. Two participants thought that the temptation to check patient 24/7 would impair their ability to rest and relax during their off time.

**Conclusions:** Telemetry system that enabled clinicians to remotely check ventilator screen met the expectation of clinicians, who mainly demanded to check flow patterns, tidal volumes and pressures. Concerns were mainly about psychological impact of using this technology. These need to be addressed.

**Acknowledgement:** Supported by Grant Ministry of Health, Czech Republic, AZV NU22-06-00625.


**Reference**

https://www.ventconnect.cz/en.html

**Figure Figj:**
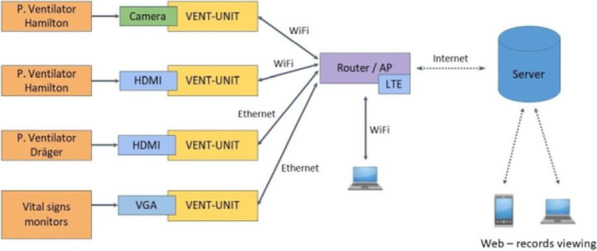
**Fig. 1 (abstract P027)**. Scheme of VentConnect System

## P028

### Understanding the influences on clinical decisions regarding ventilation of critically ill COVID-19 patients

#### K Matsumoto, H Malcolm, W Thomson, M Osman, Z Puthucheary, J Prowle, T Stephens

##### Critical Care and Perioperative Medicine Research Group, William Harvey Research Group, Queen Mary University of London, London, UK

*Critical Care* 2023, **27(S1)**: P028

**Introduction:** National Service Evaluations of COVID-19 ARDS care in the US and UK showed significant variability in clinical practice, and adherence to existing guidelines. To better understand the basis for this, we explored factors influencing decision-making around mechanical ventilation in COVID-19.

**Methods:** We conducted interprofessional focus groups identifying factors that influenced decision-making through thematic analysis. From this, we developed a questionnaire to validate these themes with a larger sample of critical care professionals across the UK. Kruskal–Wallis or Mann–Whitney U tests were used for data analysis.

**Results:** There were 179 complete responses from doctors, nurses and physios. In their usual practice, 66% of clinicians reported adherence to national ARDS guidelines. However, 80% thought COVID-19 ARDS presented differently to their previous clinical knowledge/experience of ARDS and 72% thought deviating from usual practice was necessary. Doctors were more likely to think deviation was necessary (*p* < 0.001) but there was no difference across level of ICU experience (*p* = 0.845). Clinicians reported their ventilatory decision-making was most influenced by disease factors, followed by team then contextual and least by environmental factors (*p* < 0.001). Disease factor was seen as most important across profession and experience level. During COVID-19, 68% of clinicians reported not being confident in their ventilatory decision-making; however, clinicians who felt COVID-19 ARDS presentation fitted with their previous clinical knowledge/experience of ARDS reported greater confidence (*p* < 0.001). Confidence was not affected by experience (*p* = 0.522) or profession (*p* = 0.294) (Fig. 1).

**Conclusions:** Clinicians were influenced by the uncertain understanding of COVID-19 ARDS, especially when they considered previous experiences to be less relevant. In the event of another novel disease, developing a consistent, understandable clinical models of disease should be prioritised to optimise decision making.

**Figure Figk:**
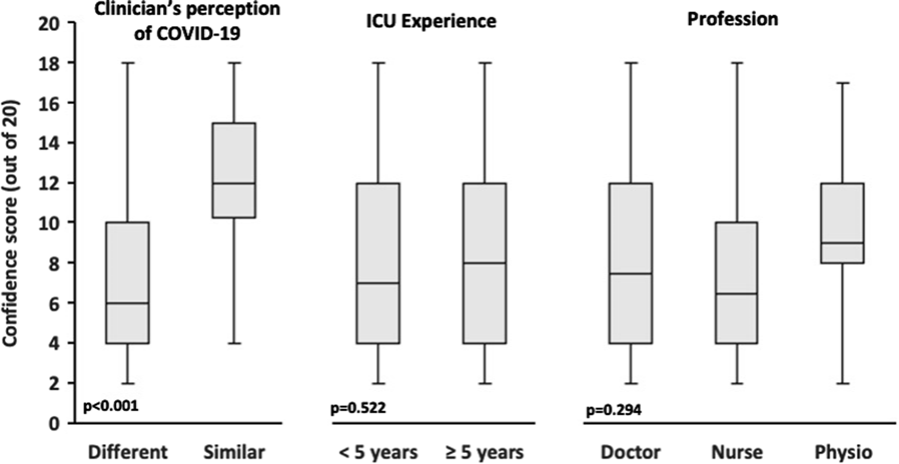
**Fig. 1 (abstract P028)**. Whisker and Boxplot chart of findings. Charts compare confidence scores (out of 20) based on clinician’s perception of COVID-19 (different or similar to their previous clinical knowledge/experience of ARDS), ICU experience and profession. *p* values are based on Mann–Whitney U test or Kruskal–Wallis test

## P029

### Prolonged mechanical ventilation: a retrospective regional study to identify need for specialist weaning service

#### G Thomas^1^, J Qiu^1^, A Campbell^2^, V Sundaram^1^, E Farley-Hills^3^, R Pugh^4^

##### ^1^Department of Anaesthetics, Bodelwyddan, UK, ^2^Wrexham Maelor Hospital, Critical Care, Wrexham, UK, ^3^Ysbyty Gwynedd, Department of Anaesthetics, Bangor, UK, ^4^Department of Anaesthetics, Glan Clwyd Hospital, Bodelwyddan, UK

*Critical Care* 2023, **27(S1)**: P029

**Introduction:** Prolonged mechanical ventilation (PMV) has been defined as the need for ≥ 21 consecutive days of mechanical ventilation for ≥ 6 h per day [1], representing approximately 5% ventilated ICU patients but consuming 30% ICU bed days [2, 3]. Specialist weaning units offer dedicated multi-professional care to patients within a cohorted environment while freeing acute critical care capacity. Elsewhere in the UK, the impact of weaning unit on critical care capacity has been modelled using a transfer threshold of 21 days [2], whereas for Wales the benefit of centralising patients ventilated > 100 days has been evaluated [4].

**Methods:** The aim of this study was to evaluate over a 5-years period: numbers of patients requiring prolonged ventilation regionally, their underlying characteristics, critical care utilisation, outcome and discharge disposition. We conducted a retrospective analysis, utilising the critical care database of three North Wales ICUs from April 1st 2017 to 31st March 2022.

**Results:** During the study period, 3640 critically ill patients received invasive respiratory support over 22,245 ventilator days. Those requiring ventilation for ≥ 30 days represented a small proportion (3%) of all ventilated patients but consumed nearly a quarter of all ventilator days (Table 1). The majority were admitted for primary respiratory aetiology (55%) with a smaller proportion (15%) for intra-abdominal pathology. Two patients were transferred to an external weaning unit for specialist management after median 203 days.

**Conclusions:** Patients requiring a mechanical ventilation for ≥ 30 days account for a significant proportion of ventilator days. The majority of such patients have a respiratory primary diagnosis and survive to hospital discharge; their needs may be sufficiently homogenous and the impact on wider critical care capacity sufficiently great that cohorting within an appropriately resourced specialist weaning unit may be appropriate. This requires further exploration.


**References**
MacIntyr NR et al. Chest 2005;128:3937–3954.Lone NI and Walsh TS. Crit Care 2011;15: R102.Hill AD et al. Ann Am Thorac Soc 2017;14: 355–362.Welsh Government. Task and Finish Group Report on Critical Care, 2019.

**Table Tabf:** **Table 1 (abstract P029)** Patient characteristics and outcomes

	≥ 30 days	≥ 60 days	≥ 100 days
No. (% vs. all patients)	113 (3%)	14 (< 1%)	3 (< 1%)
Ventilator days (% vs. all vent days)	5144 (23%)	1329 (6%)	564
Median age (years)	63	62	69
Primary diagnosis	Respiratory (55%)	Respiratory (57%)	Spinal (67%)
Survival to hospital discharge (%)	83 (73%)	12 (86%)	3 (100%)
Transfer to specialist weaning unit	2	2	1

## P030

### Accuracy, consumption and NO_2_ production of inhaled NO administration devices: a comparative bench study

#### A Vuillermoz^1^, M Lefranc^2^, N Prouvez^2^, A Mercat^1^, JC Richard^2^, F Beloncle^1^

##### ^1^CHU Angers, Service de Réanimation Médicale, Médecine Hyperbare, Angers, France, ^2^Air Liquide Medical Systems, Med2Lab, Antony, France

*Critical Care* 2023, **27(S1)**: P030

**Introduction:** Several inhaled nitric oxide (iNO) devices are available: basic continuous administration (MiniKINOX), “guided” (OptiKINOX) and “synchronized-to-ventilator” (NO-A, SoKINOX). We compared accuracy, consumption and NO2 formation in simulated ventilation conditions.

**Methods:** Four iNO devices were evaluated on a Michigan test lung (acute-respiratory-distress-syndrome model), connected to two ventilators (V800, Dräger and R860, GE Healthcare), seated at three different bias flows. Four NO concentration-settings were tested: 5, 10, 14 and 20 ppm. Actual NO and NO2 concentrations were measured in the test lung by NO and NO_2_ electrochemical cells (SoKINOX). NO–N_2_ mixture flow was measured by TSI-flowmeter to assess gas consumption.

**Results:** Actual NO concentration measured with “synchronized-to-ventilator” devices was more accurate than with “guided” and “basic continuous administration” devices in which NO concentration was mainly lower than the target and depended on the ventilator bias flow (Fig. 1). Despite differences in iNO administration, NO_2_ never exceeded the maximal safety threshold (1 ppm). “Synchronized-to-ventilator” devices which are synchronized to inspiratory triggers consumed less gas than “basic continuous administration” due to an adaptation of iNO administration. The consumption based on iNO concentration targeted was not lower with “synchronized-to-ventilator” than “guided” devices.

**Conclusions:** Last generation iNO devices, synchronized with ventilator insufflation and measuring actual flow in the inspiratory line, regulate iNO-delivery more precisely compared to older generation devices. Ventilator settings often blind for the clinician (eg. bias flow) may impact iNO accuracy.

**Figure Figl:**
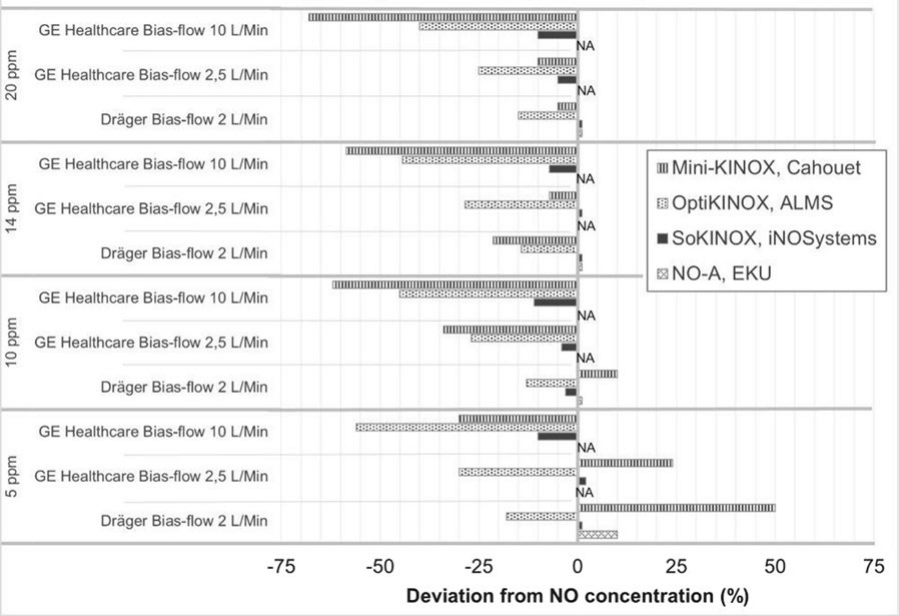
**Fig. 1 (abstract P030)**. Deviation from NO measured value to NO setting, expressed as percentage, from four devices, in three mechanical conditions (NA: Not applicable)

## P031

### Bridging the gap: association of ventilator adjustments between the operating room and intensive care unit and 28-day mortality in surgical patients

#### D Von Wedel^1^, S Redaelli^1^, A Suleiman^1^, LJ Wachtendorf^1^, D Shay^1^, BA Azizi^1^, G Chen^1^, D Talmor^1^, EN Baedorf-Kassis^2^, MS Schaefer^1^

##### ^1^Beth Israel Deaconess Medical Center, Harvard Medical School, Department of Anesthesia, Critical Care and Pain Medicine, Boston, USA, ^2^Beth Israel Deaconess Medical Center, Harvard Medical School, Department of Pulmonary, Critical Care & Sleep Medicine, Boston, USA

*Critical Care* 2023, **27(S1)**: P031

**Introduction:** Intensity of mechanical ventilation can be quantified through concepts such as mechanical power (MP), integrating driving pressure (DP), tidal volume (Vt), and respiratory rate (RR). Low intensity of ventilation during surgery and in the ICU has been associated with improved patient outcomes [1, 2]. However, there is a lack of information on the effect of ventilation adjustments when transitioning surgical patients to the ICU. We hypothesized that changes in MP from the operating room (OR) to the ICU are associated with 28-day mortality.

**Methods:** 1970 adult patients undergoing general anesthesia between 2008 and 2022, receiving continued, controlled mechanical ventilation in the ICU, were included in this retrospective study. Median MP was calculated for the last intraoperative hour and first six hours of ICU ventilation [3]. The association of MP changes from OR to ICU and 28-day mortality was assessed using multivariable logistic regression adjusted for patient demographics, comorbidities, and intraoperative factors. Relative importance of changes in individual parameters was determined by dominance analysis.

**Results:** 199 patients (10.1%) died within 28 days of surgery. Median (IQR) MP in the OR was 9.1 (6.7–11.7) and 9.7 (7.9–12.0) J/min in the ICU. Upon transition to ICU ventilation, Vt and DP were decreased, while RR was increased, and overall MP remained constant (Fig. 1A). An increase in MP from OR to ICU was associated with higher mortality (aOR 1.10 per 1 J/min increase, [1.05–1.14], *p* < 0.001, Fig. 1B). Dominance analysis revealed that this was primarily driven by increases in RR (Fig. 1C).

**Conclusions:** Adjustment of ventilator parameters leading to increased MP should be avoided when transitioning from OR to ICU. Clinicians should consider to avoid lowering Vt and DP at the cost of using high RR.


**References**
Santer P et al. Anesthesiology 2022;137:41–54.Serpa Neto A et al. Intensive Care Med 2018;44:1914–1922.Chiumello D et al. Crit Care 2020;24:417.

**Figure Figm:**
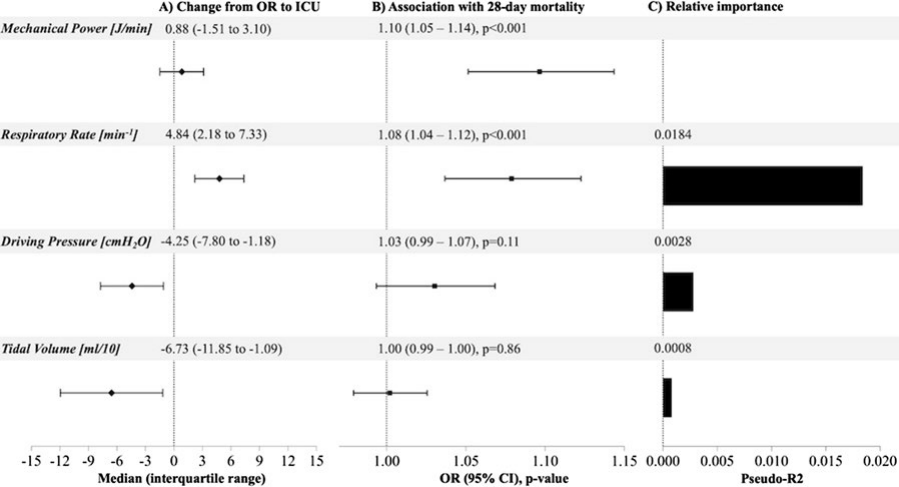
**Fig. 1 (abstract P031)**. **A** Changes in MP and its components from the OR to ICU. **B** Association of parameter changes with 28-day mortality. Estimates for MP were obtained from the primary model. A secondary model was used for estimates of MP components, including changes of RR, DP, and Vt, as independent variables in a single model. **C** Dominance analysis was performed in the secondary model. Dominance statistics (pseudo-R2) are reported as determined by contribution to 28-day mortality prediction

## P032

### Clinical value of prophylactic minitracheostomy in high-risk patients with acute cervical spinal cord injuries

#### S Maemura, M Suga, S Ishihara

##### Hyogo Emergency Medical Hospital, Department of Emergency and Critical Care Medicine, Kobe, Japan

*Critical Care* 2023, **27(S1)**: P032

**Introduction:** Cervical spinal cord injury (CSCI) is at high risk for sputum retention and respiratory exacerbation (RE). Prophylactic minitracheostomy (PMT), a technique to assist in removing airway secretions while maintaining the glottic function, allows repeated airway suctioning and can reduce incidents of intubation. However, The clinical value of PMT in acute CSCI patients is unclear. In our tertiary emergency center, PMT is performed on patients at high risk of sputum retention to prevent unexpected RE and intubation. This study aimed to evaluate the clinical value of PMT in acute CSCI patients.

**Methods:** We performed a retrospective review of patients with acute traumatic CSCI admitted to our hospital from April 2011 to April 2021. A total of 44 patients undergoing PMT were enrolled. The medical record was reviewed for age, ISS (Injury Severity Score), probability of survival (Ps), level of spinal cord injury, American Spinal Injury Association (ASIA) Impairment Scale (AIS), intubation because of RE, the success of decannulation, in-hospital mortality, complications related to the procedure.

**Results:** The median age was 67 years [interquartile range (IQR): 49–76]. The median ISS and Ps were 25 [IQR: 20–30], 0.86 [IQR: 0.69–0.94]. 33 patients out of 44 (75%) were classified as AIS grade A. 8 out of 44 (18.2%), all classified as AIS grade A, required emergency intubation at RE. That means PMT was ineffective in about one-fourth of severe CSCI patients (AIS grade A). One patient (2.3%) died in-hospital regardless of respiratory problems. One patient was intubated on the next day of PMT because of a tracheal hemorrhage. Other minor complications, including mild subcutaneous emphysema and bleeding, were found in a few patients, but all recovered quickly.

**Conclusions:** PMT for a CSCI patient with a high risk for sputum retention can prevent intubation, but we should keep in mind its failure, especially in severe CSCI patients. Identifying patients at risk of sputum retention who will benefit from PMT is important.

## P033

### Does tracheostomy predict outcome in severe COVID-19?

#### R Pinto Medeiros^1^, M Bourbon Ruão^2^, B Vale ^2^, S Salvador^3^, A Novo^2^, R Pereira^2^, Á Moreira da Silva^2^

##### ^1^Centro Hospitalar do Porto, Departamento de Anestesia e Cuidados Intensivos, Porto, Portugal, ^2^Centro Hospitalar do Porto, SCI, Porto, Portugal, ^3^Centro Hospitalar do Porto, Anesthesiology, Porto, Portugal

*Critical Care* 2023, **27(S1)**: P033

**Introduction:** The association of tracheostomy timing and clinical outcomes in ventilated COVID-19 patients remains controversial. Data from the pre-pandemic era has demonstrated the use of tracheostomy for ventilator weaning [1]. However, the use of tracheostomy in COVID-19 patients was a subject of discussion [2]. Nevertheless, evidence of the impact of tracheostomy on the outcome in critically ill COVID patients is still lacking. This study aims to evaluate the impact on Intensive Care Unit (ICU) outcome (survival) of tracheostomy in COVID-19 ventilated patients.

**Methods:** Monocentric descriptive observational study. Demographic and clinical data, timing of tracheostomy and outcome (ICU mortality) from 1st January to 31st December 2021 were registered. Analysis of descriptive statistics for continuous variables and survival analysis (log rank test).

**Results:** 115 patients were included (72% males), all mechanically ventilated, 7 (6%) were subjected to tracheostomy. The mean age was 67.2 years (range 36–84 years). The ICU mortality was 62% (71). The group of patients not submitted to tracheostomy had a mean survival time of 24.4 days (SD ± 1.5) and median survival time of 22 days (SD ± 1.7). The group of patients that were subjected to tracheostomy, the mean survival time was 68.5 days (SD ± 12.2) and median survival time was 50 days (SD ± 2). This comparison is significative (Log Rank test, *p* = 0.0001).

**Conclusions:** The present study demonstrates a better survival likelihood of the tracheostomized subpopulation. Tracheostomy was only done in 6% of patients, which elucidates a need to further prospective, randomized studies to assess the impact on the outcome of tracheostomy in ventilated COVID19 patients.


**References**
Trouillet JL et al. Ann Intensive Care 2018;8:37.Ji Y et al. Crit Care 2022;26:40.


## P034

### Early versus late tracheostomy in critically-ill COVID-19 patients

#### AS Szafran^1^, K Dahms^1^, K Ansems^1^, N Skoetz^2^, I Monsef^2^, T Breuer^1^, C Benstoem^1^

##### ^1^Medical Faculty, RWTH Aachen University, Department of Intensive Care Medicine and Intermediate Care, Aachen, Germany, ^2^Department I of Internal Medicine, Faculty of Medicine and University Hospital Cologne, University of Cologne, Cochrane Haematology, Cologne, Germany.

*Critical Care* 2023, **27(S1)**: P034

**Introduction:** In the context of the COVID-19 pandemic and a qualitatively questionable evidence base, a thorough understanding of the available evidence for the management of critically ill patients with COVID-19 is needed. Previous reports have described ICU stays and weaning as prolonged in patients who developed COVID‐19 ARDS. Prolonged MV is the most common indication for tracheostomy in ICU patients. Pre-pandemic evidence on the benefits of early tracheostomy is conflicting but suggests shorter hospital stays and lower mortality rates compared to late tracheostomy.

**Methods:** We followed the standard Cochrane methodology to conduct the systematic review and meta-analyses. RCTs and observational studies evaluating concepts of early tracheostomy compared to late tracheostomy in critically-ill COVID-19 patients were included. Methodological quality was assessed using the Cochrane RoB 2 tool and ROBINS I tool. The quality of evidence in our prioritized categories was assessed using GRADE.

**Results:** We included 1 RCT with 150 participants and 24 observational studies with 6372 critically-ill COVID-19 patients (Fig. 1). RoB assessment for the RCT was considered to be low. RoB for the observational studies was considered to be critical.

**Conclusions:**
We found low-certainty evidence that early tracheostomy may result in little to no difference in overall mortality in critically-ill COVID-19 patients requiring mechanical ventilation (MV). In terms of clinical improvement, early tracheostomy results in little to no difference in duration to liberation from MV. We are not certain about the impact on clinical worsening and ICU length of stay (LOS).Our sensitivity analysis suggests that early tracheostomy may reduce the duration of invasive MV and the ICU LOS.The overall evidence in this review is graded as low and can only be interpreted as a indication that early compared to late tracheostomy may be recommended to shorten the duration of MV in critically-ill COVID-19 patients and to reduce all-cause mortality.

**Figure Fign:**
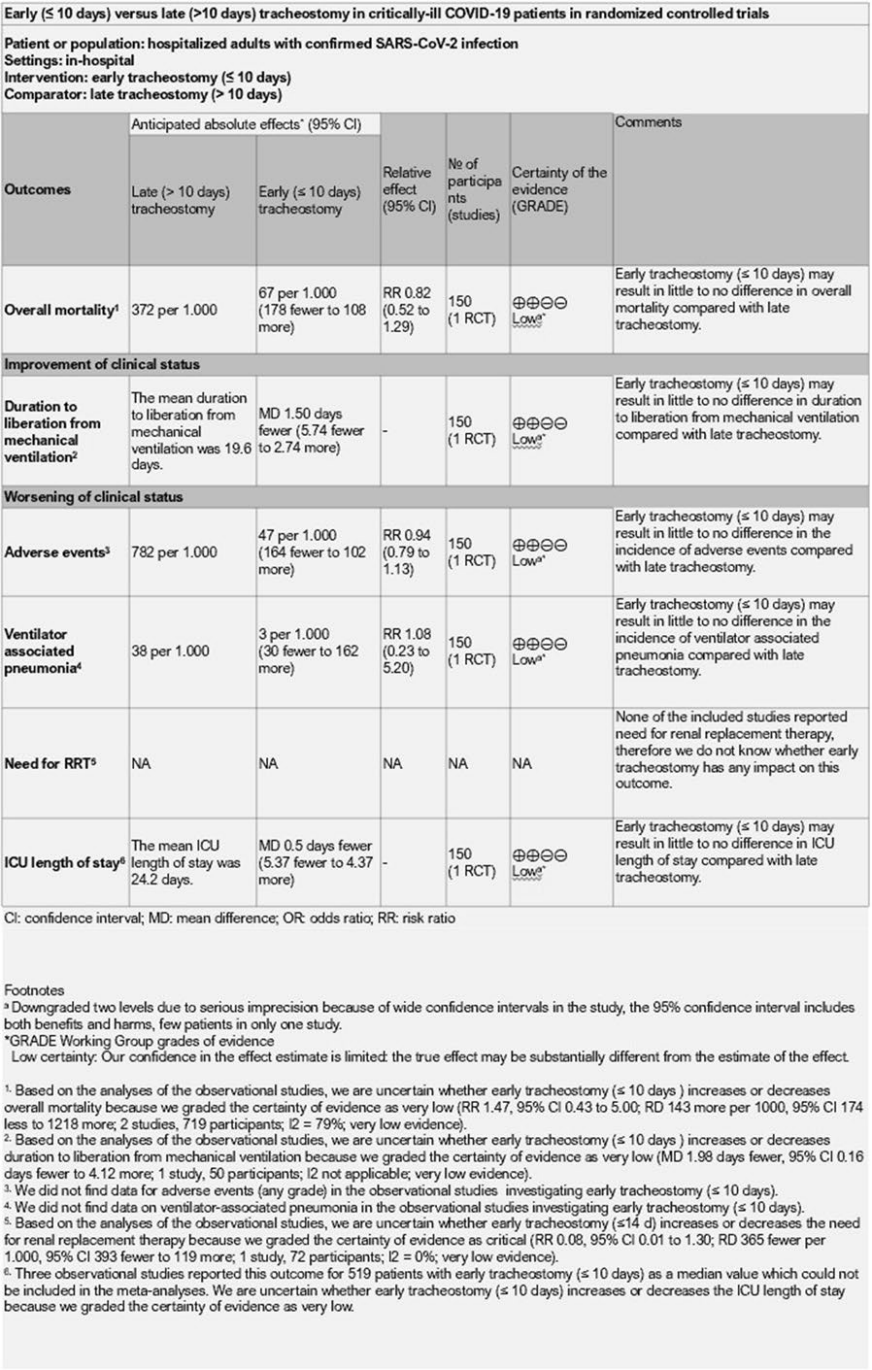
**Fig. 1 (abstract P034)**. Summary of findings

## P035

### Tracheostomy management in spinal cord injured patients and the role of diaphragm pacing

#### RPO Onders, M Elmo, N Carl

##### University Hospitals Cleveland Medical Center, Department of Surgery, Cleveland, USA

*Critical Care* 2023, **27(S1)**: P035

**Introduction:** Tracheostomies have significant risks both for the patient and health care providers including: being an aerosol generating procedure, hemorrhage, tracheomalacia, infections, granulation tissue, mucous production, pneumonias and the most severe—obstruction and dislodgement leading to death or hypoxic brain damage. In spinal cord injured (SCI) patients 75% of all patients require intubation for mechanical ventilation (MV) acutely with 20% of all cervical SCI patient requiring tracheostomy MV. We report on diaphragm pacing (DP) to decrease acute and long term need for tracheostomies in SCI patients.

**Methods:** This is a retrospective analysis of an IRB approved prospective, non-randomized interventional experience at a single institution. Subgroup analysis was limited to endotracheal intubation and tracheostomy management of all patients with traumatic cervical SCI that were implanted laparoscopically with DP (NeuRx, Synapse Biomedical) within 30 days of injury to wean from MV.

**Results:** For the study group of early DP, there were 13 (all male) patients with average age at implant 49.3 years (range 17–70). Injury mechanisms included falls (6), MVA (4), GSW (2) and diving (1). One patient withdrew care 14 days post injury and is exclude from further analysis. 2 patients had tracheostomies before DP due to specific nature of their injuries. 3 patients had weak diaphragms with nerve injuries identified at surgery requiring prolonging weaning with tracheostomies. In total, 4 of 12 patients did not require tracheostomy (33%). For patients with intact diaphragms and no need for tracheostomies because of their injury-57% did not require tracheostomy (4 of 7). 3 additional patient(7 of 12 or 58%) had decanullation within 6 months of injury. Hospital length of stay for patients without tracheostomy was 19 days (range 19–27) and those requiring tracheostomies was 31 days (21–42).

**Conclusions:** Early DP allowed up to 57% of previously non-weanable patients to be extubated without tracheostomies.

## P036

### Early versus late tracheostomy in multiple trauma patients in the intensive care unit: systematic review and meta-analysis

#### K Ansems, E Aleksandrova, AS Szafran, K Dahms, T Breuer, C Benstoem

##### Medical Faculty, RWTH Aachen University, Department of Intensive Care Medicine and Intermediate Care, Aachen, Germany

*Critical Care* 2023, **27(S1)**: P036

**Introduction:** The objective of this systematic review and meta-analysis is to assess the effects and safety of concepts of early tracheostomy (ET) compared to concepts of late tracheostomy (LT) in patients with multiple trauma on the ICU.

**Methods:** We followed standard Cochrane methodology. Randomized controlled trials (RCTs) and observational studies comparing ET with LT in adult patients with polytrauma in the ICU were included, regardless of disease severity, gender, ethnicity, or setting. Methodological quality was assessed using the Cochrane RoB 2 tool and ROBINS-I tool. The quality of evidence, in the categories we prioritized, was assessed using GRADE.

**Results:** We included one RCT (n = 60) and nine observational studies with a total of 1.694 multiple trauma patients who underwent tracheostomy. Statistical analysis of the RCT data showed that ET (≤ 8 days) was likely to have little or no effect on all-cause mortality. We found no differences in duration of mechanical ventilation, length of ICU stay, or number of patients with ventilator-associated pneumonia. Data from observational studies showed that the evidence is very uncertain about the effect of ET (≤ 7 days) on ICU mortality. ET (≤ 7 days) may reduce the duration of mechanical ventilation, ICU length of stay, VAP. ET (≤ 7 days) may have little to no effect on hospital length of stay but the evidence is very uncertain.

**Conclusions:** The evidence is very uncertain about the effect of ET performed ≤ day 7 on ICU mortality, reduction of the duration of MV, ICU length of stay, hospital length of stay and the incidence of VAP in patients with multiple trauma on the ICU. We strongly recommend performing high-quality (prospectively registered) observational studies and implementing the New Berlin definition for multiple trauma to ensure a more standardized patient population across studies. Additionally, we encourage authors to pool patients by similar injury patterns and provide information about the tracheostomy timing of the individual groups.

## P037

### Tracheostomy outcomes in COVID-19 and non-COVID-19 critical patients

#### RV Jorge^1^, A Orfão^2^, J Pacheco Pereira^3^, J Patrício^3^, P Patrício^3^

##### ^1^Hospital Beatriz Ângelo, Intensiva Medicine Department, Loures, Portugal, ^2^Hospital Professor Dr. Fernando Fonseca, Internal Medicina Department, Amadora, Portugal, ^3^Hospital Beatriz Ângelo, Intensive Medicine Department, Loures, Portugal

*Critical Care* 2023, **27(S1)**: P037

**Introduction:** Tracheostomy is a common surgical procedure in the setting of acute respiratory failure. And improves outcomes for critically patients requiring prolonged mechanical ventilation. Initially avoided due to it’s high risk to biosafety, tracheostomy soon became a routine procedure in the critical support of critical ill patients affected by COVID-19. The aim of this review was to compare tracheostomy done in COVID-19 and non-COVID-19 pneumonias in an UCI.

**Methods:** This retrospective, observational study included 78 patients (23 female, 55 male; age range: 23–90 years, mean age: 66) with severe pneumonia who were admitted to the intensive care unit (ICU) of Hospital Beatriz Ângelo (Portugal) between 01/03/2012 until 31/12/2021, to whom a tracheostomy was performed. Patients underwent orotracheal intubation with invasive mechanical ventilation, followed by percutaneous or open surgical tracheotomy. Indications, timing of the procedure, and time needed to complete weaning and decannulation, as well as complications, were reported and compared between patients with COVID-19 (N = 38) and non-COVID-19 (N = 40) pneumonias.

**Results:** In both groups, weaning from difficult ventilation was the most common indication for the procedure, followed by prolonged mechanical ventilation in the COVID-19 group (42%) and protection of the airway/secretions management in the non-COVID-19 group (22.5%). Timing of the procedure was 14.6 and 16.4 days after mechanical ventilation in the COVID-19 and non-COVID group, respectively. The non-COVID-19 group reported more days to decannulation (39.3 vs 15.1 days) as well as more days to wean off from mechanical ventilation (20.3 vs 14.1 days) and more major complications (12.5% vs 2%). Hospital discharge rate was similar in both groups (COVID-19 with 42.1% and 42.5% to non-COVID-19).

**Conclusions:** Although the differences between both groups are multifactorial, it’s useful for self-evaluation observations, as well as sharing practices and outcomes for further analysis.

## P038

### Rapidly progressive brain atrophy in mechanically ventilated patients: a retrospective descriptive study using CT volumetry

#### R Nakae^1^, T Sekine^2^, T Tagami^3^, Y Murai^4^, G Warnock^5^, A Morita^4^, S Yokobori^1^

##### ^1^Nippon Medical School Hospital, Department of Emergency and Critical Care Medicine, Tokyo, Japan, ^2^Nippon Medical School Musashi Kosugi Hospital, Department of Radiology, Kawasaki, Japan, ^3^Nippon Medical School Musashi Kosugi Hospital, Department of Emergency and Critical Care Medicine, Kawasaki, Japan, ^4^Nippon Medical School Hospital, Department of Neurological Surgery, Tokyo, Japan, ^5^PMOD Technologies Ltd., Zürich, Switzerland

*Critical Care* 2023, **27(S1)**: P038

**Introduction:** Mechanical ventilation has the potential to decrease cerebral blood flow; however, changes in brain volume associated with mechanical ventilation are not well understood. We assessed brain atrophy in mechanically ventilated patients using brain CT scans, and their findings’ relationship to outcomes.

**Methods:** We retrospectively analyzed mechanically ventilated patients admitted to an ICU who underwent at least two head CT scans during hospitalization. Patients with stroke, traumatic brain injury, or hypoxic encephalopathy were excluded. The first brain CT scan was routinely performed on admission, and the second and further brain CT scans were obtained whenever prolonged disturbance of consciousness was observed. Brain volume was estimated using an automatic segmentation method, and patients with a brain volume change < 0% from the first CT scan to the second CT scan were defined as the “brain atrophy group” and those with ≥ 0% were defined as the “no brain atrophy group.” Duration of mechanical ventilation, length of ICU stay, and mortality were compared between the groups.

**Results:** A total of 52 patients were included, 44 in the brain atrophy group and 8 in the no brain atrophy group. Analysis of the brain atrophy group showed a significant decrease in brain volume (first CT scan: 1.042 ± 0.124 L vs. second CT scan: 0.998 ± 0.123 L, t (43) = 9.705, *p* < 0.001). The mean change in brain volume in the brain atrophy group was − 43.5 cm^3^ over a median of 33 days. The median time on mechanical ventilation was 33 (IQR: 18–60) days and 25 (19–43) days, length of ICU stay was 30 (16–61) days and 30 (17–56) days, and mortality rate was 31.8% (14/44 cases) and 50.0% (4/8 cases) in the brain atrophy group and the no brain atrophy group, respectively.

**Conclusions:** Many ICU patients on mechanical ventilation who developed prolonged mental status changes showed signs of brain atrophy. Patients with rapidly progressive brain atrophy were more likely to have a longer duration of mechanical ventilation.

## P039

### A quality improvement project to analyse the impact of an extubation checklist on ICU re-intubation rates and efficiency

#### PI Jenkinson, D Lau, J Sokhi, M Gabrel

##### Chelsea and Westminster Hospital, Intensive Care Medicine, London, UK

*Critical Care* 2023, **27(S1)**: P039

**Introduction:** Reintubation within 48 h of extubation, termed “failed extubation”, is associated with increased morbidity, mortality and length of ICU stay. An initial audit of our mixed adult ICU demonstrated a failed extubation rate of 23.3%, far higher than the recommended rate of 5–10% [1]. Our aim was to determine whether an extubation checklist would reduce reintubations and improve flow through the ICU.

**Methods:** All extubations at Chelsea and Westminster ICU during two 3-month periods, pre and post-intervention, were analysed. Patients who underwent primary tracheostomy, self-extubation, transfer to a different facility or extubation as part of end-of-life care were excluded. Our intervention consisted of a checklist, to be completed prior to extubation, which was based on current literature and case analyses of failed extubations in the first 3-month period. The primary outcome was rates of failed extubation and the secondary outcome was extubation time. Compliance with the intervention was determined by measuring rates of completed checklists and doctors were surveyed to assess reasons for non-compliance.

**Results:** Pre-checklist (Oct–Jan 2022) there were 30 primary extubations, of which 7 failed. Post-intervention (Feb–May 2022) there were 28 primary extubations, of which 2 failed. This showed that rates of failed extubation reduced by 16.2% following checklist introduction (Fig. 1) and on average, patients were extubated 1 h and 45 min earlier in the day. Compliance with the intervention was 39%. Our survey results showed that 55% of non-compliance was due to administration issues.

**Conclusions:** Our findings suggest that an extubation checklist is a simple and effective tool to reduce rates of failed extubation. Although compliance was low, our survey indicated that this was largely due to administrative issues which could be easily addressed. More research is warranted to assess the effect on patient mortality, morbidity and length of ICU stay.


**Reference**
Krinsley J et al. Crit Care. 2012;16:111.

**Figure Figo:**
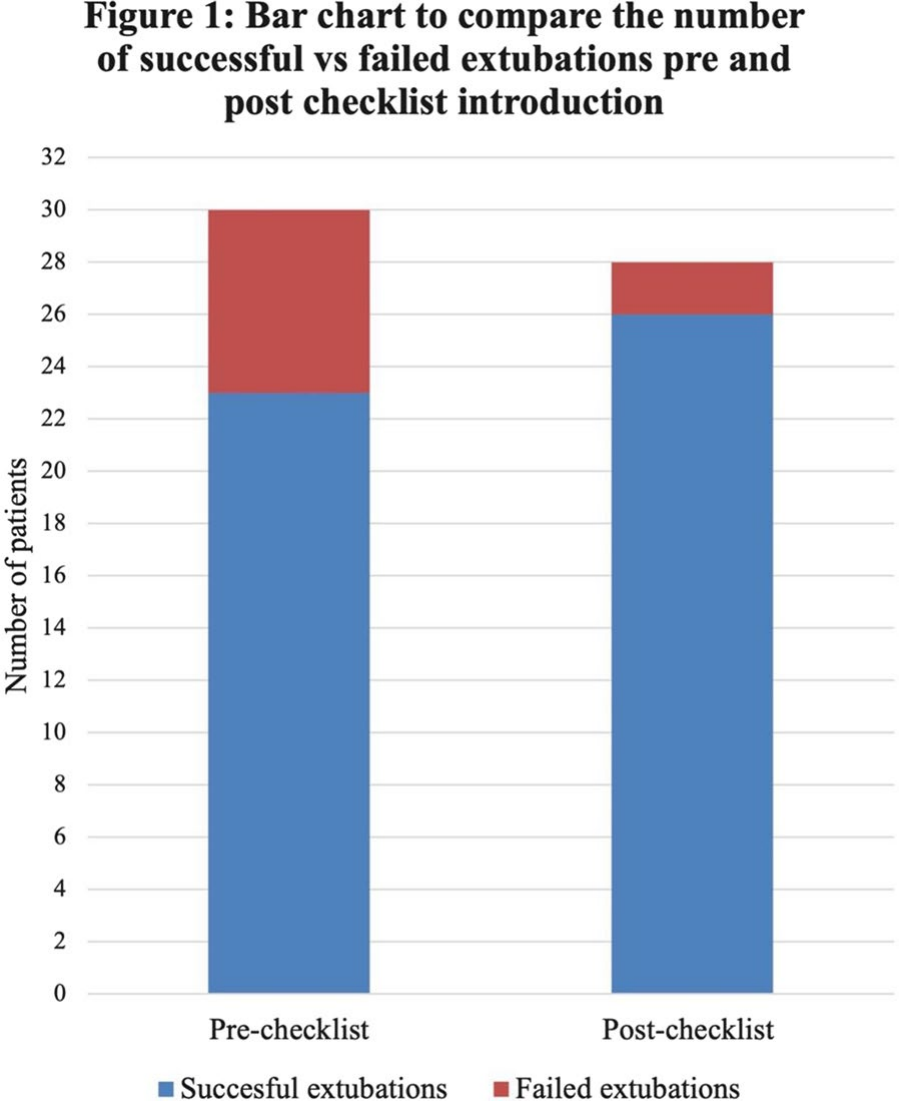
**Fig. 1 (abstract P039)**. Bar chart to compare the number of successful versus failed extubations pre and post checklist introduction

## P040

### A quality improvement project (QIP) to introduce a standardised extubation checklist at the Royal Free Hospital ICU (phase 1)

#### LY Goh, A Carnachan, C Greenfield, M Bakir, A Walecka

##### Royal Free Hospital, Intensive Care Unit, London, UK

*Critical Care* 2023, **27(S1)**: P040

**Introduction:** We aim to introduce a standardised extubation checklist to determine suitability for extubation. We hope that with clearly defined parameters we can reduce extubation failure and reintubation, which is associated with worse outcomes and increased mortality [1].

**Methods:** Retrospective data was collected from 20 extubations in December 2021, which included associated airway risk factors, prior respiratory support requirements, and number of reintubations. A survey of nursing staff highlighted the need for clear guidance and protocol. A preliminary checklist was developed based on review of the Difficult Airway Society (DAS) guidelines [2]. After comparing with a pre-existing extubation standard operating procedure (SOP), this checklist was expanded on and approved for implementation. We will evaluate the use of this checklist by auditing documentation of its use, staff feedback and further assessment of reintubation rates (Phase 2).

**Results:** Data collected showed that six of the 20 patients (30%) studied were reintubated within 48 h of their initial extubation. Reasons for reintubation included stridor, epistaxis, drowsiness, and failed non-invasive ventilation (NIV) trial. The preliminary checklist created defined a list of airway risk factors and included steps from identifying readiness for extubation until post-extubation care. The SOP incorporated standard extubation criteria for use in all patients, and an extended criteria for complex patients as well as clearly defined targets for each parameter. The final checklist contains elements from both, and patients intubated for more than 10 days qualify for the extended criteria (Fig. 1).

**Conclusions:** This project highlights the importance of contributions from the multidisciplinary team to ensure the successful uptake and implementation of an intervention.


**References**
Thille AW et al. Am J Respir Crit Care Med. 2013;187:1294–302.Difficult Airway Society. DAS extubation algorithm. 2011.

**Figure Figp:**
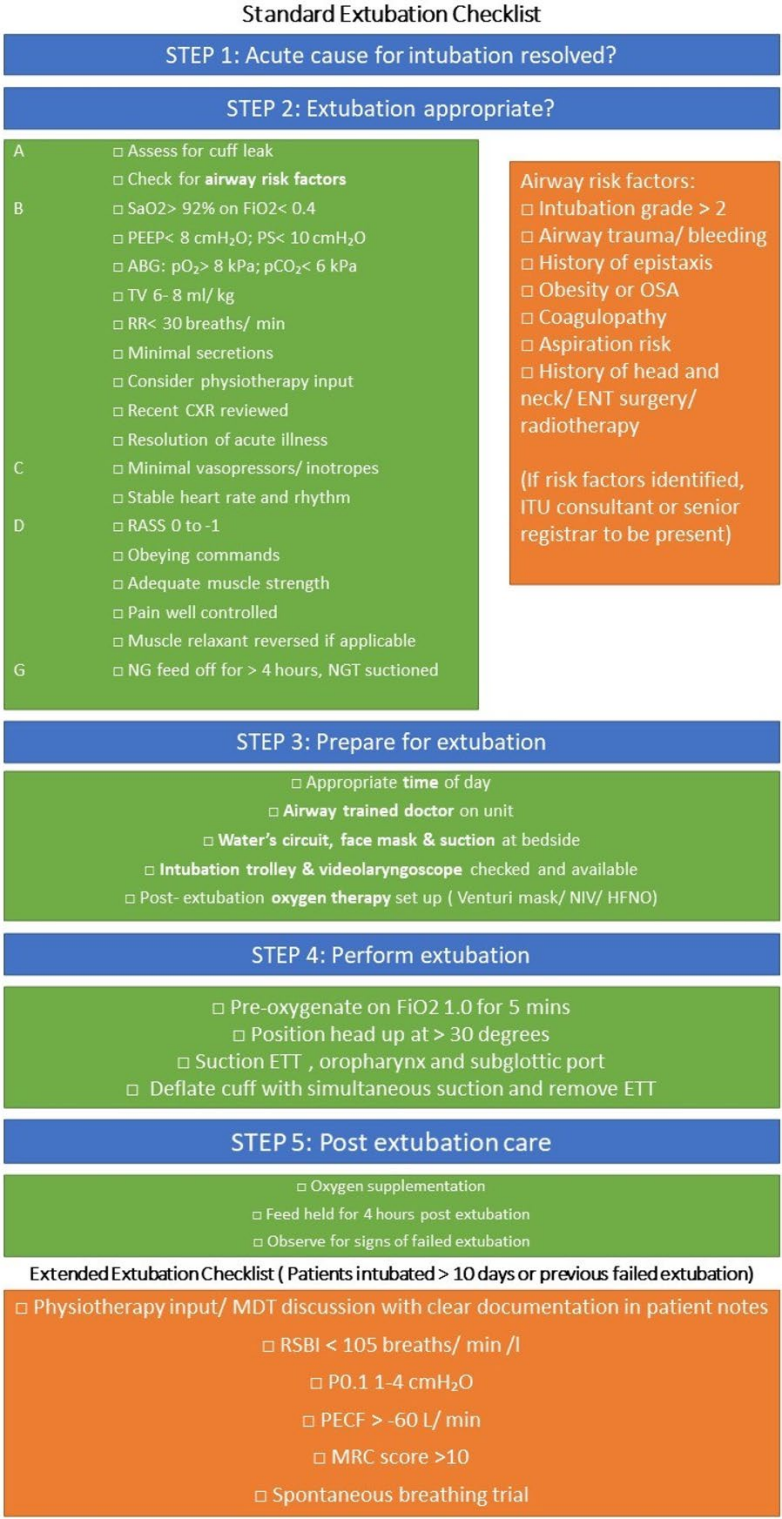
**Fig. 1 (abstract P040)** Standard and extended extubation checklists

## P041

WITHDRAWN

## P042

### Artificial neural network predicts extubation failure with uncertainty

#### YI Igarashi

##### Nippon Medical School, Department of Emergency and Critical Care Medicine, Tokyo, Japan

*Critical Care* 2023, **27(S1)**: P042

**Introduction:** Ventilator liberation is one of the most critical decisions in the intensive care unit; however, prediction of extubation failure is difficult, and the proportion thereof remains high. Machine learning can potentially provide a breakthrough in the prediction of extubation success.

**Methods:** This is a retrospective study using a two-center database. Using 27 features and data from 24 h prior to extubation, we developed models with and without uncertainty output using an artificial neural network consisting of three connected layers and one dropout layer. Five-fold cross validation was performed on each model to compare the accuracy of models with and without uncertainty output. Outcome was extubation failure, which was defined as reiintubation within 72 h after extubation.

**Results:** In MIMIC-III, 1203 patients underwent extubation, 1075 successfully and 128 unsuccessfully. In the NMS, 88 patients were extubated, 77 successfully and 11 unsuccessfully. The accuracy of the model with uncertainty was higher than that of the model without incertainty. In addition, the accuracy increased with external validation, with accuracy 0.51, precision 0.17, recall 0.73, negative predictive value 0.93, and F1 score 0.27.

**Conclusions:** An ANN model with uncertainty was developed to predict extubation, and the sensitivity of the model was increased. This model is expected to reduce the extubation faliure.

## P043

### Greater concentration of *N*-methyl-d-aspartate receptor in the brainstem in moderate-ARDS pigs ventilated for 12 h with diaphragm neurostimulation

#### T Bassi^1^, E Rohrs^2^, J Witmann^2^, K Fernandez^2^, M Nicholas^2^, M Orowska^2^, M Gani^1^, D Evans^1^, S Reynolds^2^

##### ^2^Lungpacer Medical Inc., Burnaby, Canada, ^2^Royal Columbian Hospital, New Westminster, Canada

*Critical Care* 2023, **27(S1)**: P043

**Introduction:** Studies have shown that mechanical ventilation (MV) and acute respiratory distress syndrome (ARDS) are associated with hippocampal cellular apoptosis. NR2A is an *N*-methyl-d-aspartate receptor linked to cellular survival and death. A greater concentration of synaptic NR2A is associated with cell survival and improved working memory. Temporary transvenous diaphragm neurostimulation (TTDN) in association with MV has been shown to mitigate hippocampal apoptosis in preclinical models. This study focused on investigating whether 12 h of MV with moderate ARDS would lead to a change in NR2A tissue concentration in the brainstem and whether diaphragm neurostimulation would affect that.

**Methods:** Juvenile pigs (4–5 months, 50–87 kg) underwent protective MV (volume control, PEEP 5 cmH_2_O, tidal volume 8 ml/kg) for 12 h, after being induced with moderate ARDS by injecting oleic acid into the pulmonary artery until PaO_2_/FiO_2_ was between 100 and 200. Subjects were assigned to three groups (n = 6 per group): lung injury with MV only (MV), lung injury with MV and with TTDN every other breath (MV + TTDN50%), and lung injury with MV and with TTDN every breath (MV + TTDN100%). Diaphragm neurostimulation was delivered using methods published previously. After the study, the brainstem (pons and medulla oblongata) was harvested and marked with enzyme-linked immunoassay to measure the tissue concentration of NR2A. The Kruskal–Wallis test was used for statistical analyses. *p* values < 0.05 are considered statistically significant.

**Results:** Brainstem NR2A concentrations were 4 pg/ml (3–5) for the MV group, 5 pg/ml (5–8) for the MV + TTDN50% group, and 7 pg/ml (6–8) for the MV + TTDN100% group, with a significant difference between the MV and the MV + TTDN100% groups, *p* = 0.0125 (Fig. 1).

**Conclusions:** In a preclinical model of moderate ARDS, diaphragm neurostimulation on every breath in association with MV for 12 h led to significantly greater concentrations of NR2A in the brainstem, compared to the group receiving MV, only, for 12 h.

**Figure Figq:**
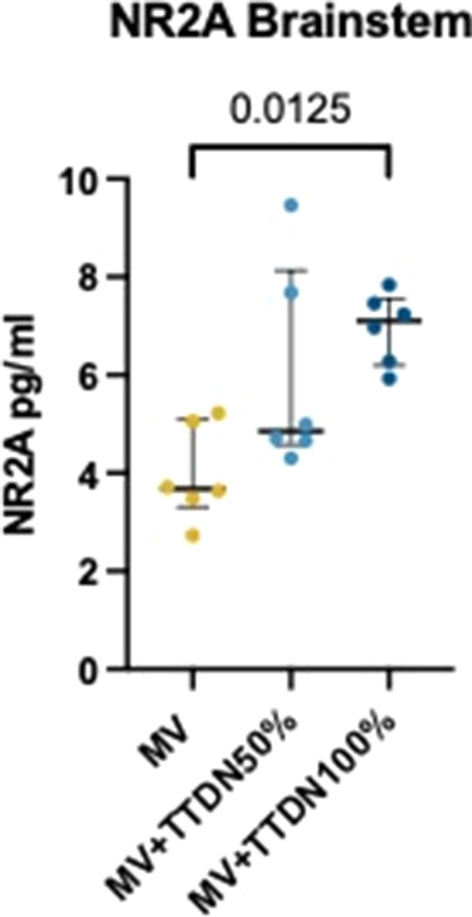
**Fig. 1 (abstract P043)** Brainstem NR2A concentrations

## P044

### Heart rate fluctuations and mortality in patients with acute respiratory failure: a retrospective analysis of the MIMIC-III database

#### L Tao^1^, J. Liu ^2^, C. Li^1^, Y Pan^1^

##### ^1^The Affiliated Suzhou Hospital of Nanjing Medical University, Suzhou Municipal Hospital, Suzhou Clinical Medical Center of Critical Care Medicine, Suzhou, China, ^2^The Affiliated Suzhou Hospital of Nanjing Medical University, Suzhou Municipal Hospital, Suzhou Clinical Medical Center of Critical Care Medicine, Department of Critical Care Medicine, Suzhou, China

*Critical Care* 2023, **27(S1)**: P044

**Introduction:** There is limited evidence for an association between heart rate fluctuations in patients with acute respiratory failure and 28-day mortality. Therefore, the objectives of this paper were to investigate if changes in heart rates are connected with 28-day mortality among patients with acute respiratory failure after controlling for other factors.

**Methods:** A total of patients 6719 with acute respiratory failure was recruited into the Medical Information Mart database for Intensive Care (MIMIC)-III in this investigation, which was conducted as a cohort study. Within 24 h of admission, variations in heart rate were estimated as the difference between maximum heart rate and minimum heart rate. Individuals were divided into three groups: those with low heart rate fluctuations [less than thirty beats per minute], those with central rate fluctuations (30–49 beats per minute), and those with high heart rate fluctuations (more than fifty beats per minute). We used models of multivariate logistics regression, Generalized additive models and linear regression models of two segments to examine the influence of 24-h heart rate variations on death on the 28 day.

**Results:** Sixty-seven hundred and ninety patients suffering from acute respiratory failure were included in the study. The fluctuations in heart rates were independent due to 28-day mortality (dominance ratio [OR] 1.63; 95% confidence interval [95% CI (1.4–1.89); *p* < 0.001) adjusted for possible confusion. There was a non-linear association between heart rate fluctuations and 28-day mortality with a threshold of approximately 21 beats per minute. The effects sizes and ci were 0.98 (0.96, 1.00) and 1.01 (1.00, 1.01) respectively.

**Conclusions:** In patients suffering from acute respiratory failure, there is a non-linear association between heart rate fluctuations over 24 h and 28-day mortality, with a particularly marked increase in death on the 28th day when heart rate fluctuations exceed 21 beats per minute.

## P045

WITHDRAWN

## P046

### Mechanical power in patients receiving mechanical ventilation in the surgical intensive care unit and the association with increased mortality

#### A Piriyapatsom, A Trisukhonth, O Chintabanyat

##### Department of Anesthesiology, Faculty of Medicine Siriraj Hospital, Mahidol University, Anesthesiology, Bangkok, Thailand

*Critical Care* 2023, **27(S1)**: P046

**Introduction:** Mechanical power (MP) represents the amount of energy delivered by mechanical ventilation (MV) to overcome elastic static, elastic dynamic and resistive components of the respiratory system during each inspiration. Evidences show the correlation between the MP and clinical outcomes in mechanically ventilated patients. The aim of this study was to determine the MP and the associated outcomes in patients admitted to surgical ICU (SICU) and receiving MV for more than 12 h.

**Methods:** This was a retrospective analysis of the prospective observation cohort study. Patients whose age ≥ 18 years old admitted to the SICU and receiving MV for at least 12 h were included. Demographic and clinical data were recorded. Ventilator parameters at the initiation and at 24 h of MV support were collected and were used to calculate the MP. Cox regression was analyzed to determine the association between the MP and 90-day mortality.

**Results:** There were 306 patients included in this study. Overall, 90-day mortality was 19.3%. The MP at the initiation and at 24 h of MV was significantly higher in non-survivors (median 11.00 (IQR 7.89–14.18) J/min vs. 7.97 (6.51–10.42) J/min and 8.86 (7.01–12.55) J/min vs. 7.24 (5.61–9.43) J/min, both *P* < 0.001, respectively). The Cox regression analyses demonstrated that MP ≥ 10 J/min at the initiation of MV and MP ≥ 10 J/min both at the initiation and at 24 h of MV were associated with increased 90-day mortality (HR 1.855, 95% CI 1.074–3.203 and HR 1.906, 95% CI 1.080–3.361, respectively). In subgroup of patients with MP ≥ 10 J/min at the initiation of MV support, further increasing in MP at 24 h of MV was associated with increased 90-day mortality (HR per J/min 1.093, 95% CI 1.007–1.187).

**Conclusions:** In mechanically ventilated patients in the SICU, the MP was an independent factor associated with 90-day mortality. Change in the MP during MV support potentially affected 90-day mortality in these patients.

## P047

### The impact of prone episodes on oxygenation in COVID-19 mechanically ventilated patients

#### F Neyroud, A Jackson, J Barnsely, E Hunter, M Radharetnas, R Beecham, A Dushianthan

##### University Hospital Southampton, Intensive Care, Southampton, UK

*Critical Care* 2023, **27(S1)**: P047

**Introduction:** Prone positions have been used extensively to improve oxygenation in patients with acute respiratory distress syndrome (ARDS). During the COVID-19 pandemic there was widespread adoption of proning in patients with acute severe hypoxic respiratory failure. Few studies explore the use of prone positioning in mechanically ventilated COVID-19 patients.

**Methods:** This study was part of the REACT COVID observational study at University Hospital Southampton (UHS) [1]. Eligibility included admission to UHS with a positive COVID-19 RT-PCR between 03/2020 and 03/2022. Data was collected from all available electronic clinical data sources using semi-automated and manual data extraction.

**Results:** 184 patients received invasive mechanical ventilation with documented evidence for 931 prone episodes. We performed detailed analysis for 763 prone episodes. The rest were excluded due to insufficient data. The median duration of each cycle was 16 h (IQR 15–17 h). 459 cycles were done within 7 days of intubation (early), 202 in 7–14 days (intermediate) and 102 after 14 days (late). The change in oxygenation defined as delta PaO_2_/FiO_2_ ratio (ΔPF) for early, intermediate, and late cycles were 2.4 ± 5.2 kPa, 1.6 ± 3.7 kPa and 1.4 ± 4.0 kPa, (*p* = 0.03) respectively. The overall ΔPF for all groups after a cycle was 2.1 ± 4.7 kPa. There was an increase in PaCO_2_ following proning with an overall change of 0.30 ± 1.0, however, this was not statistically significant (*p* = 0.30).

**Conclusions:** Following proning, there was significant improvement in oxygenation. Cycles lasted for 16 h consistent with current ARDS guidelines [2]. Although the results suggest a diminishing response in those proned at later times, the ΔPF ratio was still significant. Overall, this suggests a beneficial effect on oxygenation. However, findings cannot be translated into survival benefit. Further research including randomised controlled trials is recommended.


**References**
Burke H et al. BMJ Open. 2021;11:e043012.Guérin C et al. N Engl J Med. 2013;368:2159–68.


## P048

### Association between pulmonary extravascular water, dead space, and clinical outcome in intubated patients with COVID ARDS

#### S Leighton^1^, M Pontony^2^, R Pérez^2^, J Graf^2^, R López^2^

##### ^1^Clínica Alemana de Santiago, Intensive Care Unit, Santiago, Chile, ^2^Clínica Alemana de Santiago, Departamento Paciente Crítico, Santiago, Chile

*Critical Care* 2023, **27(S1)**: P048

**Introduction:** The aim of this study was to determine whether there is an association between extravascular lung water index (EVLWi) and physiological respiratory dead space (VDphys/VT) and to determine if these factors are associated with the possibility to being discharged alive on day 28.

**Methods:** We analyzed a prospective cohort of patients with COVID ARDS supported with invasive mechanical ventilation (IMV) admitted in our ICU who were monitored with volumetric capnography and transpulmonary thermodilution (TPTD). First day TDTP and VDphys/VT were considered. Bohr-Enghoff formula was used to obtain VDphys/VT. This protocol was approved by the local IRB and informed consent was waived.

**Results:** 31 patients with matched TPTD and VDphys/VT during the first 24 h were analyzed in who EVLWi correlated with VDphys/VT (r = 0.599 *p* = 0.002), however, EVLWi did not associated with PaFi. Patients with EVLWi > 10 ml/kg had higher APACHE II and VDphys/VT. These patients had a lower cumulative incidence to be discharged alive on day 28 with aHR 7.3 [1.4–39.1] *p* = 0.02 (adjusted by APACHE II and VDphys/VT, Fig. 1A). Remarkably, patients with EVLWi > 10 ml/kg + VDphys/VT > 57% had worse outcome compared to those who had EVLWi > 10 ml/kg + VDphys/VT < 57% (25% vs 75%, *p* = 0.032, Fig. 1B).

**Conclusions:** In patients with COVID ARDS supported with IMV, VDphys/VT give prognostic data additional to EVLWi.

**Figure Figs:**
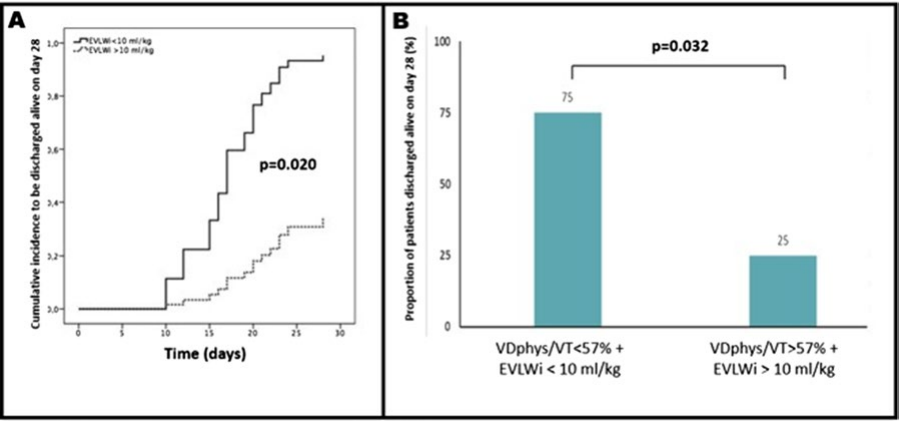
**Fig. 1 (abstract P048)**.** A**: Outcome comparison between EVLWi < 10 ml/kg and > 10 ml/kg adjusted by APACHE II and VDphys/VT.** B**: outcome comparison in patients with EVLWi > 10 ml/kg + VDphys/VT < 57% versus patients with EVLWi > 10 ml/kg + VDphys/VT > 57%

## P049

### Effect of positive end-expiratory pressure (PEEP) on the distance of pleura to subclavian vein and on the cross-sectional area of the subclavian vein in mechanically ventilated patients

#### Y Vercammen, E Mangelschots, P Vets

##### ZNA Middelheim, Intensive Care, Antwerp, Belgium

*Critical Care* 2023, **27(S1)**: P049

**Introduction:** Pneumothorax is a well-known complication of subclavian vein (SCV) catheterization because of the adjacent localization of the pleura [1]. Although there are no strict recommendations on the use of PEEP during catheterization, there is a common clinical practice of reducing PEEP to 0 cm H2O in order to prevent a pleural puncture. We investigated the effect of applying PEEP on the distances from SCV to pleura (D_SCV-Pleura_) as well as the effect of PEEP on the cross-sectional area of the SCV (CSA_SCV_). We hypothesize that administration of PEEP will not affect the D_SCV-Pleura_ thereby not increasing the risk of pneumothorax. In addition, applying PEEP may facilitate SCV catheterization by increasing the CSA_SCV_.

**Methods:** We included 20 adult patients admitted at the ICU of ZNA Antwerp in 2022 following cardiac surgery who were mechanically ventilated. Using ultrasound we measured the cross-sectional diameter of the SCV (CSD_SCV_) and D_SCV-Pleura_ after administration of PEEP 0 till 15 cm H_2_O. Differences of CSD_SCV_ and D_SCV-Pleura_ after applying PEEP were assessed with repeated measures ANOVA and paired sample t-test.

**Results:** There was no significant difference of D_SCV-Pleura_ between PEEP 0 and PEEP 15 (*p* = 0.336). Measurements of CSD_SCV_ showed no significant difference of CSA_SCV_ between PEEP 0 and PEEP 15 (*p* = 0.085) (Table 1).

**Conclusions:** The administration of PEEP did not affect the D_SCV-Pleura_ neither the CSA_SCV_, thereby questioning the common clinical practice of reducing PEEP to 0 cm H_2_O during SCV catheterization. Our findings suggest we could maintain a level of PEEP during SCV catheterization in mechanically ventilated patients with the aim of preventing alveolar derecruitment and desaturation [2]. Moreover, there was no proof of facilitation of catheterization by applying PEEP. Further research could help us to confirm these findings and improve medical practice.

**Table Tabh:** **Table 1 (abstract P049)** CSDSCV and DSCV-pleura after applying PEEP

	D_SCV-Pleura_ (mm)	CSD_SCV_ (mm)
PEEP 0	7.70 ± 3.03 (6.73–8.70)	10.26 ± 2.14 (9.58–10.95)
PEEP 15	7.38 ± 2.38 (6.62–8.14)*	9.90 ± 2.43 (9.12–10.68)**

Data of D_SCV-pleura_ and CSD_SCV_ are shown as mean ± SD (95% confidence interval). CSD = cross-sectional diameter; SCV = subclavian vein; D_SCV-pleura_ = distance between the subclavian vein and pleura. PEEP 0 = PEEP 0 cmH_2_O, PEEP 15 = PEEP 15 cmH_2_O. CSA (cross-sectional area) was calculated with the formula: CSA = r^2^ x Π (radius r = CSD/2). The paired sample t-test was used to compare means of D_SCV-pleura_ and CSD_SCV_ between PEEP 0 and PEEP 15. A *p* value of < 0.05 was considered to be statistically significant. **p* = 0.336 > 0.05, ***p* = 0.084 > 0.05


**References**
McGee DC et al. N Engl J Med 2003;348:1123–3.Martinsson A et al. Br J Anaesth 2021;126:1067–74.


## P050

### Association between lactate level in bronchoalveolar lavage fluid and outcome and regional pulmonary inflammation in acute respiratory distress syndrome

#### XL Liu, WC Chang

##### Jiangsu Provincial Key Laboratory of Critical Care Medicine, Department of Critical Care Medicine, Zhongda Hospital, School of Medicine, Southeast University, Nanjing, China, Nanjing, China

*Critical Care* 2023, **27(S1)**: P050

**Introduction:** Altered metabolism and inflammation was reported to be associated with activated glycolysis and induced production of lactate. Although as a hallmark in the development of sepsis, the lactate concentration in bronchoalveolar fluid (BALF) in acute respiratory distress syndrome (ARDS), which was characterized by pulmonary endothelial injury due to inflammation, has not be widely investigated.

**Methods:** BALF were collected from patients with ARDS and post-operative, non-ARDS, patients. Lactate and inflammatory mediators including interleukin (IL)-1beta, IL-6, IL-4, IL-10, endothelial adhere molecules including von Willebrand Factor (vWF) and nitric oxide (NO), were evaluated in BALF supernatant by fluorescent immunoassays. Mechanical ventilation and ICU stay days, and mortality was also recorded. All the patients were followed-up to 90 days with dead or alive recorded.

**Results:** A total of 28 patients with ARDS and 8 patients with non-ARDS were enrolled in this study. The results indicated that the normalized BALF lactate level in ARDS patients was significantly higher compared with non-ARDS patients (mmol/l, 20.6[13.8, 34.3] vs. 11.04[5.2, 21.4], *p* = 0.048), IL-1beta (ng/ml, 3277[653.5, 13865] vs. 537.7[337.9, 780.6], *p* = 0.027), IL-6 (ng/ml, 1798[689, 4136] vs. 65[48, 203], *p* < 0.001), IL-10 (mmol/l, 33.8[24.3, 68.2] vs. 14.6[7.3, 19.1], *p* < 0.001). BALF lactate was also associated with mortality in ARDS patients (HR 4.8, 95% CI 0.9–23.7, log-rank *p* = 0.0167), by a cut-off value of adjusted BALF lactate.

**Conclusions:** BALF lactate was elevated in ARDS patients, and was associated with inflammatory cytokines, and endothelial adherence molecules. BALF lactate was also associated with mortality in ARDS patients.

## P051

### Reduced concentration of IL-8 in the brainstem in moderate-ARDS pigs ventilated for 12 h with diaphragm neurostimulation

#### T Bassi^1^, E Rohrs^2^, K Fernandez^2^, M Ornoskwa^2^, M Nicholas^2^, J Witmann^2^, M Gani^1^, D Evans^1^, S Reynolds^2^

##### ^1^Lungpacer Medical Inc., Burnaby, Canada, ^2^ Royal Columbian Hospital, New Westminster, Canada

*Critical Care* 2023, **27(S1)**: P051

**Introduction:** Studies have shown that mechanical ventilation (MV) and acute respiratory distress syndrome (ARDS) are associated with neuroinflammation.IL-8 is a proinflammatory cytokine associated with cognitive impairment. Temporary transvenous diaphragm neurostimulation (TTDN) in association with MV has been shown to mitigate hippocampal neuroinflammation and apoptosis in preclinical models. This study focused on investigating whether 12 h of MV with moderate ARDS would lead to neuroinflammation, measured as IL-8 tissue concentration in the brainstem, and whether diaphragm neurostimulation in association with MV would affect IL-8 brainstem tissue concentration.

**Methods:** Juvenile pigs (4–5 months, 50–87 kg) underwent protective MV (volume control, PEEP 5 cmH_2_O, tidal volume 8 ml/kg) for 12 h, after being induced with moderate ARDS by injecting oleic acid into the pulmonary artery until PaO_2_/FiO_2_ was between 100 and 200. Subjects were assigned to three groups (n = 6 per group): lung injury with MV only (MV), lung injury with MV and with TTDN every other breath (MV + TTDN50%), and lung injury with MV and with TTDN every breath (MV + TTDN100%). Diaphragm neurostimulation was delivered using methods published previously. After the study, the brainstem (pons and medulla oblongata) was harvested and marked with ELISA to measure the tissue concentration of IL-8. The Kruskal–Wallis test was used for statistical analyses. *p* values < 0.05 are considered statistically significant.

**Results:** Brainstem IL-8 concentrations were 36 pg/ml (28–48) for the MV group, 29 pg/ml (18–64), for the MV + TTDN50% group, and 0 pg/ml (0–18) for the MV + TTDN100% group, with a significant difference between the MV and the MV + TTDN100% groups, *p* = 0.0319 (Fig. 1).

**Conclusions:** In a preclinical model of moderate ARDS, diaphragm neurostimulation on every breath in association with MV for 12 h led to significantly lower concentrations of IL-8 in the brainstem, compared to the group receiving MV, only, for 12 h.

**Figure Figt:**
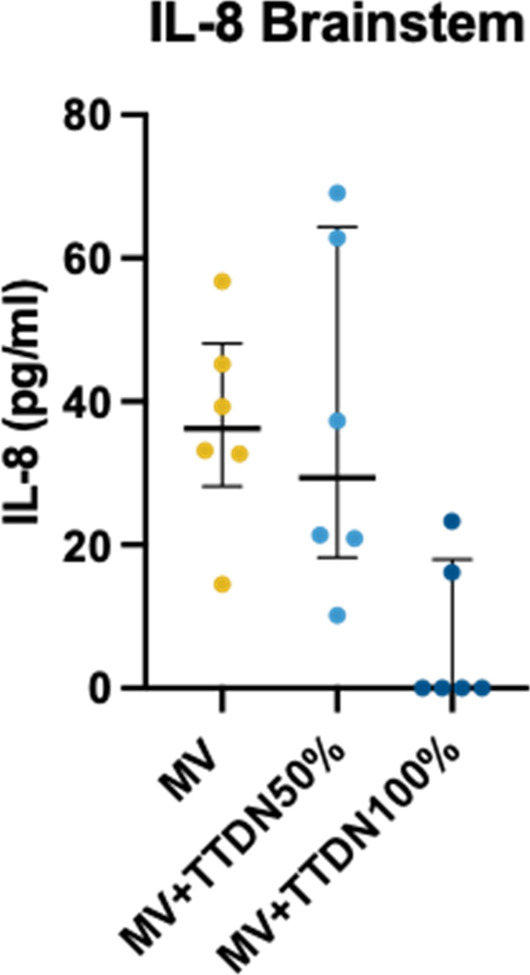
**Fig. 1 (abstract P051)**. Brainstem IL-8 concentrations

## P052

### Alveolar macrophage-derived secretory autophagosomes exacerbate inflammation in lipopolysaccharide-induced acute respiratory distress syndrome

#### X Xu, L Liu, Y Yang

##### Jiangsu Provincial Key Laboratory of Critical Care Medicine, Department of Critical Care Medicine, Zhongda Hospital, School of Medicine, Southeast University, Jiangsu Provincial Key Laboratory of Critical Care Medicine, Department of Critical Care Medicine, Zhongda Hospital, School of Medicine, Southeast University, Nanjing, China

*Critical Care* 2023, **27(S1)**: P052

**Introduction:** This study investigated the effects and the mechanisms through which alveolar macrophage(AM) -derived secretory autophagosomes (SAPs) contribute to acute respiratory distress syndrome(ARDS)-associated lung injury and inflammation.

**Methods:** SAPs were isolated from cell culture supernatants of AMs treated or untreated with lipopolysaccharide (LPS) and intratracheally injected to determine whether SAP exacerbated lung injury in mice with ARDS. The SAP proteome was analysed using mass spectrometry. Lung fibroblasts were treated with SAPs and collected for transcriptome sequencing. A transwell assay was conducted to study the chemotactic activity of fibroblasts induced by SAPs.

**Results:** We found that AMs contributed to ARDS-associated lung injury by releasing a novel type of pro-inflammatory vesicles named SAPs, which were characterised as double-membraned vesicles approximately 200 nm in diameter and light-chain-3 expression. Proteomic analysis of SAPs and gene ontology (GO) enrichment analysis revealed that LPS-SAPs contain several differentially expressed proteins involved in effecting an inflammatory response. In addition, we conducted transcriptome sequencing of lung fibroblasts treated with SAPs to explore the effect of SAPs on fibroblasts in ARDS, which demonstrated that the expression of several chemokines (chemokine ligand (CXCL)2, CXCL15, and so forth) significantly increased after SAP stimulation. Furthermore, the transwell assay indicated that SAPs could enhance the chemotactic activity of fibroblasts, suggesting that SAPs may exacerbate inflammation in ARDS by promoting neutrophil or monocyte recruitment.

**Conclusions:** This is the first study to show that AM-derived SAPs contribute significantly to ARDS through promoting inflammatory cell recruitment by activating fibroblasts, which may be beneficial for therapeutic development of ARDS.

## P053

### Comparison of effect of chest percussion and expiratory rib cage compression on blood gases and static lung compliance in mechanically ventilated patients with acute respiratory distress syndrome: a randomised crossover study

#### A Srivastava^1^, R Kumar ^1^, M Gurjar ^1^, B Poddar ^1^, A Azim ^1^, S Gutte ^1^, P Mishra ^2^, V Kapoor^2^

##### ^1^Sanjay Gandhi Postgraduate Institute of Medical Sciences, Critical Care Medicine, Lucknow, India, ^2^Sanjay Gandhi Postgraduate Institute of Medical Sciences, Biostatistics, Lucknow, India

*Critical Care* 2023, **27(S1)**: P053

**Introduction:** This study compared the effect of two techniques of chest physiotherapy, chest percussion (CP) and expiratory rib cage compression (ERCC), on blood gases (PaO_2_ and PaCO_2_), static lung compliance (Cstat) and secretion yield in mechanical ventilated patients with moderate-severe acute respiratory distress syndrome (ARDS).

**Methods:** After institutional ethics committee approval, all adult ICU patients with moderate-severe ARDS on invasive ventilation were screened for this randomized crossover study (CTRI/2022/01/039650). Exclusion criteria were: age < 18/ > 65 years, suspected/confirmed raised intracranial pressure, acute coronary syndrome, arrhythmias, abdominal compartment syndrome, pneumothorax or pregnancy. Included patients received both the techniques on the same day, six hours apart. PaO_2_ and PaCO_2_ (mmHg) and Cstat (ml/cmH_2_O) were obtained at baseline, 20 and 60 min after each physiotherapy technique. Tracheal secretion yield measurement was also done after both techniques.

**Results:** During study period (Jan–Oct 2022), 212 patients screened and 19 included with median age 49 (33–59) years and 9 were males. On the study day, median SOFA score was 10 (8–10) and median PaO_2_/FiO_2_ ratio was 155 (130–174). Before CP technique, baseline PaO_2_, PaCO_2_ and Cstat were 80, 41.9 and 24.8 which were changed at 20 min -2.3 (*p* = 0.11), 0.2 (*p* = 0.11) and -1 (*p* = 0.39); and at 60 min -0.4 (*p* = 0.79), -1.3 (*p* = 0.27) and 0.7 (*p* = 0.26) respectively. While baseline values in ERCC were 75.9, 41.5 and 24.3 which changed at 20 min 0 (*p* = 0.79), 0.6 (*p* = 0.39) and 0.2 (*p* = 0.8); and at 60 min 1.1 (*p* = 0.23), 0.6 (*p* = 0.43) and 1 (*p* = 0.26) respectively.The median volume of secretion yield (ml) was 4.0 (3.0–6.0) and 5.0 (3.5–6.0) (*p* = 0.88), respectively.

**Conclusions:** In moderate-severe ARDS patients, both CP and ERCC techniques did not affect arterial blood gases and static lung compliance significantly at either 20 or 60 min (Fig. 1). Also the secretion yield was similar after both techniques.

**Figure Figu:**
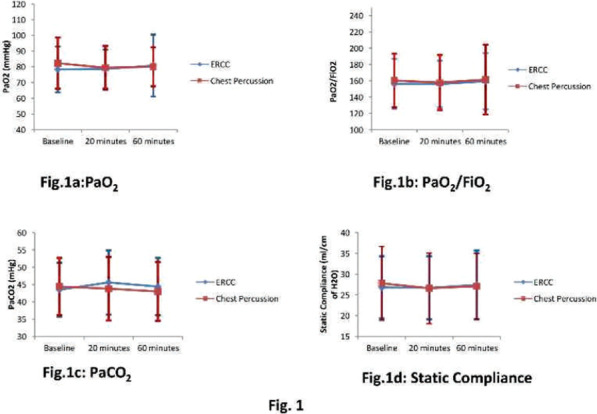
**Fig. 1 (abstract P053)**. Primary outcomes

## P054

### Postoperative acute respiratory distress syndrome in therapeutic randomized controlled trials

#### VG Giannakoulis, E Papoutsi, A Kotanidou, II Siempos

##### First Department of Critical Care Medicine and Pulmonary Services, Evangelismos Hospital, National and Kapodistrian University of Athens Medical School, Athens, Greece

*Critical Care* 2023, **27(S1)**: P054

**Introduction:** Large studies to inform the epidemiology of postoperative acute respiratory distress syndrome (ARDS) are scarce. We, therefore, aimed to perform a secondary analysis of seven high-quality therapeutic randomized controlled trials.

**Methods:** A secondary analysis of participants enrolled in 7 trials of ARDS and PETAL Networks [1–7].

**Results:** We analyzed 5316 patients with ARDS, including 256 with postoperative ARDS. We found that prevalence of postoperative ARDS gradually declined between 2000 and 2011, and it was stabilized afterwards (R^2^ = 0.821). Patients with postoperative ARDS, were older (*p* < 0.001), had more frequently sepsis (*p* < 0.001) and multiple transfusions (*p* < 0.001) as ARDS risk factors and presented lower 90-day mortality when compared to patients with ARDS not related to surgery [24.6% versus 30.9%, adjusted hazard ratio 0.66, 95% confidence intervals (CI) 0.51–0.85, *p* = 0.001]. In adjusted analysis, older age (*p* < 0.001), immunosuppression (*p* = 0.009), and increased cumulative fluid balance during the first 3 days (*p* < 0.001) were factors associated with 90-day mortality of patients with postoperative ARDS.

**Conclusions:** Our findings put the epidemiology and clinical features of postoperative ARDS in perspective, and therefore, fill a missing gap in the literature. We also reveal that increased fluid balance (a potentially modifiable, practice-dependent factor) is associated with higher 90-day mortality in postoperative ARDS. Therefore, patients with postoperative ARDS may benefit from a conservative fluid management strategy.


**References**
Brower RG et al. N Engl J Med. 2000;342:1301–8.Brower RG et al. N Engl J Med. 2004;351:327–36.Wiedemann HP et al. N Engl J Med. 2006;354:2564–75.Matthay MA et al. Am J Respir Crit Care Med. 2011;184:561–8.Rice TW et al. JAMA. 2012;307:795–803.Truwit JD et al. N Engl J Med.2014;370:2191–200.Moss M et al. N Engl J Med.2019;380:1997–2008.


## P055

### Ultra lung protective ventilation and biotrauma in severe ARDS patients on veno-venous extracorporeal membrane oxygenation: a randomized controlled study

#### C Guervilly^1^, T Fournier^2^, J Chommeloux^3^, S Valera^2^, R Lacroix^4^, S Hraiech^2^, A Roch^2^, M Schmidt^3^, L Papazian^5^

##### ^1^Médecine Intensive Réanimation, Hôpital Nord, APHM, Intensive Care Unit, Marseille, France, ^2^Médecine Intensive Réanimation, Hôpital Nord, APHM, Marseille, France, ^3^Service de Médecine Intensive-Réanimation, Institut de Cardiologie, APHP, Sorbonne Université Hôpital Pitié- Salpêtrière, Paris, France, ^4^Laboratoire d’Hématologie et de Biologie Vasculaire, APHM,Université Aix-Marseille, INSERM 1263, Institut National de Recherche pour l’Agriculture, l’Alimentation et l’Environnement, Centre de Recherche en CardioVasculaire et Nutrition, Marseille, France, ^5^Centre Hospitalier de Bastia, Service de Réanimation, Bastia, France

*Critical Care* 2023, **27(S1)**: P055

**Introduction:** Ultra-lung-protective ventilation may be useful during veno-venous extracorporeal membrane oxygenation (vv-ECMO) for severe acute respiratory distress syndrome (ARDS) to minimize ventilator-induced lung injury and to facilitate lung recovery. Objective was to compare pulmonary and systemic biotrauma evaluated by numerous biomarkers of inflammation, epithelial, endothelial injuries, and lung repair according to two ventilator strategies on vv-ECMO.

**Methods:** Prospective randomized controlled study. Patients were randomized to receive during 48 h either ultra-lung-protective ventilation combining very low tidal volume (1–2 ml/kg of predicted body weight), low respiratory rate (5–10 cycles per minute), positive expiratory transpulmonary pressure, and 16 h of prone position or lung-protective-ventilation which followed the ECMO arm of the EOLIA trial (control group) (Fig. 1).

**Results:** The primary outcome was the alveolar concentrations of interleukin-1-beta, interleukin-6, interleukin-8, surfactant protein D, and blood concentrations of serum advanced glycation end products and angiopoietin-2 48 h after randomization. Enrollment was stopped for futility after the inclusion of 39 patients. Tidal volume, respiratory rate, minute ventilation, plateau pressure, and mechanical power were significantly lower in the ultra-lung-protective group. None of the concentrations of the pre-specified biomarkers differed between the two groups 48 h after randomization. However, a trend to higher 60-day mortality was observed in the ultra-lung-protective group compared to the control group (45 vs 17%, *p* = 0.06).

**Conclusions:** Despite a significant reduction of the mechanical power, ultra-lung-protective ventilation during 48 h did not reduce biotrauma in patients with vv-ECMO-supported ARDS. The impact of this ventilation strategy on clinical outcomes warrants further investigation.

**Figure Figv:**
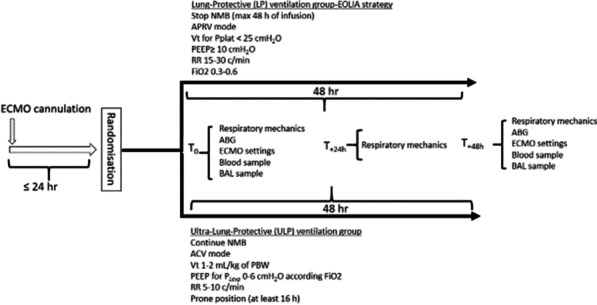
**Fig. 1 (abstract P055)**. Study design

## P056

### Pulmonary endothelial cells-derived extracellular vesicles promote neutrophil extracellular trap formation during ARDS

#### SF Z, XJ Wu, HB Qiu

##### Zhongda Hospital, School of Medicine, Southeast University, Department of Critical Care Medicine, Nanjing, China

*Critical Care* 2023, **27(S1)**: P056

**Introduction:** The acute respiratory distress syndrome (ARDS) is a common cause of respiratory failure in critically ill patients which causes high mortality, but its molecular mechanisms are poorly understood. Neutrophil extracellular traps (NETs) originate from decondensed chromatin released to immobilize pathogens, and they can trigger contribute to sustained inflammation. However, their role in NET formation remains unclear. Extracellular vesicles (EVs) mediate intercellular communication between cells in physiological and pathological conditions. Our previous studies have demonstrated that EVs released during severe bacterial pneumonia were inflammatory and induced acute lung injury. Moreover, Endothelial cell (EC)-derived EVs increased markedly. Study has shown that EVs sustain NET formation. However, whether EC-derived EVs play a role in NET formation in ARDS has yet to be addressed.

**Methods:** Polymorphonuclear neutrophils (PMNs) were cocultured with EVs isolated from the supernatant of pulmonary microvascular ECs stimulated with phosphate buffer saline (PBS) or lipopolysaccharide (LPS). A cecal ligation and puncture (CLP) mouse model was used to mimic septic ARDS in vivo; then, NET formation and molecular pathways were detected by transcriptome of EVs-Treated PMNs and mass spectrometry of EVs.

**Results:** In the animal CLP model, EVs depletion reduced NET formation, lung inflammation and injury. NET components were significantly increased in response to treatment with PMECs-derived EVs in vitro and vivo. NETs inhibition reduced lung inflammation and lung injury after EVs injection intravenously. Mechanistic studies demonstrated that exosomal mitochondrial associated protein induced NET formation through the Fpr1 mediated-NF-KB pathway.

**Conclusions:** EC-derived EVs promote excessive NET formation and aggravate inflammation and subsequent lung injury. This finding suggests a previously unidentified role of EC-derived exosomes in ARDS and may lead to new therapeutic approaches.

## P057

### Macrophage-derived LC3 positive extracellular vesicles induce lung microvascular endothelial cell- mediated excessive inflammatory by transferring tRF-1024

#### X Zhu, L Liu

##### Southeast University, Jiangsu Provincial Key Laboratory of Critical Care Medicine, Nanjing, China

*Critical Care* 2023, **27(S1)**: P057

**Introduction:** Acute respiratory distress syndrome (ARDS) is an unsolved clinical conundrum globally due to its elusive pathogenesis. Pulmonary microvascular endothelial cells (PMVECs) activation and injury are the hallmarks of ARDS pathogenesis. Thus, the elucidation of endothelial activation mechanisms might uncover new approaches for treating ARDS. Extracellular vesicles (EVs) have emerged as an important means of cell–cell signaling by loading nucleic acid and other cargo. tRNA-derived small RNA (tsRNA) is an emerging class of small non-coding RNA molecules that involve in the regulation of multiple physiological processes and diseases. This study describes macrophages-derived LC3^+^EV mediate PMVECs activation in ARDS and how this process is regulated by LC3^+^EVs carried-tsRNA.

**Methods:** Flow cytometry absolute count standard was used to quantify LC3^+^EV in alveolar lavage fluid of patients from ARDS and control groups. Intratracheal instillation model was applied to investigate the effects of endothelial function activation with LC3^+^EV treatment. RNA sequencing was used to identify the critical tsRNA related to endothelial function. Further evaluation of tsRNA functional was determined through transfecting mimics.

**Results:** Clinically, the number of LC3^+^EVs had a good predictive value for 28-day mortality of ARDS patients, indicating that LC3^+^EV is a critical mediator of acute lung injury. Functionally, LC3^+^EVs up-regulated the level of PMVECs adhesion molecules and increased neutrophil infiltration in vitro and in vivo experiments. Mechanistically, we found the tRF-1024 was closely related to endothelial adhesion function in LC3^+^EVs and overexpression of tRF-1024 enhanced neutrophil adhesion of PMVECs.

**Conclusions:** Our study uncovers a novel LC3^+^EV carried-tRF1024 mechanism for the rapid recruitment of neutrophils by PMVEC activation. These findings provide novel insights into PMVEC activation and open a new avenue for developing novel therapeutic strategies in ARDS.

## P058

### BM-MSCs attenuate LPS-induced acute respiratory distress syndrome by promoting resolvin E1/protectin D1

#### S Yang^1^, J Liu^1^, C Li^2^, YY Pan^2^

##### ^1^Department of Critical Care Medicine, The Affiliated Suzhou Hospital of Nanjing Medical University, Suzhou Municipal Hospital, Suzhou, China, ^2^Department of Critical Care Medicine, Suzhou, China

*Critical Care* 2023, **27(S1)**: P058

**Introduction:** Acute lung injury (ALI) and its severe form acute respiratory distress syndrome (ARDS) are respiratory failure caused by excessive alveolar inflammation with high mortality. In this study, we investigated effects of bone morrow mesenchymal stem cells (BM-MSCs) on lung injury of lipopolysaccharide (LPS)-induced ALI and explored the associated mechanisms.

**Methods:** BM-MSCs were isolated, cultured, identified by staining with CD34 and CD44 surface markers. LPS-induced ALI mouse model was generated by injecting with LPS and divided into ALI group and ALI + BM-MSCs group. Mice treated without any reagents were assigned as Control, mice transplanted with BM-MSCs were assigned as BM-MSCs group. Regulatory T (Treg) and Th17 percentages were evaluated using flow cytometry. Pro-resolving mediators (resolvin E1 (RvE1), protectin D1 (ProD1)) in lung tissue and cytokines (interleukin-6 (IL-6) and IL-17) in serum were analyzed by ELISA. Myeloperoxidase (MPO) activity was determined. Cultured cells demonstrated typical characteristics of BM-MSCs.

**Results:** BM-MSCs transplantation (ALI + BM-MSCs) obviously alleviated LPS-induced ALI in mice. BM-MSCs transplantation significantly decreased MPO activity in LPS-induced ALI in mice compared to Control group (*p* < 0.05). BM-MSCs transplantation markedly increased Treg percentages, and decreased dendritic cells (DCs) and Th17 cells percentages compared to those of Control group (*p* < 0.05). BM-MSCs transplantation remarkably enhanced RvE1 and ProD1 levels in LPS-induced ALI (ALI + BM-MSCs) compared to ALI group (*p* < 0.05). BM-MSCs transplantation significantly attenuated IL-6 and IL-17 levels in serum of mice treated with LPS (ALI + BM-MSCs) compared to those of ALI group (*p* < 0.05).

**Conclusions:** BM-MSCs transplantation effectively attenuated LPS-induced pathological injury of ALI in mice, at least partly through promoting pro-resolving mediators RvE1 and ProD1 and modulating balance of Treg/Th17.

## P059

### Derivation and validation of clinical phenotypes and associated treatment responses in critically ill COVID-19 patients

#### N Bruse^1^, A Motos^2^, R Van Amstel^3^, E De Bie ^1^, J Kennedy^4^, N De Keizer^5^, L Van Vught^3^, A Torres^2^, P Pickkers^1^, M Kox^1^

##### ^1^Radboudumc, Department of Intensive Care Medicine, Nijmegen, Netherlands, ^2^Institute of Health Carlos III, Pulmonary Department, Madrid, Spain, ^3^Academic Medical Center, Department of Intensive Care Medicine, Amsterdam, Netherlands, ^4^University of Pittsburgh, Department of Anesthesiology and Intensive Care Medicine, Pittsburgh, USA, ^5^Academic Medical Center, National Intensive Care Evaluation (NICE) Foundation, Amsterdam, Netherlands

*Critical Care* 2023, **27(S1)**: P059

**Introduction:** Due to variability in the host response, a uniform treatment strategy for severe COVID-19 may be inadequate. We applied unsupervised clustering methods to large cohorts of COVID-19 ICU patients to derive and validate clinical phenotypes, and to explore treatment responses in these phenotypes.

**Methods:** Phenotypes were derived in 13.279 critically ill COVID-19 patients admitted to 82 Dutch ICUs from September 2020 to February 2022. Twenty-one features were selected from clinical characteristics measured within 24 h after ICU admission. Phenotypes were assigned using consensus *k* means clustering. External validation was performed in 6225 critically ill COVID-19 patients admitted to 55 Spanish ICUs from February 2020 to December 2021. Individual patient data on corticosteroids therapy enabled us to investigate phenotype-specific responses in this cohort.

**Results:** Three distinct clinical phenotypes were derived (Fig. 1A). Patients with phenotype 1 (43%) were younger, had lower APACHE IV scores, higher BMI as well as a lower P/F ratio and 90-day in-hospital mortality (18%, Fig. 1A). Phenotype 2 patients (37%) were older and had slightly higher APACHE IV scores compared with phenotype 1, a lower BMI, and higher mortality compared to phenotype 1 (24%, *p* = 2.95e−07). Phenotype 3 (20%) included the oldest patients with the most comorbidities and highest APACHE IV scores, severe renal and metabolic impairment, and the worst outcome (47% mortality, *p* = 6.6e−16 and *p* = 6.6e−16 versus phenotypes 1 and 2, respectively). Phenotype distribution and outcome were very similar in the validation cohort (Fig. 1B). This cohort also revealed that corticosteroid therapy only benefited phenotype 3 (65% vs. 54% mortality, *p* = 2.5e−03, Fig. 1C).

**Conclusions:** COVID-19 ICU phenotypes based on clinical data are related to outcome and treatment responses. This can inform treatment decisions as well as randomized trials employing precision medicine approaches.

**Figure Figw:**
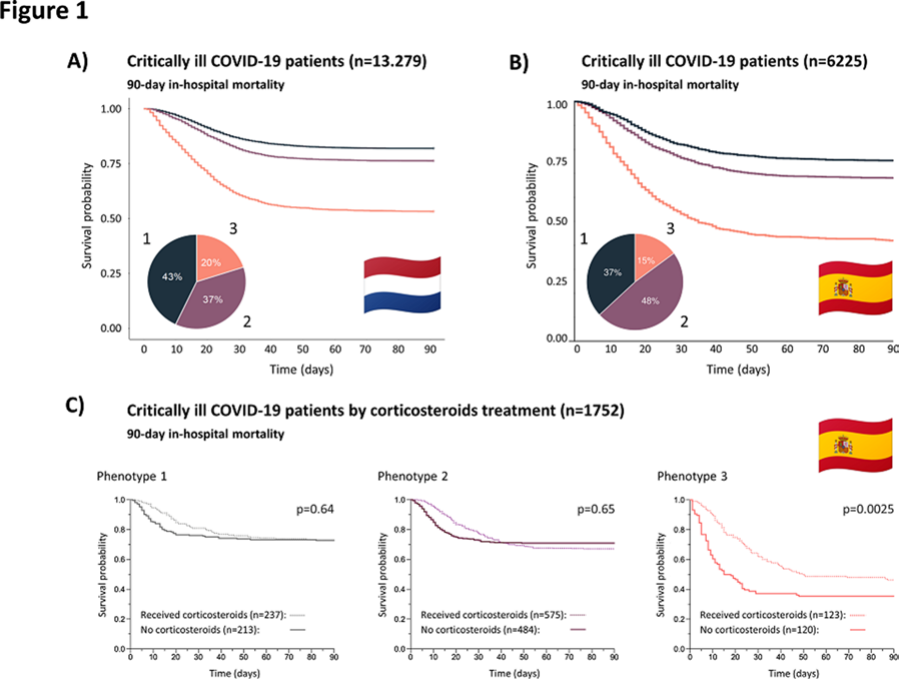
**Fig. 1 (abstract P059)**. Phenotype distribution and 90-days in-hospital mortality for critically ill COVID-19 patients in the derivation (**A**) and validation (**B**) cohorts. **C** Comparison of 90-days in-hospital mortality for the three phenotypes in the validation cohort between patients who did and did not receive corticosteroid treatment after the first 24 h of ICU admission. *p* values were calculated using log-rank tests. COVID-19: coronavirus disease 2019

## P060

### Machine learning uncovers blood test patterns subphenotypes at hospital admission discerning increased 30-day ICU mortality rates in COVID-19 elderly patients.

#### NRG Romero-Garcia^1^, LZ Zhou^2^, RB Badenes^1^, TGM Garcia-Morales^2^, AGC Gomez-de la Camara^2^, FGR Garcia-Ruiz^2^, JMGG Garcia-Gomez^2^, CS Saez^2^

##### ^1^Hospital Clinico Universitario de Valencia, Anaesthesiology and Critical Care, Valencia, Spain, ^2^Universidad Politécnica de Valencia, Valencia, Spain

*Critical Care* 2023, **27(S1)**: P060

**Introduction:** Since March 2020, a number of SARS-CoV-2 patients have frequently required intensive care unit (ICU) admission, associated with moderate survival outcomes and an increasing economic burden. Elderly patients are among the most numerous, due to previous comorbidities and complications they develop during hospitalization [1]. For this reason, a reliable early risk stratification tool could help estimate an early prognosis and allow for an appropriate resources allocation in favour of the most vulnerable and critically ill patients.

**Methods:** This retrospective study includes data from two Spanish hospitals, HU12O (Madrid) and HCUV (Valencia), from 193 patients aged > 64 with COVID-19 between February and November 2020 who were admitted to the ICU. Variables include demographics, full-blood-count (FBC) tests and clinical outcomes. Machine learning applied a non-linear dimensionality reduction by t-distributed stochastic neighbor embedding (t-SNE) [2]; then hierarchical clustering on the t-SNE output was performed. The number of clinically relevant subphenotypes was chosen by combining silhouette and elbow coefficients, and validated through exploratory analysis.

**Results:** We identified five subphenotypes with heterogeneous inter-clustering age and FBC patterns (Fig. 1). Cluster 1 was the ‘healthiest’ phenotype, with 2% 30-day mortality and characterized by moderate leukocytes and eosinophils. Cluster 5, the severe phenotype, showed 44% 30-day mortality and was characterized by the highest leukocyte, neutrophil and platelet count and minimal monocytes and lymphocyte count. Clusters 2–4 displayed intermediate mortality rates (20–28%).

**Conclusions:** The findings of this preliminary report of Eld-ICU-COV19 patients suggest the patient’s FBC and age can display discriminative patterns associated with disparate 30-day ICU mortality rates.


**References**
COVID-ICU Group. Intensive Care Med. 2021;47:60–73.Van der Maaten L et al. J Mach Learn Res. 2008;9:2579–605.

**Figure Figx:**
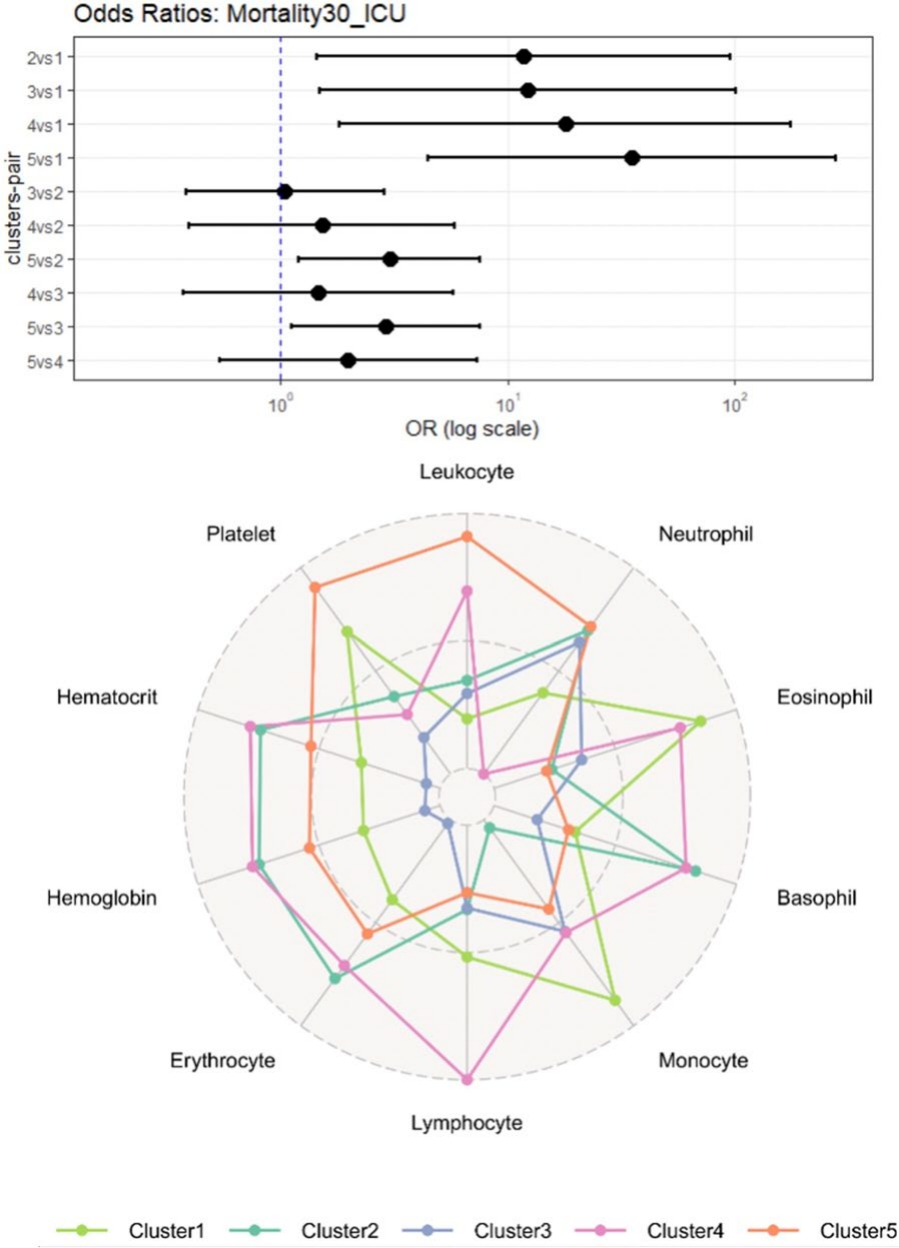
**Fig. 1 (abstract P060)**. Differences in standardised average blood cell counts between the five Eld-ICU-COV19 subphenotypes

## P061

### Latent class analysis of imaging and clinical respiratory parameters from patients with COVID-19-related ARDS identifies recruitment subphenotypes

#### DFL Filippini^1^, E Di Gennaro^2^, RBE Van Amstel ^1^, LFM Beenen^3^, S Grasso^4^, L Pisani^5^, LDJ Bos^6^, MR Smit^1^

##### ^1^Amsterdam UMC, Intensive Care, Amsterdam, Netherlands, ^2^University of Bari, Faculty of Medicine, Bari, Italy, ^3^Amsterdam UMC, Radiology and Nuclear Medicine, Amsterdam, Netherlands, ^4^University of Bari, Emergency and Organ Transplantation, Bari, Italy, ^5^Mahidol Oxfornd Research Unit, Critical Care Africa Asia Network, Bangkok, Thailand, ^6^Amsterdam UMC, Intensive Care, Plumonology, LEICA, Amsterdam, Netherlands

*Critical Care* 2023, **27(S1)**: P061

**Introduction:** Patients with COVID-19-related acute respiratory distress syndrome (ARDS) require respiratory support with invasive mechanical ventilation and show varying responses to recruitment manoeuvres. In patients with ARDS not related to COVID-19, two pulmonary subphenotypes that differed in recruitability were identified using latent class analysis (LCA) of imaging and clinical respiratory parameters [1]. We aimed to validate these phenotypes and evaluate if similar subphenotypes are present in patients with COVID-19-related ARDS.

**Methods:** This is the retrospective analysis of mechanically ventilated patients with COVID-19-related ARDS who underwent CT scans at positive end-expiratory pressure of 10 cmH_2_O and after a recruitment manoeuvre at 20 cmH_2_O. LCA was applied to quantitative CT-derived parameters, clinical respiratory parameters, blood gas analysis and routine laboratory values before recruitment to identify subphenotypes.

**Results:** 99 patients were included. Using 12 variables, a two-class LCA model was identified as best fitting. Subphenotype 2 (*recruitable*) was characterized by a lower PaO_2_/FiO_2_, lower normally aerated lung volume and lower compliance as opposed to a higher non-aerated lung mass and higher mechanical power when compared to subphenotype 1 (*non-recruitable*) (Fig. 1). Patients with subphenotype 2 had more decrease in non-aerated lung mass in response to a standardized recruitment manoeuvre (*p* = 0.024) and were mechanically ventilated longer until successful extubation (adjusted SHR 0.46, 95% CI 0.23–0.91, *p* = 0.026), while no difference in survival was found (*p* = 0.814).

**Conclusions:** A *recruitable* and *non-recruitable* subphenotype were identified in patients with COVID-19-related ARDS. The subphenotypes are similar to non-COVID-19-related ARDS and are promising for identification of recruitable patients in future practice as they can be classified with only few clinically available parameters before the recruitment manoeuvre.


**Reference**
Wendel Garcia PD et al. Crit Care 2021;25:154

**Figure Figy:**
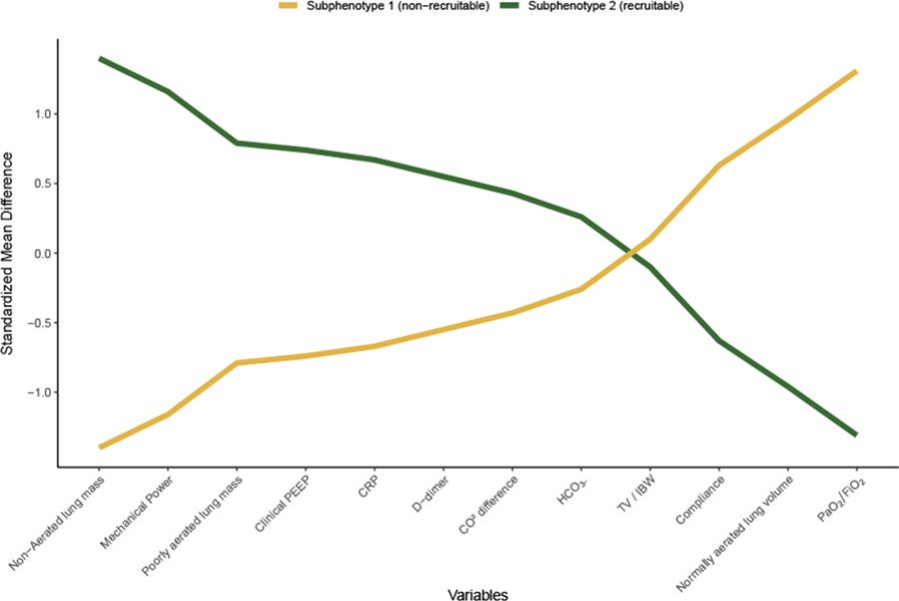
**Fig. 1 (abstract P061)**. Profile plot of the two subphenotypes identified by the latent class analysis (LCA). All variables used in the LCA are plotted on the x-axis, with the y-axis displaying the standardized mean difference (SMD) of the corresponding variables in both of the LCA derived subphenotypes. SMDs are calculated by standardizing the variable to a mean of 0 and a standard deviation of 1. The variables on the x-axis are ordered by the y-value of the recruitable phenotype in a descending way

## P062

### Distinct clinical phenotypes of critically ill COVID-19 patients: a cohort observational study

#### JP Cidade^1^, VSD Cés de Sousa Dantas^2^, AF Thompson^3^, R Miranda^3^, P Póvoa^1^

##### ^1^Hospital São Francisco Xavier, Intensive Care Department, Lisbon, Portugal, ^2^Instituto D´Or de Pesquisa e Ensino, Intensive Care Department, Rio de Janeiro, Brazil, ^3^Hospital Copa D´Or, Intensive Care Department, Rio de Janeiro, Brazil

*Critical Care* 2023, **27(S1)**: P062

**Introduction:** COVID-19 presents a complex pathophysiology and evidence collected points towards an intricated interaction of viral-dependent and individual immunological mechanisms. The identification of phenotypes, through clinical and biological markers, may provide a better understanding of the subjacent mechanisms and an early patient-tailored characterization of illness severity.

**Methods:** Multicenter prospective cohort study performed in 5 hospitals of Portugal and Brazil, during one year, between 2020–2021. All adult patients with an Intensive Care Unit admission with SARS-CoV-2 pneumonia were eligible. COVID-19 was diagnosed using clinical and radiologic criteria with a SARS-CoV-2 positive RT-PCR test. A two-step hierarchical cluster analysis was made using several class-defining variables.

**Results:** 814 patients were included. The cluster analysis revealed a three-class model, allowing for the definition of three distinct COVID-19 phenotypes: 244 patients in phenotype A, 163 patients in phenotype B, and 407 patients in phenotype C. Patients included in the phenotype C were significantly older, with higher baseline inflammatory biomarkers profile, and significantly higher requirement of organ support and mortality rate (Table 1 (abstract P062)). Phenotypes A and B demonstrated some overlapping clinical characteristics but different outcomes. Phenotype B patients presented a lower mortality rate, with consistently lower C-reactive protein, but higher procalcitonin and interleukin-6 serum levels, describing an immunological profile significantly different from phenotype A (Table 1).

**Conclusions:** Severe COVID-19 patients exhibit three different clinical phenotypes with distinct profiles and outcomes. Their identification could have an impact in patients’ care, justifying different therapy responses and inconsistencies identified across different randomized control trials results.

**Table Tabo:** **Table 1 (abstract P062)**. Demographic, clinical and outcome variables of patients according to the COVID-19 phenotype

	PHENOTYPE A (n = 244; 24%)	PHENOTYPE B (n = 163; 16%)	PHENOTYPE C (n = 407; 60%)	*p*
Age, years (median (IQR))	62 (47–79)	63 (47–80)	81 (65–97)	< 0.001
SOFA at admission (median (IQR))	3 (2;5)	1 (0; 3)	10 (5; 13)	< 0.001
Max C-Reactive protein, mg/dL (mean ± SD)	25.3 ± 10.4	18.6 ± 12.5	32.3 ± 11.0	< 0.001
Max registered Procalcitonin, ng/mL (median (IQR))	0.34 (0.06; 0.74)	1.30 (0.70; 1.40)	9.73 (0.86;13.54)	< 0.001
IL-6 serum levels, (median (IQR))	35.4 (6.6; 42.7)	41.0 (16.0; 49.0)	57.9 (5.7; 61.0)	0.01
ICU length of stay, days, (median (IQR))	6 (5; 11)	10 (3; 10)	14 (11; 15)	< 0.001
Mortality, (n, %)	17 (7.0%)	2 (1.2%)	49 (12.0%)	0.007

## P063

### Comparison of the efficacy of tocilizumab and itolizumab for the treatment of severe COVID-19: a retrospective cohort study

#### A Kumar, N Kumar, A Kumar, A Pattanayak

##### All India Institute of Medical Sciences Patna, Anaesthesiology, Patna, India

*Critical Care* 2023, **27(S1)**: P063

**Introduction:** Itolizumab, a CD6 inhibitor has been found to be effective in COVID-19 in some studies [1] but there is no randomised controlled trial at present to prove its effectiveness.

**Methods:** The study population was adults (> 18 years) with severe COVID-19 pneumonia admitted in the ICU who received either tocilizumab or itolizumab in their course of stay in ICU. The primary outcome of the study was a clinical improvement (CI). The secondary outcomes were time for clinical improvement, improvement in PO_2_/FiO_2_ ratio, best PO_2_/FiO_2_ ratio, need for mechanical ventilation (MV) after administration of study drugs, time to discharge and survival days.

**Results:** 126 patients were included in the study; 92 received tocilizumab, and 34 received itolizumab. CI was seen in 46.7% and 61.7% of the patients in the tocilizumab and itolizumab groups, respectively and was statistically non-significant. The time to CI was also non-significant between the tocilizumab and itolizumab groups (median 12 vs 11 days). The number of days required to achieve the improvement of 100 in the PO_2_/FiO_2_ ratio was significantly less with itolizumab as compared to tocilizumab. (6 vs 8 days, *p* value = 0.028). The best PO_2_/FiO_2_ ratio achieved was also significantly better with itolizumab as compared to tocilizumab (315 vs 250, *p* value = 0.043). The incidence of serious adverse events due to the study drugs was significantly higher with itolizumab as compared to tocilizumab (14.7 vs 3.26%). The estimated median time for CI was 12 days and 11 days in the tocilizumab and itolizumab groups, respectively and was non-significant (log-rank *p* value = 0.262) (Fig. 1).

**Conclusions:** The clinical improvement and survival rates with itolizumab are similar to tocilizumab. Better oxygenation can be achieved with itolizumab and can be a substitute for tocilizumab in managing severe COVID-19 infection.


**Reference**
Kumari P et al. Indian J Crit Care Med 2021;25:467–469.

**Figure Figz:**
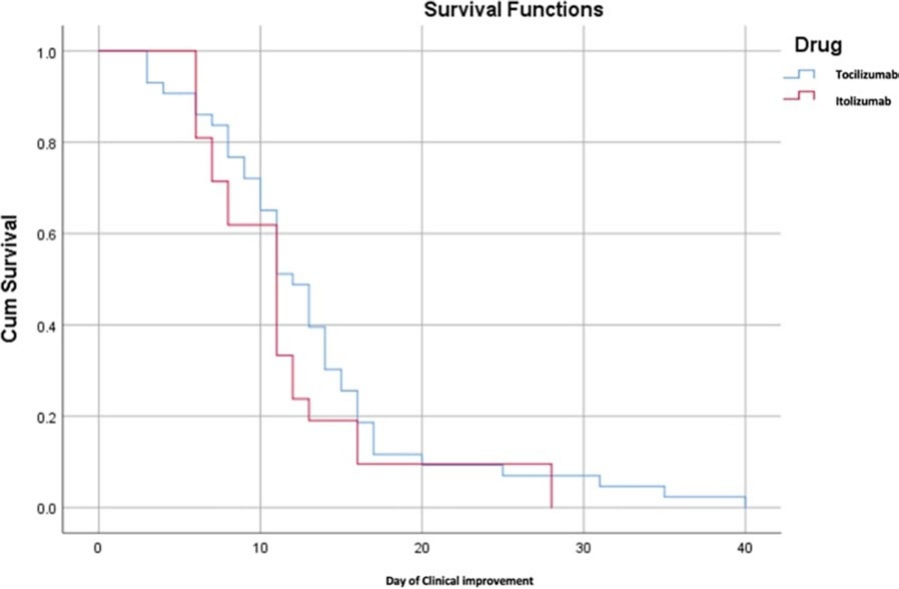
**Fig. 1 (abstract P063)**. Kaplan–Meier estimate of the cumulative probability of clinical improvement by treatment group

## P064

### Stress rate in mechanically ventilated patients with ARDS: a novel concept of ventilator induced lung injury

#### S Redaelli^1^, E Costa^2^, D Von Wedel^1^, A Suleiman^1^, R Munoz^1^, A Santarisi^1^, D Talmor^1^, M Amato^2^, EN Baedorf-Kassis^3^, MS Schaefer^1^

##### ^1^Beth Israel Deaconess Medical Center, Harvard Medical School, Department of Anesthesia, Critical Care and Pain Medicine, Boston, USA, ^2^Heart Institute (Incor), Hospital das Clínicas da Faculdade de Medicina da Universidade de São Paulo, Laboratório de Pneumologia LIM-09, Disciplina de Pneumologia, Sao Paulo, Brazil, ^3^Beth Israel Deaconess Medical Center, Harvard Medical School, Department of Pulmonary, Critical Care & Sleep Medicine, Boston, USA

*Critical Care* 2023, **27(S1)**: P064

**Introduction:** Excessive lung stress, measured by driving pressure (DP) may result in ventilator-induced lung injury (VILI) and mortality in patients with ARDS [1]. Integrative concepts like mechanical power (MP) have been associated with mortality in ARDS, although it is unclear whether information gained from including respiratory rate (RR) [2] reflects rate of deformation (strain rate), rather than repetition [3]. While strain is difficult to measure in clinical settings, stress rate could be a surrogate, but has not yet been studied.

**Methods:** This secondary analysis included adult patients with ARDS from the EPVent2 trial [4]. We defined stress rate as DP over inspiratory time. We investigated its correlation with dynamic parameters including RR and MP on study day 1. As exploratory analysis, the association of DP and stress rate with 28-day mortality was investigated through logistic regression adjusting for age, sex, BMI and SOFA score.

**Results:** 193 patients were included. Characteristics of patients by high (> 15.3 cmH_2_O/s) and low (≤ 15.3 cmH_2_O/s) stress rate are presented in Table 1. The median (IQR) DP at baseline after study initiation was 12 (10–14) cmH_2_O. Median inspiratory time was 0.8 (0.7–0.9) s and stress rate was 15 (11–19) cmH_2_O/s. Stress rate was highly correlated with DP (r = 0.80, *p* < 0.001) and moderately correlated with RR (r = 0.38, *p* < 0.001). Low level correlation was observed with MP (r = 0.21, *p* = 0.02). In adjusted analyses, stress rate was not associated with 28-day mortality (aOR 1.04 [0.99–1.09], *p* = 0.09; per 1 cmH_2_O/s increase), opposed to DP (aOR 1.11 [1.02–1.21], *p* = 0.02, per 1 cmH_2_O increase).

**Conclusions:** Stress rate, as surrogate for strain rate showed poor correlation with mechanical power, suggesting that incorporating respiratory rate into this concept reflects repetition rather than rate of deformation.


**References**
Amato M et al. N Engl J Med 2015;372:747–755.Schaefer MS et al. Intensive Care Med 2021;47:130–132.Protti A et al. Crit Care Med 2016;44:838–45.Beitler J et al. JAMA 2019;321:846–857.

**Table Tabp:** **Table 1 (abstract P064)**. Patient characteristics and distribution of variables by high (> 15.3 cmH_2_O/s) and low (≤ 15.3 cmH_2_O/s) stress rate

	Stress rate ≤ 15.3 cmH_2_O/s, n = 97	Stress rate > 15.3 cmH_2_O/s, n = 96	StdDiff
Age, years	58.0 (47.0–65.0)	57.5 (42.5–70.5)	− 0.11
Female, n (%)	39 (40%)	49 (51%)	− 0.22
SOFA score	6.0 (4.0–9.5)	9.0 (6.0–10.0)	− 0.41
Airway driving pressure, cmH_2_O	10.0 (8.0–11.0)	14.0 (13.0–17.0)	− 1.58
Inspiratory time, s	0.9 (0.8–1.0)	0.7 (0.6–0.8)	0.45
Respiratory rate, breaths per min	26.0 (21.0–29.0)	30.0 (24.0–34.0)	− 0.59
Mechanical power, J/min	24.9 (20.2–31.0)	31.7 (23.6–38.6)	− 0.34

Data are presented as median (IQR). SOFA, Sequential Organ Failure Assessment

## P065

### Transpulmonary pressure as a predictor of successful lung recruitment: reanalysis of a multicenter international randomized clinical trial

#### A Santarisi^1^, A Suleiman^1^, S Redaelli^1^, D Von Wedel^1^, JR Beitler^2^, D Talmor^1^, V Banner-Goodspeed^1^, B Jung^3^, MS Schaefer^1^, E Baedorf-Kassis^3^

##### ^1^Beth Israel Deaconess Medical Center, Department of Anesthesia, Critical Care and Pain Medicine, Boston, USA, ^2^Columbia University College of Physicians & Surgeons, Center for Acute Respiratory Failure and Division of Pulmonary, Allergy, and Critical Care Medicine, New York, USA, ^3^Beth Israel Deaconess Medical Center, Department of Pulmonary, Critical Care & Sleep Medicine, Boston, USA

*Critical Care* 2023, **27(S1)**: P065

**Introduction:** Recruitment maneuvers (RM) are used in ARDS patients to improve oxygenation and lung mechanics [1]. 50% of patients do not respond due to unpredictable transpulmonary pressures (P_L_) suggesting that individualized care based on P_L_ may optimize recruitment and prevent overdistension [2].

**Methods:** This retrospective cohort study included ARDS adult patients, previously enrolled in the EPVent2 trial, receiving RM and with available waveforms recordings [3]. We estimated dose–response associations of end-recruitment end-inspiratory P_L_ and change in lung elastance after RM (ΔEL). Analysis was done through a generalized propensity score (GPS) with logistic link function model, multivariable logistic and linear regression models. Negative ΔEL represented successful recruitment, and positive ΔEL overdistension.

**Results:** 123 patients were included. 69 (65.1%) had a negative ΔEL. The means (± SD) of P_L_ were 14.0 cmH_2_O (± 4.0) and 15.1 cmH_2_O (± 4.9) in patients with negative and positive ΔEL, respectively. In the GPS model, a higher P_L_ was associated with higher probability of overdistension (*p* = 0.003) and predicted an inverse S-shaped dose–response curve, where the probability of successful recruitment is below 50% when P_L_ values were > 18.1 cmH2O (Fig. 1). In the adjusted logistic regression, higher values of P_L_ were associated with decreased probability of successful recruitment (OR_adj_ 0.73 per 1 cmH_2_O P_L_; 95% CI 0.62–0.86; *p* < 0.01). Volume of recruitment (V_RM_) was dependent on P_L_ (*p* < 0.01, *R*^*2*^ = 0.49) and was inversely dependent on ΔEL controlling for the baseline lung elastance (*p* < 0.01, *R*^*2*^ = 0.43).

**Conclusions:** Higher P_L_ results in more V_RM_, but also more overdistension. Evaluation of P_L_ should be considered while performing lung recruitment.


**References**
Hess DR et al. Respir Care. 2015;60:1688–1704.Baedorf Kassis E et al. Intensive Care Med. 2017;43:1162–1163.Beitler JR et al. JAMA. 2019;321:846–857.

**Figure Figaa:**
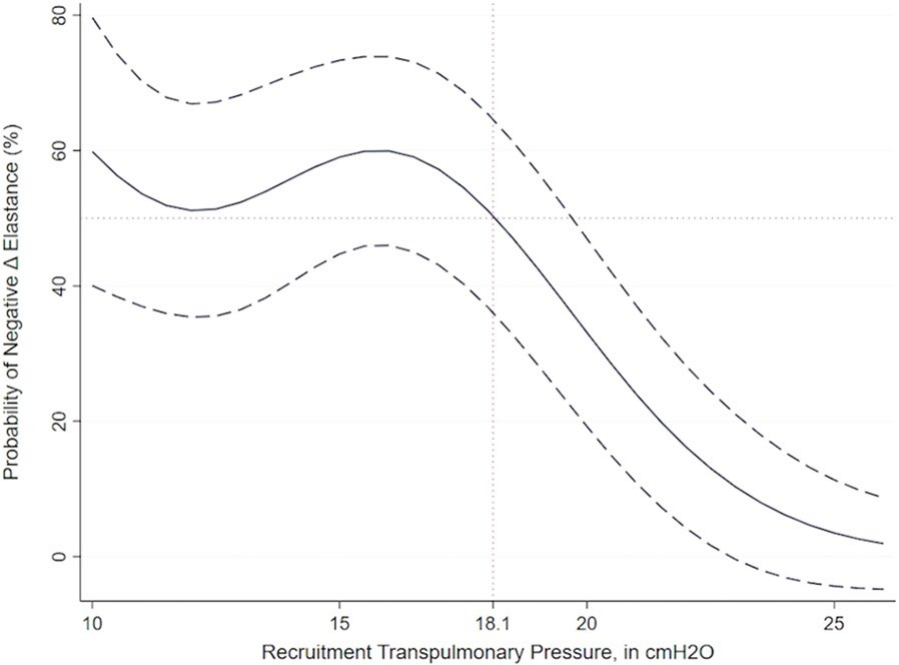
**Fig. 1 (abstract P065)**. Dose–response associations of end-recruitment end-inspiratory PL and change in lung elastance after RM (ΔEL), where positive ΔEL represents lung overdistension. Analysis was done through a generalized propensity score (GPS) with logistic link function model

## P066

### Are there any alternatives to the standard esophageal balloon catheter to monitor esophageal pressure in ICU patients?

#### A El Kik, H Eid, T Mehanna, M Riachy

##### Hôtel-Dieu de France – Saint Joseph University, Pulmonary and Critical Care Department, Beyrouth, Lebanon

*Critical Care* 2023, **27(S1)**: P066

**Introduction:** Some intensive care physicians practicing in underdeveloped countries are limited in the use of esophageal pressure due to the unavailability and/or to the high cost of the esophageal balloon catheters. The aim of this study is to identify medical devices with inflatable balloons that could be alternatives to traditional esophageal balloon catheters.

**Methods:** Four different balloons were studied: the esophageal (EB) and gastric balloons (GB) of a Blakemore tube (Rüsch©, 18 Ch), the balloon of a Swan-Ganz catheter (Edwards Lifesciences©) (SGB) and the balloon of a Foley catheter (Bardia©, 24 Ch) (FB). Each balloon was connected apart to an ICU ventilator (Engström Carestation©) and was studied in vitro. The pressure–volume curves was drawn for each balloon by measuring the pressure for every imposed volume on room atmosphere. A plateau pattern is searched for each balloon to avoid any additional pressure due to balloon wall stretching [1]. Then each balloon was submerged in a water column of a progressive depth of 10, 20 and 30 cm in order to create an external pressure of 10, 20 and 30 cmH2O respectively. The pressure–volume curves were again obtained under these conditions.

**Results:** Zero-plateau curve was only observed for the esophageal (EB) (volume ≤ 45 mL) and gastric (GB) (volume ≤ 10 mL) balloons of the Blakemore tube. When outside pressure of 10, 20 and 30 cm H2O were applied to EB and GB, the plateau was successfully achieved. The volume varied from 20 to 40 mL for the EB with a plateau at 9, 17 and 27 cmH2O respectively. The volume varied from 4 to 10 mL for the GB with a plateau at 7, 20, and 26 cmH2O respectively (Fig. 1).

**Conclusions:** The use of EB to monitor the esophageal pressure is limited by the need of high volumes due to its intrinsic elastance. The GB is a good alternative to standard catheter with an adapted volume that ranges between 4 to 10 mL.


**Reference**
Mojoli F et al. Crit Care 2016;20:98.

**Figure Figab:**
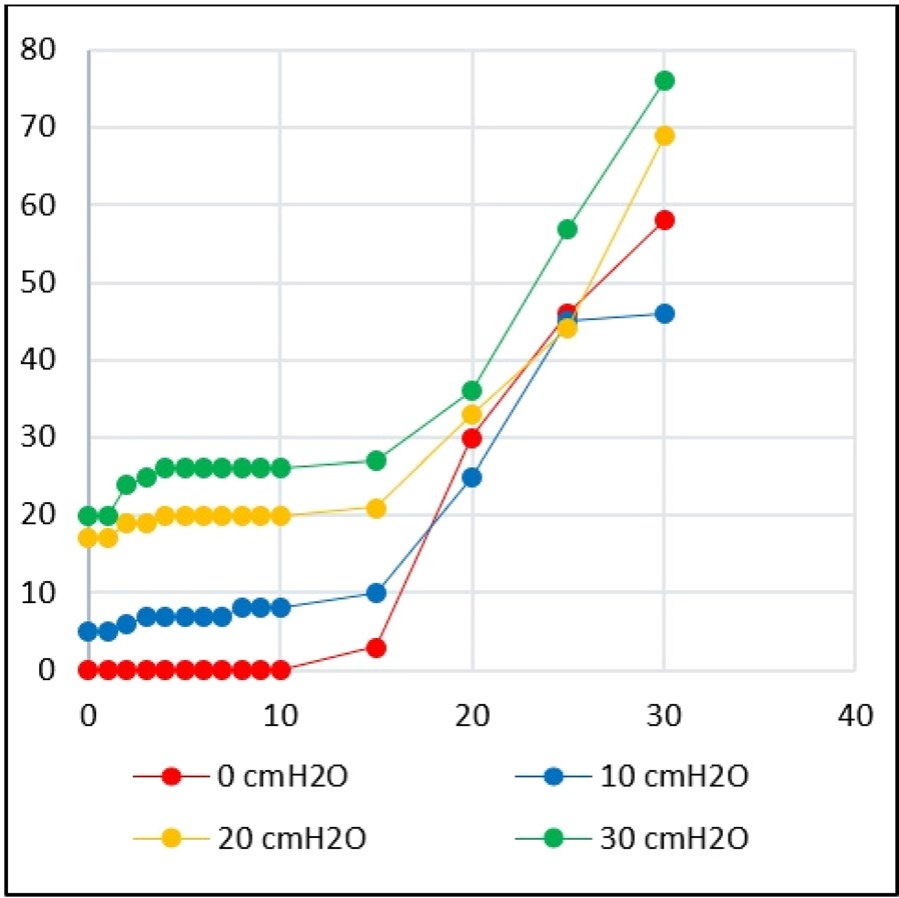
**Fig. 1 (abstract P066)**. Pressure–volume curves of the Blakemore tube’s gastric balloon (GB)

## P067

### Impact of the COVID-19 pandemic on acute pancreatitis in the Czech Republic: pilot data from the PANACOTA (pancreatitis acuta in COVID time analysis) study

#### M Harazim, R Kroupa

##### Department of Gastroenterology and Internal Medicine, University Hospital Brno, Czech Republic

*Critical Care* 2023, **27(S1)**: P067

**Introduction:** The COVID-19 pandemic had a significant impact on healthcare. Acute pancreatitis (AP) is a clinically serious disease that almost always requires hospitalisation and treatment that cannot be delayed or planned. International studies have highlighted the potential negative impact of COVID-19 infection on the onset and severity of the disease. The aim of this study is to analyse the impact of the COVID-19 pandemic on the epidemiology, treatment and prognosis of acute pancreatitis at the population level.

**Methods:** Comparison of parameters of hospitalizations for acute pancreatitis, care provided and treatment outcomes during the pandemic (2020 + 2021) with previous years (2010–2019) using data from the National Registry of Covered Health Services. Presentation of pilot data.

**Results:** Hospitalizations for AP increased slightly from 2010 to 2015 to about 7000/year with a relatively stationary number in 2016–2021. Hospitalizations for AP in each month of 2020 and 2021 did not correlate with the number of COVID-19 positive patients and showed a similar trend (with peaks in summer and at the end of the year) as in the years prior to the pandemic. Concurrently with AP, 2.3% and 3.7% of patients had COVID-19 infection in 2020 and 2021, respectively. AP mortality in the pre-pandemic and pandemic years was virtually the same at 3.9%. There was no difference in length of hospital or ICU stay. There was a downward trend in the number of ERCPs and surgical interventions performed.

**Conclusions:** According to pilot population data, the COVID-19 pandemic did not have an impact on the incidence or prognosis of acute pancreatitis in the Czech Republic. Changes in trends in interventions performed correlate with recent expert recommendations and are unlikely to be related to the reduction in care due to the pandemic.

## P068

### A nationwide cohort study of pregnant and postpartum patients with severe COVID-19 pneumonitis in ICU in Israel

#### E Fatnic

##### Hadassah Medical Organization and Faculty of Medicine, Hebrew University of Jerusalem, Jerusalem, Israel, Department of Anesthesiology, Critical Care and Pain Medicine, Jerusalem, Israel

*Critical Care* 2023, **27(S1)**: P068

**Introduction:** This study included pregnant patients with severe COVID to test the hypothesis that the impact of delivery on maternal outcome depends upon illness severity at the time of delivery; we hypothesized that patients not yet requiring IPPV would improve following delivery (due to improvement in respiratory mechanics), while patients already on IPPV, or close to requiring ventilation, would deteriorate (due to maternal cardiovascular intolerance to autotransfusion).

**Methods:** This multicenter, prospective/retrospective cohort study evaluated Israeli ICU admissions of pregnant women with COVID-19 pneumonitis from 1-Feb-2020 to 31-Jan-2022. We assessed maternal, neonatal outcomes and longitudinal maternal clinical data. The primary outcome was maternal outcome (no-IPPV, IPPV, ECMO, death). The primary longitudinal outcome was SOFA score, the secondary longitudinal outcome was the novel PORCH score (PEEP, Oxygenation, Respiratory-support, Chest-X-ray, Haemodynamic-support). Patients were classified into: “no-delivery”, “postpartum admission”, “delivery-critical” and “delivery-not-critical” groups.

**Results:** 84 patients in 13 ICUs were analysed; there were 34 “no-delivery”, 4 “postpartum”, 32 “delivery-critical”, 14 “delivery-not-critical” patients. “Delivery-critical” and “postpartum” had worse outcomes with, 26/32(81%) and 4/4(100%) requiring IPPV; 12/32(38%) and 3/4(75%) requiring ECMO; 1/32(3%) and 2/4(50%) dying. “Delivery-not-critical” and “no-delivery” had far better outcomes with, respectively, 6/34(18%) and 2/14(14%) requiring mechanical ventilation; no patients required ECMO or died. SpO2, S/F ratio, P/F ratio in “Delivery-critical” deteriorated on the day of delivery, continued to deteriorate, and took longer to recover; “delivery-not-critical” improved rapidly following delivery. The day of delivery was a highly significant covariate for PORCH (*p* < 0.0001), not SOFA (*p* = 0.09).

**Conclusions:** Interventional delivery should be considered for maternal indications before patients deteriorate and require IPPV.

## P069

### Sars-CoV-2 in critical immunocompromised patients. Evolution and prognosis

#### A Rivas Bilbao, C Hoya Gonzalez, D Iglesias Posadilla, J Higuera Lucas, M Corera Cia, M Vazquez, E Iñigo Morras, S Kadi Ayad, M Rubio Gaztelu, A Jimeno Rodriguez

##### Hospital Universitario de Cruces, Medicina Intensiva, Baracaldo, Spain

*Critical Care* 2023, **27(S1)**: P069

**Introduction:** In this study, we share the results of immunosuppressed patients who suffered from acute respiratory distress syndrome (ARDS) secondary to COVID-19 pneumonia managed in our ICU.

**Methods:** We tracked all patients admitted to ICU of a Tertiary Hospital diagnosed with severe SARS-COV2 pneumonia from March 1, 2020 to January 31, 2022. The definition of Immunocompromised patient is based on history of transplantation, active neoplasia, autoimmune diseases or HIV. Collected data includes: sex, age, type of immunosuppression, vaccination, mechanical ventilation, ECMO VV, incidence of superinfections and mortality.

**Results:** From a cohort of 425 patients, 55 met the inclusion criteria. 33% were women and 67% male. The average age was 58 years for women and 62 years for men. Out of these patients, 27% had solid organ transplants. 40% suffered from neoplasic disease. 27% had autoimmune diseases and were under treatment with immunosuppressants. 3 had HIV. Only the 29% had received at least 1 dose of COVID 19 vaccine. 80% required orotracheal intubation. 3.64% (2) required Veno-Venous ECMO. 61% presented bacterial superinfection, with the most frequent germs being Pseudomonas aeruginosa and Enterococcus. 36% had viral superinfection, being cytomegalovirus the most frequent one. 32% had fungal superinfection, mainly by Aspergillus fumigatus. 27% did not suffer any superinfection. 40% of the total sample died. After logistic regression, in our model (AUC 83,4% (Se 57.1%, Sp 87.9%), we identified need of intubation as independent variable of mortality (OR 27,06 IC95% 1.76–415.55, *p* = 0.018).

**Conclusions:** Immunocompromised patients with ARDS secondary to COVID-19 pneumonia present high mortality, with statistically significant difference when mechanical ventilation is needed. The most frequently isolated germs causing superinfection in this group of patients are bacterias. We believe that this group of patients require special care in our ICU units and an in-depth analysis and study to optimize their prognosis.

## P070

### Geodemographic factors associated with COVID-19 mortality in indigenous population in Colombia

#### J Cárdenas^1^, J Leon^2^

##### ^1^Hospital Universitario San Vicente Fundación, Critical Medicine and Intensive Care/Medical Intensive Care Unit, Medellin, Colombia, ^2^Centro de Tratamiento e Investigación sobre Cáncer Luis Carlos Sarmiento Angulo (CTIC), Hospitalization Unit/Internal Medicine, Bogotá DC, Colombia

*Critical Care* 2023, **27(S1)**: P070

**Introduction:** Since 2019 there have been over 80,000 confirmed COVID-19 cases in the indigenous ethnic groups in Colombia. Age, sex, and region of residency might be factors that contribute to COVID-19 mortality in these ethnic populations. The objective of this research is to describe COVID-19 whether these are associated with COVID-19 mortality in this population. According to the 2018 national population census, there are 1.905.617 persons who identified themselves as indigenous, 50.1% of whom are women, younger than 64 years old and live in rural areas [1].

**Methods:** This is a retrospective cohort study, using data collected through the national retrospective cohort of confirmed COVID-19 cases. The study population were the confirmed COVID-19 cases in the indigenous population in Colombia since 03/2019 until 10/2022. A Cox Regression Model was used to estimate the HR by age, sex, and geographical location.

**Results:** There were 83,436 confirmed COVID-19 cases in the indigenous population in this period. The association between age and COVID-19 mortality shows that older individuals and males have higher mortality risk. The geographical location was explored as a risk factor for COVID-19 mortality. Results are shown in Table 1. Most of the regions have HR very close to 1.0, and none reached statistical significance.

**Conclusions:** Age and sex remain significant factors associated with COVID-19 mortality, as they are in other population studies [2]. The region of residency is not a factor significantly associated with COVID-19 mortality in this study, as this characteristic does not seem to reflect socioeconomic inequalities that have been proven to impact COVID-19 mortality in Colombia [2].


**References**
Ethnic groups population census, DANE. Available at: https://www.dane.gov.co/index.php/estadisticas-por-tema/demografia-y-poblacion/grupos-etnicos/informacion-tecnica.Cifuentes MP et al. J Epidemiol Community Health. 2021, 75: 60–615.

**Table Tabq:** **Table 1 (abstract P070)**. Geographic regions and risk of death due to COVID-19

Region of residency	HR (95% CI)	*p* value
Andine region	1.05	0.413
Amazonic region	Ref	Ref
Caribbean region	1.04	0.495
Orinoquia region	1.12	0.083
Pacific region	0.94	0.697

## P071

### Impact of COVID-19 pandemic in solid organ transplant in Bogota, Colombia

#### J Leon^1^, J Cardenas^2^

##### ^1^Centro de Tratamiento e Investigación sobre Cáncer Luis Carlos Sarmiento Angulo (CTIC), Hospitalization Unit/Internal Medicine, Bogota DC, Colombia, ^2^Fundación Cardioinfantil, Critical Medicine and Cardiovascular Intensive Care/Cardiovascular Intensive Care Unit, Bogotá DC, Colombia

*Critical Care* 2023, **27(S1)**: P071

**Introduction:** During the COVID-19 pandemic the number of solid organ transplants (SOT) lessened globally due probably to decreased donation and detour of resources to other prioritized activities [1, 2]. The aim of this paper is to analyze the SOT behavior during the COVID-19 pandemic in this city.

**Methods:** An exploratory analysis was performed on the data of the Statistics on donation and transplant of organs and tissues in Bogota from 2018 until the third trimester of 2022 [3].

**Results:** 416 SOT from 365 organ donors were performed in Bogota during 2018. The first COVID-19 case in Colombia was documented in March 2020. During the following two years there was a decline in the number of performed SOT and total organ donors, as shown in Table 1. During 2020 there was a drastic reduction on the total SOT, compared with 2018. During 2021 there was a slight recovery in the total SOT, and in the first three trimesters of 2022 there was a drastic increase in the total SOT with 380 procedures performed and 380 organ donors. There was a steeper reduction in the number of heart and lung transplants during 2020 and 2021. The SOT waiting lists remained stable from 2018 to 2022, with 1804 patients in 2021, up to 1950 patients in 2022.

**Conclusions:** The reduction in SOT might be due to COVID-19 in donors, reduced incidence of brain death and lessened capability to preserve viable organ donors. The SOT waiting list didn’t increase possibly caused by high mortality due to end-stage organ failure. The SOT increase during 2021 coincides with a decrease in COVID-19 lethality in Bogota. This decline in SOT was observed globally during 2020, whilst the rapid recovery in SOT and availability of organ donors during 2022 is a phenomenon that has not been described yet to our knowledge.


**References**
Dominguez-Gil B et al. Am J Transplant 2020;20:2593–2598.Kute VB et al. Transplant Proc. 2022;54:1412–1416.Statistics on donation and transplant of organs and tissues in Bogota. Health Secretary.

**Table Tabr:** **Table 1 (abstract P071)**. SOT behavior in Bogota, 2018–2021

	2018	2019	2020	2021
Total SOT	416	487	277	351
Total organ donors	365	316	208	264
Kidney transplants	273	310	171	217
Liver transplants	112	120	91	101
Heart transplants	13	28	10	23
Lung transplants	13	22	4	8
Waiting list	1861	1854	1855	1804

## P072

### Regional effects on efficacy and safety of vilobelimab in mechanically ventilated patients with severe COVID-19

#### EHT Lim^1^, D Van de Beek^2^, S De Bruin^1^, S Rückinger^3^, C Thielert^4^, R Guo^5^, BP Burnett^4^, MC Brouwer^2^, NC Riedemann^4^, APJ Vlaar^1^

##### ^1^Amsterdam UMC, Intensive Care Medicine, Amsterdam, Netherlands, ^2^Amsterdam UMC, Neurology, Amsterdam, Netherlands, ^3^Metronomia Clinical Research GmbH, Munich, Germany, ^4^InflaRx GmbH, Jena, Germany, ^5^InflaRx Pharmaceuticals Inc., Ann Arbor, USA

*Critical Care* 2023, **27(S1)**: P072

**Introduction:** Poor outcomes in COVID-19 patients (pt) are associated with C5a-C5aR axis activation. A C5a-specific monoclonal antibody, vilobelimab (VILO), improves outcomes in critically ill COVID-19 pt in a Phase 3 randomized, double-blind, placebo (PLC)-controlled study [1].

**Methods:** COVID-19 pt within 48 h of intubation were randomly assigned to receive 6, 800 mg infusions of VILO or PLC at a 1:1 ratio on top of standard of care. Predefined subgroup analyses by region and country were performed.

**Results:** Forty-six (46) hospitals on 4 continents randomized 369 pt: VILO (n = 178), PLC (n = 191). VILO significantly reduced 28- (HR 0.67; 95% CI 0.48–0.96; *p* = 0.027) and 60-Day mortality (HR 0.67; 95% CI 0.48–0.93, *p* = 0.0163) using a predefined, unstratified per protocol analysis. Mortality rates at 28- and 60-days and VILO treatment effects, however, differed substantially between regions: Western Europe HR for 60-day mortality 0.59 [0.37–0.95], South Africa plus Russian Federation HR 0.62 [0.28–1.38] and South America HR 0.80 [0.46–1.39] (Fig. 1). The weak signal in South America is predominately driven by Brazil (n = 74), which showed a significant age imbalance with a median 9-years younger PLC group (44.5-years-old vs 53.5-years-old) with low 60-day mortality of ~ 32.5% in the PLC group versus ~ 43.3% in Western Europe. Adjusting for age group categories (≤ 30, 31–40, 41–50, 51–60, > 60; Cox regression) for 60-day mortality changed the HR in Brazil (0.96 [0.44–2.10] for continuous age-adjustment) to values near the estimate for the entire study population (HR 0.77 [0.35–1.69] for age in categories), suggesting a “by chance” imbalance and not a statistically evident weaker effect in Brazil.

**Conclusions:** Regional efficacy differences between the rest of the world and South America were driven by age imbalances between treatment groups, which do not diminish the robust efficacy signal for VILO in severe COVID-19.


**Reference**
Vlaar APJ et al. Lancet Respir Med. 2022;10:1137–1146.

**Figure Figac:**
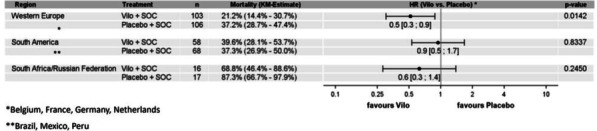
**Fig. 1 (abstract P072)**. Forest plot for 60-days all-cause mortality by region (Western Europe, South America, South Africa/Russian Federation)—FAS. *Hazard ratio from the Cox proportional hazards regression model with outcome 60-day all-cause mortality as a censored time-to-event variable and explanatory variables treatment arm and age. Harzard ratios are only displayed for subgroups with at least 10 patients and for which at least one event per treatment group has been observed

## P073

### High-dose corticosteroids and biomarker profiles in COVID-19 acute respiratory distress syndrome at the intensive care unit: a retrospective cohort study

#### K Daenen^1^, K Tong-Minh^2^, J Huijben^1^, V Dalm^3^, E Van Gorp^2^, H Endeman^1^

##### ^1^Erasmus MC, Intensive Care, Rotterdam, Netherlands, ^2^Erasmus MC, Viroscience, Rotterdam, Netherlands, ^3^ Erasmus MC, Internal Medicine, Rotterdam, Netherlands

*Critical Care* 2023, **27(S1)**: P073

**Introduction:** COVID-19 patients with non-resolving ARDS may benefit from treatment with high-dose steroids (HDS), because of a presumed persistent systemic and alveolar hyperinflammation. At ICU admission, it is unknown which patients require HDS. Obtaining insights in their inflammatory state by using biomarkers and determining their association with the effect of HDS could support decision-making. The goal of this study is to compare the patient characteristics and biomarker profiles at ICU admission of the patients that received HDS to the patients who did not.

**Methods:** This was a retrospective cohort study including COVID-19 patients admitted to the ICU of the Erasmus MC between 2020 and 2022. The primary intervention was treatment with HDS, defined as 1000 mg methylprednisolone or > 40 mg prednisolone for three consecutive days. We compared demographics, comorbidities, biomarkers and mortality between patients treated with HDS and without. Logistic regression multivariate analyses was used to analyze which biomarkers were associated with initiation of HDS.

**Results:** We included 151 patients, of which 48 were treated with HDS at a median of 6 days after ICU admission (Table 1). There were no significant differences in demographics and comorbidities. Patients treated with HDS had a significantly longer ICU length of stay (*p* < 0.001) and higher hospital mortality rate (*p* < 0.001). LDH (*p* = 0.02) and ferritin (*p* = 0.05) levels on admission were significantly higher in patients treated with HDS, whereas their CRP was significantly lower (*p* = 0.02). In multivariate regression analysis, these were not independently associated with the initiation of HDS.

**Conclusions:** At ICU admission, demographics and comorbidities did not differ between patients treated with HDS and without. There were no factors associated with the initiation of HDS in COVID-19 patients in the ICU. Further studies on the association between the inflammatory state, mortality, and the effect of HDS are required to understand which patients may benefit from HDS.

**Table Tabt:** **Table 1 (abstract P073)**. Patient characteristics and outcomes

	Non high-dose steroids (N = 113)	High-dose steroids (N = 48)	Overall (N = 151)	*p* value
Age in years (median (IQR))	65.0 [15.0]	62.0 [11.0]	64.0 [14.3]	0.616
Comorbidities (total (%))	53 (46.9%)	25 (52.1%)	78(48.4%)	0.821
LDH in U/L (median (IQR))	401 [178]	448 [190]	420 [182]	0.0168
Ferritine in U/L (median (IQR))	1090 [1260]	1550 [1720]	1260 [1630]	0.0545
CRP in mg/L (median (IQR))	169 [154]	97.3 [169]	159 [178]	0.0232
ICU length of stay in days (median (IQR))	15.0 [16.0]	27.0 [18.0]	17.0 [19.0]	< 0.001
Mortality (total (%))	25 (22.1%)	25 (52.1%)	50 (31.1%)	< 0.001

*IQR* Interquartile range; *ICU* intensive care unit

## P074

### Antithrombotic strategies in COVID-19 patients: a systematic review and Bayesian network meta-analysis

#### HB Chen^1^, P Chen^2^, C Gao^3^, JF Xie^3^, HB Qiu^3^

##### ^1^Jiangsu Provincial Key Laboratory of Critical Care Medicine, Department of Critical Care Medicine, Zhongda Hospital, School of Medicine, Southeast University, Nanjing, China, ^2^The Hospital of the Second Mobile Corps, Chinese People’s Armed Police Forces, Fuzhou, China, ^3^Jiangsu Provincial Key Laboratory of Critical Care Medicine, Nanjing, China

*Critical Care* 2023, **27(S1)**: P074

**Introduction:** COVID-19 is a public health emergency of international concern. Clinicians are likely to adopt various antithrombotic strategies to prevent embolic events, but the optimal antithrombotic strategy remains uncertain. We performed a Bayesian network meta-analysis to evaluate various antithrombotic strategies comprehensively.

**Methods:** We systematically searched PubMed, Cochrane Library, Web of Science, EMBASE and Clinical trials. gov to screen trials comparing different antithrombotic strategies. The primary outcome is 28-day mortality, and the secondary outcomes include major thrombotic event, major bleeding and in-hospital mortality, etc. We assessed the risk of bias using the Cochrane Collaboration’s tool and the quality of evidence according to the Grading of Recommendations Assessment, Development and Evaluation (GRADE) approach. We successively performed traditional pairwise and Bayesian network meta-analysis using R v4.2.1 software.

**Results:** Twenty-six eligible randomized controlled trials were included, giving a total of 35 paired comparisons with 32,041 patients randomized to 7 antithrombotic strategies. In comparison to standard of care (SoC) strategy, therapeutic anticoagulation (TA) (RR 0.36, 95% CrI 0.13–0.86) and prophylactic anticoagulation (PA) (RR 0.35, 95% CrI 0.12–0.85) strategy significantly reduced the mortality of COVID-19 patients (Fig. 1). The antiplatelet (AP) strategy was associated with high risk of major bleeding when compared with SoC strategy (RR 2.5, 95% CrI 1.1–8.9), and the TA (RR 0.43, 95% CrI 0.17–0.98), PA (RR 0.27, 95% CrI 0.10–0.63) and PA with Fibrinolytic agents (FA) strategy (RR 0.12, 95% CrI 0.01–0.81) was associated with low risk of major thrombotic event.

**Conclusions:** This network meta-analysis indicates that the TA and PA strategies probably reduce mortality and confer other important benefits in COVID-19 patients. These findings provide guidance on how to choose optimal antithrombotic strategies for COVID-19 patients.

**Figure Figad:**
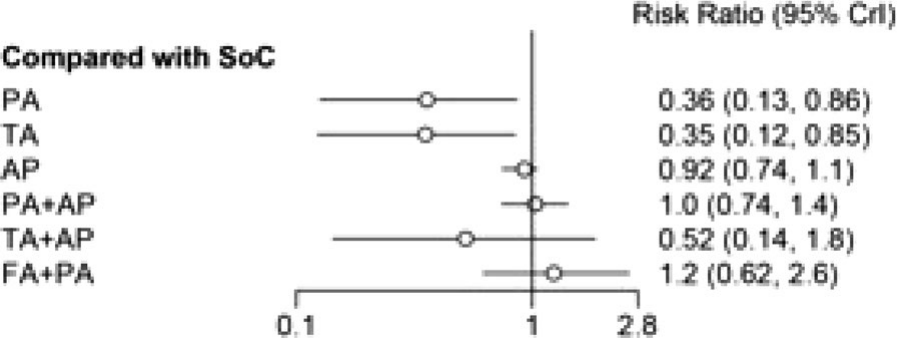
**Fig. 1 (abstract P074)**. The forest plot of network effect sizes of various antithrombotic strategies compared with standard of care. CrI = credible interval, SoC = standard of care, PA = prophylactic anticoagulation, TA = therapeutic anticoagulation, A*P* = antiplatelet, FA = fibrinolytic agents

## P075

### A new score to predict severe respiratory failure and guide anakinra treatment in COVID-19 pneumonia: analysis of the randomized SAVE-MORE trial

#### E Kyriazopoulou^1^, G Poulakou^2^, H Milionis^3^, S Metallidis^4^, M Fantoni^5^, A Angheben^6^, P Panagopoulos^7^, G Dalekos^8^, M Netea^9^, E Giamarellos-Bourboulis^2^

##### ^1^National and Kapodistrian University of Athens, 4th Department of Internal Medicine, Athens, Greece, ^2^National and Kapodistrian University of Athens, Athens, Greece, ^3^University of Ioannina, Ioannina, Greece, ^4^Aristotle University of Thessaloniki, Thessaloniki, Greece, ^5^Fondazione Policlinico Gemelli IRCCS, Rome, Italy, ^6^Tropical Diseases and Microbiology, IRCSS Sacro Cuore Hospital, Negrar, Italy, ^7^University of Thrace, Alexandroupoli, Greece, ^8^University of Thessaly, Larissa, Greece, ^9^Radboud University, Nijmegen, Netherlands

*Critical Care* 2023, **27(S1)**: P075

**Introduction:** Anakinra treatment is approved for the treatment of COVID-19 pneumonia in hospitalized adults in need of oxygen and at risk for progression into severe respiratory failure (SRF) defined as circulating concentrations of the biomarker suPAR (soluble urokinase plasminogen activator receptor) ≥ 6 ng/mL by the EMA and has been authorized for emergency use by FDA under an EUA [1]. This is based on the results of the randomized SAVE-MORE trial where suPAR ≥ 6 ng/mL was used to select patients at risk for SRF [2]. The suPAR test is not commercially available in the USA and an alternative method of patient selection was needed.

**Methods:** In collaboration with the US FDA, an alternative method to select patients most likely to have suPAR ≥ 6 ng/mL based on commonly measured patient characteristics was developed. Patients with at least 3 of the following criteria are considered likely to have suPAR ≥ 6 ng/ml: age ≥ 75 years, severe pneumonia by WHO criteria, current/previous smoking status, Sequential Organ Failure Assessment score ≥ 3, neutrophil-to-lymphocyte ratio ≥ 7, hemoglobin ≤ 10.5 g/dl, history of ischemic stroke, blood urea ≥ 50 mg/dl and/or history of renal disease.

**Results:** The positive predictive value of this new score was 95.4% in SAVE-MORE population. However, a lower sensitivity meant a small proportion of patients with suPAR ≥ 6 ng/ml will not be identified by the new score. The adjusted hazard ratio for survival at 60 days for patients meeting this score and who receive anakinra is 0.45 (Fig. 1).

**Conclusions:** The developed score predicts accurately patients with suPAR levels ≥ 6 ng/mL and may be used as an alternative to guide anakinra treatment in patients with COVID-19 pneumonia. Based on these subgroup results, patients in SAVE-MORE who met the new score appeared to show beneficial efficacy results with treatment of anakinra consistent with the overall studied population.


**References**
Fact sheet for healthcare providers: emergency use authorization for kineret: https://www.fda.gov/media/163075/download.Kyriazopoulou E et al. Nat Med 2021;27:1752.

**Figure Figae:**
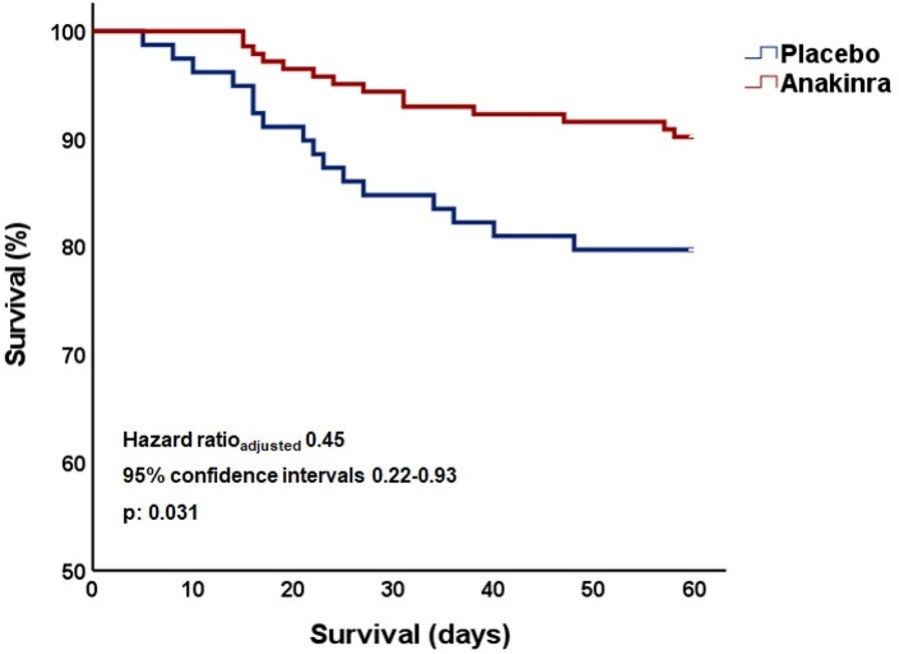
**Fig. 1 (abstract P075)**. Cox survival analysis by Day 60 of patients enrolled in the SAVE-MORE trial and meeting the new score

## P076

### Outcomes of a community-based bamlanivimab infusion program for COVID-19

#### E Michelson, S Crawford, S Monks

##### Paul Foster School of Medicine, Texas Tech University HSC, El Paso, Emergency Medicine, El Paso, USA

*Critical Care* 2023, **27(S1)**: P076

**Introduction:** The U.S. FDA issued an Emergency Use Authorization in 11/2020 for Bamlanivimab (Bam), a monoclonal antibody infusion, in high-risk outpatients with COVID-19. We examined the effectiveness of Bam given in a coordinated community wide initiative in reducing ED visits, hospitalizations, and deaths during the first 30 days post-treatment.

**Methods:** Bam treated adults at a community-based (CB) infusion center in the SW USA were identified in a regional EMR database covering all local hospitals. Individual patients were de-identified but tracked for 30 days for ED visits, hospital admissions, ICU admissions, and deaths. Patient demographics including age, sex and ethnicity were recorded. Our local IRB approved the project as exempt.

**Results:** A total of 2242 patients received Bam between 11/2020 and 3/2021, while the Wuhan variant was predominant. The mean age was 52, 54% were female and 64% identified as Hispanic or Latino. During the 30 days post infusion 13% of patients visited the ED, but only 2% of the total patients were admitted, 0.6% were admitted to the ICU and 14 died (0.6%). For comparison, a historical placebo control group of COVID patients in Chen et al. [1] is shown in Table 1. Hospitalizations among patients over 60 occurred at the same rate as the total cohort versus more than double the rate in older patients in the control group.

**Conclusions:** A CB Bam infusion program reduced ED visits, and hospitalizations, particularly among older patients, with a smaller reduction in ICU admissions and death. Our population is older and including a higher percentage of Hispanic patients who tend to have worse outcomes from COVID-19 [2]. While Bam has now been replaced by newer monoclonal antibodies, our results support the potential community benefit of coordinated monoclonal antibody treatment of patients with COVID-19, with a need for more research to identify patient subgroups who may receive the greatest benefit.


**References**
Chen P et al. N Engl J Med 2021;384:229–237De Ramos P et al. Am J Public Health 2022;112:1034–1044

**Table Tabv:** **Table 1 (abstract P076)**. Comparison of historical control to patients treated with Bam

Subjects	Historical control 143 (Chen et al. [1])	CB Bam Infusion Center 2242
Average age	44	52
Hispanic	44%	64%
Hospitalized	6.3%	2%
Hospitalized (age > 65)	14.6%	2% (age > 60)
Hospitalized ICU	0.7%	0.6%
Fatality	0.7%	0.6%

## P077

### Population pharmacokinetics of dexamethasone in critically ill COVID-19 patients: inflammation might play a role

#### L Li^1^, S Sassen^1^, N Hunfeld^2^, T Smeets^1^, T Ewoldt^2^, B Koch^1^, H Endeman^2^

##### ^1^Erasmus Medical Center, Pharmacy, Rotterdam, Netherlands, ^2^Erasmus Medical Center, Intensive Care, Rotterdam, Netherlands

*Critical Care* 2023, **27(S1)**: P077

**Introduction:** One of the common causes of COVID-19 related death is acute respiratory distress syndrome (C-ARDS). Dexamethasone is the cornerstone in the therapy of C-ARDS and reduces mortality probably by suppressing inflammatory levels in ICU patients. Its anti-inflammatory effects may be concentration-related. However, no pharmacokinetic studies of dexamethasone have been conducted in ICU patients. Therefore, we designed a population pharmacokinetic study to gain a deeper understanding of the pharmacokinetics of dexamethasone in critically ill patients in order to identify relevant covariates that can be used to personalize dosing regimens and improve clinical outcomes.

**Methods:** This was a retrospective pilot study at the ICU of the Erasmus Medical Center. Blood samples were collected in adults at the ICU with COVID who were treated with fixed dose intravenous dexamethasone (6 mg/day). The data were analyzed using Nonlinear Mixed Effects Modelling (NONMEM) software for population pharmacokinetic analysis and clinically relevant covariates were selected and evaluated.

**Results:** A total of 51 dexamethasone samples were measured in 18 patients. A two-compartment model with first-order kinetics best fitted the data. The mean population estimates for drug clearance and inter-compartment clearance were 2.85 L/h (IIV 62.9%) and 2.11 L/h, respectively, and central and peripheral volumes of distribution were 15.4 L and 12.3 L, respectively. The covariate analysis showed a significant correlation between dexamethasone clearance and CRP. Dexamethasone clearance decreased significantly with increasing CRP in the range of 0–50 mg/L and a correlation was observed with CRP up to 100 mg/L.

**Conclusions:** The dexamethasone PK parameters of ICU COVID patients were quite different from those come from healthy populations. Inflammation might play an important role in dexamethasone clearance and the dosing should be more individualized in order to achieve best therapeutic effect in ICU patients.

## P078

### Pharmacokinetic/pharmacodynamic analysis of vilobelimab and anaphylatoxin c5a as well as antidrug antibodies in Panamo: a phase 3 study in critically ill, mechanically ventilated COVID-19 patients

#### EHT Lim^1^, APJ Vlaar^1^, S De Bruin^1^, S Rückinger^2^, C Thielert^3^, R Guo^4^, BP Burnett^3^, MC Brouwer^5^, NC Riedemann^3^, D Van de Beek^5^

##### ^1^Amsterdam UMC, Intensive Care Medicine, Amsterdam, Netherlands, ^2^Metronomia Clinical Research GmbH, Munich, Germany, ^3^InflaRx GmbH, Jena, Germany, ^4^InflaRx Pharmaceuticals Inc., Ann Arbor, USA, ^5^Amsterdam UMC, Neurology, Amsterdam, Netherlands

*Critical Care* 2023, **27(S1)**: P078

**Introduction:** C5a-C5aR axis activation is associated with increased mortality in severe COVID-19. Vilobelimab (VILO), a C5a-specific monoclonal antibody, improved mortality in severe COVID-19 patients (pts) in a Phase 3 multicenter, randomized, double-blind, placebo (PLC)-controlled study [1]. A pharmacokinetic/pharmacodynamic (PK/PD) analysis was undertaken to assess VILO and C5a as well as antidrug antibodies (ADA) levels in the study.

**Methods:** Forty-six (46) hospitals on four continents randomized 369 COVID-19 pts (VILO [n = 178], PLC [n = 191]) within 48 h of being mechanically ventilated to receive 6, 800 mg infusions of VILO or PLC at a 1:1 ratio on top of standard of care. Blood samples were taken at screening, Day 8 and at hospital discharge for VILO and C5a and at screening and hospital discharge for ADA. Enzyme‐linked immunosorbent assays were used to analyze levels.

**Results:** Screening blood samples for VILO and C5a were available for VILO (n = 93) and PLC (n = 99) from sites in Western Europe. On Day 8 after 3 infusions, mean VILO trough concentrations were 21799.3–302972.1 ng/mL (geometric mean 137881.3 ng/mL) (Fig. 1). At screening, C5a was highly elevated and comparable between groups: VILO median 118.3 ng/mL, mean 130.3 ng/mL, PLC median 104.6 ng/mL, mean 123.2 ng/mL. By Day 8, C5a levels were reduced by 84.6% in the VILO group (median 14.5 ng/mL [mean 16.8 ng/mL], *p* < 0.001) versus a 19.6% increase in the PLC group (median, 119.2 ng/mL, mean 129.8 ng/mL). Beyond Day 8, though PD sampling was sparse, C5a levels remained elevated for PLC whereas C5a slowly rose but did not reach screening levels for VILO. Treatment-induced ADA were observed in 1 pt in the VILO group (Day 40 discharge) and 1 pt in the PLC group (Day 25 discharge), both appeared independent of treatment.

**Conclusions:** The PK/PD analysis shows that VILO efficiently inhibits C5a in pts with severe COVID-19 resulting in a robust clinical effect on mortality reduction without inducing ADA.


**Reference**
Vlaar APJ et al. Lancet Respir Med. 2022;10:1137–1146.

**Figure Figaf:**
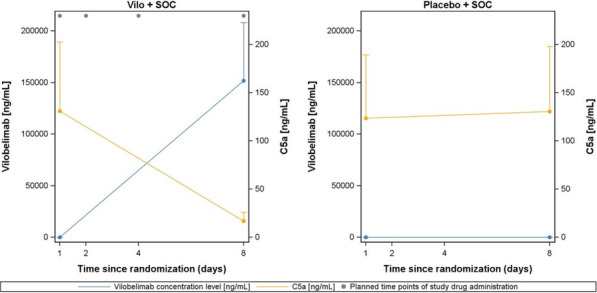
**Fig. 1 (abstract P078)**. Vilobelimab [ng/mL] and C5a [ng/mL] at screening and Day 8—safety analysis set

## P079

### Lung cavitation as a rare complication of severe COVID-19: report of 3 cases

#### E Ntikoudi, E Bourgani, S Giannakaki, A Aiginitou, M Karagianni, C Merkouri, M Poulou, E Kourtelesi, M Daganou, A Flevari

##### Sotiria Thoracic Diseases Hospital, Polyvalent ICU Department, Athens, Greece

*Critical Care* 2023, **27(S1)**: P079

**Introduction:** Lung cavitation is a rare radiological finding of COVID-19 pneumonia associated with unfavorable outcome. Its pathogenesis is unclear and it is characterized by diffuse alveolar damage, intra-alveolar hemorrhage and necrosis of parenchymal cells.

**Methods:** We retrospectively reviewed the radiological findings of COVID-19 patients admitted to our ICU during the pandemic in order to identify the development of lung cavitary lesions.

**Results:** From 11/2020 until 10/2022 1000 patients were admitted to our COVID-19 ICU (92% on invasive mechanical ventilation). According to our data there were three cases of lung cavity formation. The first case was a 78 years male with history of hypertension. Chest CT (Day26) showed a 11.6 cm cavity in the right middle lobe (Fig. 1). The second case was a 52 year old female with history of diabetes mellitus, obesity, hypertension and rheumatoid arthritis. Follow up chest CT (D29) revealed progressive development of multiple bilateral cavitary lesions. The third case was a 61 year old male with no medical history, who developed (D17) multiple cavitary lesions in both lower lobes, concomitant with left-sided pulmonary embolism. The presence of other well defined etiologies of cavitary lesions such as mycobacterial and fungal infections as well as neoplasmatic or autoimmune diseases had been widely excluded. However, since pulmonary cavitation is a late complication of severe COVID disease, we cannot overlook the fact that all patients suffered from superinfections by XDR Acinetobacter baumanii and/or Klebsiella pneumonia, as most of our patients with prolonged length of stay. Moreover, two of the three patients developed pneumothorax. All patients finally died.

**Conclusions:** Although bacterial co-infection does not allow absolute association between cavitary formation and coronavirus disease, it seems that destructive triggers, such as bacteria or mechanical ventilation, may aggravate COVID underlying lung lesions leading to cavitation.

**Figure Figag:**
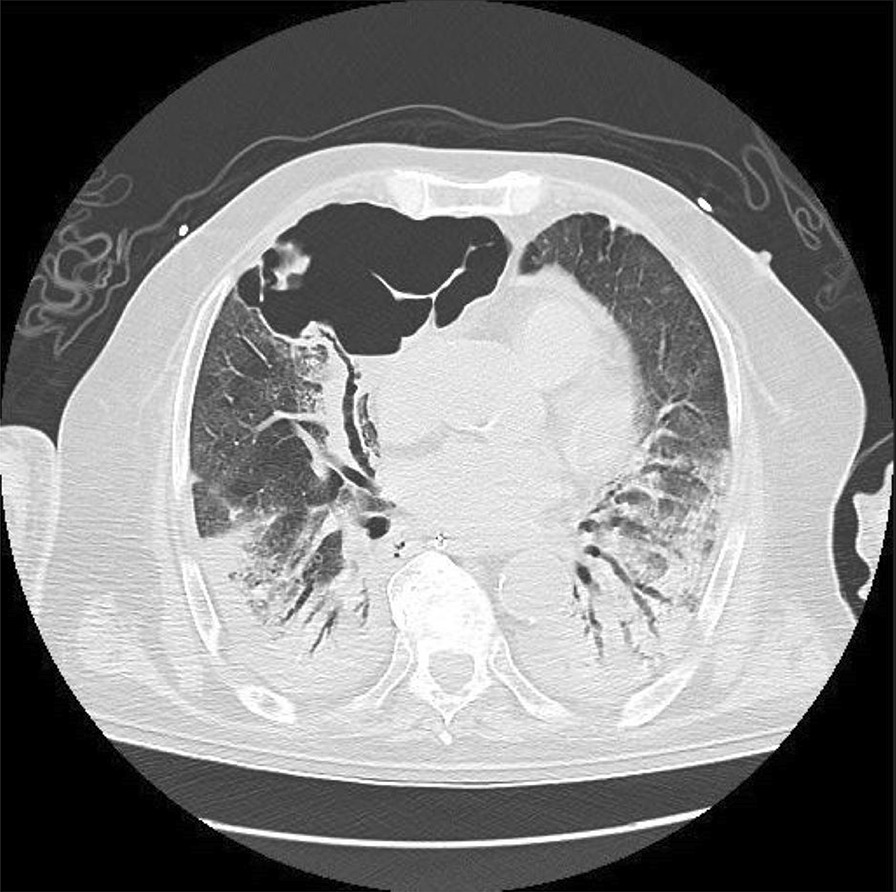
**Fig. 1 (abstract P079)**. CT finding of first case (78 year male). Lung cavity formation during COVID-19 pneumonia

## P080

### Lung inhomogeneities in COVID-19 related acute respiratory distress syndrome

#### A Santini^1^, A Protti^1^, F Pennati^2^, C Mercalli^1^, G Picardo^1^, L Pugliese^1^, N Martinetti^1^, C Chiurazzi^3^, A Aliverti^2^, M Cecconi^1^

##### ^1^Humanitas University, Department of Biomedical Sciences, Pieve Emanuele (MI), Italy, ^2^Politecnico di Milano, Dipartimento di Elettronica, Informazione e Bioingegneria, Milan, Italy, ^3^IRCCS Humanitas Research Hospital, Department of Anesthesia and Intensive Care Units, Rozzano (MI), Italy

*Critical Care* 2023, **27(S1)**: P080

**Introduction:** In acute respiratory distress syndrome (ARDS) inhomogeneities in lung aeration can act as local multipliers of pressure during inspiration (“stress risers”), increasing the risk of lung damage even in presence of airway pressures considered “safe” [1]. In this study we aimed to describe lung inhomogeneities in COVID-19 related ARDS (C-ARDS) and to relate these to disease severity and lung morphology.

**Methods:** We enrolled patients with C-ARDS within 3 days from mechanical ventilation start, deeply sedated and paralyzed. Lung CT scan was obtained at PEEP of 5 cmH_2_O to measure lung weight compartments (non-, poorly-, well- and over-aerated). Lung inhomogeneities were computed as the gas/tissue ratio of each voxel compared to the neighboring voxels. We considered values > 1.61 as pathologic lung inhomogeneities, as previously described [1]. The fraction of total lung volume with pathologic inhomogeneities (“extent”) and the average severity of inhomogeneities contained in that fraction (“intensity”) was calculated. Respiratory system compliance and blood gas analysis were obtained at the same PEEP level of the CT scan. Some results have been presented in another publication [2].

**Results:** Forty patients were studied in the supine position 1 (0–1) days after ICU admission. The extent of pathologic lung inhomogeneities represented 18 ± 4% of total lung volume. The intensity of pathologic lung inhomogeneities was on average 2.53 ± 0.12. “Extent” was positively correlated with the amount of poorly aerated lung weight (r^2^ = 0.51, *p* < 0.001) (Fig. 1) and negatively correlated with the amount of non-aerated lung weight (r^2^ = 0.22, *p* = 0.002). No correlation was found between “extent” and “intensity” and PaO_2_/FiO_2_, dead space fraction or respiratory system compliance.

**Conclusions:** In C-ARDS lung inhomogeneities represent roughly 20% of total lung volume. In these regions local stress is increased with risk of secondary lung damage.


**References**
Cressoni M et al. Am J Respir Crit Care Med 2014;189:149–58.Protti A et al. Chest 2022;161:979–988.

**Figure Figah:**
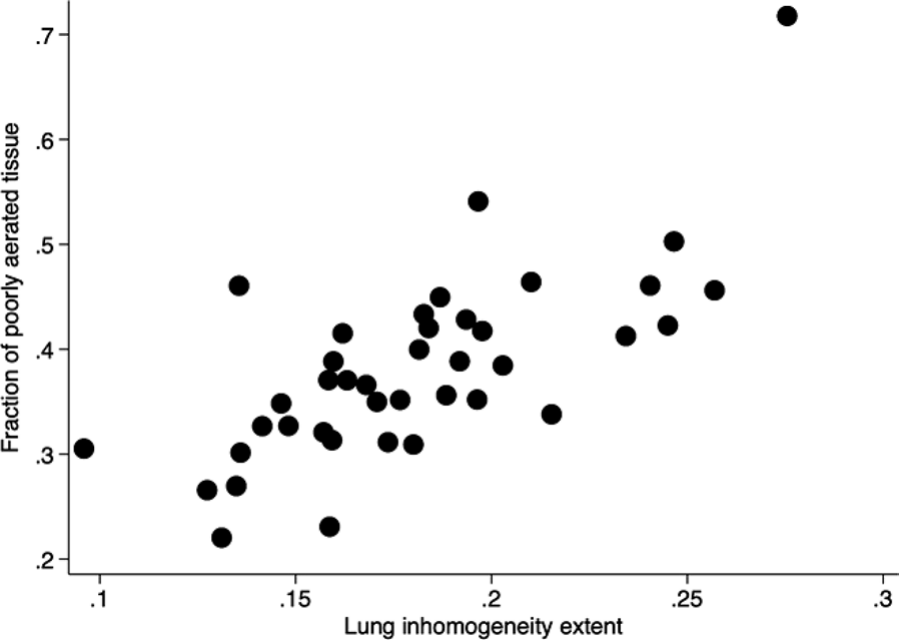
**Fig. 1 (abstract P080)** Fraction of poorly-aerated lung tissue and extent of lung inhomogeneities at PEEP 5 cmH_2_O

## P081

### Risk factors for nosocomial infections in COVID-19 ICU population

#### P Costa^1^, J Alves^1^, A Martinho^1^, C Silva^1^, L Simões^1^, G Almeida^1^, J Guimarães^2^, S Teixeira^1^, JP Baptista^1^, P Martins^1^

##### ^1^Coimbra’s University Hospital, Intensive Care Department, Coimbra, Portugal, ^2^Coimbra’s University Hospital, Cardiology Department, Coimbra, Portugal

*Critical Care* 2023, **27(S1)**: P081

**Introduction:** The aim of this study is to identify the factors associated with an increased risk of developing nosocomial infections (NI) in COVID-19 patients admitted with pulmonary involvement in the ICU. NI in COVID-19 ICU population are an important cause of morbidity and mortality worldwide and its prompt identification might lead to its prevention and better outcomes.

**Methods:** This is a retrospective observational study of patients admitted with COVID-19 pneumonia in the ICU of a tertiary center in Portugal, between March 2020 and December 2021. We considered NI as any infection acquired > 48 h post ICU admission. Clinical, analytical and baseline patient data were evaluated. Logistic regression analysis was performed to correlate patient related variables with the development of NI.

**Results:** A total of 338 patients were enrolled, from which 167 (47.9%) presented with NI. Baseline characteristics are described in Table 1. In the logistic regression analysis, older age (OR 1.13; 95% CI 1.03–1.25; *p* = 0.013), coronary artery disease (CAD) (OR 28.7; 95% CI 1.92–429; *p* = 0.02), obesity (OR 3.14; 95% CI 0.86–11.42; *p* = 0.008), chronic liver disease (CLD) (OR 104.33; 95% CI 1,.04–1008.49; *p* = 0.04), use of dexamethasone (OR 21.89; 95% CI 3.04–157.85; *p* = 0.002) and days in RASS < 3 (OR 1.4; 95% CI 1.05–1.86; *p* = 0.02) were associated with an increased risk of developing NI in the ICU. Surprisingly, SOFA at admission, days of invasive mechanical ventilation, days of sedation and PaO_2_/FiO_2_ ratio at admission, although statistically significantly different between groups, did not correlate with the risk of infection.

**Conclusions:** We identified prolonged deep sedation, corticosteroid use, and patient characteristics (CAD, obesity, CLD, older age) as independent risk factors for NI development in COVID-19 critically ill patients. It is also noteworthy to point out for the presence of confounding variables, including the excessive workload in the ICU during this period, leading to an increase in NI numbers.

**Table Tabz:** **Table 1 (abstract P081)**. Baseline patient characteristics

	With NI (n = 162)	Without NI (n = 176)	*p* value
Male sex (%)	69.1	65.0	0.46
Age (years)	59.7 (± 12.2)	58.6 (± 13.5)	0.37
Charlson comorbidity index	2.2 (± 1.6)	2.1 (± 1.7)	0.41
SOFA at admission	6.9 (± 2.8)	5.3 (± 2.9)	< 0.001
Invasive mechanical ventilation (days)	21.5 (± 13.2)	9.0 (± 4.7)	< 0.001
Sedation (days)	20.6 (± 12.0)	6.1 (± 5.7)	< 0.001
PaO_2_/FiO_2_ ratio at admission	105.9 (± 47.0)	118.5 (± 48.3)	0.02

## P082

### Incidence and microbiological characteristics of ventilator-associated pneumonia assessed by bronchoalveolar lavage and endotracheal aspirate in COVID-19 patients: COV-AP study

#### D Mangioni^1^, J Fumagalli^2^, A Meli^2^, M Tomasello^1^, C Bobbio^1^, C Matinato^3^, A Muscatello^1^, M Panigada^4^, A Bandera^1^, G Grasselli^2^

##### ^1^Fondazione IRCCS Ca’ Granda Ospedale Maggiore Policlinico, Infectious Disease Unit, Milan, Italy, ^2^Fondazione IRCCS Ca’ Granda Ospedale Maggiore Policlinico, Anesthesia, Critical Care and Emergency, Milan, Italy, ^3^Fondazione IRCCS Ca’ Granda Ospedale Maggiore Policlinico, Microbiology Laboratory, Clinical Laboratory, Milan, Italy, ^4^Fondazione IRCCS Ca’ Granda Ospedale Maggiore Policlinico, Anesthesia, Critical Care and Pain Medicine, Milan, Italy

*Critical Care* 2023, **27(S1)**: P082

**Introduction:** Diagnosis of ventilator-associated pneumonia (VAP) in COVID-19 patients remains challenging. Also, the lack of gold standard for microbiological sampling undermines clinical judgement and management. We studied incidences of microbiologically-confirmed VAP comparing endotracheal aspirate (ETA) and bronchoalveolar lavage (BAL) in COVID-19 patients. Etiological agreement between ETA and BAL was then assessed.

**Methods:** Single-center prospective cohort study (NCT04766983). Patients were enrolled within 48 h from intubation; surveillance ETA (ETA_SURV_) was performed twice weekly. ETA (ETA_CX_) and BAL (BAL_CX_) samples were collected upon VAP suspicion (Johanson’s criteria). CDC definitions were used for microbiological confirmation. ETA-BAL agreement (interrater reliability and Cohen’s kappa) and clinical/microbiological data were assessed for the first episodes of suspected VAP per patients.

**Results:** Ninety intensive care (ICU) patients enrolled from 01/2021 to 05 06/2022, of which 26 females (28.9%); median age was 60 [52–66] years. In-ICU mortality was 30/90 (33.3%), median length of stay in survivors 19 (10–32) days. Fifty-three patients (58.9%) had ≥ 1 episode of suspected VAP after 6 [5; 10] days from ICU admission. ETA_SURV_ were available in 52 cases, 2 [1; 3] days before VAP suspect, and tested positive in 28 (53.8%). ETA_CX_ and BAL_CX_ resulted positive in 35 (66.0%) and 29 (54.7%) of episodes. Main microbiological results are displayed in Fig. 1, panel A. Etiological agreement between techniques is shown in Fig. 1, panel B. Incidence rate of VAP suspicions per 1000 ventilator-days was 60.2 (95% CI 43.9–76.4), while incidence rates of microbiologically-confirmed VAP were 27.4 (18.3–36.5) with ETA_CX_and 18.9 (95% CI 12.0–25.8) with BAL_CX_, respectively.

**Conclusions:** We observed different incidence of VAP in COVID-19 ICU patients depending on sampling method. Etiological agreement between techniques yielded limited interrater reliability. The potential clinical impact needs further studies.

**Figure Figai:**
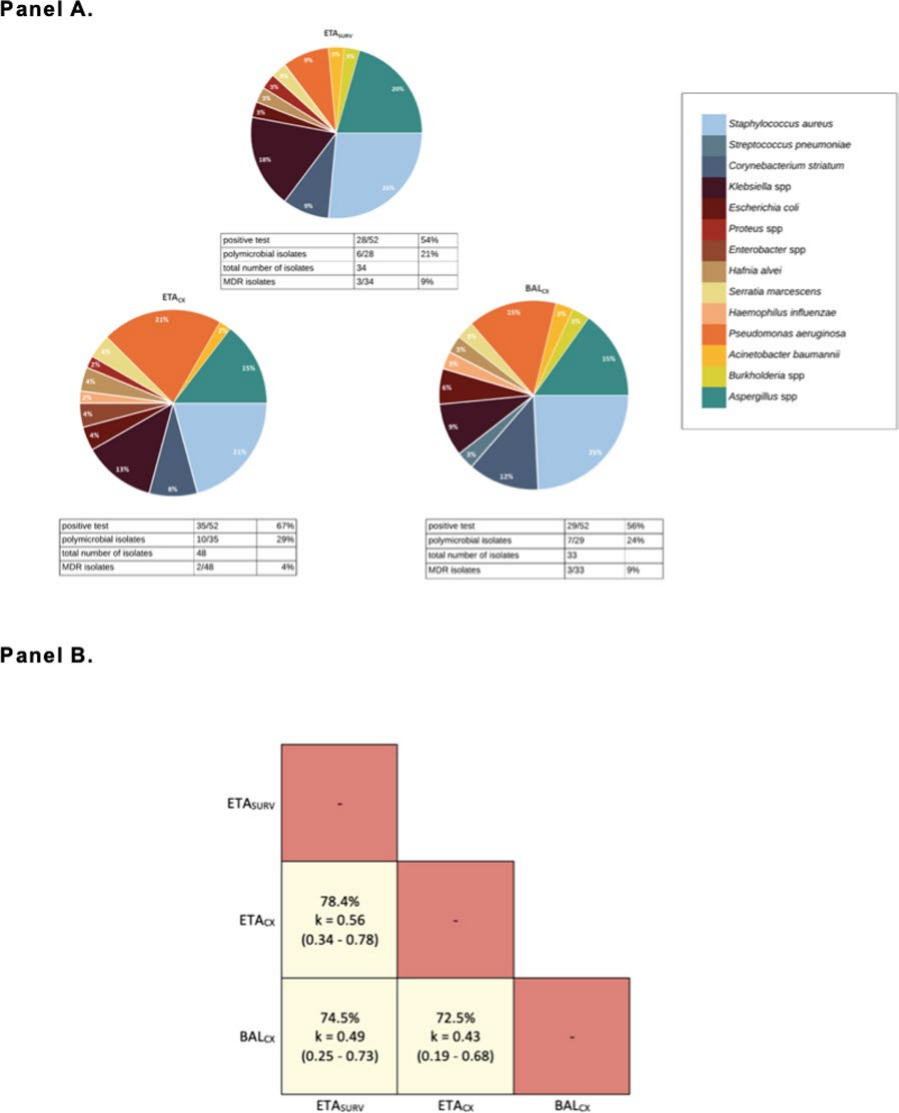
**Fig. 1 (abstract P082)**. Panel** A**: Microbiological findings of the different respiratory samples collected at the first episode of VAP suspicion; Panel** B**: Observed agreement for VAP diagnosis and interrater reliability [Cohen’s Kappa (95% CI)] for the different type of respiratory samples collected at the first episode of VAP suspicion. ETAsurv: last surveillance endotracheal aspirate obtained before the episode of VAP suspicion; ETAcx/BALcx: endotracheal aspirate/bronchoalveolar lavage obtained at VAP suspicion

## P083

### Critical illness in patients with omicron variant COVID-19: different but crucial

#### A Hana, PD Wendel Garcia, C Friedrich, D Nowak, S Keiser, RA Schüpbach, MP Hilty

##### University Hospital of Zurich, Institute of Intensive Care Medicine, Zurich, Switzerland

*Critical Care* 2023, **27(S1)**: P083

**Introduction:** During the COVID-19 pandemic, various virus variants evolved worldwide. COVID-19 omicron (CO) was associated with a decrease in length of hospital stay, ICU admission and death [1] as compared to COVID-19 delta (CD). To estimate impact of CO on ICUs worldwide, we investigated characteristics, disease course and outcome of critically ill CO patients.

**Methods:**  Of 8562 critically ill COVID-19 patients included in the prospective international multicenter RISC-19-ICU registry [2,3], characteristics and outcome were compared for 1890 CD and 272 CO patients admitted to ICU between 1–2021 and 9–2022. Mixed model analysis corrected for individual center effects and adjusted for age, sex, vaccination status, use of steroids and anticoagulants was used.

**Results:** There was no difference in age, sex and BMI between groups. CO patients had more comorbidities [mean difference (MD) 0.7, 95% CI (0.5–1.0), *p* = 0.02], especially arterial hypertension, and higher SAPS II score [MD 0.0 (0–0.1), *p* < 0.001], SOFA score [MD 0.1 (0.1–0.3), *p* < 0.0001]. CO patients presented with higher cardiovascular system SOFA subscore, but better PF-ratio at ICU admission and smaller risk for intubation and mechanical ventilation throughout their ICU stay [OR 0.5 (0.3–0.8)]. There was no difference in ECMO treatments, ICU length of stay [MD 0.6 (0–11.4), *p* = 0.72] or ICU survival [HR 1 (0.7–1.5), *p* = 0.88] between the two groups.

**Conclusions:** We show that critically ill CO patients present with more comorbidities, less severe respiratory disease but more severe hemodynamic instability at ICU admission as compared to CD patients, although the ICU length of stay and mortality was similar. These differences could be explained by differences in disease characteristics caused by CO, or by the increasing prevalence of CO as co-factor to preexisting disease. Continued monitoring of critically ill CO patients in ICUs worldwide is warranted.


**References**
Iuliano AD et al. MMWR Morb Mortal Wkly Rep. 2022;71:146–152.Wendel-Garcia PD et al. Crit Care 2022;26:199.Hilty MP et al. Intensive Care Med 2022;48:362–365.


## P084

### Clinical characteristics and outcome of older patients with severe COVID-19: a retrospective observational study

#### KS Singh, AK Kumar

##### All India Institute of Medical Sciences Patna, Anaesthesiology, Patna, India

*Critical Care* 2023, **27(S1)**: P084

**Introduction:** The objective of the study was to assess the relevant laboratory and clinical factors that may affect the prognosis of patients with severe COVID-19 in older population. Very few studies have specifically investigated the age-specific factors that affect the outcome of the patient in elderly patients [1,2].

**Methods:** The study population was adults (≥ 60 years) with severe COVID-19 admitted to the intensive care unit of a tertiary care hospital between March 2021 and June 2021. Binomial logistic regression analysis was used to analyze all variables as potential predictors for the death of older patients.

**Results:** In total, 113 older patients with severe COVID-19, with a median age of 68 years (interquartile range (IQR) 63–74), mortality rate was 61.9%. At admission, the median PO2/FiO2 ratio of the patients was 100 (IQR 90–150) and 33.6% were on mechanical ventilation. Binomial logistic regression showed that total leucocyte counts (TLC), platelet counts (PC), lactate dehydrogenase (LDH), D-dimer, and interleukin-6 levels were all significantly associated with death. Patients with poor outcomes had significantly lower PO_2_/FiO_2_ ratios at admission, higher TLC, lower PC, higher serum creatinine, and higher LDH as compared to survivals. Patients with poor outcomes had a significantly higher percentage of chronic kidney disease patients (20% vs 4%), and patients who didn’t receive remdesivir (27% vs 2.3%).

**Conclusions:** Increased TLC, LDH, D-Dimer, IL-6, and decreased PC are associated with poor outcomes. The use of remdesivir may increase the chance of survival in older patients.


**References**
Yan Y et al. BMJ Open Diabetes Res Care. 2020;8:e001343.Singhal S et al. BMC Geriatr. 2021;21:321.


## P085

### APACHE II score as a predictor of outcome in critically ill COVID-19 patients

#### M Matsko, C Bostantzoglou, A Katsogianni, K Georgiopoulos, V Karaouli, P Athanasiou, M Karagianni, C Merkouri, M Daganou.

##### ICU, Sotiria Hospital of Chest Diseases, Athens, Greece.

*Critical Care* 2023, **27(S1)**: P085

**Introduction:** APACHE II severity scoring system has been successfully used for mortality risk assessment in the ICU, however its validity in the subgroup of COVID-19 patients has been questionable. We aimed to examine the predictive value of APACHE II score in a cohort of critically ill COVID-19 patients.

**Methods:** We performed a retrospective analysis of prospectively collected data in a cohort of COVID-19 patients admitted to our 50-bed ICU between October 2020 and April 2022. Using a ROC analysis we assessed the performance of APACHE II score and identified the optimal cut-off value for mortality prediction.

**Results:** Our cohort included 783 patients (66% male) with positive PCR forSARS-Cov-2 and respiratory failure. Mean age was 66 years. Invasive mechanical ventilation was used in 92%of patients and 89.3% had at least one comorbidity. The mean APACHE II score of the whole cohort was 20.3 (± 8.5). ICU mortality was 44.7%. Death rate was similar between sexes but significantly higher in those who were older and those suffering from COPD, chronic renal or heart failure, atrial fibrillation or any kind of malignancy. Non-survivors had a significantly higher APACHE II score compared to survivors (25.2 ± 7.9 vs 16.3 ± 6.7, *p* < 0.001). ROC analysis showed an AUC 0.81 (95% CI 0.78–0.84, *p* < 0.001). At a cut-off value of 19.5 APACHE II score could predict death with a sensitivity of 77.1% (95% CI 72.4–81.4%), a specificity of 70.7% (95% CI 66.1–74.9%), PPV 68% (95% CI 63.2–72.6%) and NPV 79.3% (95% CI 74.9–83.2%).

**Conclusions:** APACHE II score is an effective tool for mortality prediction in critically ill COVID-19 patients. A cut-off value of 19.5 can be used for risk stratification in this patient population.

## P086

### Non-depolarizing neuromuscular blockade in patients with severe acute respiratory distress syndrome associated with COVID-19: profile of usage and potential deleterious impact on morbidity and mortality

#### F Bessa^1^, G Cruz^2^, P Nave^2^, SM Fernandes^3^, GN Jesus^1^, JM Ribeiro^1^

##### ^1^Centro Hospitalar Universitário Lisboa Norte, Intensive Care Department—ECMO referral centre, Lisbon, Portugal, ^2^Centro Hospitalar Universitário Lisboa Norte, Intensive Care Department, Lisbon, Portugal, ^3^Faculdade de Medicina de Lisboa, Clínica Universitária de Medicina Intensiva, Lisbon, Portugal

*Critical Care* 2023, **27(S1)**: P086

**Introduction:** Nondepolarizing neuromuscular blockade (NDMB) is a key intervention to avoid ventilation-induced lung injury in acute respiratory distress syndrome (ARDS). In patients with moderate-severe ARDS associated with SARS-CoV-2 infection (CARDS), NDMB were used for prolonged periods of time, with high cumulative doses. We hypothesize that administration of NDMB might contribute to an increased incidence of risk factors later associated with long COVID-19.

**Methods:** We designed a non-interventional, retrospective study in a large university urban hospital. From January to December 2021, data related to prescription of NDMB, respiratory physiology, mechanical ventilation (IMV) and clinical outcomes were collected from patients’ electronic records with a diagnosis of CARDS. Primary outcome was day-90 mortality. Secondary outcomes were ICU length of stay (LOS), ICU-acquired weakness and days of IMV. Mann–Whitney U test was used to compare continuous variables and logistic regression was used to evaluate the association of NDMB use with outcomes, adjusted or not for confounders.

**Results:** 116 patients diagnosed with CARDS were included, 87% with severe ARDS and overall mortality was 37.1%. Median age was 57 years (IQ:47–67) and 65.5% were male. P:F ratio at day-1 was 86 (IQ:43). Ventilator-free days (VFDs) at 28 days was 13 (IQ:0–19) in survivors and ICU-LOS was 19 (IQ:10–36). Median time and cumulative dose of NDMB were, respectively, 117 h and 1177.468 mg in patients who survived (n = 70) compared to 197 h and 1898.775 mg in patients who died (n = 41). In addition to days of NDMB exposure (OR 1.05, CI 95% 1.00–1.11), the cumulative dose of cisatracurium, expressed in logs, was correlated with risk of mortality in the ICU, with odds ratio 1.49 (CI 95% 1.08–2.04).

**Conclusions:** Patients with severe forms of CARDS received prolonged infusions of NDMB, with high cumulative doses. Both time of exposure and total doses were independently associated with higher risk of mortality.

## P087

### Prolonged prone position in COVID-19-associated ARDS: a prospective study

#### G Karlis^1^, S Kakavas^2^, M Poulou^1^, D Bakali^1^, G Katsagani^1^, M Daganou^1^, D Markantonaki^1^

##### ^1^Thoracic Diseases General Hospital Sotiria, Polyvalent ICU, Athens, Greece, ^2^Henry Dunant Hospital, Athens, Greece

*Critical Care* 2023, **27(S1)**: P087

**Introduction:** Ventilation in prone-position (PP) improves survival in moderate-to-severe ARDS. However, optimal duration of the intervention to gain maximum benefit is unknown. We sought to examine the efficacy and safety of a prolonged PP protocol in COVID-19-associated ARDS.

**Methods:** This was a prospective observational study. We included consecutive intubated and mechanically ventilated patients with ARDS and positive PCR for SARS-CoV-2 who underwent at least one session of PP from March 2021 to August 2021. PP was undertaken if P/F < 150 with FiO_2_ > 0.6 and PEEP > 10cmH_2_O. Oxygenation parameters and respiratory mechanics were recorded before PP, at the end of PP session and 4 h after supine repositioning. Patients with PP longer than 24 h (prolonged group) were compared to patients who were proned for less than 24 h (control group). The duration of PP was at the discretion of the treating intensivist.

**Results:** We recorded 56 patients (62.7% male). Five patients were excluded because PP was terminated in less than 4 h. Mean age of the 51 studied patients was 61.4 years. Patients in the prolonged group had significantly higher BMI than controls. Baseline oxygenation and respiratory mechanics were similar between groups. PP duration was 39.8 versus 20.5 h (*p* < 0.001). Increase of P/F was higher in the prolonged PP group during proning (103.8 ± 70.8 vs 66 ± 53.9, *p* < 0.05) and after supination (76.3 ± 64.6 vs 48.6 ± 34.9, *p* = 0.058). No change in respiratory mechanics was observed in either group. 28-day survival was 75% in the prolonged PP group and 69.5% in the control group (*p* = 0.665). Duration of mechanical ventilation, number of PP cycles and rate of complications were similar between groups.

**Conclusions:** In patients with ARDS due to COVID-19 prolonged PP resulted in better oxygenation, but had no impact on outcome. However, it is both feasible and safe and can be an alternative in conditions of increased work load as was the case during the recent pandemic.

## P088

### Incidence of barotrauma in an intensive care unit: new data from COVID-19?

#### JN Patrício^1^, R Jorge^1^, AA Fernandes^2^, A Gonçalves^1^, CS Pereira^1^

##### ^1^Hospital Beatriz Angelo, Intensive Care Unit, Loures, Portugal, ^2^Hospital Beatriz Angelo, Internal Medicine, Loures, Portugal

*Critical Care* 2023, **27(S1)**: P088

**Introduction:** During COVID-19 pandemic, the massive use of ventilatory support made its complications even more common. This study aimed to analyse the incidence of barotrauma in COVID-19 patients as well as its consequences.

**Methods:** Retrospective cohort study. All patients undergoing mechanical ventilation in an intensive care unit (ICU) during 2020–2021 were included. The time of both noninvasive and invasive ventilation was considered together. Statistical analysis was performed using IBM SPSS Statistics 28.0.

**Results:** A total of 967 patients were included, with 42 cases of barotrauma being reported (28 men and 14 women, median age 69 years [interval 22–94] and median APACHE 13). Out of those, 40 had severe COVID-19. Regarding patients with and without COVID-19, the incidence of barotrauma (episodes/1000 days of ventilation) was 0.64 and 9.22 (RR 14.86, *p* < 0.001) and the barotrauma rate (episodes/number of patients) was 0.4% and 8.5% (RR 21.25, *p* < 0.001), respectively. The most common type of barotrauma was subcutaneous emphysema (52.4%, CI 95% 37.3–67.5%), followed by pneumomediastinum (47.6%, CI 95% 32.5–62.7%) and pneumothorax (35.7%, IC 95% 21.2–50.2%). The median time to diagnosis was 11.5 days after initiation of ventilatory support [interval 1–67]. In the COVID-19 group, barotrauma was associated with longer ventilation (14.06 vs 7.91 days, *p* < 0.001), longer ICU stay (16.74 vs 8.17 days, *p* < 0.001) e higher mortality rates (45.0% vs 26.2%, RR 1.72, p 0.011).

**Conclusions:** We found a higher susceptibility to developing barotrauma as a potential complication of COVID-19 patients undergoing mechanical ventilation. From those, subcutaneous emphysema and pneumomediastinum seem to be more prevalent than pneumothorax. Barotrauma seems to be associated with longer periods undergoing mechanical ventilation, longer ICU stays and higher hospital mortality rates.

## P089

### Mortality in patients with COVID-19-related ARDS treated with venovenous extracorporeal membrane oxygenation: a retrospective observational study

#### R Antolini^1^, E Casarotta^2^, A Damia Paciarini^3^, G Giaccaglia^1^, E Vitali^1^, A Salvucci^1^, A Carsetti^1^, S Pantanetti^3^, V Gabbanelli^3^, A Donati^1^

##### ^1^Università Politecnica delle Marche, Ancona, Italy, ^2^Università Politecnica delle Marche, Biomedical Sciences, Ancona, Italy, ^3^AOU delle Marche, Ancona, Italy

*Critical Care* 2023, **27(S1)**: P089

**Introduction:** Venovenous extracorporeal membrane oxygenation (VV ECMO) is a technique that provides blood oxygenation and CO_2_ removal, allowing a protective ventilation strategy until the resolution of respiratory failure. A delay in ECMO initiation could worsen the outcome and prolong the duration of treatment. The study aims to describe the incidence of mortality in our intensive care unit (ICU) in patients with severe COVID-19-related acute respiratory distress syndrome (ARDS) treated with VV ECMO.

**Methods:** We performed an observational retrospective study, including patients with severe COVID-19-related ARDS admitted to our ICU and treated with VV ECMO between February 2020 and February 2022. We collected data on demographic characteristics, comorbidities, mechanical ventilation, therapies, laboratory results, VV ECMO and ICU mortality. SOFA score, SAPS II and Charlson Comorbidity Index were calculated at ICU admission.

**Results:** The average age of our cohort of 60 patients was 54.4 ± 7.7 years and 51 (85%) were males. The mean value of the SOFA score at ICU admission was 7 ± 2.3 points, and the median value of the SAPS II score was 41 [31–48] points. The incidence of mortality in the whole cohort was 48.3%. The differences between the two groups of patients, Survivors and Non-survivors, are presented in Table 1. Through a multivariate logistic regression model we found that age (OR 1.09 [95% CI 1.00–1.19], *p* = 0.03) and lymphocytes (OR 0.09 [95% CI 0.01–0.59], *p* = 0.01) were significantly associated with ICU mortality. Mechanical ventilation before ECMO placement higher than 10 days and superinfections at ICU admission were not significantly associated with the outcome in the same model.

**Conclusions:** In patients with COVID-19-related ARDS treated with VV ECMO, advanced age and lymphopenia at ICU admission are risk factors for ICU mortality. A longer duration of mechanical ventilation before ECMO placement and traditional ICU prognostic scores seem not to be relevant for the prognosis.

**Table Tabaa:** **Table 1 (abstract P089)**. Demographic and clinical characteristics of the two groups of patients

Characteristics	Survivors (n = 31)	Non-survivors (n = 29)	*p* value
Age, years	53 [48–59]	59 [50–61]	0.04
Charlson Comorbidity Index	1.2 ± 0.8	1.4 ± 0.9	0.21
SOFA score, points	7.1 ± 2.5	7 ± 2.2	0.96
Time between intubation and ECMO start, days	6 [3–9]	6 [2–9]	0.54
Gram + superinfection	25 (80.6)	24 (82.7)	0.83
Gram- superinfection	30 (96.8)	23 (79.3)	0.04
Lymphocytes, × 10^3^/mmc	0.89 [0.46–1.25]	0.56 [0.40–0.71]	< 0.01

## P090

### Can we detect in vivo hemolysis during extracorporeal membrane oxygenation support using endogenous carbon monoxide production?

#### I Seises^1^, M Serres-Gomez^2^, M Simon-Velasco^2^, KL Nanwani-Nanwani^1^, MP Sanz-de-Pedro^2^, B Fernandez-Puntero^2^, MJ Alcaide-Martin^2^, I Losantos-Garcia^3^, P Millan-Estañ^1^, R Gomez-Rioja^2^

##### ^1^University Hospital La Paz, Intensive Care Medicine, Madrid, Spain, ^2^University Hospital La Paz, Clinical Laboratory, Madrid, Spain, ^3^University Hospital La Paz, Biostatistics Department (IdiPaz), Madrid, Spain

*Critical Care* 2023, **27(S1)**: P090

**Introduction:** Extracorporeal membrane oxygenation (ECMO) has been widely used in patients with ARDS due to COVID-19. In vivo hemolysis (ivH) is one of its complications, characterised by peaks of plasma free hemoglobin (fHb). However, an increase in carboxyhemoglobin (COHb) has also been observed due to Hb metabolism by heme-oxygenase that releases carbon monoxide. The aim of this study is to evaluate the incidence of ivH events and their relation to COHb in COVID-19 patients undergoing ECMO.

**Methods:** Single-centre observational retrospective study that included 33 COVID-19 patients with ARDS who received VV-ECMO treatment in the ICU from March 2020 to September 2021. Daily analytical monitoring was carried out including arterial blood gas test with cooximetry and biochemical parameters, incorporating the estimation of fHb using quantitative hemolysis index (HI). Significant ivH was considered with fHb > 50 mg/dL after discarding in vitro hemolysis. Daily maximum values of HI and COHb were recorded and paired in order to evaluate their correlation by generalised linear model.

**Results:** The total prevalence of patients having ivH in our cohort was 27.3%. Mortality during ECMO treatment in our study was 57.6%, higher within the group of patients with ivH events (77.8% vs 50%). A total of 777 daily maximum values of fHb from all the patients were obtained. Values of COHb were significantly higher during ivH episodes. Furthermore, positive significant correlation was obtained between daily analytical values of fHb and COHb (B coefficient 42.156; *p* = 0.042), as shown in Fig. 1. The cut-off value of COHb to be discriminative for hemolysis (fHb > 50 mg/dL) was 3.85% COHb (90.5% sensitivity and 83.3% specificity).

**Conclusions:** Point-of-care carboxyhemoglobin is a cheap and widely available parameter that could be useful when detecting in vivo hemolysis during ECMO treatment.

**Figure Figaj:**
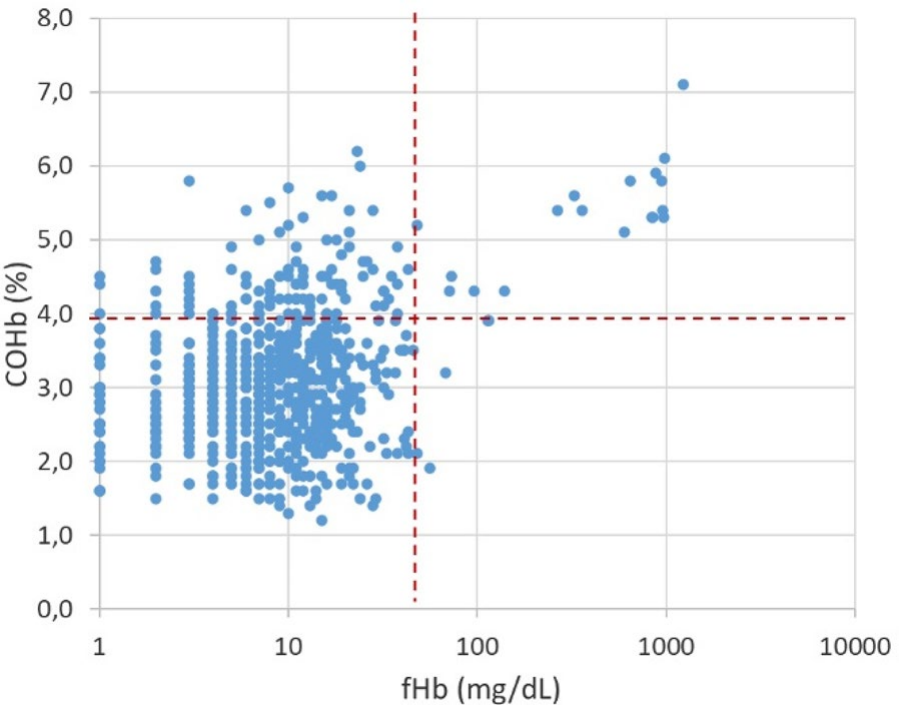
**Fig. 1 (abstract P090)**. Correlation between daily analytical values of fHb and COHb

## P091

### Bilateral non-reactive pupillary dilation during continuous muscle relaxant therapy: a retrospective study

#### Y Giladi^1^, A Giladi^2^, M Frist^3^, PD Levin^1^

##### ^1^Shaare Zedek Medical Center, Intensive Care Unit, Jerusalem, Israel, ^2^The Hubrecht Institute, Utrecht, Netherlands, ^3^Shaare Zedek Medical Center, Anesthesiology, Jerusalem, Israel

*Critical Care* 2023, **27(S1)**: P091

**Introduction:** Fixed dilated pupils in sedated ICU patients can be the first sign of brain injury. Reversible pupil dilation under rocuronium treatment has been described in case reports [1, 2]. We report a retrospective analysis of patients receiving continuous rocuronium or vecuronium to investigate the occurrence of pupil dilation.

**Methods:** Nursing data on pupil size were collected retrospectively from all adult patients receiving a continuous infusion of rocuronium or vecuronium, both during and before/after the infusion. Reversible bilateral pupil dilation ≥ 4 mm that was not responsive to light was defined as dilation. Patients with an organic cause of dilated pupils were excluded.

**Results:** Data on 197 patients admitted from 2019 to 2022 were analyzed. Rocuronium only was administered to 42 (21%) patients, vecuronium only to 134 (67%) patients and both to 23 (12%) patients. For rocuronium, transient pupil dilation occurred in 2/42 (5%) patients during treatment versus 2/42 (5%) different patients before or after treatment. Pupils were dilated for 34 ± 19 (20 ± 5%) hours during therapy versus 10 ± 6 (8 ± 4%) hours before or after therapy. For vecuronium transient pupil dilation occurred in 3/134 (2%) patients during treatment versus 4/134 (3%) other patients before or after treatment. Pupils were dilated for 12 ± 6 (23 ± 31%) hours during therapy versus 52 ± 28 (13 ± 10%) before or after therapy.

**Conclusions:** Pupil dilation seems to be more common during muscle relaxant therapy than before or after. Overall pupil dilation under muscle relaxants is rare, but seemed more common during rocuronium therapy than vecuronium. The small numbers precluded statistical significance. Familiarity with this potential side effect of rocuronium (which can be reversed with sugammadex) is important as it may prevent unnecessary CT exams in ventilated patients.


**References**
Zakynthinos GE et al. J Crit Care 2021;65:259–260.Fernandes FA et al. Braz J Anesthesiol 2022;72:829–831.


## P092

### Readiness for malignant hyperthermia crisis in intensive care unit

#### EDW Du, TCL Tan, ZXY Zhang

##### Changi General Hospital, Anaesthesiology and Intensive Care, Singapore, Singapore

*Critical Care* 2023, **27(S1)**: P092

**Introduction:** Malignant hyperthermia (MH) is a rare, life-threatening condition that manifests as a hypermetabolic response triggered by exposure to volatile anaesthetic agents and succinylcholine [1]. Prompt recognition and early treatment of a MH crisis improve survival rate. Inhaled sedation with volatile anaesthetic agents has been made feasible in intensive care unit (ICU) with the development of the anaesthetic conserving device (AnaConDa®) [2]. With its rising popularity among the intensivists, it calls for a MH crisis preparation plan for the ICU [3]. The objective of this study is to assess MH awareness, knowledge and crisis preparedness among ICU staff.

**Methods:** An online questionnaire was sent to staff working in the operating theatre (OT) and ICU in Changi General Hospital Singapore between August to October 2022. Staff included are doctors working in the anaesthesia department, nurses and attendants in the OT, ICU nurses and respiratory therapists. The survey consists of 16 single or multiple-choice questions on MH knowledge and crisis preparedness.

**Results:** Out of the 283 responders, 49.5% (140) are unsure or unaware of MH, among which ICU staff accounts for 71.5% (100). Among 114 ICU staff, only 14.0% correctly identified the signs and symptoms of MH or knew dantrolene is the drug of treatment. 17 people had prior encounters with MH either in ICU or OT settings, among them only 53% (9) chose the correct drug and dose of dantrolene. Majority of the ICU staff are not aware of the hospital MH protocol or availability of MH cart and 99.1% expressed lack of confidence in handling MH crisis (Fig. 1).

**Conclusions:** MH is a low-frequency, high fatality emergency in ICU. Current data showed a lack of awareness and preparedness for MH crisis among the staffs especially ICU colleagues. MH education plan is needed to promote early recognition, intervention and treatment of MH crisis.


**Reference**
Hopkins PM et al. Anaesthesia. 2021;76:655–664.Rosenberg H et al. Can J Anesth 2015;62:319–320.

**Figure Figak:**
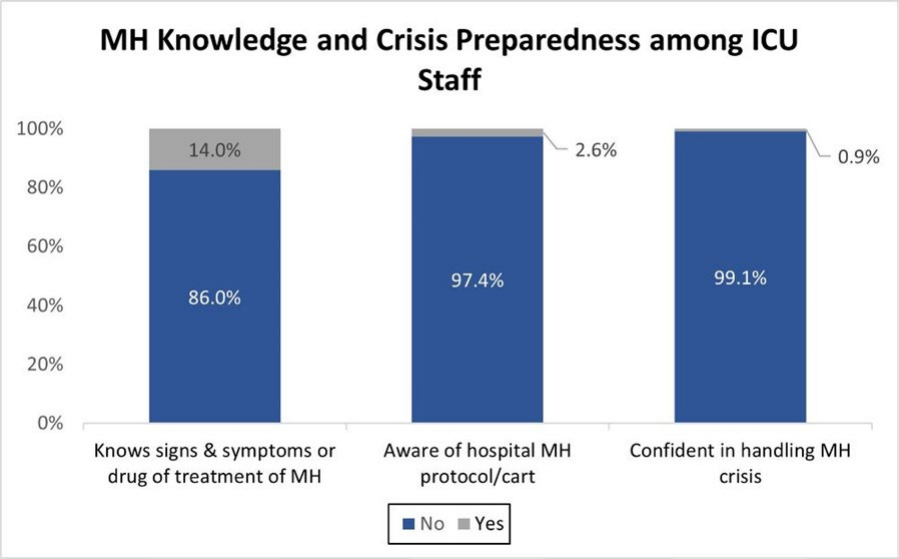
**Fig. 1 (abstract P092)**. Malignant hyperthermia knowledge and crisis preparedness among ICU staff

## P093

### Pain is a problem during critical care

#### A Wlodarczyk-Abou Elseoud^1^, K Hamunen^2^, ML Kalliomäki^3^, M Bäcklund^4^, A Laukkanen^4^, O Mäkinen^5^, AM Kuivalainen^4^

##### ^1^HUS Helsinki University Hospital, Department of Intensive Care, Helsinki, Finland, ^2^HUS Helsinki University Hospital, Department of Pain Medicine, Helsinki, Finland, ^3^Tampere University Hospital, Department of Prehospital Emergency Care, Pain Management and Anaesthesiology, Tampere, Finland, ^4^HUS Helsinki University Hospital, Department of Intensive Care, Helsinki, Finland, ^5^Tampere University, Tampere University, Tampere, Finland

*Critical Care* 2023, **27(S1)**: P093

**Introduction:** Many patients in the Intensive Care Unit (ICU) suffer from insufficient pain treatment. This leads to delay in the recovery and may evolve into chronic pain [1,2]. More information about pain intensity fluctuation and applied pain treatment throughout the stay in the ICU is needed. We conducted observational study to examine risk factors and incidence of moderate to severe pain during the first 10 days of stay in the ICU.

**Methods:** We recruited mixed population of 711 critically ill adult patients from two university hospitals in Finland. Numeric Rating Scale (NRS) and Verbal Rating Scale (VRS) were used to assess pain in patients able to communicate and Critical Care Pain Observational Tool (CPOT) for non-communicating patients. The primary endpoint of the study was the incidence of moderate to severe pain during intensive care (NRS ≥ 4/CPOT ≥ 3/VRS ≥ moderate pain). We collected data including pain intensity and other pain related factors during first 10 days in the ICU.

**Results:** 76% patients experienced moderate to severe pain during at least one of ten days of follow up in the ICU. Age below 64 years old, female gender and history of chronic pain was associated with higher number of days in moderate to severe pain. There was not significant difference between surgical and non-surgical groups of patients in the days they experienced moderate to severe pain. Pain scores throughout 10 days follow up are presented in Fig. 1.

**Conclusions:** Our results indicate that majority of patients experienced moderate to severe pain during ICU stay. We need to improve the pain assessment, especially in high-risk ICU patients. Further studies of pain trajectories during critical illness as well as identifying patient and treatment related risk factors for severe pain, will help improve pain treatment protocols and patients’ outcomes.


**References**
Puntillo KA et al. Am J Respir Crit Care Med 2014;189:39–47.Payen JF et al. Anesthesiology 2009;111:1308–16.

**Figure Figal:**
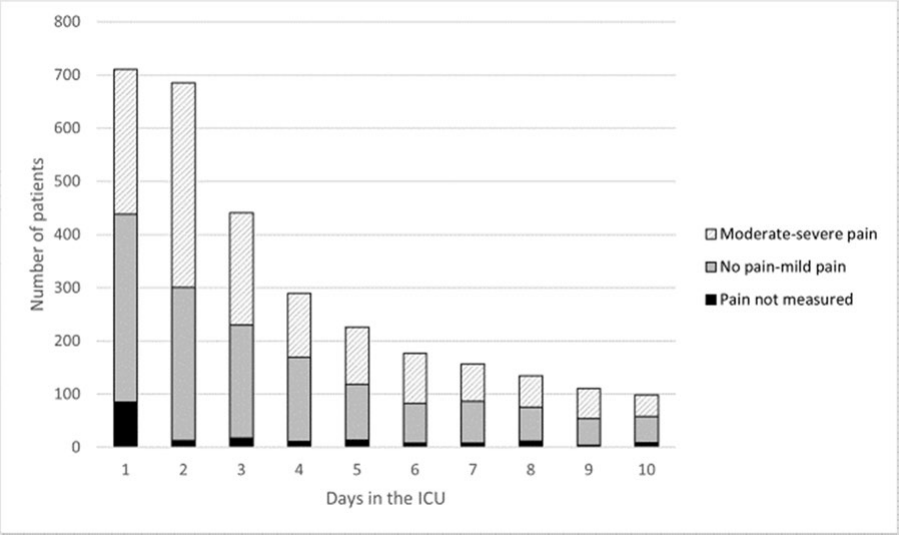
**Fig. 1 (abstract P093)**. Patients’ pain scores during 10 days follow up in the ICU. Patient had moderate to severe pain if NRS ≥ 4/CPOT ≥ 3/VRS ≥ moderate pain

## P094

### Multimodal versus opioid analgesia in cardiac surgery (MONDAY)

#### NB Belkaid^1^, SB Bogaert^2^, AM Moerman^3^, SB Bouchez^4^, HP Peperstraete^1^

##### ^1^Ghent University Hospital, Department of Intensive Care Medicine, Ghent, Belgium, ^2^AZ Groeninge, Department of Anesthesiology and Intensive Care, Kortrijk, Belgium, ^3^Ghent University Hospital, Department of Anesthesiology, Ghent, Belgium, ^4^OLV Ziekenhuis, Department of Anesthesiology, Aalst, Belgium

*Critical Care* 2023, **27(S1)**: P094

**Introduction:** Cardiac surgery performed by sternotomy is associated with moderate to severe acute postoperative pain. Our goal is to compare standard opioid based regimen to a multimodal pain management to determine which therapy provides the most comfort, the fastest extubation time, the least pain and the least delirium.

**Methods:** In this prospective randomized double-blinded trial we evaluated two groups of 50 patients, divided in an opioid group and a multimodal group. In the multimodal group pregabaline 75 mg, dexmedetomidine, ketamine and lidocaine was given versus liberal fentanyl and remifentanil in the opioid group. Postoperative pain was scored every 8 h by the NRS-scale/CPOT score in the awake/sedated patient. Delirium postoperatively is measured by the ICDSC-score. Time to extubation, LOS in ICU, LOS in hospital and rescue pain medication are compared between both groups.

**Results:** Ninety-six patients were successfully enrolled. There were no differences in baseline characteristics between both groups. The multimodal pain scheme was administered in 46 patients, while 50 patients received the opioid based scheme. After linear mixed model analysis no significant differences were seen in CPOT-score (*p* = 0.305), NRS-score (*p* = 0.182) (Fig. 1), consumption in rescue pain medication and ICDSC-score (*p* = 0.267). In the opioid group, mean LOS in ICU was 34 h in the opioid group versus 51 h in the multimodal group, (*p* = 0.032). Time to extubation or LOS in hospital showed no significant difference between both groups.

**Conclusions:** This trial shows a significant difference in LOS in ICU in favor of the opioid group. No significant difference could be found in pain, delirium, intubation time or LOS in hospital between multimodal analgesia versus an opioid-based analgetic protocol in cardiac surgery by sternotomy. This is not in line with previous studies but is probably because this trial is underpowered. It shows that there is a place for multimodal analgesia in cardiac surgery and it can be a good alternative for opioid based protocols.

**Figure Figam:**
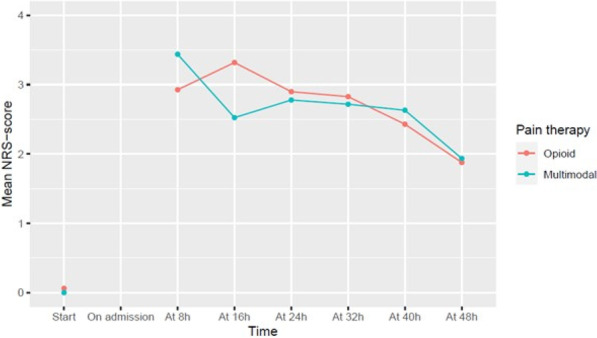
**Fig. 1 (abstract P094)**. Mean NRS-score in both groups

## P095

### Analgesic potential of a serratus anterior plane block, placed on ICU arrival, after totally endoscopic aortic valve replacement (TEAVR)

#### J Dubois^1^, I Callebaut^2^, D Vranken^2^, I Gruyters^2^, H Jalil^2^, M Van Tornout^2^, M Brands ^2^, J Herbots^2^, B Stessel^2^, J Vandenbrande^2^

##### ^1^Jessa, Critical Care, Hasselt, Belgium, ^2^Jessa, Anesthesiology & Intensive Care, Hasselt, Belgium

*Critical Care* 2023, **27(S1)**: P095

**Introduction:** Regional anesthesia is of growing importance in postoperative pain control. We examined whether a Serratus Anterior Plane Block (SAPB), placed on arrival at the Intensive Care Unit (ICU), in addition to standard-of-care after TEAVR could improve the quality of postoperative pain management.

**Methods:** We performed the first double-blinded randomized controlled trial on 75 patients undergoing TEAVR. Patients allocated to the intervention arm received a SAPB on ICU arrival when the patient was sedated. SAPB was placed by a block team, leaving the attending anesthesiologist, surgical team and critical care team blinded. Ultrasound-guided SAPB was performed on the right hemithorax at T2-T5 level: 30 ml of bupivacaine 0.25% was injected under the serratus anterior (SA) muscle and 10 ml bupivacaine 0.25% was administered between the latissimus dorsi and SA muscle. Control patients were approached by the block team for the same time span, though no SAPB was performed. Further analgesic treatment included acetaminophen and ketorolac unless contra-indications, as well as piritramide patient-controlled analgesia (PCA). Sedation was provided by means of dexmedetomidine.

**Results:** Thirty-seven patients were allocated to the control versus 38 to the SAPB group. Patients receiving SAPB suffered significantly less pain at 4, 8 and 24 h postoperatively (p values: 0.05, 0.03 and 0.04 respectively) (Fig. 1). Also 24 h-cumulative piritramide PCA consumption was significantly lower in the SABP group (median 12 mg, IQR 8–26 mg) versus control group (median 20 mg, IQR 15-31 mg) (*p* < 0.01). No differences in intraoperative use of sufentanil (*p* = 0.20) and ketamine (*p* = 0.59) neither postoperative use of NSAIDS (*p* = 0.18) and dexmedetomidine (*p* = 0.39) were observed. Time to extubation neither ICU length of stay was influenced by SAPB.

**Conclusions:** SAPB added to standard-of-care after TEAVR improved postoperative pain control, by significantly decreasing opioid consumption and pain scores in the first 24 h after surgery.

**Figure Figan:**
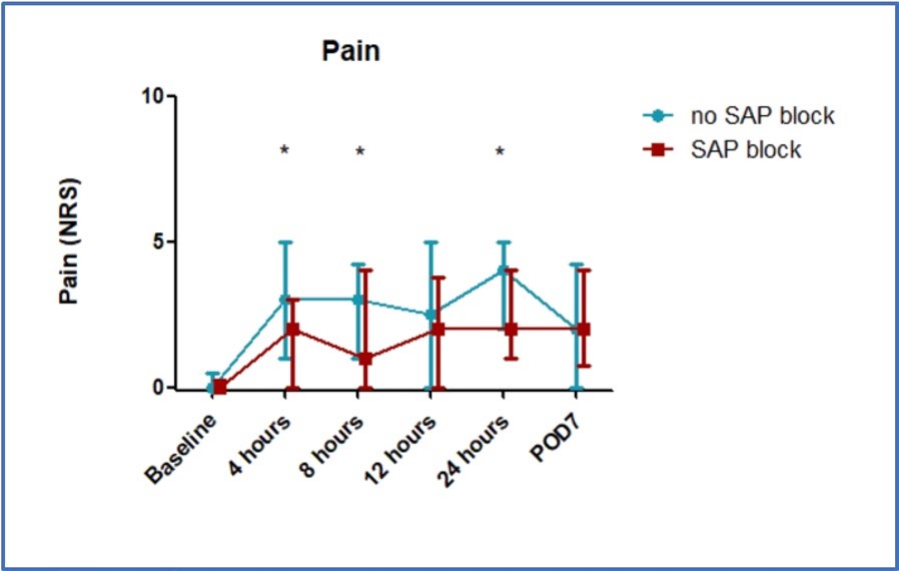
**Fig. 1 (abstract P095)**. Evaluation of pain by a blinded assessor at predefined time points after ICU arrival. Pain was assessed by means of the Numeric Rating Scale (NRS) in which 0 = no pain versus 10 = worst pain ever experienced. Statistically significant differences are marked with an asterisk (*)

## P096

### Impact of fentanyl versus remifentanil in critical care outcomes after cytoreductive surgery plus hyperthermic intraperitoneal chemotherapy (CRS-HIPEC)

#### G Madrid, A Obando, JD Guerra, JP Caceres

##### University Hospital Fundacion Santa Fe de Bogota, Bogotá, Colombia

*Critical Care* 2023, **27(S1)**: P096

**Introduction:** Anesthetic techniques and intraoperative care have an impact on outcomes after major surgery [1]. Remifentanil and fentanyl are strong opioids widely used intraoperatively due to their potency and pharmacokinetic properties. We designed a study to evaluate associations between these opioids and critical care and clinical outcomes in patients who underwent CRS-HIPEC.

**Methods:** A historical cohort of patients taken to CRS-HIPEC from 2007 to 2020 at a university hospital in Bogota, Colombia was analyzed retrospectively. We compared outcomes of patients who received intraoperative remifentanil (group R) or fentanyl (group F). Primary outcome was ICU length of stay (LOS), and secondary outcomes were total LOS, ICU readmission, inpatient mortality, time vented, need for tracheostomy, time to ambulation and oral feeds, need for transfusion, estimated blood loss, intraoperative crystalloids, and units of red blood cells and plasma transfused in surgery. We excluded patients with incomplete medical records or whose procedure was palliative.

**Results:** A total of 173 patients were included in the analysis. Mean age was 52 ± 11.4 years and female sex was preponderant (73%). 75 patients (43%) received intraoperative remifentanil and 98 (57%) fentanyl. Group R was associated with a shorter ICU stay (mean 5.9 days vs. 6.4 days in group F, *p* < 0.018). There were also statistically significant associations regarding days until extubation (Group R 3.1 vs 3.7 Group F, *p* < 0.01), need for transfusion, and estimated blood loss (Table 1).

**Conclusions:** Our results suggest an association of remifentanil and shorter ICU LOS and earlier extubation. We believe that the difference, although statistically significant, is arguably clinically significant.


**Reference**
Myles PS et al. Anesth Analg 2002;95:805–12.

**Table Tabab:** **Table 1 (abstract P096)**. Results

	Fentanyl		Remifentanil	
	Mean (SD)	Median IQR	Mean (SD)	Median IQR
ICU stay*—days	6.4 (15)	3 (3)	5.9 (6.5)	3 (4)
Days on vent*	3.7 (16)	1 (1)	3.1 (12)	1 (1)
Estimated blood loss*—ml	1756 (1944)	1000 (1490)	2137 (1835)	1765 (2000)
	Yes (%)	No (%)	Yes (%)	No (%)
Transfusion^	60 (61)	38 (39)	64 (85)	11 (15)

*Correlations with Mann–Whitney test and *p* < 0.05; ^Correlations with Pearson χ2t and *p* < 0.05

## P097

### Ketamine-fentanyl versus fentanyl for analgosedation in postoperative surgical intensive care unit patients.

#### N Kitisin^1^, K Wongtangman^1^, P Kumtip^2^, O Chaiwat^1^, N Raykateeraroj^1^, P Somnuke^1^

##### ^1^Faculty of Medicine, Siriraj Hospital, Mahidol University, Department of Anesthesiology, Bangkok, Thailand, ^2^Suratthani Hospital, Department of Anesthesiology, Suratthani, Thailand

*Critical Care* 2023, **27(S1)**: P097

**Introduction:** Ketamine at sub-anesthetic dose has been shown to provide sedative-analgesic effect and decrease opioid requirement in postoperative patients. We plan to evaluate the effects of low-dose ketamine on opioid consumption in adult surgical critically ill patients and observe its side effects.

**Methods:** A randomized, double-blinded controlled trial was conducted in the postoperative ICU patients. Fentanyl was initially titrated until Numerical Rating Scale (NRS) was < 4 and Richmond Agitation-Sedation Scale (RASS) -2 to 0. Patients received fentanyl 20 mcg/hour continuously with either ketamine (Group K) or placebo (Group C). Ketamine 0.09 mg/kg/hour (1.5 mg/kg/min) was administrated in Group K, and 0.9%NaCl was administrated at the same rate in Group C. The ICU nurse assessed NRS and RASS score in every 4 h and gave extra fentanyl bolus intravenously to keep the patients’ NRS < 4 and RASS 0 to − 2.

**Results:** 118 patients were included in the study. The patients’ characteristics were not significantly different between two groups. The fentanyl consumption at 4–8 h and 8–12 h after randomization in a ketamine group have significantly less fentanyl consumption compared to a control group (91.8 mcg vs 121.3 mcg, *p* = 0.002 and 95.6 mcg vs 121.1 mcg respectively, *p* = 0.012) (Fig. 1). The cumulative fentanyl consumption at 24 h was not different between ketamine and control group (445.8 mcg vs 499 mcg, *p* = 0.38). The ICU length of stay, duration of mechanical ventilation and adverse effects were not significantly different.

**Conclusions:** The use of low dose ketamine combined with fentanyl can reduce fentanyl consumption in postoperative SICU patients especially in the first 12 h without increase any adverse events.

**Figure Figao:**
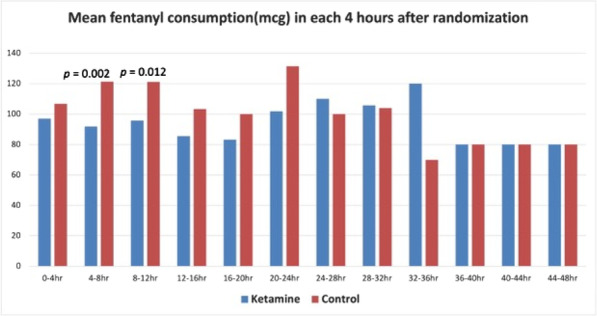
**Fig. 1 (abstract P097)**. Mean fentanyl consumption (microgram) in each 4 h after randomization

## P098

### Enteral clonidine in adult intensive care patients experiencing drug tolerance and iatrogenic withdrawal with weaning of dexmedetomidine

#### JR Berkan, TS Lam, JT Jancik

##### Hennepin Healthcare, Pharmacy, Minneapolis, USA

*Critical Care* 2023, **27(S1)**: P098

**Introduction:** Iatrogenic withdrawal has been reported in up to 73% of patients exposed to dexmedetomidine (DEX) for as few as 3 days [1, 2]. Use of enteral clonidine (CLN) to wean off DEX and manage withdrawal has become more prevalent in the intensive care unit (ICU). This study aimed to evaluate the relationship between DEX exposure and initiation of CLN in adult patients in the ICU.

**Methods:** A single-centered, retrospective cohort study was conducted in adult ICU patients admitted between January 1, 2021 and July 31, 2022 who received at least 48 h of continuous DEX. Patients weaned off DEX alone were compared to patients weaned with enteral CLN. The primary outcome was time (days) from start of wean to cessation of DEX. Secondary outcomes included DEX exposure and CLN utilization. Withdrawal (RASS > 1, HR > 100, and re-initiation of DEX or start of CLN within 48 h of DEX cessation) was assessed in patients weaned off DEX alone.

**Results:** A total of 267 patient encounters were included. Total days on DEX was greater in the CLN group (6 vs. 3 days). Days on DEX prior to wean were similar between groups, with no difference in weight-based dose of DEX (1 vs. 1 mcg/kg/hr). Time from start of wean to cessation of DEX was longer in the CLN group (3 vs. 0 days). The most common initial CLN regimen was 0.1 mg every 8 h, with a median total daily CLN dose of 0.4 mg at time of DEX cessation. At 48 h, 18.1% of patients weaned off DEX alone were re-initiated on DEX or started on CLN.

**Conclusions:** Use of enteral CLN to wean DEX may reduce rates of DEX resumption due to iatrogenic withdrawal.


**References**
Bhatt K et al. Crit Care Expl. 2020;2:e0245.Glaess S et al. Am J Health Syst Pharm. 2020;77:515–522.


## P099

### Bispectral index is useful for assessing sleep quality in postoperative patients in ICU

#### P Sirilaksanamanon^1^, S Charuluxananda^1^, T Thawitsri^1^, N Chirakalwasan^2^

##### ^1^King Chulalongkorn Memorial Hospital, Faculty of Medicine, Chulalongkorn University, Department of Anesthesiology, Bangkok, Thailand, ^2^King Chulalongkorn Memorial Hospital, Faculty of Medicine, Chulalongkorn University, Department of Medicine, Bangkok, Thailand

*Critical Care* 2023, **27(S1)**: **P099**

**Introduction:** Monitoring and improving sleep quality may help to enhance recovery from critical illness. Although polysomnography is the gold standard for assessing sleep quality, it remains impractical for routine use due to its complexity and cost. We aimed to investigate diagnostic accuracy of alternative monitor which is Bispectral Index (BIS) in assessing sleep quality in postoperative patients in ICU.

**Methods:** This study was an observational study. We included 33 adult patients who underwent elective major surgery and required postoperative ICU admission with mechanical ventilation. After postoperative admission to the ICU, polysomnography and BIS were recorded overnight and the data were obtained for analyses. The primary outcome of this study was BIS index best predicting good sleep quality. We defined postoperative sleep quality by polysomnographic sleep efficiency more than 85%.

**Results:** Receiver operating characteristics (ROC) curve (Fig. 1) was created to determine the averaged BIS index associated with good postoperative sleep quality. Area under the ROC curve (AUC) was 0.65. The optimal cut-off with best differentiation derived from Youden’s Index was 75 with sensitivity 68% and specificity 56%. When comparing the subjects with good and poor postoperative sleep quality, major postoperative outcomes including length of mechanical ventilation and ICU stay were not significantly different. Although all subjects with postoperative delirium had poor postoperative sleep quality, incidence of delirium between group was not significantly different (0% vs 10.3%; *p* = 0.184).

**Conclusions:** Bispectral Index (BIS) monitoring is a feasible tool for assessing sleep quality with acceptable accuracy in postoperative patients requiring mechanical ventilation in ICU. However, larger controlled studies are required to investigate the impact of sleep quality monitoring on clinical outcomes.

**Figure Figap:**
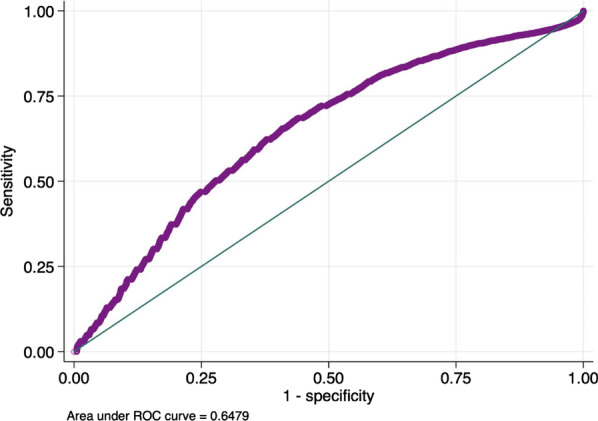
**Fig. 1 (abstract P099)**. ROC curve of BIS associated with good postoperative sleep quality

## P100

### Pursuing the real midazolam clearance during continuous renal replacement therapy in ICU patients with COVID-19: are midazolam and metabolites dialyzable?

#### TJL Smeets^1^, HRH De Geus^2^, BCP Koch^1^, D Gommers^2^, H Endeman^2^, NGM Hunfeld^1^

##### ^1^Erasmus MC University Medical Center, Hospital Pharmacy, Rotterdam, Netherlands, ^2^Erasmus MC University Medical Center, Intensive Care Adults, Rotterdam, Netherlands

*Critical Care* 2023, **27(S1)**: P100

**Introduction:** Midazolam based continuous iv sedation became again prominent during the COVID-19 pandemic. However, this sedation therapy is associated with a high incidence of benzodiazepine-related delirium and an increased number of days spent in coma. Given the high midazolam dose requirements in some patients and due to the renal clearance (CL) of the active metabolite 1-OH-midazolam-glucuronide (OHmidazolamGluc), ICU patients with COVID-19 and continuous renal replacement therapy (CRRT) may are at risk of prolonged sedation. Therefore, the aim of the study was to investigate the CL of midazolam and metabolites in 5 critically ill COVID-19 patients with CRRT.

**Methods:** Pre-filter blood samples and ultrafiltrate samples were collected simultaneously. Midazolam, 1-OH-midazolam (OHmidazolam) and OHmidazolamGluc plasma samples were analysed by an UPLC-MS/MS method. CL of midazolam and metabolites were calculated by the delivered renal dose and sieving (SC) coefficient. Subsequently, the CL and delivered renal dose were corrected for downtime therapy and filter integrity by a filter urea ratio.

**Results:** We included 4 cases of CVVHD and 2 cases of CVVHDF. Midazolam, OHmidazolam and OHmidazolamGluc concentrations in μg/l ranged from 0 to 6070, 0 to 295 and 1727 to 39,000, respectively. SCs ranged from 0.02 to 0.03 for midazolam, 0.05 to 0.06 for OHmidazolam and 0.23–0.43 for OHmidazolamGluc. The CL in ml/min by the delivered renal dose was 0.82–1.67 for midazolam, 2.20–3.46 for OHmidazolam and 4.0–27.65 for OHmidazolamGluc. The CL in ml/min by the corrected renal dose was 0.68–1.50, 1.83–2.33 and 3.40–25.4, respectively. The urea ratios were 0.53 to 1.0.

**Conclusions:** Midazolam and OHmidazolam are not removed efficiently by CRRT and OHmidazolamGluc approximately up to 43%. Type of CRRT, filter integrity and downtime of CRRT affect the CL of midazolam and metabolites. Our results have implications for more personalized titration of midazolam in COVID-19 patients with CRRT, mainly to avoid oversedation.

## P101

### Incidence of nephrogenic diabetes insipidus during prolonged sevoflurane sedation in the intensive care unit: a retrospective analysis

#### KVE Van Eeckhout^1^, BS Sol^2^, JG Glissenaar^3^, JP Poelaert^1^, BB Bravenboer^4^, JDF De Filette^5^

##### ^1^Universitair Ziekenhuis Brussel, Intensive Care Unit, Jette, Belgium, ^2^AZ Alma, Endocrinology, Eeklo, Belgium, ^3^Universitair Ziekenhuis Brussel, Anesthesiology and Perioperative Medicine, Jette, Belgium, ^4^Universitair Ziekenhuis Brussel, Endocrinology, Jette, Belgium, ^5^CHU Brugmann, Endocrinology, Brussels, Belgium

*Critical Care* 2023, **27(S1)**: P101

**Introduction:** Sevoflurane is a halogenated inhalational anesthetic used for maintenance anesthesia. It is used in the intensive care unit (ICU), not in the least because of its potentially beneficial effects in patients with Acute Respiratory Distress Syndrome. However, polyuria and nephrogenic diabetes insipidus (NDI) were observed with its long-term administration. The objective of this study is to assess the incidence of NDI during prolonged sevoflurane sedation.

**Methods:** A retrospective analysis of patients receiving prolonged sevoflurane sedation (> 24 h) at the University Hospital of Brussels from 2013 to March 2021 was performed. The diagnosis of NDI was ‘confirmed’ in the presence of polyuria (urine output > 40 ml/kg/24 h), hypernatremia > 145 mmol/l, plasma osmolality > 295 mOsm/kg, urine osmolality < 300 mOsm/kg, and no or minimal response to exogenous desmopressin administration. Partial DI was established when urine osmolality was 300–600 mOsm/kg. A ‘possible’ diagnosis of DI was suggested when polyuria, hypernatremia and high plasma osmolality occurred during sevoflurane sedation in the absence of any other reasonable pathology.

**Results:** A total of 106 patients were included. Table 1 illustrates the baseline characteristics of the study population. NDI was confirmed in 9/106 patients (8.5%), of which 5 patients with partial NDI and 4 patients with complete NDI after desmopressin administration. Possible DI was observed in 16/106 patients (15%), but could not be confirmed due to absence of urine osmolality and/or density.

**Conclusions:** The occurrence of NDI during prolonged sevoflurane sedation is not uncommon and may be underestimated by clinicians. Close monitoring of diuresis, plasma sodium, plasma and urinary osmolality during prolonged sevoflurane administration is advisable for early diagnosis and treatment of NDI.

**Table Tabac:** **Table 1 (abstract P101)**. Demographics of patients receiving prolonged sevoflurane sedation in the intensive care unit

	No NDI group	Confirmed NDI group	Possible NDI group
Age (years), mean ± SD	59.6 ± 14.9	55.0 ± 15.4	55.7 ± 9.1
Male sex (%)	69.1	66.7	56.3
SAPS II on ICU admission, median (IQR)	41 (28)	22 (24)	31.5 (29)
Baseline plasme Na (mmol/l), mean ± SD	141 ± 4.6	140 ± 3.52	141 ± 2.38
Baseline urine output (ml/24 h), mean ± SD	1963 ± 1321	1491 ± 862	2249 ± 1754
Sevoflurane end-tidal concentration (%), mean ± SD	1.11 ± 0.28	1.38 ± 0.50	1.22 ± 0.30
Sevoflurane exposure time (h), mean ± SD	127 ± 98	190 ± 79	166 ± 63

## P102

### A systematic review and meta-analysis of the incidence of adverse events in adults undergoing procedural sedation in the emergency department

#### P Samara

##### University of Leicester, MRes Applied Health Research, Leicester, UK

*Critical Care* 2023, **27(S1)**: P102

**Introduction:** Procedural sedation is used in the emergency departments to facilitate the implementation of painful and distressing procedures. Several medications are used and their safety is quantified by estimating their adverse events. This systematic review and meta-analysis aimed to evaluate the incidence of adverse events of pharmacological agents used for procedural sedation of haemodynamically stable adults in the emergency department.

**Methods:** Three electronic databases were searched (Medline, Embase, Cochrane) to identify relevant literature after 2010. Randomised controlled trials comparing medications used for procedural sedation and their side effects were included in the review. After evaluating their quality, data extraction was completed for study characteristics and the outcomes of interest (agitation, hypotension, bradycardia, oxygen desaturation, apnoea, nausea/vomiting, intubation, laryngospasm, aspiration and subclinical respiratory depression. A meta-analysis of the results of the individual studies was performed using the fixed-effects model and reported as incidence with 95% confidence intervals.

**Results:** The search revealed 1004 studies, eight of which fulfilled the eligibility criteria and were included in this review. A total of 1103 patients were included in this review and meta-analysis, which focused on the study of ketamine, propofol, ketamine combined with midazolam and ketamine combined with propofol. Generally, the incidence of agitation was the highest at 14.90% (95% CI 12.67, 17.35) and the incidence of nausea and vomiting was the lowest at 4.30 (95% CI 12.67, 17.35). The rates of adverse events were lower with ketofol and higher with ketamine alone. There were no serious events present in this review.

**Conclusions:** A decision on the choice of medication used for procedural sedation depends on many factors. According to this review, there is potential in the combination of propofol and ketamine because it reduces the incidence of some of the adverse events present when using these drugs alone.

## P103

### Methadone for sedation weaning in patients receiving extracorporeal membrane oxygenation: a retrospective observational study

#### C Remmington^1^, V Liew^2^, F Hanks^1^, L Camporota^3^, A Sousa^3^, O Stubbs^3^, N.A Barrett^3^

##### ^1^Guy’s and St Thomas’ NHS Foundation Trust, Pharmacy Department, London, UK, ^2^Royal Melbourne Hospital, Department of Intensive Care, Melbourne, Australia, ^3^Guy’s and St Thomas’ NHS Foundation Trust, Department of Adult Critical Care, London, UK

*Critical Care* 2023, **27(S1)**: P103

**Introduction:** The aim of this study is to describe the effect of methadone on analgesia and sedation weaning and reported adverse effects in ECMO. Weaning analgesia and sedatives can be challenging following recovery from critical illness. Methadone is increasingly being used to facilitate weaning, however, limited data exists describing its use as a weaning strategy in adult patients receiving extracorporeal membrane oxygenation (ECMO) [1, 2].

**Methods:** Single-centre retrospective observational study including adult patients receiving ECMO for severe respiratory failure who were administered methadone between 2016 and 2022. We recorded demographics, methadone and sedation doses, adverse reactions and Richmond Agitation Sedation Scale (RASS) scores.

**Results:** Twenty-five patients were included. Median (IQR) age and weight were 45 (34, 52) years and 80 (75, 95) kg respectively. More patients were male (68% vs 32%). Median (IQR) APACHE II score were 17 (14, 20). Median (IQR) daily starting and peak doses were 30 (20, 40) and 40 (30, 50) milligrams (mg). Median (IQR) treatment duration and time to starting methadone from admission to ICU were 7 (5, 17) and 26 (5, 39) days. Median (IQR) daily doses of fentanyl, midazolam and propofol at day 0 and 5 days post methadone initiation were 6200 (4613, 8600) vs 3150 (0, 6042) micrograms/day; *p* < 0.001, 241 (151, 410) vs 130 (10, 273) mg/day; *p* = 0.30 and 4000 (1874, 5593) vs 3090 (0, 4373) mg/day; *p* = 0.04, respectively. Median (IQR) percentage of time in target RASS -2 to + 1, improved from 50 (5, 89) % to 88 (0, 100) %; *p* = 0.004. Adverse effects were: QTc prolongation in 2/25 patients, constipation in 2/25 patients and hypotension requiring noradrenaline in 1/25 patient.

**Conclusions:** Methadone was safe and effective at reducing analgesia and sedation in ECMO patients. Further studies are required to assess the effectiveness of methadone in this population.


**References**
Al-Qadheeb NS et al. Ann Pharmacother. 2012; 46:1160–6.Wanzuita R et al. Crit Care. 2012;16:R49.


## P104

### Sedation in the ICU: association between dexmedetomidine and 28-day mortality in patients with COVID-19

#### P Costa^1^, J Alves^1^, A Martinho^1^, C Silva^1^, L Simões^1^, G Almeida^1^, J Guimarães^2^, S Teixeira^1^, JP Baptista^1^, P Martins^1^

##### ^1^Coimbra’s University Hospital, Intensive Care Department, Coimbra, Portugal, ^2^Coimbra’s University Hospital, Cardiology Department, Coimbra, Portugal

*Critical Care* 2023, **27(S1)**: P104

**Introduction:** The aim of our study is to evaluate the relationship between dexmedetomidine (DEX) use as a sedative agent in mechanical ventilated ICU patients and 28-day mortality. DEX, a selective alfa-2 adrenergic receptor agonist, widely used for its sedative and analgesic properties, has been linked to increasing parasympathetic tone, reducing the inflammatory response and oxidative stress [1]. Since severe COVID-19 is associated with an hyperinflammatory state, it is hypothesized that DEX might improve outcomes in these patients.

**Methods:** This is a retrospective observational study of mechanically ventilated patients admitted with COVID-19 pneumonia in the ICU of a tertiary center in Portugal, between March 2020 and December 2021. Logistic regression analysis was performed to evaluate the association of DEX use and 28-day mortality from time of intubation.

**Results:** A total of 277 patients were analyzed, 151 in the DEX group and 126 in the no DEX group. Patients in the DEX group were younger (53.3 vs. 63.3 years, *p* < 0.001), had less comorbidities (2.8 vs. 3.5, *p* = 0.01), lower SOFA at admission (6.2 vs. 7.1, *p* = 0.01) but had a prolonged ICU stay (21.4 vs. 15.9, *p* < 0.001). Male gender (65.6 vs. 69.0, *p* = 0.54), incidence of obesity (56.3 vs. 46.8, *p* = 0.12), coronary artery disease (4.0 vs. 7.9, *p* = 0.16) and atrial fibrillation (4.0 vs. 7.1, *p* = 0.25) were similar between groups. PaO_2_/FiO_2_ ratio at admission (111.1 vs. 108.1, *p* = 0.61), days spent in RASS < 3 (13.7 vs. 12.4, *p* = 0.31) and opioid use (14.8 vs. 13.1, *p* = 0.16) were also similar. From time of intubation, 28-day mortality in the cohort receiving DEX was 14.7% compared to 59.5% in the no DEX group (OR 0.12; 95% CI 0.07–0.21; *p* = 0.01).

**Conclusions:** Use of DEX was associated with lower 28-day mortality in COVID-19 critically ill patients requiring invasive mechanical ventilation in our study analysis. Considering the limitations of a retrospective observational study, RCTs are needed to confirm the results.


**Reference**
Ma J. Anesth Analg. 2020;130:1054–1062.


## P105

### Safety and efficacy of phenobarbital for the treatment of alcohol withdrawal syndrome in patients admitted to the medical intensive care unit

#### CE Monzel, KA Considine, TS Lam, JT Jancik

##### Hennepin Healthcare, Pharmacy, Minneapolis, USA

*Critical Care* 2023, **27(S1)**: P105

**Introduction:** Sixteen to thirty-one percent of medical intensive care unit (MICU) patients have alcohol use disorder [1]. Benzodiazepines are first line therapy for alcohol withdrawal syndrome (AWS); however, phenobarbital (PHB) is emerging as an alternative therapy. PHB has demonstrated safety and comparative efficacy in reduction of withdrawal symptoms [2,3]. This study evaluated the safety and efficacy of PHB for the treatment of severe AWS in the MICU.

**Methods:** This retrospective, single-center, cohort study included patients ≥ 18 years of age admitted January 1, 2021, through July 31, 2022, to the MICU service for management of AWS. Patients who received diazepam monotherapy were compared to patients who received ≥ 1 dose of PHB ± diazepam for AWS. The primary outcome was to assess the difference in rate of intubation within 72 h of PHB administration compared to diazepam.

**Results:** A total of 67 PHB encounters and 98 diazepam monotherapy encounters were included, which yielded intubation rates of 55% and 41.4% within the 72-h timeframe, respectively. The most frequent indication for intubation within the 72-h timeframe was for airway protection which occurred in 54.6% of PHB intubations and 70.8% of diazepam monotherapy intubations. The mean PHB dose administered was 479.98 mg/dose (SD ± 223.3) or 6.48 mg/kg/dose (SD ± 2.97) based on ideal body weight. Mean diazepam dose administered over 72 h was 97.25 mg (SD ± 101.54) in the diazepam monotherapy group.

**Conclusions:** The use of PHB for control of AWS was associated with a higher rate of intubation compared to the diazepam group.


**References**
Dixit D et al. Pharmacotherapy. 2016;36:797–822.Oks M et al. J Intensive Care Med. 2020;35:844–850.Ammar MA et al. Ann Pharmacotherapy. 2021;55:294–302.


## P106

### Valproic acid for agitation: retrospective analysis of psychiatric consults

#### M Perreault^1^, N Adessky^2^, A Regazzoni^2^, G White^2^, FW Chen^2^, L Tourian^3^, M Chagnon^4^, A Gursahaney^5^, M Alharbi^3^, D Williamson^6^

##### ^1^The Montreal General Hospital, Pharmacy Suite C1-200, Montreal, Canada, ^2^McGill University Health Center, Dept of Pharmacy, Montreal, Canada, ^3^McGill University Health Center, Dept of Psychiatry, Montreal, Canada, ^4^Université de Montreal, Dept of Mathematics and statistics, Montreal, Canada, ^5^McGill University Health Center, Dept of Critical Care, Montreal, Canada, ^6^Université de Montreal, Faculté de Pharmacie, Montreal, Canada

*Critical Care* 2023, **27(S1)**: P106

**Introduction:** Agitation is a common problem in the intensive care unit (ICU). Treatment options are based on clinical experience and sparse quality literature. Our aim was to evaluate the effect of valproic acid (VPA) as adjuvant treatment for agitation in the ICU as well as identify independent predictors of response.

**Methods:** A retrospective single center observational study evaluated adult ICU patients for whom a psychiatric consultation was requested for agitation (defined as a RASS ≥ 2) from 2015 to 2021. A descriptive analysis of the proportion of agitated patients per day of follow-up, incidence of agitation-related-events (ARE) defined as device removal, as well as evolution of co-medications use over time are presented. A logistic regression model was used to assess predictors of VPA response for agitation and GEE models were used to evaluate the independent effect of VPA as an adjuvant therapy for agitation.

**Results:** 175 patients met inclusion criteria with 78 receiving VPA and 97 as a control group. Reason for admission in the VPA group was mostly TBI (62.8%) and causes of agitation were multifactorial. 97.4% were mechanically ventilated. The percentage of agitation-free patients was 6.5% on Day 1, 14.1% on Day 3 and 39.5% on Day 7 where Day 1 = day of VPA initiation.The percentage of patients with at least one ARE was highest on Day 1 with 14.3% and lowest on Day 4 with 6.76%. Multivariate regression model for variables identified female sex (n = 12) as predictor of response on Day 7 (OR 6.10 [1.18–31.64]). A significant effect of VPA on agitation was not observed when controlling for confounding variables.

**Conclusions:** Although VPA was associated with a decrease in agitation and ARE in the 7 days following initiation, its effect when controlling for confouding variables did not yield significant results. Prospective placebo-controlled studies are warranted to assess the natural evolution of agitation and the true impact of VPA on agitation and ARE in this population.

## P107

### Valproic acid for the treatment of ICU agitation and delirium

#### RM Nelson, TS Lam, KA Considine, JT Jancik

##### Hennepin Healthcare, Pharmacy, Minneapolis, USA

*Critical Care* 2023, **27(S1)**: P107

**Introduction:** Agitation and delirium are common in the intensive care setting. Rates of agitation and delirium in ICU patients have been reported up to 70% and 89% respectively [1,2]. Previous studies have demonstrated that use of valproic acid (VPA) has been associated with reductions in agitation and delirium in the ICU [3–5]. The purpose of this study was to assess the impact of VPA on resolution of ICU agitation and delirium when prescribed at comparable or higher doses than previously published.

**Methods:** This single-center retrospective analysis was conducted in adult patients admitted between 01 Jun 2018 and 31 May 2022 who were prescribed VPA for agitation or delirium in the ICU. Agitation was defined as a RASS greater than or equal to 2 for at least 2 occurrences per calendar day. Delirium was defined as a positive CAM-ICU for at least 2 occurrences per calendar day. The primary objective was time to resolution of agitation or delirium from VPA initiation.

**Results:** The analysis included 177 patient encounters. Agitation or delirium was present in 61.0% and 94.4% of patients respectively. Agitation resolved in 85.2% of patients with median time to resolution of 3 days (IQR 4). Delirium resolved in 75.4% of patients with median time to resolution of 3 days (IQR 4.5). Agitation or delirium recurred in 53.2% and 61.9% of patients respectively during the same ICU stay. The median starting dose of VPA was 22.2 mg/kg/day (IQR 11.4).

**Conclusions:** VPA at doses greater than 20 mg/kg/day may be useful to aid in resolution of agitation or delirium in the ICU.


**References**
Aubanel S et al. Anaesth Crit Care Pain Med. 2020;39:639–646.Reade MC et al. N Engl J Med. 2014;370:444–454.Gagnon DJ et al. J Crit Care. 2017;37:119–125.Crowley KE et al. Clin Ther. 2020;42:e65–e73.Quinn NJ et al. Ann Pharmacother. 2021;55:311–317.


## P108

### Risk factors for cognitive impairment in critically ill surgical patients

#### KJ Jeong^1^, YJ Jung^1^, SB An^2^, HJ Lee^2^, SK Hong^2^

##### ^1^Asan Medical Center, Nursing, Seoul, South Korea, ^2^Asan Medical Center, Division of Acute Care Surgery, Department of Surgery, Seoul, South Korea

*Critical Care* 2023, **27(S1)**: P108

**Introduction:** As the ICU survival rate increases, the number of patients experiencing long-term cognitive impairment is increasing. The aim of this study is to evaluate cognitive impairments at the time of discharge from the surgical intensive care unit and identify the incidence and risk factors of cognitive impairments.

**Methods:** A prospective study was conducted from July 1, 2021, to May 31, 2022. The participants were adult patients who stayed in the surgical intensive care unit for more than 48 h. At the time of discharge from the ICU, cognitive function was evaluated with with the Mini-Mental State Examination (MMSE) and the risk factors (general, disease, treatment-related characteristics) of cognitive impairment were analyzed.

**Results:** In this study, the prevalence of cognitive impairments was 41.8% (64/153). As a result of analyzing the factors related to the occurrence of cognitive impairments, age, education level, living with family, and history of cerebrovascular diseases, CCI, delirium, use of sedatives, length of stay in ICU, use of insulin were different between groups (Table 1). According to multivariate regression, lower education level, history of cerebrovascular diseases, prolonged delirium, prolonged hyperglycemia, and absence of caregiver were identified as a predictive factors of cognitive impairment at the time if discharge from ICU.

**Conclusions:** The cognitive impairment was relatively high. The correction of modifiable risk factors for cognitive impairment such as prevention of delirium and hyperglycemia can attenuate cognitive dysfunction. It is necessary to develop and apply an individual and step-by-step cognitive rehabilitation program considering the characteristics of the patient by assessing the level of education and the history of cerebrovascular diseases such as stroke.

**Table Tabad:** **Table 1 (abstract P108)**. Risk factors of cognitive impairment when discharging in SICU

Variables	OR	95% CI	*p*
Education(< high school)	5.166	2.20–12.098	< .001*
Living with family	0.369	0.460–0.853	.020*
History of stroke	5.553	1.759–17.532	.003*
Presence of delirium	4.759	1.668–13.582	.004*
Use of insulin	2.586	1.101–6.679	.042*
Duration of ICU stay days	1.020	0.979–1.063	.337

## P109

### Greater serum concentration of IL-6 at hospital admission is associated with increased risk of developing delirium during hospitalization

#### T Bassi^1^, E Rohrs^2^, M Nicholas^2^, S Reynolds^2^

##### ^1^Lungpacer Medical Inc., Burnaby, Canada, ^2^Royal Columbian Hospital, New Westminster, Canada

*Critical Care* 2023, **27(S1)**: P109

**Introduction:** Delirium is a prevalent syndrome in hospitalized patients. Chronic inflammation has been associated with an increased risk of developing delirium during hospitalization. The focus of our study was to investigate existing literature to determine whether, at hospital admission, serum concentration of a biomarker for systemic inflammation, interleukin (IL)-6, was associated with the likelihood of developing delirium during hospitalization. We conducted a systematic review and meta-analysis to investigate that question.

**Methods:** Our meta-analysis followed the Preferred Reporting Items for Systematic Review and Meta-Analysis (PRISMA) protocol. Independent extraction with multiple reviewers' consensus was used to determine the studies included. The weight and heterogeneity of the manuscripts were calculated using inverse covariance with a random-effects model. The inclusion criteria were articles that investigated links between serum concentration of IL-6 at hospital admission and delirium during hospitalization.

**Results:** After excluding duplicates, sixteen studies that investigated the role of IL-6 serum concentration at hospital admission were included. Our search found evidence that patients who developed delirium during hospitalization had, at hospital admission, a mean serum concentration of IL-6 significantly greater than patients who did not develop delirium during hospitalization. The mean difference in IL-6 serum concentration was independent of other confounding variables such as the patient’s severity of illness. The mean difference in serum concentration of IL-6 at hospital admission between patients who developed delirium and those who did not was 24.05 pg/ml, *p* < 0.00001.

**Conclusions:** Our findings indicate that a greater serum concentration of IL-6 at hospital admission is associated with an increased risk of developing delirium during hospitalization.

**Figure Figaq:**
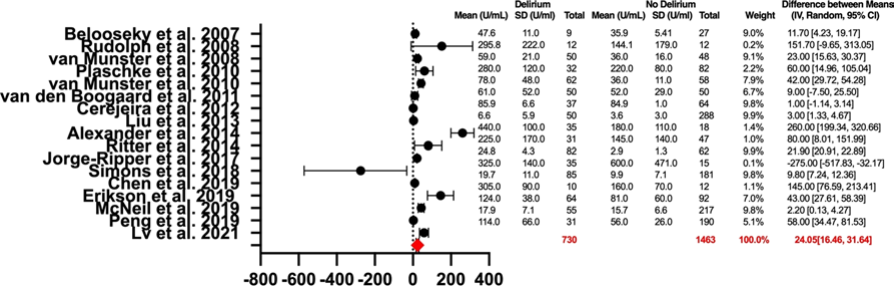
**Fig. 1 (abstract P109)** Forest plot showing mean difference in IL-6 serum concentrations at hospital admission between patients who develop delirium and who do not develop delirium during hospitalization

## P110

### Thiamine supplementation and delirium occurrence in the intensive care unit

#### MA Mumin^1^, C McKenzie^2^, L Aitken^3^, D Hadfield^4^, F Hanks^5^, J Yick^6^, V Page^7^, B Blackwood^8^, D McAuley^8^, P Spronk^9^

##### ^1^Pharmacy, King’s College Hospital NHS Foundation Trust, Pharmacy, London, UK, ^2^Perioperative and Critical Care Theme, Southampton NIHR Biomedical Research Centre, University Hospital Southampton, Southampton, UK, ^3^School of Health and Psychological Sciences, City, University of London, London, UK, ^4^Critical Care, King’s College Hospital NHS Foundation Trust, London, UK, ^5^Pharmacy, Guy’s and St. Thomas’ NHS Foundation Trust, London, UK, ^6^Pharmacy, King’s College Hospital NHS Foundation Trust, London, UK, ^7^Watford General Hospital, Watford, UK, ^8^Wellcome-Wolfson Institute for Experimental Medicine, Queen’s University Belfast, Belfast, UK, ^9^Gelre Ziekenhuizen Apeldoorn, Apeldoorn, Netherlands

*Critical Care* 2023, **27(S1)**: P110

**Introduction:** We hypothesized that routine intravenous (IV) thiamine supplementation in intensive care unit (ICU) will reduce delirium occurrence. Thiamine di-phosphate (TDP), the active form of thiamine, is an essential cofactor in glucose metabolism, glutamate transformation and acetylcholinesterase activity; all reported in delirium occurrence [1].

**Methods:** An anonymized, approved patient dataset was obtained from consecutive ICU admissions over two periods in Gelre Hospitals, Netherlands; October 2014–October 2016 (period 1) and April 2017–April 2019 (period 2). Routine scheduled prescription of IV thiamine, 300 to 600 mg within 72 h of admission, followed by 100 mg daily for 5 days, began in October 2016 after reporting of low whole blood thiamine [2]. Delirium was defined as 1 or more positive confusion assessment method-intensive care unit (CAM-ICU) score in 24 h. PREDELIRIC scores were calculated as a measure of prediction of delirium [3].

**Results:** There was a total of 3074 ICU admissions recorded. In period one, 111 of 1508 (7%) received IV thiamine, compared to 937 of 1566 (60%) patients during period 2 (Table 1). Data eligible for PREDELIRIC risk assessment was available for only 575 (38%) and 549 (35%) patients in period 1 and 2 respectively. Our analysis reported a reduction in delirium incidence during period 2 (0.16), compared to period 1 (0.20); OR: 0.78 [95% CI 0.65–0.94; *p* = 0.009]. There was no change in the mean PREDELIRIC score between period 1 and period 2, 0.30 (± 0.11).

**Conclusions:** IV thiamine administration may reduce delirium occurrence in the ICU; although it did not appear to confer benefit in those at greater risk of delirium. Limitations include low adherence to IV thiamine regime in period 2, and observational and retrospective data. Future work should focus on mechanism of thiamine in delirium and randomised control efficacy trials.


**References**
Kealy J et al. JNeurosci 2020;40:5681–5696.van Snippenburg W et al. J Iintensive Care Med 2017;32:559–564.van den Boogaard M et al. BMJ 2012;344:e420.

**Table Tabae:** **Table 1 (abstract 110)**. Overall delirium incidence

	Number of patients receiving thiamine	PREDELIRIC score (mean ± SD)	Delirium incidence (mean)	OR (95% CI)
Period 1 (Oct’14–16)	111 (7%)	0.30 ± 0.11	0.20	
Period 2 (Apr’17–19)	937 (60%)	0.30 ± 0.11	0.16	0.78 (0.65–0.94)

## P111

### Differences in delirium presentation in non-intubated COVID-19 and non-COVID-19 patients in the ICU

#### MA Xanthoudaki, A Valsamaki, D Papadopoulos, P Katsiafylloudis, A Papadogoulas, K Oikonomou, E Papapostolou, D Tsimpida, D Xenou, P Papamichalis

##### General Hospital of Larissa, Intensive Care Unit, Larissa, Greece

*Critical Care* 2023, **27(S1)**: P111

**Introduction:** Delirium in intensive care unit (ICU) patients is a common disorder which is characterized by non-specific brain dysfuncion and is associated with increased mortality [1]. Coronavirus disease 2019 (COVID-19), caused by the novel coronavirus SARS-COV-2 is associated with increased delirium rates through various mechanisms including hypoxaemia, circulation of inflammatory mediators and direct central nervous system damage [2]. Due to life-threatening respiratory manifestations in COVID-19 patients delirium is often underestimated. Aim of the study was to record the pharmacologic treatment of delirium in ICU patients treated with non-invasive ventilation and the differences in delirium treatment between COVID-19 and non-COVID-19 patients.

**Methods:** Consecutive patients with respiratory failure were retrospectively included. All patients were hospitalized in the ICU and non-invasive ventilation was applied. Patient characteristics, length of ICU stay and medication were recorded.

**Results:** Totally 64 patients were included (33 patients with COVID-19). Pharmacologic management of delirium included administration of remifentanyl, dexmedetomidine, propofol and combinations of the above. COVID-19 patients were younger (61 vs 70.4 years, *p* = 0.02), had longer ICU hospitalization (*p* < 0.001) and needed longer intravenous medication administration (*p* = 0.001). Additionally, the proportion of patients with COVID-19 treated with intravenous medication due to delirium was higher than non-COVID-19 patients (81.8% vs 48.4%, *p* = 0.008). COVID-19 seemed to have worse outcomes than non-COVID-19 patients and this difference tended to be statistically significant (*p* = 0.09).

**Conclusions:** Delirium is quite common and requires aggressive treatment among COVID-19 patients, despite being younger than non-COVID-19 patients. Physicians should be vigilant to assess and manage delirium to improve the long-term outcomes of COVID-19 patients.


**References**
Pisani MA et al.Arch Intern Med 2007;167:1629–1634.Kotfis K et al. Crit Care 2020;24:176.


## P112

### Pathogenesis of encephalopathy and delirium: neocortical neuronal membrane potential and action potential firing are changed by coronavirus-associated cytokines

#### SR Rajayer, SM Smith

##### OHSU, PACCM, Portland, USA

*Critical Care* 2023, **27(S1)**: **P112**

**Introduction:** Encephalopathy and delirium are common following coronavirus infection [1], and the associated neuroinflammation often results in long-term behavioral and cognitive impairment. Neurovirulent cytokines (NVC) are strongly implicated in the pathogenesis of coronavirus encephalopathy [2]. We hypothesized that characterizing the abnormal signaling in NVC exposed neurons will enable us to identify targets to treat encephalopathy and prevent its downstream effects.

**Methods:** We incubated primary mouse neocortical cultures in NVC known to be increased in coronavirus encephalopathy (TNF-α, IL-1β, IL-6, IL-12 and IL-15). Using whole-cell patch clamp methods, we tested how neuronal function was impacted by 22–28-h exposure to NVC.

**Results:** We found that NVC depolarized the resting membrane potential (RMP), reduced the firing threshold of neocortical neurons, and increased baseline spontaneous action potential (AP) firing. NVC altered the sensitivity (or input–output properties) of single neurons to changes in their microenvironment. Specifically, decreasing external Ca^2+^ and Mg^2+^ from physiological to low (1.1–0.2 mM) levels increased evoked AP firing in control, but not following exposure to NVC. AP firing threshold and spontaneous firing rates returned to control levels 1 h after NVC wash-out. However, the RMP and attenuated sensitivity of evoked APs to changes in the microenvironment remained persistently abnormal suggesting two distinct mechanisms were at play. Interestingly, hyperpolarizing the RMP reversed this altered response.

**Conclusions:** Sustained exposure to NVC reversibly depolarizes neocortical neuronal RMP, altering excitability and the ability of neurons to respond to microenvironment changes. By characterizing the pathogenesis of the underlying changes in neuronal function in our model of coronavirus encephalopathy we will identify intervenable drug targets.


**References**
Uginet M et al. J Med Virol 2021;93:4374–81.Li Y et al. J Virol 2004;78:3398–406.


## P113

### Long-term outcomes of delirium in critically ill surgical patients: a multicenter prospective cohort study

#### O Chaiwat^1^, P To-adithep^2^, K Chittawatanarat^3^, S Morakul^4^

##### ^1^Faculty of Medicine Siriraj Hospital, Anesthesiology, Bangkok, Thailand, ^2^Faculty of Medicine Siriraj Hospital, Department of Anesthesiology, Bangkok, Thailand, ^3^Department of Surgery, Faculty of Medicine Chiang Mai Hospital, Chiang Mai University, Chiang Mai, Thailand, ^4^Department of Anesthesiology, Faculty of Medicine Ramathibodi Hospital, Bangkok, Thailand

*Critical Care* 2023, **27(S1)**: P113

**Introduction:** Postoperative delirium in critically ill surgical patients was associates with short and long term worsening outcomes. There has been limited data regarding long-term outcomes for delirious patients in Thailand. The aims of this study were to identify the mortality rates and dependency rate (functional outcomes) of delirious patientsat 12 months after surgical intensive care unit (SICU) admission and to determine the independent risk factors of 12-month mortality and dependency rate in a cohort of SICU patients.

**Methods:** A prospective, multi-center study were conducted in 3 university-based hospitals. Critically-ill surgical patients who were admitted to surgical intensive care unit and followed up at 12 months after ICU admission were enrolled. The 12-month mortality was retrieved from medical records or from the Bureau of Registration administration. We assessed dependency state by basic activity of daily living (ADL) and instrumental ADL by telephone interview.

**Results:** A total of 630 eligible patients were recruited. 170 patients (27%) had postoperative delirium. The overall 12-month mortality rate in this cohort was 25.2%. Delirium group showed significantly higher mortality rates than non-delirium group at 12-months after ICU admission (44.1% vs 18.3%, *p* 0.001). Independent risk factors of 12-month mortality were ages, diabetes mellitus, preoperative dementia, high SOFA score and postoperative delirium. The overall dependency rate was 52%. Independent risk factors of dependency rate at 12-months included ages ≥ 75 years, cardiac diseases, duration of mechanical ventilation and postoperative delirium.

**Conclusions:** Postoperative delirium in SICU demonstrated higher mortality rates at 12-months after SICU admission than non-delirium group. Postoperative delirium was an independent risk factor of death at 12-months after SICU admission and becoming dependence after discharge from hospitals.

## P114

### Study on the value of neutrophil to lymphocyte ratio and neutrophil to prealbumin ratio in prognosis and early warning of organ injury in patients with sepsis

#### J Liu, Q He, C Li, Y Pan, Y Wu

##### Department of Critical Care Medicine, The Affiliated Suzhou Hospital of Nanjing Medical University, Suzhou Municipal Hospital, Suzhou Clinical Medical Center of Critical Care Medicine, Suzhou, China

*Critical Care* 2023, **27(S1)**: P114

**Introduction:** To explore the early warning value of neutrophil to lymphocyte ratio (NLR) and neutrophil to prealbumin ratio (NPRI) in hospital death and organ dysfunction of septic patients.

**Methods:** The clinical data of 175 patients with sepsis were analyzed retrospectively. Patients were divided into survival group and death group according to whether they died in hospital. According to the scores of each system in SOFA score, they were divided into positive group and negative group of organ dysfunction. Then analyze and compare the indexes of each group and their value in predicting patients' death and organ dysfunction.

**Results:** The scores of NLR, NPRI, SOFA and the number of organ injuries in the death group were higher than those in the survival group (*p* < 0.05), while the prealbumin and hospitalization time were lower than those in the survival group (*p* < 0.05). Univariate binary logstic regression analysis showed that NLR [OR = 1.035, 95% CI (1.015–1.055), *p* = 0.001], SOFA [OR = 1.198, 95% CI (1.040–1.380), *p* = 0.012], *p* = 0.025], the number of organ injuries [OR = 1.619, 95% CI (1.031–2.541), *p* = 0.036] were the risk factors for the hospital death of sepsis patients, and prealbumin [OR = 0.992, 95% CI (0.985–0.998). Multivariate binary Logstic regression analysis showed that NLR [OR = 1.037, 95% CI (1.016–1.059), *p* = 0.000] and SOFA [OR = 1.236, 95% CI (1.024–1.491), *p* = 0.027] were predictive sepsis patients ROC curve analysis showed that NLR [AUC = 0.706, 95% CI (0.524–0.887), *p* = 0.005], NPRI [AUC = 0.7586,95% CI (0.621–0.895), *p* = 0.001] *p* = 0.004] and the number of organ injuries [AUC = 0.655, 95% CI (0.610–0.818), *p* = 0.043] are all predictive values for the hospital death of sepsis patients.

**Conclusions:** NLR and SOFA scores are independent risk factors for nosocomial death in patients with sepsis, and prealbumin is an independent protective factor for nosocomial death in patients with sepsis. NLR, SOFA score, NPRI and the number of organ injuries can better predict the hospital death of sepsis.

## P115

### Peripheral dendritic cells as early biomarkers to predict persistent organ failure in patients with predicted severe acute pancreatitis

#### S Yang, J Liu

##### Department of Critical Care Medicine, The Affiliated Suzhou Hospital of Nanjing Medical University, Suzhou Municipal Hospital, Suzhou Clinical Medical Center of Critical Care Medicine, Suzhou, China

*Critical Care* 2023, **27(S1)**: P115

**Introduction:** To investigate whether circulating dendritic cells (DC) and its subtypes were able to serve as early biomarkers in predicting persistent organ failure (POF) in predicted severe acute pancreatitis (pSAP).

**Methods:** We measured the proportions of dendritic cells (DC) and its subtypes. We conducted a prospective study to study whether dendritic cells (DC) and its subtypes can predict persistent organ failure (POF) in patients with predicted SAP. A total of 59 predicted SAP patients who were admitted within 72 h after symptom onset. In addition, 12 age-matched healthy normal controls served as controls (Con). The fasting peripheral venous blood samples were taken in the group 24 h. Flow cytometry (FCM) assay in peripheral blood dendritic cells (DC) and myeloid dendritic cells (mDC) and plasmacytoid dendritic cells (pDC).

**Results:** A total of 12 healthy volunteers and 59 AP patients were enrolled in the study. AP patients were divided into TOF and POF groups, and there was no significant difference between the two groups. The changes of DC, mDC and pDC in peripheral blood of patients with AP: compared with the control group, the peripheral blood DC and mDC of the AP group decreased significantly (*p* = 0.000). DC and mDC in peripheral blood of patients with AP were negatively correlated with the severity of the disease (APACHE, II, Ranson scores and revised Marshall scores) (*p* < 0.05). DC prediction in patients with early AP prediction of POF AUC 0.808 (0.667–0.949).

**Conclusions:** The percentages of DC, mDC and pDC in peripheral blood mononuclear cells of patients with AP are significantly lower. The expression of DC, mDC and pDC in peripheral blood monocytes correlated with the severity of the disease. The quantity of DC in peripheral blood mononuclear cells of patients with AP may be a good index to predict the occurrence of POF.

## P116

### Real-time, leukocyte-related, four times validated, patient data partitioning

#### A Rivas^1^, C Libertin^2^, P Satashia^3^, S Isha^3^, A Jenkins^3^, A Hanson^3^, D Sanghavi^3^, P Moreno Franco^3^

##### ^1^Center for Global Health, School of Medicine, University of New Mexico, Albuquerque, USA, ^2^Mayo Clinic, Infectious Disease, Jacksonville, USA, ^3^Mayo Clinic, Critical Care Medicine, Jacksonville, USA

*Critical Care* 2023, **27(S1)**: P116

**Introduction:** Indicators that assess relationships among leukocytes may inform more and/or earlier than those measured in isolation.

**Methods:** Blood leukocyte differential counts collected from 101 Mayo Clinic COVID-19 patients were related to later outcomes following two approaches: (i) as unstructured data (e.g., lymphocyte percentages) and (ii) as data structures that assess intercellular interactions. Analyzing the same primary data, it was asked whether information contents differed among methods and/or when two sets of structured indicators are used.

**Results:** While unstructured data did not distinguish survivors from non-survivors (Fig. 1, rectangle A), one data structure (here identified with letters expressed in italics) exhibited one perpendicular inflection that differentiated two patient groups (B). Two survivor-related observations were also distinguished from the remaining data points (B). A second data structure also revealed a single line of observations and a perpendicular data inflection (C), while more (four) patient groups were identified (D). Four validations were conducted: (i) increasing mortality levels among contiguous data subsets (0, 7.1, 16.2, or 44.4%) suggested *construct validity* (D); (ii) *internal validity* was indicated because 22 of the 45 survivors detected by the first data structure were also captured by the second one; (iii) the analysis of patients that differed in address, co-morbidities and other aspects supported *external validity*; and (iv) quasi non-overlapping data intervals predicted *statistical validity* (E, F). The structured approach also uncovered new and/or dissimilar information: different leukocyte-related ratios explained the clusters identified in these analyses (E, F).

**Conclusions:** Structured data may yield more information than methods that do not assess multicellular interactions. Possible applications include daily, longitudinal, and personalized analysis of hospital data.

**Figure Figar:**
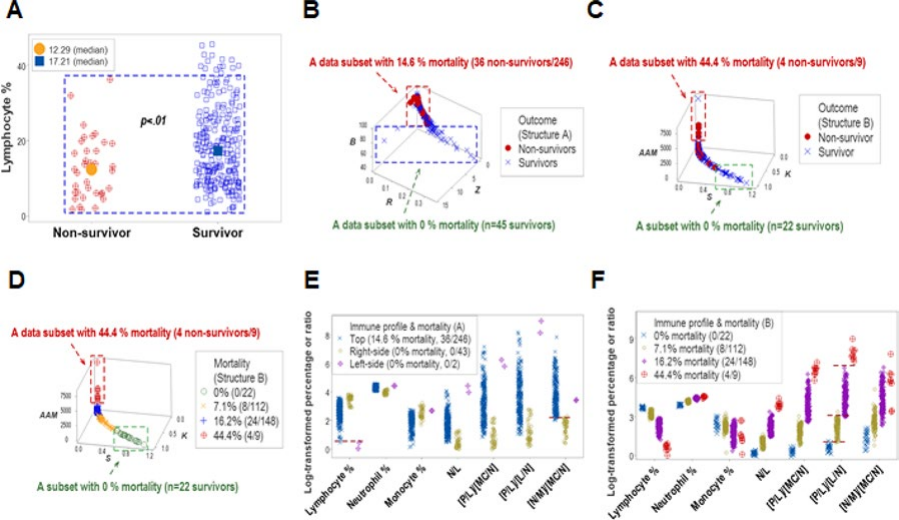
**Fig. 1 (abstract P116)**. Data partitioning validated by qausi non-overlapping immune profiles

## P117

### Associations between red cell distribution width and outcomes of critically ill patients ≥ 90 years

#### P Theile, J Müller, R Daniels, S Kluge, K Roedl

##### University Medical Centre Hamburg-Eppendorf, Department of Intensive Care Medicine, Hamburg, Germany

*Critical Care* 2023, **27(S1)**: P117

**Introduction:** Different clinical conditions are associated with changes in red cell distribution width (RDW). High levels of RDW(> 14.5%) were described as predictive marker for unfavorable outcome/mortality in critically ill patients. There is a lack of data in the very elderly critically ill, why we aimed to investigate the association of RDW with outcome in critically ill patients ≥ 90 years.

**Methods:** Retrospective analysis of all consecutive critically ill patients ≥ 90 years of the Medical University Centre Hamburg-Eppendorf (Hamburg, Germany) with RDW on admission. Clinical course and laboratory were analysed from all patients. High RDW was defined as (> 14,5%). We clinically assessed factors associated with mortality. Univariable and multivariable cox regression analysis was performed to determine the prognostic impact of RDW on 28-day mortality.

**Results:** We could identify 863 critically ill patients ≥ 90 years with valid RDW values and complete data. 32% (n = 275) died within 28 days and 68% (n = 579) survived 28d. Median RDW levels on ICU admission were significantly higher in non-survivors compared with survivors (15.6% vs. 14.8%, *p* < 0.001). The proportion of high RDW (> 14.5%) was significantly higher in non-survivors(73% vs. 57%, *p* < 0.001). Patients with low RDW presented with a lower Charlson Comorbidity index (*p* = 0.014). SAPS II on admission was lower in patients in the low RDW (35 vs. 38 points, *p* < 0.001). 32% (n = 104) in the low and 35% (n = 190) in the high RDW group were mechanically ventilated (*p* = 0.273). Use of vasopressors (35% vs. 49%, *p* < 0.001) and RRT (1% vs. 5%,*p* = 0.007) was significantly higher in the high RDW group. Cox regression analysis demonstrated that RDW was significantly associated with 28d mortality [crude HR 1.768, 95% CI (1.355–2.305); *p* < 0.001; adjusted HR 1.372, 95% CI (1.045–1.802); *p* = 0.023].

**Conclusions:** High RDW was significantly associated with mortality in critically ill patients ≥ 90 years. RDW can be used as simple parameter for risk stratification in very elderly critically ill patients.

## P118

### Sepsis outcomes: is there a place for leukocyte cell population data?

#### S Šundalić^1^, I Košuta^1^, R Radonić^1^, I Baršić Lapić^2^, I Rako^2^, D Rogić^2^, A Vujaklija Brajković^1^

##### ^1^University Hospital Centre Zagreb, Department of Internal Medicine, Zagreb, Croatia, ^2^University Hospital Centre Zagreb, Department of Laboratory Diagnostics, Zagreb, Croatia

*Critical Care* 2023, **27(S1)**: P118

**Introduction:** Hematological analyzers rapidly provide information on the morphological and functional characteristics of leukocytes, known as leukocyte cell population data (CPD) which is automatically available without the need to collect additional samples [1]. Previous research mostly described the role of CPD in sepsis diagnosis with little emphasis on prognosis. We evaluated the utility of CPD on clinical outcomes.

**Methods:** This prospective observational study was conducted in a medical ICU of a tertiary level hospital. Complete blood count, CPD data and biochemistry were collected in antibiotic-naive sepsis cases at admission and repeated on the third day. The Sysmex XN-3000 analyzer was used for leukocyte CPD analysis. The association of procalcitonin (PCT), interleukin-6 (IL-6) and CPD on survival, need for mechanical ventilation (MV) and development of acute kidney injury (AKI) was evaluated. AKI was defined by the Kidney Disease Improving Global Outcomes (KDIGO) Criteria. Patients with leukopenia were excluded (< 4.00 × 10^9^/l white blood cells).

**Results:** Of the 98 cases, 50 were male, and 44 had hemoculture-positive sepsis. The comorbidities included arterial hypertension (n = 55, 56.1%), diabetes (n = 32, 32.7%) and nonhematologic malignancies (n = 24, 24.5%) (Table 1). Binary logistic regression revealed significant association of IL-6 (*p* = 0.04, OR 1.01, 95% CI 1.001–1.021) and the following leukocyte CPD data on 3rd day: cytoplasmic granulation of neutrophils (NEUT-GI) (*p* = 0.02, OR 0.85, 95% CI 0.735–0.974), and reactivity of neutrophils (NEUT-RI) (*p* = 0.04, OR 1.15, 95% CI 1.008–1.321) with AKI development. Leukocyte CPD and PCT were not found to be associated neither with survival nor need for MV.

**Conclusions:** Leukocyte CPD parameters such as NEUT-GI and NEUT-RI could be used for early detection of patients prone to developing AKI.


**Reference**
Urrechaga E. Ann Transl Med. 2020;8:953–953.

**Table Tabaf:** **Table 1 (abstract P118)**. Descriptive data at intensive care unit admission presented as median value and interquartile range

	Reference range	Median ± IQR	Min	Max
SOFA score		7 ± 6	1	17
L (× 109/l)	3.4–9.7	15.95 ± 13.8	5.2	35.4
CRP (mg/l)	< 5	200.45 ± 152.3	75.0	388.9
PCT (mcg/l)	< 0.25	1.98 ± 33.73	0.21	51.42
IL-6 (pg/ml)	< 7	158.75 ± 859.38	12.90	1509
NEUT-GI (SI)	142.8–159.3	153.45 ± 6.9	144.1	157.6
NEUT-RI (FI)	39.8–51.0	60.45 ± 12.7	49.2	67.3

## P119

### Assessment of a rapid cellular host response test in predicting sepsis in a subpopulation of patients who are administered steroids

#### HE Bunch^1^, M Sorrells^2^, R Sheybani^3^, H Tse^3^, A Shah^3^, CB Thomas^1^, HR O’Neal^1^

##### ^1^LSU Health Sciences Center/Our Lady 0f the Lake Regional Medical Center, Baton Rouge, USA, ^2^Cytovale LLC, Medical Affairs, San Francisco, USA, ^3^Cytovale LLC, San Francisco, USA

*Critical Care* 2023, **27(S1)**: P119

**Introduction:** Sepsis is defined as host immune dysregulation in response to an infection causing organ dysfunction. In patients taking steroids, identification of sepsis may be difficult due to the numerous effects of steroids on the immune system. The IntelliSep test is an investigational diagnostic that quantifies immune activation by measuring the biophysical properties of leukocytes from a blood sample in < 10 min. The test provides a single score, the IntelliSep Index (ISI), between 0.1 and 10.0, stratified into three interpretation bands of increasing disease severity risk: green, yellow, and red. Previously, we demonstrated the utility of the test on risk-stratifying patients with suspicion of sepsis. The purpose of this study was to analyze the performance of the ISI on a subpopulation of patients taking steroids.

**Methods:** Adult patients presenting to the ED with signs or suspicion of infection were prospectively enrolled at multiple US sites (Feb. 2016–Oct. 2021). EDTA-anticoagulated blood was assayed and patients were followed by retrospective chart review for outcome information. From this population, we compared ISI risk stratification to sepsis-3 criterion between a population of patients not receiving steroids (Steroid− group) to a smaller population of patients receiving steroids (Steroid+ group).

**Results:** 1108 patients were included in this study: 985 in the Steroid− group and 123 in the Steroid+ group. These two populations were stratified across interpretation bands with 49.0% and 55.3% Green Band, 25.7% and 23.6% Yellow Band, and 25.3% and 21.1% Red Band for the Steroid− and Steroid+ groups, respectively. The groups showed no differences in risk stratification across ISI interpretation bands and showed similar sepsis detection based on Sepsis-3 criterion (Fig. 1).

**Conclusions:** The ISI, a quantitative measure of immune dysregulation, may aid clinicians in the rapid stratification for risk of sepsis in patients with signs or suspicion of sepsis, independent of steroid use.

**Figure Figas:**
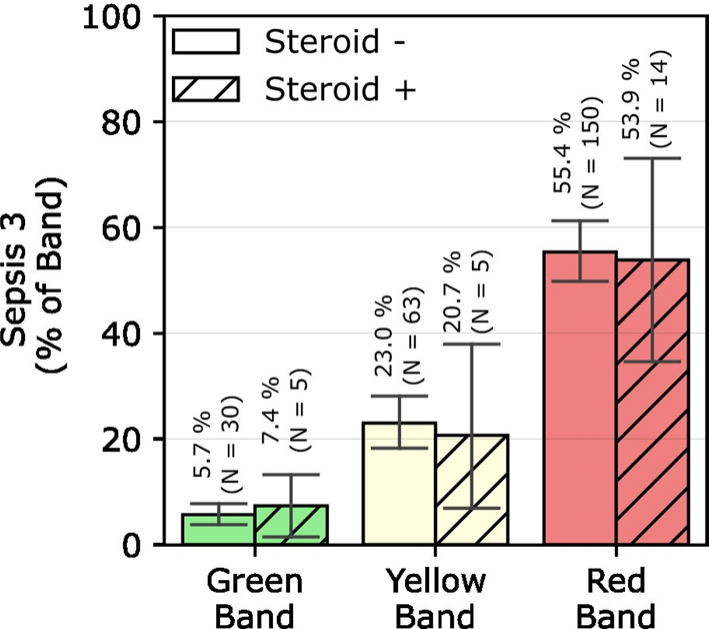
**Fig. 1 (abstract P119)**. Risk stratification of sepsis-3 across interpretation bands for Steroid− and Steroid+ groups (Steroid− : *p* < 10^E−9^ across all bands; Steroid+ : *p* = 0.22 Green-Yellow, *p* < 10^E−5^ Green–Red, *p* < 0.01; No significant differences were observed between Steroid+ and Steroid− groups at each band)

## P120

### An artificial neuronal network for the early prediction of sepsis in intensive care units

#### A Ogica, A Krannich, D Thiele, K Ulrich, A Grieser, C Storm

##### TCC GmbH, Hamburg, Germany

*Critical Care* 2023, **27(S1)**: P120

**Introduction:** Machine learning algorithms showed promising results in predicting sepsis and septic shock before onset. Current meta-analysis revealed a few models with good accuracy using more than 10 predictors [1]. We propose an artificial neural network (ANN) to predict the risk of sepsis a few hours in advance for the use in intensive care units (ICU).

**Methods:** Data were collected from an ICU of a tertiary care university centre. Septic patients and controls over 18 years old were included. To build a prognostic model only patients were selected with data being available at least 24 h before the onset of sepsis. Controls were matched accordingly. The vital signs were continuously monitored using high frequency automated measurements. At each timepoint of the measurement the median of the values from the last two hours was calculated to smoothen the measurement curves and account for single missing values. We used several clinically relevant vital signs combinations with less than 10 variables to select predictors for the ideal model. The area under curve (AUC), the accuracy and the precision were calculated for each model to evaluate the prediction performance.

**Results:** We identified about 5000 patients from the database matching the inclusion criteria. Of these 1.5% had sepsis identified by the qSOFA criteria. The best time point to predict sepsis was ten hours before the onset of sepsis. An ANN achieved a high prediction accuracy of over 80% ROC AUC beside lower performing models like logistic regression.

**Conclusions:** We identified a model capable to predict the risk of sepsis hours ahead before onset. Compared with other models with the same accuracy, ours contains less than 10 predictors. The use of machine learning algorithms will enhance the diagnostic accuracy thus reducing the time to receive appropriate therapy.


**Reference**
Fleuren et al. Intensive Care Med 2020;46:383–400.


## P121

### Effect of C-reactive protein (CRP)-guided corticosteroid use on outcomes in hospitalized patients with community acquired pneumonia (CAP): a target trial emulation

#### YE Odeyemi^1^, W Farah ^1^, A LeMahieu^2^, E Barreto^3^, H Yadav^1^, O Gajic^1^, P Schulte^2^

##### ^1^Mayo Clinic, Pulmonary and Critical Care Medicine, Rochester, USA, ^2^Mayo Clinic, Clinical Trials and Biostatistics, Rochester, USA, ^3^Mayo Clinic, Department of Pharmacy, Rochester, USA

*Critical Care* 2023, **27(S1)**: P121

**Introduction:** Existing evidence for adjuvant corticosteroid use in CAP is equivocal. CRP is an inflammatory marker that can be used to individually tailor steroid use in patients with CAP.

**Methods:** Using a retrospective cohort of hospitalized patients with CAP from 2009 to 2020, we conducted a target trial emulation comparing CRP-guided corticosteroid use to usual care. CRP-guided corticosteroid use arm specified measurement of CRP within 48 h of CAP admission and administration of corticosteroids for those with CRP ≥ 150 mg/dl but not those with CRP < 150 mg/dl. Remaining patients were classified as ‘usual care’. In eligible patients, inverse probability of treatment weighting methodology was used to balance potential confounders between CRP-guided and the usual care cohorts. The primary outcome was worst clinical status using the adapted WHO clinical progression scale. Secondary outcomes included need for non-invasive and invasive ventilation, and mortality. Weighted proportional odds logistic regression and logistic regression compared outcomes, using generalized estimating equations for robust variance estimates. Estimates reflect a per-protocol analysis of the specified target trial.

**Results:** In total, 184 patient received CRP-guided corticosteroid treatment and 4369 received usual care. Median age was 74, and the sample was 54% male and 94% white race. There was no difference in the primary outcome between the CRP-guided and usual-care groups. Specifically, in the unweighted analysis: OR 0.74 (95% CI 0.52–1.05; *p* = 0.093) and weighted analysis (OR 0.84 (95% CI 0.57–1.24, *p* = 0.38). There was no difference between groups in any of the secondary outcomes (Table 1).

**Conclusions:** In this target trial emulation, CRP-guided corticosteroid use in hospitalized patients with CAP was not associated with any differences in patient outcomes.

**Table Tabag:** **Table 1 (abstract P121)**. Clinical outcomes

	CRP-guided arm (n = 184)	Usual care arm (n = 4369)	Unweighted, OR (95% CI),* p* value	Weighted, OR (95% CI), *p* value
Worst condition, n (%)			0.74 (0.52, 1.05), 0.093	0.84 (0.57, 1.24), 0.381
Supplemental oxygen	148 (80%)	3278 (75%)		
Non-invasive mechanical ventilation	13 (7%)	453 (10%)		
Invasive mechanical ventilation	20 (11%)	484 (11%)		
Non-invasive mechanical ventilation or higher,	36 (20%)	1091 (25%)	0.83 (0.67, 1.03), 0.095	0.91 (0.71, 1.16), 0.452
Invasive mechanical ventilation or higher, n (%)	23 (13%)	638 (15%)	0.85 (0.63, 1.15), 0.301	1.00 (0.71, 1.41), 0.995
Mortality, n (%)	3 (12%)	154 (4%)	0.53 (0.34, 1.19), 0.126	0.60 (0.24, 1.45), 0.253

## P122

### Epigenetic assays identify leukocyte subpopulations that differentiate outcomes in a West African sepsis cohort

#### J Chenoweth^1^, J Brandsma^1^, S Krishnan^1^, J Michalska^2^, U Hoffmueller^2^, J Achenbach^2^, A Rosselló Chornet^2^, G Oduro^3^, N Adams^4^, D Clark^1^

##### ^1^Henry M. Jackson Foundation for the Advancement of Military Medicine, Inc., ACESO, Bethesda, USA, ^2^Precision for Medicine GmbH, Berlin, Germany, ^3^Komfo Anokye Teaching Hospital, Accident and Emergency Department, Kumasi, Ghana, ^4^Naval Medical Research Center, Directorate for Department of Defense Infectious Disease Research, Silver Spring, USA

*Critical Care* 2023, **27(S1)**: P122

**Introduction:** Cellular immunophenotyping has emerged as a powerful tool for sepsis endotyping and overcoming the problem of heterogeneity. However, technologies such as single‐cell RNA‐sequencing and cytometry by time‐of‐flight are not readily accessible in low resources settings. More recently developed epigenetic quantitative polymerase chain reaction methods enable immune cell characterization and counting by evaluating cell type-specific unmethylated DNA. This approach is suited for low-resource settings and low- and middle-income countries.

**Methods:** We conducted comprehensive epigenetic cellular immunophenotyping of innate and adaptive immune cells in sepsis patients enrolled in an observational study in Ghana. Fourteen epigenetic assays were used to analyze whole blood of 103 subjects upon admission to the emergency department with up to 5 serial samples (0, 1, 3 and 28 days, 6 and 12 months).

**Results:** Comparison between sepsis patients and healthy donors showed significant differences at enrolment, including decreased proportions of natural killer (NK) cells, and helper CD4 + and cytotoxic CD8 + T-cells, whereas neutrophil levels were elevated. Inspection of the data by mortality showed significantly decreased levels of T cells and CTLA4 + cells in the early non-survivor group (died within 28 days), but otherwise their immune cell profiles were comparable to the late non-survivor or survivor groups. Recovery from neutrophilia and lymphopenia initiated within 72 h in the sepsis survivors, but not in the early or late non-survivor groups. Finally, some cell types took up to a year to re-equilibrate back to normal levels, whereas others recover more quickly.

**Conclusions:** Taken together these results show that epigenetic immune cell profiling is a promising new tool for diagnostic and prognostic profiling of sepsis subjects in low resource settings. Future analysis will use these data and available medical data to identify clinically relevant endotypes.

## P123

### The role of IRS-1 gene polymorphism G972 to the increase of IGFBP-1 concentration and mortality risk in hyperglycemia severe sepsis patients

#### T Maskoen^1^, TH Achmad^2^, T Bisri^1^

##### ^1^Faculty of Medicine Universitas Padjadjaran, Anesthesiology and Intensive Therapy, Kota Bandung, Indonesia, ^2^Faculty of Medicine Universitas Padjadjaran, Biochemistry, Kota Bandung, Indonesia

*Critical Care* 2023, **27(S1)**: P123

**Introduction:** Hyperglycemia and insulin resistance are common in severe sepsis and associated with poor outcomes. The pathogenesis of both hyperglycemia and insulin resistance have not been clearly understood up till now. The advancement of molecular biology made the expert start investigating the possibility of sepsis pathogenesis that also come from genetic concept likes polymorphism [1].

**Methods:** This was a cohort molecular epidemiology study to investigate the role of polymorphism G972A gene IRS-1 to increase of IGFBP-1 concentration (as a marker of insulin resistance), and mortality risk in hyperglycemia severe sepsis patients. We divided into two groups, IGFBP-1 group and IRS-1 group. The differences of two varibles were analyzed by X^2^ and mortality risk was measured with relative risk. Significance of differences was determined by *p* < 0.05.

**Results:** In IGFBP-1 group that consist of 40 patients, the role of polymorphism to increase of IGFBP-1 concentration was analyzed, and in IRS-1 group that consist of 70 patients, the role of polymorphism to mortality was analyzed. In IGFBP-1 group, the role of IRS-1 gene polimorhism G972A to increase IGFBP-1 concentration was 27% (RR: 0.73; 95 CI: 0.1–4), and it was also found that mortality risk in polymorhism patients was 1.6 higher than in patients without polymorphism (RR: 1.6; 95% CI 1–4).

**Conclusions:** In this study, we found that in hyperglycemia severe sepsis patients, IRS-1 gene polymorphism G972A had a slight role to increase IGFBP-1 concentration, and it is also act as mortality risk factor. However further study needs to be conducted to strengthen the exact mechanism of pathogenesis as a whole.


**Reference**
Lin M, Albertson T. Crit Care Med 2004;32:569–79.


## P124

### Motor, a circular RNA-encoded micropeptide, regulates LPS tolerance of monocyte in sepsis

#### W Huang

##### Zhongda Hospital, School of Medicine, Southeast University, Department of Critical Care Medicine, Nanjing, China

*Critical Care* 2023, **27(S1)**: P124

**Introduction:** LPS tolerance, referred to an impaired capacity of monocyte to produce proinflammatory cytokines upon bacterial agonists, is a key feature of immune suppression in sepsis. However, the underlying mediators and molecular pathways remain poorly understood. Emerging studies have uncovered that translatable circular RNAs (circRNAs), as novel findings in the life sciences, perform unexpected function in many fundamental biological and pathological processes. This study aimed to explore the function and potential mechanism of circRNA-encoded peptide in regulating LPS-tolerance of monocyte during sepsis.

**Methods:** CircRNA microarray was used to identify the expression pattern of circRNAs in the blood of sepsis and septic shock patient. Coding potential of circRNAs was assessed by bioinformation analysis. Human monocytes (THP-1) and mice cecal ligation and puncture (CLP) model were used to evaluate the function of micropeptide encoded by circRNAs.

**Results:** We identified a novel circRNA-encoded micropeptide named “Motor” for the first time in sepsis. Motor was found lowly expressed in septic shock patients and lower Motor level correlated with poor prognosis, indicating that Motor plays a protective role in sepsis. Silencing Motor by siRNA dramatically suppressed chemokines and cytokines production globally in human monocyte THP1 cells upon LPS stimulation. Consistently, overexpression of Motor promoted pro-inflammatory cytokines releasing. Mechanically, transcriptome profiling indicated that Motor regulated NF-κB signaling. In an experimental model, exosome-based delivery of Motor enhanced the pro-inflammatory function of monocyte and relieved sepsis-associated organ damage and mortality.

**Conclusions:** Our results highlight the pro-inflammatory function of Motor in converting LPS tolerance of monocytes and the clinical potential of targeting Motor for monocyte-cell-directed immunotherapy in sepsis.

## P125

### Health economic impact of the use of calprotectin immunoassay for early detection of infections in intensive care patients

#### A Havelka^1^, M Lipcsey^2^, M Hultström^2^, A Larsson^3^

##### ^1^Gentian Diagnostics, Stockholm, Sweden, ^2^Uppsala University, Department of Surgical Sciences, Anesthesiology, Uppsala, Sweden, ^3^Akademiska University Hospital, Department of Medical Sciences, Clinical Chemistry, Uppsala, Sweden

*Critical Care* 2023, **27(S1)**: P125

**Introduction:** Early diagnosis of bacterial infections in critically ill patients is challenging, as the clinical manifestation is non-specific. Neutrophil activation is a major response to bacterial infection and calprotectin is an important marker for neutrophil mediated inflammation. Earlier studies have shown the ability of calprotectin to predict bacterial infections before onset of clinical symptoms. With early diagnosis of bacterial infections delayed treatment will be avoided as well as deterioration due to severe infections/sepsis.

**Methods:** A decision tree model is employed to estimate the cost-effectiveness of calprotectin analysis for early detection of bacterial infection and thus, the earlier start of antibiotic treatment compared to other diagnostic biomarkers. The comparators included in the analysis are white blood cell count, procalcitonin, C-reactive protein, and no testing. The analysis is based on patients admitted to an ICU in a Swedish hospital. The model allows for different diagnostic outcomes based on correctly and incorrectly diagnosis of bacterial infection and timing of antibiotic treatment: patient survival, length of stay in ICU and in general ward and total costs.

**Results:** The base-case results show that predictively measuring of calprotectin in an ICU saves total costs, reduces mean duration of in-patient care and mortality, compared with the comparators. Using GCAL® immunoassay reduces total costs by approximately 13,000–18,000 EUR per patient, overall mortality rate by 0.11, and mean length of stay in an ICU and general ward by 1.3–2 days and 6–8 days, respectively.

**Conclusions:** The base-case scenario identified GCAL® Calprotectin Immunoassay as cost-effective for a patient cohort presenting in a Swedish ICU. Compared to the comparators, GCAL® saves total costs, reduces the mean duration of in-patient care, and reduces in-hospital mortality in those patients. In the sensitivity analysis, when key model inputs are varied, GCAL® Calprotectin Immunoassay remains the dominant option.

## P126

### Serum catestatin is correlated with clinical parameters in non-critical COVID-19 patients

#### I Jerković^1^, V Kovacic^2^, T Ticinovic Kurir^3^

##### ^1^Department for Urgent and Intensive Medicine with Clinical Pharmacology and Toxicology, Internal Medicine Clinic, University Hospital Centre Split, University of Split School of Medicine, Split, Croatia, ^2^University Hospital Centre Split, University of Split School of Medicine, Department for Urgent and Intensive Medicine with Clinical Pharmacology and Toxicology, Internal Medicine Clinic, Split, Croatia, ^3^Department of Pathophysiology, University of Split School of Medicine, Split, Croatia

*Critical Care* 2023, **27(S1)**: P126

**Introduction:** Catestatin (CST) is a peptid with imunomodulatory, antiinflammatory, and antimicrobial activities. Acute coronavirus disease 2019 (COVID-19) caused by the SARS-CoV-2 virus can cause a systemic disease range unpredictably from mild flu-like disease to multiple organ failure. Despite many studies and scientific interest for COVID 19, there is lack of information regarding correlation between serum CST levels and clinical course od COVID 19. There are only few studies investigated CST plasma levels at COVID 19 patients, but mostly at ICU-patients, and those studies revealed that COVID 19 patients release significant amounts of CST in the plasma and CST predicts a poor COVID-19 outcome. In our work the aim was to demonstrate plasma CST levels and correlation with clinical outcome in a group of severe COVID 19 patients admitted in non-ICU department.

**Methods:** The subjects were patients admitted during second surge of COVID 19 in April and May 2020 in non-ICU unit for COVID 19 patients (high dependency unit) in Infectology department of University Hospital Split, Croatia. The reason of admission was pulmonary infiltrates and COVID 19 positivity confirmed with nucleic acid test. In study were included 32 subjects (25 females, 7 males) (Table 1). An enzyme-linked immunosorbent assay was used for serum CST levels assessment.

**Results:** We found significant positive correlation between serum CST levels and: C-reactive protein (r = 0.423, *p* = 0.008), D-dimers (r = 0.395, *p* = 0.013), hsTNT (high sensitivity troponin T) (r = 0.603, *p* < 0.001), proBNP (N-terminal-pro brain natriuretic peptide) (r = 0.569, *p* < 0.001), and hospitalisation days (r = 0.388, *p* = 0.014). There was significant difference between groups of participants with SOFA < 3 (n = 18) and SOFA > 3 (n = 14) in catestatin serum levels (7.25 ± 3.66 vs. 11.05 ± 9.52 ng/ml; *p* = 0.065).

**Conclusions:** This study confirmed that serum CST levels could have important role as clinical prognostic parameter among non-ICU COVID 19 patients.

**Table Tabai:** **Table 1 (abstract P126)**. Clinical features of participants

	Mean ± Std. Deviation	Minimum	Maximum
catestatin (ng/ml)	8.91 ± 7.00	3.68	42.55
age (years)	80.50 ± 9.86	58.00	97.00
SOFA score	3.16 ± 2.41	1.00	12.00
Sat O2 (%)	92.06 ± 6.89	64.00	98.00
creatinine (μmol/l)	105.12 ± 140.58	20.00	829.00
CRP (mg/l)	32.60 ± 40.76	0.60	195.70
hospitalisation (days)	30.59 ± 10.97	15.00	61.00

## P127

### Which biomarker(s) to predict worsening of SARS-CoV2 infection? An ancillary study from the COVIDeF cohort

#### M Cancella de Abreu^1^, J Ropers^2^, N Oueidat^3^, L Piéroni^4^, C Frere^5^, M Fontenay^6^, L Velly^1^, K Torelino^2^, P Hausfater^1^

##### ^1^Sorbonne Université APHP Hôpital Pitié-Salpêtrière, Emergency Department, Paris Cedex 13, France, ^2^Sorbonne Université APHP Hôpital Pitié-Salpêtrière, Unité de Recherche Clinique, Paris Cedex 13, France, ^3^Sorbonne Université APHP Hôpital Pitié-Salpêtrière, Biochemistry Laboratory, Paris Cedex 13, France, ^4^Sorbonne Université APHP Hôpital Tenon, Biochemistry Laboratory, Paris, France, ^5^Sorbonne Université APHP Hôpital Pitié-Salpêtrière, Hemtology Department, Paris Cedex 13, France, ^6^Institut Cochin, Hematology Laboratory, Paris, France

*Critical Care* 2023, **27(S1)**: P127

**Introduction:** COVID-19 has been responsible for millions of deaths and intensive care unit (ICU) admissions all over the world. Identifying the patients at risk of developing a severe form is crucial for an optimized orientation and allocation of resources. The main objective of our study was to identify among a selection of biomarkers, those predictive of short term worsening in COVID-19.

**Methods:** This is an ancillary study using clinical data and collected biobanking from the multicentric cohort study COVIDeF, which included prospectively from March 31th 2020 to March 30th 2021, patients admitted with a suspected Sars-CoV2 infection in the Assistance-Publique-Hôpitaux de Paris network, France. Patients with confirmed COVID-19 were divided in 2 groups: a severe (ICU admission or invasive or non-invasive ventilation or ARDS or death) and a control group (no worsening). The routine blood tests and following biomarkers: troponin, C Reactive Protein (CRP), procalcitonin, Mild-Regional pro-Adrenomedulin (MR-proADM), pro-endothelin, SuPAR, NT-proBNP, calprotectin, PF4, D-dimers, were measured in plasma or serum and compared between both groups using a conditional logistic regression.

**Results:** Among the 1040 first patients included in the COVIDEF cohort, we selected 512 patients having a blood sample drawn at admission before worsening, of which 60 secondarily worsened (severe group). The mean age was 59.5 (± 19.5) years and 50.2% were females. Among the biomarkers tested, three were independently associated with worsening: CRP (mg/l) OR 1.01 [IC 1.01–1.02], procalcitonin (ng/ml) OR 0.4428 [0.21–0.95] and MR-proADM (pg/ml) OR 3.012 [1.06–8.53].

**Conclusions:** Among a selection of biomarkers of interest, MR-proADM appears to best identify at admission COVID-19 patients at risk of worsening. Future interventional studies should test the efficacy and security of this biomarker to rule-in and rule-out severe outcome and the usefulness for allocating resources.

**Acknowledgement:** On behalf of the COVIDeF Study Group.

## P128

### Correlation between serum levels of IL-6 and positive fluid balance in critical COVID-19

#### OI Aguilera Olvera^1^, GA Aguirre Gómez^1^, GL Arcos López^1^, CD Del Ángel Argueta^2^, YN López Esquivel^1^, JC Muñoz Cháves^1^, GL Velázquez Estrada^1^, JA Villalobos Silva^1^, A Zarate Gracia^1^

##### ^1^High Specialty Regional Hospital “Bicentenario 2010”, Critical Care, Victoria, Mexico, ^2^High Specialty Regional Hospital “Bicentenario 2010” Anesthesiology, Victoria, Mexico

*Critical Care* 2023, **27(S1)**: P128

**Introduction:** IL-6 has been correlated as a prognostic biomarker for worsening sepsis and COVID-19 as well as positive fluid balance for duration of mechanical ventilation [1, 2].

**Methods:** We performed a retrospective cohort study to analyze the correlation between high levels of serum IL-6 and positive fluid balances in the first 24 h of ICU arrival with mechanical ventilation days. We included adult patient records of critical COVID-19 during 2020 from the High Specialty Regional Hospital “Bicentenario 2010”, all patients were intubated, received treatment according to guidelines inforced in that time. We obtained mean and standard deviation for continuous variables and frequencies for categorical variables, calculated Kolmogorov–Smirnov for non-parametric test and Spearman correlation, OR for severe hypoxemia, RRT.

**Results:** We analyzed 102 patient records, 72% were male, mean age 54.8 years (SD 19.4), tracheostomy was performed in 8.8% of cases, mean APACHE II 16.7 (SD 8.4), values of inflammatory markers were C-reactive protein 108 mg/dl (SD 95), IL-6 118 pg/ml (SD 240), mean paO2/FiO2 was 150 mmHg (SD 82), 93% were on vasopressors, fluid balance mean was 1542 ml (SD 839), severe hypoxemia was present on 62.7% (P/F below 150 mmHg), prono was used in 47.1%, with an overall mortality occurred in 69%. We found no correlation between serum IL-6 levels and positive fluid balance with mechanical ventilation days and outcomes (rs -0.11 *p* = 0.23, Fig. 1). Elevated serum IL-6 + positive fluid balance at 24 h ICU arrival was associated with severe hypoxemia (OR 2.82, CI 95% 1.14–6.97, x^2^
*p* = 0.022), OR for discharge was non-significant (0.48 CI 0.19–1.20 *p* = 0.11), RRT (1.09 CI 95% 0.27–4.37, *p* = 0.9).

**Conclusions:** In our study no correlation was found between serum IL-6 levels, positive fluid balance and mechanical ventilation days, but there was a significant association with severe hypoxemia.


**References**
Gorham J et al. PloS ONE 2020;15:e0244628.Wang W et al. Crit Care Med 2022;50:307–316.

**Figure Figat:**
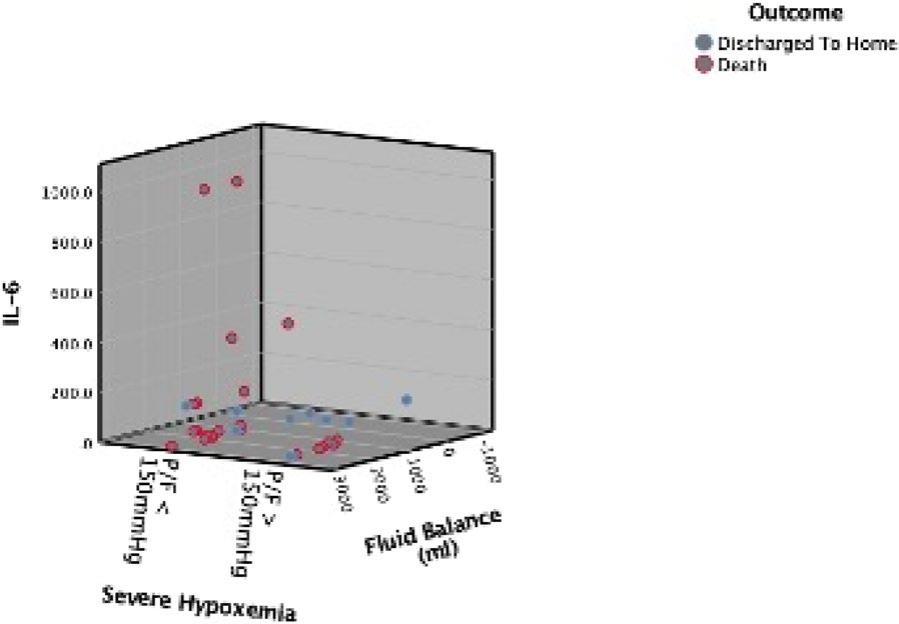
**Fig. 1 (abstract P128)**. Correlation between serum IL-6 levels + positive fluid balance with severe hypoxemia

## P129

### Usage of highly elevated procalcitonin levels for identification of urosepsis in intensive care unit patients

#### ID Basset^1^, V Kehl^2^, G Lorenz^3^, RM Schmid^1^, T Lahmer^1^

##### ^1^Klinikum rechts der Isar der Technischen Universität München, Klinik und Poliklinik für Innere Medizin II, München, Germany, ^2^Klinikum rechts der Isar der Technischen Universität München, Institut für KI und Informatik in der Medizin, München, Germany, ^3^Klinikum rechts der Isar der Technischen Universität München, Abteilung für Nephrologie, München, Germany

*Critical Care* 2023, **27(S1)**: P129

**Introduction:** Identification of a specific infection site is a cornerstone in sepsis treatment. Urosepsis is beside pneumonia one of the most common reasons for ICU administration in elderly patients. Procalcitonin (PCT) is a promising biomarker to help diagnose sepsis and guide treatment decisions. We aimed to assess the utility of highly elevated PCT concentrations in serum to predict urosepsis in critically ill patients.

**Methods:** We compared in a monocentric, retrospective study septic patients with PCT values > 50 ng/ml to patients with PCT < 15 ng/ml in terms of primary focus of infection. Kruskal–Wallis test was performed to show differences in PCT-values between different foci of infection in both groups. Chi-square automatic interaction detection (CHAID) was used to find cut-off-values to distinguish between foci of infection.

**Results:** 88 out of 255 included patients had a maximum PCT > 50 ng/ml within the first 48 h of ICU admission. Median age was 66 (60–76) years, 63.6% were male and median SOFA-Score was 12 (10–15). Patients with PCT > 50 ng/ml showed significantly more positive urine culture results compared to patients with PCT < 15 ng/ml (*p* < 0.001). In patients with PCT > 50 ng/ml, there was a significant difference in PCT values between patients with respiratory and genitourinary focus of infection (*p* = 0.019). Using CHAID, a genitourinary focus was only present in 12,7% of cases when PCT was < 180.5 ng/ml but in 47.1% when it was > 180.5 ng/ml (*p* = 0.011).

**Conclusions:** When reaching high values, PCT can help to differentiate genitourinary focus of infection in critically ill patients at admission to ICU, using a cut-off-value of 180.5 ng/ml. This may help timely administration of specific urological treatment and result in further decline of mortality rates in urosepsis.

## P130

### Procalcitonin combined to point of care molecular respiratory panel testing for antibiotic stewardship in the emergency room: the PROARRAY study

#### L Velly^1^, M Cancella de Abreu^2^, D Boutolleau^3^, I Cherubini^2^, E Houas^2^, P Hausfater^1^

##### ^1^Sorbonne Université APHP Hôpital Pitié-Salpêtrière, Emergency Department, Paris Cedex 13, France, ^2^Sorbonne Université APHP Hôpital Pitié-Salpêtrière, Paris Cedex 13, France, ^3^Sorbonne Université APHP Hôpital Pitié-Salpêtrière, Virology Department, Paris Cedex 13, France

*Critical Care* 2023, **27(S1)**: **P130**

**Introduction:** Molecular syndromic respiratory panel (RP) or procalcitonin (PCT)-driven algorithms have reported conflicting efficacy for antibiotic ATB) stewardship in LRTI. We hypothesized that combining real-time PCT measurement and virus identification would reduce ATB exposition in LRTI suspicions presenting to the emergency department (ED).

**Methods:** PROARRAY study is a prospective, randomized interventional trial, conducted in the adult ED of an academic 1600-bed hospital. Patients attending the ED with a suspicion of LRTI were randomized into the intervention arm (systematic PCT measurement and point of care BIOFIRE® RP2*plus* (then 2.1) testing, with the recommendation to withhold or withdraw ATB if PCT < 0.25 µg/L and/or identification of a virus) or a standard of care (SOC) arm. The primary endpoint was the duration of antibiotic exposure in the first 28 days.

**Results:** 451 patients were randomized (intervention: 225, SOC: 226), mean age 62.5 ± 19.4 years, hospitalization rate 59.9%, mean length of stay 7.4 ± 8.4 days. Main diagnoses were CAP (n = 129), COVID-19 (n = 91), AECOPD (n = 31). The BIOFIRE® RP2.1*plus* identified at least one viral species in 112 patients (49.8%). The duration of ATB exposition in ITT population was 6.00 [0.00;9.00] and 5.00 [0.00–9.00] days in the SOC and interventional arm respectively (*p* = 0.71). ATB was started in 31.3% and 34.1% respectively (*p* = 0.54). ATB exposure was below 6 days in 100 (47.2%) and 108 patients (50.59%) respectively (*p* = 0.58).

**Conclusions:** Displaying real-time PCT and RP results failed to significantly reduce the ATB exposition in LRTI suspicions. However, the ATB duration and rate of initiation were already low in SOC arm, which comprised PCT measurement in routine in our ED. Routine PCT measurement probably participated to the lower median ATB duration (6.0 days) than hypothesized (9.0 days) and argues for the main contribution of PCT in ATB stewardship. Moreover, as the intervention was done at ED’s level, we did not control for ATB stewardship in wards for inpatients.

## P131

### Temporal analysis of biomarker, severity-of-illness score, and machine learning model performance for sepsis prediction

#### M Patton^1^, VX Liu^2^, M Might^1^

##### ^1^University of Alabama at Birmingham, Hugh Kaul Precision Medicine Institute, Birmingham, USA, ^2^Kaiser Permanente Division of Research, Oakland, USA

*Critical Care* 2023, **27(S1)**: P131

**Introduction:** Adoption of electronic medical records has accelerated development of machine learning (ML) surveillance systems for predicting sepsis. To assess the progress of these new methods, we conducted a retrospective analysis of published models for sepsis detection.

**Methods:** We performed a systematic query of MEDLINE for predictive outcome research from 1995 to 2022. Studies were manually curated and selected based on a search criterion for a sepsis, bacteremia, or septic-shock outcome model with a performance evaluation using an area under the receiver operating characteristic curve (AUROC) or precision recall curve (AUPRC). We categorized models as biomarker, severity score, and/or ML algorithm.

**Results:** We identified 3393 sepsis prediction publications and selected 74 for further evaluation. The top performing biomarker and severity score-based models for sepsis detection were procalcitonin (AUROC range = 0.67–0.91) and the SIRS score (AUROC range = 0.65–0.81) for studies including > 1000 patient records. ML model performance for sepsis prediction as binary outcome exhibited a wide range in performance (AUROC range = 0.63–0.93). Newer approaches to assessing sepsis as a time-to-onset prediction had AUROCs ranging from 0.74 to 0.91, with neural networks exhibiting a higher performance range (AUROC = 0.64–0.91) compared to traditional regression methods (AUROC = 0.74–0.85) (Fig. 1). Studies incorporating novel data elements like clinical documentation often exhibit improved model performance. Inter-study comparison revealed a tremendously wide range in sepsis outcome rates from < 1% to as high as 50% across datasets and different definitions of sepsis, thereby underscoring the challenge of comparing performance across time and methods.

**Conclusions:** A review of sepsis prediction models published over 3 decades revealed a wide variability in model performance and identified numerous confounding differences between studies limiting robust comparison.

**Figure Figau:**
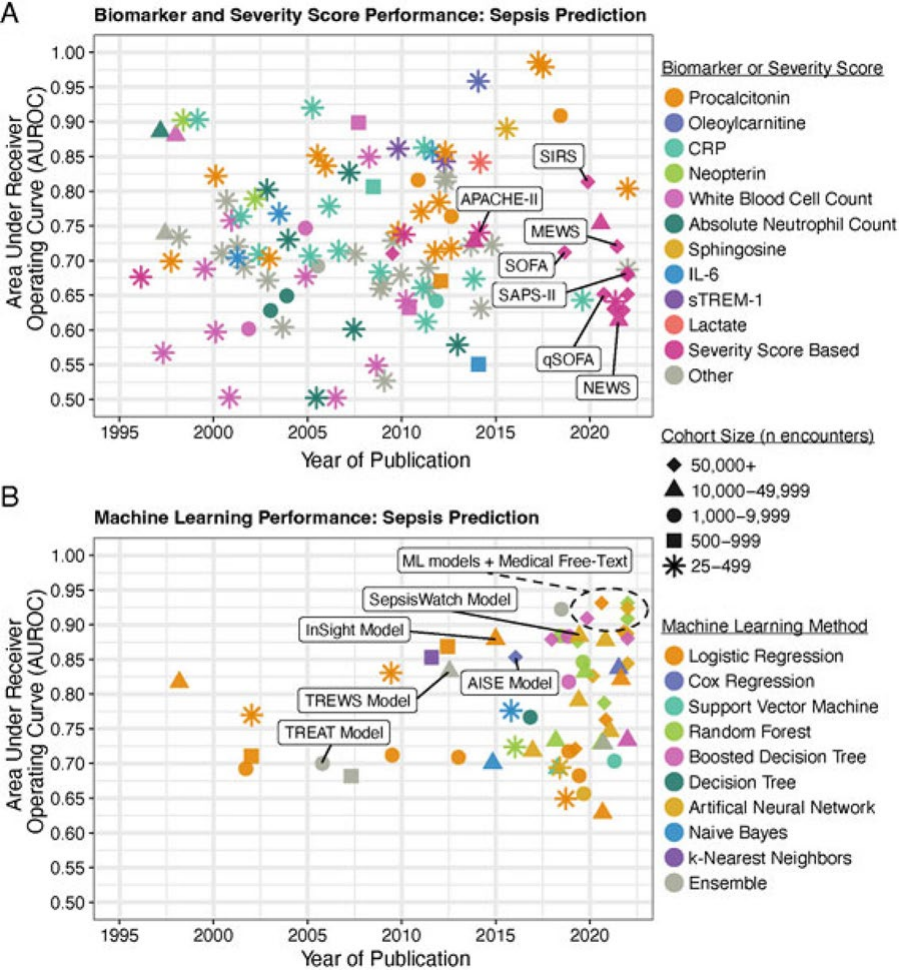
**Fig. 1 (abstract P131)**. Temporal analysis of biomarker, severity-of-illness score, and machine learning model performance for sepsis prediction from 1995 to 2022

## P132

### Predictive performance of pancreatic stone protein for ICU mortality in hospitalized adults with infection: a systematic review and individual patient level meta-analysis

#### J Prazak^1^, A Moser^2^, P Zuercher^1^, LG De Guadiana-Romualdo^3^, MJ Llewelyn^4^, R Graf^5^, T Reding^5^, P Eggimann^6^, YA Que^1^

##### ^1^Inselspital, Department of Intensive Care Medicine, Bern, Switzerland, ^2^University of Bern, CTU, Bern, Switzerland, ^3^Santa Lucia University Hospital, Biochemistry Department, Cartagena, Spain, ^4^Brighton and Sussex Medical School, Falmer, UK, ^5^Universitätsspital Zürich, Department of Visceral and Transplantation Surgery, Zürich, Switzerland, ^6^Lausanne University Hospital, Department of Locomotor Apparatus, Lausanne, Switzerland

*Critical Care* 2023, **27(S1)**: P132

**Introduction:** Pancreatic stone protein (PSP) has been shown in several studies to be a promising biomarker for prediction of mortality among patients with infection. The aim of this systematic review and individual patient level meta-analysis was to evaluate PSP predictive performance on ICU mortality and compare it to those of procalcitonin (PCT) and C-reactive protein (CRP).

**Methods:** In a systematic search on PubMed and Cochrane databases we identified 46 studies. Among them, five studies measuring the PSP by the ELISA technique explored the performance of PSP in stratifying unselected hospitalized adult patients on admission to the ICU/ED according to prognosis of mortality. We used hierarchical regression models to predict ICU mortality and evaluated predictive performance of PSP, PCT and CRP reporting classification plots besides AUC [1].

**Results:** Among 678 patients included into the analysis, the estimated overall ICU mortality was 17.8% with a 95% prediction interval (PI) ranging from 4.1 to 54.6% with a substantial heterogeneity between studies (I-squared 87%). For the three studied biomarkers, sensitivity and 1-specifity with continuous risk thresholds are shown as classification plots (Fig. 1). For PSP, the sensitivity was 0.96, 0.52, 0.30 for risk thresholds 0.1, 0.2 and 0.3 (values for false positive rate were 0.84, 0.25, 0.10). The AUC was 0.69 for PSP, 0.61 for PCT and 0.52 for CRP.

**Conclusions:** Classification plots are a key tool to support clinical decision making for treating patients with infections, because they allow to incorporate continuous risk threshold information. Compared to CRP and PCT, PSP revealed better predictive ability for ICU mortality in the studied heterogeneous population.


**Reference**
Verbakel JY et al. J Clin Epidemiol 2020;126:207–16.

**Figure Figav:**
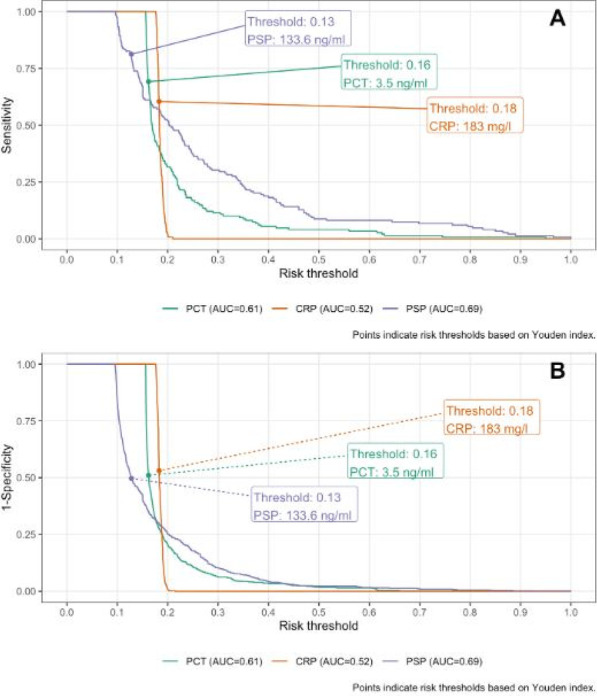
**Fig. 1 (abstract P132)**. Classification plots—ICU mortality, sensitivity (Panel A) and 1-specifity (Panel B) with continuous risk thresholds. Depicted risk thresholds are derived from Youden’s index

## P133

### Serum-calprotectin for identification of severe COVID-19 in hospitalized patients

#### Å Parke^1^, M Hansson^2^, B Strunz^3^, K Strålin^4^

##### ^1^MedH, Department of Medicin, Karolinska Hopital Huddinge, Huddinge, Sweden, ^2^MedH, Department of Clinical Chemistry, Karolinska University Hospital, Stockholm, Sweden, ^3^MedH, Center for Infectious Medicine, Department of Medicine Huddinge, Karolinska Institutet, Stockholm, Sweden, ^4^MedH, Department of Medicine Huddinge, Karolinska Institutet, Stockholm, Sweden

*Critical Care* 2023, **27(S1)**: P133

**Introduction:** The disease severity of COVID-19 varies from mild upper respiratory tract infection to life-threatening lower respiratory tract infection. Biomarkers can support severity assessment. We aimed to compare scalprotectin with routine biomarkers to study if s-calprotectin could identify patients with risk for severe COVID-19. We also aimed to study if ongoing corticosteroid therapy would alter levels of s-calprotectin.

**Methods:** We collected serum samples within 8 days from admission from 162 adult patients hospitalized for COVID-19 from April to October 2020. The serum tubes were centrifuged within 2 h and serum was aspirated and frozen in aliquots at −80 °C. scalprotectin was measured according to the routine method at Karolinska University Laboratory. Study endpoint was severe COVID-19 any time during the hospital stay. Severe COVID-19 was defined as treatment with either low-flow oxygen ≥ 10 l/min, high-flow nasal oxygen, non-invasive ventilation, or invasive mechanical ventilation. For patients hospitalized before the results of the RECOVERY Dexamethasone study were presented, a propensity score matched sub-study was performed, comparing patients treated and not treated with corticosteroids prior to study sample.

**Results:** Median s-calprotectin was, 2.7 ml/L (IQR, 1.6–4.2) for non-severe COVID-19 cases (n = 59) and 5.0 ml/l (IQR, 3.5–9.1) for severe COVID-19 (n = 103) (*p* < 0.001). ROC-curve analysis compering s-calprotectin, C-reactive protein (CRP), neutrophils, 1/lymphocytes, and D-dimer, showed the highest AUC for s-calprotectin, 0.775 (Fig. 1). In the sub-study of with corticosteroid treated (n = 35) and non-treated patients (n = 26), the median s-calprotectin was 4.0 (IQR, 2.5–5.6) and 3.8 (IQR, 2.7–3.8), respectively (*p* = 1.0).

**Conclusions:** The study showed a good performance of s-calprotectin for identification of severe COVID-19, in agreement with previous studies. It also indicated that s-calprotectin results are not affected by ongoing corticosteroid treatment.

**Figure Figaw:**
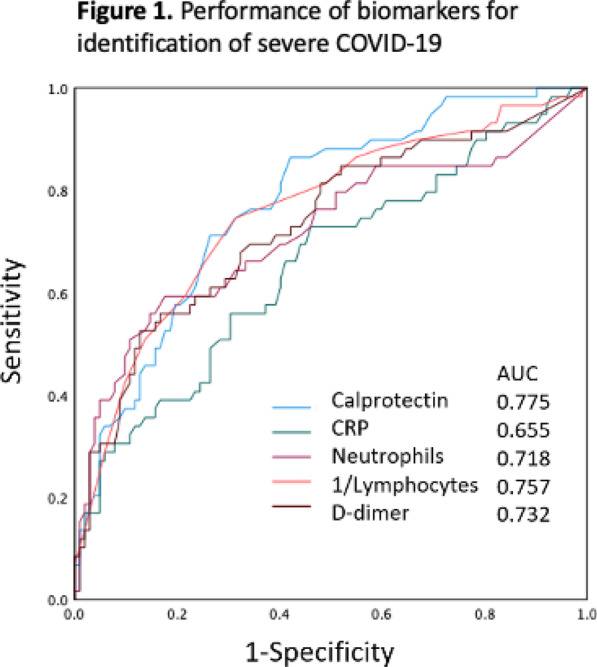
**Fig. 1 (abstract P133)**. Performance of biomarkers for identification of severe COVID-19

## P134

### Performance of a molecular assay in sepsis patients stratified by qSOFA

#### S Cermelli^1^, K Navalkar^1^, RF Davis^1^, R Balk^2^

##### ^1^Immunexpress, Seattle, USA, ^2^Rush Medical College and Rush University Medical Center, Chicago, USA

*Critical Care* 2023, **27(S1)**: P134

**Introduction:** We categorized ICU patients suspected of sepsis using quick Sequential Organ Failure Assessment (qSOFA), expert panel diagnosis, and a molecular assay (SeptiCyte RAPID) to demonstrate clinical utility of measuring host response to identify patients with an infection & systemic inflammation. Sepsis-3 definition can be operationalized by clinicians by assessing a qSOFA ≥ 2 [1]. However, clinicians first need to identify patients with an infection, which can be challenging because of heterogenous clinical signs [2].

**Methods:** Data was analyzed from clinical trials that enrolled ICU adult patients suspected of sepsis (n = 419) [3]. A full qSOFA calculated for 173 of these patients enabled stratification into < 2 or ≥ 2 groups. Expert panel diagnosis was performed retrospectively and independently classifying cases into systemic inflammation in response to infection (Inf+), or not (Inf-). SeptiCyte RAPID score (0–15) measured mRNA expression of two genes.

**Results:** For 173 ICU patients suspected of sepsis, 71 (41%) were retrospectively diagnosed as Inf + and 102 (59%) were diagnosed as Inf- (Fig. 1). Inf+ patients consisted of 55 (77%) with qSOFA ≥ 2, and 16 (23%) with qSOFA < 2. Inf + patients could be differentiated from Inf- using SeptiCyte RAPID irrespective of qSOFA ≥ 2 (AUC 0.82) or qSOFA < 2 (AUC 0.81).

**Conclusions:** SeptiCyte RAPID was able to differentiate patients suspected of sepsis into those with a systemic inflammatory response to infection (Inf+), versus those with a systemic inflammatory response due to non-infectious causes (Inf-). SeptiCyte RAPID achieved this independent of the qSOFA score < 2 or ≥ 2. The key to rapidly identifying patients with sepsis is to first determine infection status & then use qSOFA to determine likelihood of poor outcome.


**References**
Singer M et al. JAMA. 20,156;315: 801–810.Liu VX et al. Crit Care Explor. 2021;3: e0344.Balk R et al. medRxiv 2022.

**Figure Figax:**
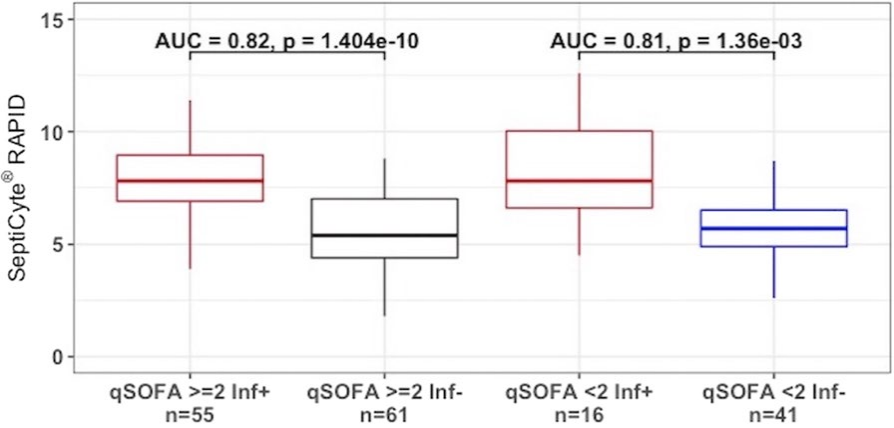
**Fig. 1 (abstract P134)**. Stratification of ICU patients (n = 173) suspected of sepsis based on qSOFA, expert panel diagnosis and SeptiCyte RAPID

## P135

### Biomarkers for organ dysfunction and outcome prediction in septic patients admitted in emergency department with community-acquired pneumonia

#### A Degrassi^1^, G Coppalini^2^, N Fiotti^3^, G Biolo^3^

##### ^1^ASUGI Institute, Department of Emergency Medicine, Trieste, Italy, ^2^Humanitas Research Hospital, Anaesthesiology and Intensive Care, Rozzano, Italy, ^3^ASUGI Institute, Department of Medical, Surgical and Health Sciences, Trieste, Italy

*Critical Care* 2023, **27(S1)**: P135

**Introduction:** Community-acquired pneumonia (CAP) is a lung infection acquired outside hospitals. Specific biomarkers could help identification of patients who may rapidly develop organ dysfunction and are at risk of early mortality, guiding physicians to decide for best patients allocation (ICU/medical ward) and relocate resources. Aim of our study is analyze the role of biomarkers in the prediction of specific organ dysfunction and mortality in septic CAP patients.

**Methods:** We conducted a secondary analysis of a prospective study held in the emergency department (ED) of five university hospitals in Italy [1]. Levels of CD64, PLA2GIIA, CD14 and CD25 were evaluated from samples collected within 12 h from ED arrival. Patients were followed until discharge for development of neurological, hepatic, renal, respiratory, cardiovascular and coagulation dysfunctions and in-hospital mortality was assessed.

**Results:** Of 368 patients included in the analysis, 87 (24%) died during their hospital stay. All biomarkers showed correlation with mortality, but only CD14 and CD25 had significant increase in non-survivor at multivariate analysis corrected for age and comorbidities (452.5 [292 -754] vs 769 [453–1402] *p* < 0.01 and 13215 [9003–20041] vs 20329 [12841–36729] *p* < 0.01). Higher levels of CD14 were associated with neurologic dysfunction, hepatic and cardiovascular injury. All biomarkers were increased when coagulation disorder and renal injury developed. CD14 was the best predictor of mortality (Fig. 1) and of both these complications at ROC curve analysis (AUC 0.684, OR 4.07 [CI 2.14–7.73] and AUC 0.757, OR 5.59 [CI 3.42–9.14]).

**Conclusions:** Higher levels of CD25 and CD14 were the best predictors of mortality in septic CAP patients. Regarding organ dysfunctions, CD14 was the best predictor of neurologic, cardiovascular, renal and coagulation disorders. Further studies will be needed to better characterize role of biomarkers for outcome stratification in CAP patients.


**Reference**
Mearelli F et al. Crit Care Med, 20,018;46:1421–1429.

**Figure Figay:**
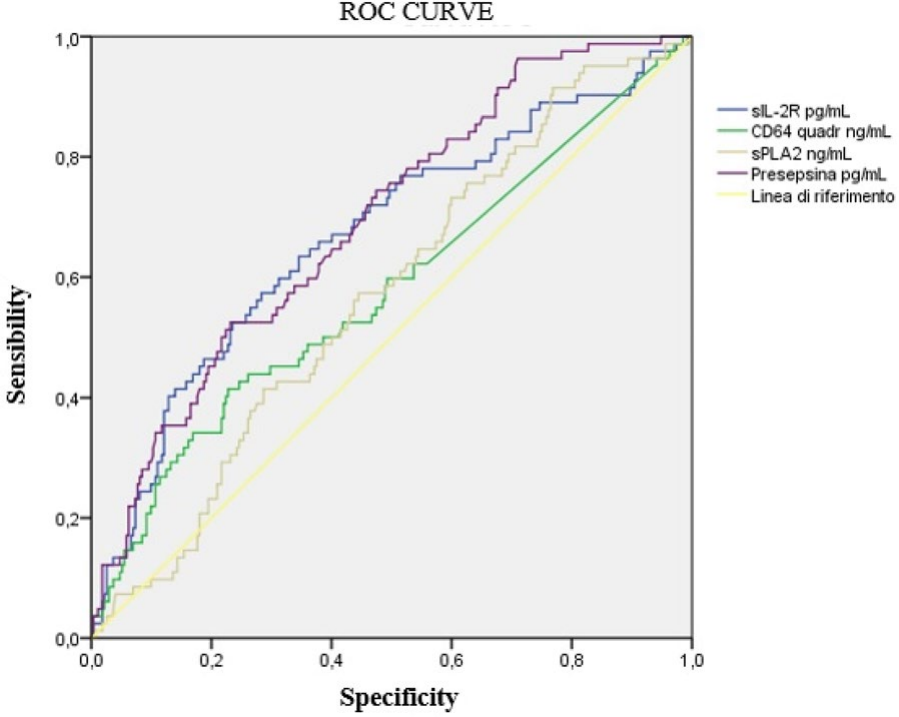
**Fig. 1 (abstract P135)**. ROC curve of considered biomarkers to predict mortality in CAP patients. Best prognostic role was found to be CD14 with 0.692 AUC

## P136

### Importance of damage-associated molecular patterns and pathogen-associated molecular patterns for severe outcome in bacteraemic infection

#### H Alpkvist^1^, I Ziegler^2^, P Mölling^2^, E Tina^2^, A Norrby-Teglund^3^, S Cajander^2^, K Strålin^4^

##### ^1^Karolinska Institutet, Department of Medicine Huddinge, Division of Infectious Diseases, Stockholm, Sweden, ^2^Department of Infectious Diseases, Örebro University Hospital, Örebro, Sweden, ^3^Karolinska Institutet, Center for Infectious Medicine, Department of Medicine Huddinge, Karolinska Institutet, Stockholm, Sweden, ^4^Karolinska Institutet, Department of Medicine Huddinge, Division of Infectious Diseases, Karolinska Institutet, Stockholm, Sweden

*Critical Care* 2023, **27(S1)**: P136

**Introduction:** We aimed to study the importance of damage-associated molecular patterns (DAMPs), i.e. nuclear DNA (nDNA), and Pathogen-associated molecular patterns (PAMPs), i.e. bacterial 16S DNA, and their correlation to severe outcome in patients with bacteraemic infections.

**Methods:** Adult patients hospitalized with culture-proven bacteraemic infection caused by *Streptococcus pneumoniae* (n = 28), *Staphylococcus aureus* (n = 24), or *Escherichia coli* (n = 11) were included in a prospective study. Plasma samples were collected on admission (day 0) and on days 1–2, 3–4, 7 ± 1, 14 ± 2, and 28 ± 4. Plasma was analyzed for nDNA and 16S DNA using specific quantitative PCR methods. Sepsis was defined according to Sepsis-3. Study outcome was severe outcome, defined as admission to an intensive care unit (ICU) and/or death within 60 days from admission.

**Results:** Of 63 included patients with bacteraemic infection, 37 (59%) had sepsis at admission. Severe outcome was noted in 16 patients (25%), including 11 patients admitted to an ICU and 7 non-survivors. In the univariate analysis, severe outcome was was associated with DAMP levels (nDNA) and PAMP levels (16S DNA) on day 1–2, but not with gender, age, comorbidity or aetiology (Table 1). In a multivariate analysis, both DAMP and PAMP levels on day 1–2 were independently associated with severe outcome (Table 1). 16S DNA was most often cleared from the circulation within a few days, only 3 patients had measurable 16S DNA on day 14. nDNA, on the contrary, remained at high levels over time in non-survivors, and nDNA on day 28 was significantly higher in patients who died within 60 days.

**Conclusions:** High levels of DAMP and high levels of PAMP were independently associated with severe outcome in patients with bacteraemic infection.

**Table Tabak:** **Table 1 (abstract P136)**. Univariate and multivariate analysis for severe outcome

Factors	Univariate analysis odds ratio (95% CI)	Univariate analysis *p* value	Multivariate analysis odds ratio (95% CI)	Multivariate analysis *p* value
Female	1.90 (0.60–5.96)	0.28		
Age	1.03 (0.98–1.08)	0.26		
Charlson comorbidity index	1.17 (0.92–1.50)	0.20		
*Staphylococcus aureus* versus *Escherichia coli*	1.10 (0.22–5.40)	0.91		
*Streptococcus pneumoniae* versus *Escherichia coli*	0.73 (0.15–3.62)	0.70		
Plasma nDNA, log10 copies/μL (DAMP) on day 1–2	8.99 (1.63–49.57)	0.012	7.88 (1.21–51.49)	0.031
Plasma 16S DNA, log10 copies/ml (PAMP) on day 1–2	1.47 (1.12–1.92)	0.005	1.41 (1.08–1.85)	0.013

## P137

### The biomarkers sRAGE and d_*f*_ in combination are significant predictors of mortality in COVID-19 patients

#### S Pillai^1^, JC Zaldua^1^, L Butcher^2^, JA Carnicero^2^, K Hawkins^1^, J Whitley^1^, R Mothukuri^1^, K Morris^2^, JD Erusalimsky^2^, PA Evans^1^

##### ^1^Welsh Centre for Emergency Medicine Research, Emergency Department, Morriston Hospital, Swansea, UK, ^2^The Cellular Senescence and Pathophysiology Group, Cardiff Metropolitan University, Cardiff, UK

*Critical Care* 2023, **27(S1)**: P137

**Introduction:** COVID-19 infection is associated with marked inflammatory response and the patients who are admitted to the hospital are at increased risk of developing venous thromboembolism. sRAGE (soluble receptor for advanced glycation end-products) are acutely elevated in host inflammatory response to infections [1]. Fractal dimension (d_*f*_), the biomarker of clot microstructure that measures thrombogenicity has shown to be elevated in acute inflammatory conditions such as sepsis and severe sepsis. The aim of the study was to analyse these biomarkers in COVID-19 infection and whether these biomarkers help to predict mortality.

**Methods:** 120 suspected COVID-19 patients were recruited from the Emergency Department of a tertiary teaching hospital. One patient was excluded because they were anticoagulated, blood samples were taken to perform fractal dimension (d_*f*_) and sRAGE.

**Results:** When compared to PCR -ve group, 95 patients in the PCR + ve group had significantly elevated sRAGE (*p* < 0.001), but not d_*f*_ (*p* = 0.43). When compared to those who survived, sRAGE was significantly elevated (*p* = 0.01) in 14 patients who died in PCR + ve group, but not d_*f*_ (*p* = 0.08). No significant correlation existed between sRAGE levels and d_*f*_ in those patients who survived (*p* = 0.72) or died (*p* = 0.92). Logistic regression analysis showed that sRAGE and d_*f*_ in combination acted as highly significant predictors of mortality in COVID-19 (*p* = 0.009) in PCR + ve group.

**Conclusions:** COVID-19 patients had a profound inflammatory response as evidenced by significantly elevated sRAGE levels. This inflammatory process was more profound in those who died. The thrombogenicity in COVID-19 patients and those who died with COVID-19 appears to be not significant as measured by d_*f*_. sRAGE in combination with d_*f*_ can be utilised as significant predictors of mortality in COVID-19 patients.


**Reference**
Butcher L et al. Respir Res 2022;23:303


## P138

### VAP BioFire utilisation in diagnosis of ventilator associated pneumonia—are we misfiring in our application of this tool?

#### D Clarke, R Bart Koranteng, S McKechnie

##### Oxford University Hospitals Foundation Trust, Oxford Critical Care, Oxford, UK

*Critical Care* 2023, **27(S1)**: P138

**Introduction:** Ventilator associated pneumonia (VAP) is a nosocomial infection with high associated morbidity and mortality. Timely, accurate diagnosis of VAP and appropriate antibiotic therapy can impact outcomes. The disease itself is poorly defined and diagnosis often limited by low yield sampling techniques and delays in growth of microbiological samples. The use of rapid diagnostics such as BioFire VAP FilmArray have shown promise in reducing time to identification of pathogen, with hopes this may lead to more targeted antibiotic therapy. However, the benefit of this is unclear and relies on ICU clinicians having trust in the sampling technique and test itself. There Is a signficant cost burden to the test which on our unit was part of a routine order-set in patients suspected of VAP. We aimed to reduce uncessary sample through a QI process.

**Methods:** We performed a retrospective analysis of clinical data from electronic health records of all patients admitted to our ICU between December 2021 to January 2022, and then a repeat analysis post intervention between December 2022. All patients ventilated greater than 48 h were included for analysis. Data on length of stay, mortality, duration of antibiotics, number of BioFire samples sent, and change to therapy post test were recorded.

**Results:** BioFire lead to a change in therapy in 21% of patients. This was an escalation of antibiotics 66% of the time. The test never led to a cessation of antibiotic therapy. Following intervention (a package of education and removal of the test from our routine order set) there was an 90% reduction in unnecessary sampling. There was no significant change to duration of antibiotic, ICU length of stay or mortality in patients diagnosed as VAP following the change.

**Conclusions:** The use of BioFire FilmArray tests rarely led to a clinical change on our unit. Removing the test from a routine order set did not seem to have a significant clinical impact. More work Is required to assess their use as an microbial stewardship tool.

## P139

### Prognostic analysis of cuproptosis-related gene in sepsis

#### HB Chen, HB Qiu

##### Jiangsu Provincial Key Laboratory of Critical Care Medicine, Department of Critical Care Medicine, Zhongda Hospital, School of Medicine, Southeast University, Nanjing, China

*Critical Care* 2023, **27(S1)**: P139

**Introduction:** Sepsis is a common disease in intensive care unit with high morbidity and mortality, which is a serious threat to human health. Regulated cell death plays an important role in the development of sepsis and serves as a potential therapeutic target. Cuproptosis is the most recently identified copper-dependent regulated cell death form that relies on mitochondria respiration. However, its role in the development of sepsis remains unclear. The correlation between cuproptosis-related genes (CRGs) with sepsis prognosis is also unclear.

**Methods:** We screened the data of sepsis patients with survival data through the Gene Expression Omnibus datasets (GEO), and collected CRGs based on previous studies. We used consensus clustering to identify gene subtypes based on CRGs, then univariate Cox regression analysis were performed on the differentially expressed genes to identify prognostic genes, and LASSO Cox regression were used to identify core genes and calculate CRG score, in addition, we create a prognostic model by multivariate Cox regression.

**Results:** In total, 479 sepsis samples with survival data were collected from GEO database. Most of CRGs were differentially expressed between normal and sepsis patients, and we identified two subclusters based on CRGs, and patients in cluster B had better clinical outcomes (Fig. 1). We identified 463 differentially expressed genes between the two subclusters, and 191 of them were associated with the prognosis of sepsis patients. Then, a 17-gene signature was identified utilizing the LASSO Cox regression model, and we created and validated a CRG score based on the 17-gene expression. Multivariate Cox regression analysis demonstrated CRG score was an independent prognostic factor.

**Conclusions:** Our thorough examination of CRGs in sepsis patients revealed the potential effects of CRGs on prognosis of sepsis. The CRGs is helpful in prognostic prediction and in guiding treatment for sepsis patients.

**Figure Figaz:**
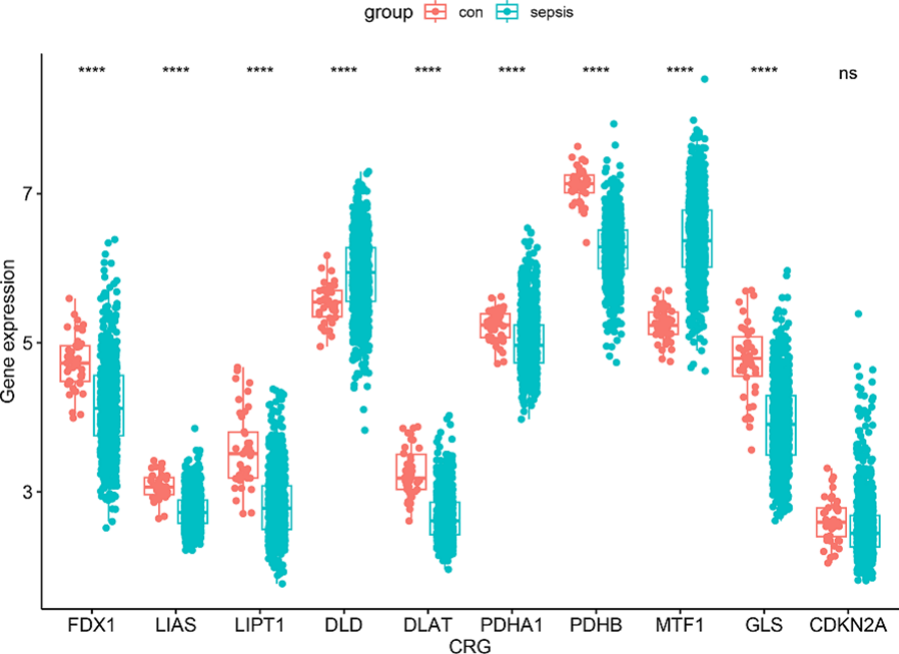
**Fig. 1 (abstract P139)**. CRGs expressed in sepsis and normal patients. CRGs = cuproptosis-related genes

## P140

### Plasma nucleosome measurement as a biomarker for NETs in intensive care patients

#### J Micallef^1^, M Herzog^1^, J Candiracci^1^, L Morimont^2^, J Douxfils^2^, A Aswani^3^, D Genkin^4^, P Skorup^5^, M Lipcsey^6^

##### ^1^Belgian Volition SRL, Isnes, Belgium, ^2^Department of Pharmacy, University of Namur, Namur, Belgium, ^3^Department of Critical Care Medicine, Guy’s and St Thomas’ NHS Foundation Trust, London, UK, ^4^Santersus AG, Kusnacht, Switzerland, ^5^Department of Medical Sciences, Uppsala University, Uppsala, Sweden, ^6^Department of Surgical Sciences, Uppsala University, Uppsala, Sweden

*Critical Care* 2023, **27(S1)**: P140

**Introduction:** Circulating NETs have been measured using a variety of proxy biomarkers including MPO, MPO-DNA, NE, histones and cfDNA. These assays are not standardised and typically not analytically validated. We have developed the first analytically valid automated immunoassay for nucleosomes which has been CE marked as a diagnostic tool to aid the detection and evaluation of diseases associated with NETosis in Europe.

**Methods:**


*Human studies—correlation of NETs levels with SOFA and APACHE II scores in sepsis patients*. The Volition Nu.Q®-NETs assay was used to measure plasma nucleosome levels in samples taken from 46 patients with septic shock (Sepsis-3) within 2 days of admission to ICU and from 48 age, gender and comorbidity matched controls.


*Animal studies to monitor therapeutic removal of NETs*


The Volition Nu.Q®-NETs assay was used to monitor plasma nucleosome levels to assess the efficacy of a novel apheresis treatment to remove circulating NETs in a porcine model of sepsis induced by a 3 h infusion of *E. coli*. The apheresis column used contained histone H1 as a specific binder of cfDNA and NETs. The efficiency of NETs removal from pig plasma passed through the apheresis column was also assessed.


**Results:**


*Human studies:* Measured nucleosome levels correlated with disease severity as indicated by SOFA and APACHE II scores.

*Animal studies: E. coli* infusion resulted in a continuous increase in nucleosome levels over 7 h from 58 to 356 ng/ml in a control (sham treated) pig. In contrast, levels plateaued at 120 ng/ml in the treated pig with attenuated septic shock, reduced lactate (4.3 vs 9.1 mmol/l), reduced noradrenaline requirement (120 ug vs 12,800 ug) and increased urine output (875 vs 340 ml). 97.7–99.0% of *E. coli* induced NETs present in the plasma of the treated pig were removed by a single pass of plasma through the apheresis column.

**Conclusions:** Nucleosome measurements using the Volition Nu.Q®-NETs assay may be useful in the management of patients diagnosed with sepsis.

## P141

### Prognostic value of detection of bloodstream bacterial DNA in sepsis

#### D Yu^1^, B Stammler Jaliff^2^, A Somell^3^, P Dinnétz^4^, M Ullberg^1^, J Wernerman^5^, V Özenci^1^, K Strålin^2^

##### ^1^Karolinska Institutet, Department of Laboratory Medicine, Huddinge, Sweden, ^2^Karolinska University Hospital, Department of Infectious Diseases, Stockholm, Sweden, ^3^Karolinska University Hospital, Functional Area of Perioperative Medicine and Intensive Care, Stockholm, Sweden, ^4^Södertörn University, School of Natural Sciences, Technology and Environmental Studies, Stockholm, Sweden, ^5^Karolinska Institutet, Department of Clinical Science, Intervention and Technology, Huddinge, Sweden.

*Critical Care* 2023, **27(S1)**: P141

**Introduction:** Using advanced molecular biological methods, such as PCR/electrospray ionization—mass spectrometry (PCR/ESI–MS), specific bacterial DNA can be detected in the bloodstream more frequently than bacteria can be detected by blood culture (BC) [1]. In the present study, we aimed to study the prognostic value of detected bloodstream bacterial DNA in patients with suspected sepsis.

**Methods:** Single center prospective cohort study conducted in Stockholm, Sweden. Adult patients admitted to the intensive care unit (ICU) with suspected infection based on the Centre for Disease Control and Prevention criteria for classifying infections in critically ill patients were included and subjected to sampling with BC and a whole blood tube (5 mL) for bacterial DNA (PCR/ESI–MS) on ICU admission. Presence of bloodstream bacterial DNA, positive BC and clinical characteristics were included in a multivariate analysis determining odds ratio (OR) for 60-days all-cause death after ICU admission.

**Results:** A total of 153 patients with infection were included, of which 48 patients (31.4%) had septic shock and 97 patients (63.4%) had sepsis without shock. In total, 52 patients (34.0%) had positive BC, and 72 patients (47.1%) had bloodstream bacterial DNA detected. Death within 60 days occurred in 42 patients (27.5%) and the adjusted odds ratio for death was 1.72 (95% CI 0.62–4.74) for bloodstream bacterial DNA and 1.07 (95% CI 0.36–3.23) for blood culture.

**Conclusions:** Neither blood culture nor bloodstream bacterial DNA was significantly associated with mortality in sepsis. However, the absolute OR of 1.7 for bloodstream bacterial DNA was similar to that of a previous study showing an association between bloodstream bacterial DNA and mortality in sepsis [2]. Additional studies are needed to further assess the prognostic value of bloodstream bacterial DNA in sepsis.


**References**
Tassinari M et al. PLoS One 2018;13:e0197436.O’Dwyer MJ et al. Clin Microbiol Infect 2017;23:208 e201–208 e206.


## P142

### Impact of corrective audits on the prevalence of primary bloodstream infections

#### EP Perecmanis, GDS Santos

##### Caxias Dor Hospital, Intensive Care, Duque de Caxias, Brazil

*Critical Care* 2023, **27(S1)**: P142

**Introduction:** Assess the impact of corrective audits on the prevalence of primary bloodstream infections.

**Methods:** Study conducted with prospectively collected data from January 2021 to June 2022 with a total of 3271 patients included. The intervention was implemented in June 2021, totaling 728 patients before the intervention and 2543 patients after. The central venous catheter utilization rate, the incidence of primary bloodstream infections and the mortality rate standardized by the SAPS3 score were compared. The monthly incidence rate can be viewed on Fig. 1.

**Results:** The incidence density of microbiologically proven primary bloodstream infections prior to the implementation of the audits was 0.79, with a central venous catheter utilization rate of 49.41%. The average achieved by the SAPS3 score of these patients was 56.63 points with a probability of death of 29.88%. The standardized in-hospital mortality rate (observed/expected) was 0.27. After the intervention, the incidence of laboratory-proven infections was 0.20, with a central venous catheter utilization rate of 33.80%. The mean by the SAPS3 score in the period was 50.62, with an in-hospital mortality rate (observed/expected) of 0.27.

**Conclusions:** Although there was no reduction in the standardized mortality rate, the implementation of audits seems to be effective in reducing the prevalence of primary bloodstream infections.

**Figure Figba:**
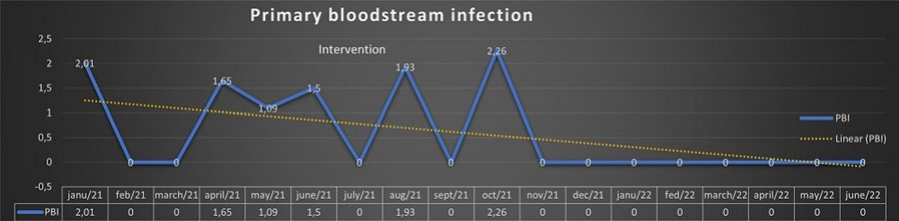
**Fig. 1 (abstract P142)**. Primary bloodstream infection incidence

## P143

### Who is in my brain?

#### T Aydin^1^, SC Cetintas^2^, AB Keles^3^, HB Tokman^3^, O Kizilkilic^4^, MM Hanci^2^, OF Tabak^5^, I Bavunoglu^6^, G M. Koksal^7^

##### ^1^Cerrahpasa Faculty of Medicine, Internal Medicine, Intensive Care, Istanbul, Turkey, ^2^Cerrahpasa Faculty of Medicine, Department of Neurosurgery, Istanbul, Turkey, ^3^Cerrahpasa Faculty of Medicine, Department of Medical Microbiology, Istanbul, Turkey, ^4^Cerrahpasa Faculty of Medicine, Department of Radiology, Istanbul, Turkey, ^5^Cerrahpasa Faculty of Medicine, Department of Infectious Diseases and Clinical Microbiology, Istanbul, Turkey, ^6^Cerrahpasa Faculty of Medicine, Department of Internal Medicine, Istanbul, Turkey, ^7^Cerrahpasa Faculty of Medicine, Department of Internal Intensive Care, Istanbul, Turkey

*Critical Care* 2023, **27(S1)**: P143

**Introduction:** Primary amoebic meningoencephalitis (PAM) is rare but a life-threatening infection caused by free-living amoebas (FLA) [1]. Herein we present a complex PAM case that has been successfully treated.

**Methods:** 87 year old male was transferred to intensive care unit with an initial diagnosis of viral encephalitis. Medical history was normal except for a subdural hematoma operation 20 years ago. He was in Turkey for vacation. The patient was somnolent; had fever, altered sensorium and pneumonia. Leukocytosis and hyponatremia were present. Meropenem, vancomycin, clarithromycin and acyclovir treatments were started. Cultures and Herpes, Legionella, Listeria and tuberculosis tests were negative. The patient was orotracheal intubated due to generalized status epilepticus. Cranial imaging showed increased intracranial pressure, meningeal enhancement and subdural empyema in the right frontoparietal Burr-Hole region. He was operated. Incisions were made on the old scars and the Burr-Holes from previous surgery had been used. The CSF sample was colorless and had clean appearance. 20 leukocytes were counted and motile microorganisms with flagella were noticed during the count. A protozoa with vibratory movement was seen in the wet-mount preparation at 40× magnification (Fig. 1). Intravenous(IV) amphotericin-B, azithromycin, metronidazole and dexamethasone were administered. The patient, who was intubated for 11 days, was transferred from ICU on the 26th day healthily.

**Results:** PAM is an acute disease of the central nervous system caused by N. *fowleri*. Mortality of FLA infections may exceed 90%. Only 7 of 381 confirmed PAM cases survived and all of them got IV amphotericin-B [2].

**Conclusions:** Early diagnosis, neuroprotective management and effective treatment improve prognosis. Recently, miltefosine is proved to be effective.

**Acknowledgement:** Informed consent was obtained from the patient to publish the clinical details.


**References**
Jain R et al. Neurol India 2002;50:470–2.Gharpure R et al. Clin Infect Dis. 2021;73:e19–e27

**Figure Figbb:**
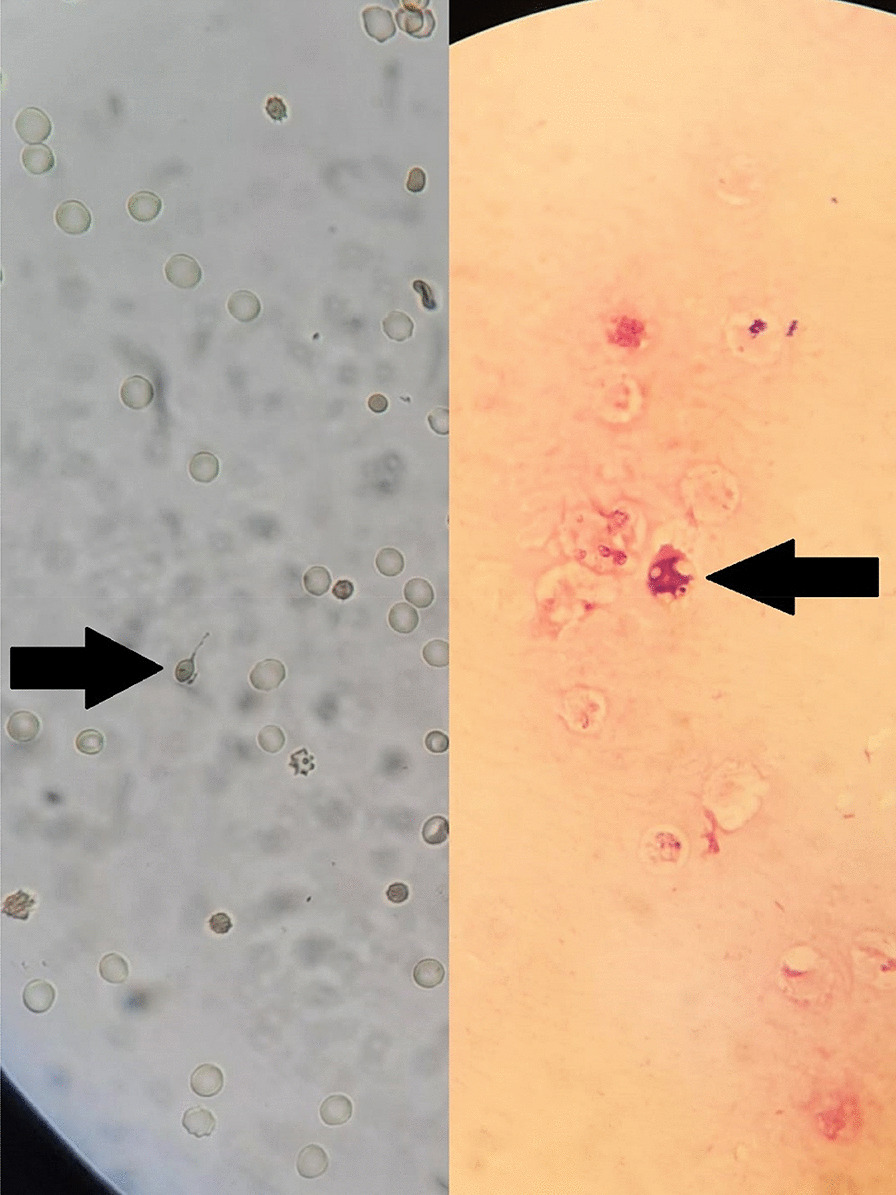
**Fig. 1 (abstract P143)**. The pathognomonic images of the protozoa are shown by wet-mount preparation (left) and Gram staining (right)

## P144

### Toxic epidermal necrolysis, a difficult diagnosis

#### J Rábano Alonso, L Cachafeiro Fuciños, A Agrifoglio Rotaeche, M Sánchez Sánchez, I Seises García, J Vejo Gutierrez, J Soto Gomez—Cambronero, R Yébenes Calvo

##### Hospital Universitario La Paz, Intensive medicine, Madrid, Spain

*Critical Care* 2023, **27(S1)**: P144

**Introduction:** Toxic epidermal necrolysis(TEN) and Stevens-Johnson syndrome (SJS) are rare, potentially disabling and fatal cutaneous diseases. Despite recent advances mortality is high, diagnosis remains a challenge and controversy exists regarding its treatment [1]. Our study describes the main characteristics of patients admitted with TEN/SJS.

**Methods:** A retrospective descriptive study was developed in the Critical Burn Unit of Hospital La Paz between 2013 and 2021. The diagnosis of TEN/SJS was confirmed by biopsy. Medical care was provided by a multidisciplinary team and immunosuppressive drugs was decided on an individualized basis. Demographic data, SCORTEN, frequencies of ARDS, shock, AKI requiring renal replacement therapy (RRT), infections, immunosuppressive drugs used, doubts in the clinical diagnosis and mortality were collected.

**Results:** 28 patients were admitted; 13 TEN, 8 TEN-SJS overlap and 6 SJS. In 5 patients with TEN/SJS (confirmed by biopsy) there were diagnostic doubts at clinical evaluation, and 5 patients with clinical suspicion of TEN were eventually diagnosed with other serious skin conditions: graft-versus-host disease, paraneoplastic pemphigus, and drug reaction with eosinophilia and systemic symptoms. Among those with TEN we found: mean age 56 ± 17 years; 13 men; mean skin detachment area 33 ± 26% (5–90); SCORTEN 3; mortality 31%; 11 presented shock, 5 ARDS, 5 needed RRT and 14 had severe infections. Figure 1 shows immunosuppressive drugs.

**Conclusions:** Data and mortality are consistent with literature and SCORTEN. Heterogeneity in immunosuppressive drugs used highlights the lack of consensus in treatment. Finally, similarities between the clinical presentation with other serious skin diseases highlight the difficulty in diagnosis and the possible delay in treatment. In any case, high-quality multidisciplinary supportive care is essential in all patients with severe skin diseases. Further studies are needed to improve the care of these patients.


**Reference**
Jacobsen A et al. Cochrane Database Syst Rev 2022:CD013130.

**Figure Figbc:**
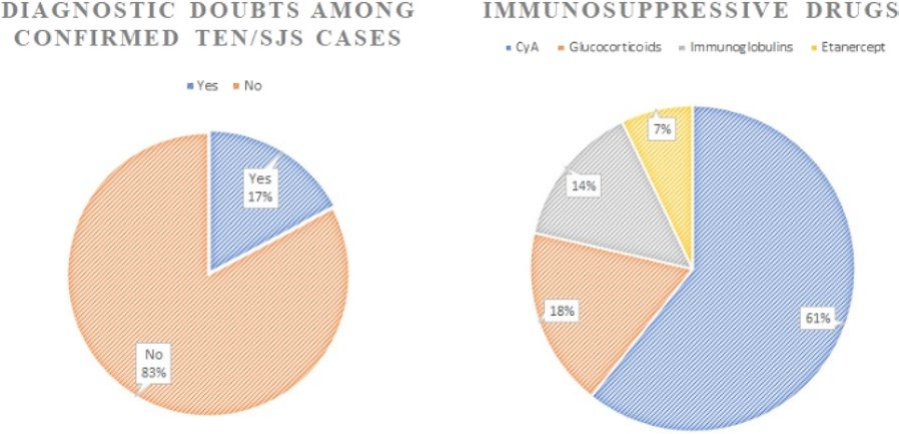
**Fig. 1 (abstract P144)**. Relative frecuencies of diagnostic doubts among confirmed TEN/SJS cases and immunosuppressive drugs used

## P145

### Dynamics of severe comorbidity and survival in* Clostridioides difficile* patients during 10 year period

#### D Adukauskienė, R Mickus, A Dambrauskienė

##### Medical Academy, Lithuanian University of Health Sciences, Kaunas, Lithuania

*Critical Care* 2023, **27(S1)**: P145

**Introduction:** Comorbidities are a well-established risk factor for contracting *Clostridioides difficile* infection (CDI) [1]. The aim of this study was to evaluate the prevalence of severe comorbidity and its impact on in-hospital mortality in patients (pt) with CDI.

**Methods:** We conducted a single-center, retrospective 10 years study in a reference Lithuanian university hospital. Pt with initial episode of CDI (diarrhoea and positive stool test for *C. difficile* toxin A/B) were included. The impact of severe comorbidity on 30 day in-hospital survival rate has been estimated in period of 2011–2020. Prevalence of severe comorbidity (Charlson Comorbidity Index (CCI) ≥ 5) and its relation to in-hospital survival have been estimated in both group 1 (2011–2016) and 2 (2017–2020). IBM SPSS 23.0, Kaplan Meier model, log-rank and Pearson’s χ^2^ tests were used for statistics, level of significance—*p* < 0.05.

**Results:** A total of 370 pt was enrolled. In 2011–2020 a lower 30 day in-hospital survival amongst CDI pt with CCI ≥ 5 was found (*p* = 0.000001, Fig. 1). In group 1 there were n = 197 pt and in group 2 n = 173 pt. In group 1 CCI ≥ 5 has been estimated in n = 121 (61.4%) pt and n = 169 (76.9%) have survived. In group 2 on contrary, both severe comorbidity and survival decreased: n = 80 (47.1%) pt and n = 143 (62.5%), accordingly (*p* = 0.005, *p* = 0.038).

**Conclusions:** Severe comorbidity has decreased, but in-hospital survival recently has not improved in CDI patients. Severe comorbidity as a prognostic factor has been associated with lower 30 day in-hospital survival in a period of 10 years.


**Reference**
Anjewierden S et al. Infect Control Hosp Epidemiol 2021;42:565–72.

**Figure Figbd:**
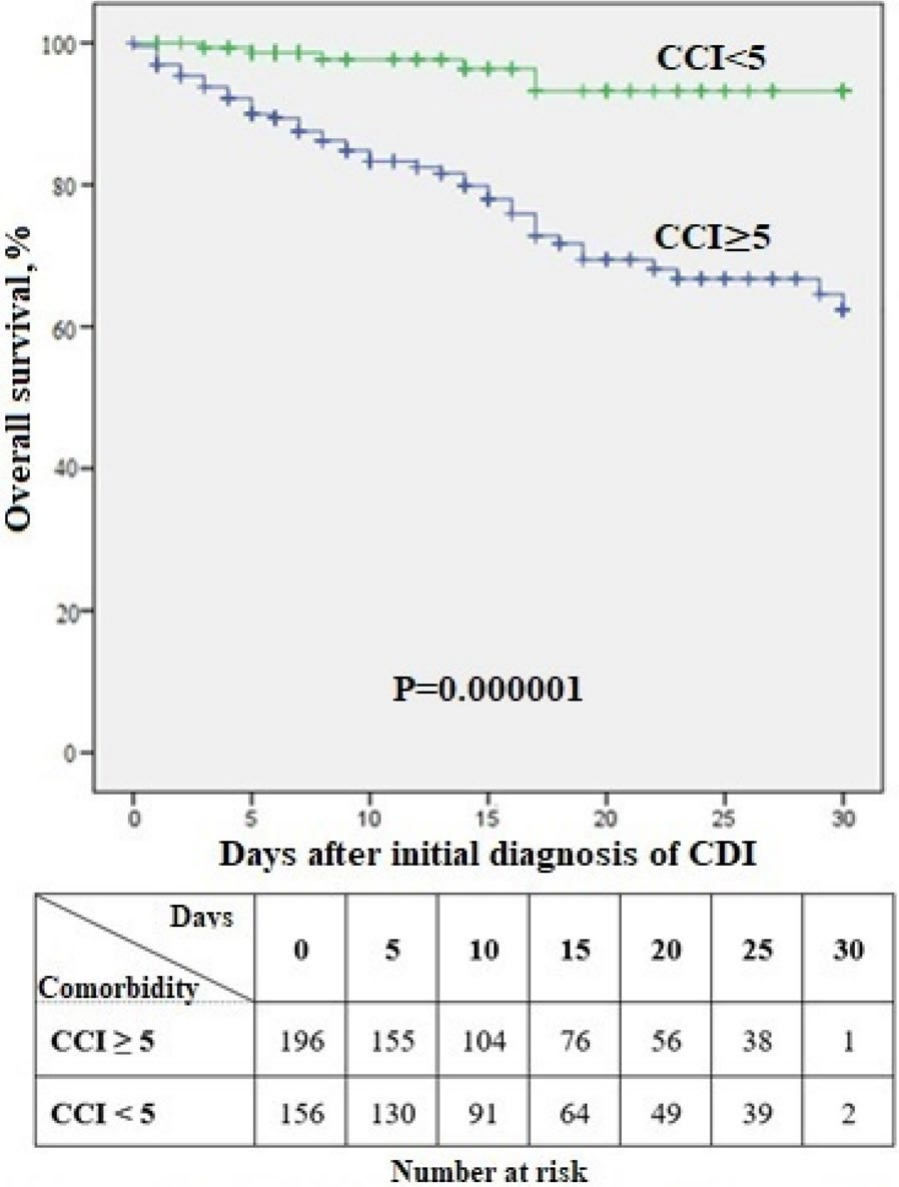
**Fig. 1 (abstract P145)**. Kaplan–Meier plots estimating 30-day survival of CDI with and without severe comorbidity in a period of 10 years

## P146

### In vitro activity of antibiotics against* Clostridioides difficile*: can we rely on treatment guidelines?

#### J Závora, G Kroneislová, V Adámková

##### General University Hospital, Clinical Microbiology and ATB Centre, Prague, Czech Republic

*Critical Care* 2023, **27(S1)**: P146

**Introduction:**
*Clostridioides difficile* (CLDI) is a well-recognized causative agent of severe healthcare associated diarrhoea. Recently, there was an update of European CLDI infection (CDI) treatment guidelines removing metronidazole from recommended regimen if vancomycin and fidaxomicin are available. Vancomycin is the most used agent against CDI and fidaxomicin is recommended only for treatment of initial CDI or the first recurrence. Several other agents with potential anti-CDI effect, such as tigecycline or rifampicin, are being considered and tested.

**Methods:** We have chosen 60 isolates of CLDI obtained from stool samples of patients hospitalized in General University Hospital in Prague in years 2020–2021. All samples have been initially tested by quick immunochromatographic test for detection of antigen (GDH) and toxin A and/or B. When positive, culture on selective agar media was performed. Minimal inhibition concentration of vancomycin, metronidazole, tigecycline and rifampicin was then determined by gradient strip method (Etest®). There is no commercial method for testing susceptibility to fidaxomicin available.

**Results:** Interpretation criteria (breakpoints) are provided only for vancomycin and metronidazole. All the strains tested were susceptible to both antibiotics, the range of MIC was 0.064–2 mg/l in vancomycin and 0.064–1 mg/l in metronidazole. Therefore, several strains were approaching breakpoint and there is certainly potential of resistance in this cohort. It is not possible to interpret the susceptibility of tigecycline and rifampicin, but the MICs suggested possibility of low efficiency, especially with the range of 0.016–32 mg/l in rifampicin. The MICs overall were the lowest in tigecycline, 0.016–1 mg/l.

**Conclusions:** The established anti-CDI therapy regimen is appropriate, but it is important to investigate potential patterns of resistance. There are alternative antibiotic agents with activity against CLDI (the best results given by tigecycline), but further testing is crucial.

## P147

### Septic shock due to *C. canimorsus* treated with IgM-enriched immunoglobulin as adjuvant therapy in an immunocompetent woman

#### J Braunsteiner, S Siedler, D Jarczak, S Kluge, A Nierhaus

##### University Hospital Hamburg-Eppendorf, Department of Intensive Care, Hamburg, Germany

*Critical Care* 2023, **27(S1)**: P147

**Introduction:**
*Capnocytophaga canimorsus* is a facultative anaerobic Gram-negative bacillus and is part of the microflora of canine and feline oral cavities. *C. canimorsus* possesses active and passive mechanisms of immune evasion. Invasive infections are rare and have been primarily described in immunocompromised patients.

**Methods:** We report a case of a healthy, immunocompetent 51-year-old woman with purpura fulminans due to septic shock caused by *C. canimorsus* after having been scratched by cats.

**Results:** The patient was admitted to a community care hospital and treated with empirically targeted antibiotic therapy. 48 h after admission, *C. canimorsus* could be identified as causative pathogen, and her antibiotic regimen was adapted. She was referred to our department because of multi-organ dysfunction and refractory shock. The blood gas analysis showed hyperlactatemia with 17 mmol/l. The inflammatory markers were increased (WBC 16.2 G/l, procalcitonin 49.6 µg/l, CRP 247 mg/l, IL-6 3321 ng/l). Further analysis revealed pathologically low IgM levels (0.31 g/l). Adjuvant therapy with IgM-enriched immunoglobulin (Pentaglobin®) was initiated to restore normal values. The further course was complicated by acute respiratory failure, multiple regional infarctions, and recurrent septic episodes. IgM had decreased to 0.32 g/l on day 78, which was again treated with Pentaglobin®. After prolonged intensive care therapy, her condition improved, and she was transferred to a rehabilitation center.

**Conclusions:** Data on immunoglobulin therapy in sepsis is conflicting and there is a lack of evidence in patients with invasive *C. canimorsus* infection. However, following the administration of immunoglobulins the patient showed sustained clinical improvement. Patients with both a high inflammatory load and IgM deficiency might benefit from therapeutic IgM-administration.

**Acknowledgement:** We obtained informed consent from the patient to publish the clinical details. All participating physicians also consented to the publication.

## P148

### Sepsis after being licked in the face by a dog

#### M Bouwens, Y Acardag, JJ Arends

##### Albert Schweitzer Ziekenhuis, Intensive Care, Dordrecht, Netherlands

*Critical Care* 2023, **27(S1)**: P148

**Introduction:** We present a case where sepsis was caused by a dog licking its owners face. Sepsis is usually caused by pet bites.

**Methods:** Case report.

**Results:** A 58-year-old male presented at our hospital with severe sepsis. Patient history did not present any potential diagnostical clues. The patient owned a dog, but denied being bitten. He did not use any immunosuppressive medication. No focus for an infection was found during physical examination. Imaging studies were non-conclusive. He was admitted to the intensive care unit (ICU) with rapid progressive multi–organ failure and treated with mechanical ventilation, inotropes and broad–spectrum antibiotics. Initial blood and urine cultures remained negative. On day 8 of his ICU stay a 16S rRNA gene-sequencing test revealed an bacteremia with *Capnocytophagus canimorsus*, a slow–growing, gram–negative rod, present in dog or cat saliva and associated with pet bites [1]. Antibiotic treatment was adapted, and the patient made a quick recovery. Revision of the CT–images showed a small spleen (52.4 mm). Additional blood tests showed Howell-Jolly bodies, indicating functional asplenism. After cessation of mechanical ventilation, the patient admitted to letting his dog lick his face as a sign of friendship. The Dutch national guidelines for asplenic patients advice vaccination for capsulated bacteria and the use preventive antibiotics when being *bitten* by a pet [2].

**Conclusions:** Facial pet-licking can cause severe sepsis in functional asplenic humans.

Acknowledgement: Written consent for publication was obtained from this patient.


**References**
Brenner DJ et al. J Clin Microbiol. 1989;27:231–5.Dutch National Institute for Public Health and the Environment: https://lci.rivm.nl/richtlijnen/asplenie.


## P149

### Evaluation of risk factors associated with mortality in patients with ventilator-associated pneumonia caused by *Pseudomonas aeruginosa* with “difficult-to-treat” resistance

#### I Seises, M Rodríguez-Aguirregabiria, JM Añon-Elizalde, KL Nanwani-Nanwani, MJ Asensio-Martin

##### University Hospital La Paz, Intensive Care Medicine, Madrid, Spain

*Critical Care* 2023, **27(S1)**: **P149**

**Introduction:** Evaluation of prognostic factors in patients with ventilator-associated pneumonia (VAP) due to *P. aeruginosa*. The effectiveness of novel antipseudomonal antibiotics was reviewed.

**Methods:** Retrospective, single-center cohort analysis between April 2018 and June 2022. Data were obtained from the ENVIN-HELICS and electronic medical records. Demographic variables, underlying diseases and diagnosis to admission were registered. We considered each treatment appropriate according to Tamma PD et al. [1] criteria. We registered ventilator-associated tracheobronchitis (VAT) and pneumonia (VAP) episodes together with the recurrency of the infection.

**Results:** From 61 patients included, 77% were admitted for ARDS due to COVID-19. The mean APACHE-II was 14.3 ± 6.6. 7 patients required ECMO and 4 required RRT. The median length of stay in the ICU was 52 (ICR 36–84) days. 91 respiratory infections were recorded: 60 VAP and 31 VAT. On the first episode, carbapenem-resistance to meropenem was 40%; rising up to 58% on the second one. 6 patients developed a third episode (VAT) with a 100% of carbapenem-resistance. 13 (14%) respiratory infections showed resistance to the novel β-lactamase inhibitor cephalosporins (8 to ceftalozane-tazobactam and 5 to ceftazidime-avibactam). No resistance to cefiderocol was detected. During ICU stay, 21 patients (34%) developed secondary bacteremia from other foci and 7 (11%) invasive mycoses. Overall mortality was 49.2%. On the univariate analysis we found statistical significant relationships between mortality and COVID-19 admission, SOFA ≥ 7 points on the first VAP or the development of secondary bacteremia (Table 1).

**Conclusions:** COVID-19 admission, SOFA ≥ 7 points on the first VAP or other secondary bacteremia were associated with mortality. The 14.3% of respiratory infections were resistant to the new β-lactamase inhibitor cephalosporins. No resistance to cefiderocol was detected.


**Reference**
Tamma PD et al. Clin Infect Dis. 2022;75:187–212.

**Table Tabal:** **Table 1 (abstract P149)**. Univariate analysis of risk factors associated with ICU mortality

Charlson index ≥ 3	Alive 12 (37.5%)	Deceased 20 (62.5%)	OR 2.36	p 0.099
Admission due to severe COVID-19 pneumonia	Alive 19 (40.4%)	Deceased 28 (59.6%)	OR 3.68	p 0.41
SOFA-score ≥ 7 on the 1st episode of infection	Alive 10 (31.3%)	Deceased 22 (68.8%)	OR 4.18	p 0.007
ECMO therapy	Alive 1 (14.3%)	Deceased 6 (65.7%)	OR 6.46	p 0.061
Bacteremia by other microorganisms	Alive 5 (23.8%)	Deceased 16 (76.2%)	OR 4.80	p 0.007
Carbapenemase colonization prior to infection	Alive 5 (31.3%)	Deceased 11 (66.8%)	OR 2.51	p 0.129
Inappropriate treatment on first episode of infect	Alive 5 (33.3%)	Deceased 10 (66.7%)	OR 2.18	p 0.204
Resistance to new β-lactams with β-lactamase inhib	Alive 1 (20%)	Deceased 4 (80%)	OR 4	p 0.198

## P150

### Are new antibiotics efficient against DTR (difficult-to-treat resistance) isolates? Prevalence and susceptibility of invasive DTR strains

#### G Kroneislová, J Závora, V Adámková

##### General University Hospital Prague, Clinical Microbiology and ATB Center, Prague, Czech Republic

*Critical Care* 2023, **27(S1)**: P150

**Introduction:** Bacterial resistance surveillance is one of the main outcomes of microbiological laboratories and its results are an important part of antimicrobial stewardship (AMS). We point out a relatively new ‘difficult-to-treat’ (DTR) category of resistance, which—when used—has a significant influence on predictability of morbidity and mortality in patients with invasive infections.

**Methods:** The susceptibility of 100 unique isolates of the most common Gram-negative bacilli (*Enterobacterales*, *Pseudomonas aeruginosa*, *Acinetobacter baumannii*) obtained from blood cultures of patients hospitalised in General University Hospital in Prague in 2017–2021 was tested, 50% of these fulfilled DTR criteria (nonsusceptibility to all beta-lactams—incl. carbapenems—and fluoroquinolons) and 50% were susceptible.

**Results:** 10 *Enterobacterales* strains met DTR criteria and 20% of those were resistant to colistin (COL), 20% to cefiderocol (FCR), 70% to imipenem/cilastatin/relebactam (I/R), 30% to ceftazidime/avibactam (CAT), 50% to fosfomycin (FOS) and 50% tigecycline (TGC). For *Enterobacterales* we also tested aztreonam/avibactam (AZA) for which there are no breakpoints yet. The highest MIC of AZA observed was 0.75 mg/l, in the susceptible cohort the range was 0.023–0.064 mg/l and in the DTR cohort (incl. class B beta-lactamase producers) it was 0.047–0.75 mg/l. In *P. aeruginosa* (15 DTR strains) the resistance to COL were 13%, to FCR 6.6%, to I/R 86.6%, to CAT 46.6%, and to ceftolozane/tazobactam 26.6%. All DTR isolates of *A. baumannii* were susceptible to COL and FCR and resistant to I/R and ampicillin/sulbactam.

**Conclusions:** The cooperation of microbiologists and clinicians is crucial when treating severe infections, especially in the era of increasing antimicrobial resistance. Not even new antimicrobial agents are 100% efficient against DTR strains. Therefore, it is necessary to perform susceptibility testing of these antibiotics, use the data for surveillance (including local surveillance) and conform to AMS standards.

## P151

### Amoxicillin/clavulanate versus 3rd generation cephalosporins for community-acquired pneumococcal pneumonia: is it an arbitrary choice?

#### P Teixeira^1^, L Costa^2^, JG Pereira^3^, SM Fernandes^1^

##### ^1^Faculdade de Medicina da Universidade de Lisboa, Clinica Universitária de Medicina Intensiva, Lisboa, Portugal, ^2^Hospital de Braga, Braga, Portugal, ^3^Hospital de Vila Franca de Xira, Serviço de Medicina Intensiva, Hospital de VIla Franca de Xira, Portugal

*Critical Care* 2023, **27(S1)**: P151

**Introduction:** European guidelines recommend ceftriaxone or amoxicillin/clavulanate (AAC) as equally effective first-line antibiotic therapy for community-acquired pneumonia (CAP). This study aims to identify factors, including patients' clinical characteristics, that may influence the choice of beta-lactam empirical therapy for CAP and to relate this with patients' prognosis.

**Methods:** This is a sub-analysis of a retrospective multicentric cohort of pneumococcal CAP. Clinical characteristics and disease severity at admission were compared according to their initial empirical antibiotic therapy: ceftriaxone, AAC, or other. Mortality and days of hospitalization were compared between groups, adjusting for severity, age, and ICU admission. STATA v15.0 software was used for statistical analysis.

**Results:** We included 797 patients. 40.9% received ceftriaxone, 41.1% AAC, and 17.9% other therapy. Patients receiving ceftriaxone were more likely to be septic (61% vs 34% vs 48%, *p* < 0.001). Patients admitted to the hospital due to cardiovascular disease (8% vs 13% vs 6%, respectively; *p* = 0.01) and with hyponatremia (29.9 vs 76.8 vs 29.8%, *p* < 0.001) were more likely to receive AAC. Patients receiving “other” antibiotic therapy were less likely to receive corticosteroids or a macrolide. Age, comorbidities, multilobar pneumonia or pleural effusion did not influence empirical antibiotic choice. No differences in mortality were noted between different empirical therapy choices (non-adjusted: OR 0.70 (0.46–1.06), *p* = 0.07; adjusted for sepsis, hyponatremia, and age: OR 0.79 (0.5–1.2), *p* = 0.28). As expected, sepsis (OR: 1.6, *p* = 0.02) and age (OR:1.1, *p* < 0.001) were relevant risk factors for death.

**Conclusions:** The choice between empirical therapy for CAP does not appear to be random despite comparable outcome effect between first line antibiotics. This data may inform educational strategies on antibiotic stewardship.

## P152

### The combination of ceftazidime-avibactam plus meropenem can be a reliable therapeutic option for ventilator-associated pneumonia (VAP) due to pan-drug resistant *Klebsiella pneumoniae*

#### E Kourtelesi^1^, A Aiginitou^1^, C Merkouri^1^, M Poulou^1^, E Bourganie^1^, S Giannakaki^1^, E Dikoudi^1^, M Karagianni^1^, M Daganou^1^, A Flevari^2^

##### ^1^Sotiria Thoracic Diseases Hospital, Athens, Greece, ^2^Sotiria Thoracic Diseases Hospital, Polyvalent ICU, Athens, Greece

*Critical Care* 2023, **27(S1)**: P152

**Introduction:** Over the last two decades, the prevalence of pandrug-resistant *Klebsiella pneumoniae* (PDR-Kp) has increased worldwide. Such strains along with the burden of co-morbidities, especially in ICU patients, pose therapeutic challenges and contribute to high mortality rates. Combination of antimicrobial drugs is one of the few options available to overcome this challenge. Among others the combination of ceftazidime-avibactam (CZA) plus meropenem (MEM) has been proposed to be effective and safe [1].

**Methods:** A prospective single cohort study was conducted from March to August 2022. Among 50 patients admitted to our General ICU, 8 patients were diagnosed with VAP due to PDR-Kp. After clinical suspicion, VAP diagnosis was performed from microbiological and clinical tools, namely positive tracheal aspirate cultures (≥ 10^5^ cfu/ml) and CPIS score ≥ 6. Kp was considered PDR if microbiological tests revealed resistance to cephalosporins, carbapenems, monobactams, quinolones, sulfamethoxazole-trimethoprim, aminoglycosides, colistin, tigecycline, fosfomycin and ceftazidime/avibactam. If patients were haemodynamic stable, with no need of vasopressors, combination therapy CZA-MEM was introduced. Primary outcome was the resolution of pneumonia and secondary outcome was 30-day mortality.

**Results:** Table 1 tabulates clinical characteristics of the population enrolled. Among 8 patients (3 female) with PDR-Kp associated VAP, who received the combination of CZA-MEM, all showed signs of clinical resolution of pneumonia. CPIS score reduction after 8–10 days of therapy was statistically positive (*p* < 0.001). Tracheal aspirate cultures, who were subsequently obtained, showed microbiological eradication of the specific strain. All patients tolerated therapy with no adverse events. 30-day mortality was 25% (2/8).

**Conclusions:** In the era of multidrug resistance the synergistic activity of MEM plus CZA against PDR-Kp can be an efficient therapeutic option in critically ill patients.


**Reference**
Michail S et al. Antimicrob Agents Chemother 2019;63:e00779–19.

**Table Tabam:** **Table 1 (abstract P152)**. Clinical characteristics of patients with VAP due to PDR/Kp

	Mean	SD
Age (years)	66.38	9.4
Length of ICU stay (days)	41.38	29
APACHE II on admission	21.88	7.1
SOFA score on admission	6.50	3
CHARLSON comorbidity index	5.75	3.9

## P153

### Model-informed precision dosing of beta-lactam antibiotics and ciprofloxacin in critically ill patients

#### TMJ Ewoldt^1^, A Abdulla^2^, WJR Rietdijk^2^, AE Muller^3^, BCM De Winter^2^, NGM Hunfeld^1^, D Gommers^1^, H Endeman^1^, BCP Koch^2^

##### ^1^Erasmus University Medical Center, Intensive Care Medicine, Rotterdam, Netherlands, ^2^Erasmus University Medical Center, Hospital Pharmacy, Rotterdam, Netherlands, ^3^Erasmus University Medical Center, Microbiology, Rotterdam, Netherlands

*Critical Care* 2023, **27(S1)**: P153

**Introduction:** Individualising drug dosing using model-informed precision dosing (MIPD) of beta-lactam antibiotics and ciprofloxacin has been proposed as an alternative to standard dosing to optimise antibiotic efficacy in critically ill patients. However, randomised clinical trials (RCT) on clinical outcomes have been lacking.

**Methods:** This multicentre RCT, including patients admitted to the ICU who were treated with antibiotics, was conducted in eight hospitals in the Netherlands [1]. Patients were randomised to MIPD with dose and interval adjustments based on monitoring serum drug levels (therapeutic drug monitoring) combined with pharmacometric modelling of beta-lactam antibiotics and ciprofloxacin (Fig. 1). The primary outcome was ICU length of stay (LOS). Secondary outcomes were ICU mortality, hospital mortality, 28-day, and 6-month mortality, delta sequential organ failure assessment (SOFA) score, adverse events, and target attainment.

**Figure Figbe:**
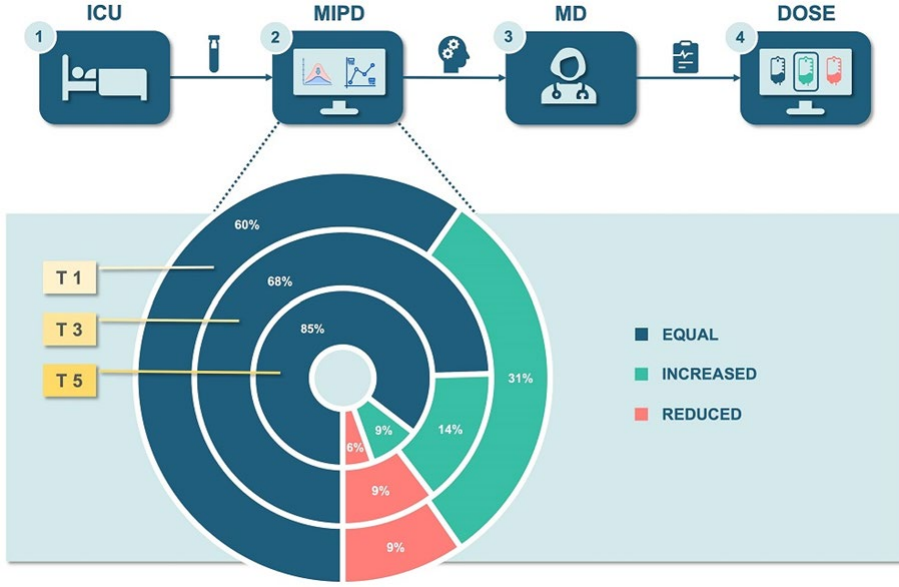
**Fig. 1 (abstract P153)**. Study flow and dose advice given

**Results:** In total, 388 (MIPD n = 189; standard dosing n = 199) patients were analysed (median age 64 [IQR 55–71]). In the intervention group a dose adjustment was recommended for 71 (37.6%), 27 (23.1%), and 8 (13.3%) patients at T1, T3, and T5, respectively. We found no significant differences in ICU LOS between MIPD compared to standard dosing (10 MIPD vs 8 standard dosing; IRR = 1.16; 95% CI 0.96, 1.41; *p* = 0.13). There was no significant difference in target attainment before intervention at day 1 (T1) (55.6% MIPD vs 60.9% standard dosing; *p* = 0.24) or at day 3 (T3) (59.5% vs 60.4%; *p* = 0.84). There were no significant differences in other outcomes.

**Conclusions:** We could not show a beneficial effect of MIPD of beta-lactam antibiotics and ciprofloxacin on ICU LOS in all critically ill patients. Maximal dosing according to the summary of product characteristics was often insufficient to reach target attainment. Our data highlights the need to identify other approaches to dose optimization, including earlier optimisation and patient selection.


**Reference**
Ewoldt TMJ et al. Intensive Care Med. 2022;48:1760–1771.


## P154

### Beta-lactam concentrations in patients with sepsis treated in the ICU

#### E Kryss^1^, K Liljedahl Prytz^2^, M Sundqvist^3^, J Oxelbark^4^, K Nilsson^5^, J Källman^6^, J Savilampi^5^

##### ^1^Institution of Medical Sciences, Örebro, Sweden, ^2^Institution of Medical Sciences, Örebro Univerisity, Örebro, Sweden, ^3^Institution of Medical Sciences, Clinical Microbiology, Örebro, Sweden, ^4^Institution of Medical Sciences, Clinical Chemistry, Örebro, Sweden, ^5^Institution of Medical Sciences, Anaesthesiology and Intensive Care, Örebro, Sweden, ^6^Institution of Medical Sciences, Infectious Diseases, Örebro, Sweden

*Critical Care* 2023, **27(S1)**: P154

**Introduction:** Sepsis pathophysiology may lower plasma concentrations of antibiotics thus reducing antibacterial effects. Optimal dosing of beta-lactam antibiotics in septic patients remains challenging [1]. Time above minimal inhibitory concentration (MIC) is the determinant of beta-lactam antibiotic efficacy. Plasma concentration from 1 to 4 × MIC for 100% of the dosing interval has been suggested as target level. Repeated measurement of plasma concentrations are proposed to avoid insufficient concentrations of beta-lactams in critically ill patients. The aim was to describe plasma concentration achievement of beta-lactams in septic patients during the first 48 h after admittance to the ICU.

**Methods:** A prospective, observational study included adults with community-acquired sepsis admitted from the emergency department to the ICU. Antibiotic plasma concentration measurements during administration of piperacillin-tazobactam [PTZ, 4 g× 3–4], cefotaxime [CTX, 2 g× 3] and benzyl-penicillin [PEN, 3 g× 3]) were collected at 2–7 occasions/patient and analysed using liquid chromatography-mass-spectrometry. To evaluate time above MIC, theoretical MICs representing the EUCAST I breakpoints were used for bacteria common in community acquired pathogens.

**Results:** Out of the thirty-two patients included, 56% were men (18/32), with median age of 71 (min–max 28–85) and with median SOFA score of 7 (min–max 3–13). They were either treated with PTZ (n = 23), PEN (n = 4) or CTX (n = 11). Seven patients had an altered antibiotic regimen during the 48 h, resulting in a total of 169 plasma concentration measurements. The percentage of plasma concentrations above MIC × 1/ × 4 during the 48 h was 97/89% for PTZ (n = 113), 91/73% for PEN (n = 11) and 98/76% for CTX (n = 45) (Fig. 1).

**Conclusions:** In septic patients receiving standard dosing of CTX, PTZ or PEN, plasma concentrations had a large variation but most were above effective concentrations.


**Reference**
Petersson J et al. Acta Anaesthesiologica 2016;60:1425–1436.


**Figure Figbf:**
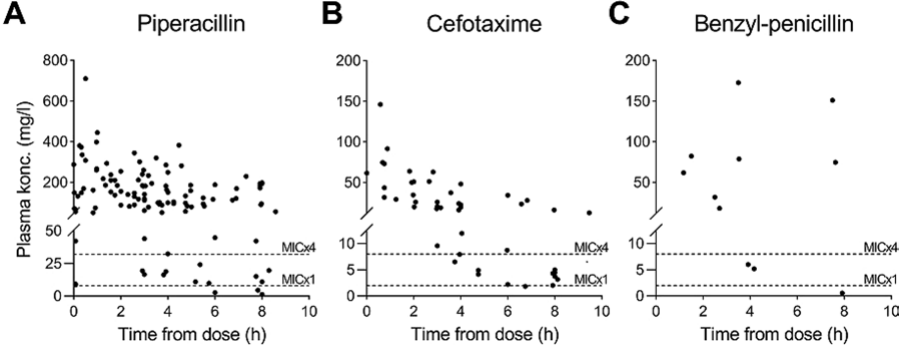
**Fig. 1 (abstract P154)**. Plasma concentrations of beta-lactams (**A**: PTZ [n = 113],** B**: CTX [n = 45] and** C**: PEN [n = 11]), related to time from antibiotic dose to blood sampling. Concentrations were compared to the clinical susceptibility breakpoints for *Enterobacteriaceae* (PTZ 8 mg/l and CTX 2 mg/l) and *S. pneumoniae* (PEN 2 mg/l). These breakpoints are the highest MICs for pathogens considered susceptible if increased exposure for these antibiotics. Target plasma concentration levels are shown in graphs as MICx1 and MICx4

## P155

### A photo finish of machine learning models for predicting beta-lactam target non-attainment in ICU patients

#### A Wieringa^1^, TMJ Ewoldt^2^, R Gangapersad^3^, M Gijsen^4^, A Abdulla^3^, BCP Koch^3^

##### ^1^Isala, Dept. of Clinical Pharmacy, Zwolle, Netherlands, ^2^Erasmus University Medical Center, Department of Hospital Pharmacy and Intensive Care, Rotterdam, Netherlands, ^3^Erasmus University Medical Center, Department of Hospital Pharmacy, Rotterdam, Netherlands, ^4^KU Leuven and UZ Leuven, Department of Pharmaceutical and Pharmacological Sciences, Leuven, Belgium

*Critical Care* 2023, **27(S1)**: P155

**Introduction:** In the ICU, the infection-related mortality is high (30%) and beta-lactam target non-attainment (TNA) occurs in 37% of ICU patients [1, 2]. The recommended pharmacodynamic target for beta-lactam antibiotics is 100% of the time above the minimal inhibitory concentration [1]. Antibiotic dose optimization can be performed with therapeutic drug monitoring (TDM). In this study we therefore developed prediction models for TNA in ICU patients receiving beta-lactams.

**Methods:** Data of the Dolphin and Expat studies were used to train and internally validate three machine learning models to predict TNA on the day after antibiotic initiation [2]. After excluding patients on dialysis, 378 patients remained. For covariate selection, the Boruta algorithm was used in the training set and afterwards manually verified by adding and removing covariates. A random forest (RF), logistic regression (LR) and naïve bayes (NB) model were developed and externally validated with data from UZ Leuven and Amsterdam UMC (150 patients). Model performance was evaluated using a 1000-times cross-validation. The main performance metric used was the area under the receiver operator curve (AUROC).

**Results:** The covariates age, sex, serum creatinine and type of antibiotic were found to be TNA predictors. In the internal validation, the NB, LR and RF models showed an AUROC of 0.82 [0.74, 0.91], 0.81 [0.76, 0.91] and 0.81 [0.75, 0.91], respectively. In the external validation, the models showed an AUROC of 0.75 [0.67, 0.82], 0.80 [0.73, 0.87] and 0.79 [0.72, 0.86], respectively (Fig. 1 including other metrics).

**Conclusions:** All models showed good performance in both validation sets using four readily available covariates. These models can therefore be easily implemented in daily clinical practice to identify patients at risk of TNA, in which TDM of beta-lactams is indicated, hence allowing more efficient use of limited hospital resources.

## References


Abdul-Aziz MH et al. Intensive Care Med 2020;46:1127–1153.Abdulla A et al. Crit Care 2020;24:558–569.

**Figure Figbg:**
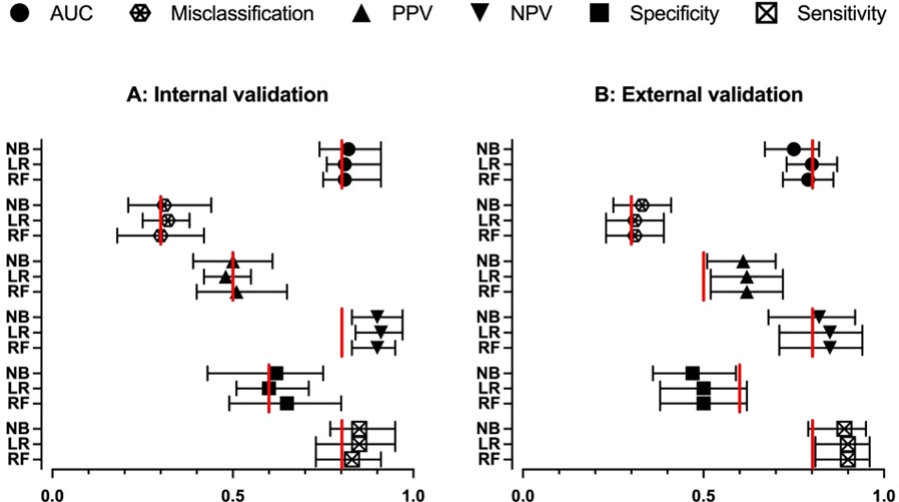
**Fig. 1 (abstract P155)**. Performance metrics for the target non-attainment models in the internal validation set (**A**) and the external validation set (**B**). NB: naïve bayes; LR: logistic regression; RF: random forest; AUC: Area under the receiver operator curve; PPV: positive predictive value; NPV negative predictive value; Red line: The chosen threshold for each metric

## P156

### How many vancomycin levels are required for PK/PD dosing recommendation using Bayesian dose-optimizing software in critically ill patients?

#### ND Dreyse, P Baeza, N Salazar, R Lopez

##### Clínica Alemana de Santiago, Paciente Crítico, Santiago, Chile

*Critical Care* 2023, **27(S1)**: P156

**Introduction:** The aim of this study was to determine of the quantity of vancomycin levels be required for PK/PD dosing recommendation using Bayesian dose-optimizing software with more accuracy and less bias. The trearment guidelines recommend an individualized target of AUC/MIC 400–600, assuming MIC 1 mg/l. However, most programs performing Bayesian estimation require only one vancomycin level. Currently no data suggest how many vancomycin levels should be collected for the correct estimation AUC/MIC in critically ill patients.

**Methods:** In this prospective study performed in the ICU of Clinica Alemana de Santiago, patients receiving vancomycin as part their care was enrrolled. Vancomycin levels were taken at the end-infusion (peak), 120 min following the end-infusion (beta) and immediately before the next dose (trough). The AUC_REF_ was calculated by linear-long trapezoidal rule using all vancomycin levels taken. The Bayesian software used was PrecisePK™, PreciseRX, San Diego, USA. Five different AUCs were obtained by PrecisePK™ as follow: 1.- peak, beta and trough; 2.- beta and trough; 3.- peak and trough; 4.- trough; 5.- AUC obtained PrecisePK™ without vancomycin level. Accuracy was defined as the median ratio of the estimated AUC to AUC_REF_. Bias was defined as the median of the absolute value of the percentage difference of the estimated AUC from AUC_REF_. Then each AUC obtained by PrecisePK™ was comapred with AUC_REF_ using Mann–Whitney *U* Test. *p* value was fixed at < 0.05.

**Results:** Thirty-six patients were inclued. In general, no significant differences were observed between five ways to approach estimated AUC. However, the most accurate and the least biased estimated AUC was using peak and trough.

**Conclusions:** The findings of this study showed that the critically ill patients Bayesian dose-optimizing program using peak and trough produce estimated AUC more accurate and with less bias. These results give an empirical basis to support the recommendations of current clinical guidelines.

## P157

### Aminoglycoside dosing in intensive care: are we getting it right?

#### F Mohideen, A Collins, L Coslett, A Conway Morris, D Sapsford

##### Addenbrooke’s Hospital, John V Farman Intensive Care Unit, Cambridge, UK

*Critical Care* 2023, **27(S1)**: P157

**Introduction:** Aminoglycosides (AMGs) are extensively used in intensive care units (ICUs) to treat gram-negative bacterial infections, yet there is much variation in practice to ensure optimal dosing. Adverse effects are often associated with sustained trough concentrations and include neuromuscular weakness, ototoxicity and nephrotoxicity, with some studies reporting the latter in > 58% of all ICU patients receiving AMGs [1]. Therefore, we investigated the AMG prescribing practices in the ICU of a large tertiary centre in the UK.

**Methods:** All ICU patients prescribed AMGs were identified from March 2021 to March 2022, excluding indications for infective endocarditis and medical or surgical prophylaxis. A retrospective review of patients’ clinical notes alongside the hospital’s AMG guideline was performed to assess prescribing and protocol compliance as a registered service evaluation.

**Results:** 228 patients were identified with 136 receiving gentamicin (60%) and 92 receiving amikacin (40%). 159 patients received a single dose, where 94 (59%) of these did not have a subsequent trough level taken in case of requirement for further doses. 69 patients (30%) received multiple doses. Prescription errors were identified in 28 of these (41%). Errors included subsequent doses being given too early (< 24 h) in 43% (12/28), without a confirmed trough level in 21% (6/28) or a combination of both in 21% (6/28). Moreover, 14% of these patients (4/28) received a subsequent dose despite a high trough level.

**Conclusions:** There was a risk of significant harm for a small number of patients receiving multiple doses. ICU-specific AMG guidelines need to be developed in line with current evidence and a safety alert will be added onto the prescribing system. Re-audit following these interventions is planned alongside engagement of ICUs from other centres to promote collaborative learning and service improvement.


**Reference**
Oliveira JF et al. Antimicrob Agents Chemother 2009;53:2887–91.


## P158

### Concentration target attainment after therapeutic drug monitoring of linezolid in critically ill patients

#### J Langui^1^, P De Cock^1^, N Verougstraete^2^, J Boelens^2^, A Somers^1^, J De Waele^3^, B Claus^1^

##### ^1^Ghent University Hospital, Pharmacy Department, Ghent, Belgium, ^2^Ghent University Hospital, Department of Laboratory Medicine, Ghent, Belgium, ^3^Ghent University Hospital, Department of Intensive Care Medicine, Ghent, Belgium

*Critical Care* 2023, **27(S1)**: P158

**Introductions:** Linezolid (LZD) is often used to treat severe Gram-positive infections. In recent years, pharmacokinetic variability of LZD has been described in critically ill patients. The aim of this study was to analyse LZD exposure in critically ill patients monitored with TDM.

**Methods:** A retrospective observational study of all adult intensive care unit (ICU) patients receiving LZD for at least three days was conducted between August 2021 and February 2022 (Ethics Committee BC-11209). According to the institutional protocol at the ICU of the Ghent University Hospital, TDM of LZD is recommended in patients with augmented renal clearance, renal/hepatic impairment, low baseline platelet count, sepsis/septic shock and/or expected treatment duration > 14 days [1]. Decision to monitor is at the discretion of the treating physician. Target attainment, defined as a trough concentration between 2 and 7 mg/L, was the primary endpoint and TDM-guided dose adjustments were descriptively analysed.

**Results:** Of the 47 patients treated with LZD during the study period, TDM was performed in 14 patients (29.8%), all meeting the criteria. Noteworthy, 90.9% of the patients without monitoring were eligible for TDM as well. Intra-abdominal infections were the most common infection (42.6%). Median treatment duration of LZD in ICU was 5.7 [3.7–8.5] days for all patients. Therapeutic range was reached at first measurement in 9 of 14 (65.0%) patients. After TDM-guided dose adjustments, a total of 11/14 (78.6%) patients were within therapeutic range after a median of 2.6 [2.5–4.5] days.

**Conclusions:** Two thirds of the TDM patients reached therapeutic concentrations at first measurement and an extra 13.6% patients after dose adjustment. More patient data are needed to refine the criteria for monitoring critically ill patients most at risk for target non-attainment and to draw more robust conclusions.


**Reference**
Rao GG et al. Ther Drug Monit. 2020;42:83–92.


## P159

### Meropenem model-informed precision dosing (MIPD) in the treatment of critically ill patients: which population pharmacokinetic model to choose?

#### L Li^1^, S D.T. Sassen^1^, T M.J. Ewoldt^2^, A Abdulla^1^, N G.M. Hunfeld^2^, A E. Muller^3^, B C.M. de Winter^1^, H Endeman^2^, B C.P. Koch^1^

##### ^1^Erasmus Medical Center, Pharmacy, Rotterdam, Netherlands, ^2^Erasmus Medical Center, Intensive Care, Rotterdam, Netherlands, ^3^Erasmus Medical Center, Medical Microbiology and Infectious Diseases, Rotterdam, Netherlands

*Critical Care* 2023, **27(S1)**: P159

**Introductions:** Population pharmacokinetic (PopPK) studies of meropenem in intensive care unit (ICU) patients have shown large differences in estimated pharmacokinetic (PK) parameters, making it difficult to select an appropriate model for clinical use. We performed external validation using real-world ICU patient data to assess the suitability of published meropenem models for clinical application in initial dosing and therapeutic drug monitoring (TDM) based dose adjustments.

**Methods:** All these models were replicated in NONMEM® 7.4. The predictability of the models was evaluated using a dataset collected from 20 ICU patients. The ability of the models to estimate the starting dose and the ability to adjust towards the target concentration based on previous concentrations was evaluated using prediction-based and simulation-based diagnostics.

**Results:** 18 parametric PopPK models were included for assessment. For initial dosing prediction evaluation with the patients’ characteristics only, neither the GOF plot nor VPC plot fit well, except for 3 models. The predictive power of all models was low, with prediction error (PE)% of the interquartile range (IQR) exceeds ± 30% threshold (Fig. 1). For TDM-based dose adjustment assessment, the model fit improved after accounting for measured concentrations in the GOF plot. When incorporating 1–3 concentrations into five selected models, two models had an IQR PE% within ± 30%, when 2 or 3 concentrations were utilized.

**Conclusions:** Pharmacokinetic parameters from controlled ICU trial should not be used to guide initial dosing in real-world patients. On the other hand, certain models are suitable for TDM based dose adjustment with at least two or more samples incorporated.

**Figure Figbh:**
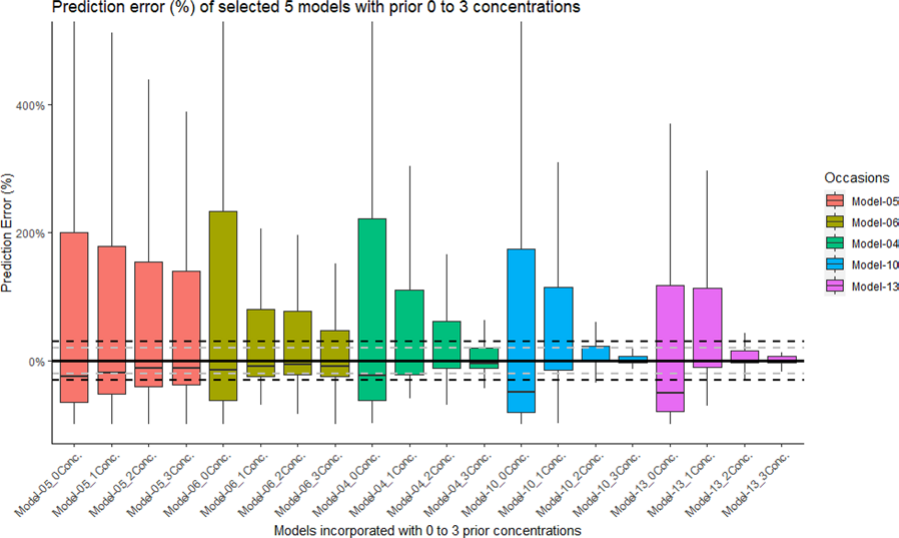
**Fig. 1 (abstract P159)**. Prediction error of 5 models with prior 0–3 concentrations

## P160

### COVID-19 patients are at greater risk of developing fungal infections with prolonged course of steroids and invasive ventilation in intensive care unit (ICU)

#### J Ainsworth, P Sewell, S Eggert, S Pillai

##### Morriston Hospital, Ed Major Intensive Care Unit, Swansea, UK

*Critical Care* 2023, **27(S1)**: P160

**Introduction:** COVID-19 pandemic infection has affected over 650 million people with over 6 million deaths. Critically unwell patients are at increased risk of developing invasive fungal infections [1]. The aim of this study was to identify the number of patients admitted to ICU with COVID-19 who developed fungal infections and to compare these patients (fungal group) with those without fungal infections (non-fungal group) to investigate which factors may have contributed to increased risk of infection.

**Methods:** Retrospective study undertaken in a tertiary teaching hospital ICU. 174 patients admitted with severe COVID-19 infection during March 2020 until May 2021 were included.

**Results:** 81(47%) patients developed fungal infections of which 94% had Candida and 6% had Aspergillus infection. Age and smoking history did not appear to be a contributing factor. The non-fungal group had significantly higher body mass index (33 ± 8 vs 31 ± 7, *p* = 0.01). ICU length of stay [23(1–116) vs 8(1–60), *p* < 0.001], hospital length of stay [30(3–183) vs 15(1–174) ± 7, *p* < 0.001], steroid days [10(1–116) vs 4(0–28), *p* = 0.02] and ventilation days [18(0–120) vs 2(0–55), *p* < 0.001] were significantly higher in the fungal group. The mortality rate in both groups were similar (51% vs 51.6%).

**Conclusions:** Fungal infections are extremely common in COVID-19 patients admitted to ICU, seen in almost half of patients in this cohort (47%). Longer treatment with corticosteroids appears to increase the risk of developing fungal infections. Increased length of ICU stay, and a greater length of mechanical ventilation significantly increase the risk of fungal infections in COVID-19 patients in intensive care. Fungal infection, however was not associated with increase in mortality.


**Reference**
Coşkun AS. et al. Polish J Microbiol 2021;70:395–400.


## P161

### Candida colonization and mortality in an ICU: is candida colonization a poor prognosis factor in the critical patient?

#### RV Jorge^1^, A Carvalho^2^, A Fernandes^3^, J Patrício^4^, P Patrício^4^, T Nascimento^5^

##### ^1^Hospital Beatriz Ângelo, Intensiva Medicine Department, Loures, Portugal, ^2^Hospital da Sra. da Oliveira, Guimarães, Portugal, ^3^Hospital Beatriz Ângelo, Internal Medicina Department, Loures, Portugal, ^4^Hospital Beatriz Ângelo, Loures, Portugal, ^5^Instituto de Higiene e Medicina Tropical/Universidade Nova de Lisboa(IHMT/UNL)/Instituto Universitário Egas Moniz, Lisboa, Portugal

*Critical Care* 2023, **27(S1)**: P161

**Introduction:** Candida colonization in hospitalized patients may predispose to the establishment of severe infections namely in the critical patient. The aim of this prospective study was to evaluate if the patients admitted in the ICU who are Candida colonized have higher mortality associated rates.

**Methods:** A total of 497 patients (177 female, 320 male; age range: 12–92 years, mean age: 64) who were in ICU of Beatriz Ângelo Hospital (Lisbon, Portugal) between September, 2021 and September, 2022 were included in the study. After admission and consent, axillary and groin region cultures have been collected in D1, D5 and D8 of staying. Isolation and identification of Candida strains were performed by using conventional mycological methods. Patient records were used to describe and compare the demographic, health indicators and risk factors of the screened. The primary end point was 3, 6 and 12 months all-cause mortality, when available. Multiple logistic regression analysis was then applied.

**Results:** In our study, yeast growth was detected in 137 patients (27.6%) and *C. albicans* (72.3%) and *C. parapsilosis* (43.1%) were the most common. 21.9% were mixed cultures and *C. glabrata* was isolated in 13.9% of the positive cultures. Our results have indicated a statistically significant relationship between colonization by *C. albicans* (odds ratio 1.864; 95% CI 1.133–3.068) and *C. glabrata* (odds ratio 3.627; 95% CI 1.312–10.022) with 3-month mortality. Also, patients with anemia and mechanic ventilation are more likely to be candida colonized. however, the correlation between candida colonization and the presence of other underlying diseases, invasive procedures, parenteral nutrition, haemodialysis, surgery and use of extended-spectrum antibiotics was not statistically significant.

**Conclusions:** These findings could imply candida colonization as a poor prognosis factor in the critical patient. Further analysis and high-scale studies are needed to support our data.

## P162

WITHDRAWN.

## P163

### Characterization of secondary bacterial and fungal infections in ICU hospitalized patients with COVID-19: retrospective observational study

#### AR Rutins^1^, EK Kalnins^2^, SK Kazune^3^, ZS Smatcenko^4^

##### ^1^ iga Stradins University, Faculty of Residency, Riga, Latvia, ^2^University of Latvia, University of Latvia- Residency Studies, Riga, Latvia, ^3^Riga Stradins University, Riga, Latvia, ^4^Riga East University Hospital, Toxicology and Sepsis Department, Riga, Latvia

*Critical Care* 2023, **27(S1)**: P163

**Introduction:** We aimed to describe the incidence, risk factors, and clinical outcomes of bacterial and fungal co-infections and superinfections in intensive care patients with COVID-19 in a retrospective observational study.

**Methods:** A retrospective cohort of intensive care patients with confirmed SARS-CoV-2 by PCR was analysed from January to March 2021. This was contrasted to a control group of influenza-positive patients admitted during 2012–2022. Patient demographics, microbiology and clinical outcomes were analysed.

**Results:** A total of 70 patients with confirmed SARS-CoV-2 were included; 6 (8.6%) of 70 had early bacterial isolates identified rising to 42 (60%) of 70 throughout admission. Blood cultures, respiratory samples, and urinary samples were obtained from 66 (94.3%), 18 (25.7%) and 61 (87.1%) COVID-19 patients. Positive blood culture was identified in 13 patients (18.6%). Bacteraemia resulting from respiratory infection was confirmed in 3 cases (all ventilator-associated). Line-related bacteraemia was identified in 9 patients (6 *Acinetobacter baumannii*, 4 *Enterococcus* spp. and 1 *Pseudomonas aeruginosa* and 1 Micrococcus lylae). No concomitant pneumococcal, *Legionella* or influenza co-infection was detected. Invasive fungal infections with *Aspergillus* spp. were identified in 2 cases. Pneumococcal coinfections (7/68; 10.3%) were identified in the control group of confirmed influenza infection; clinically relevant bacteraemias (6/68; 8.8%), positive respiratory cultures (15/68; 22.1%). The rate of hospital-acquired infections was 51.4% for COVID-19 and 27.9% for influenza. Longer intensive care stay, type 2 diabetes, obesity and hematologic diseases were independent risk factors for superinfections in the COVID-19 cohort.

**Conclusions:** Respiratory coinfections occurred in influenza but not in COVID-19 patients. The rate of hospital-acquired infections (51.4% for COVID-19; 27.9% for influenza) was unexpectedly high in both groups.

## P164

### Efficacy and safety of rezafungin in critically ill patients with candidaemia and/or IC: Outcomes of patients treated in the ICU from an integrated analysis of Phase 2 and Phase 3 trials

#### P.M. Honoré^1^, M. Kollef^2^, G.R. Thompson^3^, A. Soriano^4^, O.A. Cornely^5^, C Dignani^6^, N Manamley^7^, T Sandison^8^, PG Pappas^9^, M Bassetti^10^, On behalf of the ReSTORE trial investigators

##### ^1^UCLouvain Medical School, Intensive Care Unit Department, Brussels, Belgium, ^2^Washington University, St. Louis, USA, ^3^University of California Davis Medical Center, Davis, USA, ^4^Hospital Clínic de Barcelona, IDIBAPS, University of Barcelona, Barcelona, Spain, ^5^University of Cologne, Cologne, Germany, ^6^PSI-CRO, Durham, NC, USA, ^7^Mundipharma Research Ltd, Cambridge, UK, ^8^Cidara Therapeutics, Inc., San Diego, CA,, USA, ^9^University of Alabama at Birmingham, Birmingham, AL, USA, ^10^University of Genoa, Genoa, Italy

*Critical Care* 2023, **27(S1)**: P164

**Introduction:** Critically ill patients are susceptible to invasive candidiasis (IC), with approximately one-third of all candidaemia (C) cases occurring in the intensive care unit (ICU) [1]. This post-hoc, integrated analysis of STRIVE (Phase 2; NCT02734862) and ReSTORE (Phase 3; NCT03667690) investigated the efficacy and safety of rezafungin (RZF), a novel, once-weekly (QW) echinocandin, in a subgroup of patients with C and/or IC in the ICU.

**Methods:** Patients received RZF QW (400 mg in Week 1, then 200 mg) or caspofungin (CAS) once daily (70 mg on Day [D] 1, then 50 mg with optional oral fluconazole stepdown) for 2‒4 weeks. The primary endpoint (U.S. FDA) was all-cause mortality (ACM) at D30. Other endpoints included mycological eradication at D5 and D14 (secondary) and time to negative blood culture (TTNBC; exploratory). Safety was evaluated by adverse events (AEs).

**Results:** Baseline patient characteristics were generally similar between the RZF (n = 55) and CAS (n = 71) arms; 32.1% (17) and 29.6% (21) of patients receiving RZF and CAS, respectively had an acute physiologic assessment and chronic health evaluation II score ≥ 20. ACM at D30 was 36.4% (20/55) with RZF and 26.8% (19/71) with CAS. Through Day 30, 4 (7.3%) deaths were attributable to C/IC in the RZF arm and 6 (8.5%) in the CAS arm. Mycological eradication at D5 and D14 and the proportion of patients with negative blood culture at 24 h and 48 h are shown in Table 1. Median TTNBC was 18 h with RZF versus 38 h with CAS (stratified log-rank *P* = 0.003; not adjusted for multiplicity). Safety profiles were similar between treatment arms; the incidence of treatment-emergent AEs was 90.9% (50/55) with RZF and 81.7% (58/71) with CAS.

**Conclusions:** RZF is efficacious and well tolerated in critically ill patients with C and/or IC, with high rates of mycological eradication observed early in treatment. This integrated dataset provides additional insights into RZF efficacy and safety in the critical care population.


**Reference**
Logan C et al. Intensive Care Med 2020;46:2001‒2014.

**Table Taban:** **Table 1 (abstract P164)**. Efficacy outcomes in RZF and CAS arms

Efficacy outcomes of patients	RZF (n = 55) 400/200 mg QW	CAS (n = 71) 70/50 mg once daily
Negative blood culture; 24 h, n/N (%)	25/41 (61.0)	22/52 (42.3)
Negative blood culture; 48 h, n/N (%)	30/40 (75.0)	29/52 (55.8)
Mycological eradication; D5, n/N (%)	40/55 (72.7)	41/71 (57.7)
Mycological eradication; D14, n/N (%)	37/55 (67.3)	45/71 (63.4)

## P165

### Renal safety of liposomal amphotericin B in critically ill mechanically ventilated patients with SARS-CoV-2 pneumonia

#### Á Estella^1^, I Blanco^1^, M Gracia Romero^2^, A Garrino^1^, M Recuerda Nuñez^1^

##### ^1^University Hospital of Jerez, University of Cadiz, INIBiCA, Intensive Care Unit University Hospital of Jerez, Jerez de la Frontera, Spain, ^2^University Hospital of Jerez. University of Cadiz, INIBiCA, Intensive Care Unit, Anatomy Department, Jerez de la Frontera, Spain

*Critical Care* 2023, **27(S1)**: P165

**Introduction:** Liposomal amphotericin B (L-AmB) represent a good treatment strategy for critically ill patients according to its unique pharmacological characteristics and a relatively broad spectrum of action. The aim of the present study is to asses the impact on renal function of L-AmB during the first days of ICU admission in critically ill patients.

**Methods:** Retrospective, single-center case series of patients with SARS-CoV-2 pneumonia admitted in ICU. Setting: 19-bed medical-surgical ICU of a community hospital. Time of study: 2 years. Study variables: APACHE II and SOFA at admission,clinical characteristics, oliguria and creatinine level at admission and 72 h after L-AmB treatment were recorded. Oliguria was defined as urinary output less than 400 ml per day or less than 20 ml per hour. Two groups of patients were selected according to whether or not they received anticipated antifungal treatment pending microbiologic confirmation or discarding of aspergillosis; dosage of L-AmB was 3 mg/kg/d. Statistical analysis: Data were analyzed by SPSS 18 and quantitative variables were expressed as a mean ± standard deviation.

**Results:** 160 patients were included, 102 who received 3 days of anticipated treatment with L-AmB at ICU admission or at the beginning of mechanical ventilation were compared with patients without this treatment. There were not differences in age, median 65 [57–71] years, gender with 28% female and BMI (kg/m^2^), 30,4 [26,6–33,2]. APACHE II at admission was higher in the treated group of patients 17 [12–23] vs 12 [9–14]. SOFA was 7 [4–8] in the treated group of patients vs 6 [3–8]. There were not differences in urinary output between groups during the three first days of ICU stay. Table 1 shows creatinine levels.

**Conclusions:** According to our retrospective analysis, L-AmB is safe in the first days of treatment in critically ill patients admitted in ICU requiring mechanical ventilation.

**Table Tabao:** **Table 1 (abstract P165)**. Creatinine levels according use of liposomal amphotericin B at ICU admission and at third day of treatment

Variable	Overall (n = 160)	Not anticipated antitifungal treatment (n:58)	Anticipated antitifungal treatment (n = 102)	*p*
Admission to ICU: Creatine (mg/dl)	0.9 [0.7–1.2]	0.9 [0.7–1.2]	0.8 [0.7–1.1]	0.793
Third day to admission to ICU Creatine (mg/dl)	0.9 [0.7–1.2]	0.9 [0.7–1.3]	0.9 [0.7–1.2]	0.776

## P166

### The immunological aspects of the efficiency of prevention of sepsis with inhaled nitrogen oxide in newborns on mechanical ventilation

#### M Pukhtinskaya

##### State Medical University, Rostov-on-Don, Department of Anesthesiology and Critical Care Medicine, Rostov on Don, Russian Federation

*Critical Care* 2023, **27(S1)**: P166

**Introduction:** It is known, that the molecule NO—is fundamental factor of anti-infectious resistance of an organism. Research objective: evaluate the efficacy and safety of inhalation nitrogen oxide (iNO) to prevent sepsis.

**Methods:** The controlled, randomized, blind clinical trial included 97 full-term newborns admitted for mechanical ventilation; no clinical signs of bacterial infection were diagnosed. Group I (n = 44) received iNO (10 ppm, 48 h). Group II (n = 53) did not receive iNO. On the 1, 5, 20 days the plasma concentration of IL-1β, IL-6, IL-8, TNF-α, G-CSF, sFasL, FGF, NO was determined by capture ELISA; CD3^+^CD19^−^, CD3^+^CD19^+^, CD3^+^CD4^+^, CD3^+^CD8^+^, CD3^+^CD69^+^, CD3^+^CD71^+^, CD3^+^CD95^+^, HLA-DR^+^, CD34^+^, CD14^+^, CD3^+^CD56^+^, AnnexinV-FITC^+^PI^−^, AnnexinV-FITC^+^PI^+^—immunophenotype analysis. Statistical programs RStudio Desktop; Kaplan–Meier, Fisher-Irwin method bilateral alternative at 5% significance level.

**Results:** Development of sepsis: I group—4 patients; II—13 (p_1=_0.04; p_2_ = 0.005); fatal outcomes: I—6 patients, II—10 (p_1_ = 0.37; p_2_ = 0.59). Median of the duration of mechanical ventilation: I—5 days, II—10 (*p* = 0.00007); hospitalizations: I—11 days, II—15 (*p* = 0.026). Comparative dynamics of indicators of I group relative to II: a decrease (*p* < 0.05) the concentration of TNF-α, IL-8, IL-6; CD3^+^CD69^+^; CD3^+^CD95^+^, lymphocytes in apoptosis; increase (*p* < 0.05) G-CSF, sFasL, FGF, NO; CD14^+^, CD3^+^CD19^−^.

**Conclusions:** iNO as a part of intensive care decrease in frequency of development of sepsis, the duration of mechanical ventilation, hospitalization; provides a tendency to decrease in frequency of lethal outcomes; reduces cytokine aggression; inhibits apoptosis of lymphocytes, activates the monocyte-macrophage immunity and proliferative processes. It is relevant to continue a research.

## P167

### Left and right ventricular dysfunction in patients with septic shock in relation to seven day and hospital mortality

#### S Kim^1^, YJ Kim^2^, YH Kim^3^, JH Kim^1^

##### ^1^Korea University Ansan Hospital, Critical Care Medicine, Ansan, South Korea, ^2^Korea University Ansan Hospital, Pulmonology, Internal Medicine, Ansan, South Korea, ^3^Korea University Ansan Hospital, Cardiology, Internal Medicine, Ansan, South Korea

*Critical Care* 2023, **27(S1)**: P167

**Introduction:** The prognostic implication of septic cardiomyopathy has not been clearly delivered with the complex hemodynamic change during septic shock. Herein, we evaluated the serial change of left (LV) and right ventricular (RV) function in patients with septic shock and their clinical impact on seven-day and hospital mortality.

**Methods:** Patients were performed transthoracic echocardiography within 48 h from the diagnosis of septic shock and after seven days from the initial evaluation. LV systolic function was evaluated with global longitudinal strain (GLS) and RV function was evaluated with tricuspid annular posterior systolic excursion (TAPSE).

**Results:** A total of 162 patients (men 83, 51.5%; 70.7 ± 13.4 years; APACHE II 30.6 ± 9.2) was enrolled. Their baseline GLS was-14.9 ± 5.2% and TAPSE was 16.9 ± 5.5 mm, and they were improved in follow-up study (GLS-17.1 ± 4.5%, TAPSE 19.9 ± 5.0 mm). Seven-day and hospital mortality was 24 (14.9%) and 64 (39.8%). Seven-day mortality was significantly related with GLS > -16% (odds ratio [OR] 5.938, 95% confidence interval [CI] 1.031–34.190, *p* = 0.046) and APACHE II score (*p* = 0.004). Among seven-day survivors, hospital mortality of seven-day survivors were related with TAPSE < 16 mm at follow-up evaluation (OR 8.855, 95% CI 1.524–51.446, *p* = 0.015) and follow-up SOFA score (OR 1.352, 95% CI 1.083–1.687, *p* = 0.008) but not with follow-up GLS > − 16% (*p* = 0.506) or initial GLS > − 16% (*p* = 0.177) (Fig. 1).

**Conclusions:** LV and RV dysfunction was common in septic shock, but shortly began to improve. Seven-day mortality of septic shock patients was related with GLS, whereas hospital mortality of 7 day survivors were related with TAPSE but not with GLS. However, for the interpretation of the impact of cardiac dysfunction on the prognosis of septicshock, cautious approach is in need considering the stage of septic shock and cut-off values for the parameters.

**Figure Figbi:**
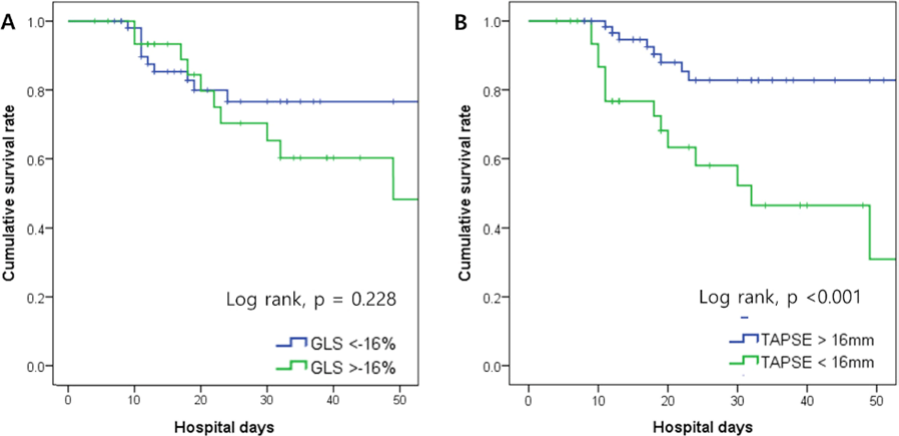
**Fig. 1 (abstract P167)**. Kaplan–Meier curve for the hospital mortality in seven-day survivors, by global longitudinal strain < -16% and ≥ -16% (**A**) and by tricuspid annular posterior systolic excursion > 16 mm and ≤ 16 mm (**B**)

## P168

### Longitudinally monitored hemodynamic responsiveness characteristics in non-invasively monitored shock patients predict hospital mortality

#### J Sahatjian^1^, I Douglas^2^, M Abs^1^, T Hiller^3^, D Tran^3^, A Garcia^3^, J Oakes^3^, C Rosenthal^3^, D Hansell^4^

##### ^1^Baxter Healthcare, Deerfield, USA, ^2^Denver Health Hospital, Critical Care, Denver, USA, ^3^Denver Health Hospital, Denver, USA, ^4^Massachusetts General Hospital, Boston, USA

*Critical Care* 2023, **27(S1)**: P168

**Introduction:** Non-invasive hemodynamic monitoring of cardiac output (CO) and stroke volume (SV), including dynamic measures of fluid responsiveness, has the potential to guide fluid and vasopressor (VP) management in patients with circulatory shock. The goal of this study was to explore the variables that may influence SV and CO improvement and mortality in shock patients when monitored during usual care resuscitation.

**Methods:** The Starling Registry trends CO and SV over time as related to patient outcome (NCT04648293). Patients were managed at a single community academic medical ICU using dynamic assessments of fluid responsiveness through passive leg raise (PLR) to guide the administration of fluid and VP. Patients exhibiting overall improvement in SV and CO (Responders) from initial presentation through hours of monitoring were compared to those who did not improve (Non-Responders).

**Results:** 31 patients received hemodynamic monitoring during their ICU stay. 45% were female; average age was 58 years. 26% of patients had sepsis; 35% had known LV/RV dysfunction. 16 patients (52%) were classified as responders and received a total fluid volume of 500 ml (± 273.86). 15 patients (48%) were non-responders and received 1232 ml of fluid (± 635.37), (Δ732 ml, *p* = 0.040). Both groups were matched with regard to age, gender, and co-morbidities. 100% of patients that had VP started in response to fluid challenge exhibited an improved SV. Responders received longer VP administration (4.3 days ± 4.51 vs 1.7 days ± 1.72, *p* = 0.041). Notably, responders experienced significantly lower mortality (6%) compared with non-responders (33%, *p* = 0.056).

**Conclusions:** Routine incorporation of non-invasive hemodynamic monitoring to inform a personalized approach to shock resuscitation yields novel insights regarding circulatory responsiveness to fluid and VP administration. Trending SV improvement identified non-responder patients at high risk for in hospital death, who receive higher fluid volumes but shorter durations of VP administration.

## P169

### Early versus late administration of intravenous immunoglobulins as adjunctive treatment in patients with septic shock. A retrospective observational study

#### M Benlabed^1^, S Benlabed^2^, R Gaudy^3^, K Benlabed^4^, A Ladjouze^5^, S Aissaoui^6^

##### ^1^Lille University, Anesthesiology, Lille, France, ^2^Free University of Brussels, Intensive Care Unit, Brussels, Belgium, ^3^Lille University, Emergency Department, Lille, France, ^4^Algiers University, Pharmacology, Algiers, Algeria, ^5^Algiers University, Anesthesiology, Algiers, Algeria, ^6^Algiers University, Intensive Care Unit, Algiers, Algeria

*Critical Care* 2023, **27(S1)**: P169

**Introduction:** The efficacy of intravenous immunoglobulins (IVIG) as adjunctive therapy in sepsis is controversial. Some authors emphasized,in randomized controlled trials, the importance of early administration of IVIG in patients with septic shock.So, the objective of our study was to test, in these patients, the impact of timing of IVIG administration( early versus late) on the outcome in terms of morbidity and mortality.

**Methods:** In this retrospective observational study,we collected and analyzed between 2018 and 2021,the data of 2 groups of 20 patients in septic shock according to definition of Sepsis 3 criteria,admitted in university hospital ICU..The patients were 62 + -10 years old, mechanically ventilated, with the same baseline charasteristics and severity scores on admission.All the patients received empirical antibiotics and norepinephrine in the first hour of admission in ICU.The patients were divided into 2 groups according to the timing of IVIG administration: the first group receiving IVIG early on the first hour of admission (Early IVIG group) and the second group receiving IVIG 48 h after admission on ICU(Late IVIG group). IVIG was infused continuously at a dose of 0.5 g/kg with a flow of 3 ml/kg/h during 3 days in the 2 groups. We recorded in the 2 groups: SOFA score day 1 and day 4, blood lactate every day, C-reactive protein (CRP), mean arterial pressure (MAP), P/F ratio day 1 and day 3, time on vaspressors, time on mechanical ventilation, ICU stay, hospital length of stay (LOS) and 28 day mortality.

**Results:** Statistic analysis used Mann Whitney test and results expressed as Mean ± standard deviation. Results are summarized in Fig. 1. We observed a significant reduction of morbidity and 28 day mortality in the Early IVIG group compared to Late IVIG group respectively 20% versus 40%.

**Conclusions:** Our study confirm previous works [1] showing the beneficial effect of early administration of IVIG in sepsis in terms of morbidity and mortality. A large RCT is needed.


**Reference**
Rodriguez A et al. Shock 2005;23: 298–304.

**Figure Figbj:**
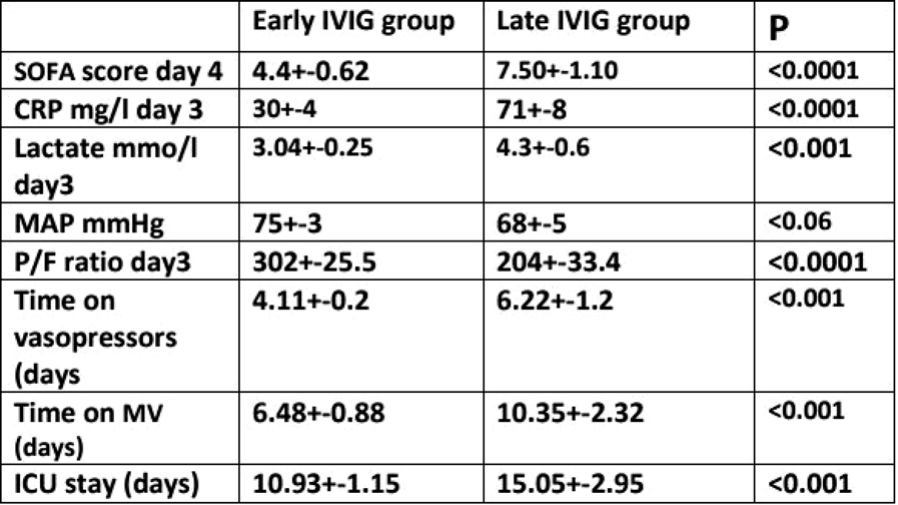
**Fig. 1 (abstract P169)**. Variables in relation with outcome in early IVIG and late IVIG groups

## P170

### Secondary infections in critically ill patients with COVID-19: effects of intravenous IgM-enriched immunoglobulin therapy

#### E Vitali^1^, A Salvucci^1^, A Damia Paciarini^2^, E Casarotta^3^, R Antolini^1^, G Giaccaglia^1^, C Scorcella^2^, R Domizi^2^, E Damiani^1^, A Donati^1^

##### ^1^Università Politecnica delle Marche, Ancona, Italy, ^2^AOU delle Marche, Ancona, Italy, ^3^Università Politecnica delle Marche, Biomedical Sciences, Ancona, Italy

*Critical Care* 2023, **27(S1)**: P170

**Introduction:** Critically ill patients with severe COVID-19 have an increased risk of bacterial and fungal superinfections due to a dysregulated immune response characterized by lymphopenia and low immunoglobulins levels. The intravenous immunoglobulins are involved in pathogen/toxin scavenging and inhibition of inflammatory mediators gene transcription with anti-apoptotic effects on immune system cells. This research aimed to describe the effects of intravenous IgM-enriched immunoglobulins in COVID-19 patients with sepsis due to secondary infections and low IgM levels.

**Methods:** We performed an observational retrospective study, including patients admitted to our intensive care unit (ICU) between March 2020 and February 2021 with severe COVID-19 and sepsis due to a superinfection (known or suspected) treated with intravenous IgM-enriched immunoglobulins. We collected demographic data and comorbidities. We noted hemodinamic data, antimicrobial and adiuvant therapies, laboratory results at ICU admission (T0), at the beginning (T1) and at the end (T2) of the IgM-enriched immunoglobulins infusion and at ICU discharge (T3).

**Results:** In our cohort of 36 patients (Table 1) the prevalence of documented secondary infections was 83%. We observed a significant reduction of leukocytes from T0 to T3 (10.4 [8.3–14.5] × 10^3^/mmc vs 7.1 [4.8–11.2] × 10^3^/mmc, *p* < 0.01) and the SOFA score from T0 to T2 (7 [6–19] vs 5 [3–7], *p* < 0.01) and from T0 to T3 (7 [6–10] vs 4 [2–9], *p* < 0.01); from T1 to T2 (7 [6–9] vs 5 [3–7], *p* < 0.01) and from T1 to T3 (7 [6–9] vs 4 [2–9], *p* < 0.01). Cardiovascular SOFA showed a statistically significant reduction from T1 to T2 (4 [3–4] vs 0 [0–3], *p* < 0.01).

**Conclusions:** The IgM-enriched immunoglobulins could improve organ function, as evidenced by the reduction of the SOFA score. Although the latest Surviving Sepsis Campaign guidelines suggest against using of IgM-enriched immunoglobulins, our study supports its use as an adjunctive therapy in COVID-19 patients with septic shock.

**Table Tabaq:** **Table 1 (abstract P170)**. Summary of demographic and clinical characteristics at ICU admission

Characteristics	Patients, n (%)
Males	31 (86)
Age, years	64 + 11
Hypertension	19 (53)
Obesity	17 (47)
Cardiovascular disease	5 (14)

## P171

### Continuous renal replacement therapy with oXiris filter in critically ill COVID-19 patients

#### R Bounab^1^, N Heming^1^, V Maxime^1^, E Kuperminc^1^, M Carlos^1^, P Moine^1^, S Chevret^2^, D Annane^1^

##### ^1^Raymond Poincaré Hospital (AP HP), Critical Care Unit, GARCHES, France, ^2^Saint-Louis Hospital, Biostatistics and Clinical Epidemiology, Paris, France

*Critical Care* 2023, **27(S1)**: P171

**Introduction:** COVID-19 causes a major inflammatory response, which may progress to shock and multiple organ failure. We explored whether continuous renal replacement therapy (CRRT) using adsorption membrane (oXiris®) could reduce the inflammatory response in critically ill COVID-19 patients with acute renal failure (ARF) [1, 2].

**Methods:** Case–control study including 24 critically ill COVID-2019 patients requiring RRT using an oXiris filter. We measured cytokines before and during treatment as well as relevant clinical endpoints. The control group was selected among COVID-19 patients included into our ongoing RECORDS trial (NCT04280497) who received RRT without adsorbing filters.

**Results:** 24 severe COVID-19 patients, admitted to the intensive care unit (ICU) and treated with CRRT using the oXiris filter between March and April 2020 (20 males and 4 females); median age 67. The average time from COVID-19 symptoms to initiation of oXiris treatment was 18 ± 7 days, and from ICU admission to initiation of oXiris treatment 9.5 ± 7.8 days and from ARF to oXiris treatment was 3 ± 5 days. The average length of treatment was 152.8 ± 92.3 h. Treatment was associated with cytokine decreases for IL-1beta (*p* = 0.00022), MCP-1 (*p* = 0.03), and MIP-1 alpha (*p* = 0.03). The SOFA scores also showed a reduction over 48 h of therapy without reaching statistical significance. Our study found no significant effect of hemodynamic status. The average ICU stay length was 14 ± 5 days and the mortality rate was 79% in the Oxiris group. We compared the mortality across the two matched groups, there was no evidence of any difference in mortality (Fig. 1).

**Conclusions:** In our study, CRRT using the oXiris filter seemed to effectively remove IL-1 beta, MCP-1, and MIP-1 alpha in COVID-19 patients. These exploratory results should be confirmed in a randomized controlled study.


**References**
Broman ME et al. PLoS One 2019;14:e0220444.Stockmann H et al. Crit Care Med 2022;50: 964–976.

**Figure Figbk:**
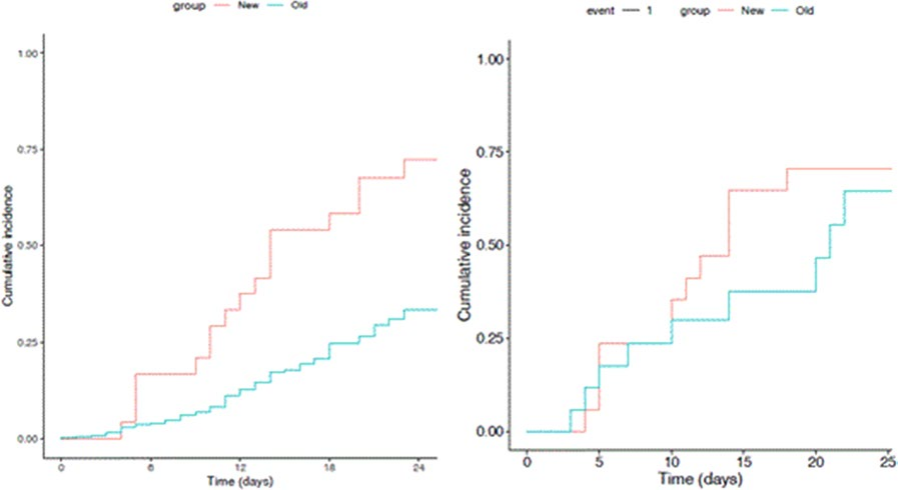
**Fig. 1 (abstract P171)**. Cumulative incidence of death before (left) and after (right) matching

## P172

### A novel haemofiltration system to selectively remove IL-6 from the bloodstream: preclinical efficacy and safety in healthy volunteers

#### N Waalders^1^, D Van Lier^1^, L Moran^2^, M Cheppallan^2^, D Gold^2^, W Twiggers^2^, C Blanco-Andujar^2^, G Frodsham^2^, M Kox^1^, P Pickkers^1^

##### ^1^Radboudumc, Department of Intensive Care Medicine, Nijmegen, Netherlands, ^2^MediSieve Limited, MediSieve Limited, London, UK

*Critical Care* 2023, **27(S1)**: P172

**Introduction:** Extracorporeal blood purification therapies have not yet demonstrated to selectively remove cytokines from the blood or to improve outcome of septic patients. A novel method is represented by the MediSieve Magnetic Haemofiltration System (MMHS), which aims to selectively remove interleukin (IL)-6 from the bloodstream by infusing magnetic beads coupled to anti-IL-6 antibodies into an extracorporeal circulation system containing a filter placed inside a magnet (Fig. 1A). We assessed the efficacy of MMHS to remove IL-6 in a benchtop experiment and its safety in humans.

**Methods:** Four human plasma samples were spiked with a mixture of several cytokines and passed through the MMHS at a flow rate of 120 ml/min. Magnetic beads coupled with anti-IL-6 antibodies were infused into the system and cytokine concentrations were determined at several timepoints. Safety was evaluated by performing haemoperfusion using the MMHS in six healthy male and female volunteers (both n = 3) for five hours with a flow rate of 120 mL/min. Vital signs were monitored continuously and blood samples were serially obtained.

**Results:** The MMHS rapidly and selectively removed IL-6 from human plasma, without affecting concentrations of other cytokines (Fig. 1B). No serious adverse events occurred during haemoperfusion in healthy volunteers, who did not experience any symptoms either. Several mild adverse events were observed, including a small haematoma around the canulation site and transient, clinically irrelevant increases in iron, chromium and manganese. Haemofiltration using the MMHS did not cause relevant changes in vital signs (Fig. 1C, D), thrombocytes, leukocytes (Fig. 1E, F), or increases in plasma cytokine concentrations.

**Conclusions:** The MMHS selectively removes IL-6 from human plasma and is well-tolerated and safe in healthy volunteers. In future studies, we will use the experimental endotoxemia model to assess the efficacy of the MMHS in removing IL-6 from the circulation in humans in vivo.

**Figure Figbl:**
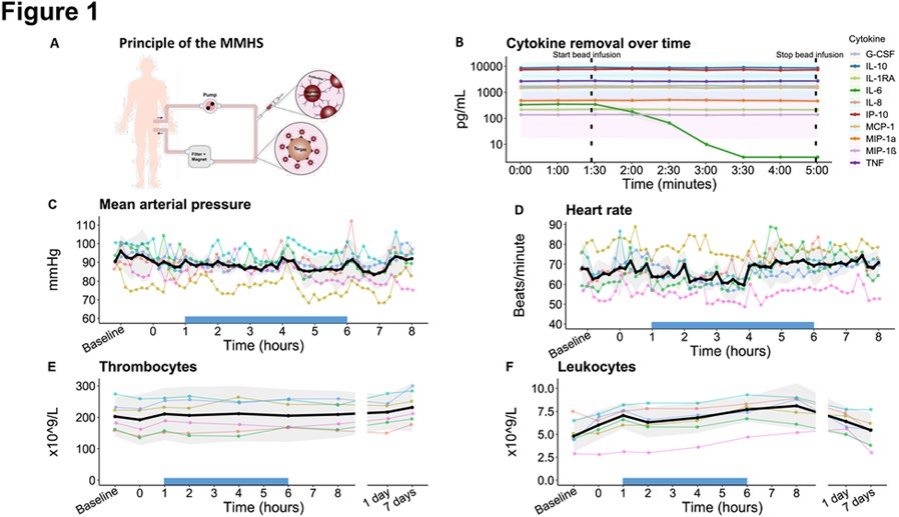
**Fig. 1 (abstract P172)**. **A** Principle of the MediSieve Magnetic Haemofiltration System (MMHS). Blood passes through an extracorporeal circuit containing the MediSieve Filter placed inside the MediSieve Magnet. Magnetic beads coupled to an antibody directed against a substance or cell type of choice are infused into this extracorporeal circuit before the Filter. Of note, bead infusion was not used in the current human safety study. **B** Cytokine removal by MMHS in the benchtop experiment. Data are presented as median

## P173

### Cytosorb hemoperfusion markedly attenuates circulating cytokine concentrations during systemic inflammation in humans in vivo

#### A Jansen^1^, N Waalders^1^, DPT Van Lier^1^, EN Deliargyris^2^, J Scheier^3^, M Kox^1^, RP Pickkers^1^

##### ^1^Radboud University Medical Center, Department of Intensive Care Medicine, Nijmegen, Netherlands, ^2^CytoSorbents Corporation, Princeton, USA, ^3^CytoSorbents Europe, Berlin, Germany

*Critical Care* 2023, **27(S1)**: P173

**Introduction:** Although the CytoSorb hemoadsorption device has been demonstrated to be capable of clearing inflammatory cytokines, it has not yet been shown to attenuate plasma cytokine concentrations in humans in vivo. We investigated the effects of CytoSorb hemoperfusion on plasma levels of various cytokines using the repeated human experimental endotoxemia model, a highly standardized and reproducible human in vivo model of systemic inflammation and immunological tolerance induced by administration of bacterial lipopolysaccharide (LPS).

**Methods:** Twenty-four healthy male volunteers (age 18–35) were intravenously challenged with LPS (a bolus of 1 ng/kg followed by continuous infusion of 0.5 ng/kg/hr for three hours) twice: on day 0 to quantify the initial cytokine response and on day 7 to quantify the degree of endotoxin tolerance. Subjects were randomized to receive either CytoSorb hemoperfusion for 6 h during the first LPS challenge (CytoSorb group), or no intervention (control group). Blood samples were serially obtained to determine cytokine plasma concentrations and clearance rates.

**Results:** LPS administration led to a profound increase in cytokine plasma concentrations during both LPS challenge days (Fig. 1). Compared to the control group, significantly lower plasma levels of tumor necrosis factor (TNF, −58%, *p* < 0.0001), interleukin (IL)-6 (−71%, *p* = 0.003), IL-8 (−48%, *p* = 0.02) and IL-10 (−26%, *p* = 0.03) were observed in the CytoSorb group during the first LPS challenge. Peak clearance rates ranged from a median of 75.1 [70.7–87.1] ml/min for TNF, to 32.5 [28.0–44.2] ml/min for IL-6. The degree of endotoxin tolerance apparent during the second LPS challenge was not affected by previous CytoSorb use.

**Conclusions:** CytoSorb hemoperfusion effectively attenuates circulating cytokine concentrations during systemic inflammation in humans in vivo, whereas it does not affect tolerance induction. Therefore, CytoSorb therapy may be of benefit in conditions characterized by excessive cytokine release.

**Figure Figbm:**
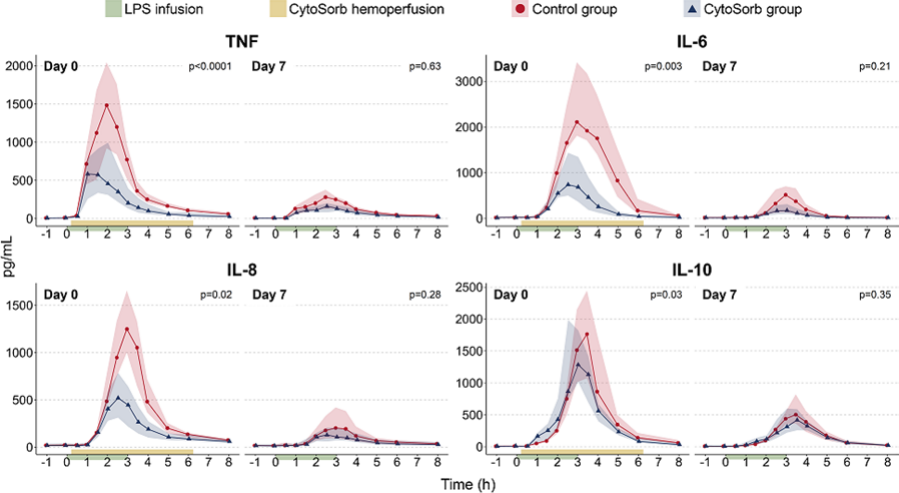
**Fig. 1 (abstract P173)**. Plasma cytokine concentrations during the first (day 0) and second (day 7) LPS challenge. Data displayed as median and interquartile range. P values were computed by repeated measures two-way analysis of variance (time*group interaction term) on log-transformed data

## P174

### Cytokine hemoadsorption with CytoSorb® in post-cardiac arrest syndrome, a pilot randomized controlled trial

#### C Monard, N Bianchi, E Poli, M Altarelli, A Debonneville, M Oddo, L Liaudet, A Schneider

##### Centre Hospitalier Universitaire Vaudois, Service de Médecine Intensive Adulte, Lausanne, Switzerland

*Critical Care* 2023, **27(S1)**: P174

**Introduction:** Hemoadsorption (HA) might mitigate the systemic inflammatory response associated with post-cardiac arrest syndrome (PCAS) and improve outcomes. We investigated the feasibility, safety and efficacy of HA with CytoSorb® in cardiac arrest (CA) survivors at risk of PCAS.

**Methods:** In this pilot randomized controlled trial, we included patients admitted in ICU following CA, who required norepinephrine (> 0.2 µg/kg/min), and/or had serum lactate > 6 mmol/l and/or a time-to-return of spontaneous circulation (ROSC) > 25 min. Those requiring ECMO or renal replacement therapy were excluded. Eligible patients were randomly allocated to receive standard-of-care (SOC) or SOC plus HA. HA was performed as standalone therapy for 24 h, using CytoSorb® and regional heparin-protamine anticoagulation. We collected feasibility, safety, clinical data and serial plasma cytokines levels over 72 h of randomization.

**Results:** We enrolled 21 patients, 16 (76%) had out-of-hospital CA and median (IQR) time-to-ROSC was 30 (20, 45) minutes. Eleven received SOC and 10 HA, which was initiated in all 10 patients within 18 (11, 23) hours after ICU admission, and for a duration of 21 (14, 24) hours. The intervention was well tolerated except for a trend for higher rate of aPTT elevation (5 (50%) vs 2 (18%) *p* = 0.18) and mild (< 150 g/l) thrombocytopenia (5 (50%) vs 2 (18%) *p* = 0.18). Interleukin (IL)-6 plasma levels at randomization were low (< 100 pg/ml) in 10 (48%) patients and elevated (> 1000 pg/ml) in 6 (29%). Median relative reduction of IL-6 at 48 h was 75% (60, 94) in the HA group versus 5% (-47, 70) in the SOC group (*p* = 0.06). A similar decrease was observed in all tested cytokines (Table 1).

**Conclusions:** In CA survivors at risk of PCAS, HA was feasible, safe and was associated with a higher reduction in cytokine plasma levels. Future trials are needed to further define the role of HA after CA. Those studies should include cytokine assessment to enrich study population.

**Acknowledgement:** These data have been published in full in Crit Care. 2023;27:36.

**Table Tabat:** **Table 1 (abstract P174)**. Relative reduction (%) in cytokine blood levels within 48 h post randomization

	Control, n = 10	Hemoadsorption, n = 8	Total, n = 18	*p* value
Interleukin-6	5 (− 47, 7)	75 (60, 94)	60 (− 13, 86)	0.06
Interleukin-8	72 (13, 90)	90 (68, 98)	83 (33, 93)	0.06
Interleukin-10	62 (41, 76)	91 (35, 99)	75 (41, 92)	0.20
Tumor necrosis factor-α	60 (5, 100)	100 (96, 100)	98 (25, 100)	0.29
Interleukin-5	20 (2, 100)	100 (89, 100)	94 (4, 100)	0.10
Interleukin-2	77 (35, 90)	82 (63, 100)	77 (63, 100)	0.67
Interleukin-4	38 (− 14, 93)	84 (72, 97)	75 (16, 97)	0.47

Data are presented as median (interquartile range), and compared between groups with Fisher exact test. Relative reduction = (value at randomization-value at 48 h)/value at randomization. One patient received hemoadsoprtion for < 12 h and was excluded from efficacy analysis. One patient in each group died within 48 h, reduction at 48 h could be evaluated in 10 controls and 8 hemoadsorption patients

## P175

### Italian retrospective multicentric observational study on the use of CytoSorb® in critically ill paediatric patients (CYTOPED Study)

#### S Bianzina^1^, A Tessari^2^, M Agulli^3^, G Bregant^4^, P Lonardi^5^, S Scollo^6^, A Moscatelli^1^, C Serpe^7^, G Longo^8^, G Bottari^7^

##### ^1^Neonatal and Pediatric Intensive Care Unit, Emergency Department, IRCCS G. Gaslini Hospital, Genoa, Italy, ^2^Pediatric Intensive Care Unit, Department of Women and Children’s Health, University Hospital of Padua, Padua, Italy, ^3^Anaesthesiology and Intensive Care, Cardiothoracic and Vascular Department, IRCCS University Hospital of Bologna, Bologna, Italy, ^4^Pediatric Intensive Care Unit, Children Hospital Burlo Garofalo, Trieste, Italy, ^5^Pediatric Nephrology Unit, AOU Città della Salute e della Scienza di Torino, Torino, Italy, ^6^Mediterranean Pediatric Cardiac Center, Taormina – San Vincenzo Hospital, Taormina, Italy, ^7^Pediatric Emergency Department, Pediatric Intensive Care Unit, Children Hospital Bambino Gesù, IRCCS, Rome, Italy, ^8^Pediatric Nephrology, Dialysis and Transplant Unit, Department of Pediatrics, University Hospital of Padua, Padua, Italy

*Critical Care* 2023, **27(S1)**: P175

**Introduction:** CytoSorb® is a cartridge for the adsorption of inflammatory mediators, bilirubin, myoglobin and other xenobiotics, directly from whole blood. The clinical experience is widely documented in adults, whereas experience in paediatric settings is currently limited to single case reports or monocentric retrospective studies. In order to be able to collect evidences in larger paediatric population, an Italian multicentre network was founded (CYTOPED Study Group).

**Methods:** Italian multicentric observational study on the use of CytoSorb® in critically ill paediatric patients. The prospective enrollment by Italian children hospitals is ongoing since February 2021. A retrospective analysis was conducted by Italian centres from January 2018 to February 2021.

**Results:** 50 patients were enrolled. PELOD-2 score at PICU admission was 7.38 (SD 4.29, risk of mortality 10.60%) and before hemoperfusion 8.72 (SD 4.44, risk of mortality 16.46%). Clinical indications to hemoperfusion were sepsis or septic shock (29 patients), liver failure (4 patients), rhabdomyolysis (4 patients), hearth surgery (3 patients) and other indications (11 patients). CytoSorb® has been applied in CKRT (56% of cases), ECMO (32%), hemoperfusion (6%) and cardiac by-pass (6%) (Fig. 1). Median time of hemoperfusion was 3 days and median number of sorbents used was 3. Anticoagulation in extracorporeal circuits has been managed with heparin (71% of cases) and regional anticoagulation with citrate (29%). 8 adverse events were recorded (hemodynamic instability at hemoperfusion starting, haemodilution, circuit clotting and thrombocytopenia).

**Conclusions:** Our data demonstrates that CytoSorb® therapy is safe and feasible in children. The advancement of our study and the prospective arm of CYTOPED Study will allow to investigate further aspects of this therapy, such as dose, timing and use of antibiotics in conjunction with extracorporeal therapies.

**Figure Figbn:**
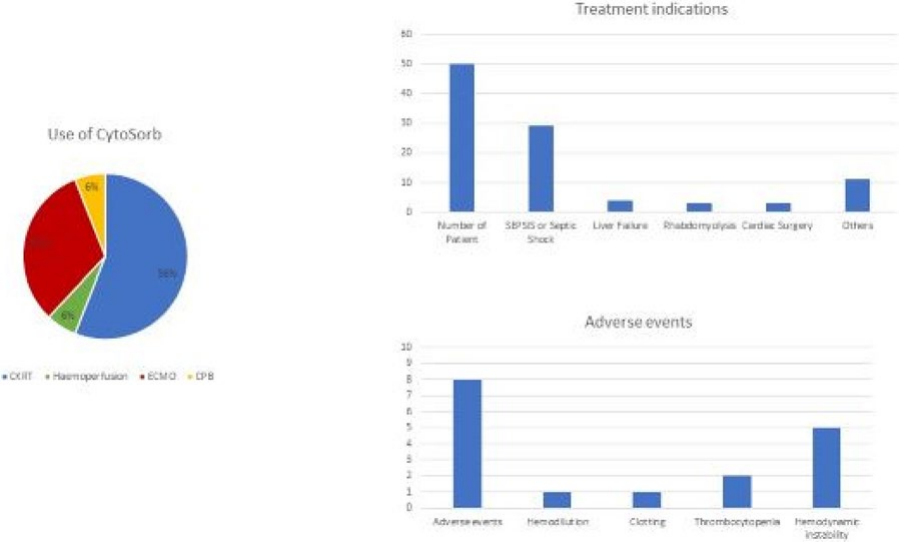
**Fig. 1 (abstract P175)**. Use of CytoSorb, treatment indications and adverse events

## P176

### CytoSorb for apixaban clearance

#### A Harris, NJ Lees

##### Harefield Hospital, Intensive Care Unit, Uxbridge, UK

*Critical Care* 2023, **27(S1)**: P176

**Introduction:** Planned cessation of oral anticoagulation (DOAC) prior to elective surgery is straightforward, however challenges arise when urgent treatment is required [1]. Patients taking DOACs have an increased risk of mortality due to bleeding so it is essential to have effective tools to reduce circulating levels prior to emergency surgery [1]. Use of CytoSorb haemoadsorber (Cytosorbents) [2] is EU approved to remove rivaroxaban during on pump cardiac surgery [3], but only a few case reports exist of its effects on apixaban [4,5].

**Methods:** A patient presented with decompensated heart failure and acute kidney injury (AKI), with a background of rheumatic heart disease, multiple cardiac surgeries and apixaban use for atrial fibrillation. The patient was admitted to cardiac intensive care unit (ICU) and required renal replacement therapy (RRT). At admission, serum apixaban levels were 204 ng/ml, 40% higher than upper limit of normal and the patient was commenced on RRT. After 4 h, apixaban levels continued to rise so the CytoSorb haemoadsorber was added to the filtration system (Prismaflex).

**Results:** After 3.5 h, apixaban levels were reduced by 77% and the patient experienced no adverse bleeding; this was essential for their heart transplant assessment (Fig. 1). After removal of the CytoSorb at 14 h, the apixaban remained at safe levels and RRT was stopped 4 days later.

**Conclusions:** Approximately 27% of apixaban clearance is via the kidneys [6], so AKI can lead to bleeding due to dangerously high levels. This case report shows CytoSorb can be used to effectively reduce apixaban levels in a time efficient manner and is a potential therapy for patients undergoing emergency procedures and surgery.

**Acknowledgement:** Informed consent was obtained from the patient to publish the clinical details.


**References**
Bolliger D et al. J Cardiothorac Vasc Anesth 2022;36:1645–7Cytosorbents Europe GMBH. CytoSorb at https://cytosorb-therapy.com/en/Hassan K et al. Ann Thorac Surg 2019;108:45–51Hassan K et al. J Clin Med 2022;11:5889Tripathi R et al. Eur Hear Journal Acute Cardiovasc Care 2021;10: zuab020.229Byon W et al. Clin Pharmacokinet 2019;58:1265–79

**Figure Figbo:**
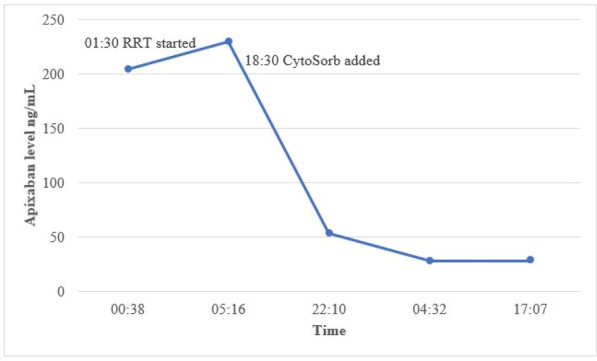
**Fig. 1 (abstract P176)**. Graph showing apixaban levels measured at various intervals during ICU admission

## P177

### Hydrocortisone treatment in septic mice increases cholesterol availability without improving adrenal function and with exacerbated wasting of lean tissue

#### L De Bruyn, A Téblick, T Van Oudenhove, S Vander Perre, I Derese, L Pauwels, S Derde, G Van den Berghe, L Langouche

##### KU Leuven, Department of Cellular and Molecular Medicine, Leuven, Belgium

*Critical Care* 2023, **27(S1)**: P177

**Introduction:** Sepsis-induced hypocholesterolemia is associated with poor outcome but the underlying mechanism is unclear. As it has been postulated that low plasma cholesterol could partly be due to an increased conversion to cortisol, we hypothesized that hydrocortisone (HC) treatment, via reduced de novo adrenal corticosterone (CORT) synthesis, might improve cholesterol availability and as such affect the adrenal gland and skeletal muscle.

**Methods:** In a catheterized, fluid-resuscitated, antibiotics-treated mouse model of prolonged sepsis (5 d), septic mice received either HC (1.2 mg/d) or placebo, whereas healthy mice served as controls (N = 50). Plasma CORT, cholesterol, and adrenocortical cholesterol ester content were quantified. Steroidogenic capacity of ACTH was evaluated in an adrenal explant study. Total body mass loss was measured to evaluate CORT-induced wasting in addition to ex vivo muscle force, myofiber size and mRNA expression of atrogenes *(Trim63*, *Fbox32, Foxo3*) and regeneration markers (*Myostatin*, *Myf5*, *Myogenin*).

**Results:** Plasma CORT was normalized in HC septic mice, whereas the sepsis-induced reduction in plasma HDL- and LDL-cholesterol, and adrenocortical cholesterol ester content was attenuated as compared with placebo septic mice (*p* < 0.05), but without improving the blunted ACTH-induced CORT secretion (*p* = 0.5). Total body mass was median 13% further decreased in HC septic mice (*p* < 0.01) as compared with placebo septic mice, with no additional effect on sepsis-induced loss in muscle mass, force and myofiber size (p > 0.05). The sepsis-induced rise in muscle atrogenes was not further affected by HC treatment (p > 0.05), whereas *Myostatin* and *Myf5*/*Myogenin* expression was respectively increased (*p* = 0.0003) and decreased (*p* < 0.05), as compared with placebo septic mice.

**Conclusions:** Glucocorticoid treatment partly restored the sepsis-induced hypocholesterolemia without improving adrenal function, and at a cost of exacerbated loss of total body mass and suppressed muscle regeneration mechanisms.

## P178

### Effects of enrichment strategies on outcome of adrecizumab treatment in septic shock: post-hoc analyses of the phase II AdrenOSS-2 trial

#### DPT van Lier^1^, A Picod^2^, G Marx^3^, PF Laterre^4^, O Hartmann^5^, F Azibani^6^, J Struck^7^, K Santos^8^, A Mebazaa^2^, P Pickkers^1^

##### ^1^Radboudumc, Intensive Care, Nijmegen, Netherlands, ^2^hôpital Lariboisière, Anesthésie-réanimation, Paris, France, ^3^Universitätsklinikum Aachen, Klinik fur Operative Intensivmedizin und Intermediate Care, Aachen, Germany, ^4^Cliniques Universitaires Saint-Luc, Unité de Soins Intensifs, Bruxelles, Belgium, ^5^Sphingotec GmbH, Hennigsdorf, Germany, ^6^Hôpital Lariboisière, 2. Anesthésie-réanimation, Paris, France, ^7^Adrenomed AG, Hennigsdorf, Germany, ^8^4TEEN4 Pharmaceuticals GmbH, Hennigsdorf, Germany

*Critical Care* 2023, **27(S1)**: P178

**Introduction:** Adrecizumab, a non-neutralizing antibody of adrenomedullin (ADM) was recently investigated regarding its potential to restore endothelial barrier function in septic shock patients. Circulating dipeptidyl peptidase 3 (cDPP3), a protease involved in the degradation of several cardiovascular mediators, represents another biological pathway associated with septic shock outcome, although unrelated to ADM. Therefore, the prognosis of patients with elevated cDPP3 may not be influenced by adrecizumab. The aim of our study was to evaluate the effects of cDPP3-based population enrichment, as well as time until initiation of treatment, on the treatment efficacy of adrecizumab.

**Methods:** Post-hoc analysis of AdrenOSS-2, a phase-II, double-blind, randomized, placebo-controlled biomarker-guided trial of adrecizumab.

**Results:** Compared to the total study cohort (HR for 28-day mortality of 0.84 (95% CI 0.53; 1.31), *p* = 0.439), therapeutic benefit of adrecizumab tended to be more pronounced in the subgroup of 249 patients with low cDPP3 (< 50 ng/ml); HR of 0.61 (95% CI 0.34; 1.08), *p* = 0.085) (Fig. 1A/B). Median duration to study drug infusion was 8.5 h. In the subgroup of 129 patients with cDPP3 < 50 ng/ml and an early start of treatment (< 8.5 h after septic shock diagnosis) HR for 28-day mortality versus placebo was 0.49 (95% CI 0.21–1.18), *p* = 0.094 (Fig. 1C). In multivariate interaction analyses corrected for baseline disease severity, both cDPP3, as well as the cDPP3 * treatment interaction term were associated with a reduced HR for 28-day mortality in the Adrecizumab treated group; *p* = 0.015 for cDPP3 in univariate analysis, *p* = 0.025 for the interaction term between cDPP3 and treatment group. In contrast, treatment timing was not significantly associated with 28-day mortality in multivariate interaction analyses.

**Conclusions:** In septic shock patients, a further post-hoc enrichment strategy based on cDPP3 may indicate that therapeutic efficacy is most pronounced in patients with lower cDPP3 levels.

**Figure Figbp:**
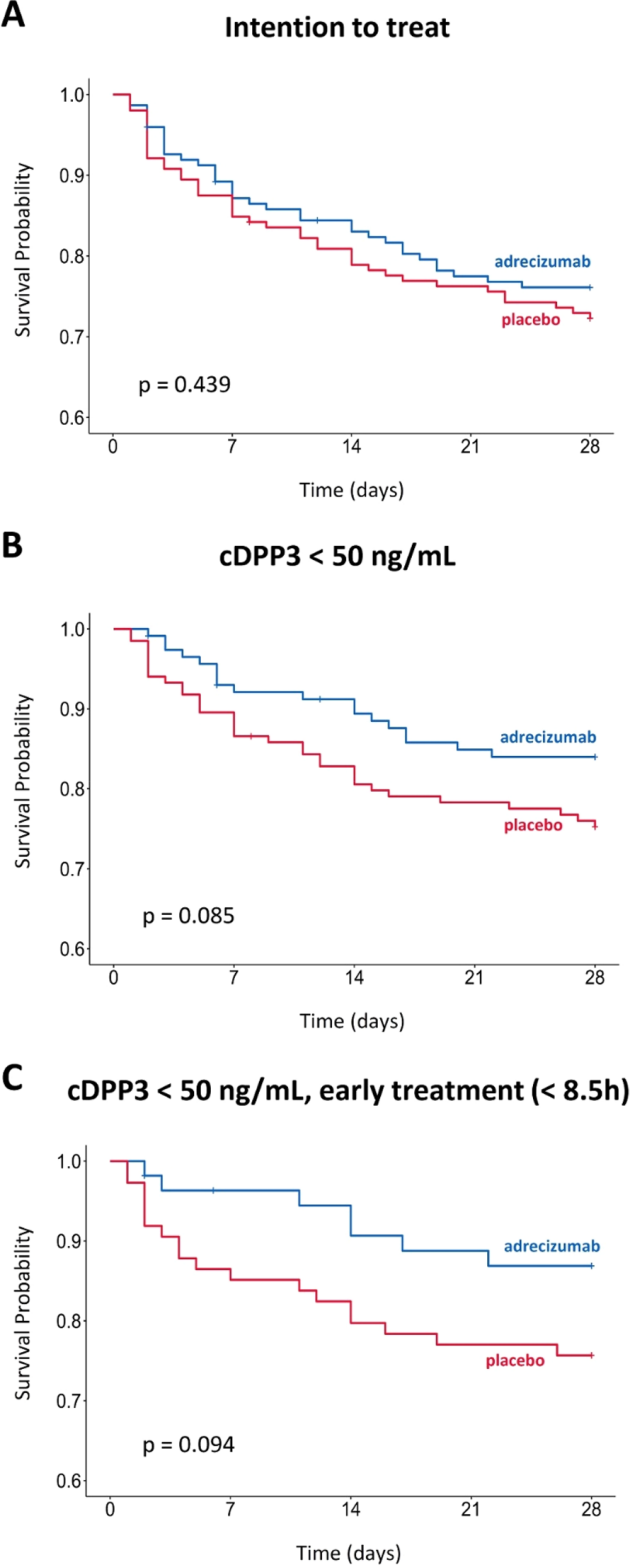
**Fig. 1 (abstract P178)**. Kaplan–Meier of 28-day mortality after adrecizumab/placebo infusion, based on cDPP3 and time until initiation of treatment stratification. Data is displayed for the original ITT analysis (panel **A**), the cDPP3 < 50 ng/mL subgroup (panel **B**) and the combined stratification (cDPP3 < 50 ng/mL and time until initiation of treatment < 8.5 h) subgroup (panel **C**). Both adrecizumab dosing groups were combined. Crosses represent censored patients

## P179

### Ilofotase alfa exerts renal protective effects in patients with sepsis-associated acute kidney injury

#### K Liu^1^, K Doi^2^, R Bellomo^3^, M Kraan^4^, P Pickkers^5^

##### ^1^University of California, Division of Nephrology and Critical Care Medicine, San Francisco, USA, ^2^University of Tokyo Hospital, Emergency and Critical Care Medicine, Tokyo, Japan, ^3^Austin Health, Intensive Care Research, Melbourne, Australia, ^4^AM-Pharma B.V., Utrecht, Netherlands, ^5^Radboudumc, Intensive Care, Nijmegen, Netherlands

*Critical Care* 2023, **27(S1)**: P179

**Introduction:** Sepsis-associated AKI (SA-AKI) is associated with mortality and long-term sequelae, but no pharmacological options are available. Ilofotase alfa is a human recombinant alkaline phosphatase with renoprotective effects that showed improved survival in SA-AKI patients in a Phase 2 study [1]. ‘REVIVAL’, a large global phase 3 trial (NCT04411472), was conducted to confirm the efficacy and safety of Ilofotase alfa in patients with SA-AKI.

**Methods:** REVIVAL was conducted in SA-AKI patients (on vasopressor and < 48 h of AKI, according to KDIGO definition) recruited from 120 sites in Europe, North-America, Australia, New Zealand, and Japan. The primary endpoint was 28-day all-cause mortality. The key secondary endpoint was Major Adverse Kidney Events on day 90 (MAKE90). A pre-unblinding modified MAKE90 was defined as death up to Day 90, RRT through Day 28 and on Day 90, > 25% drop in eGFR at Day 90, rehospitalization to Day 90.

**Results:** The trial stopped for futility after 649 inclusions (placebo n = 319; IA n = 330) following a planned interim analysis with an observed 28-day mortality of 27.9% on both arms. No safety concerns were identified. A beneficial effect on the modified MAKE90 was observed (*p* = 0.031) (Fig. 1), mainly driven by reduction of the number of patients receiving renal replacement therapy (RRT) (28.2% Ilofotase alfa vs 36.4% placebo). This effect was most pronounced in patients with pre-existent renal impairment.

**Conclusions:** Ilofotase alfa exerts renoprotective effects in SA-AKI. Further research is required to confirm a role for treatment of SA-AKI in patients with pre-existent renal impairment.


**Reference**
Pickkers et al. JAMA 2018;320:1998–2009.

**Figure Figbq:**
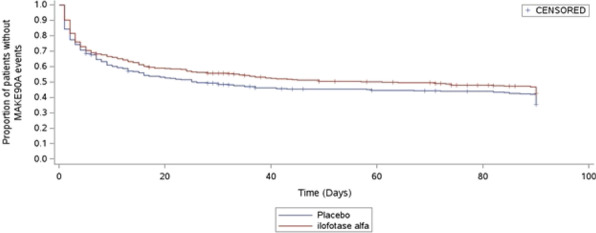
**Fig. 1 (abstract P179)**. Proportion of patients without a MAKE90 at day 90. *p* value for the probability difference of ilofotase alfa compared to placebo 0.031. All patient events prior to Day 90 are censored/counted on the date of the event, all patient events following Day 90 are marked on Day 90

## P180

### Correlation of preoperative albumin value and EVLW shift followed by LUS in patients after esophagectomy

#### A Šimunić Forić^1^, M Ilić^2^, L Štefančić^1^, A Peršin Beraković^1^, H Feljan^3^, M Karaman Ilić^4^

##### ^1^Radiochirurgia Zagreb, Anesthesiology and ICU, Sveta Nedelja, Croatia, ^2^UVA Department of Psychological Methods and Statics, Amsterdam, Netherlands, ^3^Radiochirurgia Zagreb, Thoracic Surgery, Sveta Nedelja, Croatia, ^4^Radiochirurgia Zagreb, University of Osijek, School of Dental Medicine, Anesthesiology and ICU, Zagreb, Croatia

*Critical Care* 2023, **27(S1)**: P180

**Introduction:** Pulmonary complications, occurs in 16–67% of post esophagectomy patients. The most frequent is pulmonary edema. Malnutrition is the cause of preoperative low albumin value. The aim of our study was to investigate whether there is a correlation between preoperative albumin values and new-onset pulmonary edema.

**Methods:** We retrospectively analyzed one year data that included: age, gender, BMI, preoperative albumin, value, intraoperative volume, B-line score (BLS) and lactate value 24 h and 48 h after admission in ICU. For BLS Blue protocol was followed.

**Results:** The average age of the patients was 61.12 years ± 8.41(for a men 59.31 years ± 7.81 and for the women 69 years ± 8.1). There was no difference in the age of the subjects (*p* = 0.18). The patients received intraoperativly an average of 8.44 ml/kg/h of volume of which a restrictive approach was used in 5 patients. The highest infused volume was 14.08 ml/kg/h. BLS was independent of intraoperativly infused volume in both measurements (*p* = 0.823, vrs *p* = 0.113).Preoperative albumin values were in range between 25 and 47 g/l with an average of 35.9 ± 6.62 g/l. BLSs were between ≤ 5 and ≥ 15. Four patients had BLS > 15 24 h versus two 48 h after admission (Fig. 1). Our data showed that BLS and preoperative albumins are independent values, in both measurements (*p* = 0.972 vs *p* = 0.6). Postoperative lactate values were independent of preoperative albumin level in both measurements (*p* = 0.546 vs *p* = 0.334).

**Conclusions:** We have not found evidence to support our hypothesis that preoperative albumin values are in correlation with new-onset pulmonary edema. Despite that, we note an increase in EVLW. Factors that could have contributed to a null result are small sample size and type of sugical procedure that could itself result in an increase of BLS. Further studies need to be done on this matter, as corroborating evidence for this topic.

**Figure Figbr:**
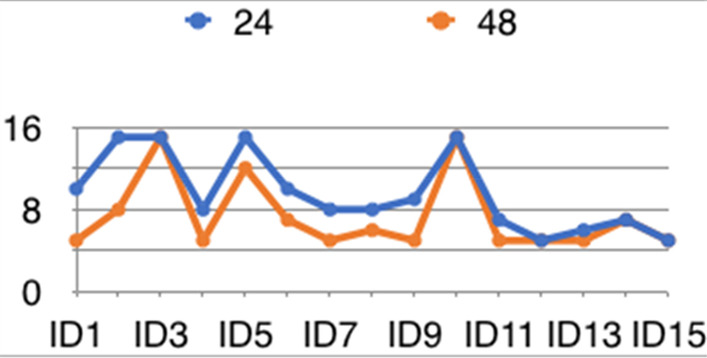
**Fig. 1 (abstract P180)**. BLS 24 and 48 h after admission in ICU. ID = patient identification

## P181

### Colloid administration for cytoreductive surgery plus hyperthermic intraperitoneal chemotherapy (CRS-HIPEC), comparative clinical outcomes from a historical cohort

#### G Madrid, JD Guerra, JP Caceres

##### University Hospital Fundacion Santa Fe de Bogota, Bogotá, Colombia

*Critical Care* 2023, **27(S1)**: P181

**Introduction:** Colloids have been associated with increased unfavorable outcomes in patients who underwent major surgery [1]. Their safety profile is under scrutiny due to increased risk of renal failure and allergic reactions, as albumin remains as the mainstay of colloid therapy. We aimed to explore associations between types of colloids administered in CRS-HIPEC and postoperative outcomes.

**Methods:** A cohort of patients taken to CRS-HIPEC from 2007 to 2020 at a university hospital were analyzed retrospectively. We compared outcomes for two groups: albumin only, and hyroxyethyl starch (HES, Voluven 6% ®) or oxypolygelatine (OPG. Gelifundol 5.5%). HES/OPG were used at our institution from 2007 to 2013. Outcomes were ICU length of stay (LOS), total LOS, ICU readmission, inpatient mortality, time for extubation, need for tracheostomy, time to ambulation and oral feeds, need for transfusion, estimated blood loss, intraoperative crystalloids, and units of red blood cells and plasma transfused in surgery.

**Results:** 148 patients were included. 98 (60%) patients received albumin, and 50 (30%) HES/OPG. ICU (median 3 [IQR 1], vs 6 [IQR 6], *p* < 0.01), hospital LOS (16 [IQR 13] vs. 22.5 [IQR 22], *p* < 0.01), and days for extubation (mean 0.7 [SD 0.78] vs 7.5 [SD 22], *p* < 0.01) were significantly shorter in patients who received albumin. 4 patients in the HES/OPG group received a tracheostomy compared to 0 in the other group (*p* = 0.01) (Table 1). Patients who received albumin had less crystalloid and blood product requirements, bled less, and were able to feed and ambulate earlier (*p* < 0.05).

**Conclusions:** Patients who received intraoperative albumin during CRS-HIPEC and were admitted to ICU had shorter LOS, days to extubation, and less blood product and fluid requirements when compared to patients who received other types of colloids.


**Reference**
Elgendy et al. Indian J Anaesth 2019;63:805–13.

**Table Tabas:** **Table 1 (abstract P181)**. Correlations with Mann–Whitney test and ***p*** < 0.05

	Albumin		OPG or HES	
	Mean (SD)	Median (IQR)	Mean (SD)	Median (IQR)
ICU stay—days	3.2 (2.14)	3 (1)	11.2 (20.3)	6 (5)
Days on vent	0.7 (0.78)	1 (1)	7.5 (22)	1 (1)
PRBC units transfused	1.5 (1.7)	1 (2)	5.7 (4.8)	4 (6)
Plasma units transfused	0.5 (1.6)	0 (0)	3.8 (5.3)	1.5 (6)
Crystalloids—ml	9080 (4453)	8000 (3800)	14,182 (12,000)	12,000 (9500)
Estimated blood loss—ml	1125 (1025)	800 (100)	2885 (1837)	2000 (2400)

## P182

### Plasma compared to crystalloid resuscitation does not reduce endothelial and organ injury in a pneumosepsis rat model

#### DP Van den brink^1^, DJB Kleinveld^1^, J Vos ^1^, JJTH Roelofs^2^, NC Weber^1^, JD Van Buul^3^, NP Juffermans^1^

##### ^1^Amsterdam UMC, AMC, Laboratory of Experimental Intensive Care and Anesthesiology, Amsterdam, Netherlands, ^2^Amsterdam UMC, AMC, Department of Pathology, Amsterdam, Netherlands, ^3^Sanquin Research and Landsteiner Laboratory, Molecular Cell Biology Laboratory at Department of Molecular Hematology, Amsterdam, Netherlands

*Critical Care* 2023, **27(S1)**: P182

**Introduction:** Endothelial permeability is a hallmark of septic shock leading to tissue edema and organ failure. Primary treatment of sepsis patients consists of resuscitation with crystalloids. However, crystalloids aggravate endothelial permeability, which may be related to low protein content. We hypothesized that resuscitation with plasma compared to crystalloids limits endothelial and organ injury in a pneumosepsis rat model.

**Methods:** Male Sprague–Dawley rats (n = 11) were intratracheally inoculated with 5 × 10^8^
*Streptococcus pneumoniae* and randomized to receive no resuscitation (NR) or resuscitation with a fixed volume (8 ml/kg) of either Ringer’s lactate (RL) or rat derived fresh frozen plasma (rFFP). Healthy controls received sham inoculation. Five hours after start of resuscitation, animals were infused with FITC-dextran and sacrificed. Pulmonary FITC-dextran leakage and plasma levels of thrombomodulin and syndecan-1 were measured as markers of endothelial injury. To assess organ injury, pulmonary wet/dry ratios and histopathology injury scores were measured.

**Results:** Pneumosepsis increased median lactate levels (2.77 vs 1.56 mmol/l, *p* = 0.001), lung wet/dry ratios (5.6 vs 4.3, *p* < 0.01) and pulmonary FITC leakage (134 vs 52 µg/mL, *p* = 0.01) compared to healthy controls (Fig. 1 (abstract P182)). rFFP did not reduce median pulmonary FITC-leakage (58 vs 87 µg/ml, *p* = 0.39), thrombomodulin (*p* = 0.70) or syndecan-1 levels (*p* = 0.98), or pulmonary wet/dry ratios (*p* = 0.16) compared to animals receiving RL (Fig. 1). rFFP did significantly reduce pulmonary FITC leakage compared to animals receiving no resuscitation (*p* =  < 0.05).

**Conclusions:** Plasma resuscitation did not limit endothelial and organ injury compared to crystalloids in a pneumosepsis rat model.

**Figure Figbs:**
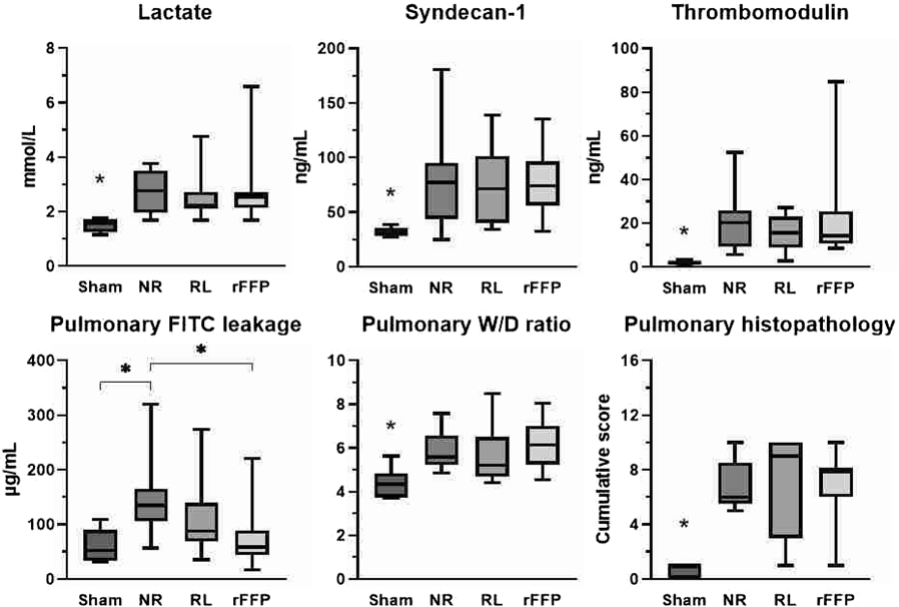
**Fig. 1 (abstract P182)**. The effects of rat fresh frozen plasma (rFFP), Ringer’s lactate (RL) and no resuscitation (NR) on endothelial and organ injury

## P183

### Rapid infusion of Ringer’s lactate solution at different temperatures and the effects on circulation in healthy volunteers: a randomized crossover trial

#### PB Biesenbach^1^, M Bitten Mølmer^2^, E Lobner Svendsen^2^, G Berg-Beckhoff^3^, D Teichmann^4^, LE Lilholm Laugesen^1^, M Rahbek Kristensen^1^, M Brabrand^2^

##### ^1^Esbjerg Hospital, Department of Emergency Medicine, Esbjerg, Denmark, ^2^Odense University Hospital, Department of Emergency Medicine, Odense, Denmark, ^3^University of Southern Denmark, Unit for Health Promotion Research, Odense, Denmark, ^4^University of Southern Denmark, Department of Health Informatics and Technology, Odense, Denmark

*Critical Care* 2023, **27(S1)**: P183

**Introduction:** Intravenous fluids are routinely administered to treat hypotension. However, increases to blood pressure and other hemodynamic changes are limited in duration and scope. This study investigates if changes to mean arterial pressure (MAP) after fluid bolus (FB) are altered by fluid temperature.

**Methods:** Eighteen healthy volunteers are randomly allocated to 30 ml/kg Ringer’s lactate at cold (15 °C) or body (37 °C, warm) temperature over 30 min. For 2 h after starting the FB, we measured MAP, systolic blood pressure, diastolic blood pressure and pulse rate continuously. We recorded temperature every 15 min. In a second session, volunteers crossed over.

**Results:** 15 min after the FB was administered, mean MAP increased more with cold fluids (+ 6.5 mmHg [95% CI 4.8–8.2]) vs warm fluids (+ 0.6 mmHg [95% CI −1.6 to 2.8]; *P* < 0.001) (Fig. 1). Furthermore, a minimum increase in MAP of ≥ 5 mmHg was observed over longer periods of time after cold FB (51 min [95% CI 38–65]) vs warm FB (11 min [95% CI 0–21]; *p* < 0.001). Pulse rate decreased similarly in both groups (−8.8 beats/min [95% CI −4.8 to −12.8] versus −5.4 beats/min [95% CI −1.7 to −9.2]; *p* = 0.35). The temperature decreased more drastically after cold FB (−0.3°Celsius [95% CI −0.1 to −0.4]) vs warm FB (−0.1°C [95% CI 0 to −0.2], *p* = 0.02).

**Conclusions:** Intravenous FB at cold temperature leads to a greater increase in MAP compared with body temperature, and the effect is more prolonged. These findings imply that in healthy volunteers, when a cold temperature FB is given, the temperature of the fluid rather than its volume accounts for most of the MAP increase.

**Figure Figbt:**
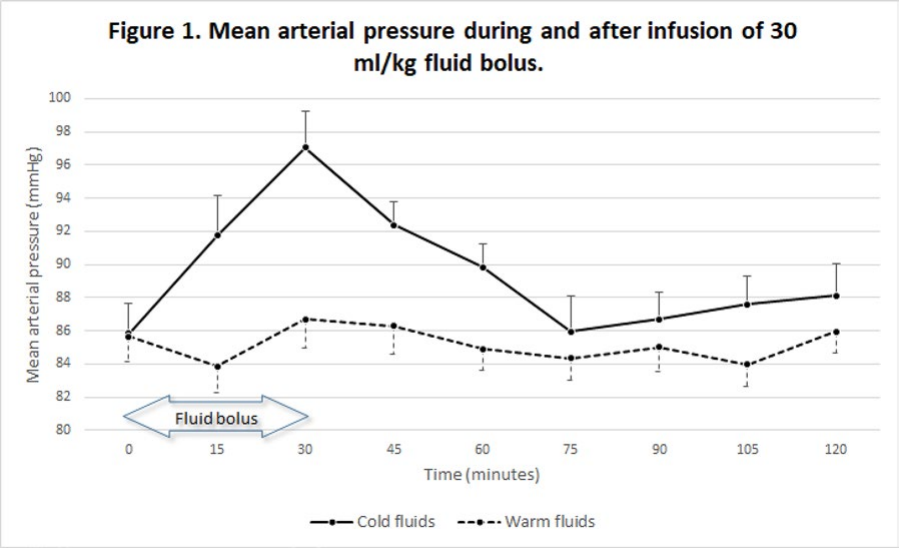
**Fig. 1 (abstract P183)**. Mean (standard error of the mean) of MAP (mean arterial pressure) during the 30 min of fluid bolus administration and observational period in subjects receiving a warm (dotted line) and cold (solid line) fluid bolus, respectively

## P184

### Effect of isotonic sodium bicarbonate infusion on perioperative acid–base status among patients undergoing emergency laparotomy surgery for acute abdomen: a randomised controlled trial

#### A Singh^1^, RK Chaudhary^1^, NB Naik^1^, YR Sakaray^2^

##### ^1^Post Graduate Institute of Medical Education & Research Chandigarh, Department of Anaesthesia & Intensive Care, Chandigarh, India, ^2^Post Graduate Institute of Medical Education & Research Chandigarh, Department of General Surgery, Chandigarh, India

*Critical Care* 2023, **27(S1)**: P184

**Introduction:** Acid–Base disorders are common among surgical patients undergoing emergency laparotomy. Normal anion gap metabolic acidosis develops due to direct loss of sodium bicarbonate from GIT or administration of chloride rich solutions during resuscitation in these patients.In order to treat this, administering sodium bicarbonate makes physiological sense.

**Methods:** After ethical approval and written informed consent, 90 patients (ages 18 to 60 year) diagnosed with perforated peritonitis and required emergency laparatomy surgery were randomaly allocated in two groups 45 each. Group ISB (isotonic sodium bicarbonate group) received 2 ml/kg/hr 1.3% isotonic bicarbonate and group BSS (balanced salt solution group) received 2 ml/kg/hr Ringer lactate (RL) as maintenance fluid during the intraoperative period. Primary outcome was to compare the difference in base excess (BE) between the two groups at the end of surgery. Secondary outcomes (pH, pCO2, HCO3, lactate levels, postoperative duration of mechanical ventilation, ionotrope requirement, ICU/HDU stay and incidence of acute kidney injury) were observed and compared at different time intervals between the groups.

**Results:** The BE, median (Q1, Q3) was −7.30(−8.50, −6.30) in the ISS and −4.80(−6.80, −4.10) in the BSS group (*p* = 0.017) (Fig. 1). The median (Q1, Q3) pH, HCO_3_ and lactate values [7.3 (7.27, 7.32) vs 7.33 (7.31, 7.35); 18.1 (17.3, 19.3) vs 19.0 (18.1, 20.2); 1.6 (1.3, 2.1) vs 1.3 (1.1, 1.89)] were found significantly different at the end of surgery between group ISS and BSS respectively. The difference in duration of mechanical ventilation, ionotrope requirement and ICU/HDU stay were also found significant between the groups.

**Conclusions:** Intraoperative administration of isotonic sodium bicarbonate infusion improves perioperative acis-base status among patients undergoing emergency laparotomy.

**Figure Figbu:**
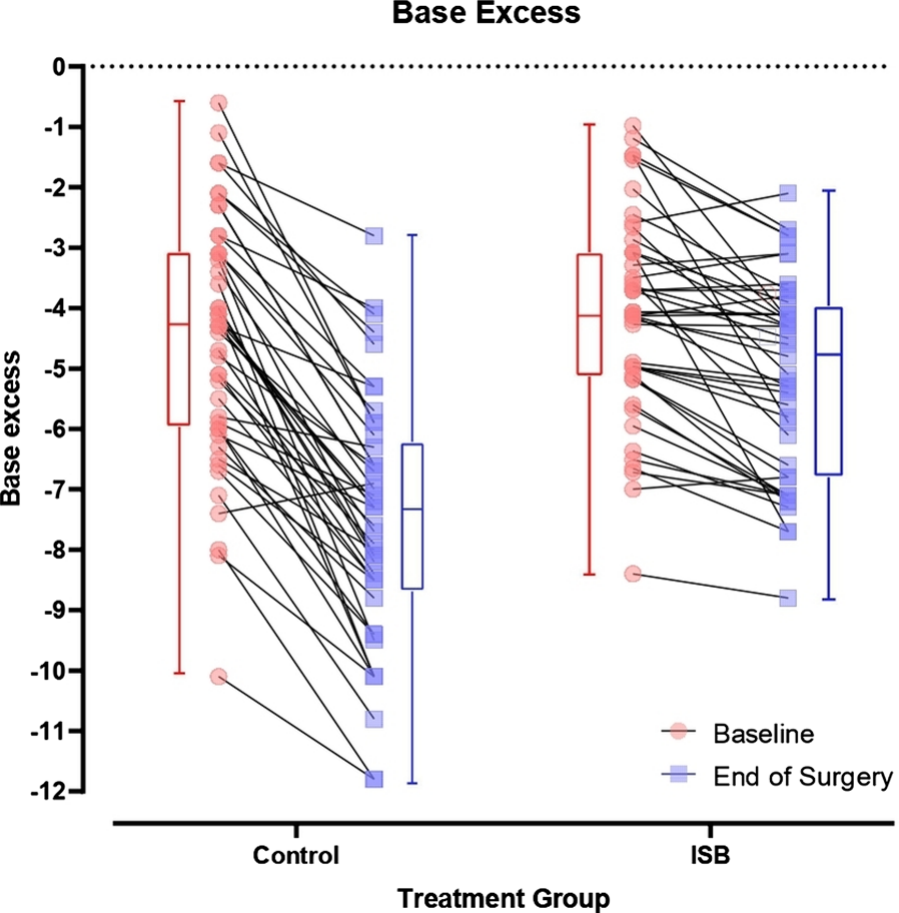
**Fig. 1 (abstract P184)**. The BE values at the end of the surgery among the Study and the Control group

## P185

### Investigating the incidence of hypophosphatemia after elective on-pump cardiac surgery and the response to phosphate substitution: a retrospective single centre cohort study

#### T Vansteenkiste^1^, U Janssens^2^, S Van de Velde^1^, W Haerens^3^, J Heerman^1^

##### ^1^AZ Maria Middelares, Intensive Care, Gent, Belgium, ^2^Vrije Universiteit Brussel, Anesthesiology, Brussel, Belgium, ^3^AZ Maria Middelares, Data Science, Gent, Belgium

*Critical Care* 2023, **27(S1)**: P185

**Introduction****:** The aim of this study was to investigate the incidence of hypophosphatemia after elective on-pump cardiac surgery and the response to phosphate substitution.

**Methods:** We conducted a retrospective single centre cohort study at AZ Maria Middelares among patients who underwent elective on-pump cardiac surgery from January 1st 2019 until January 1st 2022. Inclusion criteria were: age ≥ 18 years; serum phosphate concentrations available from the day of surgery (pre- and postoperative) and the first postoperative day. Hypophosphatemia was defined as mild (0.64–0.8 mmol/l), moderate (0.32–0.63 mmol/l) or severe (< 0.32 mmol/l). Exclusion criteria were: chronic kidney disease (eGFR < 45 ml/min) and preoperative hypo- or hyperphosphatemia. The primary endpoint was hypophosphatemia immediately after surgery. The secondary endpoint, which was analysed in the subgroup of patients with hypophosphatemia, was persistent hypophosphatemia on day 1.

**Results:** The incidence of immediate postoperative hypophosphatemia was 62.0% (623/1005), of which most was mild or moderate. Patients with hypophosphatemia immediately after surgery were divided into two groups: phosphate substitution vs. no substitution (Fig. 1). The incidence of persistent hypophosphatemia on day 1 in both groups was low and did not significantly differ from each other (*p* = 0.1966; Fisher’s Exact Test; two tailed test; *α* = 0.05).

**Conclusions:** Hypophosphatemia is common after on-pump cardiac surgery (62.0%). In contrast, persistent hypophosphatemia on the first postoperative day was uncommon (4.0%) despite only 19.3% of patients receiving substitution. In hypophosphatemic patients, substitution of phosphate did not significantly lower the incidence of persistent hypophosphatemia in comparison to those who did not receive substitution.

**Acknowledgement**: T Vansteenkiste and U Janssens contributed equally as first authors.

**Figure Figbv:**
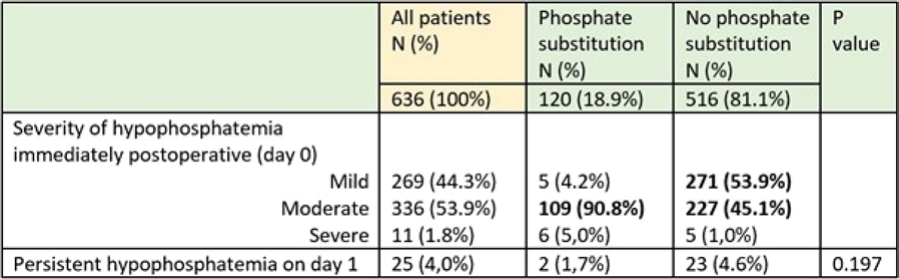
**Fig. 1 (abstract P186)**. Results

## P186

### Hypokalemia and hypophosphatemia in the ICU: a retrospective analysis of incidence and correction in a mixed ICU population

#### KF Bachmann^1^, B Hess^2^, M Mändul^3^, A Bloch^2^, A Regli^4^, A Reintam Blaser^5^

##### ^1^Inselspital, Bern University Hospital, University of Bern, Department of Intensive Care Medicine, Bern, Switzerland, ^2^Lucerne Cantonal Hospital, Department of Intensive Care, Lucerne, Switzerland, ^3^Institute of Genomics, Estonian Genome Center, Tartu, Estonia, ^4^Fiona Stanley Hospital, Department of Intensive Care, Perth, Australia, ^5^University of Tartu, Department of Anaesthesiology and Intensive Care, Tartu, Estonia

*Critical Care* 2023, **27(S1)**: P186

**Introduction:** This retrospective analysis aims to describe the prevalence of hypokalemia (HypoK^+^) and hypophosphatemia (HypoPO_4_^3−^) and to determine cut-offs for overcorrection.

**Methods:** Consecutive patients admitted to a mixed ICU from November 2019 to December 2020 were included for up to a maximum of 96 h. Data on electrolyte administration were collected from the minimum below threshold (hypo) up to the end of the study period or, if correction or overcorrection occurred, up to the first value above threshold if substitution occurred. AUC-ROC curves with an index of union were used to define cut-off points between normalization and overcorrection.

**Results:** 2056 patients were included. Median time of analysis was 5.6 [2.7–11.9] hours for K^+^ and 15.1 [8.4–24.2] hours for PO_4_^3−^. 365 (17.8%) patients had HypoK^+^, of which 38 (10.4%) remained with hypoK^+^, 238 (65.2%) normalized values and 89 (24.4%) developed hyperkalaemia. 575 (30.0%) patients had HypoPO_4_^3−^, of which 276 (48.0%) remained hypo, 249 (43.3%) normalized PO_4_^3−^and 50 (8.7%) developed hyperphosphataemia. 76 (K^+^ group) and 258 patients (PO_4_^3−^ group) had no substitution. Of these, 70 (92.1%, K^+^) and 124 (48.1%, PO_4_^3−^) normalized spontaneously. The remaining patients receiving substitution are presented in Fig. 1. For HypoK^+^ we identified a cut-off for overcorrection of 30 mmol for all grades (AUC-ROC 0.773 [95% CI 0.692–0.821]), 22 mmol for grade 1 (0.791 [0.704–0.852]) and 35 mmol for grade 2 (0.765 [0.612–0.871]). For HypoPO_4_^3−^ the cut-offs were 45 mmol for all grades (0.753 [0.67–0.816]), 29 mmol for grade 1 (0.718 [0.588–0.822]) and 55 mmol for grade 2 (0.761 [0.629–0.869]).

**Conclusions:** This study shows that HypoK^+^ and HypoPO_4_^3−^ are common and were mostly treated successfully, but overcorrection occurred. Cut-offs to reduce overcorrection may be defined using this retrospective dataset.

**Figure Figbw:**
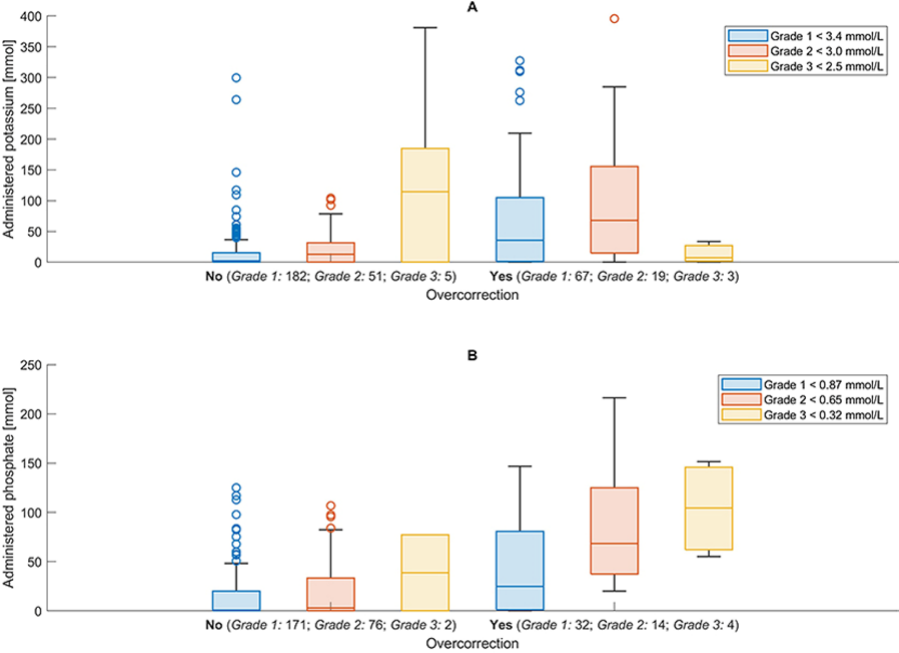
**Fig. 1 (abstract P186)**. Administration of K + and PO43- during the time of analysis, grouped by grade (HypoK + (**A**) and HypoPO4^3-^ (**B**)) and overcorrection

## P187

### Detailed analysis of the time course of metabolic derangements in patients admitted to the ICU with diabetic ketoacidosis

#### MW Van Hasselt^1^, JE Kootstra^2^, MWN Nijsten^1^

##### ^1^University Medical Center Groningen, Critical Care, Groningen, Netherlands, ^2^University Medical Center Groningen, Laboratory Medicine, Groningen, Netherlands

*Critical Care* 2023, **27(S1)**: P187

**Introduction:** Current guidelines advise the use of isotonic saline for resuscitation of patients with diabetic ketoacidosis (DKA). Recent evidence shows this may be associated with (iatrogenic) hyperchloremia [1, 2].

**Methods:** We conducted a single center, retrospective analysis of patients treated for DKA between 2000 and 2021. Selection criteria were plasma glucose > 20 mmol/l and pH < 7.1. Standard of care consisted of fluid resuscitation with NaCl 0.9% and titrated insulin suppletion. Blood glucose measurements were done by full blood gas analysis. Outcome variables were glucose, pH, sodium, chloride and anion gap (calculated as [Na^+^]–[Cl^−^]–[bic^−^]). Data were analyzed with SPSS v28.

**Results:** We identified 55 patients who met the inclusion criteria. There were 1081 blood gas samples in the first three days after admission. Forty-two patients (76%) developed hyperchloremia > 107 mmol/l. Within 8 h after initiation of treatment the mean ± SD chloride levels increased from 93 ± 12 to 107 ± 11 mmol/l (*p* < 0.001) with a maximal mean of 116 ± 8 mmol/l at 22 h (Fig. 1). In contrast, sodium levels only increased by 8 mmol/l over the same period (*p* < 0.001). After 17 h the pH had risen above 7.30, and after 16 h the mean glucose level fell below 14 mmol/l. The time to resolution of the DKA, defined as pH > 7.3, bicarbonate > 15 mmol/l and glucose < 14 mmol/l, was approximately 20 h. The anion gap was restored after 20 h.

**Conclusions:** Our detailed retrospective analysis shows that the time to normalization of metabolic derangements is similar to those reported in recent literature. However the use of NaCl 0.9% infusion was associated with a high prevalence of iatrogenic hyperchloremia.


**References**
Carillo AR et al. Ann Pharmacother 2022;56:998–1006.Van Zyl DG et al. QJM 2012;105:337–43.

**Figure Figbx:**
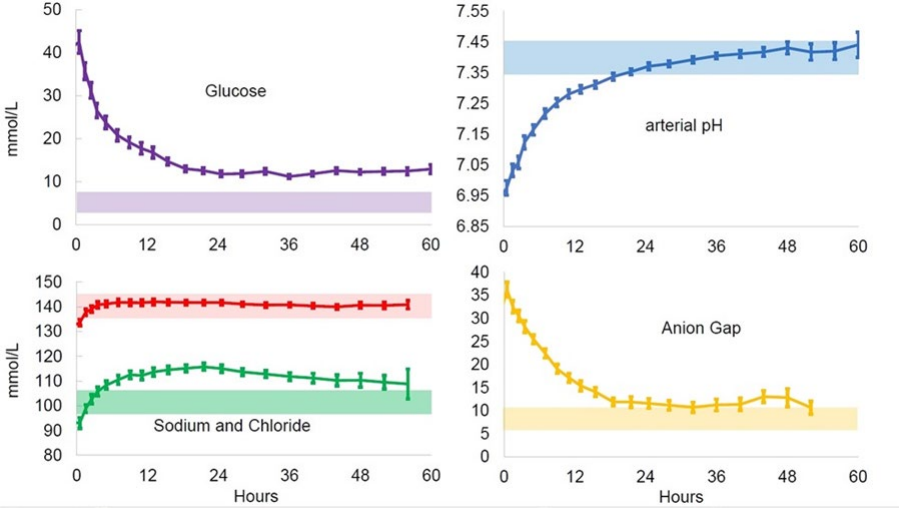
**Fig. 1 (abstract P187)**. Time courses of key parameters in 55 ICU patients after treatment for diabetic ketoacidosis (mean ± standard error of mean)

## P188

### Application of accelerometers for identifying movement related artefacts in general intensive care physiological data

#### I Piper^1^, K Sandall^1^, AB Docherty^2^

##### ^1^University of Edinburgh, Usher Informatics Insitute, Edinburgh BioQuarter, Edinburgh, UK, ^2^University of Edinburgh, Centre for Medical Informatics, The Usher Institute, University of Edinburgh, Senior Clinical Fellow and Consultant in Critical Care, Edinburgh BioQuarter, Edinburgh, UK

*Critical Care* 2023, **27(S1)**: P188

**Introduction:** Analysis and interpretation of high frequency physiological data from the bedside monitors is limited by artefact due to patient movement [1]. Addition of real-time accelerometery may identify movement related artefact and improve quantification of adverse events.

**Methods:** We recruited critically ill patients receiving invasive mechanical ventilation in the Royal Infirmary Edinburgh, Scotland. We placed an accelerometer (Active Insights) on the patient’s chest and recorded high frequency 3-lead electrocardiogram (ECG), invasive arterial blood pressure (BP) and central venous pressure (CVP) waveform resolution data together with accurate annotation of clinical events. Waveform data and annotations were processed and quantified for events that affected a) both accelerometer and physiological signals, b) physiological signals only, c) accelerometer signals only and d) neither type of signal. Time-series and frequency domain features were derived from the raw signals and both mulitinomial regression and random forest machine learning methods were used to study the relationship between clinical events and physiological movement artefact.

**Results:** 72 h of monitoring data from seven patients were collected. There were 152 clinical events annotated. Tracheal suction (n = 31), patient turns (n = 11) and blood sampling (n = 11) were most common. Addition of accelerometer derived features to models compared with physiological derived features alone significantly improved model fit (*p* < 0.001) to the event type. Time-series measures of accelerometer and BP variance and the frequency domain feature ECG to BP coherence (Fig. 1) were the most important (*p* < 0.001) features explaining the event type categorisation.

**Conclusions:** Accelerometer data enables identification of movement artefact in physiological waveform data. This may enable more accurate analysis of physiological adverse events and their impact on patient outcomes.


**Reference**
Donald et al. J Clin Monit Comput. 2019;33:39–51.

**Figure Figby:**
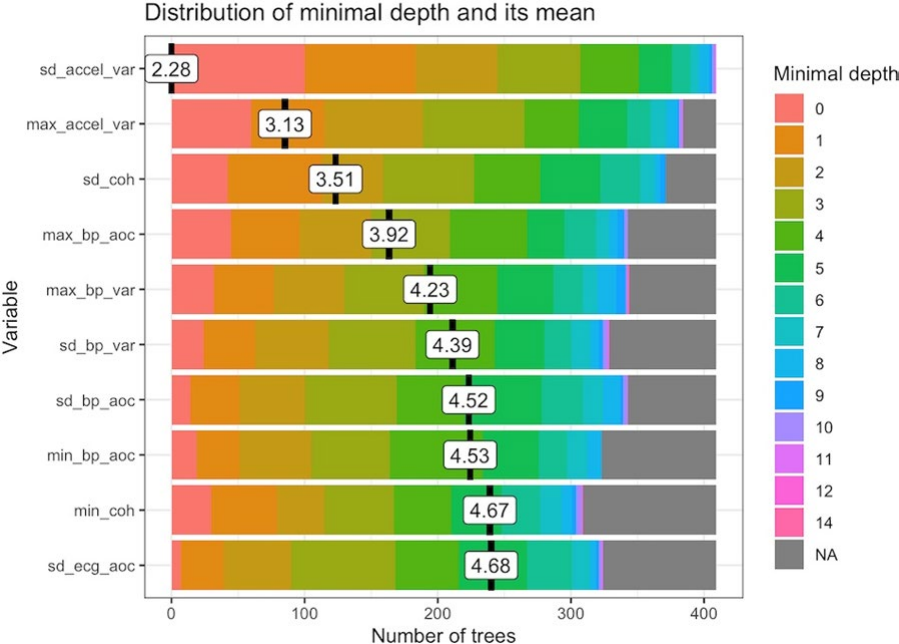
**Fig. 1 (abstract P188)**. Distribution of random forest minimal depth of tree branch by variable (black bar is mean depth). Lower numbers indicate higher importance of variable for categorisation

## P189

### Polydistrectual resistance index evaluation is an assessment of vascular compliance in patients with septic shock treated with vasopressin

#### A Barile^1^, A Recchia^2^, G Paternoster^3^, M Copetti^4^, L Mirabella^5^, G Cinnella^5^, A Del Gaudio^2^

##### ^1^University of Foggia, Departement of Medical and Surgical Servic,Intensive Care Unit, FOGGIA, Italy, ^2^IRCCS “Casa Sollievo della Sofferenza“, Anesthesia and Intensive Care 2, San Giovanni Rotondo, Italy, ^3^Cardiovascular Anaesthesia and ICU, Potenza, Italy, ^4^IRCCS “Casa Sollievo della Sofferenza“, Unit of Biostatistics, San Giovanni Rotondo, Italy, ^5^University of Foggia, Department of Anesthesia and Intensive Care, FOGGIA, Italy

*Critical Care* 2023, **27(S1)**: P189

**Introduction:** Surviving Sepsis Campaign recomends using norepinephrine (NE) as the first-line vasopressor to restore mean arterial pressure [1]. If mean arterial pressure remains inadeguate SSC suggests adding vasopressin (VA) [2]. Resistance Index (RI) is a power Doppler ultrasound assessment of vascular compliance to detect organ perfusion.

**Methods:** Aim of this study is to compare RI in septic shock patients treated with NE (Group1), NE plus VA since the beginning of vasopressor therapy (Group2) and VA plus NE where VA is added if NE dosage was 20 mcg/min (Group3). RI were measured in renal artery (ARE), radial artery (AR), central retinal artery (CRA),superior mesenteric artery (AMS) at three different time points (T0) before vasopressor therapy, (T1) at 1 h, T2 at 24 h and T3 at 48 h.

**Results:** 48 patients were divided into three groups. 17 patients Group 1; 16 Group 2, 15 Group 3. In Group 1 RI increased from T0 in CRA R[0.90(0.57–1.12)] and ARE L [0.74(0.56–0.92) to T3 in CRA R[0.97(0.97–1.14)] and ARE L [0.96(0.82–1.17)]. In Group 2 RI reduced in AMS, from T0[0.84(0.70,1.02)] to T3[0.75(0.59,0.81)],in CRA R, from T0[0.90(0.57,1.09] to T3[0.79(0.58, 0.87)], in CRA L, from T0[0.91(0.43,1.53)] to T3[0.76(0.58, 0.89]and in ARE L, from T0[0.79(0.58, 0.92)] to T3[0.72(0.59, 0.83)]. In Group 3 RI reduced in AMS, from t0[0.86(0.71,0.93)] to T3 [0.68(0.64,0.81)], in CRA R, from T0 [0.90(0.75,1.12)] to T3 [0.78(0.66,0.88)],in CRA L,from T0[0.96(0.76,1.33)] to T3 [0.96(0.76,1.33)], in ARE L, from T0[0.77(0.66, 0.99)] to T3[0.67(0.61,0.85], in ARE R, from T0[0.82[0.64, 0.90]]to T3[0.70(0.62,0.82)] and in AR R, from T0[1,10(0.81,1.30)] to T3 [0.87(0.64,1.22)].

**Conclusions:** Resistence Index was significantly reduced in patients treated with early synergic administration of NE and VA (Fig. 1). This strategy optimized multiorgan perfusion.


**References**
Evans L. Intesnive Care Med 2021;47:1181–1247.Sacha GL. Crit Care Med. 2022;50:614–623.

**Figure Figbz:**
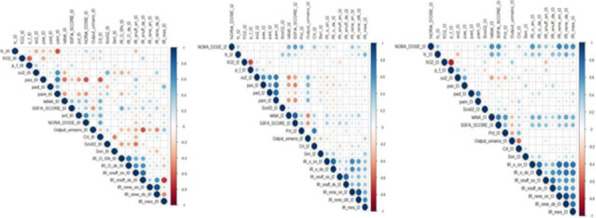
**Fig. 1 (abstract P189)**. Correlations between all the variables analyzed at T0, T2 and T3 (a,b,c). Intensity of Red indicates inverse correlations, Blue indicates direct. There is a greater direct correlation at T3 between the dose of norepinephrine and the Resistance Index. The hypothesis that can be made to explain this is linked to "receptor desensitization", meaning that the dosage of norepinephrine must be increased over time to produce the desired effect (MAP > 65 mmHg), also causing an increase in side effects such as tachyphylaxis, vasoconstriction and therefore the increase in Resistance Indices. IR MES SU*P* = AMS; IR O right/left = CRA R/L; KIDNEY IR right/left = ARE R/L; IR snuff right/left = AR R/L; NORA DOSE = Ne dose

## P190

### Microcirculatory response among patients undergoing elective cardiac surgery with cardiopulmonary-bypass

#### E Favaron^1^, C Ince^2^, J Montomoli^2^, WJ Van Boven^3^

##### ^1^Amsterdam UMC, location AMC, Cardiac Surgery, Amsterdam, Netherlands, ^2^Erasmus MC, Rotterdam, Netherlands, ^3^Amsterdam UMC, location AMC, Amsterdam, Netherlands

*Critical Care* 2023, **27(S1)**: P190

**Introduction:** Fluid administration secondary to hemodynamics impairment and priming solutions together with the use of cardiopulmonary-bypass(CPB) during cardiac surgery are likely to promote hemodilution and inflammatory responses leading to microcirculatory impairment. We used handheld vital sublingual microscopy to examine microvascular modifications.

**Methods:** Sublingual microcirculation was assessed using incident dark field technology on 17 elective open cardiac surgery patients before surgery (T0), after induction of general anesthesia (T1), 10 min following the induction of the CPB (T2), after removal of the aortic clamp (T3), and at the end of surgery (T4). Postoperatively measurements were conducted on the day of discharge from the intensive care unit(T5) and three days after (T6). Microtools [1] and manual analysis were performed for the analysis of microcirculatory tissue perfusion [2] and microcirculatory leukocytes [3], respectively. Noninvasive assessment of the macrohemodynamics and of the body fluid composition were also collected during the study.

**Results:** During surgery, microcirculation showed a general impairment of tissue perfusion when compared to the baseline and in accordance with the macro-hemodynamics(cardiac output and mean arterial pression). In the post-operative days, the microcirculation showed a rapid recover to the baseline levels. Conversely, the inflammatory response at the microcirculatory level peaked at T3 and T4 and persisted also three days after surgery (Fig. 1).

**Conclusions:** Patients undergoing elective CPB during cardiac surgery showed an impairment of microcirculation in term of tissue perfusion, coherent with hemodynamic modification and characterized by relatively fast recovery. On the contrary, microcirculatory impairments due to tissue oedema and leukocyte activation persisted in the post-operative period.


**References**
Hilty MP et al. Crit Care Med 2020;48:e864–e875.Hilty MP et al. Curr Opin Crit Care 2020;26:273–280.Uz Z et al. J Appl Physiol 2018;124:1550–1557.

**Figure Figca:**
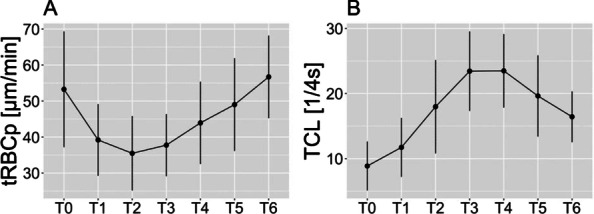
**Fig. 1 (abstract P190)**. **A** Tissue red blood cell perfusion (tRBCp) [2], mean ± sd. **B** Total microcirculatory leukocyte count (TLC) [3], mean ± sd

## P191

### Comparison of microcirculatory changes in the first 24 and 48 h between survivors and non-survivors of septic shock

#### M Briuks^1^, S Kazune^2^

##### ^1^University of Latvia, Student, Riga, Latvia, ^2^Hospital of Traumatology and Orthopedics, Anesthesiology Reanimatology, Riga, Latvia

*Critical Care* 2023, **27(S1)**: P191

**Introduction:** Microcirculatory dysfunction contributes to organ failure and mortality in patients with septic shock. Microcirculatory perfusion targeted resuscitation has been proposed as a tool to improve mortality in these patients. We hypothesized that microcirculation for non-survivers stays unchanged 24 and 48 h after admission compared to baseline, however, in survivors microcirculation improves in the first 48 h.

**Methods:** We included 36 adult sepsis patients admitted to the intensive care unit within the previous 24 h who were receiving a noradrenaline infusion. Skin oxygen saturation in the knee area was measured using a hyperspectral imaging-based method at baseline, after 24 and 48 h. The primary outcome was an increase in skin oxygen saturation depending upon survival status at 28 days.

**Results:** Median skin oxygen saturation changed from 11% (6–26) at baseline to 31 (23–33) % at 48 h in non-survivors and from 30% (23–36) to 28% (23–34) in survivors. There were significant differences in skin oxygen saturation between survivors and non-survivors at baseline (*p* < 0.01), but this difference was lost after 24 and 48 h. Independent baseline skin oxygen saturation predictors were APACHE II score and mean arterial pressure. Low baseline skin oxygen saturation was not associated with an increase in SOFA score over the first 2 days (r = − 0.38, *p* = 0.19).

**Conclusions:** At admission to intensive care, skin oxygen saturation is significantly lower in non-survivors of septic shock. Over the early course of treatment, it increases to survivor levels and is similar in all patients after 48 h of intensive care.

## P192

### Predictive value of central venous-to-arterial carbon dioxide tension in patients with ARDS treated with veno − venous extracorporeal membrane oxygenation (ECMO)

#### P Djimafo^1^, K Kaefer^1^, C Pierrakos^1^, D Velissaris^2^, S Anane^1^, L Barreto Gutierrez^1^, S Redant^1^, A Gallerani^1^, R Attou^1^

##### ^1^CHU Brugmann, ICU, Laeken, Belgium, ^2^University Hospital of Patras, Internal Medicine Department, Patras, Greece

*Critical Care* 2023, **27(S1)**: P192

**Introduction:** Central venous-to-arterial carbon dioxide tension (P_va_CO_2_) can be useful for monitoring adequacy of tissue perfusion in patients with ARDS supported with veno-venous Extracorporeal Membrane Oxygenation (VV-ECMO). However, in theory, the unavoidable mixing of venous blood with blood after the oxygenator can affect P_va_CO_2_ values by increasing central venous oxygen saturation and substantially decreasing CO_2_ concentration. This study aimed to evaluate acute changes in P_va_CO_2_ after VV-ECMO installation and determine its association with patient outcomes.

**Methods:** Retrospectively evaluated coronavirus disease 2019 (COVID–19) ARDS patients with at least one concurrent arterial and central venous blood gas analysis before and after VV-ECMO installation as standard care. The primary outcome was intensive care unit (ICU) mortality at 28 days.

**Results:** 29 patients were enrolled in the study. All the patients had a 25 F drainage multistage femoral cannula and a 21 F internal jugular infusion cannula. The median distance between the central venous sampling point and the tip of the infusion cannula was 39 [23–73] mm. No statistically significant changes in P_va_CO_2_ were observed 24–48 h after VV-ECMO installation (5 [4–7] mmHg to 6.5 [5–8.2] mmHg, *p* = 0.12). Hemoglobin concentration decreased 24 to 48 h after VV-ECMO installation (10.7 [9.5–12.7] g/dl to 9.6 [8.8–11.6] g/dl, *p* < 0.01) but neither central venous (75 [70–81]% to 73 [67–78]%, *p* = 0.46) nor arterial oxygen saturation (95 [92–97]% to 95 [93–96]%, *p* = 0.81) changed significantly. Elevated P_va_CO_2_ after VV-ECMO installation had a good predictive value for 28 day ICU mortality (calculated area under the ROC curve 0.81) (Fig. 1 veno-venous).

**Conclusions:** VV-ECMO support appears to have little effect on the P_va_CO_2_ calculation. P_va_CO_2_ can be used to evaluate patients with ARDS supported with VV-ECMO, as persistently elevated values can be associated with poor outcomes.

**Figure Figcb:**
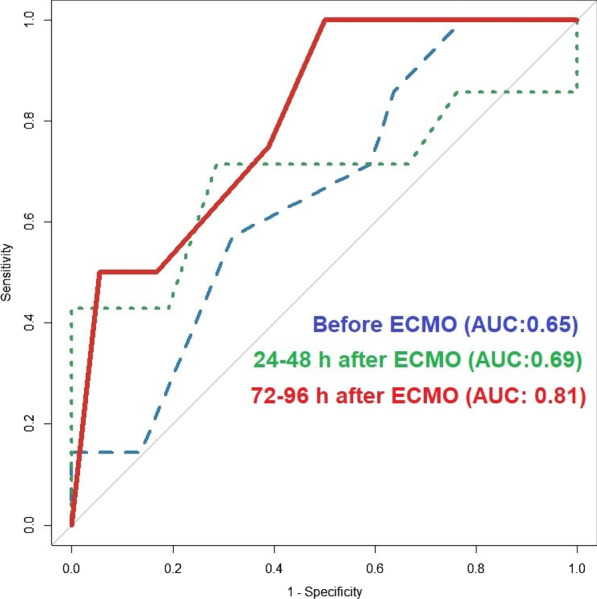
**Fig. 1 (abstract P192)**. ROC curve for PvaCO2 values calculated before and after ECMO installation in predicting 28-day mortality

## P193

### Change in perfusion index after 10-s and 20-s recruitment maneuvers to predict fluid responsiveness in mechanically ventilated patients during elective surgery

#### ST Thertchanakun^1^, SM Morakul^2^, ST Thitilerd^2^, CP Pisitsak^2^, WA Apinyachon^2^, WM Mongkolpun^3^

##### ^1^Faculty of Medicine Ramathibodi Hospital, Mahidol University, Department of Anesthesiology, Bangkok, Thailand, ^2^Faculty of Medicine Ramathibodi Hospital, Mahidol University, Bangkok, Thailand, ^3^Siriraj Piyamaharajkarun Hospital, Siriraj Hospital, Mahidol University, Bangkok, Thailand

*Critical Care* 2023, **27(S1)**: P193

**Introduction:** Excessive volume expansion during surgery worsens outcomes. The perfusion index (PI) can track changes in stroke volume (SV) during recruitment maneuvers (RMs), and thus predict fluid responsiveness. Classic RMs (30 cmH_2_O continuous positive airway pressure (CPAP) for 30 s) can cause hypotension. We evaluated whether ∆PI after RMs of 10 or 20 s could predict fluid responsiveness.

**Methods:** Adult patients having elective surgery under general anesthesia with mechanical ventilation and an arterial line were included. After induction and hemodynamic stabilization (T0), a RM (30 cmH_2_O of CPAP) was performed for 10 s (RM_10_). Hemodynamic variables, pulse pressure variation (PPV) and PI (index finger) were obtained before and during RM_10_ and relative changes calculated. After return of SV to T0 values, a 20-s RM (RM_20_) was performed and the same variables measured. Crystalloid fluid (250 ml over 10 min) was then infused and fluid responsiveness (SV increase ≥ 10%) assessed. SV and PPV were recorded using pulse contour analysis. Areas under the receiver operating characteristic curves (AUCs) of variables (T0) and their changes were calculated.

**Results:** Thirty-two patients were studied: 18 (53%) were fluid responders (FRs). At T0, only PPV was higher in FRs than in non-FRs. SV, mean arterial pressure and PI decreased during RM_10_ and RM_20_, whereas PPV increased. ∆SV (16.8 ± 0.8 vs 36.2 ± 0.7%, *p* < 0.05) and ∆PPV (36.7 ± 1.5 vs 73.4 ± 1.8%, *p* < 0.05) were lower after RM_10_ than after RM_20_. ∆PI were higher in FRs than in non-FRs for RM_10_ and RM_20_ (Table 1 (abstract P193)). PPV at T0 and ∆PI at RM_20_ had the largest AUCs to predict fluid responsiveness (Table 1), with cutoff values ≥ 7.5% (sensitivity (Sn) 56%, specificity (Sp) 86%) and ≥ 36% (Sn 82%, Sp 64%), respectively. AUCs of ∆PI were similar for RM_10_ and RM_20_ (Table 1).

**Conclusions:** ∆PI during a 10 and a 20 s-RM predicted fluid responsiveness as well as baseline PPV did in surgical patients during the perioperative period.

**Table Tabav:** **Table 1 (abstract P193)**. Changes in hemodynamic parameters and their AUCs for predicting fluid responsiveness

Hemodynamic parameters	Fluid responders (n = 18)	Non-fluid responders (n = 14)	*p* value	AUC
∆PIRM10 (%)	45.0 ± 22.2	26.4 ± 19.7	0.02	0.71 [0.52–0.89]
∆PIRM20 (%)	53.2 ± 19.2	31.9 ± 24.0	0.01	0.76 [0.59–0.9]
∆SVRM10 (%)	16.3 ± 12.5	10.6 ± 8.4	0.2	0.63 [0.43–0.83]
∆SVRM20 (%)	44.4 ± 28.5	26.1 ± 21.6	0.1	0.69 [0.52–0.88]
∆PPVRM10(%)	45.2 ± 15.5	24.9 ± 36.9	0.3	0.57 [0.36–0.79]
∆PPVRM20(%)	80.3 ± 65.6	64.6 ± 81.16	0.6	0.57 [0.35–0.78]
PPV (%) at T0	9.5 ± 4.3	5.8 ± 2.3	0.01	0.77 [0.61–0.93]

## P194

### Intraoperative hypotension when using hypotension prediction index software during major non-cardiac surgery: a European multicenter prospective observational registry (EU-HYPROTECT)

#### K Kouz^1^, MI Monge García^2^, E Cerutti^3^, G Draisci^4^, M Sander^5^, UH Frey^6^, SJ Davies^7^, P Bramlage^8^, B Saugel^9^

##### ^1^University Medical Center Hamburg-Eppendorf, Department of Anesthesiology, Hamburg, Germany, ^2^Unidad de Cuidados Intensivos, Hospital Universitario SAS Jerez, Jerez de la Frontera, Spain, ^3^Azienda Ospedaliero Universitaria delle Marche, Department of Anesthesia, Transplant and Surgical Intensive Care, Ancona, Italy, ^4^Fondazione Policlinico Universitario Agostino Gemelli—IRCCS, Department of Emergency, Intensive Care Medicine and Anesthesia, Rome, Italy, ^5^University Hospital Giessen, Department of Anaesthesiology, Intensive Care Medicine and Pain Medicine, Giessen, Germany, ^6^Marien Hospital Herne, Department of Anesthesiology, Intensive Care, Pain and Palliative Care, Herne, Germany, ^7^NHS Foundation Trust, York and Scarborough Teaching Hospitals, York, UK, ^8^ IPPMed, Institute for Pharmacology and Preventive Medicine, Cloppenburg, Germany, ^9^ University Medical Center Hamburg-Eppendorf, Hamburg, Germany

*Critical Care* 2023, **27(S1)**: P194

**Introduction:** Intraoperative hypotension is associated with postoperative complications and mortality. Current intraoperative blood pressure management is mainly reactive—with hypotension being treated only after it has occurred. Predictive hemodynamic monitoring may be a promising approach to help clinicians reduce intraoperative hypotension. The Acumen™ Hypotension Prediction Index software (HPI-software) (Edwards Lifesciences; Irvine, CA, USA) was developed to predict hypotension. We built up the European, multicenter, prospective, observational EU-HYPROTECT registry to describe the incidence, duration, and severity of intraoperative hypotension when using HPI-software monitoring in patients having non-cardiac surgery.

**Methods:** We enrolled 749 patients scheduled for elective major non-cardiac surgery in 12 medical centers in 5 European countries. All patients had intraarterial blood pressure monitoring with an arterial catheter and intraoperative HPI-software monitoring. The primary endpoint of this registry was intraoperative hypotension quantified using the time-weighted average mean arterial pressure (MAP) < 65 mmHg. Secondary endpoints included the proportion of patients with at least one ≥ 1-min-episode of a MAP < 65 mmHg, and the number of ≥ 1-min-episodes of a MAP < 65 mmHg.

**Results:** Between September 2021 and May 2022, we enrolled 749 patients in the registry and included 702 patients in the final analysis. The median time-weighted average MAP < 65 mmHg was 0.03 (0.00, 0.20) mmHg (Fig. 1). 285 patients (41%) had no ≥ 1-min-episode of a MAP < 65 mmHg—417 patients (59%) had at least one. The median number of ≥ 1-min-episodes of a MAP < 65 mmHg was 1 (0, 3).

**Conclusions:** The median time-weighted average MAP < 65 mmHg was very low in patients in this registry. This suggests that using HPI-software monitoring may help reduce the duration and severity of intraoperative hypotension in patients having non-cardiac surgery.

**Figure Figcc:**
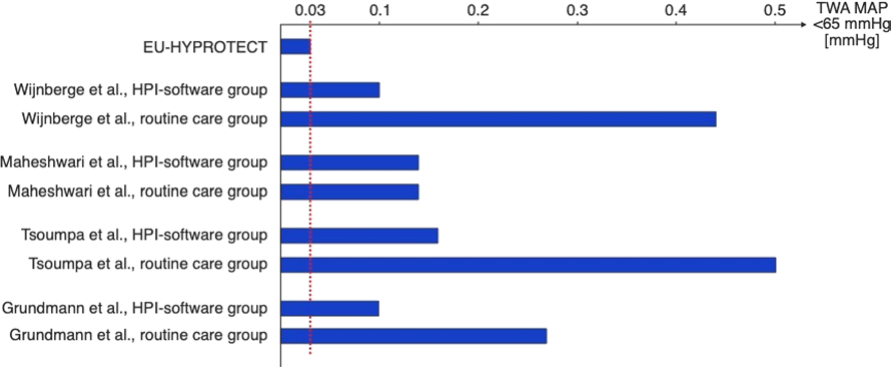
**Fig. 1 (abstract P194)**. Bar chart illustrating the time-weighted average mean arterial pressure (TWA MAP) < 65 mmHg in patients of this registry and of previously published studies on HPI-software monitoring

## P195

### Left ventricular function monitored in intensive care using artificial intelligence and transesophageal echocardiography

#### J Yu^1^, H Flade^1^, A Taskén^2^, EA Berg^3^, B Grenne^3^, A Rimehaug^1^, I Kirkeby-Garstad^4^, G Kiss^2^, S Aakhus^3^

##### ^1^Norwegian University of Science and Technology/St Olav’s Hospital, Department of Circulation and Medical Imaging/Department of Anesthesia and Intensive Care, Trondheim, Norway, ^2^Norwegian University of Science and Technology, Department of Computer Science, Trondheim, Norway, ^3^Norwegian University of Science and Technology/St Olav’s Hospital, Department of Circulation and Medical Imaging/Clinic of Cardiology, Trondheim, Norway, ^4^Norwegian University of Science and Technology, Department of Circulation and Medical Imaging, Trondheim, Norway

*Critical Care* 2023, **27(S1)**: P195

**Introduction:** Left ventricular (LV) function is usually not assessable by standard hemodynamic monitors. To automatically monitor LV function, we have developed a method for measuring mitral annular plane systolic excursion (MAPSE) by combining transesophageal echocardiography with artificial intelligence: *AutoMAPSE* (Fig. 1A). Our objective is to assess the feasibility of AutoMAPSE in intensive care, and to compare its bias and least significant change (LSC) against manual measurements.

**Methods:** We obtained midesophageal two- and four-chamber views of three beats in 40 intensive care patients. Off-line, we measured MAPSE manually and with AutoMAPSE in four LV walls in all available beats. We defined AutoMAPSE as *feasible* when it was obtainable from at least one beat in one wall. To assess *bias* and limits of agreement (LOA)*,* we used Bland–Altman analysis. We calculated *LSC* for each method, which is the least difference between two measurements required before a change is considered real.

**Results:** AutoMAPSE had a feasibility of 96.2%. Bland–Altman analysis showed that AutoMAPSE had a low bias (Fig. 1B), ranging from −0.2 mm (anterior wall, LOA:  −3.4 to 3.1 mm) to −1.1 mm (septal wall, LOA:  −4.1 to 2.4 mm). AutoMAPSE had higher LSC than manual measurements when comparing a single measurement (5.4 vs 4.7 mm). When AutoMAPSE is set to measure more beats than manual, its LSC is reduced (LSC for two beats: 3.8 mm).

**Conclusions:** AutoMAPSE is feasible in intensive care patients. Compared to manual measurements, AutoMAPSE had low bias. By using AutoMAPSE to measure more beats, its LSC is reduced.

**Figure Figcd:**
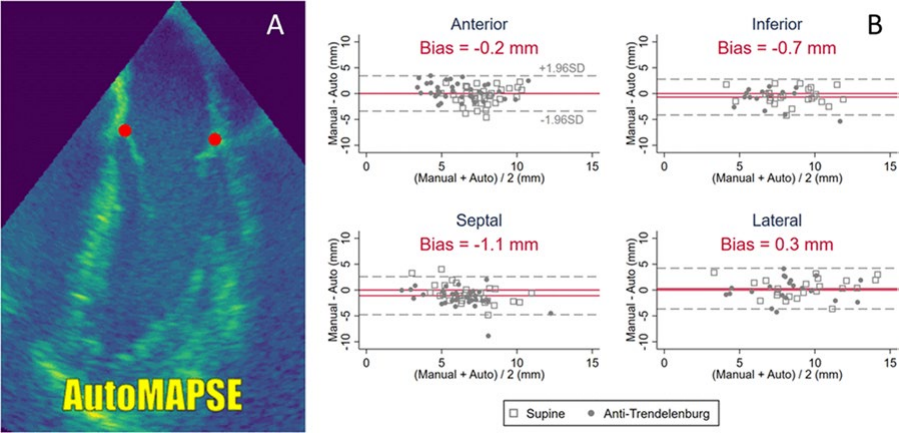
**Fig. 1 (P195)**. **A** Left ventricular function monitored using AutoMAPSE. Red dots show the detection of mitral annuli in a midesophageal four-chamber view. **B** Bland–Altman plots of two methods. Red line, bias. Dashed lines, 95% limits of agreement

## P196

### Vascular access for all

#### D Cloete, G Barker

##### John Radcliffe Hospital, Critical Care, Oxford, UK

*Critical Care* 2023, **27(S1)**: P196

**Introduction:** Peripheral (PVC) and central (CVC) venous cannulation are key components of care for many ED and ICU patients. PVC is typically obtained using direct visualization and physical palpation; and performing CVC under ultrasound-guidance (compared to the landmark technique) is the modern gold standard. Ultrasound improves success rates, particularly in patients with difficult IV access. Appropriate training is pertinent for all ED/ICU practitioners, and procedural proficiency is expected. Many departments have ultrasound machines, yet the vast majority of doctors are engaging in self-directed learning in this regard. We evaluated the training received by doctors in ultrasound-guided (U/S) PVC and CVC.

**Methods:** An electronic survey was disseminated to all ranks of practitioner from several hospital departments, and reviewed levels of training, competence and procedural preferences in both U/S-PVC and CVC.

**Results:** A total of 148 eSurvey responses were received (response rate of 52%). Regarding U/S-PVC, 68% of respondents had never been taught with 65% achieving this previously, and 12% attempting to do so but failing. For CVC, 61% have inserted them. Of these, 84% always use ultrasound and 16% elect to use either ultrasound or an anatomical landmark-based technique. 55% have undergone formal training. Despite this, 10,2%, 9,6% and 11.3% of these individuals have not received training in the procedure itself, sterility during the procedure or post CVC protocols, respectively. When reviewing operator preference for specific U/S-CVC sites, participants expressed varying levels of confidence (see Fig. 1). Respondents wishing to receive formal, comprehensive training on U/S-PVC and U/S-CVC totaled 72% and 76%, respectively.

**Conclusions:** U/S-PVC & CVC ability varied significantly in a large academic hospital. A large training gap exists, particularly for those previously taught U/S-CVC to ensure universal, comprehensive proficiency. This dataset will be used to launch an U/S-vascular access course in the region.

**Figure Figce:**
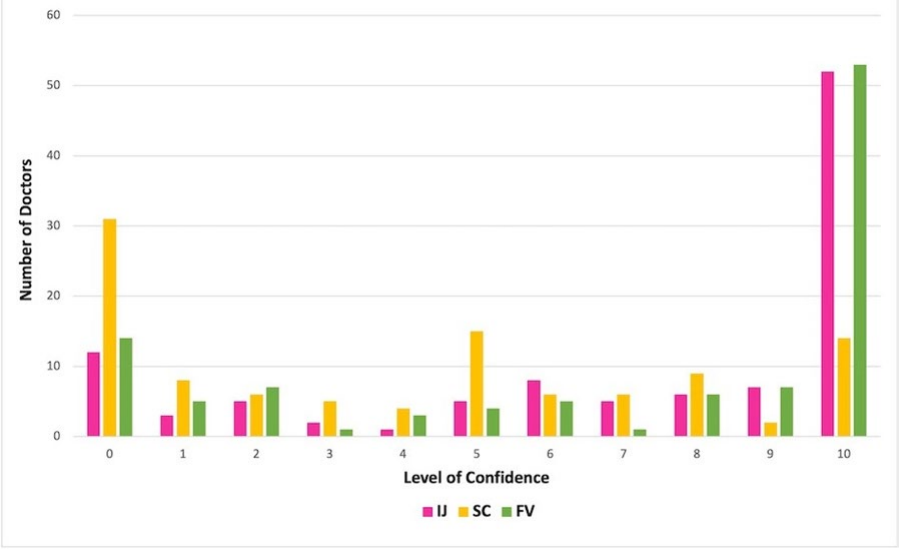
**Fig. 1 (abstract P196)**. Confidence levels of doctors performing central venous cannulation

## P197

### Evaluation of the image quality and validity of handheld echocardiography for stroke volume and left ventricular ejection fraction quantification: a method comparison study

#### FM de Raat^1^, J Van Houte^2^, LJ Montenij^2^, S Bouwmeester^3^, SEA Felix^3^, EC De Boer^1^, P Bingley^4^, P Houthuizen^3^, RA Bouwman^5^

##### ^1^University of Eindhoven, Electrical engineering, Eindhoven, Netherlands, ^2^Catharina Hospital, Anesthesiology and Intensive Care, Eindhoven, Netherlands, ^3^Catharina hospital, Cardiology, Eindhoven, Netherlands, ^4^Philips, Patient Care and Monitoring, Eindhoven, Netherlands, ^5^Catharina Hospital, Anesthesiology, Eindhoven, Netherlands

*Critical Care* 2023, **27(S1)**: P197

**Introduction:** Bedside quantification of stroke volume (SV) and left ventricular ejection fraction (LVEF) is valuable in hemodynamically compromised patients. Miniaturized, battery operated handheld ultrasound (HAND) devices are now available for clinical use. However, the performance level of HAND devices for automated quantified cardiac assessment is yet unknown. The aim of this study was to compare the validity of HAND measurements with Standard Echocardiography (SE) and three-dimensional echocardiography (3DE).

**Methods:** Thirty-six patients were scanned with HAND, SE and 3DE. The image quality of HAND and SE apical four and two chamber views were evaluated by scoring segmental endocardial border delineation (2 = good, 1 = poor, 0 = invisible). LVEF and SV of HAND was evaluated against SE and 3DE using correlation and Bland–Altman analysis. HAND and SE datasets were analysed automatically with Auto Strain. The 3DE dataset was analysed automatically with the Dynamic Heart Model.

**Results:** The correlation, bias, and limits of agreement (LOA) between HAND and SE were 0.68 [0.46:0.83], 1.60% [−2.18:5.38], and 8.84% [−9.79:12.99] for LVEF, and 0.91 [0.84:0.96], 1.32 ml [−0.36:4.01], 15.54 ml [−18.70:21.35] for SV (Fig. 1), respectively. Correlation, bias, and LOA between HAND and 3DE were 0.55 [0.6:0.74], −0.56% [−2.27:1.1], and 9.88% [−13.29:12.17] for LVEF, and 0.79 [0.62:0.89], 6.78 ml [2.34:11.21], 12.14 ml [−26.32:39.87] for SV, respectively. The image quality scores were 9.42 ± 2.0 for the apical four chamber views of the HAND dataset and 10.49 ± 1.7 for the SE dataset and (*p* < 0.001).

**Conclusions:** Clinically acceptable accuracy, precision, and image quality was demonstrated for HAND measurements compared to SE. Also, LVEF quantification with HAND showed a clinically acceptable accuracy and precision compared to 3DE.

**Figure Figcf:**
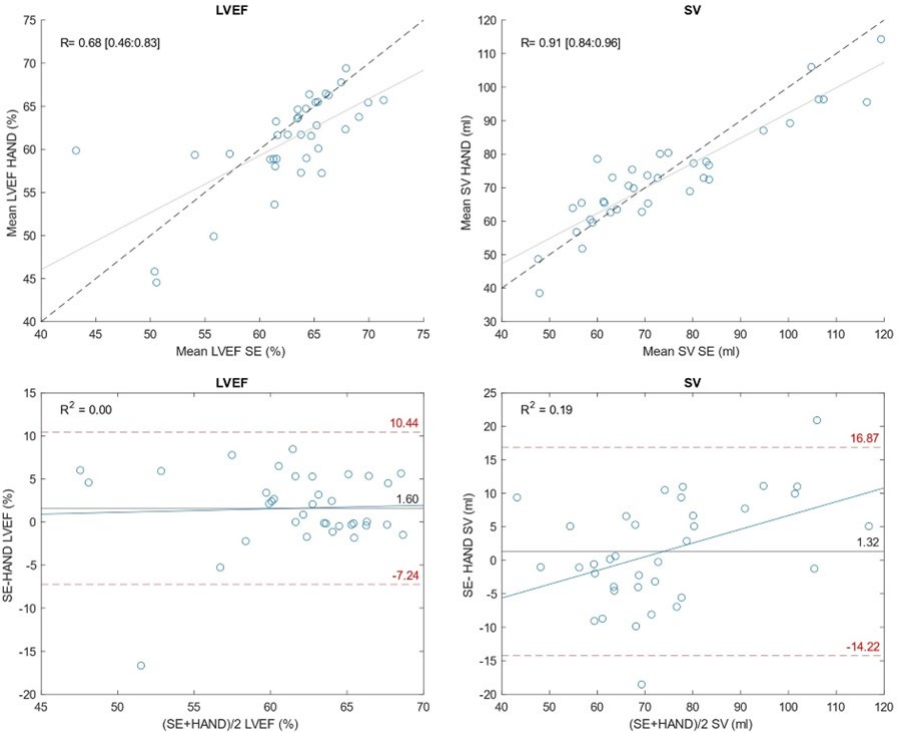
**Fig. 1 (abstract P197)**. Upper panel: Correlation analysis of the HAND and SE data for (**A**) LVEF, and (**B**) SV. Lower panel: Bland–Altman plots of HAND versus SE for (**C**) LVEF, and (**D**) SV. LVEF = left ventricle ejection fraction; SV = stroke volume; R = correlation coefficient, R2 = Regression coefficient

## P198

### Smartphone-based assessment of left ventricular ejection fraction using a silicon chip ultrasound pocket probe and a machine learning algorithm

#### J Leote^1^, FA Gonzalez^1^, J Bacariza^1^, R Varudo^1^, C Martins^1^, RM Meireles^1^, A Fernandes^1^, F Michard^2^

##### ^1^Hospital Garcia de Orta, EPE., Critical Care, Almada, Portugal, ^2^MiCo, Denens, Switzerland

*Critical Care* 2023, **27(S1)**: P198

**Introduction:** Our study aims to compare a smartphone-based assessment of left ventricular ejection fraction with a silicon-chip ultrasound (US) probe and a machine learning algorithm (LVEF_SMART_) to reference manual measurements (LVEF_REF_) done with a high-end US.

**Methods:** We studied critically ill patients requiring an echocardiography. LVEF_SMART_ measurements were made with a smartphone algorithm (Caption AI, Caption Health, USA) connected to a US probe (IQ, Butterfly Inc., USA). LVEF_SMART_ data was compared with LVEF_REF_ done by two experts using a conventional US (Venue, GE Healthcare, USA). Measurements were done in triplicate from a 4-chamber apical view and intra-operator reproducibility was also calculated.

**Results:** We enrolled 95 patients (mean age 60 ± 17 years), 34% were mechanically ventilated and 31% received continuous vasopressor support. LVEF_REF_ ranged from 26 to 80% (mean 54 ± 12%) and the reproducibility was 9 ± 6%. Whereas LVEF_SMART_ ranged from 28 to 79% (mean 54 ± 12%) with the reproducibility of 8 ± 6%. A fair relationship (r = 0.75, *p* < 0.001) between LVEF_REF_ and LVEF_SMART_. The average difference (bias) between LVEF_SMART_ and LVEF_REF_ was 0 ± 8% (95% agreement limits of −17 to +16%). Thirty patients had a LVEF_REF_ < 50% considered as left systolic dysfunction. LVEF_SMART_ detected dysfunction with a sensitivity of 70% and specificity of 89%.The algorithm measured LVEF_SMART_ automatically in 45 patients (fully-automatic measurements). In the remaining 50 patients, the algorithm enabled automatic left ventricular border detection, but end-diastolic and end-systolic images were selected by the operator (semi-automatic measurements). The agreement between LVEF_SMART_ and LVEF_REF_ was better for fully automatic than for semi-automatic measurements (Fig. 1).

**Conclusions:** The accuracy of LVEF_SMART_ showed no bias and a high specificity to detect left ventricular dysfunction. However, sensitivity was low with a wide range of agreement limits, particularly for semi-automatic measurements.

**Figure Figcg:**
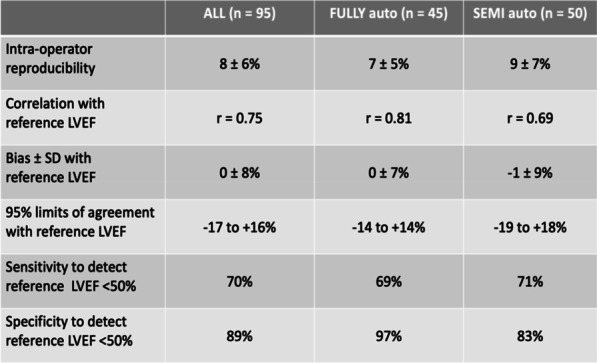
**Fig. 1 (abstract P198)**. Reproducibility, correlation, bias, and agreement between reference and semi-automatic measurements

## P199

### The evaluation of cardiac functions in deep Trendelenburg position during robotic-assisted laparoscopic prostatectomy

#### E Kilinc^1^, S Aktas Yildirim^1^, H Ulugol^1^, E Eroglu Buyukoner^2^, B Gucyetmez^1^, F Toraman^1^

##### ^1^Acibadem Mehmet Ali Aydinlar University School of Medicine, Department of Anesthesiology and Reanimation, Istanbul, Turkey, ^2^Acibadem Mehmet Ali Aydinlar University School of Medicine, Department of Cardiology, Istanbul, Turkey

*Critical Care* 2023, **27(S1)**: P199

**Introduction:** In this study, we investigated how to affect invasive cardiac parameters achieved on invasive artery monitoring by using Pressure Recording Analytical Method (PRAM) in deep Trendelenburg (dTD) position during robotic-assisted laparoscopic prostatectomy (RALP). In RALP, dTD position (25°–45°) is performed following pneumoperitoneum [1]. In dTD position, although protection of cardiac output is expected caused by increased venous return, a decrease in hemodynamic parameters can be seen because of gravity and increased intrathoracic pressure [2].

**Methods:** After ethical approval, this study was designed as a prospective observational study. Thirty patients with malignant prostate cancer who were > 18 years old, and had no chronic heart failure, valvular disease, arrhythmia, and myocardial ischemia in the last 3 months were included in the study. All invasive cardiac parameters and longitudinal strain (SL) achieved transesophageal echocardiography were recorded in pre-dTD (T_3_) and 10^th^ minute of dTD (T_4_). Delta values were calculated for CCE and SL (values at T_4_ minus values at T_3_). The estimated power was calculated as 1.0 in accordance with cardiac cycle efficiency (CCE) values at T_3_ and T_4_ (Effect size: 0.85 standard deviations of the mean difference: 0.22, alfa:0.05).

**Results:** At T_4_, heart rate, pulse pressure variation, CCE, dP/dt and SL were significantly lower than those at T_3_ (*p* = 0.009, *p* < 0.001, *p* < 0.001, and *p* < 0.001 respectively). There was a positive correlation between delta-CCE and delta-SL (r^2^ = 0.36, *p* < 0.001).

**Conclusions:** Cardiac workload is increased in dTD position and using traditional monitoring is not enough to evaluate cardiac function in this position. CCE can be used to evaluate changed cardiac workload and changes in CCE are compatible with SL. Therefore, we suggest monitoring CCE in especially risky positions during surgeries.


**References**
Ficarra V et al. Eur Urol 2009;55:1037–63.Lestar M et al. Anesth Analg. 2011;5:1069–1075.


## P200

### Machine learning-driven early detection of bleeding events in a human bleeding model

#### A Dey^1^, J Kim^2^, X Li^3^, M Hravnak^4^, MR Pinsky^5^, G Clermont^5^, A Dubrawski^3^, JH Yoon^2^

##### ^1^Georgia Institute of Technology, Department of Biomedical Engineering, Atlanta, USA, ^2^University of Pittsburgh, Pulmonary and Critical Care Medicine, Pittsburgh, USA, ^3^Carnegie Mellon University, School of Computer Science, Pittsburgh, USA, ^4^University of Pittsburgh, School of Nursing, Pittsburgh, USA, ^5^University of Pittsburgh, Critical Care Medicine, Pittsburgh, USA

*Critical Care* 2023, **27(S1)**: P200

**Introduction:** Unanticipated physiologic instabilities from hemorrhage are dangerous. With a simulated human bleeding model on whole blood donor volunteers, we sought to identify early signatures of bleeding using a supervised machine learning model.

**Methods:** 51 adult human subjects donated whole blood (mean age 49 ± 18 years; 55% male). On average, subjects pre-bleed hemoglobin was 14.3 g/dl, and 495 ml of blood was donated during 7 min 3 s. Each individual was instructed to stand and sit before and after donation to obtain orthostatic blood pressures. Noninvasive vital signs including electrocardiogram (250 Hz) and photoplethysmography (125 Hz) were obtained from a portable MP50 monitor, and non-invasive arterial waveform (100 Hz) with cardiac indices were acquired from a LiDCOrapid device. During donation, blood volume loss was documented each minute. Data were curated and 20 s moving time windows were extracted every 10 s. From these time windows, statistical, time-series, and morphologic features were computed. A random forest classifier was trained to identify bleeding events using these features, which were normalized by their non-bleeding baseline period before any orthostatic changes.

**Results:** Our model, evaluated using a leave-one-patient-out cross validation scheme, demonstrated high performance in detecting bleeding using only non-invasive vital sign features: the mean AUROC was 0.955, and the TPR and TNR at clinically relevant low error regions (1% FPR & 1% FNR) were 55% and 46% (Fig. 1). Bleeding was then subclassified across the bleeding epoch progression as early (≤ 100 ml), mid (100–250 ml), and late (> 250 ml) and analyzed. Truncated ROC curves in Fig. 1 show that the models were able to detect even mild bleeding, showing the robustness of the model in very subtle physiologic changes in bleeding human.

**Conclusions:** Using a random forest model, physiologic evidence of bleeding could be identified at a very early course of bleeding in human subjects.

**Figure Figch:**
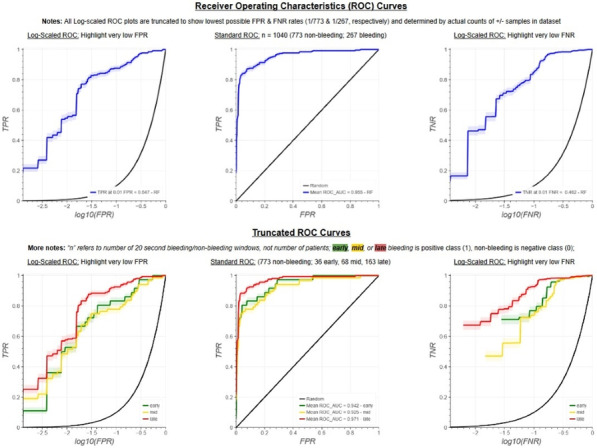
**Fig. 1 (abstract P200)**. Truncated receiver operating characteristic curves to detect bleeding events

## P201

### An experimental study via novel dynamic lower body negative pressure model to analyse simulated hemorrhage using arterial blood pressure

#### N Nesaragi^1^, LØ Høiseth^2^, HA Qadir^3^, LA Rosseland^4^, PS Halvorsen^5^, I Balasingham^6^

##### ^1^Oslo University Hospital, The Intervention Centre, Oslo, Norway, ^2^University of Oslo, Division of Emergencies and Critical Care, Department of Anesthesiology, Oslo, Norway, ^3^Oslo University Hospital, Oslo, Norway, ^4^University of Oslo, Oslo, Norway, ^5^University of Oslo, Institute of Clinical Medicine, Faculty of Medicine, Oslo, Norway, ^6^Norwegian University of Science and Technology, Department of Electronic Systems, Trondheim, Norway

*Critical Care* 2023, **27(S1)**: P201

**Introduction:** Bleeding is a life-threatening complication in trauma and surgery. Early detection of bleeding is mandatory to improve outcomes but is difficult due to physiological responses and unreliable vital signs monitoring. We explored the discriminative ability of a novel deep learning (DL) framework on arterial waveforms to classify levels of ongoing hypovolemia in a new dynamic version of the lower body negative pressure (LBNP) model.

**Methods:** Healthy subjects (n = 23) with invasive radial arterial pressure had three runs of LBNP (69 trials). The LBNP levels (range 0 to −60 mmHg) in each run were randomized in order to; 1) circumvent the time dependency of measurements, and 2) mimic both bleeding and fluid resuscitation (dynamic model). A supervised DL-based framework for ternary classification was realized by segmenting the underlying invasive signal and labeling segments with corresponding LBNP target levels. The DL framework had two inputs that were trained with respective time–frequency representations extracted on waveform segments to classify each of them into blood volume loss: Class 1 (mild); Class 2 (moderate); or Class 3 (severe). At the outset, the latent space derived from the unified DL model via late fusion among both inputs assisted in enhancing classification performance.

**Results:** The experimental study was performed using threefold cross-validation based on a patient-wise stratification scheme. i.e., within each fold, the model is trained and developed using a unique 70% (16 subjects) of total data, and the remaining 30% (7 subjects) data is considered for validation. In a threefold cross-validation setup with stratified subjects, the experimental findings demonstrated AUROC: 0.84, AUPRC: 0.73, and F1-score: 69.12% (Fig. 1).

**Conclusions:** Our proposed DL algorithm on arterial waveforms enabled to capture accurately the complex interplay in physiological responses related to both bleeding and fluid resuscitation.

**Figure Figci:**
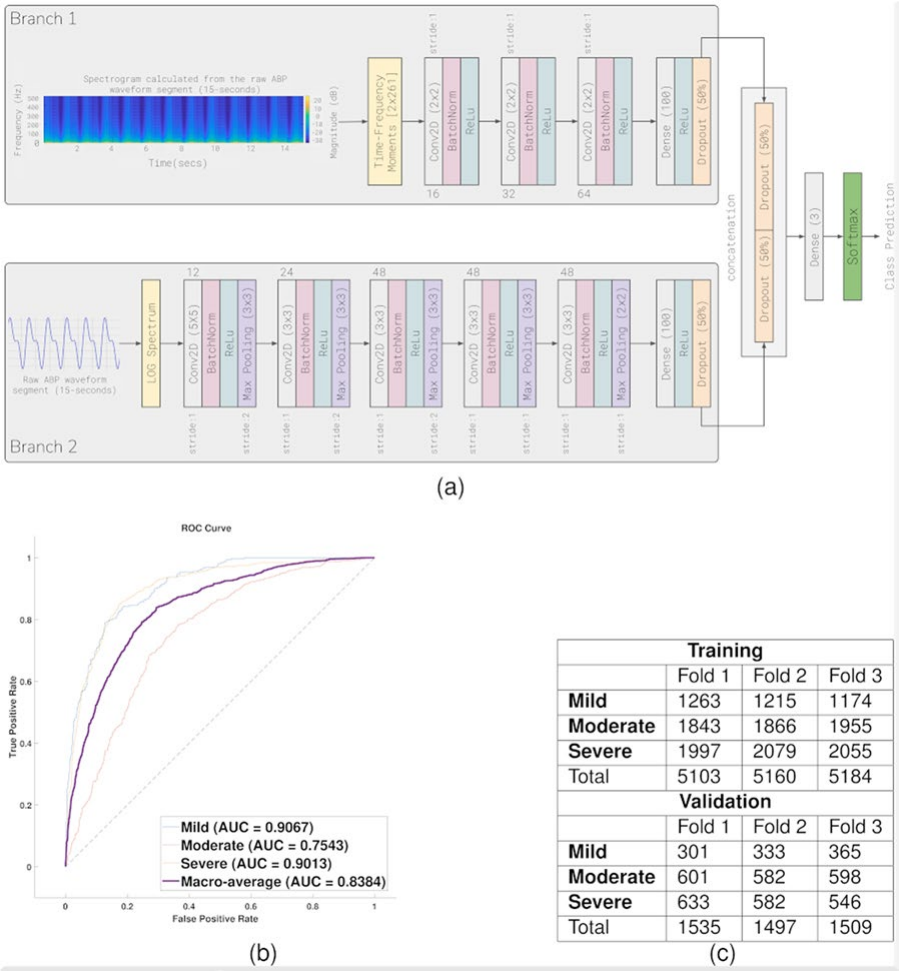
**Fig. 1 (abstract P201)**. A supervised deep learning (DL)-based framework for ternary classification of the entire dynamic LBNP trajectory depicting simulated hemorrhage is realized by segmenting the ABP and labeling segments with corresponding LBNP target levels. **a** The proposed DL model is trained with time frequency representations extracted on waveform segments to classify each of them into blood volume loss: Class 1 (mild); Class 2 (moderate); or Class 3 (severe). **b** Average area under receiver-operating characteristic curves (AUC) displaying the ability of the proposed unified model to perform the desired classification in a threefold cross-validation setup. **c** Sample distribution of waveform segments for model training and validation in threefold cross-validation

## P202

### Continuous quantitative analysis of urine in ICU patients

#### N Postma^1^, S Struiken^1^, L De Jager^1^, M Onrust^1^, J Kootstra ^2^, C Cordeiro^3^, M Nijsten^4^

##### ^1^UMC Groningen, Dpt of Critical Care, Groningen, Netherlands, ^2^UMC Groningen, Dpt of Clinical Chemistry, Groningen, Netherlands, ^3^Health Hub, Paramedir, Roden, Netherlands, ^4^UMC Groningen, ICU, Groningen, Netherlands

*Critical Care* 2023, **27(S1)**: P202

**Introduction:** With advanced sensor technology, it is in principle feasible to perform continuous monitoring of urinary electrolytes and metabolites in ICU patients with a urinary catheter. An open question is whether frequent urinary measurements in ICU patients will detect relevant changes. Thus we examined if semi-continuous quantitative monitoring of urine might be useful in the ICU.

**Methods:** Patients with a range of indications for ICU-admission and different length of stays in the ICU were included. For each patient, 20 urine samples were collected every 15 min for 5 h. In addition to urinary volume, samples were analyzed at the clinical chemistry laboratory for 10 parameters: diuresis, creatinine, urea, glucose, natrium, chloride, kalium, phosphate, total protein and albumin as well as the anion gap. Data were analyzed in R studio and examined for bivariate correlations, specific patterns and rapid changes of these parameters. For each patient and parameter, the ratio between the median value and highest/lowest value was determined. The frequency distributions of these ratios are shown to illustrate the dynamics.

**Results:** In 209 patients 3892 samples were analyzed. Remarkably rapid and pronounced changes were seen in all parameters (Fig. 1). The independent behavior of the studied parameters was underscored by limited bivariate correlations that ranged from *R* = −0.19 (creatinine and sodium) to *R* =  + 0.84 (albumin and total protein). Sudden deviations of certain parameters could be frequently associated with documented events.

**Conclusions:** The concentrations of the studied parameters could change rapidly and independently within the 5 h observation period. High time-resolution monitoring of urine electrolytes and metabolites may provide a novel near real-time and non-invasive window on the complex pathophysiology of ICU patients.

**Figure Figcj:**
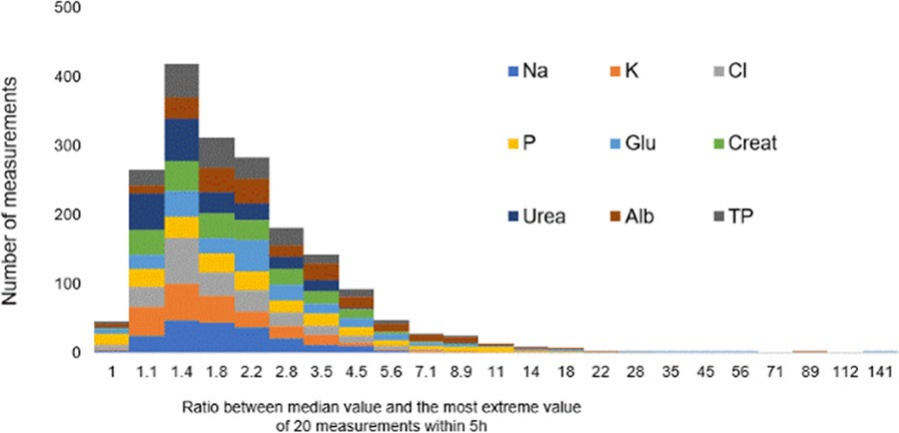
**Fig. 1 (abstract P202)**. Ratio between most extreme value and median value within 8 h study period. Na: sodium; K: potassium; Cl: chloride; P: phosphate; glu: glucose; TP: total protein

## P203

### Bioimpedance analysis in major surgeries

#### E Kyosebekirov, I Minev, CH Stefanov, D Kazakov, E Mitkovski, S Nikolova, G Pavlov

##### Medical University, Department of Anaesthesiology and Intensive Care Medicine, Plovdiv, Bulgaria

*Critical Care* 2023, **27(S1)**: P203

**Introduction:** The aim of this study is to assess the feasibility of bioimpedance analysis (BIA) in the assessment of fluid status and the effect of fluid therapy in major surgeries.

**Methods:** Observational, prospective study conducted in the period April 2021-September 2022 amongst patients hospitalized for elective major surgical interventions in University hospital St. George-Plovdiv, Bulgaria. BIA measurements were performed 1 h before and after general anesthesia. Intraoperatively standard monitoring was applied. Perspiratio insensibilis were accepted as 0.5 ml/kg/h and net balance was calculated. BodyStat Multiscan 5000 and manufacturer’s electrodes were used. Overhydration (OHY), total body water (TBW), extracellular water (ECW), and intracellular water (ICW) were the analyzed parameters.

**Results:** 40 patients aged 59.15 ± 9.68 years with BMI 27.1 ± 4.3 kg/cm^2^ and duration of the procedure 189.75 ± 87.63 min were involved in the study. There were 18 laparotomies, 15, thoracotomies and 7 craniotomies. Calculated net balance was 1344.64 ± 533.02 ml. TBW, ECW, ICW and OHY increased from the respective 44.84 ± 10.38 l, 15.08 ± 3.35 l, 29.66 ± 7.29 l, -0.77 ± 0.84 to 46.38 ± 10.57 l, 16.62 ± 3.52 l, 29.76 ± 7.3 l, 1.28 ± 0.75. There was a correlation between net balance and the increase in TBW (r = 0.41, *p* < 0.05), ECW (r = 0.47, *p* < 0.05) and OHY(r = 0.33, *p* < 0.05), whereas ICW increase was insignificant (*p* = 0.24).

**Conclusions:** BIA gives promising results in the evaluation of TBW and body fluid distribution in the perioperative period and could be a useful tool in the complex fluid therapy management. Although relatively old, the method is gaining increasing popularity and wider application amongst patients.

## P204

### Fluid balance association with mechanical ventilation time in cardiopulmonary bypass

#### CD Del Angel Argueta^1^, OI Aguilera Olvera^2^, GA Aguirre Gomez^2^, GL Arcos Lopez^2^, GL Velazquez Estrada^2^, JC Muñoz Chaves^2^, JA Villalobos Silva^2^

##### ^1^Hospital Regional De Alta Especialidad, Anestesiology, Ciudad Victoria, Mexico, ^2^Hospital Regional De Alta Especialidad, Critical Care, Ciudad Victoria, Mexico

*Critical Care* 2023, **27(S1)**: P204

**Introduction:** In patients undergoing cardiac surgery, positive fluid balances are common and are associated with longer mechanical ventilation, renal failure, and mortality, causing tissue edema, organ dysfunction, and coagulopathy, it is associated with an increased risk of post-operative complications [1, 2].

**Methods:** We performed a retrospective cohort study to analyze the association between positive fluid balances during the perioperative stage of cardiopulmonary bypass surgery and mechanical ventilation duration in a high specialty hospital of northeast Mexico during 2016–2022, we included patient records and collected data demographics, clinical variables. We use percentages, means and standard deviation accordingly, p value (< 0.05), odds ratio (CI 95%) for mechanical ventilation time (< 12 h), AKI, LOS ICU, delta lactate and urgent reintervention.

**Results:** We analyzed 60 patient records who were intervened in the Regional High Specialty Hospital, 66.7% were women, the mean age 53 years (SD 14.1), BMI mean 27.3 kg/m^2^ (SD 4.7), NYHA class III in 48.3%, 50% was coronary heart disease and 30% for valvular heart disease, EuroScore II mean 1.56% (SD 2.16), with preoperative creatinine of 0.8 mg/dl (SD 0.3 mg/dl), during the procedure the lactate maximum value 4.13 (SD 2.1 mmol/l), odds ratio (OR) for mechanical ventilation less than 12 h favoring positive fluid balance less than 2000 ml was 2.73 (CI 95% 0.35–21.17, *p* = 0.32) (Fig. 1), for AKI OR 0.23 (CI 95% 0.06–0.84, *p* = 0.21), ICU LOS OR 1.40 (CI 95% 0.42–4.66, *p* = 0.57), delta lactate 4 mmol/L OR 3.62 (CI 95% 0.72–18.06, *p* = 0.10) and urgent reintervention OR 0.78 (CI 95% 0.66–9.21, *p* = 0.84).

**Conclusions:** In our study we found that there is no association between fluid balance and the duration of mechanical ventilation, instead a correlation was observed to present acute kidney injury, however more studies with a larger sample are needed for conclusions.


**References**
Romagnoli S et al. J Cardiothorac Vasc Anesth 2016;30:1076–84.Koc V et al. Crit Care Res Pract. 2020;2020:4,836,862.

**Figure Figck:**
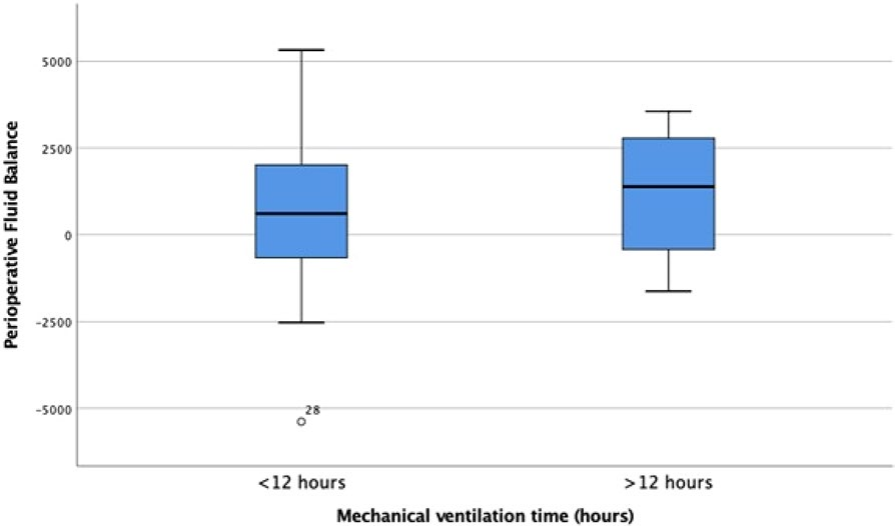
**Fig. 1 (abstract P204)**. Boxplot of perioperative fluid balance in mechanical ventilation time

## P205

### Correlation between the initial fluid balance of patients with preeclampsia with severity data and its complications in the intensive care unit

#### JA Garza Carrión, C Zarazúa Sosa, OI Aguilera Olvera, A Zárate Gracia, JA Villalobos Silva

##### General Hospital "Dr. Norberto Treviño Zapata", Adult Intensive Care Unite, Ciudad Victoria, Mexico

*Critical Care* 2023, **27(S1)**: P205

**Introduction:** Preeclampsia is one of the most frequent diagnoses for admission to the ICU. We will analyze the relationship between the initial fluid balance and the complications expressed in patients admitted to the ICU of an hospital in the Northeast of Mexico from January 2020 to October 2022.

**Methods:** In a retrospective cohort study we analized the association between fluid balance at 24, 48 and 72 h from ICU admission at an hospital in northeast of Mexico, included obstetric patients with complications such as preeclampsia-eclampsia, HELLP syndrome, fluid overload, gestational hypertension. We analyzed categorical variables with percentages, numerical variables with mean and standard deviation and obtained odds ratio for complication events.

**Results:** We analyzed data from 66 patients admitted to ICU during 2020–2022, mean age group were 21–29 years old (48.5%), with obstetric history of 2–3 pregnancies (53%), comorbidities such as previous hypertension and DM2 were 21.2 and 9.1%, most affected organ were kidney, brain and liver (63.6%), complications like pregnancy arterial hypertension (42.4%), eclampsia (9.1%), fluid overload (7.6%), HELLP syndrome (1.5%) and liver rupture (1.5%) were registered. Fluid balance at 24 h admission was 1148.95 ml mean value (sd 819.524 ml), 48 h -187.16 ml (sd 1097.167 ml), 72 h -1537 ml (sd 1302.491). Association for 24 h fluid balance and eclampsia was OR 0.82 (95% CI 0.72–0.934), fluid overload OR 8.438 (95% CI 1.028–69.247), arterial hypertension OR 0.367 (95% CI 0.074–1.829), liver rupture OR 1.067 (95% CI 0.94–1.211), HELLP syndrome OR 0.951 (95% CI 0224–4.047).

**Conclusions:** In this study we found association between positive fluid balances at 24 and 48 h in obstetric patients for fluid overload and for eclampsia at 72 h (Fig. 1). First 24 h there is a tendency for more positive fluid balances that gradually becomes negative when ICU arrival.

**Figure Figcl:**
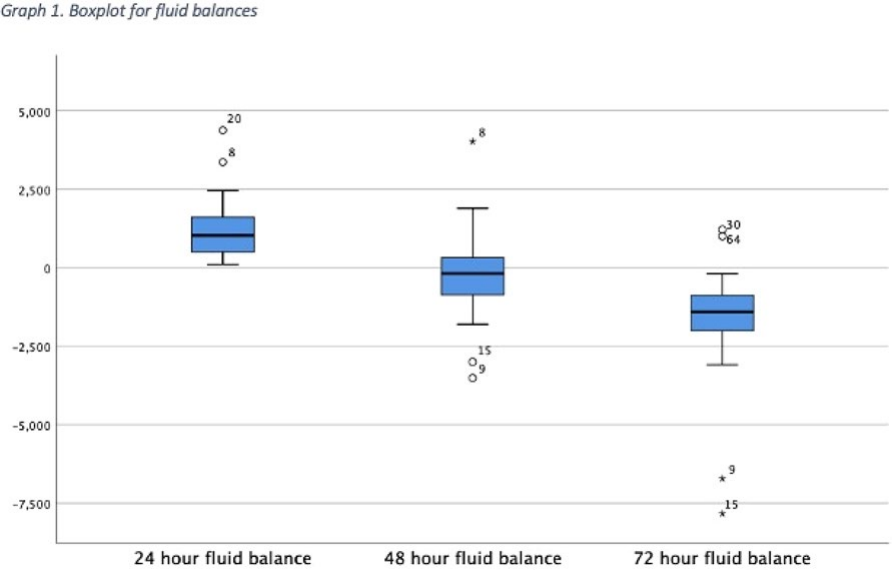
**Fig. 1 (abstract P205)**. Comparison between fluid balance at 24 h, 48 h and 72 h of patients with severe preeclampsia in ICU

## P206

### The outcomes of PICC insertion in pediatric patients at tertiary-care, university hospital

#### N Wongchompoo

##### Siriraj Hospital, Department of Anesthesiology, Bangkok, Thailand

*Critical Care* 2023, **27(S1)**: P206

**Introduction:** Peripherally-inserted central catheter (PICC) is widely used for intermediate- and long-term venous access. Venipuncture and catheterization in pediatric patients were challenging. Children's vein may become damaged by frequent and painful needle insertions. In Thailand, PICC was not yet prevailing even in adult patients and limited of the published works in pediatric patients. This study aims to demonstrate the outcome of PICC insertion in pediatric patients by the Anesthesia Line Service Team (ALiST) of a tertiary-care, university hospital in Thailand.

**Methods:** This is a retrospective, descriptive study between January 2018 and December 2021. The inclusion criteria were pediatric patients (age < 15 years), body weight more than 5 kg and no history of vein abnormality. The primary outcome is the success rate of insertion. The characteristic of patients, catheter, reason of removal and complications were also reported.

**Results:** A total of 1850 PICCs were inserted during the study period and 149 PICC were inserted in pediatric patients. There were 63 boys and girls equally. The median age of patients was 5.47 years (ranging from 3 months to 15 years). The median height was 106.06 cm, while median weight was 20.10. The successful insertion rate was 99.21%. All of them were inserted under ultrasound-guided, with or without real-time fluoroscopy. No complications during insertion were noted. The average usage days were 66.48 days (4–402). Four Fr, single lumen catheter was the most common PICC used (38.1%), followed by 3Fr, single lumen (32.5%) and 5Fr, double lumen (29.4%). Reason for removal of PICC lines were completion of therapy (50.86%), catheter malfunction (25.86%), infection (6.9%), and accidental removal (2.59%).

**Conclusions:** This is the first study about PICC lines insertion in pediatric patient in university hospital of Thailand. Our study showed the successes rate of 99.41%. PICC in pediatric patients are safe and low complication.

## P207

### Intracavitary electrocardiogram guided positioning of central venous catheter in critically ill patients

#### LC Ciatti, CB Biuzzi, SF Finetto, SS Scolletta

##### Azienda ospedaliera universitaria Senese, Dipartimento Anestesia e Rianimazione, Siena, Italy

*Critical Care* 2023, **27(S1)**: P207

**Introduction:** Our research group conducted this study with a primary objective to determine if the ECG guided technique (ECGgt) could reduce the number of post-procedural chest x-rays (CXR). In critically ill patients central venous catheter (CVC) placement is a commonly performed procedure with potential serious complications [1]. Various techniques to ascertain CVC tip placement (CVCtp) include anatomical landmark-guided methods, CXR and right atrium (RA) ECG.

**Methods:** All 128 patients enrolled in the study were hospitalized in Siena ICU, ‘Anestesia e Rianimazione’ department. Data were collected from 1° January 2022 to 31° June 2022 and retrospectively compared to data of 2021. The ECGgt included the metallic wire technique (CVC Braun Certofix + ECG adapter kit) and the fluid column technique (ECG VygoCard Kit). With ECGgt an accurate analysis of the P-shape at the ECG monitor was required. By monitoring the P-wave, we were able to determine CVCtp as it travelled through SVC-RA and RA.

**Results:** Out of the 128 patients 106 patients underwent positioning of a CVC with ECGgt (82.8%), for 22 the procedure was contraindicated. Of the 106 CVC, 80 were PICC, 26 were CICC. Intra-procedural complication rate was 1.8%. 80 patients received CXR after ECGgt and correct positioning was confirmed in 100% of cases. Data of 2022 showed that ECGgt had a 49% increase (34,1% in 2021 and 84% in 2022). Conversely a decrease in CXR was shown (Fig. 1).

**Conclusions:** Our study shows that ECGgt is a safe, reliable technique and a potential valid alternative to CXR. The advantages of ECGgt are the high feasibility, the catheter being operable just after the procedure, the optimisation of resources in the ICU and patients/health care staff x-rays exposure being avoided.


**Reference**
Soldati G. et al. Chest 2008;133;204–21.

**Figure Figcm:**
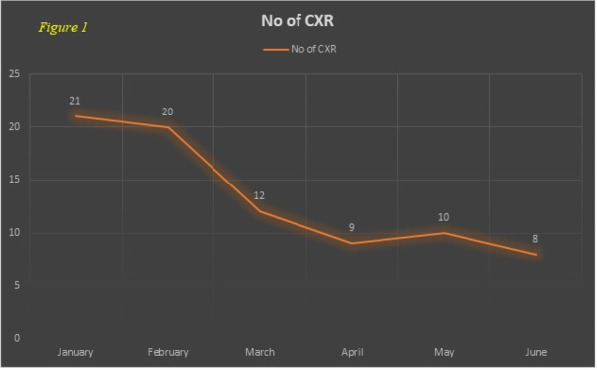
**Fig. 1 (abstract P207)**. Use of CXR

## P208

### The impact of central venous catheter (CVC) insertion on intravenous fluid burden in high-risk colorectal surgery: an observational study

#### B Milne^1^, P Beddoes^1^, A Davidson^1^, H Carter^1^, D Melia^2^

##### ^1^Whipps Cross University Hospital, London, UK, ^2^Whipps Cross University Hospital, Dept of Anaesthesia and Critical Care, London, UK

*Critical Care* 2023, **27(S1)**: P208

**Introduction:** Perioperative fluid strategy remains controversial [1]. We sought to address whether placement of a CVC influences this. There have been conflicting results from trials of goal-directed therapy [2–3], but avoidance of aggressive fluid loading appears to be associated with better outcome [4].

**Methods:** The study was granted Health Research Authority approval (London Central). All adult patients presenting to our institution for elective major colorectal resection, with planned postoperative critical care, were included between May 2019 and March 2020. Primary outcome measure was the difference in mean fluid volume infused between the two groups (‘CVC Inserted’ and ‘No CVC’). Student's T-Test was used to compare means between the two groups. Where this was not appropriate, differences in median values were calculated using non-parametric methods (i.e. Mann Whitney U Test).

**Results:** 40 patients were recruited (17 'CVC Inserted' and 23 ‘No CVC’). There were significantly lower median volumes of both crystalloid and total intravenous fluid volume (1800 ml vs. 3000 ml; *p* value < 0.0001, 95% CI 750–1950 ml and 750–1800 ml respectively) infused in the ‘CVC Inserted’ group. Postoperative fluid volumes demonstrated a trend for reduced fluid volume in the ‘CVC Inserted’ group, however this did not reach statistical significance for either total crystalloid (460 ml vs. 980 ml; p value = 0.0633, 95% CI 0–940 ml) or weight and time adjusted fluid volume (0.012 ml/kg/min vs. 0.015 ml/kg/min; p value = 0.0880, 95% CI − 0.001 to 0.015 ml/kg/min).

**Conclusions:** An average of 40% less fluids were given to the ‘CVC inserted group’ in our study across the perioperative period. The potential reasons for this are numerous and further research is required to investigate this further.


**References**
Miller TE et al. Anesthesiology 2019;130:825–832.Pearse RM et al. JAMA 2014;311:2181‐2190.Lewis SR et al. Cochrane Database of Systematic Reviews 2016;3:CD003004.Lobo DN et al. Lancet 2002;359:1812‐1818.


## P209

### Effects of fluid bolus on left ventricular myocardial performance index and global longitudinal strain in critically ill patients: a pilot study

#### C Pierrakos^1^, K Kaefer^1^, S Anane^1^, A Gallerani^1^, L Barreto Gutierrez^1^, P Djimafo^1^, S Redant^1^, D Velissaris^2^, R Attou^1^

##### ^1^CHU Brugmann, ICU, Laeken, Belgium, ^2^University Hospital of Patras, Internal Medicine Department, Patras, Greece

*Critical Care* 2023, **27(S1)**: P209

**Introduction:** Left ventricular myocardial performance index (LV − MPI) may be an attractive method for monitoring changes in LV systolic and diastolic function during fluid bolus (FB). However, it is not clear whether FB induced changes in LV preload can affect LV − MPI calculation. In this study, we evaluated changes in LV − MPI during FB.

**Methods:** This is a post-hoc analysis of a prospective observational study that evaluated patients who received a comprehensive transthoracic echocardiogram examination before and after FB. All patients received 4 mL/kg crystalloids at a rate of 1000 mL/h as local standard care. Using the tissue Doppler technique, LV − MPI was calculated as the ratio between the sum of the iso-volumetric contraction and relaxation time to the ejection time. Patients were considered FB responders when an increase in cardiac index (CI) of > 10% was observed.

**Results:** 24 patients were included in the analysis. No significant correlation was observed between the changes in central venous pressure (CVP) during FB and LV − MPI (r = 0.12, *p* = 0.67). Thirteen patients did not have any significant increase in CI after FB (2.4 ml/min/m^2^ to 2.3 ml/min/m^2^, *P* = 0.83) despite a significant increase in CVP (7.5 [2.5–12] mmHg to 10 [8.1–15.2] mmHg, *P* = 0.04). In these patients, no significant change in LV − MPI was observed (0.47 [0.43–0.61] to 0.48 [0.41–0.68], *p* = 0.68) (Fig. 1). In 11 patients, FB increased CI (1.86 [1.41–2.77] ml/min/m^2^ to 2.27 ml/min/m^2^ [1.82–3.33], *p* < 0.01) without statistically significant changes in central venous pressure (3.1 [1.0–8.3] mmHg to 4.0 [2.0–9.5], *p* = 0.44). In these patients, a trend for decreasing LV − MPI was observed (0.47 [0.36–0.48] to 0.34 [0.28–0.41], *p* = 0.17) (Fig. 1).

**Conclusions:** Fluid bolus (FB) induced acute preload changes which did not affect LV − MPI. Decreases in LV − MPI observed during FB may be associated with heart function improvement.

**Figure Figcn:**
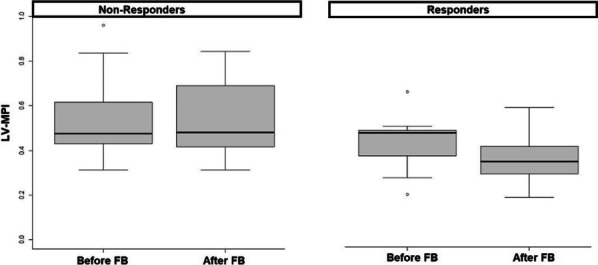
**Fig. 1 (abstract P209)**. Changes in left ventricular myocardial performance index (LV − MPI) in fluid bolus (FB) non-responders and responders

## P210

### Monitoring of fluid responsiveness using mitochondrial oxygenation after cardiac surgery

#### B Hilderink^1^, R F Crane^1^, B Van den Bogaard^1^, J Pillay^2^, N.P. Juffermans^3^

##### ^1^OLVG, Intensive Care, Amsterdam, Netherlands, ^2^University Medical Center Groningen, Intensive Care, Groningen, Netherlands, ^3^Erasmus University Medical Center, Laboratory of Intensive Care, Rotterdam, Netherlands

*Critical Care* 2023, **27(S1)**: P210

**Introduction:** Fluid responsiveness is conventionally defined at the level of the heart. The aim of volume expansion is to improve organ perfusion and oxygenation by increasing cardiac output. However, after cardiac surgery, there is marked dissociation between cardiac output and tissue oxygenation due to the deleterious effects of cardiopulmonary bypass on the microcirculation. We investigated the utility of mitochondrial oxygen tension (mitoPO_2_) to guide fluid therapy in patients after cardiac surgery by measurements of cardiac output in parallel with mitoPO_2_. MitoPO_2_ is a novel bed-side quantitative marker which measures the balance between oxygen delivery and consumption in the skin.

**Methods:** Patients undergoing cardiac surgery with cardiopulmonary bypass were included. Cardiac output was measured using non-invasive finger cuff pulse contour analysis (Nexfin, BMEYE ®, Amsterdam). Fluid responsiveness was defined as an increase in CO of > 10% after a fluid bolus of > 500 ml in 1 h. MitoPO_2_ was measured before and 15 min after the administration of a fluid bolus using the COMET device (Photonics healthcare, Utrecht, The Netherlands).

**Results:** 44 patients after cardiac surgery were included in whom 46 fluid boluses were investigated. Of these, 13 (28%) were fluid responders. In fluid responders, mitoPO2 increased by 7.6 (1.4–9.7) mmHg whereas in fluid non-responders, mitoPO_2_ decreased by 4.8 mmHg (*p* = 0.004). Mitochondrial oxygen consumption (mitoVO_2_) increased in fluid responders 0.52 (−0.15 to 1.5) mmHg/s compared to non-responders (*p* = 0.02). Mean arterial pressure, core-toe temperature gradient, lactate did not change when compared to fluid non-responders. The AUROC of mitoPO_2_ to monitor fluid responsiveness was 0.78 (*p* < 0.05).

**Conclusions:** Changes in mitoPO_2_ and mitoVO_2_ are associated with fluid responsiveness and can potentially be used to guide fluid resuscitation after cardiac surgery.

## P211

### The increase in cardiac output induced by a decrease in positive end-expiratory pressure reliably detects volume responsiveness: the PEEP-test study

#### C Lai^1^, R Shi^1^, A Beurton^2^, F Moretto^1^, N Fage^1^, S Ayed ^1^, M Dres^2^, JL Teboul^1^, X Monnet^1^

##### ^1^AP-HP, Service de Médecine Intensive-Réanimation, Hôpital de Bicêtre, DMU 4 CORREVE, Inserm UMR S_999, FHU SEPSIS, CARMAS, Université Paris-Saclay, Le Kremlin-Bicêtre, France, ^2^AP-HP, Groupe Hospitalier Universitaire APHP-Sorbonne Université, site Pitié-Salpêtrière, Service de Pneumologie, Médecine intensive Réanimation (Département R3S), Paris, France

*Critical Care* 2023, **27(S1)**: P211

**Introduction:** In patients on mechanical ventilation, positive end-expiratory pressure (PEEP) can decrease cardiac output through a decrease in cardiac preload and/or an increase in right ventricular afterload. We hypothesized that a transient decrease in PEEP (PEEP-test) may be used as a test to detect volume responsiveness.

**Methods:** Mechanically ventilated patients with PEEP ≥ 10 cmH_2_O (“high level”) and without spontaneous breathing were prospectively included. Volume responsiveness was assessed by a positive passive leg raising (PLR) test, defined as an increase in cardiac index (CI) during PLR ≥ 10%. The PEEP-test consisted in reducing PEEP from the high level to 5 cmH_2_O for one minute. Changes in CI were monitored with pulse-contour-derived CI (PiCCO2).

**Results:** We included 64 patients among whom 31 were volume responsive. The increase in CI during PLR was 14%(11–16%). The PEEP at baseline was 12(10–15) cmH_2_O and the PEEP-test resulted in a median decrease in PEEP of 7(5–10) cmH_2_O, without difference between volume responsive and unresponsive patients. Among volume responsive patients, the PEEP-test induced a significant increase in CI of 16%(12–20%) (from 2.4 ± 0.7 to 2.9 ± 0.9 L/min/m^2^, *p* < 0.001) compared to volume unresponsive patients. In volume unresponsive patients, PLR and the PEEP-test increased CI by 2%(1–5%) and 6%(3–8%). Volume responsiveness was predicted by an increase in CI > 8.6% during the PEEP-test with a sensitivity of 96.8%(95% confidence interval (95% CI) 83.3–99.9%) and a specificity of 84.9% (95% CI 68.1–94.9%). The area under the receiver operating characteristic curve of the PEEP-test for detecting volume responsiveness was 0.94 (95% CI 0.85–0.98) (*p* < 0.001 vs. 0.5). Spearman’s correlation coefficient between the changes in CI induced by PLR and the PEEP-test was 0.76 (95% CI 0.63–0.85, *p* < 0.001).

**Conclusions:** A CI increase > 8.6% during a PEEP-test, which consists in reducing PEEP to 5 cmH_2_O, reliably detects volume responsiveness in mechanically ventilated patients with a PEEP ≥ 10 cmH_2_O.

## P212

### Systolic volume variation and pulse pressure variation as fluid responsiveness predictors in patients in septic shock: assessment of limitations in a prospective study

#### T Petrucci^1^, A Costa^2^, D Silva^1^, G Nobre de Jesus^1^, J Ribeiro^1^

##### ^1^University Hospital Santa Maria, Intensive Care Department, Lisbon, Portugal, ^2^Faculdade de Medicina de Lisboa, Clínica Universitária de Medicina Intensiva, Lisboa, Portugal

*Critical Care* 2023, **27(S1)**: P212

**Introduction:** Fluid resuscitation is a key stone in septic shock (SC) treatment. Nonetheless, assessment of fluid responsiveness (FR) has many confounders. Systolic volume variation (SVV) and pulse pressure variation (PPV) are validated as surrogate markers for FR, but some conditions limit their application: low tidal volume (VT) (< 8 ml/kg), right heart (RH) disfunction, high intra-abdominal pressure (IAP), low compliance, dysrhythmia, and patient triggered respiratory cycles. We conducted a prospective pragmatic study with the aim of characterizing the prevalence of these limitations in a cohort of patients with SC.

**Methods:** Patients were included in the first 24 h after SC recognition, identified by hyperlactacidemia and vasopressor dependence associated with presumed or declared infection. Hemodynamic (HD) data was gathered whenever a FR assessment was performed. Demographic, clinical and laboratory data were also collected.

**Results:** Twenty-three patients were included (Table 1), of which 20 had a minimally invasive HD monitoring device. There were limitations in 76.2% of the patients: 9 had a VT < 8 ml/kg, 9 had RH disfunction, 3 had high IAP, 2 had low compliance, 6 had dysrhythmia and 10 patients had patient triggered respiratory cycles. About 56.2% of the patients only had one limitation, 25% had two limitations and 18.8% had three. ICU mortality was 45%. Table 1 summarizes other important data collected.

**Conclusions:** In this preliminary study, the use of SVV or PPV to assess fluid responsiveness in patients with septic shock had many limitations. Most were related to invasive mechanical ventilation or right heart dysfunction. Albeit SVV and PPV are validated variables to predict FR in patients in SC, their applicability is limited, and our data suggests they may be of little use in a large percentage of ICU population. Intensivists should be aware of these conditions when they choose minimally invasive HD monitoring to guide fluid therapy in SC.

**Table Tabba:** **Table 1 (abstract P212)**. Additional data

Characteristics	Mean value
Age (years)	66.3 ± 10.2
SAPS II	61.7 ± 15.6
SOFA at ICU admission	10.3 ± 6.6
Initial lactate (mmol/l)	4.7 ± 3.1

## P213

### Heart rate variability as a surrogate for fluid responsiveness in patients in septic shock

#### A Costa^1^, T Petrucci^2^, S Mendes Fernandes^1^, D Silva^2^, G Nobre de Jesus^3^, JM Ribeiro^2^

##### ^1^Faculdade de Medicina de Lisboa, Clínica Universitária de Medicina Intensiva, Lisboa, Portugal, ^2^University Hospital Santa Maria, Intensive Care Department, Lisbon, Portugal, ^3^Faculdade de Medicina de Lisboa, Intensive Care Department, Lisboa, Portugal

*Critical Care* 2023, **27(S1)**: P213

**Introduction:** Fluid resuscitation is paramount in septic shock (SC) treatment and should be performed when hemodynamic benefits likely outweigh the risks of fluid overload. Usually, benefits are based on fluid responsiveness (FR) assessment. Defining FR is essential to avoid iatrogenesis. We hypothesized that heart rate (HR) variation, evaluated before and after a fluid challenge, might be a useful tool to validate fluid responsiveness.

**Methods:** The study was performed in a large urban, university hospital. Patients were included in the first 24 h after SC recognition, identified by hyperlactacidemia and vasopressor dependence associated with presumed or declared infection. Whenever clinical judgement recommended, fluid challenge was performed (500 mL crystalloid infusion in 30 min), without other interventions. FR data was evaluated with echocardiography and considered to be present when there was a 10% increase in cardiac output (CO) or left ventricle outflow tract-velocity time integral (VTI). An average of three VTI and peak velocity measures was used. Demographic, clinical and laboratory data, and other hemodynamic variables were collected, including HR prior and after FR.

**Results:** Preliminary assessment of data was performed in 23 patients. Three of which were excluded due to poor acoustic TE window. ICU mortality was 45%. All patients were under noradrenaline (NA); 19 were under invasive mechanical ventilation, 2 with non-invasive ventilation and 2 were spontaneously breathing. Table 1 summarizes other important data collected. Using the aforementioned FR definition, we identified 12 responsive and 8 nonresponsive patients. Mean HR variation was -3% (± 9.6; −18 to 22%). In responsive patients, there was no correlation between ΔVTI% and ΔHR% (*p* = 0.235) nor between ΔVTI% and ΔHR% if initial HR > 100 (*p* = 0.114).

**Conclusions:** In this pragmatic study, preliminary data suggests that ΔHR does not correlate with ΔVTI and cannot predict fluid responsiveness.

**Table Tabbb:** **Table 1 (abstract P213)**. Additional data

Characteristics	Mean value
Age (years)	66.3 (± 10.2)
SAPS II	61.7 (± 15.6)
SOFA at admission	10.3 (± 6.6)
Noradrenaline	0.54 mcg/kg/min (± 0.76)
Initial lactate	4.73 mg/dl (± 28.6)
Initial HR	88.8 (± 22.2)
Final HR	85.2 (± 19.4)

## P214

### Mottling in septic shock: skin colour matters!

#### S Jog, L Vikram

##### Deenanath Mangeshkar Hospital and Research Center, Intensive Care Medicine, Pune, India

*Critical Care* 2023, **27(S1)**: P214

**Introduction:** Skin mottling as a peripheral perfusion marker in septic shock is correlated well with severity and outcome [1–3]. Skin mottling is well studied in Caucasian population and its validity as a clinical sign in dark skinned ethnic population is not known. Our aim was to study the mottling in septic shock in the Indian ethnic population who has different skin colour as compared to Caucasian population.

**Methods:** We conducted a prospective observational study of consecutive patients with skin colour category 21–34 on von Luschan scale or Fitzpatrick type IV and V who had septic shock needing high dose of norepinephrine ≥ 0.2 mcg/kg/min after fluid optimisation.The study was conducted in a mixed medical—surgical ICU over a span of 12 months. Two experts (a dermatologist and a cosmetic surgeon) independently classified the skin type and scored mottling in our patients. We recorded the demographics, hemodynamic variables, mottling score and observed for the incidence of mottling and its correlation with predictors of severity of septic shock.

**Results:** We included 108 patients. Mean age was 61.3 years. Mean SOFA score and APACHE II score at enrolment was 10.3 and 21.9 respectively. Overall mortality was 72%. Incidence of mottling was 22/86 (20.3%). Development of mottling was significantly associated with APACHE II score > 22, SOFA score > 10 and norepinephrine dose > 0.3 mcg/kg/min (Table 1). Occurrence of mottling irrespective of its grade was strongly associated with mortality 19/22 (86.3%) (*p* = 0.028).

**Conclusions:** Incidence of mottling in septic shock is much less in Indian ethnicity with brown skin colour as compared to Caucasian population. Occurrence of mottling in this setting is associated with very high mortality.


**References**
Ait-Oufella H et al. Intensive Care Med. 2011;37:801–7.Ferraris A et al. Indian J Crit Care Med. 2020;24:672–676.Dumas G et al. Crit Care. 2019;23:211.

**Table Tabbf:** **Table 1 (abstract P214)**. Variables associated with development of mottling

Mottling	Yes (n = 22) Mean ± sd	No (n = 86) Mean = /−sd	t	*p* value
Age	58.5 ± 15.41	62.06 ± 15.68	− 0.953	0.343
MAP	65.96 ± 11.01	66.76 ± 10.07	− 0.325	0.746
SOFA score	11.59 ± 3.13	9.93 ± 2.94	2.333	0.022
APACHE II score	25.05 ± 7.07	21.15 ± 7.1	2.297	0.024
Norepinephrine dose	0.37 ± 0.12	0.29 ± 0.12	2.934	0.004
Duration of vasopressor support	6.32 ± 6.07	6.13 ± 4.26	0.17	0.865

## P215

### Assessing diuresis response in stable cardiogenic shock

#### SA Narra, H Batool, S Isha, S Jonna, K Singh, A Jena, D Sanghavi, A Bhattacharyya

##### Mayo Clinic, Critical Care Medicine, Jacksonville, USA

*Critical Care* 2023, **27(S1)**: P215

**Introduction:** Optimizing preload via diuresis and dialysis is one of the prime modalities for the management of cardiogenic shock. There is little consensus in the ICU on how to monitor patients’ readiness for fluid removal beyond the initial decompensated phase of cardiogenic shock. Echocardiography-based tools help in assessing hemodynamic status. We look at various Echo and Lab parameters to see if fluid removal can be dictated by them.

**Methods:** A retrospective single-center observational study was performed on 69 patients diagnosed with cardiogenic shock and on mechanical circulatory support. We collected echocardiographic parameters, lab values, vital signs, medications, and outcomes for 24 h from the day that echocardiograms were performed. This data was analyzed to study the association with bad outcomes. A bad outcome was defined by—fluid administration after diuresis, or an increase in vasopressor requirements. We used R 4.2.2 for statistical analysis and chi-square test or Fischer’s exact test used to calculate p values.

**Results:** Most of the patients in our cohort were males (78.3%) and of white (58%) ethnicity. 13% of them were on IABP, and 87% were on Impella devices, with Impella 5.5 being the most common (68.3%). 80% of our total cohort survived the hospital stay. Our analysis of 242 serial observations revealed that patients in the bad outcome category had more negative fluid balance [(− 995 ± 2040 vs. − 96 ± 1760); *p* < 0.001], and a higher prevalence (28.2% vs. 18.6%, *p* = 0.011) of reduced IVC collapsibility (< 50%) than the better outcome group. Relevant results are demonstrated in Table 1.

**Conclusions:** Beyond initial fluid optimization, echo-guided determination of volume status with euvolemic states is associated with better outcomes. Aggressive attempts to obtain a negative fluid balance is associated with poor short-term outcomes. Further prospective trials are required to determine causality.

**Table Tabbg:** **Table 1 (abstract P215)**. Results

Variables	Bad outcome group; Mean ± SD/%	Good outcome group; Mean ± SD/%	*p* value
IVC collapsibility reduced (< 50%)	28.20%	18.60%	0.011
RA pressure, mmHg	11.90 ± 5.80	12.70 ± 5.19	0.456
RV pressure, mmHg	46.00 ± 12.50	46.80 ± 13.20	0.761
Cardiac index, l/min/m^2^	2.59 ± 1.11	2.19 ± 0.98	0.119
Input–output, mL	−995 ± 2040	−96 ± 1760	< 0.001
BUN/Cr ratio	23.30 ± 19.80	20.20 ± 10.80	0.117
Bicarbonate, mmol/l	25.40 ± 9.22	24.10 ± 3.85	0.124

Bad outcome grou*p* = fluid administration after diuresis, or an increase in vasopressor requirements

## P216

### Diastolic shock index in sepsis and septic shock: A predictive factor of mortality

#### W Bahria, F Hamdi, W Demni, B Fradj, S Hayouni, S Drira, M Bouraoui, N Nouira

##### Mongi Slim Academic Hospital, Emergency Department, La Marsa, Tunisia

*Critical Care* 2023, **27(S1)**: P216

**Introduction:** Hypotension results from an interaction between vasodilatation, myocardial dysfunction and blood flowdisturbance in patients with sepsis and septic shock. In this cases, the diastolic blood pressure (DBP) and theheart rate (HR) may be usefull to reflect the hypotension conditions. We aimed to evaluate The relationshipbetween the diastolic shock index (DSI) and mortality in the ED.

**Methods:** This observational prospective and analytical study was conducted over three years in an universal hospitalincluding patients admitted to the ED for sepsis and septic shock. DSI defined as a ratio of HR to DBP (HR/DBP)and SSI defined as a ratio of HR to SBP (HR/DBP) were calculated for all patients on admission. A cutt-off pointof SSI > 0.9 and DSI > 1.4 were used. The primary end-poind was the intra-hospital mortality. ROC curves were used to identify variables predicting mortality.

**Results:** Two hundred and four patients were included in our study. One hundred and thirty three patients (65%) were in sepsis and 71 patients (35%) were in septic shock. The mean age was 64 years old [17–94] with a sex ratio of 1.08. SSI > 0.9 and the DSI > 1.4 are significantly associated with mortality in the ED with respectively (*p* = 0.000; OR = 2.3; 95% CI [1.57–3.59]) and (*p* = 0.004; OR = 1.8; 95% CI [1.18–3.00]). The SSI and DSI area underthe curve for in-hospital mortality were respectively (*p* = 0.000; OR = 0.725; 95% CI [0.64–0.80]) and (*p* = 0.000; OR = 0.68; 95% CI [0.60–0.76]).

**Conclusions:** DSI and SSI calculated on admission to the ED might identify patients with sepsis or septic shock at high risk ofdeath. In this study, DSI didn’t show a significant superiority to SSI for predicting mortality. This funding deserves more research in multicentre studies to be useful.

## P217

### Predictive value of intra-hospital mortality of "age-shock index" and "age-modified shock index" in ST-segment elevation myocardial infarction patients (STEMI)

#### Y Walha^1^, D Hamdi^1^, S Drira^1^, F Lazez^1^, B Fradj^1^, M Boussen^1^, F Azaiez^2^, N Nouira^1^

##### ^1^Mongi Slim Academic Hospital, Emergency Department, La Mara, Tunisia, ^2^Mongi Slim Academic Hospital, Cardiology Department, La Mara, Tunisia

*Critical Care* 2023, **27(S1)**: P217

**Introduction:** Early recognition of patients at high risk of intra-hospital death, will allow better, faster and more rigorous therapeutic management. The association between A-SI (age-shock index) or A-mSI (age-modified shock index) and short-term mortality in STEMI patients has not yet been sufficiently examined. The aim of this study was to prove the predictive values of A-SI and A-mSI for intra-hospital mortality in patients with myocardial infarction.

**Methods:** Two hundred STEMI patients admitted to the emergency department between January 2022 and November 2022 were analyzed in a retrospective cohort study. A-SI and A-mSI were calculated for all patients, the primary outcome was intra-hospital mortality.

**Results:** During the study period, 200 patients with STEMI were admitted to the emergency room. The mean age was 59 ± 12 years. The sex ratio was 4.52. Only 20% of patients consulted within the hour, the median consultation time was 3 h with extremes of up to 48 h, the rates of thrombolysis and primary ATC were respectively 63.5% and 22.5%. For other patients, 13% consulted late with STEMI semi recent and two cases of myocardial infarction spontaneously reperfused were hospitalized in the cardiology intensive care unit awaiting angioplasty. Intra-hospital mortality was 8%. The predictive value of SI and mSI mortality were tested statistically but no significant results were found. However by testing A-SI and A-mSI, the following significant results were found: A-SI (*p* = 0.01, odds ratio (OR) = 12.29, area under the curve (ROC-AUC) = 0.68, *p* = 0.018, CI[0.53–0.83]); A-MSI (OR = 16.13, *p* = 0.03, ROC-AUC = 0.70, *p* = 0.009), CI[0.54–0.85]).

**Conclusions:** Both A-SI and A-mSI could predict in-hospital mortality of patients presenting to the emergency department with a myocardial infarction.

## P218

### The prognostic value of reverse shock index in patients with sepsis at the emergency department

#### W Demni, F Hamdi, W Bahria, Y Zouaghi, K Jemai, F Lazzez, Z Farhani, N Nouira

##### Mongi Slim Academic Hospital, Emergency Department, La Marsa, Tunisia

*Critical Care* 2023, **27(S1)**: P218

**Introduction:** The “reverse shock index” (rSI) defined by the ratio of systolic blood pressure (SBP) to heart rate, can be quickly calculated to identify the prognosis of trauma patients mainly. In this study, we sought to evaluate the relationship between the rSI and in-hospital mortality in patients with sepsis at the emergency department (ED).

**Methods:** This was a prospective and analytical study, conducted over 9 months (February-October 2022). We included all patients with sepsis aged over 18 years and admitted to the ED. RSI and quick sequential organ fail assessment (qSOFA) were calculated at admission. We have determined the predictive factors of in-hospital mortality in uni and multivariate analysis. The area under the ROC curve of the rSI was analyzed.

**Results:** We enrolled 185 patients. The average age was 64 ± 16 years. The Sex ratio was 1.17. In-hospital mortality was 31.4%. On admission: 33% of the patients had a GCS < 15, 41% had SBP ≤ 100 mmHg, and 44% had a breathing rate ≥ 22 cpm. Half of the patients studied had a qSOFA ≥ 2, 69% were in sepsis and 31% were in septic shock. Predictive factors of in-hospital mortality in univariate analysis were: GCS < 15 (*p* < 0.05;OR = 2.85;95% CI = [1.86–4.36]), SPB ≤ 100 mmHg (*p* < 0.05; OR = 2.59;95% CI = [1.66–4.04]), qSOFA ≥ 2 (*p* < 0.05;OR = 2.30; 95% CI = [1.40–3.77]), and rSI < 1 (*p* < 0.05;OR = 2.70; 95% CI = [1.75–4.16]). In multivariate analysis, predictive factors of in-hospital mortality were: GCS < 15 (*p* < 0.002;OR = 4.7;95% CI = [1.75–12]), qSOFA ≥ 2 (*p* < 0.01;OR = 3.38;95% CI = [1.69–6.74]), and rSI < 1 (*p* < 0.001;OR = 4.8;95% CI = [2.43–9.58]). ROC curve analysis showed that the area under the curve of rSI < 1 was 0.68 (*p* < 0.05;95% CI = [0.59–0.77]).

**Conclusions:** Our study shows that the rSI calculated early in the ED appears to be a predictive factor of in-hospital mortality in patients with sepsis. The usefulness of rSI in directing therapeutic procedures in the management of sepsis should be examined in future studies.

## P219

### Angiotensin II for the treatment of vasodilatory shock in patients with acute kidney injury

#### A Zarbock^1^, L Forni^2^, J Hästbacka^3^, E Korneva ^4^, S Herzig^4^, G Landoni^5^, P Pickkers^6^, R Bellomo^7^

##### ^1^University Hospital Münster, Department of Anesthesiology and Critical Care, Münster, Germany, ^2^Royal Surrey Hospital & Faculty of Health Sciences, University of Surrey, Department of Critical Care, Guildford, UK, ^3^Helsinki University Hospital and University of Helsinki, Department of Perioperative, Intensive Care and Pain Medicine, Helsinki, Finland, ^4^Paion AG, Development and Regulatory Affairs, Aachen, Germany, ^5^IRCCS San Raffaele Hospital and University, Department of Anesthesia and Intensive Care, Milan, Italy, ^6^Radboud University Medical Center, Department of Intensive Care Medicine, Nijmegen, Netherlands, ^7^Intensive Care Medicine, The University of Melbourne, Melbourne, Australia

*Critical Care* 2023, **27(S1)**: P219

**Introduction:** Angiotensin II may improve patient outcomes in patients with vasodilatory shock associated with acute kidney injury (AKI) [1]. In preparation for a large phase 4 randomised controlled trial we aimed to identify an optimal target population for this vasopressor.

**Methods:** Post-hoc analysis of the data from the Phase 3 Angiotensin II for the Treatment of High-Output Shock trial was performed in a sub-population of patients with AKI to evaluate the effect of angiotensin II on mortality and renal-replacement therapy (RRT) utilisation. AKI was defined using the Kidney Disease Improving Global Outcomes (KDIGO) criteria. For comparisons between treatment arms, Fisher’s exact test or the chi-square test or the two-sample Wilcoxon rank-sum test was applied.

**Results:** From a total of 321 patients with distributive shock, 203 patients (63%) had AKI (stage 1: 25 patients (12.3%), stage 2: 42 patients (20.7%), stage 3: 136 patients (67%)). Among AKI patients 98 received angiotensin II and 105 received placebo. For combined AKI stages 2 and 3, the 28-day mortality rate was 51% (95% CI 40–62%) with angiotensin II vs 65% (95% CI 54–75%) with placebo (*p* = 0.068); this outcome became significant in patients with AKI stage 3 (48% (95% CI 35–61%) with angiotensin II vs. 67% (95% CI 55–78%) with placebo (*p* = 0.025)). No statistically significant difference was observed for the patients with AKI stage 1 or 2 or AKI overall. In the subgroups of the patients with AKI stage 3, stages 2 and 3, and AKI overall, the mean number of RRT free days up to Day 7 was significantly higher in the angiotensin II group; the same trend was observed for RRT duration (Fig. 1).

**Conclusions:** In the trial the majority of the patients had AKI at baseline. Angiotensin II treatment resulted in decreased RRT utilization in patients with AKI stages 2 and 3 and in reduced mortality in patients with AKI stage 3. These results introduce a target population for future trials.


**Reference**
Tumlin J et al. Crit Care Med 2018;46:949–957.


**Figure Figco:**
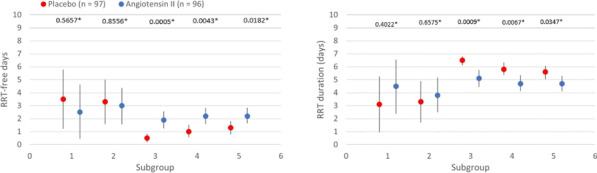
**Fig. 1 (abstract P219)**. Mean number of RRT-free days and RRT-duration (days) up to Day 7 with 95% confidence intervals. Patients who died up to Day 7 were counted as patient on RRT for 7 days. Subgroup 1—AKI, stage 1 at baseline; 2—AKI, stage 2 at baseline; 3—AKI, stage 3 at baseline; 4—AKI stage 2 and 3 at baseline; 5—AKI overall at baseline. **p* value

## P220

### Rates and complications of peripheral vasopressor use in the CLOVERS trial

#### ES Munroe^1^, IN Co^2^, IS Douglas^3^, RC Hyzy^1^, K Nelson^1^, PK Park^4^, TW Rice^5^, S Seelye^1^, NI Shapiro^6^, HC Prescott^1^

##### ^1^University of Michigan, Department of Internal Medicine, Ann Arbor, USA, ^2^University of Michigan, Department of Emergency Medicine, Ann Arbor, USA, ^3^Denver Health Medical Center and University of Colorado, Pulmonary Science and Critical Care Medicine, Denver, USA, ^4^University of Michigan, Department of Surgery, Ann Arbor, USA, ^5^Vanderbilt University, Division of Allergy, Pulmonary, and Critical Care Medicine, Nashville, USA, ^6^Beth Israel Deaconess Medical Center, Department of Emergency Medicine, Boston, USA

*Critical Care* 2023, **27(S1)**: P220

**Introduction:** Vasopressors are traditionally administered via central venous catheters (CVCs) given concern for extravasation and resultant tissue injury with peripheral administration. However, vasopressor infusion via peripheral IV may be safe and obviate the need for CVC placement in some patients. Little is known about how peripheral vasopressors are used in practice or how safety data from existing small, single-center studies extrapolate to routine care.

**Methods:** We conducted a post hoc analysis of peripheral vasopressor use in the Crystalloid Liberal or Vasopressors Early Resuscitation in Sepsis (CLOVERS) trial, which enrolled 1563 patients with early sepsis-induced hypotension in 60 US hospitals (3/2018–2/2022). Trial protocol allowed vasopressor administration through central access or a large peripheral IV, per discretion of the treating team, with vasopressor monitoring guided by local hospital protocols. We analyzed peripheral vasopressor use and assessed complications among patients treated with peripheral vasopressors and with CVC placement. Complications were graded on a 5-point scale from no clinical effect (1) to death (5).

**Results:** Of 1563 patients enrolled in CLOVERS, 750 (48.0%) received vasopressors, including 500 (32.0%) who received vasopressors peripherally. 363 patients (23.2%) had a CVC placed within 72 h of study enrollment, including 234 (64.5%) who received peripheral vasopressors prior to CVC placement and 129 (35.5%) who did not. Use of peripheral vasopressors did not vary over the study period (*p* = 0.39 for trend). Complications occurred in 3/500 patients who received peripheral vasopressors (0.6%; 0 Grade 3 +), and in 14/363 patients who had a CVC placed (3.9%; 42.9% Grade 3 +).

**Conclusions:** In this large, multicenter trial, peripheral vasopressor use was common and complications were rare and low-grade, suggesting peripheral vasopressors can likely be used safely in clinical practice.

**Acknowledgement:** Submitted on behalf of the NHLBI PETAL Network.

## P221

### Midodrine for the early liberation from vasopressor support in the ICU – LIBERATE

#### D Opgenorth^1^, S Kilcommons^1^, K Fiest^2^, J Kutsogiannis^1^, E Macintyre^1^, J Senaratne^1^, J Slemko^1^, X Wang^3^, SM Bagshaw^1^, OG Rewa^1^

##### ^1^University of Alberta, Department of Critical Care Medicine, Edmonton, Canada, ^2^University of Calgary, Cumming School of Medicine, Calgary, Canada, ^3^Alberta Health Services, Health Services Statistical and Analytic Methods, Edmonton, Canada

*Critical Care* 2023, **27(S1)**: P221

**Introduction:** A pilot study to determine the feasibility of conducting an adequately powered clinical trial to evaluate the efficacy of the implementation of an oral vasoactive agent (midodrine) for early liberation of IV vasopressor support in ICU patients.

**Methods:** This was a single centre, concealed-allocation parallel-group blinded pilot RCT. Patients were randomly assigned in a 1:1 ratio to midodrine (enteral, 10 mg every 8 h) or placebo for the duration of their IV vasopressor therapy and 24 h following the discontinuation of their IV vasopressor therapy. The investigation product (IP) was administered orally, or by ng tube. Primary outcomes were feasibility of recruitment and duration of IV vasopressor support. Secondary outcomes included ICU length of stay, 90 day all-cause mortality, rates of ICU re-admission, rates of re-initiation of IV vasopressors and safety endpoints.

**Results:** 20 patients were enrolled into the study. One patient withdrew consent prior to data collection. 11 patients were assigned to midodrine and 9 to placebo. Median age was 60 (49.5–64.5) and 56.5 (43.2–68.2) respectively. 54.5% of the midodrine group were female vs 12.5% in the placebo group. Median APACHE II scores were 24.0 (19.5–27.5) and 17.5 (11.5–27.2). Treatment interventions measured included invasive mechanical ventilation (72.7% and 75.0%), CRRT (9.1% and 12.5%), IV hydrocortisone (36.4% and 37.5%) and blood products (45.4% and 37.5%). Outcomes measured included total duration of IV vasopressor support (hours) (48.6 (20.2–120.5) and 37.5 (21.4–81.0), vasopressors re-initiated over 24 h after ICU discharge (36.4% and 25.0%), ICU mortality (9.1% and 37.5%), hospital mortality (18.2% and 37.5%) and ICU length of stay 6.0 (2.5–14.8) and 4.2 (3.2–6.2) days. No adverse events related to the IP were identified.

**Conclusions:** This pilot study provides evidence that recruitment into a larger multi-site RCT is feasible to evaluate the role of midoddrine in earlier liberation from IV vasopressor therapy.

## P222

### Effects of levosimendan on cardiovascular performance in critically ill patients

#### TY Kim, EM Bowcock, CF Duncan, RA Rowley, SR Orde

##### Nepean Hospital, Intensive Care Unit, Kingswood, Australia

*Critical Care* 2023, **27(S1)**: P222

**Introduction:** Cardiac dysfunction is a frequent complication in critically ill patients. Levosimendan is a unique inodilator that does not increase intracellular calcium concentration while relaxing vascular smooth muscle, hence, reducing preload and afterload of left ventricle (LV) and right ventricle (RV). There is still a lack of data assessing the trend of cardiovascular effects of levosimendan in critically ill patients.

**Methods:** Single centre, retrospective parallel analysis of 49 patients with non-septic cardiomyopathy (NC) and septic cardiomyopathy (SC). Echocardiogram was performed median time of 1 day prior to levosimendan infusion and median time of 3 days after levosimendan infusion. Physiological markers were collected 24 h before and after therapy. Echocardiographic parameters of LV and RV function were measured by cardiac sonographers and reported by intensivists with advanced echocardiography qualifications. These values were compared between NC and SC.

**Results:** Prior to the commencement of levosimendan infusion, patients received median amount of 46 ml/kg fluid resuscitation and 40% were on at least two cardiovascular support agents. pH and lactate improved after levosimendan therapy (7.3 vs 7.4, *p* < 0.01; 2.2 vs 1.3 mmol/l, p 0.01). LV ejection fraction, LV outflow tract velocity time integral (LVOT-VTI) and RVOT-VTI were higher post-therapy (31 vs 44%, *p* < 0.01; 12 vs 15 cm, *p* < 0.01; 7 vs 10 cm, *p* < 0.01). Tricuspid annular plane systolic excursion increased following therapy (15 vs 17 mm, *p* < 0.01). RVOT-VTI/pulmonary artery systolic pressure also improved (0.20 vs 0.25 cm/mmHg, p 0.03). Improvement in LV systolic function were more prominent in NC than SC.

**Conclusions:** Early use of levosimendan infusion may be helpful in improving LV and RV systolic function in critically ill patients, especially in NC. It may also increase pulmonary artery compliance. Further prospective echocardiographic studies are required to assess its therapeutic benefit amongst pre-defined cardiac dysfunction phenotypes.

## P223

### Predictors for failure of early recovery after totally endoscopic aortic valve replacement (TEAVR)

#### S Lambrecht^1^, J Dubois^2^, L Heremans^1^, D Vranken^1^, I Gruyters^1^, E Geerts^1^, I De Pauw^1^, L Geebelen^1^, B Stessel^1^, J Vandenbrande^1^

##### ^1^Jessa, Anesthesiology & Intensive Care, Hasselt, Belgium, ^2^Jessa, Critical Care, Hasselt, Belgium

*Critical Care* 2023, **27(S1)**: P223

**Introduction:** We investigated the association between perioperative variables and failure for Enhanced Recovery After Cardiac Surgery (ERACS). ERACS is gaining importance since it has been shown to be safe and cost-reducing. Identification of risk factors for ERACS Failure (EFa) is useful in the optimization of individual patients on one hand and in the further expansion of ERACS on the other.

**Methods:** We performed a retrospective analysis on our TEAVR database. All patients with EuroSCORE II ≤ 3 are enrolled in our ERACS pathway. Postoperatively, patients are admitted to the Intensive Care Department (ICU) while ventilated under dexmedetomidine sedation. Analgesia consists of the routine administration of acetaminophen and ketorolac (unless contra-indications) and supplemental opioids as needed. The primary purpose is to achieve extubation within 6 h after surgery, as stated in the ERACS guidelines. Hence, we defined EFa as intubation for more than 6 h postoperatively and/or ICU length of stay for more than 1 day. Based on the literature, 6 variables (Table 1) were analyzed as possible predictors for EFa. First, a univariate regression analysis was performed. Variables with p value < 0,1 were taken into the multivariate regression analysis.

**Results:** Between 10/2019 and 12/2021, 176 TEAVR patients were included in the ERACS pathway. The ERACS trajectory was unsuccesful in 55/176 (31%) patients: 20/55 (36%) were intubated ≥ 6 h postoperatively, 40/55 (73%) were not discharged on postoperative day 1 and 5/55 (9%) had a combination of both. After multivariate regression analysis, the need for revision surgery < 24 h appeared to be the main risk factor for unsuccessful ERACS after TEAVR, whereas a higher EuroSCORE II showed no significant association with EFa. Preoperative use of RAAS inhibition did not appear to be a risk factor for failure of ERACS.

**Conclusions:** Revision surgery within 24 h was the only identifiable risk factor for failure of the ERACS trajectory in our TEAVR patient population.

**Table Tabbt:** **Table 1 (abstract P223)**. Results of the univariate and multivariate regression analyses on potential risk factors for failure of ERACS after TEAVR

Patients n = 176	Odds ratio [95% CI] univariate	*p* value univariate	Odds ratio [95% CI] multivariate	*p* value multivariate
Age (years)	1.00 [0.97, 1.03]	0.93		
EuroSCORE II	1.42 [0.95, 2.13]	0.09	1.43 [0.93, 2.19]	0.10
Revision surgery < 24 h	5.95 [1.12, 31.70]	0.04	6.02 [1.10, 33.10]	0.03
Coronary artery disease	0.83 [0.28, 2.46]	0.74		
RAAS inhibitors	0.72 [0.37, 1.41]	0.34		
BMI (kg/m^2^)	1.04 [0.97, 1.10]	0.29		
Chronic kidney disease (eGFR < 50 ml/min)	1.78 [0.70, 4.51]	0.22		

Variables with univariate *p* value < 0.1 were taken into the multivariate regression analysis

## P224

### Time for restoration of radial arterial pressure after a transient arm vascular occlusion correlates with outcome in critically ill patients: preliminary results of an observational study

#### C Lai, R Shi, JL Teboul, F Moretto, L Guérin, T Pham, X Monnet

##### AP-HP, Service de médecine intensive-réanimation, Hôpital de Bicêtre, DMU 4 CORREVE, Inserm UMR S_999, FHU SEPSIS, CARMAS, Université Paris-Saclay, Le Kremlin-Bicêtre, France

*Critical Care* 2023, **27(S1)**: P224

**Introduction:** Septic shock is characterized by an impairment of vascular reactivity as assessed by the resaturation slope of the muscular tissue oxygen saturation after a transient vascular occlusion (TVO). Another way to assess vascular reactivity might be to observe the time of the increase in radial arterial pressure after a TVO of the arm.

**Methods:** In mechanically ventilated patients equipped with a radial artery catheter, a brachial cuff was rapidly inflated to induce a transient arterial stop-flow. Arterial pressure decreased to the mean systemic pressure (Pms_arm_). After a 60-s occlusion, the cuff was deflated and time to return from Pms_arm_ to baseline arterial pressure was measured (T_revasc_).

**Results:** We included 59 patients, among whom 45 (76%) where in shock, including 22 (37%) with septic shock. Twenty-three (39%) had acute respiratory distress syndrome and 12 (20%) were intubated for neurological impairment. The dose of norepinephrine was 0.46 (0.19–0.93) μg/kg/min in patients with shock. Measurements were obtained 3 (1–5) days after onset of mechanical ventilation or vasopressors infusion. Mean arterial pressure was 83 ± 12 mmHg, Pms_arm_ was 28 ± 9 mmHg, central venous pressure was 13 ± 4 mmHg and the (Pms_arm_-CVP) gradient was 15 ± 8 mmHg, with no difference between patients with or without shock. In the whole population, T_revasc_ was 26 (20–37) sec, with no difference between patients with or without shock (28(23–38) sec vs. 22(18–33) sec, respectively, *p* = 0.14). Among shock patients, T_revasc_ was longer in patients with septic shock than in the other ones (37 (29–44) vs. 23 (19–27) sec, respectively, *p* < 0.001). T_revasc_ was increased in the 24 (41%) patients who died compared to survivors (35 ± 19 vs. 27 ± 11 s, respectively, *p* = 0.03).

**Conclusions:** T_revasc_ was increased in patients with septic shock compared to patients with non-septic shock or without shock. Mortality was associated with higher T_revasc_. This new variable may indicate vasoreactivity and be associated with mortality.

## P225

### Adding an extension piece to the end of the purge side arm of the Impella device can prevent the incidence of the Impella device failure rate secondary to the cassette breaking and positively impact patient outcome

#### P Satashia^1^, J Blasavage^1^, A White^1^, S Isha^1^, S Paghdar^1^, S Kiley^1^, P Moreno Franco^1^, P Patel^2^, S Chaudhary^1^, D Sanghavi^1^

##### ^1^Mayo Clinic Florida, Critical Care Medicine, Jacksonville, USA, ^2^Mayo Clinic Florida, Advanced Heart Failure and Heart Transplantation, Jacksonville, USA

*Critical Care* 2023, **27(S1)**: P225

**Introduction:** Impella 5.5® with Smart Assist is a type of minimally invasive left ventricular assist device (LVAD) approved by the Food and Drug Administration (FDA) for the treatment of ongoing cardiogenic shock for up to 14 days. The Impella® intends to reduce ventricular workload and provide the circulatory support necessary for myocardial recovery. The replacement of the purge tube led to the breakage of the luer lock connector leading to Impella device failure, and ultimately needed device replacements. The practice of adding the extension piece to the purge tube sidearm was implemented to avoid such incidents.

**Methods:** A retrospective chart review of ICU patients was done at a tertiary care center from August 2018 to August 2022 to assess the differences in patient outcomes related to Impella® Device utilization before and after the implementation of the extension piece to the purge tube sidearm. Among patients reviewed, a total of 18 were included in our review, with 5 not having the purge tube side arm extension added, while 13 patients had the extension.

**Results:** There was no significant difference in patient health outcomes between the two study groups. Additionally, there were no instances of device failure requiring explanation without the extension tubing. However, there were no cases of the purge cassette cracking with the addition of the extension tubing added.

**Conclusions:** The addition of extension tubing to the purge cassette of the Impella® Device did not impact patient health outcomes or affect the incidence of device failure. There was a complete reduction in the incidence of the purge cassette cracking, which could reduce the potential for infection or device failure over a long period of mechanical support. There is a need for long-term prospective studies to confirm the results.

## P226

### Prehospital advanced life support by physician versus emergency medical service personnel after pediatric out-of-hospital cardiac arrest: results from a Japanese registry-based study

#### T Fukuda^1^, K Matsui^2^, M Amaki^2^, H Sekiguchi^3^

##### ^1^Toranomon Hospital, Department of Emergency and Critical Care Medicine, Tokyo, Japan, ^2^Toranomon Hospital, Tokyo, Japan, ^3^University of the Ryukyus, Okinawa, Japan

*Critical Care* 2023, **27(S1)**: P226

**Introduction:** Controversy remains as to whether advanced life support (ALS) should be provided for pediatric out-of-hospital cardiac arrest (OHCA) in the prehospital setting. We sought to assess whether prehospital ALS by physician would be associated with an increased chance of neurologically favorable survival after pediatric OHCA compared with prehospital ALS by emergency medical service (EMS) personnel.

**Methods:** We conducted a nationwide population-based observational study using the Japanese government-led registry data of OHCA. This study included pediatric patients (1–17 years) who experienced OHCA in Japan from January 1, 2011, to December 31, 2019. The primary outcome was 1-month neurologically favorable survival with the Glasgow-Pittsburgh cerebral performance category score of 1 or 2.

**Results:** A total of 2532 patients were included (mean [SD] age, 11.3 [5.5] years; 65.5% male); 1054 received prehospital ALS by physician, and 1478 received prehospital ALS by EMS personnel. Among these patients, 187 (7.4%) survived with a favorable neurological status 1 month after OHCA, including 127/1054 (12.0%) for ALS by physician, and 60/1478 (4.1%) for ALS by EMS personnel. 757 patients receiving prehospital ALS by physician were propensity-matched with 757 patients receiving prehospital ALS by EMS personnel. In the propensity score-matched cohort, prehospital ALS by physician was associated with an increased chance of neurologically favorable survival compared with prehospital ALS by EMS personnel (8.9% vs. 4.1%; RR 2.16; 95% CI 1.43–3.27; *p* = 0.0002).

**Conclusions:** Among pediatric OHCA patients, prehospital ALS by physician was associated with an increased chance of neurologically favorable survival compared with ALS by EMS personnel in the prehospital setting.

## P227

### AED delivery at night – can drones do the job? A feasibility study of unmanned aerial systems to transport automated external defibrillators during night-time

#### SS Scholz^1^, D Wähnert^2^, G Jansen^3^, O Sauzet^4^, E Latka^5^, S Rehberg^1^, KC Thies^1^

##### ^1^Protestant Hospital of Bethel Foundation, University Hospital OWL, Department of Anaesthesiology, Intensive Care, Emergeny Medicine, Transfusion Medicine and Pain Therapy, Bielefeld, Germany, ^2^Protestant Hospital of Bethel Foundation, University Hospital OWL, Department of Orthopaedics and Trauma Surgery, Bielefeld, Germany, ^3^Klinikum Bielefeld, Department of Anaesthesiology, Intensive Care, Emergency Medicine and Pain Therapy, Bielefeld, Germany, ^4^University Bielefeld, Epidemiology and International Public Health, Bielefeld School of Public Health, Bielefeld, Germany, ^5^Studieninstitut für kommunale Verwaltung Westfalen-Lippe, Fachbereich Medizin und Rettungswesen, Bielefeld, Germany

*Critical Care* 2023, **27(S1)**: P227

**Introduction:** In their recent guidelines the European Resuscitation Council have recommended the use of Unmanned Aerial systems (UAS) to overcome the notorious shortage of AED. Exploiting the full potential of airborne AED delivery would mandate 24 h UAS operability. However, current systems have not been evaluated for nighttime use [1, 2]. The primary goal of our study was to evaluate the feasibility of night-time AED delivery by UAS. The secondary goal was to obtain and compare operational and safety data of night versus day operations.

**Methods:** We scheduled two (one day, one night) flights each to ten different locations to assess the feasibility of AED delivery by UAS during night-time. We also compared operational data and safety data of night versus day missions.

**Results:** All missions were completed without safety incident. The flights were performed automatically without interventions, apart from manually choosing the landing site and correcting the descent. Flight distances ranged from 910 to 6960 m, corresponding mission times from alert to AED release between 3:48 min and 11:20 min. Night missions (T_m:night_ = 7:26 ± 2:29 min) did not take longer than day missions (T_m:day_ = 7:59 ± 2:27 min). Despite inferior visibility of the target site at night, statistical analysis suggested non-inferiority of night landings versus day landings (*p* = 0.9).

**Conclusions:** Our results demonstrate the feasibility of UAS supported AED delivery during nighttime. Operational and safety data indicate no major differences between day- and night-time use. Future research should focus on integration of drone technology into the chain of survival.


**References**
Baumgarten MC et al. Resuscitation 2022;172:139–145.Schierbeck S et al. Eur Heart J 2022;43:1478–1487.


## P228

### Reliability and limits of bag valve mask (BVM): a comparative bench study

#### A Broc^1^, F Morin^2^, L Polard^1^, A Drouet^3^, H Schmit^4^, L Lahmaut^5^, M Taillantou-Candau^6^, F Beloncle^6^, JC Richard^6^, D Savary^2^

##### ^1^Air Liquide Medical Systems, Med2Lab, Antony Cedex, France, ^2^University Hospital of Angers, Emergency Department, Angers, France, ^3^CHUV Centre Hospitalier Universitaire Vaudois, Emergency Department, Lausanne, Switzerland, ^4^Centre Hospitalier Annecy Genevois, Emergency Department, Annecy, France, ^5^SAMU de Paris and Intensive Care Unit, Necker Hospital, Assistance Publique-Hopitaux de Paris (APHP), Emergency Department, Paris, France, ^6^University Hospital of Angers, Medical ICU, Angers, France

*Critical Care* 2023, **27(S1)**: P228

**Introduction:** BVM is the most common device used for pre-oxygenation and ventilation during resuscitation. The objective is to compare the performances of 9 BVM in simulated pre-oxygenation and cardiopulmonary resuscitation (CPR) conditions in terms of inspired fraction of oxygen (FiO_2_), insufflated volume (VTi) and rebreathing.

**Methods: 9** BVM were evaluated according to a specific bench protocol.Two levels of spontaneous breathing (occurring during pre-oxygenation) were simulated on an ASL test lung at ZEEP and PEEP 5 with 15 L/min O_2_ for each BVM tested. Actual FiO_2_ was measured and compared to the minimum 85% FiO_2_ target imposed by the ISO standard.BVM ventilation during CPR was performed by healthcare volunteers on a specific manikin. Ventilation was tested during mechanical (Lucas 2) Interrupted Chest Compressions strategy (30:2) and after Return of Spontaneous Circulation (ROSC) according to CPR guidelines. 36 caregivers from 2 University Hospitals and 1 General Hospital provided manual ventilation.BVM were drilled to evaluate CO_2_ rebreathing during simulated spontaneous ventilation.


**Results:**
Spontaneous breathing: The actual FiO_2_ delivered did not reach 85% for Weinmann (32%), O-two (48%), Intersurgical Small (60%) and Laerdal (83%) (Fig. 1, Panel A). Interestingly, FiO_2_ was above 85% in all BVM tested when a PEEP valve was added (Panel B).Ventilation during CPR and after ROSC: actual VTi delivered were in the target range 5 (± 1) ml/PBW but 4 BVM delivered significantly less volume (MI3, Mercury Small, Intersurgical Small and O-two).Rebreathing: 3 BVM (Intersurgical Adult, Mercury Small, Laerdal) showed significant CO_2_ rebreathing.


**Conclusions:** The 9 BVM tested exhibited varying performances on this bench study. Actual FiO_2_ delivered during simulated pre oxygenation was substantially less than the minimal 85% FiO_2_ target in 4 BVM in these experimental conditions while 3 of them exhibited CO_2_ rebreathing.

**Figure Figcp:**
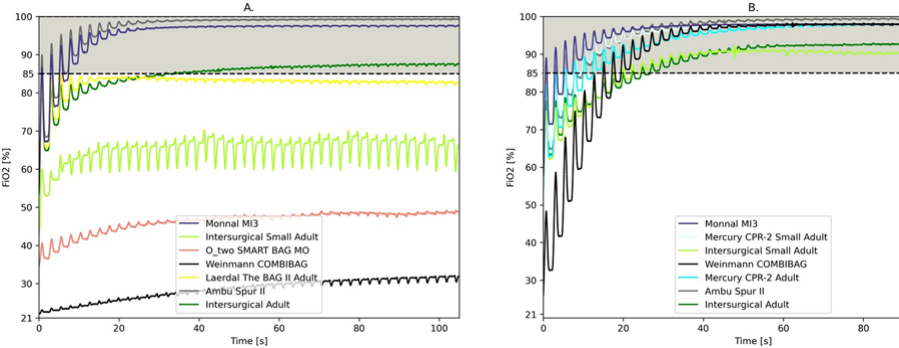
**Fig. 1 (abstract P228)**. Actual FiO_2_ measured in % according to time. Spontaneous breathing was simulated with a severe patient profile with a fixed breath of 500 ml, I:E ratio of 1:3, C = 40 ml/cmH_2_O, R = 10 cmH_2_O/l/s. Panel A. ZEEP. Panel B. PEEP = 5 cmH_2_O

## P229

### Supraglottic airway versus endotracheal intubation in out-of-hospital cardiac arrest

#### LD Darginavičius, MZ Zaremba, DB Bajoriūnaitė

##### Lithuanian University of Health Sciences, Emergency Department, Kaunas, Lithuania

*Critical Care* 2023, **27(S1)**: P229

**Introduction:** Airway management in a prehospital setting during resuscitation in patients with nontraumatic out-of-hospital cardiac arrest (OHCA) using a non-cuffed laryngeal device or endotracheal intubation (ETI) remains controversial. Our aim is to compare the time of airway establishment with a non-cuffed laryngeal device and ETI in patients with nontraumatic OHCA and to evaluate the effect of non-cuffed laryngeal device insertion and endotracheal intubation on the return of spontaneous circulation (ROSC).

**Methods:** This study is a retrospective analysis of 479 patients in Lithuania who had an OHCA in whom resuscitation was attempted by emergency medical personnel with subsequent transport to medical institutions from January 2020 through December 2021. The primary outcome was ROSC on arrival at the hospital. The secondary outcome was successful ventilation using either ETI or a non-cuffed laryngeal device. Capnography measures between 35 and 45 mmHg were defined as successful ventilation following Kaunas city EMS protocol.

**Results:** Among 479 enrolled patients, 3 were traumatic out-of-hospital cardiac arrest cases, and 166 patients received either no airway management device, or both a non-cuffed laryngeal device and EIT. These cases were excluded. 176 (36.9%) received a non-cuffed laryngeal device, and 129 (27.1%) received EIT as the primary airway management device. The remaining patients were managed without an advanced airway technique. ROSC on arrival at the hospital was achieved in 126 (26.4%) cases. Further study results will be presented.

**Conclusions:** Among patients with non-traumatic out-of-hospital cardiac arrest in Kaunas city EMS, a non-cuffed laryngeal device insertion was associated with a greater ROSC on arrival to the hospital than ETI. These findings suggest that non-cuffed laryngeal device insertion may be considered an initial airway management strategy in patients with non-traumatic OHCA. However, these are not the primary factors influencing successful resuscitation.

## P230

### A novel capnogram analysis to guide ventilation during cardiopulmonary resuscitation: clinical and experimental observations

#### A Lesimple^1^, A Hutin^2^, D Savary^3^, F Lidouren^4^, F Beloncle^5^, A Broc^6^, A Mercat^5^, L Brochard^7^, R Tissier^4^, JC Richard^5^

##### ^1^Air Liquide Medical Systems, Medical Department, Antony, France, ^2^SAMU of Paris, Necker Hospital, SAMU, Paris, France, ^3^Emergency Department, University Hospital of Angers, Emergency, Angers, France, ^4^Ecole Nationale Vétérinaire d’Alfort, IMRB, Ecole Nationale Vétérinaire d’Alfort, Maisons-Alfort, France, ^5^Medical ICU, Angers University Hospital, University of Angers, Intensive Care Unit, Angers, France, ^6^Air Liquide Medical Systems, Med2Lab, Antony, France, ^7^Keenan Research Centre for Biomedical Science, Li Ka Shing Knowledge Institute, St. Michael’s Hospital, Keenan Research Centre for Biomedical Science, Toronto, Canada

*Critical Care* 2023, **27(S1)**: P230

**Introduction:** During cardiopulmonary resuscitation (CPR), chest compressions (CC) tend to decrease lung volume below the functional residual capacity (FRC). This may induce the recently described “intrathoracic airway closure” [1]. On the opposite, large insufflations above the FRC can generate thoracic distension and may jeopardize circulation. These phenomena affect differently capnogram during CC. The objective of the present study was to test whether a specific capnogram may identify thoracic distension during CPR and to assess the impact of thoracic distension on gas exchange and hemodynamics.


**Methods:**
In out-of-hospital cardiac arrest patients, we identified on capnograms three patterns: intrathoracic airway closure, thoracic distension or regular pattern. An algorithm was designed to classify them automatically.To link CO_2_ patterns with ventilation, we conducted two experiments: reproducing the CO_2_ patterns in human cadavers exploring the impact of thoracic distension patterns on different circulation parameters during CPR on a pig model.



**Results:**


Clinical data: 202 capnograms were collected. Intrathoracic airway closure was present in 35%, thoracic distension in 22% and regular pattern in 43% (Fig. 1).

Experiments:Higher insufflated volumes reproduced thoracic distension CO_2_ patterns in 5 cadavers.In six pigs during CPR with various tidal volumes, a CO_2_ pattern of thoracic distension, but not tidal volume per se, was associated with a significant decrease in blood pressure and cerebral perfusion.

**Conclusions:** EtCO_2_ interpretation during CC remains challenging. In this context, we proposed an original approach to identify and interpret more accurately expired CO_2_ during CC. Both intrathoracic airway closure and thoracic distension can be identified using CO_2_ patterns. Further research is needed to assess the impact on ventilation and circulation of a ventilatory strategy guided by capnograms.


**Reference**
Grieco et al. Am J Respir Crit Care Med 2019;199:728–737.

**Figure Figcq:**
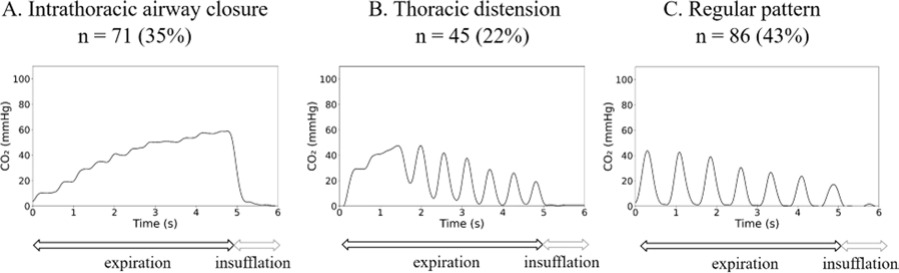
**Fig. 1 (abstract P230)**. The figure illustrates the distribution of capnograms according to the classification. Each panel shows a typical CO_2_ pattern obtained from clinical observations after numerical treatment from raw capnogram data (python, Python Software Foundation, Wilmington, Delaware, USA). X-axis represents inspiratory and expiratory time. **A** Intrathoracic airway closure: oscillations due to chest compressions and decompressions are small or absent. **B** Thoracic distension: oscillations due to chest compressions and decompressions are limited or absent at the beginning of the expiration phase and resume after a few chest compressions. **C** Regular pattern: oscillations due to chest compressions and decompressions are clearly visible during the entire duration of the expiration phase. The regular pattern corresponds to the situation when neither thoracic distension nor intrathoracic airway closure is identified

## P231

### No differences in blood gases or lactate with 30:2 vs continuous CPR when applying PEEP

#### J Kopra^1^, E Litonius^2^, PT Pekkarinen^3^, M Laitinen^4^, J Heinonen^5^, L Fontanelli^6^, MB Skrifvars^1^

##### ^1^HUCS, Department of Emergency Care and Services, Helsinki, Finland, ^2^HUCS, Division of Anaesthesiology, Department of Anaesthesiology, Intensive Care and Pain Medicine, Helsinki, Finland, ^3^HUCS, Division of Intensive Care, Department of Anaesthesiology, Intensive Care and Pain Medicine, Helsinki, Finland, ^4^VetCT, VetCT Teleconsulting – Teleradiology Small Animal Team, Helsinki, Finland, ^5^Päijät-Häme Central Hospital, Päijät-Häme Central Hospital/Emergency Medical Services, Lahti, Finland, ^6^University of Pavia, Department of Clinical-Surgical, Diagnostic and Paediatric Sciences, Unit of Anaesthesia and Intensive Care, Pavia, Italy

*Critical Care* 2023, **27(S1)**: P231

**Introduction:** In a refractory out-of-hospital cardiac arrest (OHCA), the patient is commonly transported to hospital using mechanical CPR and ventilation with 100% oxygen. However, many patients arriving to hospital are hypoxic with hypercarbia. One possible reason for the inadequate ventilation is due to the counterpressure caused by the continuous chest compressions (CCC). We hypothesized that a compression/ventilation ratio of 30:2 would provide better ventilation and gas exchange compared to CCC over ventilation during prolonged CPR. We wanted to explore the effect of adding 10 cmH_2_O PEEP to the protocols.

**Methods:** We randomized 23 anaesthetized domestic swine (weight approximately 57 kg) with electrically induced ventricular fibrillation (VF) to CCC or 30:2 chest compressions and manual bag-valve ventilation with 100% FiO_2_ and 10 cmH_2_O PEEP. We started CPR after a 5 min no-flow period and continued it up to 40 min from the induction of VF. Chest compressions were performed with a mechanical chest compression device (LUCAS®, Stryker Medical). The pigs were not defibrillated but received 3 standard doses of epinephrine i.v. during CPR. We collected arterial blood gas samples every 5 min during CPR. PaO2, PaCO2, and lactate over time were compared using a mixed linear model.

**Results:** There were no statistically significant differences in PaO_2_ (*p* = 0.11), PaCO_2_ (*p* = 0.59), or lactate (*p* = 0.57) between continuous and 30:2 compression/ventilation groups (Fig. 1). The *p* values for the interaction between compression/ventilation group and time were as follows: 0.25 for PaO_2,_ 0.96 for PaCO_2_, and 0.19 for lactate.

**Conclusions:** Prolonged CPR with mechanical chest compressions performed either continuously or with a 30:2 compression/ventilation ratio with 10 cmH_2_O PEEP resulted in similar arterial levels of oxygen, carbon dioxide, and lactate.

**Figure Figcr:**
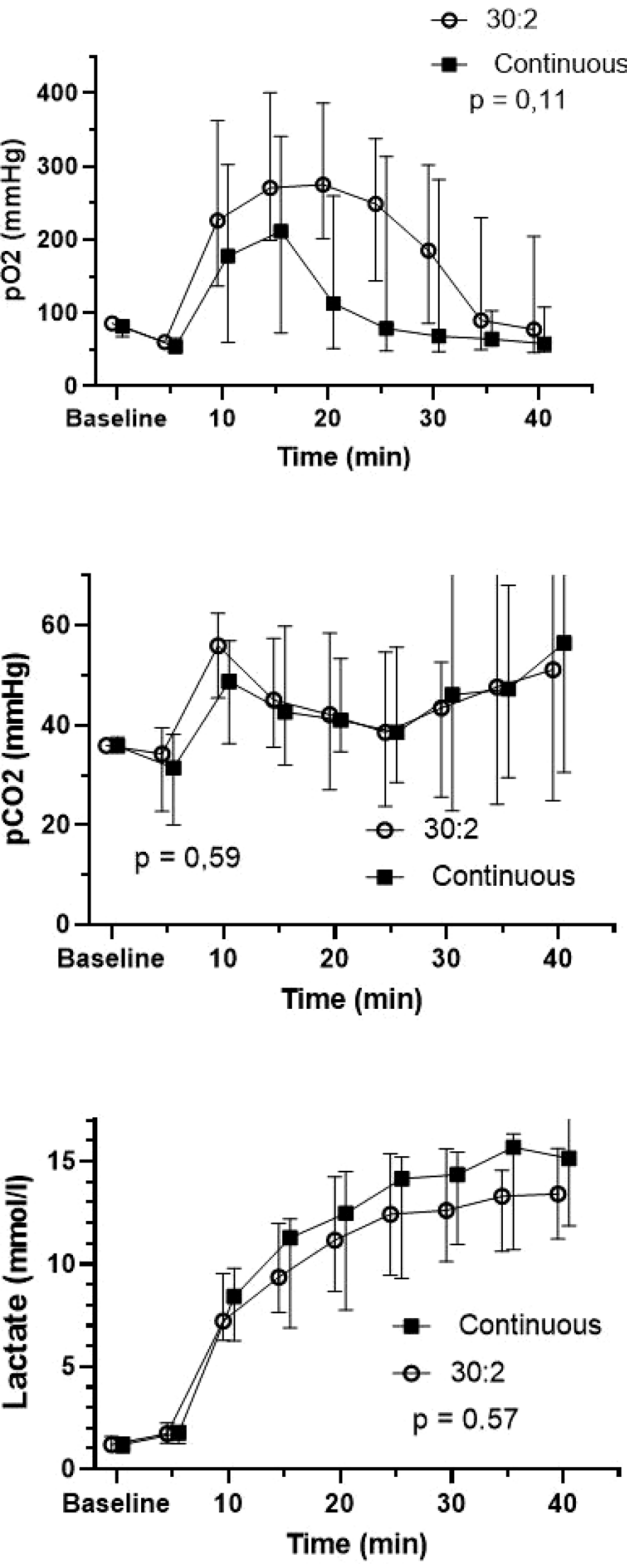
**Fig. 1 (abstract P231)**. Measurements in arterial blood samples. PaCO_2_, lactate, and PaO_2_ median values and IQR:s in the 30:2 compression/ventilation and continuous compression/ventilation groups at baseline measurements and at 5 min intervals after the cardiac arrest. The CPR was started at 5 min time point

## P232

### Out of hospital cardiac arrest ventilation using Cardiopulmonary Ventilation™ is associated with increased incidence of return of spontaneous circulation

#### S Malinverni^1^, M Sarnelli^1^, D De Longueville^1^, M Bartiaux^1^, BCAR Group^2^

##### ^1^University hospital Saint Pierre, Emergency, Bruxelles, Belgium, ^2^ B-CAR Research Group, Emergency, Anderlecht, Belgium

*Critical Care* 2023, **27(S1)**: P232

**Introduction:** The purpose of this study is to investigate the role of Cardiopulmonary Ventilation™ (CPV) in return of spontaneous circulation (ROSC) in out of hospital cardiac arrests (OHCA). CPV ventilation is a pressure control ventilation with a respiratory rate of 10 breath/min a FiO_2_ of 1, an initial inspiratory pressure of 20 cmH_2_O and a positive end expiratory pressure of 5 cmH_2_O. It includes a specific algorithm for high pressure that magnifies intrathoracic positive pressure during chest compression cardiac to increase the associated cardiac ejection; meanwhile it magnifies negative intrathoracic pressure during chest recoil, therefore enhancing venous return to the heart.

**Methods:** We performed a retrospective analysis of all non-traumatic adult cardiac arrests recorded in the Belgian cardiac arrest registry (B-CAR) form January 2017 until December 2021. We excluded traumatic arrests, pregnant women and minors from the analysis. A multivariable logistic regression backward elimination model that adjusted for known confounders was used to identify factors independently associated with ROSC.

**Results:** 1936 patients were included in the analysis. 215 (11.1%) patients were ventilated with CPV. 638 (32.9%) of OHCA victims were female and median age was 70 years (IQR 59–80). 565 (29.2%) of patients presented with a shockable rhythm and median no flow duration was 5 min (IQR1-11). Ventilation with CPV during CPR was associated, in a multivariable adjusted analysis, with an increase in the proportion of ROSC OR 1.52 (1.12–2.07); *p* = 0.007 (Table 1).

**Conclusions:** In our retrospective analysis of non-traumatic Belgian OHCA, ventilation with the CPV mode was associated with higher chances of return of spontaneous circulation.

**Table Tabbu:** **Table 1 (abstract P232)**. Adjusted analysis for factors associated with ROSC

	Odds ratio	95% Confidence interval	*p* value
CPV	1.53	1.12–2.07	0.007
Age, years	0.99	0.98–0.99	0.007
No flow, min	0.94	0.93–0.96	< 0.001
Shockable rhythm	2.62	2.1–3.18	< 0.001
Female	1.26	1.02–1.55	0.029

*CVP* cardiopulmonary ventilation

## P233

### Understanding the effect of applied pressure on hemodynamics during targeted CPR using a new integrated computational model

#### F Varghese^1^, A Cruz^2^, J Herrmann^2^, B Harvey^2^, G Beck^1^, R Branson^3^, D Kaczka^4^, J Lampe^1^

##### ^1^ZOLL Medical, Chelmsford, USA, ^2^University of Iowa, Iowa, USA, ^3^University of Cincinnati, Cincinnati, USA, ^4^ University of Iowa, Department of Anesthesia, Iowa, USA

*Critical Care* 2023, **27(S1)**: P233

**Introduction:** The thoracic pump mechanism relates chest compression generated blood flow to oscillations in intrathoracic pressure. Mechanical chest compression results in different pressures in different thoracic structures and these pressures are dependent on piston location during compression. We modeled the impact of these pressure measurements on chest compression generated blood flow using a recently developed lumped parameter computational model.

**Methods:** In a porcine VF model of cardiac arrest (n = 3), pressures in the aorta (AoP), esophagus (EsoP), right atrium (RAP), and left ventricle (LVP), as well as carotid and jugular flows, were measured during mechanical compressions (2 inch, 100 compressions per minute), targeting the left ventricle (LV) or left ventricular outflow tract (LVOT). Compression location alternated every 2 min for 6 min. Pressures averaged over four consecutive compressions were used as inputs into the model. During thoracic pump (TP) simulations, the AoP waveform was applied to all elements in the thorax. During modified thoracic pump model (MTP) simulations, the RAP was applied to the right atrium and right ventricle, the EsoP was applied to the pulmonary vasculature, and the LVP was applied to the left atrium, left ventricle and the systemic arteries.

**Results**: Table 1 shows the comparison between measured and modeled blood flows for compressions in both locations. The TP simulation appears a better model of LV compressions. The MTP simulation seems a better approximation of jugular blood flow in the LVOT compressions, but the TP model is a better approximation of the carotid blood flow.

**Conclusions:** The observation that a single pressure (TP Model) applied to all structures was a better approximation of experimental blood flows is surprising given the observed pressure differences during chest compressions in the animal model. There remains much to learn about chest compression generated blood flow.

**Table Tabbz:** **Table 1 (abstract P233)**. Mean blood flows at LV and LVOT compression locations from experimental data and computer model simulations

	Location	LV	LVOT
Experimental	Jugular (ml/s)	0.26	0.15
Experimental	Carotid (ml/s)	0.40	0.43
Thoracic pump model	Jugular (ml/s)	0.43	0.34
Thoracic pump model	Carotid (ml/s)	0.42	0.34
Modified thoracic pump model	Jugular (ml/s)	0.59	0.13
Modified thoracic pump model	Carotid (ml/s)	0.60	0.20

## P234

### Unchanged incidence but declining 6-month mortality of cardiac arrest occurring in Swedish ICUs

#### B Flam^1^, M Andersson Franko^2^, MB Skrifvars^3^, T Djärv^4^, M Cronhjort^5^, M Jonsson Fagerlund^1^, J Mårtensson^1^

##### ^1^Karolinska University Hospital, Perioperative Medicine and Intensive Care, Stockholm, Sweden, ^2^Karolinska Institutet, Department of Clinical Science and Education, South General Hospital, Stockholm, Sweden, ^3^Helsinki University Hospital and University of Helsinki, Department of Emergency Care and Services, Helsinki, Finland, ^4^Karolinska University Hospital, Medical Unit Acute/Emergency Department, Stockholm, Sweden, ^5^South General Hospital, Department of Anesthesiology and Intensive Care, Stockholm, Sweden

*Critical Care* 2023, **27(S1)**: P234

**Introduction:** We sought to determine temporal trends in incidence of cardiac arrest occurring in the ICU (ICU-CA) and its associated long-term mortality.

**Methods:** We performed a nationwide cohort study identifying ICU-CAs in Sweden between 2011 and 2017. ICU-CA was defined as a first episode of CPR and/or defibrillation following an ICU admission, as recorded in the Swedish Intensive Care Registry or the Swedish Registry of CPR. Annual adjusted ICU-CA incidence trends (all admissions) were estimated using propensity score-weighted analysis. Six-month mortality trends (first admissions) were assessed using multivariable logistic regression. Analyses were adjusted for pre-admission characteristics (sex, age, socioeconomic status, comorbidities, medications, and healthcare utilization), admission year, and illness severity on ICU admission.

**Results:** We included 231,427 adult ICU admissions. Crude ICU-CA incidence was 16.1/1000 admissions, with no significant annual trend on propensity score-weighted analysis. Among 186,530 first admissions, crude 6-month mortality in ICU-CA patients was 74.7% (95% CI 70.1–78.9) in 2011 and 68.8% (95% CI 64.4–73.0) in 2017. When controlling for multiple potential confounders, the adjusted 6-month mortality odds of ICU-CA patients decreased with 6% per year (95% CI 2–10) (Fig. 1). Patients admitted after out-of-hospital or in-hospital cardiac arrest had the highest ICU-CA incidence (136.1/1000) and subsequent 6-month mortality (76.0% [95% CI 73.6–78.4]).

**Conclusions:** In our nationwide Swedish cohort, the adjusted incidence of ICU-CA remained unchanged between 2011 and 2017. More than two thirds of patients with ICU-CA do not survive to six months following admission, but a slight improvement appears to have occurred over time.

**Figure Figcs:**
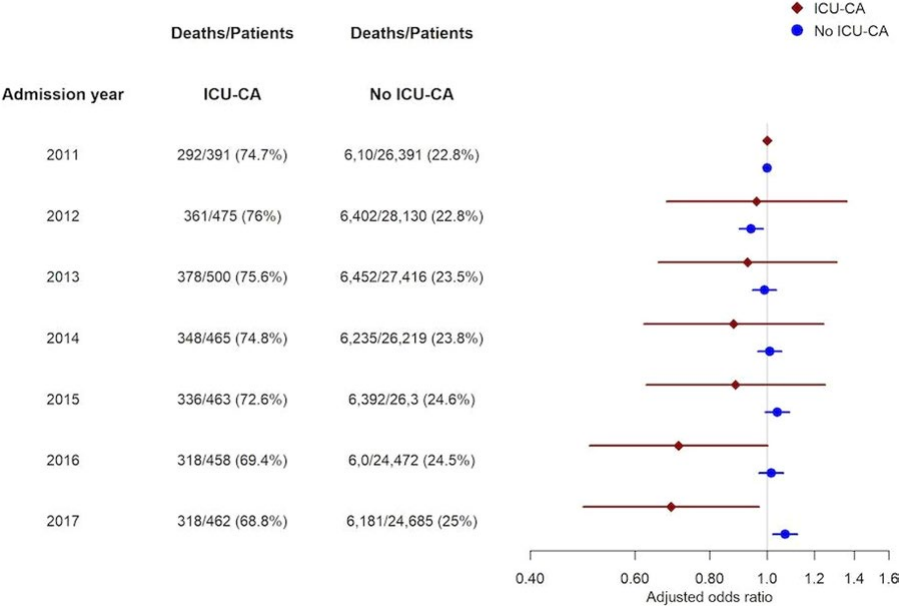
**Fig. 1 (abstract P234)**. Adjusted odds ratios (aORs) of 6-month mortality with corresponding 95% CIs for ICU-CA (red) and non-ICU-CA patients (blue), grouped by admission year (right). Over the study period, there was a declining trend in 6-month mortality for ICU-CA patients (annual aOR 0.94 [95% CI 0.90–0.98]; *p* [trend] = 0.005). Conversely, 6-month mortality for non-ICU-CA patients had increased (annual aOR 1.015 [95% CI 1.009–1.022]; *p* [trend] < 0.001), *p* (interaction) < 0.001. Annual crude mortality is displayed (left)

## P235

### Prognostic significance of ischemia–reperfusion intestine injury in patients with refractory cardiac arrest

#### J Smalcova^1^, O Franek^2^, M Huptych^3^, P Kavalkova^1^, D Rob ^1^, J Pudil^1^, M Fajkus^4^, J Belohlavek^1^

##### ^1^General University Hospital in Prague, 2nd Department of Internal Cardiovascular Medicine, Prague, Czech Republic, Emergency Medical Service Prague, Emergency Medical Service Prague, Prague, Czech Republic, ^3^Czech Technical University in Prague, Czech Institute of Informatics, Robotics and Cybernetics (CIIRC), Prague, Czech Republic, ^4^Tomas Bata University in Zlín, Faculty of Applied Informatics, Zlín, Czech Republic

*Critical Care* 2023, **27(S1)**: P235

**Introduction:** The duration of cardiac arrest (CA) with ischemia–reperfusion (IR) injury affects the severity of the post-resuscitation syndrome and prognosis in patients with refractory CA. Intestinal injury reflects the severity of IR damage, and may be a prognostic marker of poor neurological outcomes and prognosis.

**Methods:** In a randomized, prospective Prague OHCA study, we compared an invasive strategy (intra-arrest transport, ECPR) with standard on-site CPR in refractory cardiac arrest. The assessment of ischemia–reperfusion injury was based on clinical symptoms (profuse watery diarrhoea, higher nasogastric tube waste) in the early phase after CPR. We compared the incidence of intestinal damage in both study arms and its association with patients' neurological outcomes. We hypothesized that intestinal IR injury is more common in patients with refractory CA treated with ECPR (extracorporeal cardiopulmonary resuscitation). We assumed that the manifestation of the gut injury was associated with a worse prognosis.

**Results:** Intestinal damage was found in 14 of 132 patients, compared to 47 of 124 patients in the standard and invasive arms, respectively. This difference was statistically significant (*p* = 0.003). The adverse neurological outcome was observed in 39 out of 47 patients with IR injury in invasive arm and in 11 out of 14 patients in standard arm. The incidence of a poor neurological outcome (Cerebral Performance Categories 3–5) and prognosis in patients with IR intestine injury in the invasive arm on day 180 was statistically more significant compared to the standard arm (*p* = 0.007 vs. *p* = 0.46).

**Conclusions:** Ischemia–reperfusion intestine injury is observed more often in patients with refractory CA treated with ECPR. In these cases, intestinal injury is a significant adverse prognostic marker of poor neurological outcomes and prognosis.

## P236

### Knowledge and barrier of utilization among physicians about targeted temperature management and therapeutic hypothermia

#### Ali Al Bshabshe^1^, M Jan^2^

##### ^1^King Lhalid University, College of Medicine, Medicine/Critical Care, Abha, Saudi Arabia, ^2^King Lhalid University, College of Medicine, Anesthesia/Critical Care, Abha, Saudi Arabia

*Critical Care* 2023, **27(S1)**: P236

**Introduction:** We aim to evaluate the knowledge about targeted temperature management and therapeutic hypothermia and to get better understand of barriers to utilization among physicians who work with patients post-cardiac arrest [1,2].

**Methods:** An online survey distributed to the physicians working with patients at risk of cardiac arrest namely emergency medicine, internal medicine and critical care physicians at various cities in Saudi Arabia. The survey composed of demographic variables and information regarding facilities in the hospital's awareness of targeted temperature management and therapeutic hypothermia. Information about the potential barriers to the implementation of TH was also collected.

**Results:** Two hundred sixty-two replies were evaluated with a 65% response rate. Most of the responders, 90%, were males with all levels of medical practice, from residents to senior consultants. Around 80% of the participants had heard about targeted temperature management and therapeutic hypothermia. But unfortunately, 40% of them practice this therapeutic modality. When evaluating the barriers which limit physicians from using this therapeutic modality, we found that the Lack of knowledge (33%) was the significant barrier for utilizing Targeted temperature management and therapeutic hypothermia followed by the Lack of policy and procedure (26%), Lack of experience (16%), Lack of equipment's (13%), nursing issues (9%) and administrative issues were only (4%) (Fig. 1).

**Conclusions:** Lack of knowledge and experience and policy and procedures were the most common barriers to targeted temperature management and therapeutic hypothermia in a post-cardiac arrest setting.


**References**
Walker AC et al. Emerg Med Clin North Am. 2019;37:381–93.Becker LB et al. Ann Emerg Med. 1993;22:86–91.

**Figure Figct:**
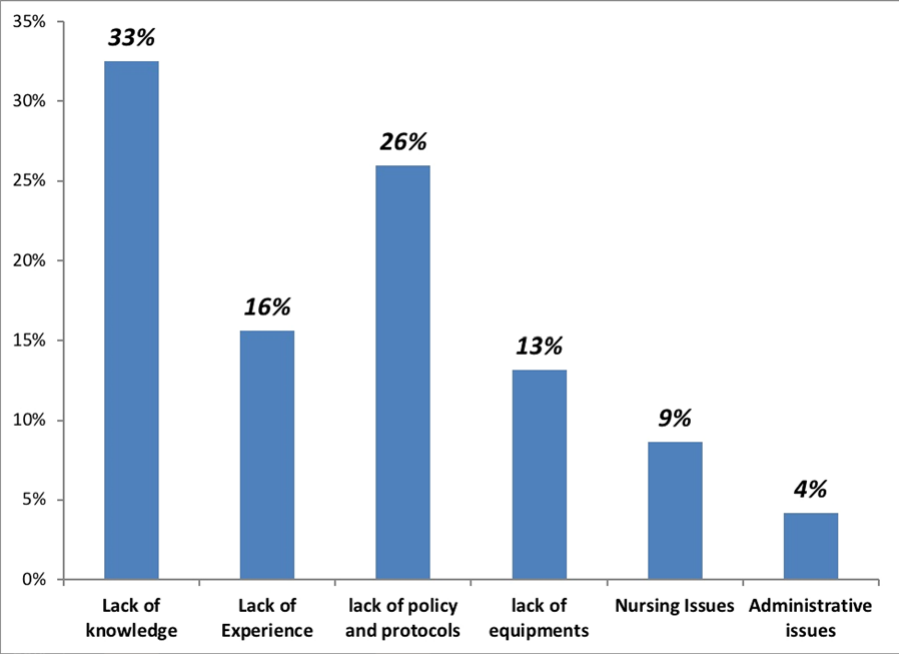
**Fig. 1 (abstract P236)**. Barriers for utilization about targeted temperature management and therapeutic hypothermia

## P237

### Neuron specific enolase implementation and practical aspect as a neuroprognostication predictor in patients post ROSC of OOHCA in William Harvey Hospital ITU

#### D Pandit, S Mohammed

##### William Harvey Hospital, Intensive Care Unit, Ashford, UK

*Critical Care* 2023, **27(S1)**: P237

**Introduction:** Hypoxic brain injury is the most serious complication of OOHCA, NSE had been implemented in WHH ITU as one of the Neuro-prognostication predictor of hypoxic brain damage in addition to clinical examination, MRI EEG and SSEP.

**Methods:** A total of 92 patients had been admitted to ITU in WHH from January 2021 till April 2022. The aim of the project is to check practicality and usefulness of using NSE as a marker of hypoxic brain damage in WHH ITU. Inclusion criteria included 1) patient post ROSC from OOHCA, 2) NSE sample taken at 48 h and 72 h exclusion criteria included with at least one result reported 1) In-hospital cardiac arrest 2) patients with sample hemolysed.

**Results:** NSE levels were significant (more than 60 ug/l) in one or 2 readings, OOHCA 25 patients. 24 patients died, one patient lived with residual cognitive impairment and evidence of hypoxic brain damage in MRI. 2 patients withdrawn from treatment because of multiorgan failure and pneumonia respectively. 22 patients 91.6 withdrawn from treatment because of hypoxic brain injury. 18 patients in addition to high NSE they had MRI, EEG and SSEPs finding consistent with the diagnosis of hypoxic brain injury, 4 patients (18.1%) no MRI was performed due to haemodynamic instability and PPM. In the other arm where total of 46. patients, 13 patient died (28% mortality), 31 patient had a complete neurological recovery and 2 patient had evidence of hypoxic brain damage in MRI and clinically.

**Conclusions:** All patient with significant high NSE values, had evidence of hypoxic brain damage in at leat of one of the other marker, it showed huge values for patient who were not able to be shifted to MRI room, the absence of significant values of NSE does not exclude the presence of hypoxic brain injury and the diagnosis should be taken based on clinical assessment in addition to other predictors.

## P238

### Predicting early perioperative stroke after cardiothoracic surgery through assessment of intraoperative regional cerebral oxygenation in affected versus non-affected hemispheres using near-infrared spectroscopy

#### R Pierik^1^, TWL Scheeren^2^, ME Erasmus^3^, WM Van den Bergh^1^

##### ^1^University Medical Center Groningen, Critical Care, Groningen, Netherlands, ^2^University Medical Center Groningen, Anesthesiology, Groningen, Netherlands, ^3^University Medical Center Groningen, Cardiac Surgery, Groningen, Netherlands

*Critical Care* 2023, **27(S1)**: P238

**Introduction:** Patients undergoing cardiothoracic surgery are at risk of developing perioperative stroke. Residual effects of anesthesia or sedation may hamper timely detection [1]. This study aims to evaluate the value of intraoperative regional cerebral oxygenation (ScO_2_) monitoring with near-infrared spectroscopy (NIRS) for predicting the occurrence of early perioperative stroke within three days after cardiothoracic surgery.

**Methods: ** We performed a retrospective observational cohort study including all consecutive cardiothoracic surgery patients with routinely perioperative ScO_2_ monitoring admitted postoperatively to the Intensive Care Unit (ICU) of the University Medical Center Groningen between 2008 and 2017. Brain images were studied. Patients with a confirmed stroke in the anterior cerebral circulation were included in the analysis. The cumulative area under the curve (AUC), duration, and the number of ScO_2_ excursions below predefined thresholds (an absolute value of 50% ScO_2_ or a relative reduction of 20% below baseline ScO_2_) were calculated per hemisphere. The intraoperative ScO_2_ trajectory of hemispheres affected by stroke was compared with non-affected hemispheres using logistic regression analyses to evaluate the association between ScO_2_ values and stroke.

**Results:**Of all included 2454 patients, 39 had a confirmed anterior stroke on brain imaging, of which five had a bilateral stroke resulting in 44 affected hemispheres which were compared to 34 non-affected hemispheres. The AUC (OR 0.99; 95% CI 0.97–1.01), duration (OR 0.99; 95% CI 0.98–1.00), and number (OR 0.99; 95% CI 0.79–1.23) of ScO_2_ excursions below predefined thresholds were not significantly different in affected hemispheres compared to non-affected hemispheres (Fig. 1).

**Conclusions:** Low ScO_2_ levels during cardiothoracic surgery could not predict the occurrence of an early perioperative stroke within three days after surgery.


**Reference**
Vlisides P et al. Can J Anesth. 2016;63:193–204.

**Figure Figcu:**
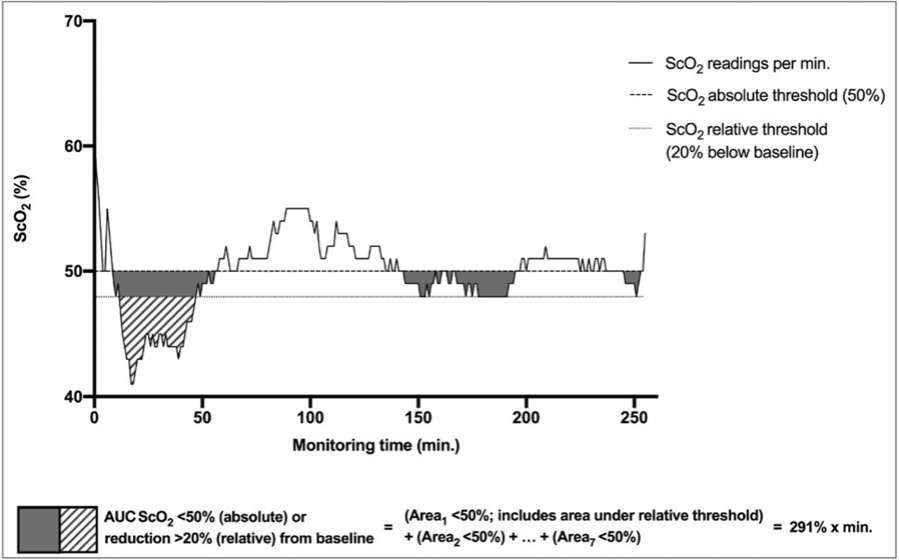
**Fig. 1 (abstract P238)**. Calculation for the area under the curve (AUC) based on intraoperative ScO_2_: representative example of a patient with thromboembolic stroke (anterior circulation)

## P239

### Near-infrared spectroscopy and processed electroencephalogram monitoring for predicting perioperative stroke risk in cardiothoracic surgery: an observational cohort study

#### R Pierik^1^, TWL Scheeren^2^, ME Erasmus^3^, WM Van den Bergh^1^

##### ^1^University Medical Center Groningen, Critical Care, Groningen, Netherlands, ^2^University Medical Center Groningen, Anesthesiology, Groningen, Netherlands, ^3^University Medical Center Groningen, Cardiac Surgery, Groningen, Netherlands

*Critical Care* 2023, **27(S1)**: P239

**Introduction:** Stroke is a feared complication after cardiothoracic surgery with an incidence of around 2–3%. Anaesthesia and postoperative sedation may obscure clinical symptoms of stroke and thus delay diagnosis and timely intervention [1]. The objective was to assess the value of intraoperative neuromonitoring and blood pressure monitoring for predicting the occurrence of perioperative stroke within three days after cardiothoracic surgery.

**Methods:** We performed a single-centre retrospective observational cohort study at the University Medical Center Groningen. All consecutive patients with cardiothoracic surgery and intraoperative neuromonitoring admitted postoperatively to the Intensive Care Unit (ICU) between 2008 and 2017 were included. The primary endpoint was the occurrence of any stroke within three days post-cardiothoracic surgery. Brain imaging was studied. Areas under the curve (AUC) of intraoperative mean arterial pressure, cerebral oxygen saturation (ScO_2_), and bispectral index (BIS) below predefined thresholds were calculated, and the association with early stroke was tested using logistic regression analyses.

**Results:** Of all 2454 included patients, 58 (2.4%) had a confirmed stroke. In univariate analysis, a larger AUC MAP < 60 mmHg and AUC BIS < 25 were associated with the occurrence of postoperative stroke while ScO_2_ < 50% or > 20% reduction from individual baseline was not (Table 1). After adjustment for MAP, ScO_2_, duration of surgery, and other relevant baseline characteristics, only AUC BIS < 25 remained independently associated with stroke occurrence.

**Conclusions:** Cumulative BIS below 25 was associated with the occurrence of a perioperative stroke within three days after cardiothoracic surgery. If avoiding BIS values below 25 during cardiothoracic surgery will reduce perioperative stroke incidence should be assessed in future prospective studies.


**Reference**
Gaudino M et al. J Am Heart Assoc. 2019;8:

**Table Tabca:** **Table 1 (P239)**. Results

Variable	All stroke	All stroke	TE stroke	TE stroke
	Univariate	Multivariable (b)/Multivariable (c)	Univariate	Multivariable (b)
	OR (95% CI)	OR (95% CI)	OR (95% CI)	OR (95% CI)
AUC MAP < 60 (mmHg) (a)	1.43 (1.21–1.68)	1.16 (0.80–1.70)	1.19 (0.91–1.55)	1.21 (0.72–2.04)
AUC ScO2 < 50% or reduction > 20% from baseline (a)	0.91 (0.50–1.67)	0.63 (0.24–1.68)	0.57 (0.13–2.49)	0.62 (0.16–2.40)
AUC BIS < 25 (index) (a)	1.51 (1.24–1.83)	1.43 (1.03–2.00)/1.45 (1.12–1.87)	1.17 (0.81–1.70)	1.27 (0.76–2.12)
Duration of surgery (hour)	1.27 (1.13–1.43)	1.00 (0.76–1.31)	1.18 (1.01–1.38)	0.91 (0.63–1.31)

TE = thromboembolic; AUC = area under the curve; MAP = mean arterial pressure; ScO_2_ = (regional) cerebral saturation; BIS = bispectral index. (a) Due to undescriptive ORs (1.00 (95% CI 1.00–1.00)) caused by high dispersions within the study group, corresponding units were assessed by 1000 × min. increase. (b) Adjusted for duration of surgery and intraoperative neuromonitoring (AUC MAP < 60 mmHg, AUC ScO2 < 50% (absolute) or reduction > 20% (relative) from baseline, AUC BIS < 25). (c) Adjusted for age, sex, isolated CABG (vs. other cardiothoracic surgeries), duration of surgery, diabetes mellitus, history of AF, and presence of other cardiovascular risk factors (carotid stenosis, PVD, hypercholesterolemia)

## P240

### The slope of cerebral oxyhemoglobin oscillation is related to improvement of cerebral perfusion after revascularization therapy in patients with symptomatic extracranial internal carotid artery: a case series study

#### S Kim^1^, TJ Kim^1^, SH Park^2^, SB Ko^1^

##### ^1^Seoul National University Hospital, Neurology and Critical Care Medicine, Seoul, South Korea, ^2^Inha University Hospital, Neurology and Critical Care Medicine, Incheon, South Korea

*Critical Care* 2023, **27(S1)**: P240

**Introduction:** Cerebral hypoperfusion shows a detrimental effect on cerebral ischemia in patients with symptomatic high-grade extracranial internal carotid artery (ICA) stenosis. Revascularization therapy including carotid endarterectomy (CEA) carotid artery stenting (CAS) is required to prevent the risk of stroke. In this study, we investigated the change in prefrontal oxyhemoglobin oscillation (ΔHbO_2_,) is associated with the perfusion status before and after revascularization therapy in symptomatic severe extracranial ICA stenosis patients.

**Methods:** We included eight patients with symptomatic severe extracranial ICA stenosis who underwent revascularization therapy such as CEA and CAS between July 2016 and August 2019. HbO2 data were measured using functional near-infrared spectroscopy before and after revascularization therapy. The slope of ΔHbO_2_ and the ipsilateral/contralateral slope ratio of ΔHbO_2_ were analyzed before and after treatment.

**Results:** Among included patients (mean age, 68.8 years, male 87.5%), ICA stenosis was found in 50% (n = 4) on the right side and 50% (n = 4) on the left side. The majority of carotid revascularization was performed by CAS (CAS, 75.0% vs CEA, 25.0%). The ipsilateral to contralateral ΔHbO_2_ slope ratio significantly decreased after revascularization procedures (mean ± standard deviation, before treatment, 1.38 ± 0.42 vs after treatment 0.96 ± 0.24, Wilcoxon signed rank test, *p* = 0.036). Moreover, follow-up perfusion imaging showed the vascular reserve was improved on the side of revascularization therapy.

**Conclusions:** This study demonstrated that the ipsilateral to contralateral ΔHbO_2_ slope ratio decreased after the successful revascularization in patients with symptomatic severe extracranial ICA stenosis. The ipsilateral/contralateral ΔHbO_2_ ratio could be used as a marker of change in vascular reserve capacity after revascularization treatment in patients with large artery steno-occlusion.

## P241

Withdrawn.

## P242

### C-terminal pro-arginine vasopressin, but not D-dimer is associated with poor functional outcome in critically ill patients with an aneurysmal subarachnoid hemorrhage

#### JAH Van oers^1^, D Ramnarain^1^, A Oldenbeuving^1^, Y Kluiters^2^

##### ^1^Elisabeth Tweesteden Ziekenhuis, Intensive Care Unit, Tilburg, Netherlands, ^2^Elisabeth Tweesteden Ziekenhuis, Clinical Chemistry, Tilburg, Netherlands

*Critical Care* 2023, **27(S1)**: P242

**Introduction:** We assessed the ability of C-terminal pro-arginine vasopressin (CT-proAVP) and D-dimer to predict one-year poor functional outcome (Glasgow Outcome Scale score 1–3) in critically ill patients with an aneurysmal subarachnoid hemorrhage (aSAH).

**Methods:** Plasma CT-proAVP and D-dimer were collected upon admission in this single-centre prospective observational cohort study. Biomarkers were tested in a multivariable logistic regression model adjusted for World Federation of Neurological Surgeons (WFNS) score and modified Fisher scale.

**Results:** In 100 aSAH patients, median CT-proAVP level was 24.9 pmol/l (interquartile range (IQR) 11.5–53.8) and median D-dimer was 1503 µg/L (IQR 668–3711). Forty-five patients had one-year poor functional outcome. Receiver-operating characteristics curves (ROC) revealed high accuracy for CT-proAVP, but not for D-dimer to identify patients with one-year poor functional outcome (CT-proAVP: Area under the curve (AUC) 0.84, 95% confidence interval (CI) 0.80–0.92, *p* < 0.001 and D-dimer: AUC 0.63, 95% CI 0.52–0.74, *p* 0.026) (Fig. 1). CT-proAVP was a significant predictor for 1-year poor functional outcome (unadjusted odds ratio (OR) 1.05, 95% CI 1.03–1.08, *p* < 0.001, adjusted OR 1.05, 95% CI 1.02–1.08, *p* < 0.001). D-dimer was not a significant predictor for one-year poor functional outcome (both unadjusted and adjusted p 0.121 and *p* = 0.708).

**Conclusions:** CT-proAVP, but not D-dimer had good ability to predict poor functional outcome in this cohort of critically ill aSAH patients, suggesting that activation of the pituitary-adrenal axis is more important for prognosis than activation of coagulation and fibrinolysis.

**Figure Figcv:**
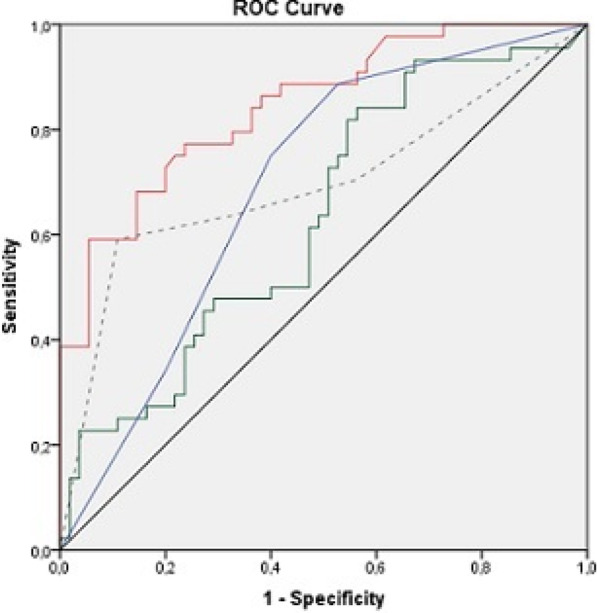
**Fig. 1 (abstract P242)**. Receiver-operating characteristics curves (ROC). Red line: CT-proAVP (AUC 0.84 (95% CI 0.80–0.92), *p* < 0.001), green line: D-dimer (AUC 0.63 (95% CI 0.52–0.74), *p* 0.026), blue line: Modified Fisher score (AUC 0.69 (95% CI 0.59–0.81), p 0.001), grey dotted line: WFNS score (AUC 0.70 (95% CI 0.59–0.80), *p* 0.001), black line: reference line

## P243

### Accuracy assessment of sonographic methods and pupillometry to detect intracranial hypertension

#### A Blandino Ortiz^1^, M Lopez Olivencia^2^, J Higuera Lucas^2^, E Morales Sorribas^2^, C Soriano Cuesta^2^, S Saez Noguero^2^, GL Alonso Salinas^3^, S Garcia Plaza^2^, R De Pablo^2^

##### ^1^Hospital Universitario Ramón y Cajal, Department of Intensive Care, Madrid, Spain, ^2^Hospital Universitario Ramón y Cajal, Intensive Care Department, Madrid, Spain, ^3^Hospital Universitario de Navarra, Intensive Care Department, Pamplona, Spain

*Critical Care* 2023, **27(S1)**: P243

**Introduction:** In the last decade, several methods are available to allow for the non-invasive assessment of intracranial pressure, mostly sonographic, with the implementation of a validated formula to estimate the cerebral perfusion pressure [1,2]. Similar approach has being extended to the automated pupillometry. The aim of this work is to assess the accuracy of sonographic and pupillometry to detect intracranial hypertension.

**Methods:** We prospectively recorded consecutive neurocritically ill patients data with ICP measurements, sonographic variables to calculate ICP with differente methos (nICP, Pulsatility index, optic nerve sheath diameter) and pupillometry, allong with clinically relevant variables. Transcranial Doppler was performed by 2 investigators, which recorded the different methods at the same time, includind ICP, n-CPP (systolic and diastolic flows), MAP, optic nerve sheath diameter and pupillometry.

**Results:** From May to October 2022, we included 13 patients, in whom we recorded 33 measurements of standard ICP, transcranial Doppler, optic nerve sheath diameter, pupillometry, hemodynamic and clinical variables. The median age of the patients was 53.8 (± 7.35); mostly male patients (female 24.24%). The most frequent diagnosis of the patients who were included was intracranial hemorrhage (51.52%). The ICP was measured with external ventricular drainage in 81,82% of the cases. We found a statistically significant (*p* < 0.001) and strong correlation (coef. 0.8954) between ICP and non-invasive ICP (n-ICP) measured by transcranial Doppler (Fig. 1). Most relevant variables in the Kappa index were n-ICP (AUC = 0.09950) and the NPi (AUC = 0.9999).

**Conclusions:** Among neurocritically ill patients with ICP monitoring, the non-invasive assessment of ICP using transcranial Doppler seems to be a reliable and reasonably accurate alternative method.


**References**
Robba C et al. Crit Care 2020;24:379.Czosnyka M et al. J Neurosurg. 1998;88:802–8.

**Figure Figcw:**
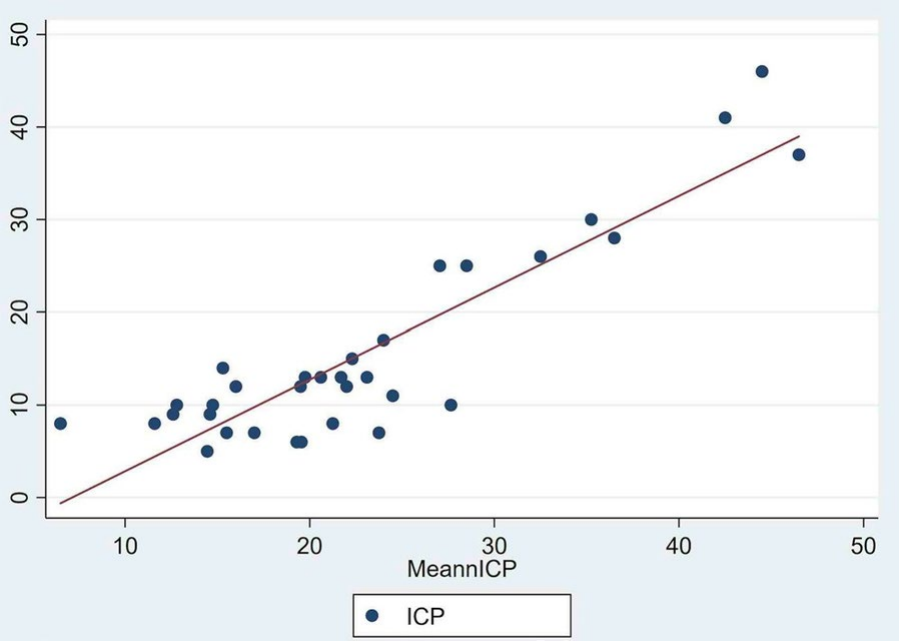
**Fig. 1 (abstract P243)**. Correlation ICP & nICP

## P244

### Combined effects of α7 nicotinic acetylcholine receptor allosteric modulation and environmental enrichment on sustained attention and cholinergic transmission after experimental brain trauma

#### CB Bondi, EH Moschonas, NS Race, PL Rennerfeldt, JP Cheng, TS Ranellone, EM Annas, AE Kline

##### University of Pittsburgh, PMR, Pittsburgh, USA

*Critical Care* 2023, **27(S1)**: P244

**Introduction:** Traumatic brain injury (TBI) is a leading cause of cognitive disability. Combining a pharmacotherapy with enriched environment (EE) housing may be efficient for cognitive recovery. We predicted that chronic NS-1738, an α7 nicotinic acetylcholine (ACh) receptor (α7-nAChR) positive allosteric modulator, will improve sustained attention post-TBI, alone and in combination with EE. Blocking α7-NAChRs with methylycaconitine (MLA) will attenuate the beneficial effects of NS-1738, confirming its mechanism of action.

**Methods:** Adult male rats were trained in the 3-choice serial reaction time task, reaching stable baselines prior to moderate controlled cortical impact (CCI) or sham injury. Rats were randomized to NS-1738 (3 mg/kg) or vehicle (saline) starting 24 h post-injury and given daily [subacute (7d); chronic (28d)]. The chronic paradigm co-investigated daily EE (6 h/d), and subgroups were also subjected to daily α7-NAChRs blockade via MLA (3 mg/kg). 3-CSRT retest occurred on days 14–24. Medial prefrontal cortex (mPFC) Western blots assessed cholinergic markers [acetylcholinesterase (AChE), choline acetyltransferase (ChAT), and α7-nAChR]. Microarray analysis examined serum inflammatory gene expression. Statistical analysis utilized ANOVAs with Newman-Keuls post hoc tests.

**Results:** TBI rats exhibited impaired sustained attention (*p* < 0.05), which was improved by chronic NS-1738 (*p* < 0.05), but not by subacute NS-1738 (*p* > 0.05). NS-1738 + EE rendered an additive effect on lowering omissions and improving inflammatory markers (*p* < 0.05) including TREM-1 and IL-1 RA. TBI decreased mPFC ChAT and AChE (*p* < 0.05) with partial restoration by subacute and chronic NS-1738. TBI reduced ChAT + cells in the nucleus basalis of Meynert, effect attenuated by EE, alone or combined with chronic NS-1738 (*p* < 0.05). TBI groups that received MLA demonstrated a reinstatement of performance deficits, as hypothesized.

**Conclusions:** Our findings suggest enhancing cholinergic transmission after TBI may be beneficial for neurobehavioral recovery.

## P245

### Ionized calcium level and neurological outcome of patients with isolated severe traumatic brain injury

#### K Badarni^1^, N Harush^2^, E Andrawus^3^, H Bahouth^3^, Y Bar-Lavie^3^, A Raz^3^, M Roimi^3^, D Epstein^1^

##### ^1^Rambam Health Care Campus, Critical Care Division, Haifa, Israel, ^2^Ruth and Bruce Rappaport Faculty of Medicine, Technion, Haifa, Israel, ^3^Rambam Health Care Campus, Haifa, Israel

*Critical Care* 2023, **27(S1)**: P245

**Introduction:** Traumatic brain injury (TBI) is a leading cause of death and disability worldwide. Ionized calcium (Ca^++^) is an essential co-factor in the coagulation cascade and platelet aggregation and hypocalcemia may contribute to the progression of intracranial bleeding. On the other hand, Ca^++^ is an important mediator of cell damage after TBI and cellular hypocalcemia may have a neuroprotective effect after brain injury. In this study, we aimed to evaluate the relationship between admission Ca^++^ levels and neurological outcomes of patients suffering from isolated severe TBI.

**Methods:** This was a retrospective single-center cohort study of all patients admitted due to isolated severe TBI between January 2014 and December 2020. The primary outcome of this study was a favorable neurological status at discharge, defined by modified Rankin Scale (mRS) of 0–2. Multivariable logistic regression was performed to determine whether Ca^++^ was an independent predictor of neurological status at discharge.

**Results:** 201 patients were included. Hypocalcemia was common among patients with isolated severe TBI (73.1%). Most of the patients had modest hypocalcemia, and only 13 (6.5%) patients had Ca^++^  ≤ 1.00 mmol/l. Hypocalcemia was independently associated with higher rates of good neurological status at discharge with an adjusted OR of 3.03 (95% CI 1.11–8.33), *p* = 0.03. In the subgroup of 81 patients with GCS > 8 at admission, 52 (64.2%) had hypocalcemia. Good neurological status at discharge was recorded in 28 (53.8%) of hypocalcemic patients vs. 17.2% of those with normal Ca^++^ (*p* = 0.002) (Fig. 1). In multivariate analyses, hypocalcemia was independently associated with good neurological status at discharge (adjusted OR of 6.67, 95% CI 1.39–33.33, *p* = 0.02).

**Conclusions:** Among trauma patients with isolated severe TBI, mild admission hypocalcemia is associated with better neurological status at hospital discharge. The prognostic value of Ca^++^ may be greater among those with admission Glasgow Coma Scale > 8.

**Figure Figcx:**
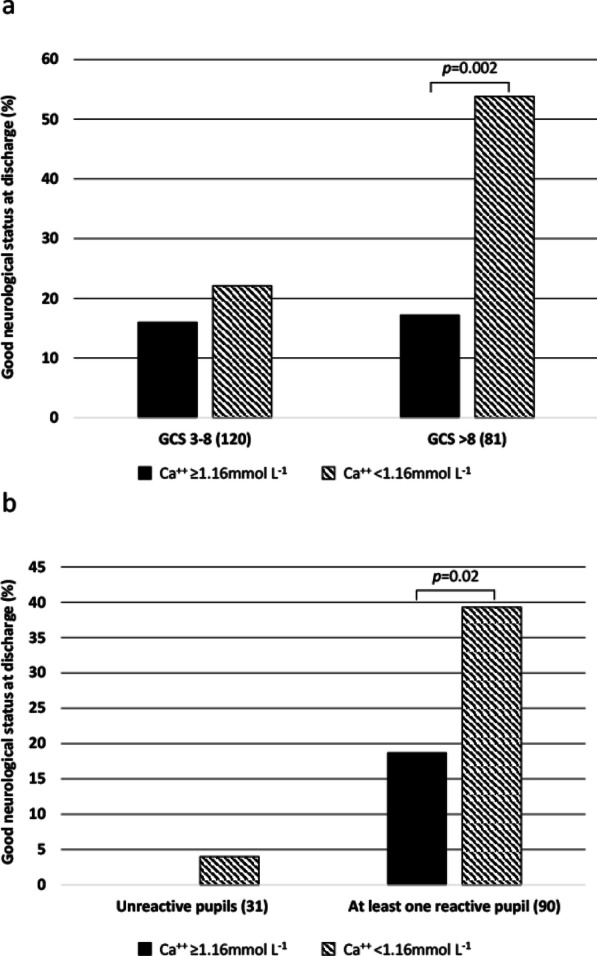
**Fig. 1 (abstract P245)**. Relationships between neurological outcome at discharge, ionized calcium level, Glasgow Coma Scale (**a**), and pupillary responses (**b**). The number of patients in each category is shown in parentheses

## P246

### Brain injury and ketamine, a prospective randomized controlled double blind clinical trial to study the efficacy and safety of ketamine in brain injured patients.

#### V De Sloovere^1^, L Mebis^2^, P Wouters^2^, F Guïza^2^, G Meyfroidt^2^

##### ^1^University Hospitals Leuven, Department of Anesthesiology, Leuven, Belgium, ^2^University Hospitals Leuven, Department and Laboratory of Intensive Care Medicine, Leuven, Belgium

*Critical Care* 2023, **27(S1)**: P246

**Introduction:** In recent decades, ketamine has been increasingly used in traumatic brain injury (TBI) as an adjunct to standard sedation, without randomised clinical trials to support this practice. Contrary to previous reports [1], a meta-analysis suggested that ketamine, when administered to sedated and ventilated TBI patients, does not increase ICP [2]. The Brain Injury and Ketamine (BIKe) study is a prospective, double-blind, randomised phase 4 clinical trial investigating the efficacy and safety of ketamine as an adjunct to a standard sedation regimen in severe TBI.

**Methods:** Adult TBI patients, requiring sedation and intracranial pressure (ICP) monitoring, are eligible for inclusion. Exclusion criteria are: imminent death, therapy limitation at screening and pre-existing neurocognitive impairment. One hundred patients will be recruited in 7 Belgian hospitals and randomised to placebo or ketamine at a dose of 1 mg/kg/h, in addition to standard sedatives. The study drug will be stopped along with the last sedative for ICP control. The primary efficacy endpoint, is the effect on the therapy intensity score (TIL), the safety endpoint is measured by the number of episodes of intracranial hypertension. A decrease of 3 points in the TIL score and an increase of 2 episodes in the median number of episodes of high ICP per ICU stay from baseline are considered clinically relevant. Secondary endpoints are the Glasgow Outcome Score determined after 6 months, doses of sedatives, vasopressors and inotropic agents, incidence of decompressive craniectomy, propofol infusion syndrome, barbiturate coma, and delirium. (ClinicalTrials.gov Identifier: NCT05097261).

**Results:** Recruitment in all 7 hospitals has started.

**Conclusions:** To our knowledge, this is the first randomised controlled double-blind study of the efficacy and safety of ketamine versus placebo as an adjunct to a standard sedation regimen in TBI patients.

## References


Shapiro HM et al. Br J Anaesth. 1972;44:1200–4.Zeiler FA et al. Neurocrit Care. 2014;21:163–73.

## P247

### Determining the relative contribution of physiological parameters to variance in cerebral oxygenation in the critically ill

#### N Cody^1^, I Bradbury^2^, R McMullan^3^, G Quinn^4^, A O’Neill^4^, K Ward^4^, J McCann^4^, D McAuley^3^, J Silversides^3^

##### ^1^Wellcome Wolfson Institute of Experiment Medicine, Queen’s University Belfast, Belfast, UK, ^2^Independent Consulting Statistician, Aviemore, UK, ^3^Wellcome Wolfson Institute of Experiment Medicine, Belfast, UK, ^4^Regional ICU, Royal Victoria Hospital, Belfast, UK

*Critical Care* 2023, **27(S1)**: P247

**Introduction:** Near infra-red spectroscopy (NIRS) is a non-invasive method of monitoring cerebral oxygenation and widely used in cardiac anaesthesia. In ICU, NIRS could be an attractive means to monitor adequacy of oxygen delivery to the brain, but little is known about the determinants of cerebral oxygen saturation in this population. The aim of this study is to assess the contribution of commonly measured physiological parameters to cerebral oxygen saturation in a general ICU population.

**Methods:** In a subset of patients participating in a randomised control trial of conservative fluid management and deresuscitation versus usual care [1], a NIRS monitor was attached to the forehead and cerebral saturation (rSO_2_) recorded continuously. Values were compressed to means for each minute. The mean arterial pressure (MAP), heart rate (HR) and pulse oximetry (SpO_2_) were recorded simultaneously. Arterial PaCO_2_, PaO_2_ and haemoglobin concentration were measured 12-hourly. Linear mixed models with random subject effects were used to determine the association between these potential physiological determinants and rSO_2_, with age as a covariate.

**Results:** 59 patients were included in analysis. Linear mixed effect models demonstrated that the selected physiological variables could explain a small proportion of variance of rSO_2_ (R^2^ 0.17, *p* < 0.001). Upon final model selection (Table 1), PaCO_2_, SpO_2_, HR and allocation to conservative fluid were all significantly associated with rSO_2_. Altogether these parameters explained just over 10% of variation in cerebral saturation (R^2^ 0.11, *p* < 0.001).

**Conclusions:** Our findings demonstrate that in this cohort of critically ill patients, only a small proportion of variance in NIRS derived cerebral oximetry can be explained by commonly measured physiological variables. We conclude that for NIRS to be a useful monitoring modality in general critical care, further research is required to understand its physiological determinants.


**Reference**
Silversides JA et al. Intensive Care Med. 2022;48:190–200.

**Table Tabcb:** **Table 1 (abstract P247)**. Final linear mixed effect model of rSO_2_

Covariate	Coefficient	Standard error	*p* value
Intercept	50.98	4.10	< 0.001
HR (bpm)	0.036	0.003	< 0.001
SpO_2_ (%)	0.098	0.020	< 0.001
PCO_2_ (Kpa)	1.547	0.623	0.016
Conservative fluid	−3.761	1.546	0.01

rSO_2_ = Intercept + HR + SpO_2_ + PCO_2_ + Cons Fluid

## P248

Withdrawn.

## P249

### Implementation of FASTHUGSBID checklist improves clinical outcome in trauma intensive care unit

#### D Seo, K Jung

##### Ajou University Hospital, Department of Trauma Surgery, Suwon-si, South Korea

*Critical Care* 2023, **27(S1)**: P249

**Introduction:** The aim of this study was to evaluate the effectiveness of the FASTHUGSBID checklist (feeding, analgesia, sedation, thromboembolic prophylaxis, head of bed elevation, ulcer prophylaxis, glycemic control, spontaneous breathing trial, bowel movement, indwelling catheter, drug de-escalation) [1, 2] in trauma intensive care unit at a single trauma center in Korea.

**Methods:** Trauma patients between 2016 and 2020 at our trauma center were analyzed retrospectively. It was divided into two groups: before implementation of FASTHUGSBID group, from 2016 to 2017 and after implementation of FASTHUGBSBID group, from 2019 to 2020. We analyzed participant characteristics and in-hospital mortality, ICU and hospital length of stay between two groups. Kaplan–Meier and logistic regression was used to perform the survival analysis and multiple linear regression was used to identify factors independently associated with the ICU and hospital length of stays. To analyze the effectiveness of checklist in detail, we compared each components of FASTHUGSBID checklist with two groups.

**Results:** After implementation of FASTHUGSBID group had lower in-hospital mortality, shorter ICU and hospital length of stay (*p* < 0.05). In multivariable analysis, FASTHUGSBID checklist were an independent factor for reducing ICU, hospital length of stay and in-hospital mortality (*p* < 0.05). In the analysis comparing each components of FASTHUGSBID, There were several positive effects: early start of enteral nutrition, decrease benzodiazepine and opioid use, increase dexmedetomidine and propofol use, increase enoxaparin use, catheter removal early, decrease antimicrobials use (*p* < 0.05).

**Conclusions:** Implementation of FASTHUGSBID checklist reduce the ICU, hospital length of stay and in-hospital mortality (Table 1) because of better effect of each components of this checklist in trauma patients.


**References**
Vincent JL. Crit Care Med 2005;33:1225–9.Vincent WR et al. Crit Care Med 2009;37:2326–7.

**Table Tabce:** **Table 1 (abstract P249)**. Comparison of primary outcome, pre- and post-FASTHUGSBID group

Primary outcome	Pre-FASTHUGSBID (n = 696)	Post-FASTHUGSBID (n = 1720)	*p*
In-hospital mortality (%)	8.3	4.8	< 0.05
Complications (%)	23.0	16.5	< 0.05
ICU length of stay (d),mean ± SD	7.8 ± 13.3	5.1 ± 10.4	< 0.05
Hospital length of stay (d),mean ± SD	24.3 ± 24.6	17.6 ± 16.0	< 0.05
Duration of mechanical ventilation (d),mean ± SD	9.2 ± 13.3 (n = 315)	5.0 ± 8.4 (n = 682)	< 0.05

## P250

### Topical anesthesia for resuscitative TEE on awake trauma patients in emergency department

#### AH Ahmad^1^, O Adi^2^, R Sallehuddin^3^, C Pei Fong^2^

##### ^1^Raja Permaisuri Bainun Hospital, Emergency Department, Perak, Malaysia, ^2^Raja Permaisuri Bainun Hospital, Emergency and Trauma Department, Perak, Malaysia, ^3^Sultanah Bahiyah Hospotal, Emergency and Trauma Department, Alor Setar, Malaysia

*Critical Care* 2023, **27(S1)**: P250

**Introduction:** Resuscitative transesophageal echocardiography (TEE) has been effectively used in haemodynamic instability or cardiopulmonary arrest [1,2]. It is a challenge to perform TEE in traumatic patients due to cervical immobilisation and related injuries. The improper anaesthesia in airway or maxillofacial injuries, cardiac and lung contusion may compromised the airway and ventilation. This study aims to review the usage of topical anaesthetic techniques for TEE in awake trauma patients.

**Methods:** We report a case series of performing topical anaesthesia for TEE insertion in suspected blunt aortic injury patients. The inclusion criteria: non-intubated, > 18 years old, GCS > 13. The exclusion criteria: moderate or severe brain injury, airway obstruction, stridor, suspected oesophageal,cervical and neck injury, oxygen requirement > 5 l/min. The complications during and after were documented. The patients were put on a nasal cannula 3L/min during the procedure. Topical lidocaine spray (10%, 20 puffs max 4 mg/kg) is applied to the pharynx. The flexible endoscopic TEE probe is passed once the patient felt numbness over the throat or loss of gag reflex. The procedure is performed by a certified TEE trained Emergency Physician.

**Results:** Twelve trauma patients were included. The average age was 52 years old (min = 38, max = 62). Average sedation time was 6 min 13 s (max = 10 min 1 s, min 0 min). TEE procedures were completed in 5 min 19 s (max = 8 min 6 s, min = 3 min 2 s). Recovery time was immediate. All patients remained haemodynamically stable. There was a single minor complication recorded (throat discomfort). One patient was unable to complete the procedure forcing awake sedation. There is no TEE related complication.

**Conclusions:** The topical anaesthesia is a good technique for a mild head injury patient to undergo a safe TEE procedure. The probe can be placed without distress with good mucosal anaesthesia while maintaining the airway and preventing major complications.


**References**
Vignon P et al. Circulation. 1995;92:2959–2968.Hahn RT et al. J Am Soc Echocardiogry. 2013;26:921–64.


## P251

### Outcomes of basic versus advanced prehospital life support in severe pediatric trauma

#### D Epstein^1^, S Goldman^2^, I Radomislensky^2^, IT Group^2^, K Badarni^3^, A Raz^3^, AM Lipsky^4^, S Lin^3^, M Bodas^2^

##### ^1^Rambam Health Care Campus, Critical Care Division, Haifa, Israel, ^2^Israel National Center for Trauma and Emergency Medicine Research, Gertner Institute for Epidemiology and Health Policy Research, Sheba Medical Center, Tel-Hashomer, Israel, ^3^Rambam Health Care Campus, Haifa, Israel, ^4^Emergency Department, Emek Medical Center, Afula, Israel

*Critical Care* 2023, **27(S1)**: P251

**Introduction:** The role of basic life support (BLS) versus advanced life support (ALS) in pediatric trauma is controversial. Although ALS is widely accepted as the gold standard, previous studies have found no advantage of ALS over BLS care in adult trauma. The objective of this study was to evaluate whether ALS transport confers a survival advantage over BLS among severely injured children.

**Methods:** A retrospective cohort study of data included in the Israeli National Trauma Registry from January 1, 2011, through December 31, 2020 was conducted. All the severely injured children (age < 18 years and injury severity score [ISS] ≥ 16) were included. Patient survival by mode of transport was analyzed using logistic regression.

**Results:** Of 3167 patients included in the study, 65.1% were transported by ALS and 34.9% by BLS. Significantly more patients transported by ALS had ISS ≥ 25 as well as abnormal vital signs at admission. The ALS and BLS cohorts were comparable in age, gender, mechanism of injury, and prehospital time. Children transported by ALS had higher in-hospital mortality (9.2% vs. 0.9%, *p* < 0.001). Following risk adjustment, patients transported by ALS teams were significantly more likely to die than patients transported by BLS (adjusted OR 2.27, 95% CI 1.05–5.41, *p* = 0.04). Patients with ISS ≥ 50 had comparable mortality rates in both groups (45.9% vs. 55.6%, *p* = 0.837) (Fig. 1) while patients with GCS < 9 transported by ALS had a higher mortality (25.9% vs. 11.5%, *p* = 0.019). Admission to a level II trauma center vs. a level I hospital was also associated with increased mortality (adjusted OR 2.78 (95% CI 1.75–4.55, *p* < 0.001).

**Conclusions:** Among severely injured children, prehospital ALS care was not associated with lower mortality rates relative to BLS measures. Rapid transport to definitive surgical care in high-volume trauma centers with only BLS-level interventions may result in improved survival.

**Figure Figcy:**
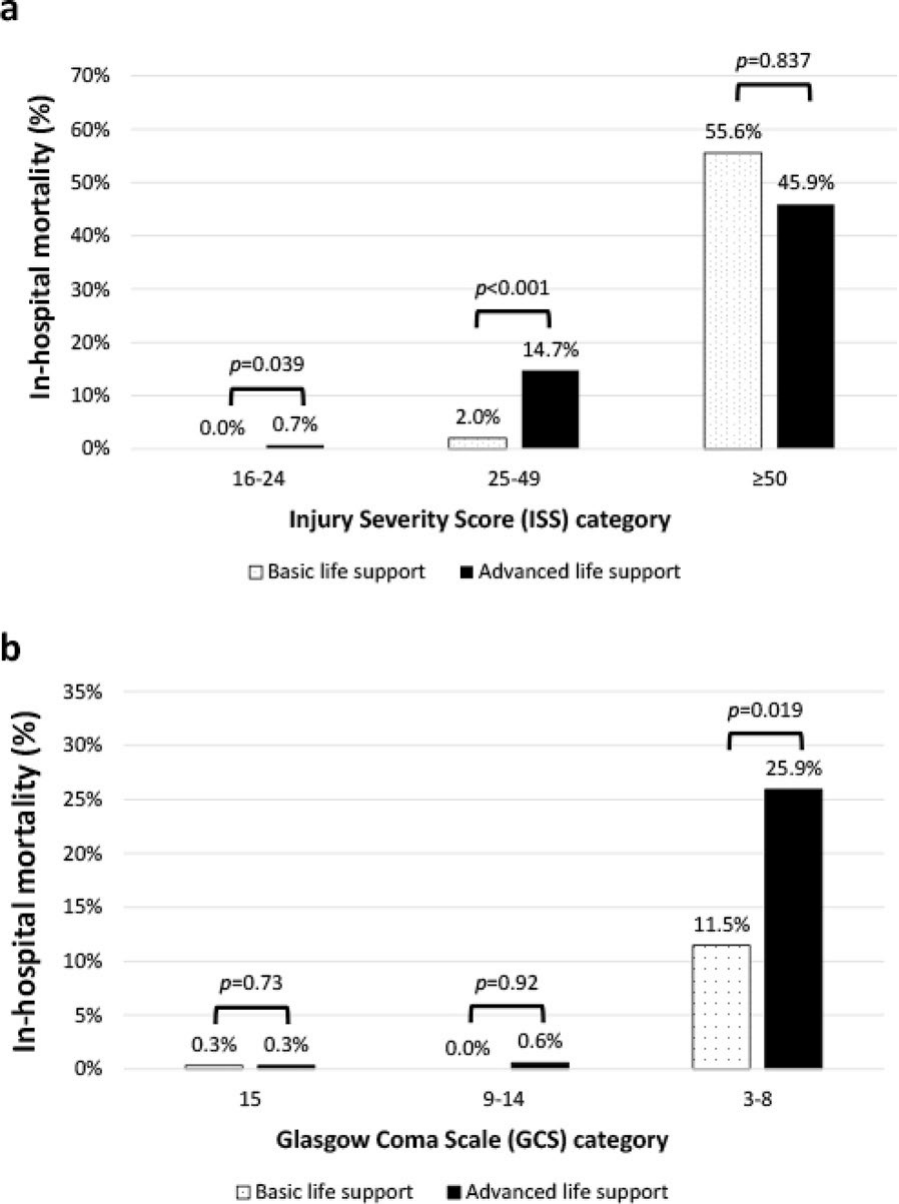
**Fig. 1 (abstract P251)**. The relationships between prehospital mode of transport, in-hospital mortality, injury severity score (**a**), and Glasgow Coma Scale at admission to the emergency department (**b**)

## P252

### The potential benefit of partial REBOA in traumatic hemorrhagic shock patient: a systematic review and meta-analysis

#### N Owattanapanich, T Wongpongsalee, K Keorochana, R Chunhasuwankun, J Sirikun

##### Faculty of Medicine Siriraj hospital, Division of Trauma, Department of Surgery, Bangkok

*Critical Care* 2023, **27(S1)**: P252

**Introduction:** Resuscitative endovascular balloon occlusion of the aorta (REBOA) has been accepted as a rescue procedure for temporary bleeding control in traumatic hemorrhagic shock patients. Complete or full REBOA is the standard procedure utilized for a decade. Some studies pointed out that complete occlusion was associated with higher mortality and complications due to the ischemic-reperfusion. Based on animal studies, partial REBOA has been proposed recently as an option that might help with better perfusion. However, there are scarce clinical data on the effect of partial REBOA [1, 2]. Our study aimed to examine the effect of partial REBOA on traumatic hemorrhagic shock patients.

**Methods:** MEDLINE, EMBASE, and Cochrane Library database searches were conducted using MesH terms for partial REBOA and trauma. An additional search of relevant primary literature and review articles was also performed. Both animal and human research were included. A random effects model was used to estimate the mortality rate using Review Manager 5.4.1 software.

**Results:** Of 512 studies, 39 articles were included in this systematic review. Thirty-six articles were conducted in animal models (swine, sheep, and pig). Due to the variety of outcomes and small sample size, the effect of partial REBOA compared to complete REBOA could not be systematically examined. There were 3 clinical studies (191 patients) on the human population. Partial REBOA demonstrated a trend toward lower mortality compared to complete REBOA (OR 0.65, 95% CI [0.34–1.21], I_2_ = 0%) (Fig. 1).

**Conclusions:** Partial REBOA might have a potential effect on mortality benefit, however, the recent data did not show significant improvement in survival comparing to complete REBOA. A large multicenter prospective study is warranted.


**References**
Matsumura Y et al. Emerg Med J 2017;34:793–799.Madurska MJ et al. Eur J Trauma Emerg Surg 2022;48:299–305.

**Figure Figcz:**
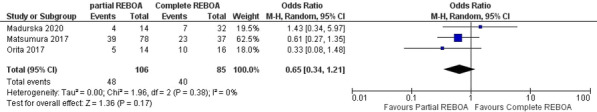
**Fig. 1 (abstract P252)**. Effect of partial REBOA on mortality

## P253

### Electric scooter trauma as a new category of injuries with high hospitalisation and morbidity rates in young and adult patients

#### C Gori^1^, L Forcella^1^, A Martinotti^1^, RM Pani^1^, S Manfroni^2^, G Ricci^2^, P Marini^2^, E Cingolani^1^

##### ^1^San Camillo-Forlanini Hospital, UOSD Shock and Trauma, Rome, Italy, ^2^San Camillo-Forlanini Hospital, UOC General and Emergency Surgery, Rome, Italy

*Critical Care* 2023, **27(S1)**: P253

**Introduction:** Electric scooters (e-scooters) are a new means of transport used worldwide. Their ease of use and low costs allowed a rapid spread in high traffic urban areas although their use increased road accidents. The goals of our study are to describe e-scooter related high morbidity injuries and to raise awareness about this topic.

**Methods:** It is a retrospective analysis conducted from September 2019 to September 2022 in San Camillo Forlanini Hospital (Rome, Italy). All patients presenting to the emergency department (ED) with injuries associated with e-scooter use were included.

**Results:** 273 patients were enrolled (156 males [57%], mean age [SD] 33.2 [13.4] years): 233 were e-scooter riders; only a limited number of them wore helmet. 256 of patients (94%) presented during night time. The mean Injury Severity Score (ISS) was 4.6 (SD ± 11.02); in 60 patients (22%) ISS was > 9. Injuries involved more frequently head (37%), face (22%) and upper limbs (44%). 70 patients required surgery and 7 of them were admitted to intensive care unit. Major head injury occurred in 22 patients (22%), 4 of them had permanent disability. Risk factors for hospitalization include: e-scooter crush during night time (OR 2.17, *p* < 0.05), loss of consciousness at the admission (OR 1.75 *p* < 0,01), need for transfer by helicopter (OR 4.12, *p* < 0.05) and anticoagulant or antiplatelet therapy (OR 5.17, *p* < 0.01).

**Conclusions:** The use of e-scooters is a growing phenomenon worldwide. However, its use is associated with a wide range of injuries that may require hospital admission and surgical intervention, resulting in high morbidity and health care costs. In this setting, use of helmet must be mandatory to reduce head injury associated morbidity. Furthermore, given the remarkably high rates of night time injuries, an e-scooter ban during night could significantly decrease the rate of accidents.

## P254

### Trauma: a stress during pandemic?

#### P Varela Ramos^1^, L Costa^2^, A Braga^2^, L Tavares^3^, AC Santos^4^, B Quental^5^, C Costa^6^, S Carvalho^7^, N Gatta^2^, JA Paiva^2^

##### ^1^Hospital de Vila Franca de Xira, Intensive Care Unit, Vila Franca de Xira, Portugal, ^2^Centro Hospitalar Universitário de São João, Intensive Care Unit, Porto, Portugal, ^3^Hospital do Divino Espírito Santo de Ponta Delgada, Intensive Care Unit, Ponta Delgada, Portugal, ^4^Centro Hospitalar de Entre o Douro e Vouga, Intensive Care Unit, Santa Maria da Feira, Portugal, ^5^Centro Hospitalar Tondela-Viseu, Intensive Care Unit, Viseu, Portugal, ^6^Hospital Beatriz Ângelo, Intensive Care Unit, Loures, Portugal, ^7^Centro Hospitalar de Trás-os-Montes e Alto Douro, Intensive Care Unit, Vila Real, Portugal

*Critical Care* 2023, **27(S1)**: P254

**Introduction:** Coronavirus disease 2019 pandemic significantly impacted on trauma systems, since emergency departments (ED) suddenly were overwhelmed by patients requiring intensive care unit (ICU) admission. Once, trauma volume was supposed to decrease due to lockdown policies, we aimed to describe ICU trauma admissions during this period.

**Methods:** Retrospective observational study of all trauma patients admitted to the ICU of a Portuguese Trauma Center between January 2020 and December 2021. Data were collected from clinical hospital records.

**Results:** 437 trauma patients (15% of all admissions), mostly male (71%), with a median age of 59 years-old (42–74) were included. At least one comorbidity was present in 71% of the patients. Median severity scores were: SAPS II 26 (19–38), SOFA 3 (1–6), ISS 13 (9–22), RTS 8 (6–8) and TRISS 96,75 (81.1–98.6). The most frequent mechanisms of injury were falls (59%) and road traffic accidents (25%). The majority consisted of blunt trauma (88%), 65% of brain trauma and 35% of musculoeskeletal trauma. Trauma Team assessment was started in < 3 min in all cases and median length of stay (LOS) in the ED was 261 min (154–418). Surgical intervention was performed in < 4 h in 56% of surgical brain trauma injuries, in < 6 h in 67% of extremity open fractures and in < 1 h in 6% of a penetrating trauma. Shock, mainly hemorrhagic, was present in 8% of the patients on hospital admission. 38% were submitted to invasive mechanical ventilation and 34% to vasopressors. The most common complication was nosocomial infection (18%). The median LOS in the UCI was 12 days (5–24). Only 8% of the patients died in the ICU and 11% in the hospital.

**Conclusions:** During pandemic, trauma persisted a major health problem with a significant consumption of time and critical care resources. The high influx of patients may have influenced the LOS in the ED before ICU admission and the time until the surgical intervention. Despite it, mortality remained low.

## P255

### Low FEV1 is associated with increased mortality in acute exacerbation of COPD (AECOPD) in one year

#### S Pillai^1^, JC Zaldua^1^, O Watson^1^, M Howard ^1^, M Lawrence^1^, J Whitley^1^, K Hawkins^2^, K Morris^3^, PA Evans^1^

##### ^1^Welsh Centre for Emergev4ncy Medicine Research, Emergency Department, Morriston Hospital, Swansea, UK, ^2^Swansea University, Swansea, UK, ^3^Cardiff Metropolitan University, Cardiff, UK

*Critical Care* 2023, **27(S1)**: P255

**Introduction:** COPD is a chronic inflammatory condition of the lungs and predominantly seen in smokers. As the disease progresses, there is deterioration of FEV1 (forced expiratory volume in 1 s) that measures the airflow limitation. Several studies demonstrated that FEV1 was a better predictor of mortality in COPD patients [1]. This study aims to assess whether FEV1 was associated with mortality in AECOPD patients.

**Methods:** This retrospective analysis included 534 patients presented to the Emergency Department (ED) of a tertiary teaching hospital with acute exacerbation of COPD from the September 2016 to August 2017. The severity of COPD is classified into mild (FEV1 ≥ 80% predicted), moderate (50% ≤ FEV1 < 80% predicted), severe (30% ≤ FEV1 < 50%) predicted and very severe (FEV1 < 30% predicted).

**Results:** 45% (239/534) of the patients had documented FEV1 with a mean age was 68 ± 10 years and 52% were females. 10/239 (4%) died during admission and 49/239 (21%) died in one-year. Most number of admissions was severe group (39%). The FEV1 was lower in patients who died on this admission, however not significant when compared to those who survived [42 ± 19 vs 49 ± 22 (*p* = 0.32)]. Patients who died at one-year had a significantly lower FEV1 when compared to those who survived [43 ± 22 vs 50 ± 22 (*p* = 0.02)] (Table 1). Patients with very severe COPD died the most during admission and at one-year (9% and 42%).

**Conclusions:** COPD exacerbation remains the illness of elderly and common in females. Less than half of COPD exacerbations had a documented FEV1. Patients with lower FEV1 are significantly associated with one-year mortality. As expected, COPD patients with very severe disease are at risk of death during admission and at one-year.


**Reference**
Bikov et al. Int J Chron Obstruct Pulmon Dis 2020;15:1135–1142.

**Table Tabch:** **Table 1 (abstract P255)**. FEV1 was significantly low in people who died in one-year following exacerbation of COPD

FEV1	Died	Survived	*p* value
On admission	42 ± 19	49 ± 22	0.32
One-year	43 ± 22	50 ± 22	0.02

## P256

### Does clot microstructure (d_*f*_) predict mortality in acute exacerbation of chronic obstructive pulmonary disease (AECOPD)?

#### S Pillai^1^, JC Zaldua^1^, O Watson^1^, M Howard^1^, M Lawrence^1^, J Whitley^1^, K Hawkins^2^, K Morris^3^, PA Evans^1^

##### Welsh Centre for Emergency Medicine Research, Emergency Department, Morriston Hospital, Swansea, UK, ^2^Swansea University, Swansea, UK, ^3^Cardiff Metropolitan University, Cardiff, UK

*Critical Care* 2023, **27(S1)**: P256

**Introduction:** COPD is an irreversible chronic inflammatory condition of the lungs and is the third leading cause for death worldwide with 3.23 million deaths in 2019. Fractal dimension (d_*f*_), the biomarker of clot microstructure has shown to be increased in acute inflammatory conditions and predict mortality in sepsis patients [1]. There is high in-hospital mortality during acute exacerbations (2.5% to 25%) and one-year mortality after exacerbation (26%). The aim of the study was to investigate whether the clot microstructure predict mortality in AECOPD patients.

**Methods:** 85 AECOPD patients were recruited from the emergency department of a tertiary teaching hospital. Blood samples were taken to perform fractal dimension (d_*f*_), full blood count (FBC), platelet aggregometry, PT, aPTT, fibrinogen, D-dimer, procalcitonin (PCT), CRP and Factor XIII.

**Results:** 10/85 (12%) patients died and 75/85 (88%) survived. The patients who died had a significantly high d_f_ when compared to those who survived (1.76 ± 0.03 vs 1.71 ± 0.06, *p* = 0.02) (Fig. 1). There was no statistical significance in inflammatory or coagulation markers between both groups. 16/75 (21%) patients died within one-year, however there was no statistical significance between the d_*f*_ of died or survived group (1.70 ± 0.05 vs 1.71 ± 0.06, *p* = 0.79).

**Conclusions:** COPD patients with exacerbations had high in-hospital and one-year mortality. The patients who died had denser and tighter clot microstructure as reflected by significantly high d_*f*_ at presentation. This shows that these patients had higher inflammatory response during exacerbations. Therefore, d_*f*_ can be used to predict mortality in COPD patients during exacerbations and may help in COPD management and prognostication, however this study was not powered for outcome.


**Reference**
Davies GR et al. Intensive Care Med 2016;42:1990–1998

**Figure Figda:**
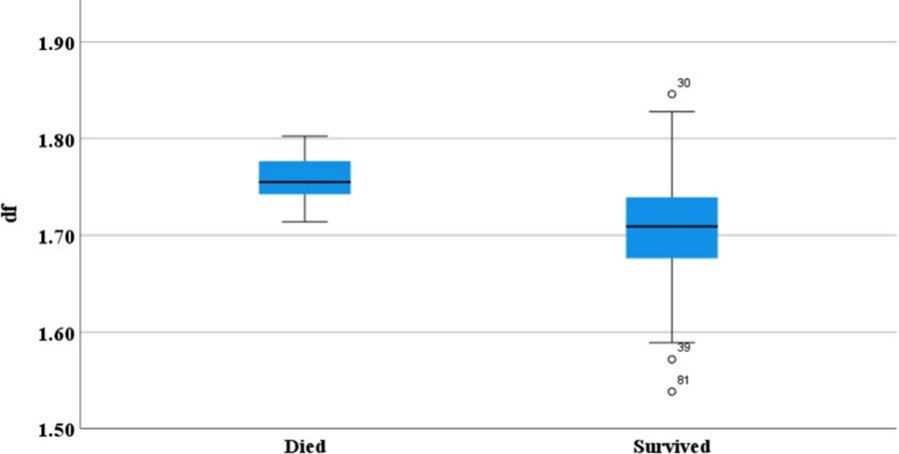
**Fig. 1 (abstract P256)**. D_*f*_ is significantly elevated in AECOPD patients who died compared to those who survived (*p* = 0.02)

## P257

### Genetic, cellular and molecular prognostic markers for suppurative lung diseases

#### V Pisarev^1^, A Chumachenko^1^, D Fetlam^2^, M Vyazmina^1^, E Ershova^1^, S Kostyuk^3^, A Grechko^4^

##### ^1^Federal Research and Clinical Center of Intensive Care Medicine and Rehabilitology, V.A.Negovsky Institute of General Reanimatology, Moscow, Russian Federation, ^2^I.V. Davidovsky City Clinical Hospital, Moscow, Russian Federation, ^3^Research Centre for Medical Genetics, Moscow, Russian Federation, ^4^Federal Research and Clinical Center of Intensive Care Medicine and Rehabilitology, Moscow, Russian Federation

*Critical Care* 2023, **27(S1)**: P257

**Introduction:** The incidence of suppurative lung diseases (SLD) including pleural empyema (PE) is increasing worldwide. Bronchopleural fistula (BPF) that may complicate PE has high mortality [1]. Prognosis of course and outcome is needed to personalize monitoring and care of SLD patients. We propose that polumorphism of OLR1 gene encoded proinflammatory receptor LOX-1, circulating extracellular DNA (cfDNA) and immune cells ratio may impact prognosis of SLD. The aim of the study was to define whether that candidate biomarkers may stratify the SLD patients according to the risk of unfavorable course/outcome.

**Methods:** Study cohort included 136 patients (29% women) with SLD (PE, lung abscess, pulmonary gangrene). Median age was 54 (IQR, 41–67), median SOFA score was 2 (IQR, 2–4) on admission. CfDNA was isolated from plasma using organic solvents, DNA concentration was determined by SYBR Green. Genotyping of *OLR*1 rs11053646 alleles was performed using a PCR and designed allele-specific tetra primers followed by electrophoretic separation of the products. Medians/odds ratio (OR) and Fisher exact test (FET)/Mann–Whitney U test were employed for calculations and statistics, respectively.

**Results:** BPF complicated PE patients less commonly if they carried minor allele *OLR*1 rs11053646 encoding 167N variant of LOX-1 molecule (*P* = 0.003 by FET, n = 134, OR = 5.9, 95% CI 1.6–21.1). Carriers of allele encoding N variant (K167N, N167N) with PE experienced decreased SOFA values (*p* = 0.038, U test, n = 102). Neutrophil-to-lymphocyte ratio (NLR) > 4 and plasma cfDNA concentration > 170 ng/ml significantly associated with poor prognosis in a whole cohort of patients with SLD (*p* = 0.0003, FET, n = 136 and *p* = 0.049, FET, n = 42, respectively) (Fig. 1).

**Conclusions:** The presence of both *OLR*1 rs11053646 alleles correspondent to KK, increased NLR ratio above 4 and enhanced cfDNA level above 170 ng/ml in plasma on admittance are novel prognostic candidate biomarkers of unfavorable course and outcome of SLD.


**Reference**
Shin K et al. Acute Med Surg. 2021;8:e621.

**Figure Figdb:**
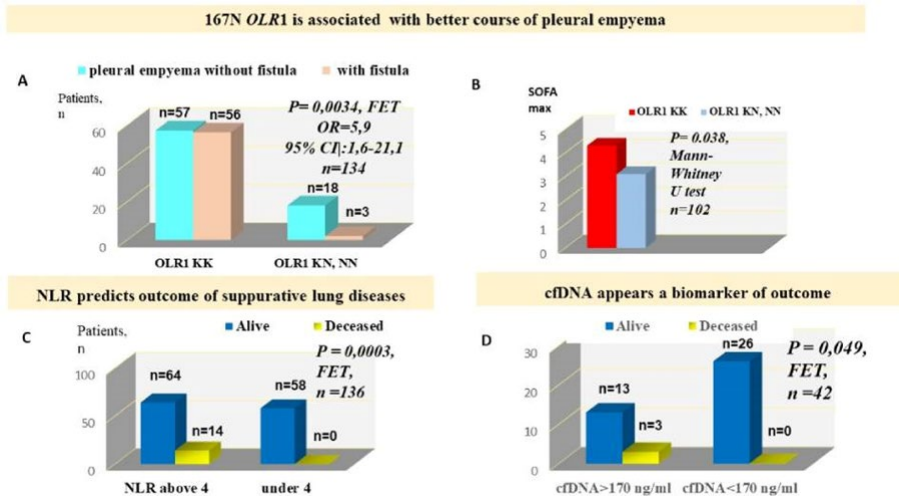
**Fig. 1 (abstract P257)**. Candidate biomarkers of purulent lung diseases course and outcome. **A** The incidence of fistulous complication in patients with pleural empyema depending on OLR1 rs11053646 genotype. **B** Maximum SOFA score during hospitalization of patients depending on OLR1 rs11053646 genotype. **C** Survival of patients depending on neutrophil-to-lymphocyte ratio. **D** Survival of patients depending on cfDNA level in plasma

## P258

### Recommendations for accepted clinical indications of hyperbaric oxygen therapy for intensive care unit patients: a systematic review

#### J Kroon, M De Waard, N Visschers, G Oedairadjsingh, H Endeman

##### Erasmus MC, Intensive Care, Rotterdam, Netherlands

*Critical Care* 2023, **27(S1)**: P258

**Introduction:** This study describes the recommendations for accepted clinical indications by the Dutch healthcare authority on hyperbaric oxygen therapy for patients that are submitted to the intensive care unit.

**Methods:** A systematic review was performed. Articles regarding clinical indications of hyperbaric oxygen therapy and patients submitted to intensive care were included. Data extraction and the assessment of the risk of bias were performed and the results were divided per indication.

**Results:** A total of eleven studies met all the inclusion criteria (Fig. 1). In five studies carbon monoxide (CO) poisoning was analysed. In four studies necrotizing soft tissue infections were analysed, in one study air embolism was reviewed and in one study osteoradionecrosis was reviewed. Mortality and altered mental status were reviewed.

**Conclusions:** There is a correlation between hyperbaric oxygen therapy and reduced mortality in carbon monoxide intoxication, but a higher incidence of delayed neurological sequelae in carbon monoxide intoxication. There is a significant decrease in neurological status in patients with CO intoxications treated with hyperbaric oxygen therapy, but these patients had also a more severe levelof CO poisoning. Hyperbaric oxygen therapy has no significant effect on patient survival for patients with necrotizing soft tissue infections. Hyperbaric oxygen therapy has no significant association with wound breakdown and flap complications in osteoradionecrosis. Our recommendation would be to further investigate the clinical use of hyperbaric oxygen therapy in intensive care patient through multicenter studies.

**Figure Figdc:**
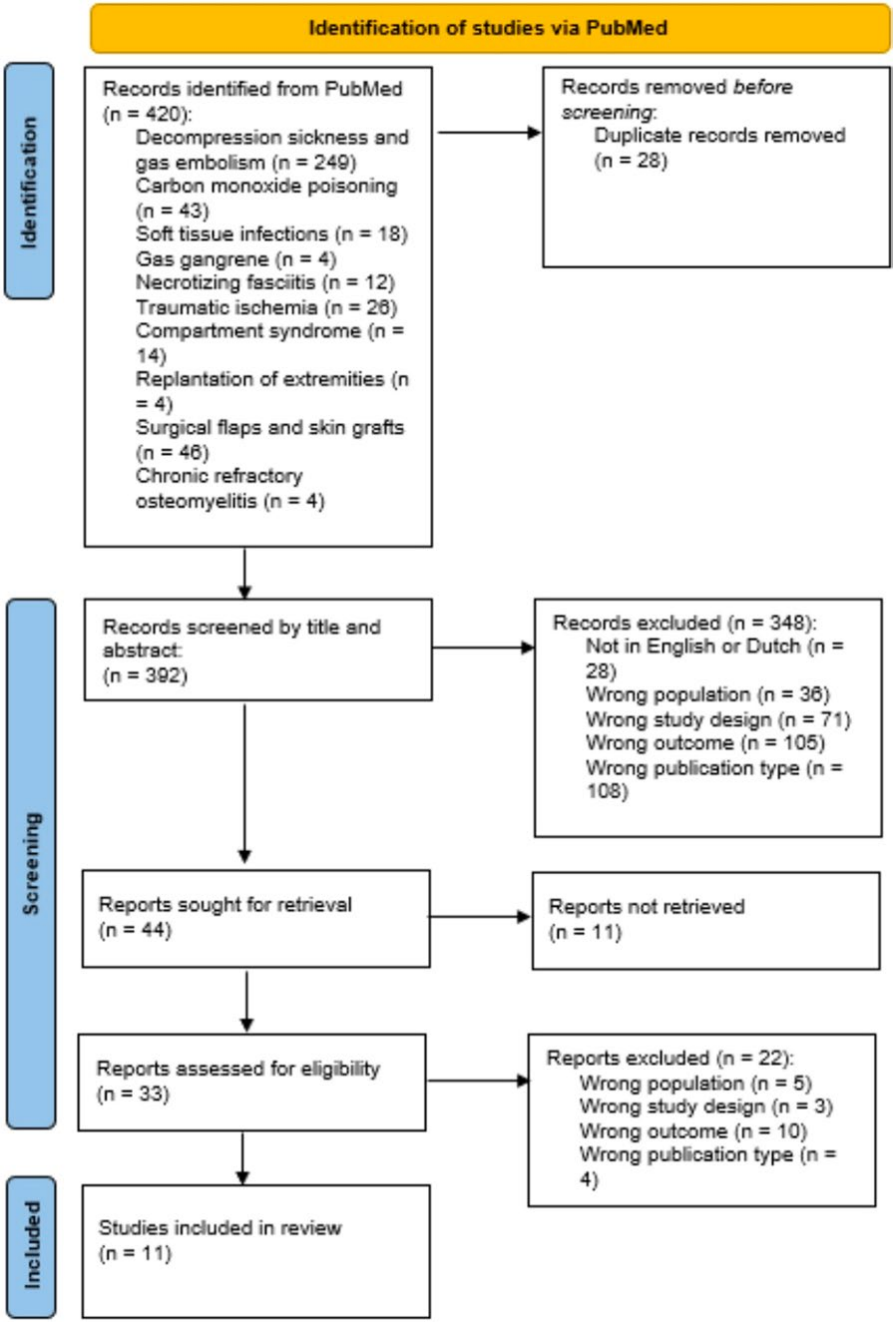
**Fig. 1 (abstract P258)**. Flowchart based on the PRISMA 2020 flow diagram

## P259

### Non-ST-elevation myocardial infarction: the prognostic value of leuko-platelet index

#### W Bahria^1^, S Yamoune^1^, Y Walha^1^, Y Zouaghi^1^, L El Mohsni^1^, A Sadki^1^, I Lagha^2^, F Azaiez^2^, N Nouira^1^

##### ^1^Mongi Slim Academic Hospital, Emergency Department, La Marsa, Tunisia, ^2^Mongi Slim Academic Hospital Cardiology Department, Cardiology Department, La Marsa, Tunisia

*Critical Care* 2023, **27(S1)**: P259

**Introduction:** Acute coronary syndrome is followed by a significant thrombotic and inflammatory reactions. Recent studies have validated a leuko-platelet index (LPI) in patients with non-ST-elevation myocardial infarction (NSTEMI) to predict the occurence of ischemic events, heart failure and mortality. We studied the prognostic value of LPI with these side effects.

**Methods:** We conducted an observational prospective single-center study over 1 year including patients admitted to the Emergency Departement (ED) for NSTEMI. The reverse leuko-platelet index (rLPI = 1/(platelet count x leucocyte count) × 10^8^) was calculated for all patients on admission in the ED. A cut-off point of LPI ≤ 24 was used. We studied the association between LPI and the risk of intra-hospital mortality, shook or heart failure and mortality at 6 months (combined end point- CEP). Describe data are presented as the means SDs or as numbers (percentages). ROC curves were used to identify variables predicting CEP.

**Results:** One hundred and eighty five patients were included in our study. The mean age was 63 years old [27–93] with a sex ratio of 1.60. Seventy five patients (41%) had a history of coronary syndrome and 63 (34%) were treated by an anti platelet treatment. The CEP was shown in 72 patients (45%). From those, 55 patients (76%) were treated by an anti platelet treatment and 63 patients had an ILP ≤ 24. The mortality at 6 months was shown in 11 patients (7%). The analytical study showed that the LPI^−1^ was significantly higher in patients with CEP (*p* = 0.000; OR = 0.769; 95% CI [0.693–0.844]) and every decrease over 24 in LPI was associated with a CEP increase (*p* = 0.000; OR = 2.52; 95% CI [1.49–4.27]).

**Conclusions:** The LPI appears to be a simply and fast index, whose the lower values identifies patients with NSTEMI at high risk for CEP, in opposition with the results of the studies in the literature.

## P260

### Supraventricular tachycardias: the prognostic value of troponin level 

#### W Bahria, S Yamoune, D Hamdi, Y Zouaghi, F Hamdi, F Lazzez, N Nouira. Mongi Slim Academic Hospital, Emergency Department, La Marsa, Tunisia

*Critical Care* 2023, **27(S1)**: P260

**Introduction:** The association between cardiac troponin elevation and supraventricular tachycardias (SVT) has been reported in a limited studies. We investigated the prognostic value of an elevated troponin level among patients presenting with SVT in the emergency departement (ED).

**Methods:** This is an observational prospective single-center study conducted over three years including patients admitted to the ED with SVT. Serum ultra-sensitive troponin (US Tn) was measured for all patients on admission. and a positive result was defined as a serum level more than 19 ng/dl. The primary endpoint was the mortality in the ED.

**Results:** One hundred and sixty nine patients were included in our study. The mean age was 66 years old [18–95] with a sex ratio of 0.62. The mean US Tn level was 499 [0–40000] ng/dl. A positif US Tn was shown in 91 patients (57%). Twenty patients (12%) died in the ED. Of those, 16 died in the subgroup of patients with a positif US Tn. The univariate analysis showed that elevated creatinine level measured on admission (*P* = 0.02; OR = 26.244; CI 95% [10.021–116.233]) and acute pulmonary edema (*p* = 0.019; OR = 2.13; CI 95% [1.022–4.412]) were associated with an elevated troponin level. However, the age, the gender and the heart rythm were not associated with an elevated troponin level with respectively (*p* = 0.485), (*p* = 0.56) and (*p* = 0.397). The US Tn, at any level was associated with the mortality in the ED for patients admitted with a SVT (*p* = 0.01; OR = 4.08; 95% CI [1.24—13.4]).

**Conclusions:** Elevated US Tn among patients presenting with SVT is associated with an increased risk of in-hospital mortality.

## P261

### Accuracy of aortic dissection detection risk score and D-dimer to rule out acute aortic syndromes in the emergency department

#### A Khan, C Masoura, A Mitra, T Smith

##### Imperial College Healthcare NHS Trust, Emergency Department, London, UK

*Critical Care* 2023, **27(S1)**: P261

**Introduction:** Acute aortic syndromes (AAS) are rare but potentially fatal vascular emergencies. Early recognition and management are essential in preventing mortality [1]. Whilst AAS is most commonly associated with severe tearing pain, it can also be painless or present with stroke-like neurological symptoms. This wide variety of presentations presents a challenge to emergency physicians when deciding which patients require computerised tomography aortogram (CTA) imaging, especially with already stretched health care services. This study aims to evaluate the diagnostic accuracy of the aortic dissection detection risk score (ADD-RS) combined with D-dimer to rule out AAS in the emergency department [2].

**Methods:** This retrospective single centre study looks at 2 years’ worth of patients who received CTA for suspected AAS at a tertiary vascular hospital emergency department. D-dimer result from day of presentation was collected and ADD-RS was calculated retrospectively from electronic patient notes.

**Results:** A total of 190 CTAs were performed, 14 (7.4%) showed a new AAS. Patients with new AAS were significantly older (73.9 vs 61.3 years, *p* < 0.001), had higher ADD-RS (1.6 vs 1.1, *p* < 0.05) and higher D-dimer (11,761 vs 1331 ng/ml, *p* < 0.05). For ruling out AAS, ADD-RS ≤ 1/normal D-dimer had sensitivity of 100.0% (95% confidence interval [CI] 73.5–100.0) and specificity of 31.0% (95% CI 22.1–41.0). Negative predictive value (NPV) was 100%. In patients with ADD-RS ≤ 2/normal D-dimer sensitivity was 100.0% (95% CI 71.5–100.0) and specificity was 50.6% (95% CI 39.3–61.9). NPV was also 100%. No patient with AAS had a normal D-dimer.

**Conclusions:** Using ADD-RS ≤ 1 or ADD-RS ≤ 2 (depending on availability of CTA) combined with normal D-dimer appears highly sensitive for ruling out AAS. Incorporation of this diagnostic pathway (Fig. 1) in the emergency room could significantly reduce the number of patients exposed to unnecessary radiation.


**References**
Harris KM et al. Circulation 2011;124:1911–8.Nazerian P et al. Circulation 2018;137:250–8.

**Figure Figdd:**
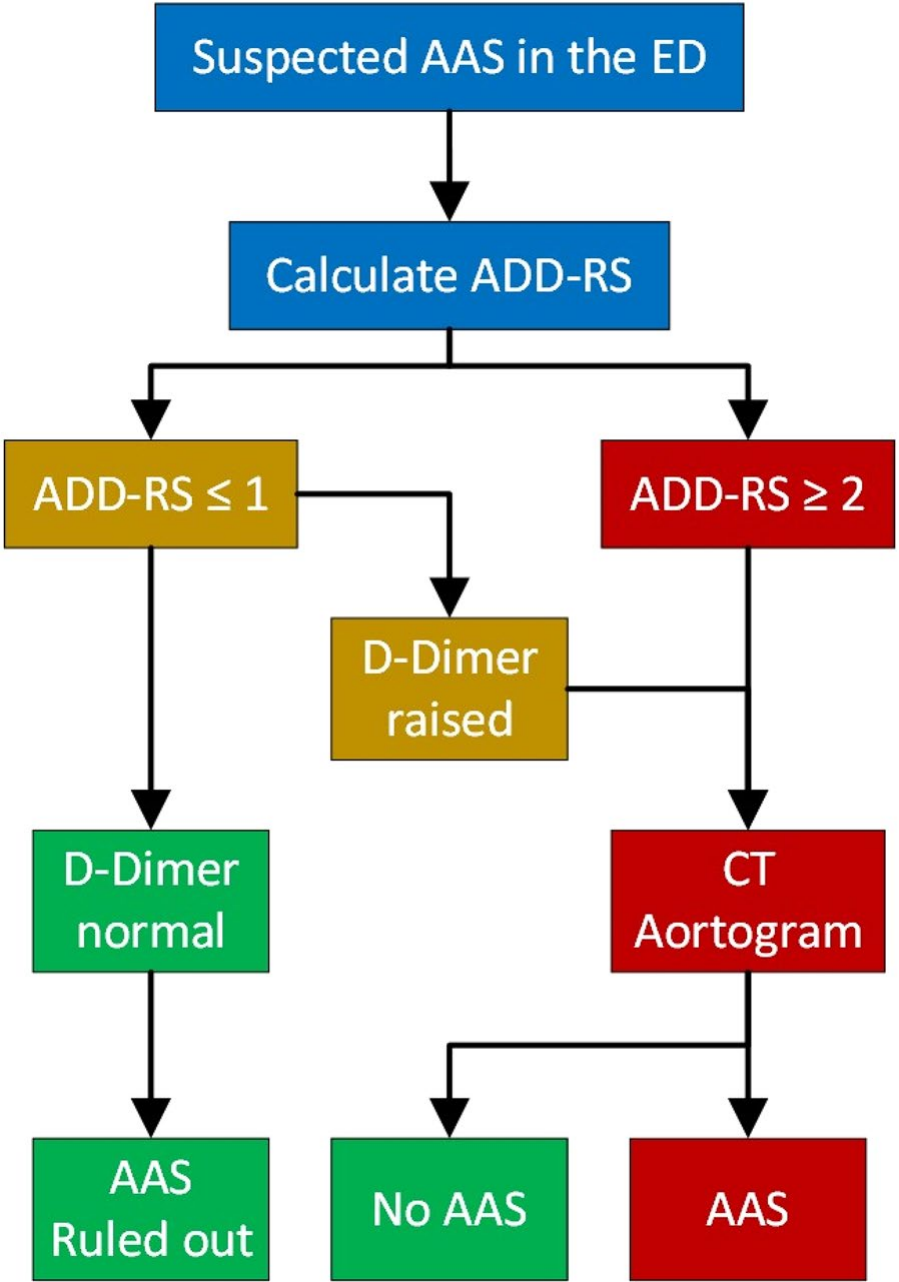
**Fig. 1 (abstract P261)**. Simplified proposed flowchart for investigating suspected AAS in the emergency department based on the ADvISED study algorithm [2]. AAS = acute aortic syndrome. ED = emergency department, ADD-RS = aortic dissection detection risk score, CT = computerised tomography

## P262

### The management of acute organophosphate toxicity in a regional hospital in Johannesburg, South Africa: a retrospective chart review

#### V Khonje, J Hart, J Venter, S Deonarain, S Grossberg

##### Thelle Mogoerane Hospital, Emergency Department, Johannesburg, South Africa

*Critical Care* 2023, **27(S1)**: P262

**Introduction:** Organophosphate exposures pose a significant health-care related burden on South African communities. This study will review the demographics, characteristics and clinical course of patients presenting with acute organophosphate toxicity to an emergency department in Johannesburg, South Africa.

**Methods:** A retrospective chart review of all patients treated for possible acute organophosphate toxicity from January 2020 to August 2021.

**Results:** One hundred and thirty-four patients were included in the study. The median age was 26 years with a male predominance (male = 56%, female = 44%) (Fig. 1). 109 patients (81.3%) survived, 18 patients (13.4%) demised and the outcome of 7 patients (5.2%) was unknown. The median hospital length of stay was 8 days, (IQR = 5–13), the longest hospital stay was 37 days in ICU. Atropinisation dose was significantly higher for intubated patients (median = 140.0 mg, IQR = 90–219.5 mg) compared to patients who were not intubated (median = 60 mg; IQR = 20.5–120 mg; *p* < 0.05). The length of stay was significantly higher for intubated patients (median = 11 days; IQR = 7–15) (*p* < 0.00) compared to patients who were not intubated (median = 5 days; IQR = 3–8, *p* < 0.00). There was a moderate positive correlation between atropinisation dose and length of stay (correlation coefficient = 0.37, *p* < 0.00). There was a moderate negative correlation between atropinisation dose and cholinesterase level (correlation coefficient = − 0.39, *p* < 0.00). Of those reported to have adverse effects 78.6% were related to atropine toxicity.

**Conclusions:** Our study shows a high mortality rate secondary to organophosphate exposure. Significant exposures and thus higher doses of atropine were associated with increased length of stay and need for intubation. We found a high incidence of atropine related adverse effects. More studies are needed to further establish the balance between the therapeutic and adverse effects of high dose atropine as a treatment modality for organophosphate toxicity.

**Figure Figde:**
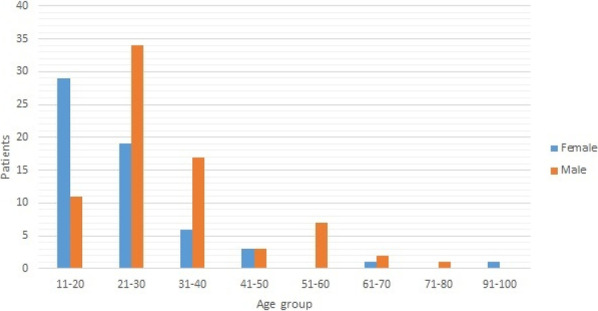
**Fig. 1 (abstract P262)**. Age and sex distribution

## P263

### Calcium channel blocker (CCB) toxicity in critically ill patients: an observational study

#### S Omar^1^, V Shukla^2^

##### ^1^University of Witwatersrand, Intensive Care/Critical Care, Johannesburg, South Africa, ^2^University of Witwatersrand, Intensive Care Unit, Johannesburg, South Africa

*Critical Care* 2023, **27(S1)**: P263

**Introduction:** Calcium channel blockers (CCB) account for 35% of cardiovascular drug overdoses in the USA, its management is variable and the burden in our setting is undefined. Our main objective was to determine the prevalence of CCB overdose at the Chris Hani Baragwanath Academic Hospital adult ICU, management strategies and the patient outcomes.

**Methods:** We performed a retrospective observational study (2020–2021). Ethical approval was obtained through the University of the Witwatersrand Human Research Ethics Committee (HREC).

**Results:** We admitted 1317 patients to the Intensive Care Unit (ICU) during the study period. Demographic and organ dysfunction/support are provided in Table 1. The prevalence of toxin ingestion admissions was 14% (180/1317), 95% confidence interval (CI) 13–15%. CCB overdose accounted for 5%,CI 3–7%, (9/128) of toxin ingestion admissions. After correction of volume status, the frequency of interventions utilised were vasopressors (78%), HIE therapy (67%), calcium (44%), methylene blue (11%) and hemoperfusion (44%). Maximum vasopressor dose was achieved in the first 8 h while lactate levels peaked after 8 h from admission. The median duration of ventilation, vasopressor use, length of ICU stay were 6 days (CI 0–7), 5 days (CI 4–6), and 9 days (CI 6–11), respectively. Patients receiving hemoperfusion had a trend to lower duration of ventilation (2 fewer days), vasopressor days (1.5 fewer days), and ICU LOS (1.5 fewer days). ICU Mortality was 33% for the group and 25% for those undergoing hemoperfusion.

**Conclusions:** Calcium channel blocker overdose is a significant healthcare burden with a high mortality. Novel strategies such as hemoperfusion may be considered as a rescue therapy when disease is refractory to standard therapy.

**Table Tabcv:** **Table 1 (abstract P263)**. Demographic and organ dysfunction/support

Variable	All, n = 9	Standard Care, n = 5	Hemoperfusion (HA) n = 4
Age years, median (IQR)	18 (18–26)	18 (17–19)	22 (18–26.5) *p* = 0.56
Weight kg (median (IQR)	70 (62–71)	71 (62–75)	69.4 (64.4–70.5) *p* = 0.56
Gender (female) n(%)	8/9 (89%)	5/5 (100%)	3/4 (75%)
SAPS II, median (IQR)	27 (18–35)	24 (18–27)	33,5 (28.5–35)
Vasopressor use	7/9 (78%)	3/5 (60%)	4/4 (100%)
Mechanical ventilation	6/9 (67%)	3/5 (60%)	3/4 (75%)
Acute kidney injury	5/9 (56%)	2/5 (40%)	3/4 (75%)

## P264

### Acute complications and treatment in critically ill MDMA-intoxicated patients

#### MJ Zuidema^1^, EJ Reimerink^2^, D Akhoundzadeh^3^, B Van den Bogaard^1^, FMJ Gresnigt^4^

##### ^1^OLVG, Intensive Care, Amsterdam, Netherlands, ^2^Amsterdam UMC, Emergency Department, Amsterdam, Netherlands, ^3^Amsterdam UMC, Amsterdam, Netherlands, ^4^OLVG, Emergency Department, Amsterdam, Netherlands

*Critical Care* 2023, **27(S1)**: P264

**Introduction:** The persistent increase in the use of 3,4-Methylenedioxymethamphetamine (MDMA) has led to an increase of emergency department presentations. We aim to study the most frequent reasons of admission to the Intensive Care Unit (ICU) and describe complications, management and outcome of critically ill MDMA intoxicated patients.

**Methods:** This retrospective cohort study included all patients with a confirmed or self-reported MDMA intoxication who were admitted to ICU of OLVG hospital, a tertiary care hospital in Amsterdam, between 2010 and 2020.

**Results:** Seventy-four (73% male) patients were included. Three patients (4%) died; all other patients made a full recovery. Twenty-three patients (31%) were admitted to the ICU because of a threatened airway due to trismus, of whom 14 (61%) were intubated. One patient developed aspiration pneumonia and in one patient, clinical course was complicated by rhabdomyolysis, acute kidney injury (AKI) and acute liver injury (ALI). The median ICU length of stay was 9 h (IQR 7–13). The second most common reason of ICU admission was hyponatremia (n = 15, 20%), of whom 11 (73%) were treated with hypertonic saline. Median sodium correction after 8 h was 13 mmol/L (IQR 7–15). One patient died due to brain edema. Lastly, 8 patients (11%) presented with hyperthermia, of whom 7 patients (88%) received cooling therapy. All displayed secondary complications, such as rhabdomyolysis, AKI, ALI and disseminated intravascular coagulation (DIC). The median length of stay was 41 h (IQR 11–123). In one patient, hyperthermia led to multiple organ failure and death.

**Conclusions:** The three most common complications of MDMA use necessitating ICU admission are trismus, hyponatremia and hyperthermia. Even though the majority of patients included in this study made a full recovery, 4% died. Especially in patients presenting with hyperthermia, complications such as rhabdomyolysis, AKI, ALI and DIC can arise.

## P265

### Metfomin associated lactate acidosis in patients with AKI

#### M Novacek, L Novackova

##### Oblastni nemocnice Kolin, a.s., Anaesthesia and ICU, Kolin III, Czech Republic

*Critical Care* 2023, **27(S1)**: P265

**Introduction:** Metformin associated lactate acidosis (MALA) is the main concern during metformin treatment with mortality rates between 30 – 50%. Metformin is used for the treatment of type 2 diabetes. Normal or slightly decreased renal function must be confirmed during metfomin treatment. In therapeutic use, the incidence of MALA is very low, 0.03 cases per 1000 patiens per year [1]. Intentional overdose with metformin is very rare. Unintentional overdose occurs in the case of acute kidney injury.

**Methods:** We present our retrospective analysis of 26 patients hospitalised in ICU in a regional hospital in Kolin, Czech Republic with MALA during the period 2017–2022.

**Results:** All patients were presented with AKI. Nausea and vomiting, often combined with diarrhea were the most common reason for AKI. All patients had plasmatic metformin level in the toxic range (therapeutic range 0.1–4 ug/ml). None of them were confirmed as an intentional overdose. CRRT was used for all 26 patients, 17 patients were treated with mechanical ventilation, and inotropic support required 24 patients. 12 patients survived and were discharged from ICU. 14 patients died during their ICU stay. There was no statistically significant relationship between mortality and observed parameters on admission to the ICU including age, pH, BE, plasmatic metformin level, daily dose of metformin and creatinine level were observed (Table 1).

**Conclusions:** Mortality rates of more than 50% were observed in our analysis. Treatment with CRRT and inotropic support is the recommended management for treatment of MALA. Plasma metformin levels, pH, lactate levels do not correlate with mortality.


**Reference**
Bailey CJ et al. Metformin. N Engl J Med. 1996;334:574–9.

**Table Tabcg:** **Table 1 (abstract P265)**. Results

Parameter on ICU admission	Survivals (n = 12) mean (range)	Non-survivals (n = 14) mean (range)	*p* value
Age (years)	67.4 (48–83)	71.4 (65–83)	0.3399
pH	6.94 (6.636–7.26)	6.91 (6.6–7.245)	0.781
BE (mmol/l)	−25.67 (−35.6 to −17.7)	−25.75 (−35.1 to −17.1)	0.9385
Lactate (mmol/l)	10.86 (5.93–15.19)	9.94 (5.78–13.97)	0.5604
Plasmatic metformin level (ug/ml)	46.04 (8–130.24)	36.6 (6.96–70.3)	0.6085
Metformin daily dose (mg)	2172 (500–3000)	1890 (850–3000)	0.1895
Serum creatinine (umol/l)	643 (352.7–1066.6)	577.5 (152–873)	0.6673

## P266

### An unusual case of lactic acidosis (LA) and hypoglycemia in adult young woman: hereditary fructose intolerance (HFI)

#### A Casazza, E Bellazzi, D Ciprandi, L Pedrotti, A Poretti, R Preda, MP Storti, R Vanzino

##### Ospedale Civile di Vigevano ASST Pavia, Anaesthesia and Intensive Care Vigevano, Vigevano, Italy

*Critical Care* 2023, **27(S1)**: P266

**Introduction:** LA and hypoglycemia are common in ED. Their contemporary presence may be a metabolic disease sign. HFI is a recessively transmitted disease, caused by Aldolase B (AB) deficiency, leading to LA, hypoglycemia, liver and kidney failure and abdominal symptoms due to fructose intake.

**Methods:** A young woman showed asthenia and drowsiness during train travel. Rescued by emergency team, she was severely hypoglycemic. Due to stupor persistence despite glucose administration, she was admitted to ED. Restless and polypneic she referred vomiting and abdominal pain. She had hemodynamic stability and no hypoperfusion signs. Laboratory findings showed pH = 7,1, lactate = 9.8 mmol/l, glycemia = 60 mg/dl and phosphorus = 1.7 mg/dl. She said she ate sweets and was affected by HFI.

**Results:** 2 days glucose and phosphate administration led to symptoms and acid–base status improvement. HFI is diagnosed in infants when, exposed to fructose, showed abdominal pain, lethargy and coma. Diet ensures growth and wellness. Undiagnosed patients develop a protective aversion to sweets and vegetables, preventing symptoms and liver and kidney disease onset. Fructose is converted to fructose-1-phosphate (F1P) which is cleaved by AB in glyceraldehyde (G3P) and dihydroxyacetonephosphate (DHAP). AB deficiency causes continuous phosphorylation with phosphorus and ATP depletion. F1P has direct renal and hepatic toxicity. G3P and DHAP reduction and F1P increase cause glycolysis and glycogenolysis impairment, leading to post prandial hypoglycemia and cause accumulation of the Krebs circle precursors LA (Fig. 1). Purines release, low ATP and acidosis cause abdominal symptoms [1].

**Conclusions:** In our case, LA, hypoglycemia, hypophosphatemia and GI symptoms were sustained by HFI. Treatment is symptomatic. Despite HFI is rare, severe symptoms may occur in some no-HFI patients when exposed to fructose. Fructose solutions should be always discouraged.

**Acknowledgement:** Written informed consent to publication obtained.


**Reference**
Singh SK et al. World J Clin Pediatr 2022;11:321–329

**Figure Figdf:**
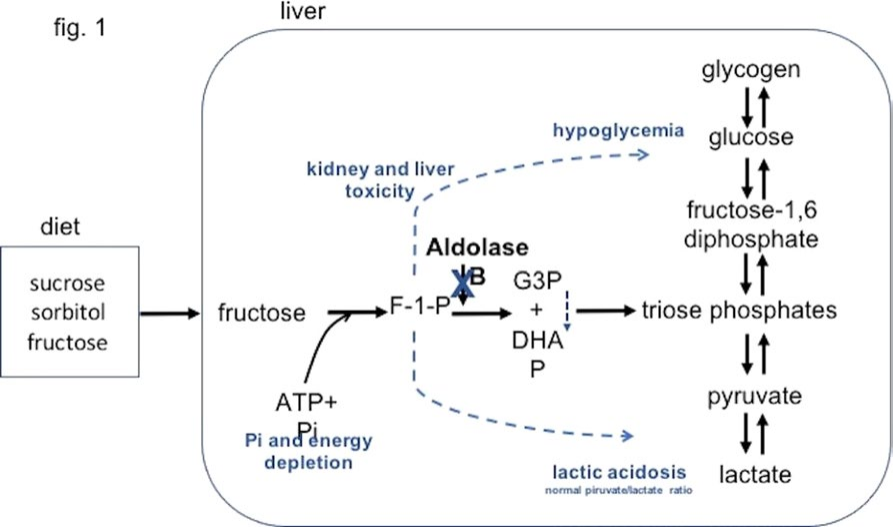
**Fig. 1 (abstract P266)**. Aldolase B deficiency

## P267

### Development of prediction score for predicting 28-day mortality of older sepsis patients in emergency department

#### P Sanguanwit^1^, C Yuksen^1^, J Khorana^2^, K Sutham^3^, Y Phootothum^1^, S Damdin^1^

##### ^1^Faculty of Medicine, Mahidol University, Emergency Department, bangkok, Thailand, ^2^Faculty of Medicine, Chiang Mai University, Center of Clinical Epidemiology and Clinical Statistic, Chiang Mai, Thailand, ^3^Faculty of Medicine, Chiang Mai University, Emergency Department, Chiang Mai, Thailand

*Critical Care* 2023, **27(S1)**: P267

**Introduction:** We had limit data to evaluate sepsis scoring to predicted mortality in older population. We aim to develop an easy prediction score that used at the point of initial assessment of emergency department (ED) for predicted 28-day mortality in suspicious older sepsis patients.

**Methods:** There was a retrospective cohort design. All data were collected through electronic medical record database (EMR) during 1 August 2018–31 December 2018. Multivariable logistic regression was using to identify independent predictors. Backward elimination was applied. Logistic coefficients were used for score weighting and transformation. Predictive performance was validated and compared among the new score with SIRS, qSOFA, NEWS and Ramathibodi early warning score (REWS) by nonparametric receiver operating characteristic (ROC) regression.

**Results:** A total of 599 suspicious older sepsis patients were included. After explored predictors and backward eliminated, the new score was composed of 7 final predictors which were malignancy, dependent status, HR, RR, oxygen saturation, consciousness and lactate. The prediction score was good discrimination area under the ROC curve (AuROC): 0.87, 95% confidence interval (CI): 0.82–0.92) which was significant higher than SIRS (AuROC: 0.62, 95% CI 0.53–0.71) *p* < 0.01, qSOFA (AuROC: 0.72, 95% CI 0.66–0.79) *p* < 0.01, NEWS (AuROC: 0.74, 95% CI 0.67–0.82) *p* < 0.01, REWS (AuROC: 0.71, 95% CI 0.62–0.80) *p* < 0.01 (Fig. 1).

**Conclusions:** The newly developed score was shown good predictive performance and superior than qSOFA and warning scores with easy and available predictors predicted mortality in ED.

**Figure Figdg:**
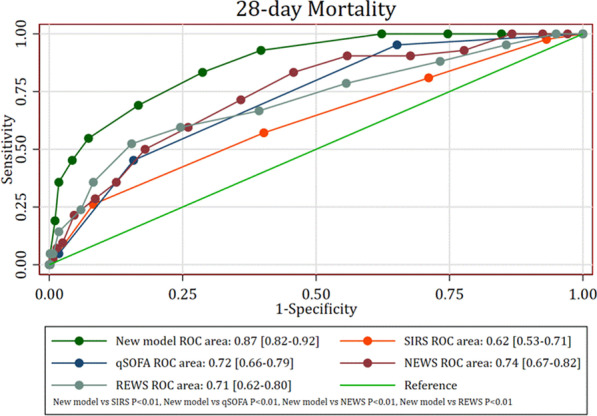
**Fig. 1 (abstract P267)**. ROC curve of the model of prognostic indicators for 28-day mortality compared New model, SIRS, qSOFA, NEWS, Ramathibodi early warning score (REWS)

## P268

### The competency of emergency doctors in managing eye emergencies in NHS emergency departments: a regional survey

#### W Raslan^1^, S Shahid^2^

##### ^1^Whipps Cross University Hospital, Emergency Department, London, UK, ^2^St Andrew’s Centre for Plastic Surgery and Burns, Broomfield Hospital, Chelmsford, UK

*Critical Care* 2023, **27(S1)**: P268

**Introduction:** Patients with eye related complaints often present to general accident and emergency (A&E) departments across the UK. It is therefore crucial that emergency department (ED) doctors feel confident in diagnosing and managing common eye related emergencies. We evaluated how confident ED doctors are in assessing, diagnosing, and treating ocular emergencies.

**Methods:** A previously published questionnaire was revised and published as an online survey [1]. Doctors from three emergency departments from London were contacted for participation.

**Results:** 38 ED doctors completed the survey which included 14 questions to assess accessibility to ophthalmoscope and slit lamps, as well as measuring confidence in managing ophthalmic emergencies in A&E. 81% of participants were senior house officers versus 9% who were registrars and the remaining 10% were consultants. 76% of participants had not had any formal training on ophthalmic emergencies in A&E departments. Despite having accessible ophthalmoscope and slit lamp in their departments, only 53% and 19% felt confident using these devices respectively. Only 11% felt confident enough in dealing with ophthalmic emergencies, whilst 50% felt a little confident, and 39% felt not confident at all.

**Conclusions:** Emergency department doctors lack confidence in diagnosing and managing eye related emergencies, as well as using ophthalmic equipment. This can be linked to a lack of formal training. This study emphasises the importance of providing more education and training on ophthalmological equipment and common ocular emergencies.


**Reference**
Sim PY et al. Eye (Lond). 2020;34:1094–1099.


## P269

### Awareness on workplace violence against healthcare professionals: an emergency medicine perspective

#### H Abbas, UA Mani

##### King George’s Medical University, Emergency Medicine and Critical Care, Lucknow, India

*Critical Care* 2023, **27(S1)**: P269

**Introduction:** Over the last few years, there have been several accounts of the rising incidence of violence against healthcare professionals in the developing world especially in emergency and critical care setting.The issue of violence in healthcare settings as a law and policy issue and its awareness continues to remain relevant.

**Methods:** A questionnaire was answered by 250 medical and paramedical professionals. Questions were designed to assess 4 parameters ie—Basic awareness of violence in emergency services, legal discourse, penal sections doctors should be aware of and its experience and impact it had on working [1,2].

**Results:** Amongst all physicians, residents and nursing officers had experienced some or other form of violence.Healthcare workers who faced verbal and physical altercation 18.7% reportedly had no impact on their mental health, 6.7% had been diagnosed as having clinical depression by a psychiatrist and 74.6% have experienced low mood ranging from a few hours to many days after the episode.Assessing the impact violence and verbal threats have on healthcare workers we noted: 67.6% stated that it did not affect them, 24.3% reported that it affected their outlook towards all patients, 8.1% of health care workers reported that this affected their outlook only towards a particular group of patients/attendants who had a predilection to violence.

**Conclusions:** The awareness of legal aspects on violence against healthcare professionals is low amongst resident physicians, undergraduates and nursing officers. It is recommended to include this topic in their core curriculum or competency assesment.


**References**
Shivakumar DK et al. Indian J Forensic Community Med 2019;6:170–1722.Jambure M et al. Int J Curr Medical Appl Sci 2017;17:09–12.


## P270

### A novel rapid fibrinogen assay based on thrombin generation and turbidity

#### RA Arisz^1^, B Bakker^2^, R Van den Ham^2^, A Ahmed^2^, MPM De Maat^1^

##### ^1^Erasmus MC, University Medical Center Rotterdam, Department of Hematology, Rotterdam, Netherlands, ^2^HEMEO B.V., Amsterdam, Netherlands

*Critical Care* 2023, **27(S1)**: P270

**Introduction:** A rapid point-of-care test to measure fibrinogen levels is crucial to adequately treat patients with major blood loss. Obtaining results from a Clauss assay, the most used laboratory fibrinogen assay, can take up to 60 min; too long in emergency situations. Alternative assays are often costly and time-consuming too. In this study we used turbidity and thrombin generation curves as inputs for a mathematical model of fibrin polymerization to derive the fibrinogen levels in diagnostically challenging patients.

**Methods:** We simultaneously measured the turbidity and thrombin generation in 44 frozen plasma samples, including 29 samples with a PT > 13 s (n = 24), with an anti-Xa > 0 U/ml (n = 14), and/or with a D-dimer > 500 ng/ml (n = 4). From these 29 samples, 8 did not produce an analysable turbidity and/or thrombin generation curve. The model derived fibrinogen levels of the remaining 36 samples were then compared to the fibrinogen levels measured with the Clauss assay (ranging between 1.1 and 16.6 g/l).

**Results:** The one sample with a very high Clauss fibrinogen level of 16.1 g/l could not accurately be determined using our method. Comparison of the predicted and Clauss fibrinogen levels in the other samples resulted in a correlation coefficient of 0.92 (Fig. 1).

**Conclusions:** This feasibility study shows that our method can determine the fibrinogen concentration very well. This method cannot predict fibrinogen levels in plasma samples from patients that generate abnormal clotting curves (e.g., due to high levels of anticoagulants). However, this can be addressed by assay changes. Of note: our mathematical model can continuously learn and improve the accuracy and speed of its fibrinogen predications based on additional measurements. Therefore, we consider this method promising as a fast fibrinogen assay for application in time-critical situations.

**Figure Figdh:**
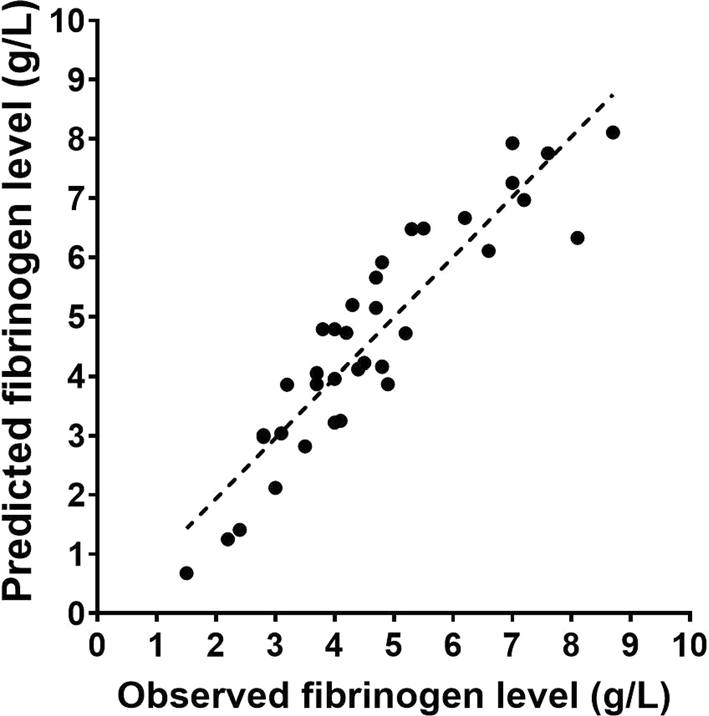
**Fig. 1 (abstract P270)**. Plotted correlation between the predicted fibrinogen levels and the observed fibrinogen levels, measured with the Clauss assay

## P271

### A novel role for ADAMTS13 in trauma-induced hyperfibrinolysis

#### PH Sloos^1^, A Vandenbulcke^2^, K Vanhoorelbeke^2^, NP Juffermans^1^, DJB Kleinveld^1^

##### ^1^Amsterdam UMC, Laboratory of Experimental Intensive Care and Anesthesiology, Amsterdam, Netherlands, ^2^KU Leuven, Laboratory for Thrombosis Research, Kortrijk, Belgium

*Critical Care* 2023, **27(S1)**: P271

**Introduction:** Hyperfibrinolysis after trauma aggravates bleeding and contributes to early mortality. The protease ADAMTS13 regulates Von Willebrand Factor (VWF) multimere size and thereby platelet adhesion to the vascular wall. The specificity of ADAMTS13 for VWF is self-regulated by CUB domains resulting in a closed conformational state. Degradation of its CUB domains by plasmin during hyperfibrinolysis could increase ADAMTS13 activity and result in decreased substrate specificity. Here, we propose a novel mechanism of trauma-induced hyperfibrinolysis. We hypothesise that ADAMTS13 can circulate in an ‘open’ conformation after trauma (= without CUB domains) which is associated with increased ADAMTS13 activity and the presence of hyperfibrinolysis. Additionally, we hypothesise that forcing an open conformation of ADAMTS13 in vitro decreases its specificity for VWF and hence increases tissue plasminogen activator (tPA)-induced fibrinolysis.

**Methods:** In 38 trauma patients with a base deficit of > 5 mM, rotational thromboelastometry (ROTEM) Fibtem and ADAMTS13 activity and conformation were determined. Additionally, 11 blood samples of healthy volunteers were incubated with either an ADAMTS13 opening antibody (17G2) or an inhibiting antibody (3H9) and ROTEM Fibtem was performed in the presence of tPA.

**Results:** Trauma patients had an open ADAMTS13 conformational state in 21% of cases, which was defined as a conformation index > 0.5 (Fig. 1A). An open ADAMTS13 was associated with increased ADAMTS13 activity (B) and hyperfibrinolysis in ROTEM Fibtem (C). An open conformation of ADAMTS13 accelerated tPA-induced fibrinolysis in vitro (D-F).

**Conclusions:** ADAMTS13 can circulate in an ‘open’ and hyperactive conformational state in severely injured trauma patients, which is associated with hyperfibrinolysis. This was confirmed in vitro, where inducing an open conformation of ADAMTS13 accelerated hyperfibrinolysis.

**Figure Figdi:**
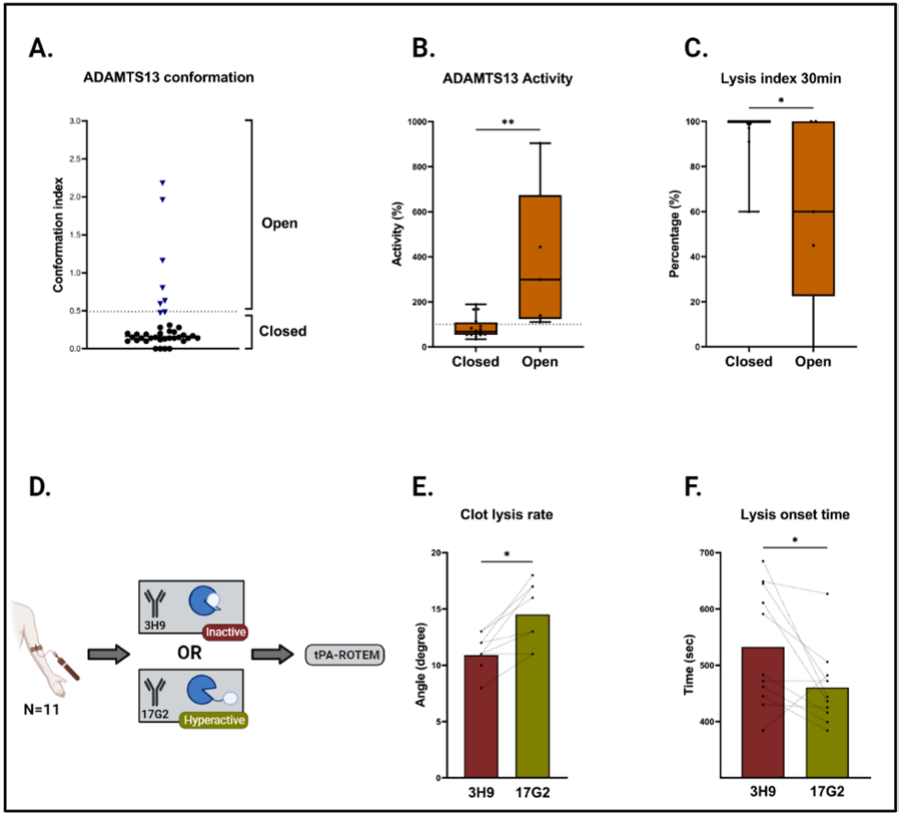
**Fig. 1 (abstract P271)**. **A** ADAMTS13 conformation index > 0.5 is defined as an open conformation.2 **B** ADAMTS13 activity levels in trauma patients with open or closed conformation of ADAMTS13. **C** Lysis index at 30 min in ROTEM Fibtem. **D**, **E**, **F** In healthy volunteers, citrated whole blood was incubated with an ADAMTS13 inhibiting antibody (3H9) or opening antibody (17G2). Thereafter ROTEM Fibtem was performed in the presence of 150 IU/ml tPA

## P272

### Analysis of hyperfibrinolysis detection with ClotPro’s ECA-test

#### I. Zátroch^1^, E. Dinya^2^, J Fazakas^3^

##### ^1^Uzsoki Hospital, Department of Anesthesiology and Intensive Therapy, Budapest, Hungary, ^2^Semmelweis University, Institute of Digital Health Sciences, Budapest, Hungary, ^3^Semmelweis University, Department of Surgery, Transplantation and Interventional Gastroenterology and Department of Anesthesiology and Intensive Therapy, Budapest, Hungary

*Critical Care* 2023, **27(S1)**: P272

**Introduction:** Hyperfibrinolysis is commonly seen during liver transplants due to the reduce of tPA-clearance. Our earlier results showed that fibrinolysis could be detected in more patients and earlier with ECA-test. Our presumption was that this could be attributed to the absence of calcium in the ECA-test, resulting reduce activation of FXIII and TAFI. On the other hand, thrombin generation (TG) is crutial in the formation of the fibrin mesh. The aim of the further analysis was to assess if the kinetics of ecarin-activated TG show any difference when compared to the tissue factor or elagic acid-induced TG, and if this could contribute to distinct sensitivity to fibrinolysis of the ECA-test.

**Methods:** From 25 adult liver transplant recipients blood samples were collected in 5 pre-defined time-points during surgery (n = 125). ClotPro’s IN-test, EX-test, and ECA-test were performed simultaneously from each sample. Hyperfibrinolysis was defined as maximal lysis > 15%. The maximal velocity (MaxV) of TG and AUC, indicative of the total amount of generated thrombin, were calculated from the first derivate of the viscoelastic curve. Independent samples t-test, Kruskal–Wallis test with Bonferroni correction were used to compare these parameters between EX-test and IN-test vs ECA-test in lysis and non-lysis groups.

**Results:** The AUC was significantly lower in the lysis groups of all tests (EX-test *p* = 0.02; IN-test *p* = 0.009; ECA-test *p* = 0.001). However, no statistically significant difference could be detected in terms of MaxV and AUC when TG capability was assessed within the groups with hyperfibrinolysis (MaxV EX vs ECA *p* = 0.774; IN vs ECA *p* = 0.105; AUC EX vs ECA and IN vs ECA *p* = 1).

**Conclusions:** Within the groups showing hyperfibrinolysis, no significant difference could be detected between TG and AUC. These results failed to indicate altered thrombin generation in the ecarin-induced test, which infers that distinct sensitivity to fibrinolysis of the ECA-test cannot be attributed to features of thrombin generation.

## P273

### Comparison of coagulation parameters associated with fibrinogen concentrate and cryoprecipitate for treatment of bleeding in patients undergoing major cytoreductive surgery: results from a randomised, controlled phase 2 study

#### A Roy^1^, S Stanford^1^, C Solomon^2^, S Knaub^2^, F Mohamed^1^

##### ^1^Basingstoke and North Hampshire Hospital, Basingstoke, UK, ^2^Octapharma AG, Lachen, Switzerland

*Critical Care* 2023, **27(S1)**: P273

**Introduction:** This *post-hoc* analysis of the FORMA-05 study explored coagulation parameters in patients undergoing cytoreductive surgery for pseudomyxoma peritonei (PMP) who received human fibrinogen concentrate (HFC) versus cryoprecipitate (cryo) for maintaining haemostasis. This analysis focussed on seven patients on cryo who developed thromboembolic events (TEEs).

**Methods:** FORMA-05 was a prospective, randomised, controlled Phase 2 study. Patients undergoing PMP surgery with predicted blood loss ≥ 2 L received HFC (4 g) or cryo (2 pools of 5 units), repeated as needed. Plasma coagulation factor levels, EXTEM A20, FIBTEM A20 and endogenous thrombin potential (ETP) were measured perioperatively.

**Results:** Patients were randomised to receive cryo (N = 23) or HFC (N = 21). Fibrinogen, platelet count, factors XIII and VIII, von Willebrand factor (VWF), EXTEM/FIBTEM A20, and ETP were maintained throughout surgery in both groups. No patients in the HFC group developed TEEs. Two patients in the cryo group developed deep vein thromboses (DVT), and appeared to have a procoagulant status preoperatively, with higher fibrinogen level (Fig. 1A), FIBTEM A20, and platelet levels which persisted perioperatively. The five patients on cryo who developed pulmonary embolism (PE) showed a disproportionate increase in VWF levels intraoperatively (post-cryo administration) which was retained postoperatively (Fig. 1B).

**Conclusions:** Patients treated with HFC versus cryo showed broad overlaps in coagulation parameters; however, patients who developed PE experienced a disproportionate rise in VWF following cryo administration, while patients who developed DVT displayed a procoagulant status before and following surgery. Preoperative testing may allow identification of these patients and thus minimise risk of TEEs.

**Figure Figdj:**
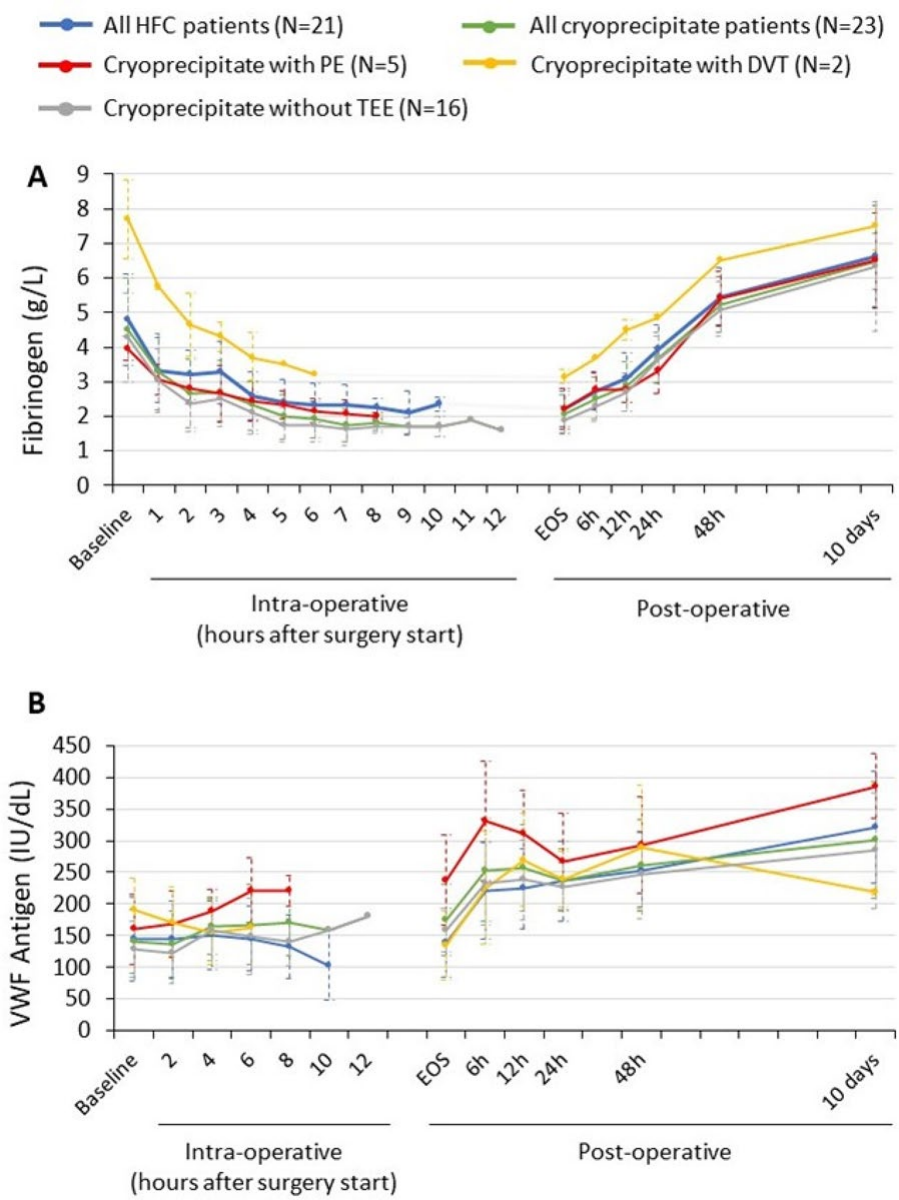
**Fig. 1 (abstract P273)**. Intra- and postoperative changes in **A** plasma fibrinogen and **B** von Willebrand factor (VWF) antigen, stratified by occurrence of thromboembolic events

## P274

### Long-term safety of a fibrinogen concentrate (RiaSTAP®/Haemocomplettan® P): analysis of more than 35 years of pharmacovigilance data

#### N Rahe-Meyer^1^, G Neumann^2^, J Whitten^3^

##### ^1^Franziskus Hospital Bielefeld, Department for Anesthesiology and Intensive Care Medicine, Bielefeld, Germany, ^2^CSL Behring Innovation GmbH, Marburg, Germany, ^3^CSL Behring LLC, King of Prussia, PA, USA

*Critical Care* 2023, **27(S1)**: P274

**Introduction:** Fibringen concentrate is recommended for both congenital and acquired fibrinogen deficiency caused by various clinical conditions, including major bleeding and cardiac surgery. However, thromboembolic events (TEEs) post-treatment remains a concern. Here, we review pharmacovigilance data and published studies of a fibrinogen concentrate (RiaSTAP®/Haemocomplettan® P) to assess its safety profile.

**Methods:** A retrospective evaluation of postmarketing pharmacovigilance data of RiaSTAP®/Haemocomplettan® P between January 1986–June 2022 was performed. This was complemented by a review of clincal studies published in the same period.

**Results:** Commercial data indicated that 7,449,797 g of RiaSTAP®/Haemocomplettan® P were distributed over the pharmacovigilance period, corresponding to 1,862,449 standard doses of 4 g each. A total of 806 adverse drug reactions (ADRs) in 337 cases were reported (~ 1 per 22,106 g or 5527 standard doses). Of these, 81/337 (24.0%) with 114 possible TEEs were reported (~ 1 per 91,973 g or 22,933 standard doses), of which 14/81 (17.3%) were fatal, although in most of these cases additional risk factors existed. A total of 79 possible hypersensitivity reaction events were identified from 47 cases (13.9% of all cases; ~ 1 per 158,506 g or 39,627 standard doses). Fifty-one possible events of transmission of infectious agents were reported in 33 cases (9.8% of all cases; ~ 1 per 225,751 g or 56,438 standard doses). All were considered by the company as unlikely to be causally related to the product due to alternative explanations, non-reactive polymerase chain reaction tests for the concerned batches, and/or related plasma pools for fractionation. The published literature showed a similar safety profile.

**Conclusions:** Pharmacovigilance data and clinical studies revealed that treatment with fibrinogen concentrate is associated with few ADRs and a low TEE rate across indications. Overall, fibrinogen concentrate showed a favorable safety profile.

## P275

### Long-term safety of a four-factor prothrombin complex concentrate (Kcentra®/Beriplex® P/N): an updated pharmacovigilance study

#### T J Milling^1^, A Voronov^2^, J Whitten^3^, D Schmidt^4^, E Lindhoff-Last^5^

##### ^1^Seton Dell Medical School Stroke Institute, Dell Medical School, University of Texas at Austin, Departments of Neurology and Surgery and Perioperative Care, Austin, TX, USA, ^2^CSL Behring Australia Pty Ltd, CSL Behring Australia Pty Ltd, Broadmeadows, Australia, ^3^CSL Behring LLC, CSL Behring LLC, King of Prussia, PA, USA, ^4^CSL Behring GmbH, Marburg, Germany, ^5^CCB Vascular Center, CCB Coagulation Center, Cardiology Angiology Center Bethanien, Frankfurt, Germany

*Critical Care* 2023, **27(S1)**: P275

**Introduction:** Four-factor prothrombin complex concentrate (4F-PCC) is indicated for urgent vitamin K antagonist reversal in patients with major bleeding or in need of surgery (Kcentra®), and treatment of bleeding in acquired deficiency of the prothrombin complex coagulation factors and congenital deficiency of any of the vitamin k-dependent coagulation factors (Beriplex®). This study evaluates the safety profile of a 4F-PCC (Kcentra®/Beriplex® P/N) by reviewing pharmacovigilance data and published studies.

**Methods:** A retrospective analysis of postmarketing pharmacovigilance data of Kcentra®/Beriplex® P/N from February 1996 to April 2022 was performed and complemented by a review of published studies in the PubMed database.

**Results:** A total of 1236 adverse drug reactions (ADRs) in 614 cases were reported from postmarketing sources (Table 1) (approximately 1 case per 3781 standard infusions). A total of 233 cases (38% of all cases) describing 314 suspected thromboembolic events (TEEs) related to 4F-PCC were reported (approximately 1 case per 9963 standard infusions). Analysis of the TEEs revealed that most cases had pre-existing or concomitant conditions likely to be significant risk factors for thrombosis. Fifty possible related anaphylaxis and hypersensitivity/allergic reactions were identified from 36 cases (5.9% of all cases) (approximately 1 case per 64,485 standard infusions). A single case of heparin-induced thrombocytopenia related to 4F-PCC use was reported but could not be assessed due to insufficient data. No confirmed case of viral transmission related to 4F-PCC use was reported. The published literature revealed a similar safety profile of 4F-PCC.

**Conclusions:** Analysis of postmarketing pharmacovigilance safety reports demonstrated that treatment with 4F-PCC was associated with few ADRs and a low rate of TEEs across multiple indications and settings, thus confirming a positive safety profile of 4F-PCC.

**Table Tabcw:** **Table 1 (abstract P275)**. Pharmacovigilance data of Kcentra®/Beriplex® P/N recorded for the period February 1996–April 2022

	Kcentra®/Beriplex® P/N
Amount manufactured (IU)	5,803,608,053
Estimated standard applications*	2,321,443
Cases reported from post-marketing surveillance	614
Suspected cases of thromboembolic events**	233
Suspected virus transmissions	0
Suspected hypersensitivity reactions	36
Suspected cases of HIT	1

*Considering a standard infusion of 2500 IU **Possibly related to Kcentra®/Beriplex® P/N (sources: spontaneous report and case reports from the scientific literature, non-interventional post-authorization studies) HIT, heparin-induced thrombocytopenia IU, international unit

## P276

### Double-blind, randomised comparison of two four-factor prothrombin complex concentrates for vitamin K antagonist reversal before urgent surgery with significant bleeding risk

#### R Sarode^1^, J Goldstein^2^, G Simonian^3^, T Milling Jr^4^

##### ^1^University of Texas Southwestern Medical Center, Dallas, USA, ^2^Department of Emergency Medicine, Massachusetts General Hospital, Harvard Medical School, Boston, Massachusetts, USA, ^3^Division of Vascular Surgery, Heart and Vascular Hospital, Hackensack University Medical Center, Hackensack, New Jersey, USA, ^4^Departments of Neurology and Surgery and Perioperative Care, Seton Dell Medical School Stroke Institute, Dell Medical School, University of Texas, Austin, Texas, USA

*Critical Care* 2023, **27(S1)**: P276

**Introduction:** The Phase 3 LEX-209 study compared an investigational four-factor prothrombin complex concentrate (4F-PCC; Octaplex®, Octapharma) for non-inferiority with the control 4F-PCC (Beriplex® P/N, CSL Behring) for rapid vitamin K antagonist (VKA) anticoagulation reversal before urgent surgery.

**Methods:** Patients were ≥ 18 years, on VKA, had International Normalised Ratio (INR) ≥ 2.0 and needed surgery with a significant bleeding risk (≥ 50 ml). A 4F-PCC dose of 25, 35 or 50 international units (IU)/kg body weight was given for baseline INR of 2– < 4, 4–6 or > 6. Primary endpoint was hemostatic efficacy at surgery end, objectively assessed by a blinded Independent Endpoint Adjudication Board. Other endpoints were INR ≤ 1.5, coagulation factor levels and thromboembolic events (TEEs).

**Results:** Patients were randomised to investigational (N = 105) or control (N = 103) 4F-PCC. Baseline characteristics were similar between groups. Median dose was 25 IU/kg for both groups: median (range) infusion time 12 (8–50) min for investigational; 13 (7–30) min for control. For both 4F-PCCs, the intravenous infusion rate was 0.12 ml/kg/minute (~ 3 IU/kg/minute), up to a maximum of 8.4 mL/minute (~ 210 IU/minute). Investigational 4F-PCC was non-inferior to control for effective hemostasis (Fig. 1). INR was ≤ 1.5 at 30 min post-infusion in 78.1% vs. 71.8% patients (proportion difference 0.063; 95% CI − 0.056, 0.181). Mean activities of factors II, VII, IX and X and proteins C and S increased similarly in both groups by 30 min post-infusion. In ≤ 21 days post-surgery, two TEEs occurred, both in the investigational group, one of which (unstable angina on day 5) was considered possibly related to treatment. One death occurred ≤ 21 days post-surgery; this was in the control group and was deemed unrelated to study drug.

**Conclusions:** Investigational 4F-PCC was hemostatically non-inferior to control 4F-PCC for VKA reversal in urgent surgeries with significant bleeding risk, with a similar safety profile.

**Figure Figdk:**
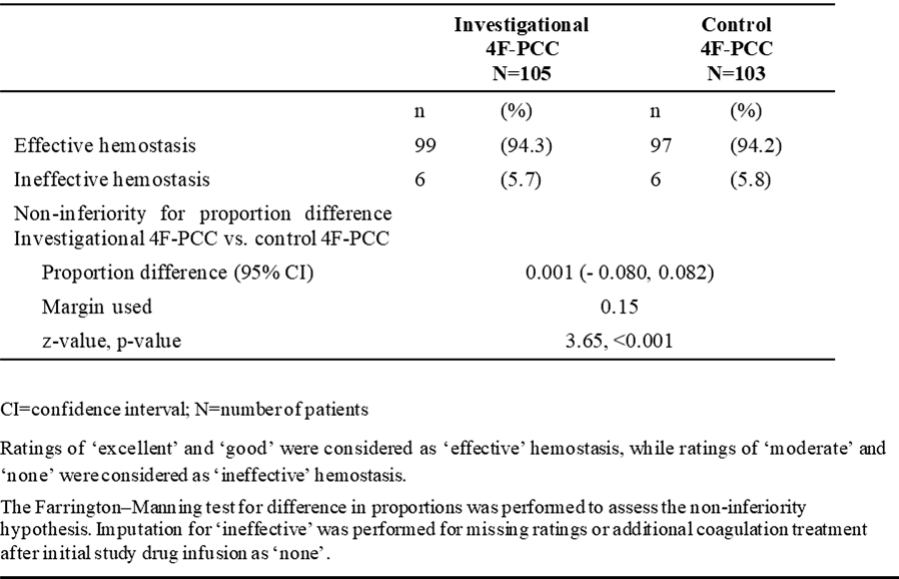
**Fig. 1 (abstract P276)**. Final analysis of the primary endpoint: hemostatic efficacy at the end of surgery as assessed by the Independent Endpoint Adjudication Board (randomized population, N = 208)

## P277

### Efficacy & safety of 3 factors prothrombin complex concentrate (3FPCC) in infants BW < 5 kg & neonates underwent congenital cardiac surgery with refractory bleeding after CPB: an exploratory retrospective propensity score matched study

#### S Kongsayreepong^1^, N Raykateeraro^1^, T Subtaweesin^2^

##### ^1^Siriraj hospital, Mahidol University, Department of Anesthesiology & Critical Care, Bangkok, Thailand, ^2^Siriraj Hospital, Mahidol University, Division of Cardiothoracic Surgery, Department of Surgery, Bangkok, Thailand

*Critical Care* 2023, **27(S1)**: P277

**Introduction:** Refractory bleeding after CPB was associated with poor outcome.PCC was off-label used for refractory bleeding in adult cardiac surgery. Urgent information is needed infants & neonates undergoing congenital cardiac surgery. The aim of this study was to determine the efficacy & safety of PCC in infants (BW < 5 kg) & neonates underwent congenital cardiac surgery with CPB who had refractory bleeding after CPB despite prophylaxis TXA, complete reversal of protamine & conventional coagulation factors.

**Methods:** A retrospective study of infants (BW < 5 kg) & neonates underwent congenital cardiac surgery with CPB between Jan 2015- Mar 2022 (n = 142). Pts with hx of thrombosis, stroke, seizure, AKI, bleeding disorder were excluded. 3 FPCC (Profinine®) was given in refractory bleeding after CPB. Demographic data, RACHS score, type & duration of surgery, CPB data, amount of TXA & PCC, postoperative bleeding, allogeneic transfusion, re-exploration, ECMO support, incidence of thrombosis, stroke, MI, stroke, CKD, ventilators days, ICU & hospital LOS, in hospital mortality were recorded.

**Results:** As baseline characteristic of pts in the TXA plus PCC gr.were significantly different from the only TXA gr. A propensity score matching was applied to reduce the impact of different baseline characteristic between pts received only TXA (n = 39) & TXA plus PCC (n = 17). The result found that TXA plus PCC gr. had significantly less PO bleeding at 12 h [8.3 (0, 18.4) vs 16.7 (4.2, 37.7) ml/kg, *p* = 0.019] & bleeding all [21.3 (11.6, 30.9) vs 46.2 (26.7, 71.2) ml/kg, *p* = 0.001]. Pts in the TXA plus PCC had a tendency to have less significant bleed at 6 h & PO allogeneic transfusion. No significantly different in ventilator day, ICU & hospital LOD. No pt in the TXA plus PCC gr needed ECMO or mortality.

**Conclusions:** 3 FPCC had shown the efficacy in significantly decrease postoperative bleeding & a tendency to decrease postoperative allogeneic transfusion without incidence of thrombosis. Large randomized control trial is needed.

## P278

### LEX-211: a phase 3, active-control, randomised study of four-factor prothrombin complex concentrate versus frozen plasma in bleeding adult cardiac surgery patients

#### C Solomon^1^, K Karkouti^2^, J Callum^3^, K Tanaka ^4^, D Grewal, S Knaub^6^, J Levy^7^

##### ^1^Octapharma AG, Clinical R&D Haematology, Lachen, Switzerland, ^2^University of Toronto, Toronto, Canada, ^3^Queen’s University, Kingston, Ontario, Canada, ^4^University of Oklahoma, Oklahoma, USA, ^5^University Health Network, Toronto, Canada, ^6^Octapharma AG, Lachen, Switzerland, ^7^Duke University, Durham, North Carolina, USA

*Critical Care* 2023, **27(S1)**: P278

**Introduction:** The LEX-211 (FARES-2) study will determine if four-factor prothrombin complex concentrate (4F-PCC, Octaplex®, Octapharma) is clinically non-inferior to frozen plasma (FP) in terms of haemostatic effectiveness in cardiac surgery patients requiring coagulation factor replacement. Cardiac surgery is often complicated by coagulopathic bleeding, leading to transfusion and poor outcomes.

**Methods:** LEX-211 will include patients ≥ 18 years old undergoing cardiac surgery with cardiopulmonary bypass (CPB) who require coagulation factor replacement due to bleeding and known/suspected coagulation factor deficiency. Exclusion criteria are heart transplant, insertion/removal of ventricular assist devices, high probability of death within 24 h, severe right heart failure, heparin contraindications, thromboembolic event within 3 months and IgA deficiency. Patients will be randomised to PCC or FP, with weight-based doses shown in Fig. 1. The primary endpoint is the haemostatic response to PCC vs. FP, rated ‘effective’ if no further haemostatic intervention (haemostatic agents or surgical re-opening for bleeding) is required 60 min–24 h after the first dose. Secondary endpoints include global haemostatic response (60 min–24 h), bleeding (24 h), blood product/coagulation factor use (24 h, 7 days), surgical re-exploration (24 h) and coagulation parameters (~ 1 h post-treatment). Safety endpoints include treatment-emergent adverse events (e.g., thromboembolic events), ICU stay and mortality (30 days). An unblinded interim analysis (100 evaluable patients per group) will test the sample size assumptions and re-estimate if necessary.

**Results:** LEX-211 has started with the first sites initiated in Q4 2022. Completion is expected Q4 2024.

**Conclusions:** The results of this study will inform clinical practice for bleeding cardiac surgery patients requiring coagulation factor replacement, potentially reducing blood product usage and improving outcomes.

**Figure Figdl:**
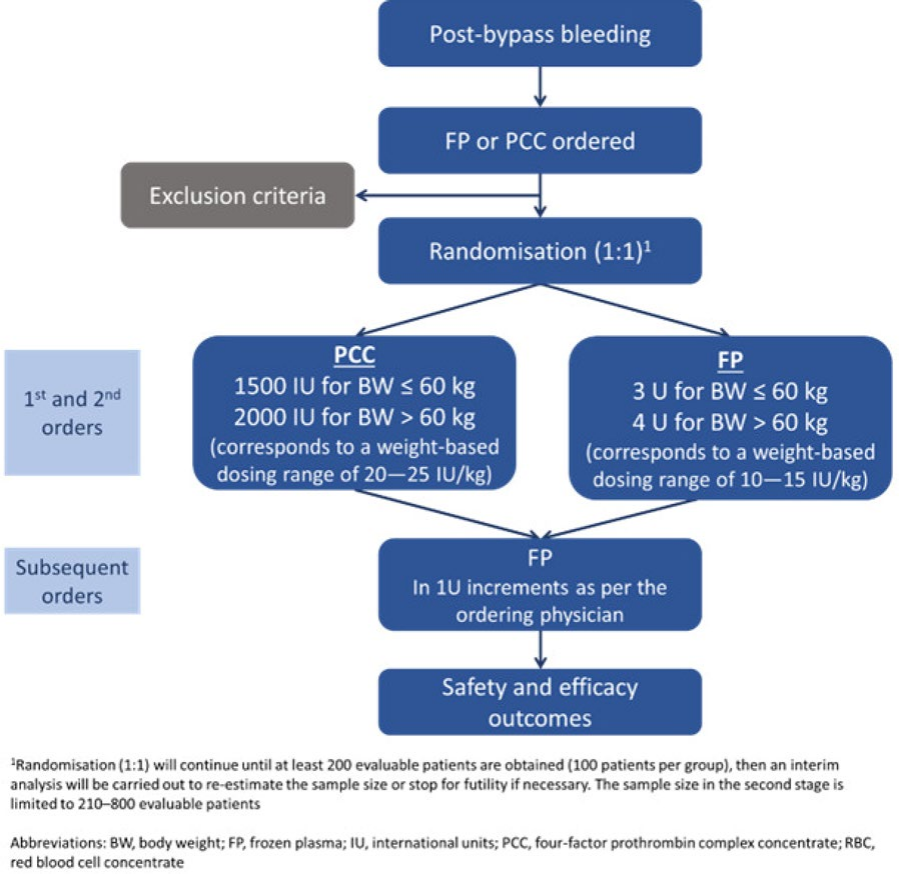
**Fig. 1 (abstract P278)**. Study flow

## P279

### Accuracy of non-invasive hemoglobin level measurement in the emergency department

#### Z Al Aseri

##### King Saud University and Dar Al Uloom University, ED & ICU, Riyadh, Saudi Arabia

*Critical Care* 2023, **27(S1)**: P279

**Introduction:** Hemoglobin measurement, along with physical examination, is important for the diagnosis of acute anemia [1–3]. We aimed to evaluate the accuracy of non-invasive hemoglobin (SpHb) measurement in comparison with serum hemoglobin measurement in the emergency department.

**Methods:** We compared the hemoglobin measurements by both methods in all the patients who visited the emergency department of King Saud University Medical City and required complete blood count analysis from March 2022 to May 2022. We used Pearson correlation (r) and Bland–Altman analysis to test the correlation, bias, and limits of agreement between SpHb and laboratory hemoglobin (Lab Hb) measurements.

**Results:** In this prospective observational study, mean of SpHb measurements of the patients was 12.15 ± 1.9 g/dl, whereas mean of Lab Hb measurements was 12.29 ± 2.39 g/dl. We found a significant correlation between SpHb and Lab Hb (r = 0.812, *p* < 0.01) levels (Fig. 1). The Bland–Altman analysis showed excellent accuracy with moderate limits of agreement (mean bias [limits of agreement]: 0.146 [−2.58 and 2.87] g/dl).

**Conclusions:** SpHb levels showed acceptable accuracy and excellent correlation with Lab Hb levels; therefore, SbHb measurement could be an easy, feasible, inexpensive, and accurate solution for Hb measurement in the emergency department.

**Figure Figdm:**
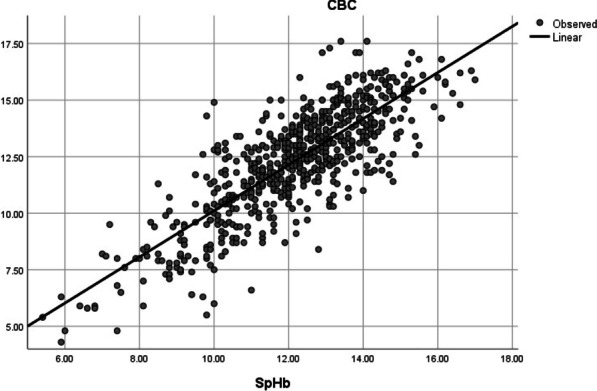
**Fig. 1 (abstract P279)**. Linear regression curve showing a highly significant correlation between SpHb and LabHb

## References


Janz TG et al. Emerg Med Pract. 2013;15:1–15.Rui P et al. National Hospital Ambulatory Medical Care Survey: 2014. U.S. Department of Health and Human Services, Centers for Disease Control and Prevention, National Center for Health Statistics; 2014:34.Bensenor IM et al. Sao Paulo Med J. 2007;125:170–173.

## P280

### Rotational thromboelastometry profiles have the potential to diagnose disseminated intravascular coagulation in the critically ill

#### RWG Dujardin^1^, MCA Müller^1^, CS Witbreuk^1^, BMP Rademaker^2^, ND Nielsen^3^, NP Juffermans^4^

##### ^1^Amsterdam UMC, Intensive Care, Amsterdam, Netherlands, ^2^OLVG Hospital, Anesthesiology, Amsterdam, Netherlands, ^3^University of New Mexico School of Medicine, Intensive Care, Albuquerque, USA, ^4^OLVG Hospital, Intensive Care, Amsterdam, Netherlands

*Critical Care* 2023, **27(S1)**: P280

**Introduction:** This study aims to investigate the potential of rotational thromboelastometry (ROTEM) profiles for the diagnosis of disseminated intravascular coagulation (DIC) in at risk critically ill patients.

**Methods:** Interim analysis of a prospective observational study conducted in the Intensive Care Unit (ICU) of two large teaching hospitals in Amsterdam, the Netherlands, between January 2021 and September 2022. Adult patients with a risk factor for DIC (severe infection, trauma, malignancy, obstetric complication or pancreatitis) and a thrombocytopenia < 150 × 10^9^/L were eligible for inclusion. ROTEM profiles of the EXTEM, INTEM and FIBTEM tracings of clotting time (CT), clot formation time (CFT), clot amplitude at 5 min (CA5), maximal clot firmness (MCF) and maximum lysis (ML) were measured once between day 1–4 of ICU stay. A DIC diagnosis was defined as an International Society on Thrombosis and Haemostasis DIC score of ≥ 5. ROTEM EXTEM parameters with a statistically significant difference between DIC and non-DIC patients were selected for construction of receiver operating characteristics (ROC) curves with subsequent calculation of the area under the curve (AUC).

**Results:** Of 55 included patients, 15 (27.3%) developed DIC. Compared to non-DIC patients, DIC patients had a statistically significant prolonged EXTEM CT with a concomitant decrease in EXTEM CA5 and EXTEM MCF, EXTEM ML was inhibited in DIC (Table 1). The results of the INTEM and FIBTEM tracings were similar. Subsequent ROC curve analysis demonstrated that for the diagnosis of DIC, EXTEM CA5 had the highest diagnostic performance with an AUC of 0.90 (95% CI 0.81–0.99), followed by EXTEM MCF with an AUC of 0.87 (95% CI 0.77–0.97).

**Conclusions:** Patients with DIC have a hypocoagulable and hypofibrinolytic ROTEM profile compared to critically ill risk patients without DIC. Furthermore, EXTEM CA5 and EXTEM MCF have a high diagnostic performance to discriminate between patients with and without DIC.

**Table Tabda:** **Table 1 (abstract P280)**. Results

EXTEM parameter	Whole cohort (N = 55)	DIC (N = 15)	No DIC (N = 40)	*p* value
CT (s)	76 (68–90)	89 (76–102)	71 (67–82)	0.004
CFT (s)	65 (46–92)	91 (63–135)	56 (43–78)	0.005
CA5 (mm)	46 (39–50)	37 (27–40)	48 (43–52)	< 0.001
MCF (mm)	62 (60–69)	58 (47–60)	65 (61–70)	< 0.001
ML (%)	2 (0–5)	0 (0–2)	2 (0–6)	0.015

*CT* clotting time, *CFT* clot formation time, *CA5* clot amplitude at 5 min, *MCF* maximum clot formation, *ML* maximum lysis

## P281

### Thromboelastographic characteristics in patients admitted to the ICU in the immediate postoperative period and their relationship with bleeding in the first 48 h.

#### EJ Marcano Millan^1^, N Albalá^1^, M Bueno Bueno^1^, N Núñez Blanco^1^, J Figueroa Falconi^1^, C Obando Martínez^1^, JF Granados Ricaurte^1^, M Paz^1^, J Robledo^1^, V Sagredo Meneses^2^

##### ^1^Hospital Universitario de Salamanca, Critical Care Department, Salamanca, Spain, ^2^Hospital Universitario de Salamanca, Salamanca, Spain

*Critical Care* 2023, **27(S1)**: P281

**Introduction:** This study aims to determine the thromboelastographic characteristics of patients admitted to a surgical ICU and their potential relationship with bleeding in the first 48 h after admission. The introduction of thromboblastography (TEG) in recent years to the diagnostic arsenal in intensive care units has allowed us to advance in bedside assessment of the coagulation status of our patients. TEG has allowed us to guide resuscitation in bleeding patients by providing the necessary data to dispense with other more subjective interpretations.

**Methods:** A prospective observational study was performed. During a 4-month period, measurements were taken in 40 postoperative patients scheduled for cardiac surgery, abdominal surgery and neurosurgery. The TEG 6sc thromboelastograph (Haemonetics®) was used to analyze the samples. Blood samples were obtained within the first half hour of admission. Subsequently, clinical and analytical follow-up was performed over the following 48 h to detect the appearance of possible significant bleeding episodes. Significant bleeding was defined as: anemization > 2 g/dL of hemoglobin in less than 6 h, or bleeding episodes requiring red cell concentrate transfusion or initiation of support with vasoactive drugs.

**Results:** Of the 40 cases studied, 55% (22/40) were men and the incidence of bleeding was 10% (4/40). Thromboelastographic data were recorded for the "bleeding" and "non-bleeding" groups (summarized in Table 1) and the data were subsequently analyzed with nonparametric *U*-Mann–Whitney tests without finding statistically significant differences between the two groups.

**Conclusions:** The results obtained suggest that TEG in our study follows the trend already published in many other studies in which the high negative predictive value in relation to bleeding stands out. However, the size of the sample, the heterogeneity of the groups and the fact that we did not consider hemostasis-modifying drugs prior to admission are very important limitations of this analysis.

**Table Tabdb:** **Table 1 (abstract P281)**. Main thromboelastographic characteristics of the study population, including heparinase clotting reaction (CKH_R) and activated clotting time (ACT) studies as reference

	Bleeding (n = 4)	No_bleeding (n = 36)	Normal Value	*p*
CK_R	7.27	7	(4.6–9.1 min)	0.845
CK_K	2.3	1.5	(0.8–2.1 min)	0.346
CK_ANG	64.1	70.4	(64–78 grad)	0.445
CK_MA	52.75	58.5	(52–69 mm)	0.648
CK_LY30	0	0	(0–2.6%)	1
CKH_R	6.95	6.05	(4.3–8.3 min)	0.262
ACT	144	125.3	(82–152 seg)	> 0.05

## P282

### Use of viscoelastic tests in the intensive care unit for the evaluation of haemostasis in SARS-CoV-2 infection

#### T Seara Sevivas^1^, I Santos^2^, J Pedro Alves^2^, J Luiz Luzio^2^, J Mariano Pego^3^, F Caramelo^4^, A Paiva^5^, P Martins^2^

##### ^1^Department of Blood and Transfusion Medicine, Coimbra Hospital and Universitary Centre, Coimbra, Portugal, ^2^Coimbra Hospital and Universitary Centre, Department of Intensive Care, Coimbra, Portugal, ^3^Department of Clinical Pathology, Coimbra Hospital and Universitary Centre, Coimbra, Portugal, ^4^Department of Statistics, Faculty of Medicine of Coimbra, Coimbra, Portugal, ^5^Flow Citometry Unit, Coimbra Hospital and Universitary Centre, Coimbra, Portugal

*Critical Care* 2023, **27(S1)**: P282

**Introduction:** COVID-19 coagulopathy is associated with poor prognosis and a state of coexisting "hypercoagulopathy" (HyperC) and “hypofibrinolysis”, only detected by viscoelastic tests (VET). VET technology has been useful in areas where conventional tests are inadequate, such as screening for HyperC, thrombotic risk assessment and systemic anticoagulants’ effect. We aim to characterize the evolution profile of coagulopathy in patients with COVID-19 infection during their intensive care unit (ICU) stay.

**Methods:** Consecutive recruitment of adult COVID-19 patients admitted to our hospital’s ICU, during a 6 months period. Patients with thrombosis in the previous 3 months, pregnancy, under hormone therapy, and congenital coagulopathies were excluded. VET were executed every 5 days, at discharge and in complications and all of them were under low weight molecular heparin (LMWH) therapy. Group 1 (G1), n = 24—less than 10 days in ICU and group 2 (G2), n = 16—more than 10 days in ICU. In G1 there was 1 death (day 3) and in G2 there were 5 deaths (between days 15 and 42). We focused current analysis on VET- Rotem® parameters (see Fig. 1).

**Results:** Prognostic scores APACHE II, SAPS II and SOFA were higher in G2, but surprisingly G1 patients are more obese. G2 patients had shorter aPTT and lower platelets. The variables CT_HepTem and MCF_Extem-MCF_Fib_Tem present a greater difference between groups, but no statistical significance. We observed an initial correlation between basophils number (which is lower) on CT Intem and CT HepTem, lost as progression to cure, probably due to cytoplasm heparin granules. As expected, VET were in accordance with HyperC: short CTs, increased MCFs, and decreased lysis.

**Conclusions:** We expected to guide/adjust LMWH dosage, using Rotem® profiles, however these were not corrected by LMWH, used transversally, and remained unchanged in all patients during their stay in ICU.

**Figure Figdn:**
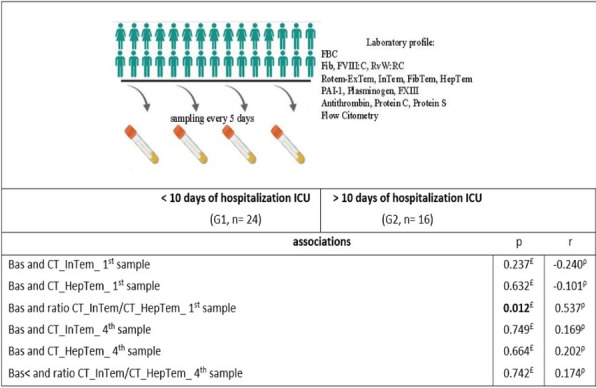
**Fig. 1 (abstract P282)**. VET- Rotem® parameters. FBC-Full Blood Count; Fib-Fibrinogen; FVIII- Factor VIII; FvW:RC-von Willebrand Factor Activity; PAI-1-Plasminogen Activator Inhibitor; FXIII- Factor XIII; Bas- Basophils

## P283

### The laboratory parameters-derived CoLab-score as an indicator of the host response in ICU COVID-19 patients decreases over time: the Maastricht intensive care COVID cohort

#### TJG Schoenmakers^1^, MPG Leers^2^, SHM Gorissen^2^, IHM Van Loo^3^, F Van Rosmalen^4^, WPHG Verboeket-van de Venne^2^, PFG Wolffs^3^, WNKA Van Mook^4^, BCT Van Bussel^4^

##### ^1^Zuyderland Medical Center, Clinical Chemistry and Hematological Department, Sittard-Geleen, Netherlands, ^2^Zuyderland Medical Center, Department of Clinical Chemistry & Hematology, Sittard-Geleen, Netherlands, ^3^Maastricht UMC+, Department of Medical Microbiology, Maastricht, Netherlands, ^4^Maastricht UMC+, Department of Intensive Care, Maastricht, Netherlands

*Critical Care* 2023, **27(S1)**: P283

**Introduction:** The CoLab-score was originally developed and validated to rule out COVID-19 in suspected patients presenting in the emergency department [1, 2]. The CoLab-score includes the patient’s age and ten blood parameters, reflecting the host response to SARS-CoV-2 infection. Here, we investigated the CoLab-score over time in mechanically ventilated COVID-19 patients at the ICU. We hypothesized that the CoLab-score will decrease over time, independent of survival, disease severity and pandemic periods. This would create the opportunity to monitor COVID-19 patients and potentially ruling out the need for isolation when the host response decreases and the infection is overcome.

**Methods:** We used serial data of the Maastricht Intensive Care Covid (MaastrICCht) cohort of mechanically ventilated COVID-19 patients to investigate the association between time and daily CoLab-score using linear-mixed models. Crude models were adjusted for sex, APACHE II score, SOFA score, and stratified for intensive care mortality.

**Results:** 324 patients (73% men), aged 64 ± 12 years with 5959 daily CoLab-scores, were included. CoLab-score decreased with 0.31 points per day (95% CI − 0.33 to − 0.28). Adjustment for sex, APACHE II and stratification for mortality did not change this result.

**Conclusions:** The CoLab-score decreased over time in mechanically ventilated ICU COVID-19 patients, with a point reduction per three days. This suggests that the CoLab-score eventually decreases to a normal state, reflecting a host response that has overcome infection. Future investigation is warranted to assess whether the need for isolation can be ruled out based on the CoLab-score.


**References**
Arjen-Kars B et al. BMJ Open 2022;12:e059111.Leers MPG et al. PLoS One 2022;17:e0270548.


## P284

### A retrospective cohort study on the effect of the low-molecular weight heparin (LMWH) nadroparin dose on anti-Xa levels in a mixed medical-surgical ICU population: clot-Xa

#### L Van Berkel, M Kuindersma, I Van Iperen, PE Spronk

##### Gelre hospitals, Intensive Care, Apeldoorn, Netherlands

*Critical Care* 2023, **27(S1)**: P284

**Introduction:** Patients admitted to the intensive care unit (ICU) are at high risk for a venous thromboembolism (VTE), even despite LMWH prophylaxis. This increased risk can be partly explained by ICU specific coagulopathy through inflammation induced by sepsis and other causes. We hypothesize that the increased incidence of VTE could be partly explained by insufficient dosing of LMWH to antagonize this coagulopathy with associated low anti-Xa levels. The primary aim of this retrospective study is to describe the anti-Xa levels in critically ill patients treated with different LMWH doses. Secondary aim is to provide data on the percentage of patients in target of anti-Xa levels with (guideline) dose therapy.


**Methods:**


*Study design*: A retrospective non-randomized study to evaluate the effect of prophylactic nadroparin dose on anti-Xa levels in a mixed medical-surgical population.

*Study population*: Patients ≥ 18 years with an ICU stay > 48 h who received at least three subcutaneous injections of nadroparin and for whom at least one anti-Xa measurement was available.

*Measurements*: Age, gender, ICU length of stay, admission diagnosis, nadroparin doses and dose adjustments, anti-Xa levels, haemoglobin, platelet count, APTT, PT, APACHE III score, SAPS score.

**Results:** 344 patients were included. Median age was 66 years, and 34% of all patients were female. Median length of ICU stay was 10 days. In hospital mortality was 21%. Nadroparin 2850 IE daily was given 1257 times in 158 patients. Nadroparin 5700 IE daily was given 811 times in 149 patients. Median anti-Xa levels after nadroparin 2850 IE/day were 0.1 U/L (IQR 0.1–0.2 U/l), compared to 0.3 U/l (IQR 0.2–0.4 U/l) after nadroparin 5700 IE/day. Nadroparin 2850 IE/day led to adequate prophylactic anti-Xa levels in 39% of measurements, compared to 83% with nadroparin 5700 IE/day.

**Conclusions:** Nadroparin 5700 IE/day leads to more adequate prophylactic anti-Xa levels compared to 2850 IE/day in critically ill patients.

## P285

### Anti-Xa guided twice daily enhanced anticoagulation for severe COVID-19 patients in intensive care unit setting: outcomes, incidence of pulmonary embolism and complications

#### RE Elsheikh^1^, GB Dani Benvenutti^2^, AP Pambouka^2^, LK Kenny^2^, LO Orlando^2^, NG Ganatra^2^, SA Audet^2^, AD Dushianthan^2^, RC COVID-19 Investigators^2^

##### ^1^Southampton General Hospital, Intensive Care Unit, Southampton, UK, ^2^Southampton General Hospital, Southampton, UK

*Critical Care* 2023, **27(S1)**: P285

**Introduction:** Pulmonary embolism (PE) is a major cause of intensive care unit (ICU) mortality and morbidity [1]. Optimal venous thromboembolism preventive strategy in COVID-19 patients remains a controversial issue. Therapeutic anticoagulation in the context of severe COVID-19 without a formal indication does not appear to offer a survival advantage and was associated with increased risk of major bleeding episodes. Locally, we adopted an enhanced anticoagulation (enoxaparin twice a day) pathway guided by anti-Xa levels.

**Methods:** This is a retrospective cross-sectional single-center study between March 2020 and March 2021. All patients admitted to the intensive care unit at University Hospital Southampton with diagnosis of COVID-19 confirmed via a reverse-transcriptase-polymerase-chain reaction (RT-PCR) test were included in this study.

**Results:** There were 292 admissions included in the study with a mean age of 60 (± 15). 67.1% received enhanced anticoagulation titrated according to anti-Xa levels. The median day 7 trough and peak anti-Xa levels were 0.33 (IQR 0.18–0.41) and 0.54 (IQR 0.33–0.68) respectively. 62 patients had CTPA for clinical suspicion of pulmonary embolism and 11 were positive. The overall incidence of PE was 3.8%. The distribution of PE was mostly bilateral segmental or unilateral segmental. There were no lobar or main pulmonary artery pulmonary embolism. There were 9 major bleeding episodes in those received enhanced anticoagulation.

**Conclusions:** For critically ill COVID-19 patients, anti-Xa guided enhanced anticoagulation protocol proved to be associated with lower than anticipated incidence of PE with minimal clot burden. Randomised controlled trials are required to explore this concept further.


**Reference**
Roncon L et al. Eur J Intern Med. 2020;82:29–37.


## P286

### Immune suppressant therapy is associated with improved anemia and a decrease in transfusion needs in critically ill COVID patients

#### S Ozkal^1^, N Juffermans^1^, M Koopmans^2^, M Bolscher^1^, R Bosman^1^

##### ^1^OLVG Amsterdam, Intensive Care Unit, Amsterdam, Netherlands, ^2^Zaans Medisch Centrum, Intensive Care Unit, Zaandam, Netherlands

*Critical Care* 2023, **27(S1)**: P286

**Introduction:** Anemia of inflammation is considered to be a main cause of anemia on the ICU. Inflammatory cytokines, most importantly IL-6, play a role in this pathogenesis. Given that both anemia and red blood cell (RBC) transfusions are associated with adverse outcomes, and iron is ineffective, novel treatments of anemia are wanted. The aim of this study is to investigate the effect of immunosuppressive agents on anemia development and RBC transfusions in critically ill COVID patients.

**Methods:** This retrospective cohort study included all ICU patients of two hospitals in the Netherlands between February 2020 and April 2022 with a PCR-positive COVID-19 ARDS. Actively bleeding patients were excluded. Evolving insights in the treatment protocol resulted in three treatment groups: no treatment, steroids or combination of steroids with tocilizumab. Daily lab results and number of RBC transfusion were retrieved and the decline in Hb level between ICU admission day 1 and 7 was calculated. A multiple linear regression analysis was used to compare outcomes.

**Results:** In total, 719 patients were included, of which 168 in the no-treatment group, 337 in the steroid group and 212 in the steroids and tocilizumab group. Hb levels declined in all groups. The median decline in Hb level in the combination group was lowest, with −0.3 mmol/l [−0.9 to 0.2], −0.8 mmol/l [−1.3 to −0.1] in the group receiving steroids in the steroid group and [−1.6 to −0.5] in the no treatment group. The number of RBC transfusions was 1 [1–3] in the group receiving combination therapy, 3[1–6] in the group receiving steroids and 3[2–8] in the group receiving no treatment (*p* < 0.002). In a multivariate analysis, the receipt of combination therapy remained associated with inhibition of decline in Hb as well as with lowering the number of RBC transfusions.

**Conclusions:** Treatment with either steroids or a combination of steroids and tocilizumab was associated with a slower decline in Hb levels during ICU stay and less RBC transfusions when compared to no treatment.

## P287

### Secondary immunodeficiency in septic hematological patients

#### M Barbosa, ME Batista, M Ferraz, S Cardoso, J Casimiro, T Duarte, N Germano

##### Centro Hospitalar Universitário de Lisboa Central, Unidade de Cuidados Intensivos Polivalente- Hospital Curry Cabral, Lisboa, Portugal

*Critical Care* 2023, **27(S1)**: P287

**Introduction:** Secondary immunodeficiency is defined as an impairment of the immune system caused by extrinsic factors, such as acquired infections, drugs and hematological malignancies. Understanding the immunodeficiency in the latter group is crucial to the management of one of the most serious complications: sepsis.

**Methods:** Retrospective observational study of 82 patients with hematologic malignancies who were admitted with sepsis to a polyvalent ICU between January 2016 and November 2022. We recorded baseline characteristics, organ failure scores and outcome. We hypothesized that cytopenia of any lineage would increase the risk for a poorer prognosis and it may help identify those who may benefit the most from early ICU admission.

**Results:** The median age was 63 years in this group. The most common hematologic malignancies were acute myeloid leukemia (47%), non-Hodgkin’s lymphoma (19%) and multiple myeloma (17%). Median SOFA score at admission was 8 (IQR 7–11), APACHE II was 17 (IQR 12.23) and SAPS II was 61 (IQR 49–77) with an estimated mortality of 30% and 66%, respectively. Median ICU stay was 8 days and time till hospital discharge was 7 days. At admission 21.6% had anemia. Leucopenia was found in 56.8%, neutropenia in 58.8%, and lymphopenia and monocytopenia was found in 81.1% and 86.5%, respectively. Median ICU and in-hospital mortality was 67.6% and 81.1%, respectively. Univariate analysis revealed that anemia (*p* = 0.027), neutropenia (*p* = 0.005), thrombocytopenia (*p* = 0.036) were associated with > 2 organ dysfunction and ICU mortality in patients with hematologic malignancies and sepsis.

**Conclusions:** There is a myriad of causes of cytopenia in this population. Each lineage disruption may augment the sepsis severity, from decreased functional capacity to direct immunosuppression. The presence of specific cytopenias should make us consider a more aggressive management to infections in hematological patients.

## P288

### A retrospective review of the critical care management of patients receiving CAR T therapy at Manchester Royal Infirmary (MRI)

#### JA Mooney, E Tholouli, A Wilson, M Nirmalan

##### Manchester Royal Infirmary, Manchester, UK

*Critical Care* 2023, **27(S1)**: P288

**Introduction:** Chimeric antigen receptor (CAR) T-cell therapy is a successful treatment for some haematological malignancies which have proved refractory to multiple lines of treatment. Cytokine release syndrome and neurotoxicity are recognised complications of CAR T therapy which may require critical care support. As indications for CAR T-cell therapy grow, this may create a resource pressure for critical care.

**Methods:** We performed a service evaluation of all CAR T admissions to the intensive care unit (ICU) at Manchester Royal Infirmary (MRI) from June 2019 to May 2022. The objectives were to describe the resource requirements and outcomes of these patients admitted to ICU and compare them with those who did not require ICU care.

**Results:** 69 patients were treated at MRI over 35 months with 23 patients (33.3%) requiring critical care admission. Of these 23 patients, 3 (13%) required renal replacement therapy, 2 (9%) required non-invasive ventilation, 2 (9%) required intubation and 6(26%) required vasopressor support. The median length of stay on ICU for patients was 4 days. 60% of ICU patients survived 6 months, versus 74% for non-ICU patients.

**Conclusions:** Our findings highlight that rates of critical care admission following CAR T treatment have reduced at our centre as the maturity of our programme has grown. In addition, we have observed that most patients admitted to ICU did not require level 2 or level 3 care. This has implications for critical care capacity planning in new CAR T centres. It also highlights that there is a need for better stratification of patients into those who truly need an ICU bed for level 2 or level 3 care and those who may be better served by enhanced monitoring in a ward-based environment.

## P289

### Hematologic patients in ICU—where do we stand?

#### ME Batista, M Barbosa, M Ferraz, J Casimiro, S Cardoso, T Duarte, N Germano

##### Centro Hospitalar Universitário de Lisboa Central, Unidade Cuidados Intensivos Polivalente—Hospital Curry Cabral, Lisboa, Portugal

*Critical Care* 2023, **27(S1)**: P289

**Introduction:** Hematologic patients have a poor prognosis in intensive care, leading to strict admission policy. Mortality rates as high as 77% raises the question: where can we improve outcomes? This study searched the peri-admission period to understand this group’s specificity profile.

**Methods:** Retrospective cohort with 117 patients with hematologic malignancies who were admitted to a non-hematologic ICU between January 2016 and November 2022. Patients baseline characteristics, organ failure scores and outcome were analyzed to find predictors of clinical deterioration.

**Results:** A total of 117 patients were included, median age was 58.17 years. The most common hematologic malignancies were acute myeloid leukemia (46.1%), non-Hodgkin's lymphoma (22.2%) and multiple myeloma (11.1%). Primary reasons for ICU admission were respiratory failure (45.3%), septic shock (40.2%), acute kidney injury (3.4%). Median ICU stay was 10 days and time till hospital discharge was 8 days. Median ICU and in-hospital mortality was 61.2% and 71.4%, respectively. Mortality was higher in patients with acute myeloid leukemia and septic shock. At admission, the median SOFA score was 8 (IQR 5–10). Median APACHE II was 16 (IQR 12–22), and median SAPS II was 57 (IQR 48–70), with significant difference between survivors and non-survival groups (*p* < 0.005). SOFA 48 h and 24 h before admission was significantly higher in the non-survival group (5 vs 4; 6 vs 5). Multivariate analysis revealed that AKI ≥ 2 and neutropenia were independent predictors of ICU mortality.

**Conclusions:** This 7 year cohort is in line with published literature regarding ICU and in-hospital mortality. This study provides some insight into the time window for early admission, as patients with worse outcomes have severity signs up to 48 h prior to ICU admission. AKI is associated with lower complete remission rate for newly diagnosed hematological malignancies; in our study, was also an independent predictor of mortality.

## P290

### Plasma expansion and renal perfusion in critical COVID-19 with AKI

#### T Luther^1^, P Eckerbom^2^, E Cox^3^, M Lipcsey^4^, M Hultström^5^, J Weis^6^, F Palm^7^, S Francis^3^, P Liss^2^, R Frithiof^1^

##### ^1^Uppsala University, Dept of Surgical Sciences, Anaesthesia and Intensive Care, Uppsala, Sweden, ^2^Uppsala University, Dept of Surgical Sciences, Section of Radiology, Uppsala, Sweden, ^3^University of Nottingham and Nottingham University Hospitals NHS, Sir Peter Mansfield Imaging Centre, School of Physics & Astronomy and NIHR Nottingham Biomedical Research Centre, Nottingham, UK, ^4^Uppsala University, Dept of Surgical Sciences, Anaesthesia and Intensive Care and Hedenstierna Laboratory, Uppsala, Sweden, ^5^Uppsala University, Dept of Surgical Sciences, Anaesthesia and Intensive Care and Integrative Physiology, Department Medical Cell Biology, Uppsala, Sweden, ^6^Uppsala University Hospital, Department of Medical Physics, Uppsala, Sweden, ^7^Uppsala University, Integrative Physiology, Department of Medical Cell Biology, Uppsala, Sweden

*Critical Care* 2023, **27(S1)**: P290

**Introduction:** We have previously described decreased renal perfusion in acute kidney injury (AKI) due to critical COVID-19 [1]. The objective of this study was to compare the effects of plasma expansion with a standardized fluid bolus on renal perfusion in patients with AKI compared to similar patients without AKI.

**Methods:** A case control study design was used to investigate group differences before and after a standardized intervention. ICU-treated COVID-19 patients without underlying kidney disease were assigned to two groups based on KDIGO Creatinine criteria for AKI. Renal perfusion was assessed by magnetic resonance imaging using phase contrast and arterial spin labeling before and directly after plasma expansion with 7.5 ml/kg Ringer’s Acetate (Baxter). Mean arterial pressure (MAP) was recorded before plasma infusion and compared with maximum value after. Data was analyzed with a mixed model repeated measures ANOVA for all kidneys using a random effect to account for research subjects.

**Results:** Nine patients with AKI and eight without were included in the study. Patients in both groups were of similar mean age and weight, 66 (SD 8) years and 94 (SD 22) kg in AKI group and 64 (SD 15) years and 93 (SD 20) kg in patients without AKI. The response to plasma expansion was similar with increased MAP by 18 (CI 8–28) mmHg and 20 (CI 10–31) mmHg respectively (Table 1). Total renal perfusion and cortical perfusion was not significantly changed by plasma expansion, however there was a reduction of medullary perfusion in patients without AKI (Table 1).

**Conclusions:** Plasma expansion with a standardized fluid bolus did not increase renal perfusion in critically ill patients with ARDS due to COVID-19.


**Reference**
Luther T et al. Crit Care 2022;26:262.

**Table Tabdc:** **Table 1 (abstract P290)**. Hemodynamic and renal perfusion data of 17 patients with ARDS due to COVID-19 of which 9 had AKI and 8 did not

Group and time related to fluid bolus	AKI Group before fluid	AKI Group after fluid	No AKI Group before fluid	No AKI Group after fluid
MAP (mmHg)‡‡‡	80 (72–89)	98 (90–107)**	90(81–99)	110(101–119)**
Renal blood flow (ml/min/kidney)	321 (224–460)	331 (231–475)	395 (274–568)	389 (270–560)
Perfusion renal cortex (ml/min/100 g)	81 (58–113)	87 (62–121)	139 (98–197)	123 (87–174)
Perfusion renal medulla (ml/min/100 g) †††	32 (23–45)	33 (24–47)	55 (39–79)	34 (24–48)**

Data are presented groupwise at times related to fluid infusion as mean (95% confidence interval). Mean arterial pressure (MAP) refers to MAP before plasma expansion and maximal MAP after. ‡‡‡ denotes as *p* < 0.001 for effect of plasma expansion. †††denotes as interaction between plasma expansion and AKI status in ANOVA. ** denotes as *p* < 0.01 for pairwise comparison before-after within group

## P291

### Clinical and therapeutic characterization of severe rhabdomyolysis in northeastern Mexico

#### C Zarazua-Sosa, JA Garza-Carrion, A Zarate-Gracia, JA Villalobos-Silva

##### General Hospital of Victoria City “Dr. Norberto Treviño Zapata”, Intensive Care Unit, Victoria, Tamaulipas, Mexico

*Critical Care* 2023, **27(S1)**: P291

**Introduction:** Rhabdomyolysis is an underdiagnosed acquired clinical syndrome [1]. We review the evolution, early treatment and complications of severe rhabdomyolysis in the ICU of an Hospital in Northeast Mexico from January 1 to October 30, 2022.

**Methods:** An observational, descriptive, retrospective cohort study was conducted. We reviewed 72 clinical records, the data obtained was transferred to a database and analyzed in the statistical program SPSS 23. A descriptive analysis was performed for each variable, in which were calculated measures of central tendency and dispersion, as mean and standard deviation. Some variables were included in t student for related samples, bivariate correlation with odds ratio (OR) for CPK > 5000 and Pearson’s coefficient for lactate.

**Results:** We reviewed 72 cases. Age 38 ± 13 (16–71); 83% male; obesity 30% (BMI > 30); comorbidities (AH + DM) in 18%; etiologies: 90% traumatic, 6% exercise, 4% other; APACHE II 19.3 ± 09.1 (6–29), SOFA 6 ± 3 (4–14), CPK 14058.8 ± 4681.9 IU/l (2160–156,128); DHL 1236.8 ± 458.2 (366–8657); lactate 4.7 ± 4.4 mmol/l (1–18.8); myoglobinuria in 15%. Serum HCO3 level 17.5 ± 4.3 (3–25); BE − 7.8 ± 5.3 (− 23 = 0.3). Fluid optimization with balanced solutions 57%, isotonic saline solution 43%, norepinephrine infusion 25%; loop diuretic 21%, osmotic diuretic 26%. Complications 43% (AKI 30%, infections 18%; liver injury 12%, mechanical ventilation (MV) 30%. Fluid balance 24 h 824.3 ± 178 ml vs 48 h 945.9 ± 370 ml (*p* = 0.669); OR for CPK > 5000 IU/l associated with MV, (1.37, CI 0.47–3.7 *p* = 0.38), OR for CPK associated with vasopressors (1.12 CI 0.44–3.12 *p* = 0.66), OR CPK > 5000 IU/l associated complications (2.64 CI 1.0–7.17 *p* = 0.04), OR CPK > 5000 associated with AKI (3.32 CI 1.3–8.4 *p* = 0.001), r = 0.88 for CPK-lactate (*p* = 0.004).

**Conclusions:** We conclude that CPK level > 5000 IU/l is associated with more clinical complications, mainly AKI, hyperlactatemia, use of vasopressors and MV. Within our limitations we consider the size of the sample.


**Reference**
Kodadek L et al. Trauma Surg Acute Care Open 2022;7:e000836.


## P292

### Estimated glomerular filtration rate among intensive care unit survivors: from the removal of race coefficient to the use of cystatin C-based equations

#### A Cela, A Pinsino, J Wu, A Mohamed, S Rednor, M Gong, A Moskowitz

##### Montefiore Medical Center, Critical Care Medicine, Bronx, USA

*Critical Care* 2023, **27(S1)**: P292

**Introduction:** Serum creatinine (sCr)-based estimated glomerular filtration rate (eGFR) equations have historically included a coefficient for Black race. Due to sarcopenia and malnutrition, sCr overestimates GFR among intensive care unit (ICU) survivors. The use of cystatin C (cysC), an eGFR marker which is independent from race and muscle mass, improves accuracy. Among ICU survivors, we investigated the implications of the removal of race coefficient from eGFR calculation and the discrepancy between sCr- and cysC-based eGFR.

**Methods:** Among 30,919 ICU survivors admitted at 4 hospitals from 2 US institutions (Montefiore, Bronx; and Beth Israel Deaconess, Boston), eGFR was calculated at discharge using sCr-based equations with and without race coefficient (eGFRsCr_+RC_ and eGFRsCr_-RC_). In a subset of patients with available cysC between ICU admission and 1-year follow-up, eGFRsCr_+RC_ and eGFRsCr_-RC_ were compared with cysC-based eGFR (eGFRcysC).

**Results:** eGFRsCr_-RC_ was higher than eGFRsCr_+RC_ by a median of 4 ml/min/1.73 m^2^ among Non-Black patients and lower by a median of 8 ml/min/1.73 m^2^ among Black patients, resulting in a variation in eGFR stage in 13.6% of the subjects (Fig. 1). Removal of race coefficient differentially impacted the 2 institutions based on the degree of diversity in the racial composition (Fig. 1). CysC was available in 51 patients with a total of 108 measurements. eGFRcysC was lower than eGFRsCr_-RC_ and eGFRsCr_+RC_ by a median of 16 and 21 ml/min/1.73 m^2^, respectively, resulting in a reclassification towards worse eGFR stage in more than 50% of the total measurements.

**Conclusions:** In a cohort of ICU survivors, the removal of race coefficient results in lower eGFR among Black patients, but appears to contribute to the overestimation of GFR among Non-Black patients. CysC may improve accuracy and reduce racial disparities in the identification of kidney dysfunction, but remains underutilized during ICU admission and the subsequent follow-up.

**Figure Figdo:**
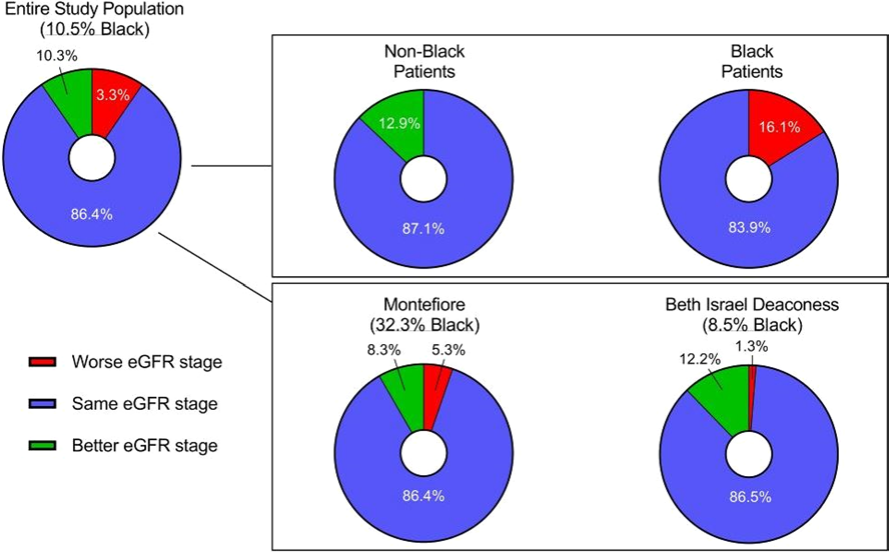
**Fig. 1 (abstract P292)**. Reclassification after removal of race coefficient: eGFRsCr_-RC_ versus eGFRsCr_+RC_. Kidney disease stage as defined by eGFR: 1 (≥ 90), 2 (60–89), 3a (45–59), 3b (30–44), 4 (15–29), 5 (< 15)

## P293

### Adjusted Cockcroft-Gault formula by lean body weight is a better approach to measured creatinine clearance in critically ill patients

#### P Vargas^1^, N Dreyse^2^, R López^3^, M Espinoza^3^, J Graf^3^, J Guerrero^3^, S Aracena^4^, JM Montes^3^

##### ^1^Clínica Alemana de Santiago, Unidad de Paciente Critico, Santiago, Chile, ^2^Clínica Alemana de Santiago, Departamento de Farmacia, Santiago, Chile, ^3^Clínica Alemana de Santiago, Departamento Paciente Crítico, Santiago, Chile, ^4^Clínica Alemana de Santiago, Servicios de Urgencia, Santiago, Chile

*Critical Care* 2023, **27(S1)**: P293

**Introduction:** The aim of this study was to assess the ability of adjusted Cockcroft-Gault formula by lean body weight as an approach to 24 h urine creatinine clearance in critically ill patients.

**Methods:** In a prospective cohort of critically ill patients admitted to our ICU, we obtained an Adjusted Cockcroft-Gault formula by lean body weight in agreement with Janmahasatian et al. [1] and compared this with creatinine clearance measured by 24 h urine collection. The agreement limits were explored by Bland–Altmann chart. This protocol was approved by local IRB and informed consent was obtained.

**Results:** One hundred patients with 110 24 h creatinine clearance measurements were analyzed with mean age of 59.8 (16.5) years, mean serum creatinine 0.93 (0.51) mg/dl, urine creatinine 817 (361) mg/24 h, 24 h creatinine clearance 67.2 (34.0) ml/min/1.73 m^2^. Accuracy and bias comparison between adjusted and unadjusted Cockcroft-Gault formula are shown in Table 1.

**Conclusions:** Adjusted Cockcroft-Gault formula by lean body weight seems a better approach to 24 h creatinine clearance than Cockcroft-Gault formula. This could decrease overestimation of kidney function allowing a better drugs prescription in critically ill patients.


**Reference**
Janmahasatian S et al. Clin Pharmacokinet 2005;44:1051–1065.

**Table Tabdd:** **Table 1 (abstract P293)**. Absolute percentage error, bias, accuracy under of 50 ml/min/1.73 m^2^ and overestimation of category over 50 and 70 ml/min/1.73 m^2^ between CGTBW and CGLBW in comparison to a 24 h urine creatinine clearance

n = 110	Absolute percentage error (IC 95%)	Bias(LA)	Accuracy Cr cl < 50 ml/min/1.73 m^2^ (%)	Overestimation(< 50 and < 70 ml/min/1.73 m^2^)
CGTBW/CrCl 24 h	58.3 (39–77)	−25.7 (−91 to 39)	37.7	43/46 (93.5%)
CGLBW/CrCl 24 h	10.7(− 2/23)	9.6 (−65 to 84)	66.7	14/30 (46.7)

## P294

### Biomarkers of renal injury as early predictors of COVID-19 associated AKI: a prospective observational trial (BRICOAKI-trial)

#### N Kumar, A Kumar, A Kumar, K Singh

##### All India Institute of Medical Sciences Patna, Anaesthesiology, Patna, India

*Critical Care* 2023, **27(S1)**: P294

**Introduction:** Early prediction by the use of serum and urinary biomarkers for the detection of acute kidney injury (AKI) may be very valuable to optimize the management and helps in improving the outcomes. This study aims to investigate whether daily measurement of urinary and plasma renal biomarkers have a role in earlier predicting COVID-19 associated AKI.

**Methods:** The study was conducted as a single-center, prospective, observational cohort study between August 2020 and December 2020 in hospitalized COVID-19 patients. A total of 65 moderate and severe COVID-19 positive adult (≥ 18 years) patients were enrolled for this study. We measured serum creatinine, cystatin C, NGAL, KIM-1, Urine-Klotho, TIMP-2, IL-6 level, and urinary microalbumin/urinary creatinine on various days. The receiver operating characteristic curve (ROC) analysis was used to find the sensitivity and specificity of various markers to predict the incidence of AKI.

**Results:** A total of 24 moderate and 41 severe COVID-19 patients were included. Out of which 47 patients developed (72.3%) acute kidney injury (AKI) over the course of COVID-19. Among these subjects, 18/47 (38.2%) developed severe AKI (KDIGO 2 + 3), and 5/47 (10.6%) required RRT. NGAL was found to be the best marker to predict the probability of AKI (Area under curve AUC of 0.713–0.786) with a sensitivity of 76–90% and specificity of 56–79% on different days of assessment from Day 1 to Day 7. IL-6 had moderate accuracy of prediction and cystatin C, KIM-1, Urine-Klotho, TIMP-2, IL-6 had poor accuracy for predicting the incidence of AKI.

**Conclusions:** Urinary biomarkers like NGAL have good predictability for AKI.

## P295

### Systematic review of risk factors and outcomes associated with the development of persistent acute kidney injury in non-renal solid organ transplant recipients

#### IE Saraiva^1^, N Hamahata^1^, A Sakhuja^2^, X Chen^3^, JS Minturn^3^, PG Sanchez^4^, E Chan^4^, DJ Kaczorowski^4^, A Al-Khafaji^3^, H Gomez^3^

##### ^1^University of Pittsburgh Medical Center, Department of Critical Care Medicine, Pittsburgh, USA, ^2^West Virginia University, Division of Critical Care, Morgantown, USA, ^3^University of Pittsburgh, Department of Critical Care Medicine, Pittsburgh, USA, ^4^University of Pittsburgh, Department of Cardiothoracic Surgery, Pittsburgh, USA

*Critical Care* 2023, **27(S1)**: P295

**Introduction:** Persistent acute kidney injury (pAKI), defined as AKI that persists beyond 72 h of the initial insult, carries worse prognosis than AKI that resolves within this timeframe in critically ill patients. However, the association of persistent AKI and outcome is not well characterized in solid organ transplant patients. The aims of this study are (1) to determine the occurrence of pAKI and the association between pAKI and clinical outcomes and (2) to identify the risk factors for developing persistent AKI among heart, lung or liver transplant recipients.

**Methods:** We performed a systematic review of the literature including PubMed, EMBASE, Web of Science, and Cochrane Library. We included human prospective and retrospective cohort and randomized clinical studies that involved recipients of heart, lung, and/or liver transplant during the index hospitalization, and reported on the rates of occurrence of pAKI, and outcomes including graft failure and mortality.

**Results:** We identified 8789 records, of which 3228 were selected for abstract review. Ten studies were selected for full text review and included in final analysis. One study reported 152 episodes of pAKI (54.5%) in 279 patients receiving 301 liver grafts. pAKI was associated with graft loss in 25% compared to 8.7% in patients without pAKI. Three studies reported occurrence of pAKI of 32.4–49.2% in 821 lung transplant recipients. Risk factors reported were body mass index, nephrotoxic agents, and hypotension. pAKI was associated with all-cause mortality with a HR varying substantially between 1.77 and 14.69. No studies investigating pAKI in heart transplant patients were found.

**Conclusions:** Persistent AKI is a common complication in non-renal solid organ transplant recipients and is associated with worse clinical outcomes. Utilization of standard nomenclature and attention to timing of renal dysfunction are essential aspects to better understand the problem and development of mitigation strategies.

## P296

### Expression of specific micro RNAs in septic patients treated with nephrotoxic antibiotic agents

#### N Petejova^1^, A Martinek^2^, J Zadrazil^3^, V Klementa^4^, M Kanova^2^, R Sigutova^5^, L Pribylova^6^, I Kacirova^2^, E Bace^7^, D Stejskal^5^

##### ^1^University Hospital, Department of Internal Medicine and Cardiology, Ostrava, Czech Republic, ^2^University Hospital, Ostrava, Czech Republic, ^3^University Hospital Olomouc, Olomouc, Czech Republic, ^4^University Hospital Olomouc, Olomouc, Czech Republic, ^5^Faculty of Medicine University of Ostrava, Ostrava, Czech Republic, ^6^Faculty of Electrical Engineering and Computer Science, VSB Technical University of Ostrava, Ostrava, Czech Republic, ^7^Department of immunodiagnostics, eBioVendor R&D, Laboratory Medicine Corp., Brno, Czech Republic

*Critical Care* 2023, **27(S1)**: P296

**Introduction:** Sepsis is a common life-threatening condition with detrimental effects on many organs and systems. Through regulation of signaling inflammatory pathways, small RNA nucleotides—microRNAs (miRNAs) can be involved in sepsis and associated organ dysfunction. The aims of this study were to track the 7-days time course of serum miRNAs in patients with sepsis treated with vancomycin, gentamicin, or a non-nephrotoxic antibiotic and miRNA associations with neutrophil gelatinase-associated lipokalin (NGAL), creatinine, procalcitonin, interleukin-6, and acute kidney injury (AKI) stage [1].

**Methods:** Prospective open clinical study included 46 adult septic patients, 7 on vancomycin, 20 on gentamicin, and 19 on another antibiotic treatment. Blood samples were collected on days 1, 4, and 7 of treatment, and circulating miRNAs were identified using quantitative reverse transcription PCR during the study.

**Results:** Four circulating miRNAs have been identified. We found no relationship between miRNA and biochemical variables on day 1. By day 7 of gentamicin treatment, *miR-15a-5p* provided good discrimination between AKI and non-AKI (area under curve, 0.828). In vancomycin group *miR-155-5p* and *miR-192-5p* positively correlated with creatinine and NGAL, and *miR-192-5p* and *miR-423-5p* positively correlated with procalcitonin and interleukin-6 in patients treated with a non- nephrotoxic antibiotic agents. In all patients together we found positive correlation between *miR-155-5p* and *miR-423-5p* and all biochemical markers during the study.

**Conclusions:** Results showed that these four miRNAs may be helpful as diagnostic or therapeutic tool in septic patients with AKI or on nephrotoxic therapy.


**Reference**
Petejova N et al. BMC Nephrol 2022;23:111.


## P297

### Identification of an optimal threshold to define oliguria in critically-ill patients: an observational study

#### N Bianchi^1^, M Altarelli^1^, C Monard^1^, T Kelevina^1^, A Chaouch^2^, A Schneider^1^

##### ^1^Centre Hospitalier Universitaire Vaudois, Service de Médecine Intensive Adulte, Lausanne, Switzerland, ^2^Centre Hospitalier Universitaire Vaudois, Faculty of Biology and Medicine (FBM), Lausanne, Switzerland

*Critical Care* 2023, **27(S1)**: P297

**Introduction:** Current threshold to define oliguria (UO < 0.5 ml/kg/h for 6 h) has been challenged by observational studies. We aim to determine the optimal threshold to define oliguria in critically-ill patients.

**Methods:** We conducted a cohort study including all patients admitted in a multi-disciplinary ICU between January 1st 2010 and June 15th 2020, except those on chronic dialysis and those who declined consent. Hourly urinary output (UO) measurements along with patient’s characteristics and severity scores were extracted from electronic medical records and 90 day mortality from the Swiss national death registry. We randomly split our data into a training (80%) and a validation (20%) set. We developed models to assess the relationship between 90-day mortality and minimum average UO calculated over time windows of 3, 6, 12 and 24 h. Models’ discrimination and calibration were tested on validation set. Optimal thresholds were determined for the minimum average UO below which predicted mortality increased substantially.

**Results:** Among the 15,500 patients included (training set: 12,440, validation set: 3110), 73.0% (95% CI [72.3–73.8]) had an episode of oliguria as defined by consensus criteria. The relationship between minimum average UO and predicted 90-day mortality was non-linear, with inflexion points at 0.2 ml/kg/h for 3 and 6 h windows and 0.3 ml/kg/h for 12 and 24 h (Fig. 1). Our models had excellent discrimination and calibration performances (AUC > 85%). With a threshold of < 0.2 ml/kg/h for 6 h, the proportion of patients with an episode of oliguria dropped to 24.7% (95% CI [24.0–25.4]). Contrary to consensus definition, this threshold identified a higher adjusted 90-day mortality risk (OR 1.98 [95% CI 1.57–2.49] vs 1.27 [95% CI 0.95–1.70]).

**Conclusions:** Current consensus cut-off for oliguria may be too conservative. A value of 0.2 ml/kg/h over 3 or 6 h is supported by the data and should be considered in further definitions of oliguria.

**Figure Figdp:**
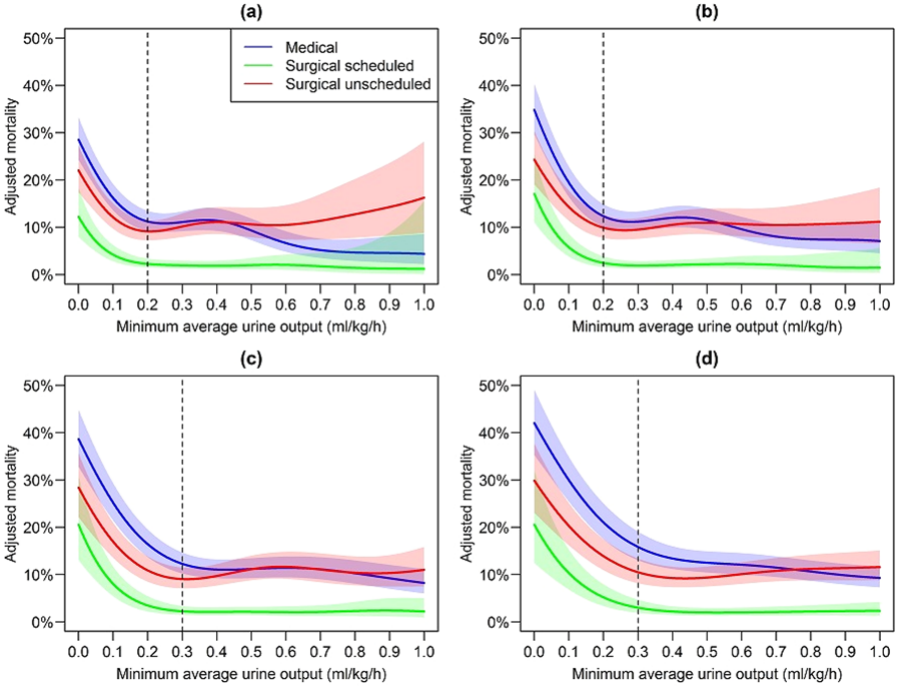
**Fig. 1 (abstract P297)**. Predicted 90-day mortality as a function of the minimum average urine output over 3 h (**a**), 6 h (**b**), 12 h (**c**) and 24 h (**d**), stratified by type of admission. Colored areas = 95% confidence intervals around regression lines. Vertical dashed lines = thresholds below which predicted mortality increases rapidly. Predictions are carried out for a fictive patient with continuous predictors fixed at their median value (65 years old at ICU admission, corrected SAPS II score of 37, Charlson index of 4)

## P298

### Early versus late acute kidney injury in critically ill patients admitted in intensive care units for sepsis

#### C Monard, N Bianchi, T Kelevina, M Altarelli, A Schneider

##### Centre Hospitalier Universitaire Vaudois, Service de Médecine Intensive Adulte, Lausanne, Switzerland

*Critical Care* 2023, **27(S1)**: P298

**Introduction:** The Acute Disease Quality Initiative (ADQI) workgroup has recently proposed to distinguish early (occurring within 2 days) from late (occurring between 2 and 7 days) sepsis associated acute kidney injury (SA-AKI). We aimed to determine the relative frequency of these entities and to report on patients’ characteristics and outcomes.

**Methods:** We analyzed all adult patients admitted to our ICU with sepsis as a main diagnosis between January 1st 2010 and June 15th 2020 except for those on chronic dialysis or who declined consent. Daily serum creatinine (sCr) and hourly urinary output (UO) measurements, along with clinical and socio-demographic data, were extracted from electronic medical records until day-7 or ICU discharge, whichever occurred first. We applied KDIGO criteria to assess, on a daily basis, the presence and severity of AKI. We compared patients who fulfilled criteria for early (present at admission or onset within 2 days of admission) or late (onset > 2 days of admission) SA-AKI.

**Results:** Among the 1989 patients included in our analyses (1314 (66%) males, median (IQR) age 67 (56–76) years and SAPS II score 50 (40–62)), 1779 (89%) fulfilled AKI criteria. Of those, 1743 (98.0%) had early AKI and 36 (2.0%) had late AKI (Fig. 1). Similar findings were observed when only considering AKI based on elevated serum creatinine and/or severe (stage 2–3) AKI. Compared to patients with early SA-AKI, those with late SA-AKI were younger [median (IQR) age: 56 (48–71) vs 68 (58–76) years old], had less comorbidities [median (IQR) Charlson comorbidities index: 2(1–6) vs 5(3–7)] and lower SAPS II scores [median (IQR) 37 (33–46) vs 52 (42–64)]. They had lower (17 vs 25%) in-hospital mortality rate.

**Conclusions:** AKI is very common in critically ill patients with sepsis and almost always occurs within 48 h of admission. The timing from ICU admission might not be a relevant criteria to distinguish sepsis-associated from sepsis-induced AKI.

**Figure Figdq:**
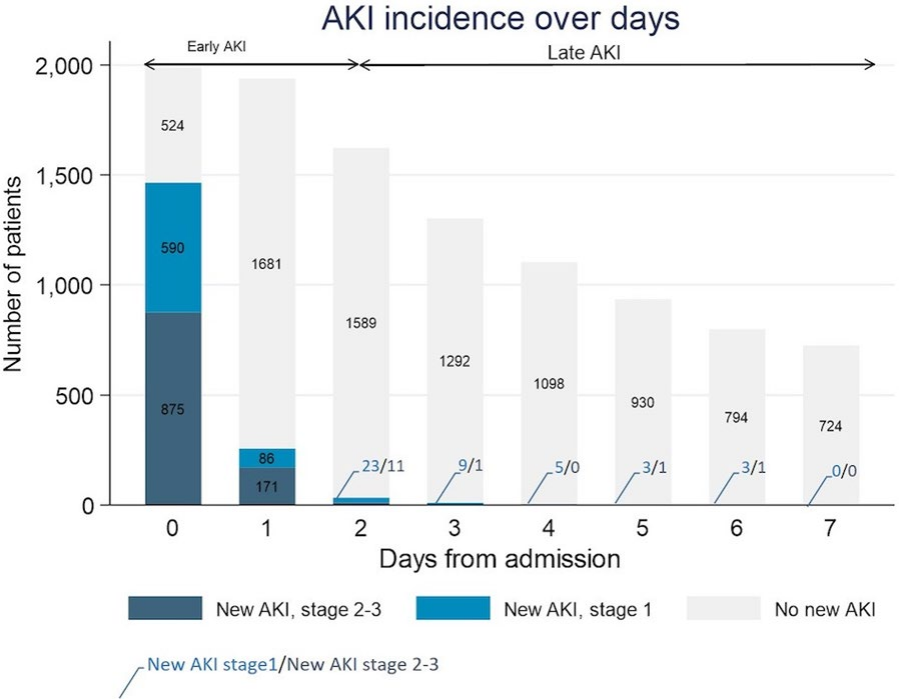
**Fig. 1 (abstract P298)**. Diagnosis of AKI from admission to day-7 among patients admitted in ICU with sepsis

## P299

### The effect of glutamine on kidney damage in cardiac surgery patients at high risk for AKI: a double-blind randomized controlled trial

#### R Weiss^1^, T Von Groote^2^, M Meersch^2^, M Schäfers^3^, JA Kellum^4^, A Zarbock^2^

##### ^1^University Hospital Münster, Department for Anesthesiology, Intensive Care and Pain Medicine, Münster, Germany, ^2^University Hospital Münster, Münster, Germany, ^3^University Hospital Münster, Department of Nuclear Medicine, Münster, Germany, ^4^University of Pittsburgh, Center for Critical Care Nephrology, Department of Critical Care Medicine, Pittsburgh, USA

*Critical Care* 2023, **27(S1)**: P299

**Introduction:** Acute kidney injury (AKI) after cardiac surgery is associated with increased morbidity and mortality. However, no specific treatment options are available. As an amino acid, glutamine serves multiple purposes in the human body, including reactive oxygen species scavenging and elimination. This trial assesses the effect of glutamine on renal damage biomarkers in cardiac surgery patients at risk for AKI.

**Methods:** 64 high risk patients were identified by high postoperative urinary [TIMP2]*[IGFBP7] levels and received either body weight adapted glutamine or saline-control for 12 h intravenously. The primary outcome was the change in urinary [TIMP2]*[IGFBP7] levels after 12 h of administration. Secondary outcomes included the change in KIM-1 and NGAL, overall AKI rates, free days of mechanical ventilation and vasoactive medication, renal replacement therapy, mortality, length of ICU/hospital stay, and major adverse kidney events at day 90.

**Results:** Patients received coronary artery bypass graft surgery (32/64), valve surgery (18/64), a combination of both (6/64), or other procedures (8/64). Mean age was 68 [SD ± 10] years, 10 of 64 were women. Mean on-pump time was 68.4 [SD ± 10.5] minutes. After glutamine administration, urinary [TIMP-2]*[IGFBP7] were significantly lower in the glutamine compared to the control group (primary endpoint, intervention: median 0.18 (Q1, Q3; 0.09, 0.29), controls: median 0.44 (Q1, Q3; 0.14, 0.79), *p* = 0.01) (Table 1). KIM-1 and NGAL were also significantly lower in the glutamine group. The overall AKI rate within 72 h was not different among groups: intervention 11/31 (35.5%) vs. control 8/32 (25.0%); *p* = 0.419; RR, 0.86% (95% CI 0.62%, 1.20%). There were no significant differences regarding further secondary endpoints.

**Conclusions:** Glutamine significantly decreased markers of kidney damage in cardiac surgery patients at high risk for AKI. Future trials need to investigate whether the administration of glutamine might be able to reduce the occurrence of AKI after cardiac surgery.

**Table Tabdf:** **Table 1 (abstract P299)**. Results

		Glutamine (n = 31)	Control (n = 32)	*p* value
[TIMP2]*[IGFBP7]	Pre-treatment; Post-treatment	0.62 (0.37, 1.45); 0.18 (0.09, 0.29)	0.58 (0.38, 0.77); 0.44 (0.14, 0.79)	0.01
	Δ [TIMP2]*[IGFBP7]	−0.42 (−1.37, − 0.21)	−0.12 (−0.40, 0.18)	0.005
KIM-1	Pre-treatment; Post-treatment	1.26 (0.65, 2.79); 3.18 (1.38, 6.10)	1.39 (0.82, 2.38); 5.20 (3.12, 8.23)	0.021
	Δ KIM-1	1.38 (−0.25, 3.64)	3.32 (2.43, 6.00)	0.011
NGAL	Pre-treatment; Post-treatment	221.69 (36.38, 2211.54); 61.32 (34.14, 87.02)	250.44 (69.48, 756.80); 104.19 (40.22, 284.14)	0.028
	Δ NGAL	−139.85 (−2154.16, −5.70)	−59.69 (−302.82, 0.88)	0.079

Presented as median (quartile 1, quartile 3). Abbreviations: Δ, difference; *CPB* cardiopulmonary bypass, *KIM-1* kidney injury molecule-1, *NGAL* neutrophil gelatinase-associated lipocalin, *TIMP-2* tissue inhibitor of metalloproteinases 2, *IGFBP7* insulin like growth factor binding protein 7, *Q* quartile. Data were tested using Wilcoxon rank-sum tests. All confidence intervals are standard 95%

## P300

### Major determinants of immediate renal graft function from kidney donation after Maastricht II circulatory death: a single center experience

#### AC Gaspar1, M Gama^2^, G Nobre de Jesus^1^, J Oliveira^3^, M Neves^3^, A Santana^3^, JM Ribeiro^1^

##### ^1^Hospital de Santa Maria, Centro Hospitalar Lisboa Norte, EPE, Intensive Care Department, ECMO Referral Center, Lisbon, Portugal, ^2^Hospital de Santa Maria, Centro Hospitalar Lisboa Norte, EPE, Transplant Coordination Department, Lisbon, Portugal, ^3^Hospital de Santa Maria, Centro Hospitalar Lisboa Norte, EPE, Nephrology and Transplantation Department, Lisbon, Portugal

*Critical Care* 2023, **27(S1)**: P300

**Introduction:** Organ donor shortage limits the effectiveness of kidney transplantation programs. Uncontrolled donation after refractory cardiocirculatory death (uDCCD) is associated with prolonged warm ischemia time and perfusion by abdominal normothermic oxygenated recirculation (ANOR), both contributing to the risk of primary non-function or delayed graft function. Our primary goal was to evaluate uDCCD process and its impact on immediate graft function.

**Methods:** A retrospective, non-interventional, ethical committee approved study was designed in a university tertiary hospital ECMO referral Centre. All kidney grafts harvested after cardiac death were considered for this study, with a minimum 6-month follow-up. Data was collected from electronic files. Immediate graft function was characterized in transplanted patients. For statistical analysis, SPSS® software was used.

**Results:** From 2017 until May 2022, 31 renal grafts collected from 21 donors, were transplanted in our center. Mean age of donors was 46.7 years (16 male). Asystole was present in 18 donors (58.1%), ventricular fibrillation in 7 (22.6%) and pulseless electrical activity in 6 (19.6%). Mean warm ischemia time was 107 ± 23 min, mean time from no-touch period to ANOR, including time to complete cannulation procedure, was 17 ± 15 min and mean duration of ANOR was 165 ± 59 min. Mean age of receptors was 43.2 years (16 women). Nine recipients (29%) had immediate graft function, 19 (61.3%) had delayed graft function and 3 (9.7%) had primary non function. There was an association between both time from no-touch period to ANOR (*p* = 0.01) and duration of ANOR with immediate graft function (*p* = 0.021). Time from no-touch period to ANOR correlated with primary graft non-function (*p* = 0.0005) and ANOR duration correlated with delayed graft function (*p* = 0.015).

**Conclusions:** Our study suggests that time from no-touch period to ANOR and duration of ANOR, were the most impacting factors for immediate graft function.

## P301

### A retrospective comparative study of acute kidney injury among COVID-19 and non-COVID-19 ICU patients

#### T Maniatopoulou, M Ksenikakis, M Pashali, N Stefanatou, P Antoniou, A Papathanasiou, N Kazakos, V Koulouras, I Andrianopoulos

##### University Hospital of Ioannina, Intensive Care Unit, Ioannina, Greece

*Critical Care* 2023, **27(S1)**: P301

**Introduction:** Acute kidney injury (AKI) appears to be prevalent in ICU COVID-19 patients. Nevertheless, there are few data in comparison with non-COVID-19 patients. The aim of our study was to compare the prevalence of AKI in COVID-19 and non-COVID-19 critically ill patients.

**Methods:** We performed a retrospective single-center study including all consecutively COVID-19 critically ill mechanically ventilated patients admitted from 03/2020 to 11/2021 to our ICU and all consecutively critically ill mechanically ventilated patients from 08/2020 to 01/2021 and from 03/2021 to 08/2021 admitted to our non-COVID-19 ICU. Patients’ demographics, comorbidity including Charlson Comorbidity Index (CCI), outcome, as well as, admission, maximum and minimum creatinine blood values, as well as KDIGO stage were recorded. Two patient groups, i.e., COVID-19 and non-COVID-19 patients were compared in terms of AKI.

**Results:** The study included 333 patients (183 COVID-19, 150 non–COVID-19), of an average age 66.3 ± 14.36 years-old. Between the two patient groups there was no difference in age or sex. COVID-19 patients had a lower CCI score (84% had a score of < 5 compared to 68.8%, *p* = 0.004). COVID-19 patients had a lower admission creatinine (1.13 ± 0.78 mg/dl vs 1.49 ± 1.33 mg/dl, *p* 0.003), nevertheless, developed more often AKI (74.3% vs 54%, *p* 0001) during their ICU hospitalization. Among COVID-19 ICU patients that developed AKI 54.4% were stage 1, 18.8% stage 2 and 26.8% stage 3, while 10.27% (19/185) of patients underwent CRRT. Twenty-eight-day mortality was high in COVID-19 patients (66.18%, 90/136). There was no difference in KDIGO stage percentage among the two groups.

**Conclusions:** COVID-19 critically-ill patients develop more often AKI compared to non-COVID-19 patients. More studies are required to assess this phenomenon, focusing also on the long-term follow-up of the kidney function of these patients.

## P302

### Impact of CCL-14 on clinical practice after severe AKI

#### T Vilaça, I Palmares, P Freitas, S Coelho

##### Hospital Prof. Dr. Fernando Fonseca, Intensive Care, Amadora, Portugal

*Critical Care* 2023, **27(S1)**: P302

**Introduction:** Urinary chemokine C–C ligand 14 (CCL-14), an immunological mediator, is a promising biomarker of renal functional non recovery after acute kidney injury (AKI) [1, 2]. We aimed to evaluate the impact of its use in clinical practice.

**Methods:** A single-center, prospective, observational study of critically ill patients with stage 2 or 3 AKI by KDIGO criteria on ICU admission was performed. CCL-14 was measured on fresh urine within 48 h of admission and 48 h afterwards using the NEPHROCLEAR TM CCL14 Test (Astute Medical ®). Patients with stage 5D chronic kidney disease; expected length of stay in ICU < 48 h; renal replacement therapy (RRT) in the week prior to ICU admission; and patients with a clinical decision to start RRT prior to study inclusion, were excluded. A questionnaire was made to the clinician in charge of the patient before and after each CCL-14 measurement.

**Results:** Thirty-nine patients were included, of which 58% men, 90% caucasian. 66.3 year-old, median APACHE II 24 and SAPS II 54. 23% developed persistent AKI and 7 required RRT. Patients with persistent AKI, had a first CCL14 median value of 7.49 and second of 4.5. Those who required RRT, had a median CCL14 value of 7.34 ng/ml. Patients with transitory AKI, had a first CCL14 median value of 1.2 and second of 1.05. When asked, 2.6% and 7.7% of clinicians answered RRT should be started; 23.1% and 30.8% believed RRT would be needed in the next 48/72 h; 71.8% and 66.6% thought drugs dose should be adjusted, before and after knowing the CCL14 value respectively. 20% changed their fluidtherapy goal after knowing CCL14 value.

**Conclusions:** CCL-14 may be useful for clinical practice decisions and future studies are needed to evaluate its use on hard outcomes.


**References**
Coelho S et al. Int Urol Nephrol. 2022;54:2047–2055.Hoste E et al. Intensive Care Med. 2020;46:943–953.


## P303

### Impact of urinary chemokine C–C ligand 14 (CCL14) on prognosis of patients with severe acute kidney injury

#### T Vilaça, I Palmares, P Freitas, S Coelho

##### Hospital Prof. Dr. Fernando Fonseca, Intensive Care, Amadora, Portugal

*Critical Care* 2023, **27(S1)**: P303

**Introduction:** Knowing the potential for persistent AKI (vs recovery of renal function) is crucial, as these patients are at increased risk for adverse outcomes, including 90-day mortality, renal replacement therapy, and persistent renal dysfunction. We aimed to evaluate urinary chemokine C–C ligand 14 (CCL14) as a valid biomarker to predict persistent severe acute kidney injury.

**Methods:** A single-center, prospective, observational study of critically ill patients with stage 2 or 3 AKI by KDIGO criteria on ICU admission was performed. CCL-14 was measured on fresh urine within 48 h of admission and 48 h afterwards using the NEPHROCLEAR TM CCL14 Test (Astute Medical ®). Patients with stage 5D CKD; expected length of stay in ICU < 48 h; renal replacement therapy (RRT) in the week prior to ICU admission; and patients with a clinical decision to start RRT prior to study inclusion, were excluded.

**Results:** Thirty-nine patients were included. Of these, 18 patients had an initial CCL14 value of less than 1.3 ng/ml, 19 with a value ≥ 1.3 to < 13 ng/ml, and two patients with dosages greater than 13 ng/ml. In 67% of cases there was no change in the value range in both assessments. 23% (n = 9) developed persistent acute kidney injury, the vast majority with stage 3 AKI on admission. CCL14 was significantly higher in end point positive at enrollement (7.34 ng/ml vs 1.22 ng/ml). CCL14 dosing higher than 1.3 ng/mL was associated with an increased risk of developing persistent AKI, odds ratio (OR) 2 (95% CI 0.4–9.5). High CCL14 values are also associated with the need for RRT in the first 5 days. 7 patients required RRT, with a median CCL14 value of 7.34 ng/ml; OR 2.6 (95% CI 0.4–15.8).

**Conclusions:** Elevated CCL14 values correlate with the development of persistent severe acute kidney injury. These data may lead to a paradigm shift in the approach of these patients.

## P304

### Donor lactate levels at hospital admission can help predict renal graft outcomes in kidney donation after Maastricht II circulatory death

#### AC Gaspar1, M Gama^2^, G Nobre de Jesus^1^, J Oliveira^3^, M Neves^3^, A Santana^3^, JM Ribeiro^1^

##### ^1^Hospital de Santa Maria, Centro Hospitalar Lisboa Norte, EPE, Intensive Care Department, ECMO Referral Center, Lisbon, Portugal, ^2^Hospital de Santa Maria, Centro Hospitalar Lisboa Norte, EPE, Transplant Coordination Department, Lisbon, Portugal, ^3^Hospital de Santa Maria, Centro Hospitalar Lisboa Norte, EPE, Nephrology and Transplantation Department, Lisbon, Portugal

*Critical Care* 2023, **27(S1)**: P304

**Introduction:** In recent years, lactate levels have been associated with neurological outcomes in patients undergoing extracorporeal cardiopulmonary resuscitation (ECPR). We hypothesize that donor lactate level could also be a good predictor for immediate graft function after uncontrolled kidney donation after refractory cardiocirculatory death (uDCCD) Maastricht class II.

**Methods:** A retrospective, non-interventional, ethical committee approved study was designed in a university tertiary hospital, ECMO referral Centre. Data was collected from electronic files of donors and recipients. Regarding the donors, warm ischemia time and lactate levels were gathered. Regarding the recipients, immediate graft function and creatinine at 1 month follow-up were analyzed. For statistical analysis we used SPSS® software.

**Results:** From 2017 until May 2022, twenty-one effective donors were identified in our ECPR/uDCCD program. Thirty-one grafts were transplanted. Mean age of donors was 46.7 years and 16 were male. Time from cardiac arrest to hospital admission was 15 ± 8 min, mean lactate level at admission was 21.4 ± 0.5 mmol/l. Regarding immediate graft outcomes, 29% (n = 9) had immediate graft function, 61.3% (n = 19) had delayed graft function (defined as need for dialysis in the first week after transplant) and 9.7% (n = 3) had primary non-function. Using ROC testing, we identified that a lactate value of 19.9 mmol/l at admission had a 100% sensitivity and 82.1% specificity to estimate primary graft non-function. We also detected a correlation between higher lactate values at admission and recipient creatinine at one-month follow-up (*p* = 0.000).

**Conclusions:** In our study, lactate level of potential donors at hospital admission was correlated with the risk for primary graft non-function after kidney transplantation after uDCCD. Lactate level of donors can also affect other outcomes, which needs to be confirmed with a larger series and prospective studies.

## P305

### Protective effects of pentoxifylline on renal microcirculatory oxygenation and perfusion in sepsis-induced AKI in rats

#### B Ergin^1^, H.R Taal^2^, I Reiss^2^, C Ince^1^

##### ^1^Erasmus Medical Center, The Department of Intensive Care, Rotterdam, Netherlands, ^2^Erasmus Medical Center, The Department of Neonatology, Rotterdam, Netherlands

*Critical Care* 2023, **27(S1)**: P305

**Introduction:** Acute kidney injury (AKI) remains one of the most frequent conditions associated with high mortality rate amongst septic patients in the ICU. In this study we hypothesized that the microcirculatory targeting therapy using the phosphodiesterase inhibitor, pentoxifylline (PTX), may have a potential beneficial effect to improve renal microcirculatory perfusion and oxygenation thereby ameliorate the development of AKI.

**Methods:** In this study, 35 fully instrumented, mechanically ventilated, anesthetized Wistar rats were randomly divided into 5 groups as sham, LPS group, LPS + PTX group, LPS + resuscitation with Ringer’s acetate (RA) group and LPS + PTX + RA group. Sepsis was induced by i.v. injection of 10 mg/kg LPS until the mean arterial pressure (MAP) reached below 60 mmHg followed by 3 h resuscitation or/and 60 mg/kg PTX infusion. The systemic and renal hemodynamics, renal cortical oxygenation (measured by phosphorescence technique and Hb spectrophotometry (O2C)), renal damage and inflammatory parameters were assessed.

**Results:** PTX increased the HR at T3 and T4 in comparison to the other groups (*p* < 0.05) (Fig. 1A). The renal cortical oxygenation, renal capillary oxygen saturation and flow were improved in both the PTX and RA groups in comparison to the LPS group (*p* < 0.05) (Fig. 1B–D). PTX protected the endothelial glycocalyx as shown by the reduction in HA levels (*p* < 0.05) (Fig. 1F). Lastly, PTX showed also protective effect on the muscle microcirculatory functional parameter such as functional capillary density and RBC velocity (*p* < 0.05 vs. LPS) (Fig. 1G–H).

**Conclusions:** Pentoxifylline has a protective effect on the renal oxygenation, RBC perfusion and oxygen availability of the kidney, and on the muscle microcirculation during sepsis. Our study strongly suggests that PTX could be effectively used as an adjuvant therapy to ameliorate AKI and microcirculatory dysfunction seen as a consequence of sepsis. The results of this study should be further investigated in randomized clinical study on septic patients.

**Figure Figdr:**
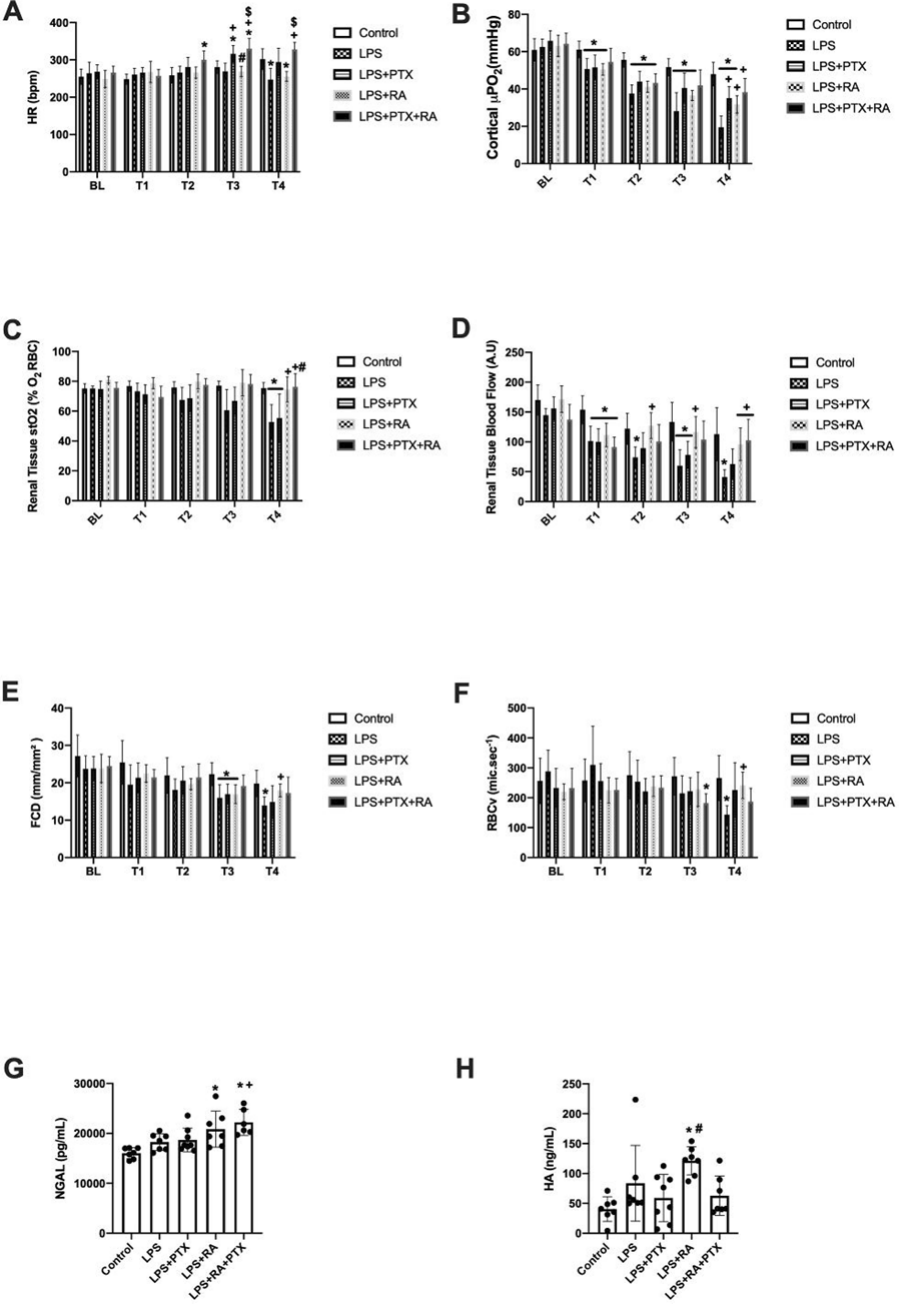
**Fig. 1 (abstract P305)** The changes in heart rate (HR), cortical microvascular oxygen pressure (cortical μPO_2_), renal capillary oxygen saturation, renal capillary blood flow, neutrophil-gelatinase lipocalin (NGAL) hyaluronic acid (HA), muscle functional capillary density and red blood cell velocity (RBCv) in all groups. Values are represented as mean ± SD, **p* < 0.05 versus Control, + *p* < 0.05 versus LPS, #*p* < 0.05 versus LPS + PTX, $*p* < 0.05 vs. LPS + RA

## P306

### Incidence and progression of perioperative renal injury in patients undergoing an emergency laparotomy

#### S Patel^1^, G Boniface^2^, K Millar^1^, H Mulrenan^3^, O Eastwood^4^, R Breeze^5^

##### ^1^Guys and St Thomas’ NHS Trust, Anaesthetics Department, London, UK, ^2^Lewisham and Greenwich NHS Trust, Anaesthetics Department, London, UK, ^3^Lewisham and Greenwich NHS Trust, Department of Medicine, London, UK, ^4^Guys and St Thomas’ NHS Trust, Department of Medicine, London, UK, ^5^Lewisham and Greenwich NHS Trust, Anaesthetics and Critical Care, London, UK

*Critical Care* 2023, **27(S1)**: P306

**Introduction:** This study aims to determine the incidence of acute kidney injury (AKI) in patients undergoing emergency laparotomy, and whether this correlated with subsequent progression to chronic kidney disease (CKD). Perioperative AKI is an important contributor to morbidity and prolonged hospital stay [1]. In addition, individuals with AKI are nine times more likely to develop CKD, a progressive and life-threatening disease [2]. Despite this, there is limited literature of the incidence of perioperative AKI & correlation with progression to CKD.

**Methods:** A retrospective observational cohort study was conducted using patients identified from the National Emergency Laparotomy Audit (NELA) database over a 2-year period within a single London hospital setting. 253 patients were identified. Creatinine and eGFR were collected from premorbid baseline, admission and day one postoperatively and used to determine perioperative AKI. Creatinine and eGFR were collected at 6 weeks and 3 months to determine progression to CKD. KDIGO (Kidney Disease Improving Global Outcomes) criteria for AKI and CKD were used [3].

**Results:** 16.6% of patients had an AKI on admission with 5.8% of those having a persistent AKI postoperatively. 11.5% of the patients without an AKI on admission developed an AKI immediately postoperatively. 0 patients from either cohort had an AKI at 1 week postoperatively or developed CKD. 2.3% of patients had CKD on admission, but this did not progress over the time period studied.

**Conclusions:** In this small, single centre pilot study, there was not a single case of perioperative AKI progressing to CKD. More research is required on a multicentre level to further describe the incidence of perioperative AKI and subsequent progression to CKD.

## References


Hobson et al. Ann Surg 2015;261:1207–1214.Devarajan et al. Prog Pediatr Cardiol 2016;41:33–40.KDIGO 2012 Clinical Practice Guideline for the Evaluation and Management of Chronic Kidney Disease.

## P307

### Prediction of initiation of renal replacement therapy in the intensive care unit

#### Z Zaiman^1^, P Morandini^2^, F Alfieri^3^, MR Botella^4^, X Prados^5^, P Ortiz^6^, CM Sauer^7^

##### ^1^Emory University, Atlanta, USA, ^2^Humanitas Research Hospital, Milan, Italy, ^3^U-Care Medical, Turin, Italy, ^4^Instituto de Investigación Sanitaria Pere Virgili, Tarragona, Spain, ^5^Hospital Universitari Arnau de Vilanova, Lleida, Spain, ^6^Hospital Universitari Doctor Josep Trueta, Girona, Spain, ^7^University Hospital Essen, Essen, Germany

*Critical Care* 2023, **27(S1)**: P307

**Introduction:** Acute kidney injury (AKI) is a common issue in the intensive care unit affecting approx. 57% of all patients [1]. Furthermore, it is a common reason for initiating continuous renal replacement therapy (CRRT). Early identification of patients requiring CRRT could help physicians to focus on preventing progression to CRRT, improve planning and possibly even patient outcomes. Here, we developed a CRRT prediction algorithm that uses patient data available within the first 12 h of admission to predict initiation of CRRT within the first five days of admission.

**Methods:** All adult patients with AKI stage 1–3 and sepsis (Sepsis-3 criteria) on their first ICU stay were extracted from the MIMIC-IV database (Boston, MA, USA [2]). Baseline characteristics including demographics, vital signs, and laboratory measurements from the first 12 h of admission were extracted. Initiation of CRRT within 12–120 h after admission was the outcome. We built a predictive logistic regression model using 70% of the cohort and evaluated model performance in a 30% validation data subset. We performed majority class undersampling to address class imbalance.

**Results:** We included a total of 12,683 patients into our final cohort. 5% received CRRT in the study period. The four most predictive covariates for the initiation of CRRT were the minimum and maximum values of pH and the minimum and maximum values of creatinine. The logistic regression model achieved an AUROC of 0.913 on the test data set, which was similar across AKI stages at admission (0.83–0.93) and the timing of CRRT initiation AUROC (0.88–0.92). Precision and recall were similar for those receiving (0.82 and 0.83) and those not receiving CRRT (0.82 and 0.82, respectively) (Fig. 1).

**Conclusions:** Utilizing single center ICU data we showed that initiation of CRRT can be accurately predicted. Next, we will investigate how this model can be transferred to other ICUs worldwide.


**References**
Hoste EAJ et al. Intensive Care Med. 2015;41:1411–23.Johnson A et al. PhysioNet. 2022.

**Figure Figds:**
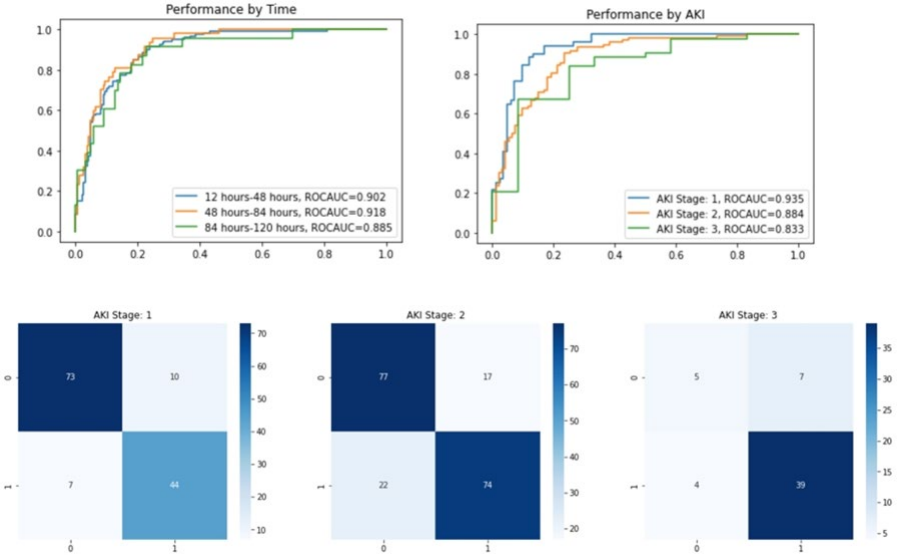
**Fig. 1 (abstract P307)**. Model performance. Left: AUROC for the predictive model by time of initiation of CRRT. Right: AUROC for the predictive model by AKI stage. Bottom: Confusion matrix showing actual versus predicted initiation of CRRT by AKI stage in the test data set

## P308

### Clinical profile and outcomes of patients with COVID-19 respiratory failure requiring renal replacement therapy: a machine learning analysis of 13,576 patients

#### AF França^1^, LSB BAstos^2^, ER Rocha^3^, EM Maccariello^3^, JRLS Lapa e Silva^4^, JIFS Salluh^5^

##### ^1^UFRJ, Critical Care, Rio de Janeiro, Brazil, ^2^PUC, Engineering, Rio de Janeiro, Brazil, ^3^D’Or Institute for Research and Education, Nephrology, Rio de Janeiro, Brazil, ^4^UFRJ, Pulmonology, Rio de janeiro, Brazil, ^5^D’Or Institute for Research and Edcucation, Critical Care, Rio de Janeiro, Brazil

*Critical Care* 2023, **27(S1)**: P308

**Introduction:** Acute kidney injury (AKI) is a frequent and severe complication of COVID-19 infection in ICU patients. We propose a structured data-driven methodology and develop a model to predict the use of renal replacement therapy for patients on respiratory support with Covid-19 in 126 ICUs from 42 Brazilian hospitals.

**Methods:** Adult ICU patients (March 2020–December 2021) with confirmed SARS-CoV-2 infection and need of ventilatory support at D1 admission in the ICU. Main outcome was the need of RRT. We estimated 3 prediction models: Logistic Regression (LR), Random Forest (RF) and XGB Boosting. Models were derived in the training set and evaluated in the test set following an 80/20 split ratio, and models' parameters were selected using fivefold cross-validation. We evaluated and selected the best model in terms of discrimination (AUC) and calibration (Brier’s Score). Variable importance was estimated for each predictor variable.

**Results:** 13,575 ICU patients with need of respiratory support, of which 1828 (14%) needed RRT. ICU and hospital mortality were respectively 15.7%, 20.3% (non-RRT) and 54.3%, 69% (RRT). Mean age was 63.9 and 55.3 years (RRT vs non-RRT). Mean ICU LOS was 27.8 vs. 12 days, in RRT vs non-RRT. RF and XGB models both showed higher discrimination performance compared to LR (95% confidence interval [95% CI]: 0.84 [0.81–0.85] and 0.83 [0.80–0.85] vs 0.78 [0.75–0.80]). RF and XGB models presented similar calibration (Brier’s Score: ([95% CI]: 0.09 [0.09–0.10] and 0.09 [0.09–0.10]), also better than in LR (0.11 [0.10–0.12]). The final model (RF) showed no sign of under or overestimation of predicted probabilities in calibration plots.

**Conclusions:** The need of RRT among patients on respiratory support diagnosed with Covid-19 was accurately predicted through machine learning methods. RF and XGB based models using data from general intensive care databases provides an accurate and practical approach for the early prediction of use of RRT in COVID-19 patients.

## P309

### Predicting early and successful liberation from renal replacement therapy in critically ill patients with acute kidney injury using proenkephalin a 119–159: a post-hoc analysis of the ELAIN trial

#### T Von Groote^1^, F Albert^2^, M Meersch-Dini^1^, C Porschen^1^, M Sadjadi^1^, O Hartmann^3^, D Bergmann^3^, P Pickkers^4^, A Zarbock^1^

##### ^1^University Hospital Münster, Department of Anaesthesiology, Intensive Care and Pain Medicine, Münster, Germany, ^2^University of Münster, Institute of Biostatistics and Clinical Research, Münster, Germany, ^3^SphingoTec GmbH, Henningsdorf, Germany, ^4^Radboud University Medical Center, Department of Intensive Care Medicine, Nijmegen, Netherlands

*Critical Care* 2023, **27(S1)**: P309

**Introduction:** Currently, clinical decisions on when to terminate renal replacement therapy (RRT) in critically ill patients with acute kidney injury (AKI) are often based on clinical judgment and return of spontaneous diuresis alone; however, this approach faces important limitations. Other markers of kidney function such as serum creatinine are distorted during RRT and therefore not utilizable. Proenkephalin A 119–159 (penKid) has been described as a novel biomarker to assess kidney function even during non-stable state, such as critical illness or sepsis. Therefore, we hypothesized that penKid may be a competent biomarker to assess recovering kidney function during ongoing continuous RRT.

**Methods:** This post-hoc analysis included 210 critically ill adult patients with AKI from the randomized ELAIN trial. PenKid levels were measured in the blood of these patients. Competing risk time-to-event analysis were performed for penKid at initiation of RRT and in a landmark analysis at day 3 after RRT initiation. Competing risk endpoints were successful liberation from RRT or death without prior liberation from RRT.

**Results:** Manufacturer cut-off of 89 pmol/l confirmed by optimal cut-off analysis. Low penKid levels (≤ 89 pmol/l) at RRT initiation were associated with early and successful liberation from RRT compared to patients with high penKid levels (Fig. 1a), estimated 28d-cumulative incidence function (CIF) of successful RRT liberation 61% vs. 45%, *p* = 0.022). This association persisted in the landmark analysis on day 3 of RRT (Fig. 1b, 28d CIF of successful liberation from RRT 67% vs. 47%, *p* = 0.018). For both timepoints, no difference in the competing event of death was detected.

**Conclusions:** This post-hoc analysis demonstrates that penKid might be a useful novel biomarker for the prediction of shorter duration and successful RRT liberation in critically ill patients, providing an individualized approach to guide RRT strategies. These findings were recently published [1].


**Reference**
von Groote T et al. Crit Care 2022;26:333.

**Figure Figdt:**
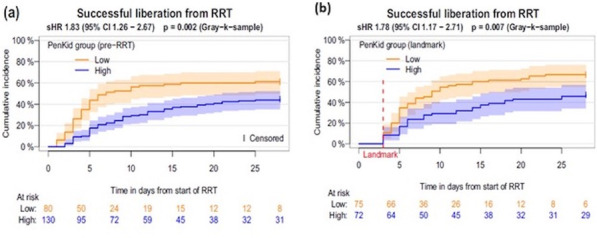
**Fig. 1 (abstract P309)**. Competing risks time-to-event analyses: liberation from RRT at (**a)** baseline; (**b**) day 3 of RRT

## P310

### Significant reduction of the standardized mortality ratio in patients with multiple organ failure treated with the advanced organ support (ADVOS): analysis of the registry on extracorporeal multiple organ support for patients with documented SOFA scores

#### V Fuhrmann^1^, A Perez^2^, A Faltlhauser^3^, B Tyczynski^4^, D Jarczak^1^, J Weinmann-Menke^5^, M Sander^6^, S Kluge^1^

##### ^1^Universitätsklinikum Hamburg-Eppendorf, Klinik für Intensivmedizin, Hamburg, Germany, ^2^ADVITOS GmbH, München, Germany, ^3^Klinikum Weiden, Medizinische Klinik 1., Weiden, Germany, ^4^Universitätsklinikum Essen, Klinik für Nephrologie, Essen, Germany, ^5^Universitätsmedizin Mainz, I. Medizinische Klinik und Poliklinik, Mainz, Germany, ^6^Universitätsklinikums Gieβen & Marburg GmbH am Standort Gieβen, Anästhesiologie, operative Intensivmedizin und Schmerztherapie, Gieβen, Germany

*Critical Care* 2023, **27(S1)**: P310

**Introduction:** The EMOS-Registry (DRKS00017068) is intended to collect real world evidence in patients treated with the ADVOS hemodialysis system. The current analysis focused on mortality in patients with documented SOFA scores.

**Methods:** In this third analysis [1], all patients enrolled until August 31, 2020, were assessed for eligibility. Patients with documented SOFA scores immediately before the first ADVOS session were included. Demographic characteristics, treatment settings, adverse events, and the SOFA Score-standardized mortality ratio (SMR) were analyzed. For the latter, patients were divided according to their SOFA score, and the expected mortality [2] was compared to the observed hospital and 28-day mortality.

**Results:** 202 patients from 5 German clinics were included. 67% were male, with a median age of 58 years. 89% received vasopressors, 73% were on mechanical ventilation and 52% had acidosis. The median SOFA Score was 15 (IQR 12, 18) and 91% had multiple organ failure. 739 ADVOS sessions were prescribed with a median of 3 (IQR: 2, 5) per patient and a duration of 19 h (IQR: 12, 23) at low blood flows (100 ml/min (IQR: 100, 150)). Less than 5% of the sessions were aborted due to device-related problems. Only 2% of the adverse events (e.g., clotting) were attributed to the ADVOS multi by the users. According to the SOFA Score 169 deaths (84%) were expected, while 134 (66%) and 119 (59%) were observed in the Hospital and after 28 days, respectively. This resulted in a SMR of 0.79 (CI 95%: 0.66–0.93) with a number needed to treat (NNT) of 5.8 and 0.70 (0.58–0.83) with a NNT of 4, respectively (Fig. 1).

**Conclusions:** Registry data must be carefully analyzed due to the inherent limitations of these studies. However, these real-world data show a statistically significant improvement of the SOFA Score-SMR in critically ill patients treated with the ADVOS hemodialysis system.


**References**
Fuhrmann V et al. Medicine (Baltimore). 2021;100:e24653.Ferreira FL et al. JAMA. 2001;286:1754–8.

**Figure Figdu:**
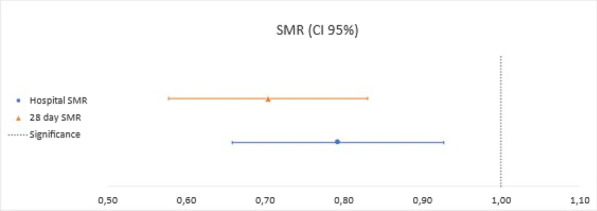
**Fig. 1 (abstract P310)**. SOFA score-standardized mortality ratio

## P311

### Outcomes of renal replacement therapy in critically ill COVID-19 patients

#### P Costa^1^, J Alves^1^, A Martinho^1^, C Silva^1^, L Simões^1^, G Almeida^1^, J Guimarães^2^, S Teixeira^1^, JP Baptista^1^, P Martins^1^

##### ^1^Coimbra’s University Hospital, Intensive Care Department, Coimbra, Portugal, ^2^Coimbra’s University Hospital, Cardiology Department, Coimbra, Portugal

*Critical Care* 2023, **27(S1)**: P311

**Introduction:** Our goal is to describe outcomes of critically ill COVID-19 patients submitted to renal replacement therapy (RRT), in particular the association of RRT with mortality. Multi-system organ failure or direct kidney injury caused by SARS-CoV-2 is associated with the development of acute kidney injury (AKI) which subsequently increases the need for RRT and may affect the outcomes.

**Methods:** This is a retrospective observational study of 338 critically ill patients admitted with COVID-19 pneumonia in the ICU of a tertiary center in Portugal, between March 2020 and December 2021. Clinical, analytical and baseline patient characteristics were evaluated. Logistic regression analysis was performed to correlate patient data with the need for RRT and ICU mortality.

**Results:** From a total of 338 patients, 5% required RRT (n = 16), 25% of which received intermittent hemodialysis (n = 4) and 87,5% continuous veno-venous hemofiltration (n = 14). Baseline characteristics are described in Table 1. In our sample, 61 patients (18%) presented with acute AKI, from whom 14 (23%) were submitted to RRT. From all the patients receiving RRT, 10 (62.5%) did not have pre-existing chronic kidney disease. In the logistic regression analysis, AKI (OR 45.4; 95% CI 7.7–269.5; *p* < 0.001), higher SOFA (OR 1.24; 95% CI 103–1.51; *p* = 0,03), creatinine (OR 2.01; 95% CI 1.4–3.0; *p* < 0.001) and C-reactive protein (OR 1.09; 95% CI 1.02–1.16; *p* = 0,01) on admission were associated with the need for RRT. Additionally, ICU mortality associated with RRT was 75% compared to 28.3% in the group not submitted to RRT (OR 7.6; 2.4–24.2; *p* = 0.001).

**Conclusions:** The need for RRT in critically ill COVID-19 patients is associated with an increased mortality rate in our study. We were also able to identify AKI, higher SOFA, creatinine and C-reactive protein at admission as risk factors for RRT. However, due to the retrospective nature of our analysis and our small sample size, more studies on this topic are needed to confirm these results.

**Table Tabdg:** **Table 1 (abstract P311)**. Baseline patient characteristics

	RTT (n = 16)	Without RRT (n = 322)	*p* value
Male sex (%)	75.0%	66.8%	0.49
Age (years)	60.7 (± 13.3)	59.1 (± 12.9)	0.62
Charlson comorbidity index	2.7 (± 1.7)	2.1 (± 1.7)	0.19
SOFA at admission	9.6 (± 3.2)	5.9 (± 2.8)	< 0.001
Invasive mechanical ventilation (days)	17.6 (± 16.0)	16.1 (± 12.0)	0.65
ICU (days)	14.8 (± 13.6)	17.1 (± 12.3)	0.47
PaO_2_/FiO_2_ ratio at admission	95.0 (± 32.0)	113.4 (± 48.5)	0.14

## P312

### Distance between central venous catheter tips can affect antibiotic clearance during continuous renal replacement therapy

#### A Bandert^1^, M Lipcsey^2^, R Frithiof^3^, A Larsson^4^, D Smekal^3^

##### ^1^Centre for Research and Development, Uppsala University/Gävleborg, Dept of Surgical Science, Gävle, Sweden, ^2^Hedenstierna Laboratory, Uppsala University, Dept of Surgical Science, Uppsala, Sweden, ^3^Dept of Surgical Science, Uppsala University, Dept of Surgical Science, Uppsala, Sweden, ^4^Dept of Medical Science, Uppsala University, Dept of Medical Science, Uppsala, Sweden

*Critical Care* 2023, **27(S1)**: P312

**Introduction:** The aim of this experimental study was to elucidate if different distances between central venous catheter tips can affect drug clearance, during continuous renal replacement therapy (CRRT). Central venous catheters (CVC) are often used in intensive care patients for drug infusion. If a patient receives CRRT, a second central dialysis catheter (CDC) is required. Where to insert CVCs is directed by guidelines but there are no recommendation how to place multiple catheters [1]. If a drug is infused in a CVC with the tip close to the CDC, it could be aspirated into the dialysis machine and by that, increasing the risk of elevated drug clearance [2]. This phenomenon of direct aspiration has been described in vitro*,* in case studies and in one experimental study [2–4]. If there is a difference in clearance, when CVC and CDC tips are at different positions is less described.

**Methods:** In this experimental model with 18 pigs, gentamicin (GM) and vancomycin (VM) were infused through a CVC, during CRRT. Clearance was measured when the tip of the CVC was 2 cm caudally of the tip of the CDC, at the same level and at 2 and 4 cm cranially of the tip of the CDC. Position was controlled by chest radiography. Serum and dialysate concentrations were sampled after 20 min of CRRT at each position. Clearance was calculated. Repeated measure ANOVA was performed, level of significance was set to *p* < 0.05.

**Results:** When GM and VM were infused through a CVC the distances between the CVC and CDC tips affected clearance of GM (F = 2,91, *p* = 0.05) and VM (F = 2.98, *p* = 0.04), during CRRT (see Fig. 1). Clearance was higher when infusion was in a CVC tip cranially to the CDC tip.

**Conclusions:** The distance between CVC and CDC tips can affect drug clearance, during CRRT.


**References**
Frykholm P et al. Acta Anaesthesiol Scand 2014;58:508–24.Frithiof R et al. ASAIO J 2019;65:408–13.Stricker KH et al. Anesth Analg 2009;109:1640–3.Kam KY et al. Anaesthesia 2012;67:115–21.

**Figure Figdv:**
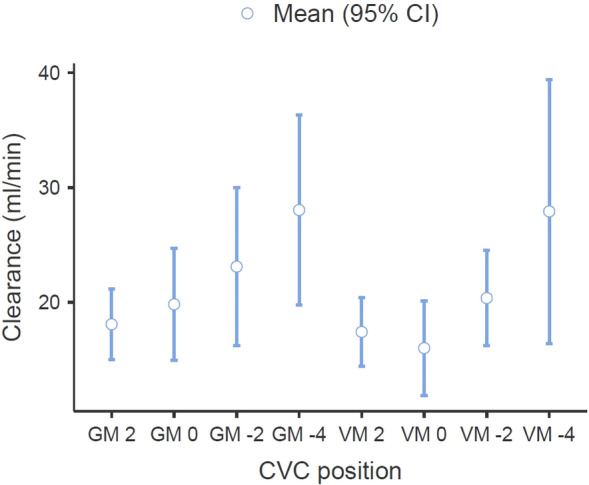
**Fig. 1 (abstract P312)**. Gentamicin (GM) and vancomycin (VM) were infused in a central venous catheter (CVC) during continuous renal replacement therapy (CRRT). The CVC tip was placed in different positions in relation to the central dialysis catheter (CDC), 2 cm caudally (GM 2 and VM 2), at the same tip level (GM 0 and VM 0), 2 cm cranially (GM-2 and VM-2) and 4 cm cranially (GM-4 and VM-4). Clearance was measured at the different positions

## P313

### Comparative analysis between mixed venous-to-arterial CO_2_ gap and lactate values during liver transplantation

#### D Rodriguez Villar, B Villa Cigales, P Gómez Aguilar, L Martínez Botet, J Villahoz, L Gajate

##### Hospital Universitario Ramón y Cajal, Anesthesiology, Madrid, Spain

*Critical Care* 2023, **27(S1)**: P313

**Introduction:** Patients going through liver transplantation suffer hemodynamic and metabolic changes. Ensuring adequate tissue perfusion is essential in this situation. Mixed venous-to-arterial CO_2_ tension gap [p_(v−a)_CO_2_] defined as the difference between mixed venous blood pCO_2_ and arterial pCO_2_ has been used as a sensitive marker of the microcirculatory state in patients with septic shock. This study analises the correlation between p_(v−a)_CO_2_ and lactate values during liver transplantation.

**Methods:** A correlation analysis between lactate and p_(v−a)_CO_2_ was conducted at the different stages of liver transplantation at Ramon y Cajal Hospital. The Shapiro–Wilk normality test was used to rule out normal distribution. Spearman’s correlation between lactate and p_(v−a)_CO_2_ was assessed. *p* value < 0.05 was defined as statistically significant.

**Results:** Perioperative data of 98 patients were collected. Lactate median at baseline was 1.2 mmol/l (IQR 0.6) and p_(v−a)_CO_2_ median 4 mmHg (IQR 5). Spearman’s correlation coefficient was r = 0.25 (*p* = 0.03). Lactate median in anhepatic phase was 4 mmol/l (IQR 1,1) and p_(v−a)_CO_2_ median 4 mm Hg (IQR 4.8). Spearman’s correlation coefficient was r = −0.1 (*p* = 0.2). Lactate median in neohepatic phase was 3.1 mmol/l (IQR 1.3) and p_(v−a)_CO_2_ median 3 mmHg (IQR 5). Spearman’s correlation coefficient was r = −0.1 (*p* = 0.07). Lactate median at ICU admision was 2.25 mmol/l (IQR 2.2) and p_(v−a)_CO_2_ median 4 mmHg (IQR 4). Spearman’s correlation coefficient was r = −0.01 (*p* = 0.9). Lactate median 24 h after liver transplantation was 1.2 mmol/l (IQR 1.1) and p_(v−a)_CO_2_ median 4 mmHg (IQR 3). Spearman’s correlation coefficient was r = −0.1 (*p* = 0.4).

**Conclusions:** We only found correlation between lactate and p_(v−a)_CO_2_ in baseline measurements before surgery. There was no correlation during the other phases of liver transplantation which leads us to think maybe p_(v−a)_CO_2_ could be a better indicator of microcirculatory state and tisular perfusion in these patients, since lactate clearance is dependent on liver function.

## P314

### Gastroesophageal reflux disease and its health-related quality of life among adult ICU survivors

#### S Wali, M Gurjar, S Gutte, S Mohindra, B Poddar, A Azim, V Kapoor, P Mishra

##### Sanjay Gandhi Postgraduate Institute of Medical Sciences, Critical Care Medicine, LUCKNOW, India

*Critical Care* 2023, **27(S1)**: P314

**Introduction:** We aimed to study gastroesophageal reflux disease (GERD) prevalence, its health-related quality of life (GERD-HRQL) and risk factors for new-onset GERD in ICU survivors.

**Methods:** After ethics approval, this prospective study done at 20-bed ICU of a university hospital (Trial Identifier NCT05193266). GERD is defined as presence of heartburn and/or regurgitation symptom ≥ 2 times a week (World Gastroenterology Organization-2017). All adult survivors screened at ICU discharge. Exclusion criteria: ICU stay < 96 h, presence of GCS < 15, feeding or tracheostomy tube, abdominal drain/surgery and pregnancy. Patients having GERD assessed for GERD-HRQL score and its changes over 6 weeks follow-up.

**Results:** During study period (Jan-Aug 2022), 151 admitted and 105 survived. After exclusion of 34 patients, 71 included with age 42 (28–58) years, 60% male, SOFA score 7 (4–11) and Charlson comorbidity index 0 (0–3). ICU stay was 15 (6–31) days, 59% needs ventilator (MV), 45% vasopressor; 14% dialysis (RRT). Feeding tube (NG) present in 76% (15 days) and tracheal tube (TT) in 46% (18 days). Sedation (49%), steroid (37%), prokinetic (39%) and other at-risk drug used in 62%. 23 patients (32%) had GERD prior to current illness (Fig. 1a). In remaining 48 patients, 17 (24%) had new-onset GERD with OR [95% CI] for risk factors: age 0.95 [0.9–0.9] (*p* = 0.02), comorbidity 0.92 [0.7–1.2], SOFA score 0.92 [0.7–1.2], GI disease 0.97 [0.3–3.2], ICU stay 1.0 [0.9–1.0], MV 1.33 [0.4–4.4], vasopressor 1.2 [0.3–4.2], RRT 0.69 [0.1–4.0], NG days 1.0 [0.9–1], TT days 0.99 [0.9–1.0], sedation 1.78 [0.5–5.9], steroid 0.66 [0.1–2.3], prokinetic 0.66 [0.1–2.3] and at-risk drugs 0.3 [0–1]. In follow-up, GERD-HRQL score improved significantly in new onset GERD group from 9 (6–14) to 4 (3–9) (*p* = 0.002); while didn’t in pre-illness GERD group from 3 (0–7) to 3 (0–8) (*p* = 0.51) (Fig. 1b, c).

**Conclusions:** One-fourth ICU survivors had new onset GERD; and its HRQL score improved significantly at 6-week follow-up.

**Figure Figdw:**
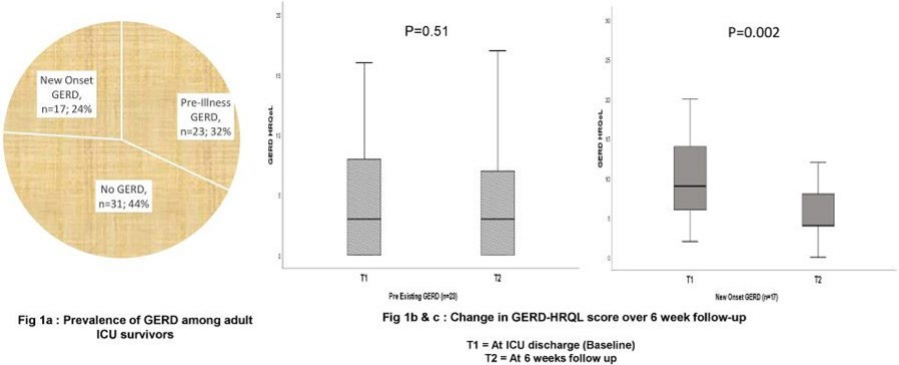
**Fig. 1 (abstract P314)**. **a** Prevalence of GERD among adult ICU survivors; **b**, **c** Change in GERD-HRQL score over 6 week follow-up

## P315

### Mortality, management and risk factors of non-occlusive mesenteric ischemia: evidence from systematic review and population-wide study

#### V Mihnovitš^1^, AL Voomets^2^, K Kase^3^, J Starkopf^2^, A Reintam Blaser^4^, K Tamme^2^

##### ^1^Tartu University Hospital, Intensive Care, Tartu, Estonia, ^2^Tartu University, Institute of Clinical Medicine, Tartu, Estonia, ^3^Tartu University Hospital, Surgery Clinic, Tartu, Estonia, ^4^Lucerne Cantonal Hospital, Center of Intensive Care Medicine, Lucerne, Switzerland

*Critical Care* 2023, **27(S1)**: P315

**Introduction:** The study aim was to characterize the demography, management and mortality of patients with non-occlusive mesenteric ischemia (NOMI).

**Methods:** We performed a substudy on NOMI based on two recent studies [1, 2]. Studies reporting data about NOMI were reviewed and data extracted using a predeveloped form.

**Results:** We reviewed 23 studies with a total of 2865 NOMI patients. Management and mortality are depicted in Table 1. Ten studies with 370 patients reported an average age of 68.8 years (min 37 and max 98). 46.4% of the patients were female in 14 studies (2691 patients) reporting gender. In seven studies (163 patients) reporting localisation of ischemia the small bowel, large bowel and combination of both was described in 30.6%, 31.4% and 38% of cases, respectively. Main risk factors were heart failure (mean 70.5% in four studies in 302 patients), vasopressor use (mean 40.2% in four studies in 1032 patients) and prior surgery (mean 21% in five studies in 114 patients).

**Conclusions:** NOMI seems to affect younger population compared to other forms of acute mesenteric ischemia. Frequently both small and large intestine are involved. Low cardiac output and use of vasopressors are important contributing factors. Overall mortality is high but seems to be lower in the vasodilator therapy group.


**References**
Tamme K et al. BMJ Open. 2022;12:e062846.Kase K et al. World J Surg. 2023;47:173–181.

**Table Tabdj:** **Table 1 (abstract P315)**. The management and mortality of patients with non-occlusive mesenteric ischemia reported in different studies

	Mean, individual patient meta-analysis (%)	Mean of the means reported in the studies (%)	Number of studies reporting	Total number of patients
Mortality	44	56.3	23	1823
Surgical management	68.1	64.3	13	615
Conservative management	36.5	28.7	10	394
Vasodilator therapy	16.4	12	8	1261
Mortality in surgical therapy group	59.2	62.4	9	162
Mortality in conservative therapy group	77.9	75.4	5	86
Mortality in vasodilator therapy group	27.4	32.9	5	197

## P316

### Deep neural network models for detection of strangulating bowel obstruction using contrast enhanced CT

#### N Okada, M Yoshida, R Noda, D Inoue, N Uozumi, H Matsuki, H Shintani, N Kataoka, T Yamaguchi

##### Kishiwada Tokusyukai Hospital, Division of Surgeon, Osaka, Japan

*Critical Care* 2023, **27(S1)**: **P316**

**Introduction:** Strangulating bowel obstruction should be treated immediately. Contrast enhanced CT is widely accepted to classify bowel obstructions. However, the diagnostic imaging criteria are not clearly defined. Accuracy of diagnostic imaging varies with physicians and even specialist can overlook strangulating bowel obstructions. We use AI models to standardize diagnostic imaging and improve clinical outcome of these disease. In this study, we aimed to develop and validate deep neural network (DNN) models detecting strangulating bowel obstruction using contrast enhanced CT.

**Methods:** We retrospectively collected 31,100 anonymous contrast enhanced CT images together with their clinical reports at the Kishiwada Tokusyukai Hospital between January 1, 2020, and December 31, 2022. We excluded incomplete, younger than 7 years old, and poorly depicted images. The part of this dataset from August 1, 2022, until December 31, 2022, was used for external test and the rest for training and validation. We develop and evaluate two binary-classification DNN models: slice model examining a CT slice to find lesions; series model examining a series of CT images to find a suspected patient. Accuracy, and Areas under the receiver operating characteristic curves (AUCs) were used to assess the models.

**Results:** The training and validation dataset contained 25,860 images. The test dataset contained 5240 images. The series model achieved 80% accuracy and an AUC of 0.72 for detecting strangulating bowel obstruction, the slice model achieved 72% accuracy and AUC of 0.62.

**Conclusions:** Our results show that DNN models can potentially identify strangulating bowel obstruction using contrast enhanced CT. Increasing the number of samples, DNN model could accurately detect strangulating bowel obstruction.

## P317

### Safety and feasibility of kefir in the critically ill

#### SSR Rajendraprasad^1^, DR Ramachandran^2^, SZ Zec^3^, JS Skalski^1^, JB Bakken^4^, WDF Freeman^5^, SRA Ahmad^1^, ASS Shah^6^, SK Khanna^7^, LVK Karnatovskaia^1^

##### ^1^Mayo Clinic, Pulmonary and Critical Care, Rochester, USA, ^2^Mayo Clinic, Hematology, Rochester, USA, ^3^Beth Israel Deaconess Medical Center, Anesthesia, Critical Care and Pain Medicine, Boston, USA, ^4^St Luke’s Hospital, Infectious Diseases, Duluth, USA, ^5^Mayo Clinic, Neurosurgery, Jacksonville, USA, ^6^Mayo Clinic, Infectious Diseases, Rochester, USA, ^7^Mayo Clinic, Gastroenterology, Rochester, USA

*Critical Care* 2023, **27(S1)**: P317

**Introduction:** Dysbiosis occurs from disruption to the microbiome, resulting in the expansion of pathogenic strains and a loss of bacterial diversity in the gastrointestinal tract. Within hours of critical illness, 90% of commensal organisms are lost, increasing morbidity and mortality. Probiotic supplementation failed to ameliorate dysbiosis in this population. Fermented foods have been shown to increase microbiome diversity and decrease inflammatory markers. This study investigated the safety and feasibility of administering kefir (a fermented milk beverage containing multiple cultures of commensal bacteria) as the first step to using fermented foods to mitigate dysbiosis in the critically ill.

**Methods:** We conducted an open-label phase I safety and feasibility investigation of kefir given in escalating doses to adult critically ill patients with intact gastrointestinal tracts, starting with 60 ml, then 120 ml 12 h later, followed by 240 ml daily. Gastrointestinal symptoms were monitored daily while patients received kefir in the medical intensive care unit.

**Results:** 52 patients participated (mean age 64 years (range 23–88), 62% male). No serious adverse events were observed other than diarrhea in two patients who also had documented laxative use. The median Acute Physiology and Chronic Health Evaluation II score was 35.5 (IQR 23.5–56.9). Kefir was administered via gastric tube to 28 patients, and the rest took it orally. Of the 370 doses ordered, 336 were administered (91%) and met our feasibility target (NPO status was the most common reason a dose was not given). There was no evidence of bacteremia related to kefir. Two patients discontinued study participation due to distaste and bloating.

**Conclusions:** Administration of kefir to critically ill adults appears to be safe and feasible. A larger evaluation of its safety, tolerability, and impact on dysbiosis and outcomes is warranted.

## P318

### Gut microbiome as a risk factor for mortality among sepsis patients of extreme severity

#### E Karakike^1^, G Damoraki^2^, E Stylianakis^2^, P Koufargyris^2^, I Kollias^2^, EJ Giamarellos-Bourboulis^2^

##### ^1^2nd Department of Critical Care, National and Kapodistrian University of Athens, 2nd Department of Critical Care, Athens, Greece, ^2^4th Department of Internal Medicine, National and Kapodistrian University of Athens, 4th Department of Internal Medicine, Athens, Greece

*Critical Care* 2023, **27(S1)**: P318

**Introduction:** Gut dysbiosis is frequently seen at sepsis onset, but impact on sepsis outcome remains unclear [1]. We aimed to assess microbiome diversity and composition, with regards to 28-day mortality and secondary infections in sepsis.

**Methods:** This is a secondary analysis of the INCLASS randomized trial (NCT03345992), evaluating the efficacy of clarithromycin treatment in patients with sepsis-associated respiratory distress and other organ dysfunctions [2]. Rectal microbiome was assessed for all patients on day 1, after DNA extraction and *16S* gene—based sequencing on Oxford Nanopore MinION followed by EPI2ME analysis (ONT/Metrichore Ltd). All two-tailed, Bonferroni-corrected, *p* values < 0.05 were considered significant.

**Results:** After quality assessment, 102 samples were included in the analysis. Biodiversity, assessed by Shannon and Simpson indexes, was significantly reduced among patients who died (*p* = 0.003 and *p* = 0.005), but greater among those with sepsis resolution (*v* = 0.010 and *v* = 0.018, respectively). The Shannon Index was an independent risk factor for mortality [OR 0.497; 95% Confidence intervals (CI) 0.286–0.864; *p* = 0.013], after adjustment for sequential organ failure assessment (SOFA) score, Charlson comorbidity index, and type of initial infection. Survivors displayed greater abundance in *Actinobacteria* (*p* = 0.00205) and *Bacteroidetes* (*p* = 0.01745) than non-survivors; distribution of *Proteobacteria* (*p* = 0.39299) and *Firmicutes* (*p* = 0.29353) did not differ (Fig. 1). Higher abundance in *Actinobacteria* was less likely associated with primary bacteremia during follow-up (*p* = 0.03264).

**Conclusions:** Gut dysbiosis is associated with 28-day mortality in sepsis, independently of severity scores. Higher abundance in *Actinobacteria* may be protective, possibly due to decreased bacterial translocation related to overgrowth of other phyla. Targeted interventions may improve sepsis outcome.


**References**
Adelman MW et al. Crit Care. 2020;24:278.Karakike E et al. Crit Care. 2022;26:183.

**Figure Figdx:**
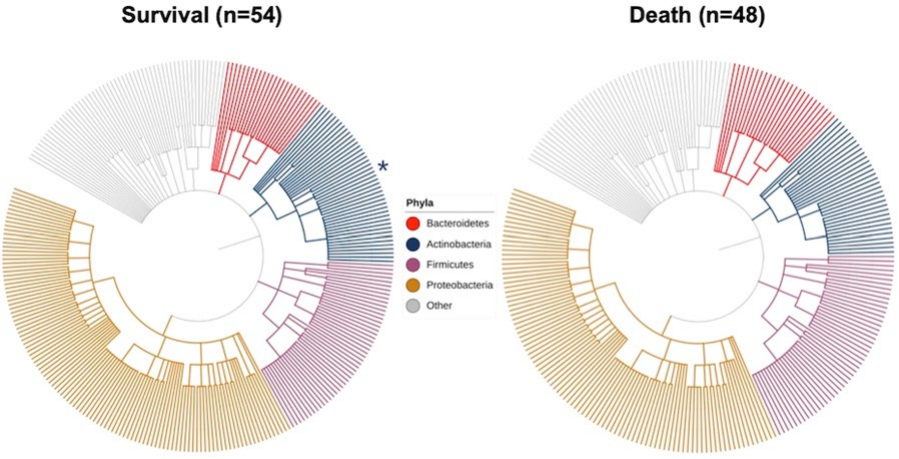
**Fig. 1 (abstract P318)**. Differences in baseline gut microbiome composition for sepsis survivors and non-survivors by day 28. *Depicts significance of *p* values after correction for comparisons in relative abundance

## P319

### The gut microbiota composition has no predictive value for the endotoxin-induced immune response or development of endotoxin tolerance in humans in vivo

#### N Bruse^1^, A Jansen^1^, J Gerretsen^1^, D Rijbroek^1^, K Wienholts^2^, M Arron^3^, H Van Goor^3^, T Ederveen^4^, P Pickkers^1^, M Kox^1^

##### ^1^Radboudumc, Department of Intensive Care Medicine, Nijmegen, Netherlands, ^2^Amsterdam UMC location University of Amsterdam, Surgery, Amsterdam, Netherlands, ^3^Radboudumc, Department of Surgery, Nijmegen, Netherlands, ^4^Radboudumc, Center for Molecular and Biomolecular Informatics (CMBI), Nijmegen, Netherlands

*Critical Care* 2023, **27(S1)**: P319

**Introduction:** Murine studies indicate that the gut microbiome may influence immunity. However, it is largely unknown whether this also applies to systemic immune responses, especially in humans. If so, profiling the gut microbiota may aid precision medicine approaches in critically ill patients.

**Methods:** The fecal microbiota composition of 108 healthy participants was determined using 16S rRNA marker gene sequencing. Immunological phenotypes were determined using (1) an ex vivo challenge model in which monocytes were stimulated with various inflammatory compounds, and (2) an in vivo challenge model of systemic inflammation and immunological tolerance induced by repeated intravenous administration of 1 ng/kg body weight *E. coli* endotoxin. This model captures hallmarks of both the hyperinflammatory and immunosuppressive phenotypes of sepsis.

**Results:** Redundancy analysis (RDA) revealed that the gut microbiota composition was associated with sex, but not with age, BMI, or diet (Fig. 1a). Significant associations were observed between the microbiota composition and ex vivo monocytic cytokine responses induced by several stimuli, although the explained variance was relatively low (0.3–4.8%, Fig. 1b). A number of pairwise correlations between *Blautia, Bacteroides* and *Prevotella* genera and cytokine production induced by these stimuli were identified (Fig. 1c). No significant relationships between microbiota composition and in vivo cytokine responses or tolerance were found (explained variance ranging from 0.4 to 1.3%, Fig. 1d,e).

**Conclusions:** Although the gut microbiota composition does explain some of the variance in ex vivo cytokine responses, its predictive value is limited. Furthermore, the gut microbiota composition is neither associated with in vivo systemic immune responses to endotoxin in humans, nor with immunological tolerance. Therefore, our findings do not support considering the gut microbiota composition for precision medicine approaches targeting the immune system in critically ill patients.

**Figure Figdy:**
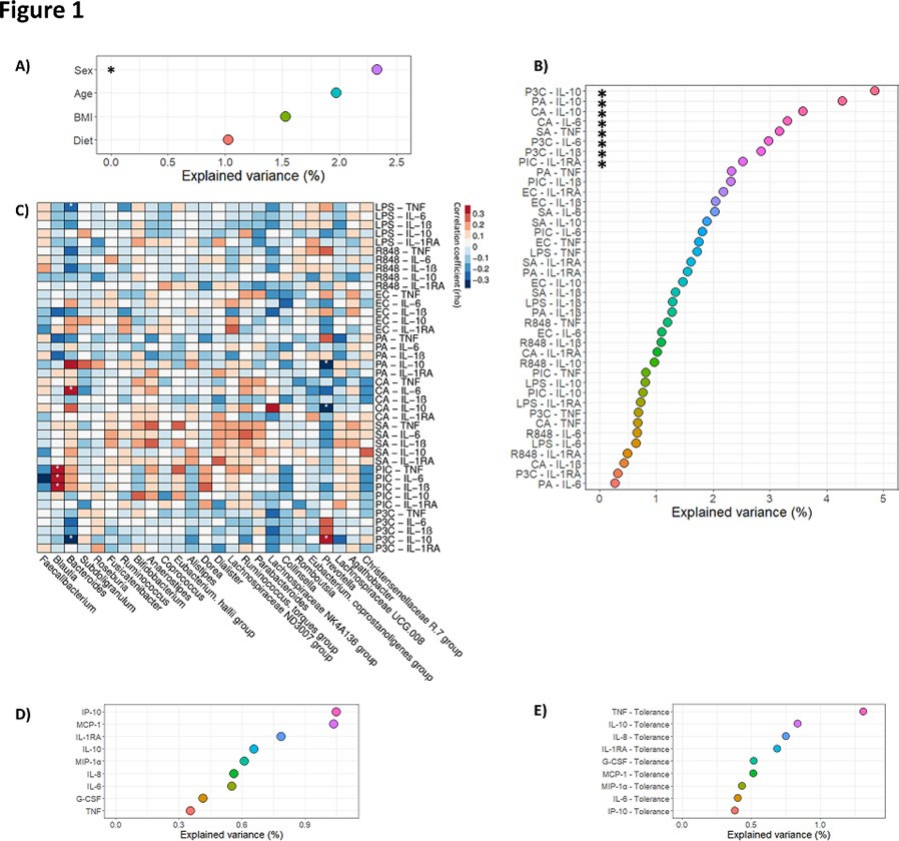
**Fig. 1 (abstract P319)**. **A** Explained variance calculated using redundancy analysis (RDA) for non-immune-related covariates. **B** Explained variance calculated using redundancy analysis (RDA) for ex vivo stimulus—cytokine pairs for all participants. **C** Pairwise correlations between the 25 most abundant microbiota on genus level and the ex vivo stimulus—cytokine pairs for all participants. **D** Explained variance calculated using redundancy analysis (RDA) for in vivo cytokine responses upon the first LPS challenge (day 0)

## P320

### Association between gastrointestinal tract function and mortality in critically ill patients with suspected sepsis

#### M Roimi^1^, A Shrot^2^, R Ilan^1^, D Epstein^1^, Y Bar-Lavie^1^

##### ^1^Rambam Health-Care Campus, Critical Care Division, Haifa,, Israel, ^2^Rambam Health-Care Campus, Independent researcher, Haifa, Israel

*Critical Care* 2023, **27(S1)**: P320

**Introduction:** Evaluating a patient suspected of having sepsis mandates an assessment of organ dysfunction. Current severity scores do not assess gastrointestinal (GI) tract function. We aimed to determine the association between GI tract function on the days preceding presumed infection with the clinical outcome.

**Methods:** A retrospective cohort study including all the adult patients admitted to the 18 beds general ICU in an academic tertiary medical center between January 2013 and December 2020. We included patients with suspected infection after at least 72-h from ICU admission. Patients with a colostomy, ileostomy, or a rectal tube were excluded. The main exposures were the number of bowel movements (BM) and consecutive days without BMs during the preceding 72-h. The primary outcome was 30-day all-cause mortality. Multivariate logistic regression was performed to assess for possible confounders including factors associate with GI tract motility, antibiotics, length of stay, APACHE II, and SOFA scores.

**Results:** The cohort included 1306 patients. The median age was 56.2 years (IQR 39.469.1); 1004 (70.0%) were male. At the time of suspected infection, 1139 (79.4%) were treated with antibiotics, and the median SOFA score was 5.0 (IQR 3.0–7.0). The 30-day all-cause mortality rate was 364 (25.4%). Patients who had 0–2, 3–5, and > 5 BMs during the 72-h preceding the suspected infection had mortality rates of 20.3%, 28.0%, and 34.3% (*p* < 0.001) (Fig. 1). Patients who had > 2 BMs had an adjusted OR of 1.55 (95% CI 1.13–2.1, *p* = 0.005) for mortality. Every additional consecutive day without BM before the suspected infectious event was associated with an adjusted OR of 0.84 (95% CI 0.73–0.95, *p* < 0.02) for mortality.

**Conclusions:** In patients suspected to have sepsis, a higher frequency of BMs during the preceding 72-h, is associated with increased mortality. Further studies are needed to confirm our findings and investigate the physiologic mechanisms related to the GI tract function during sepsis.

**Figure Figdz:**
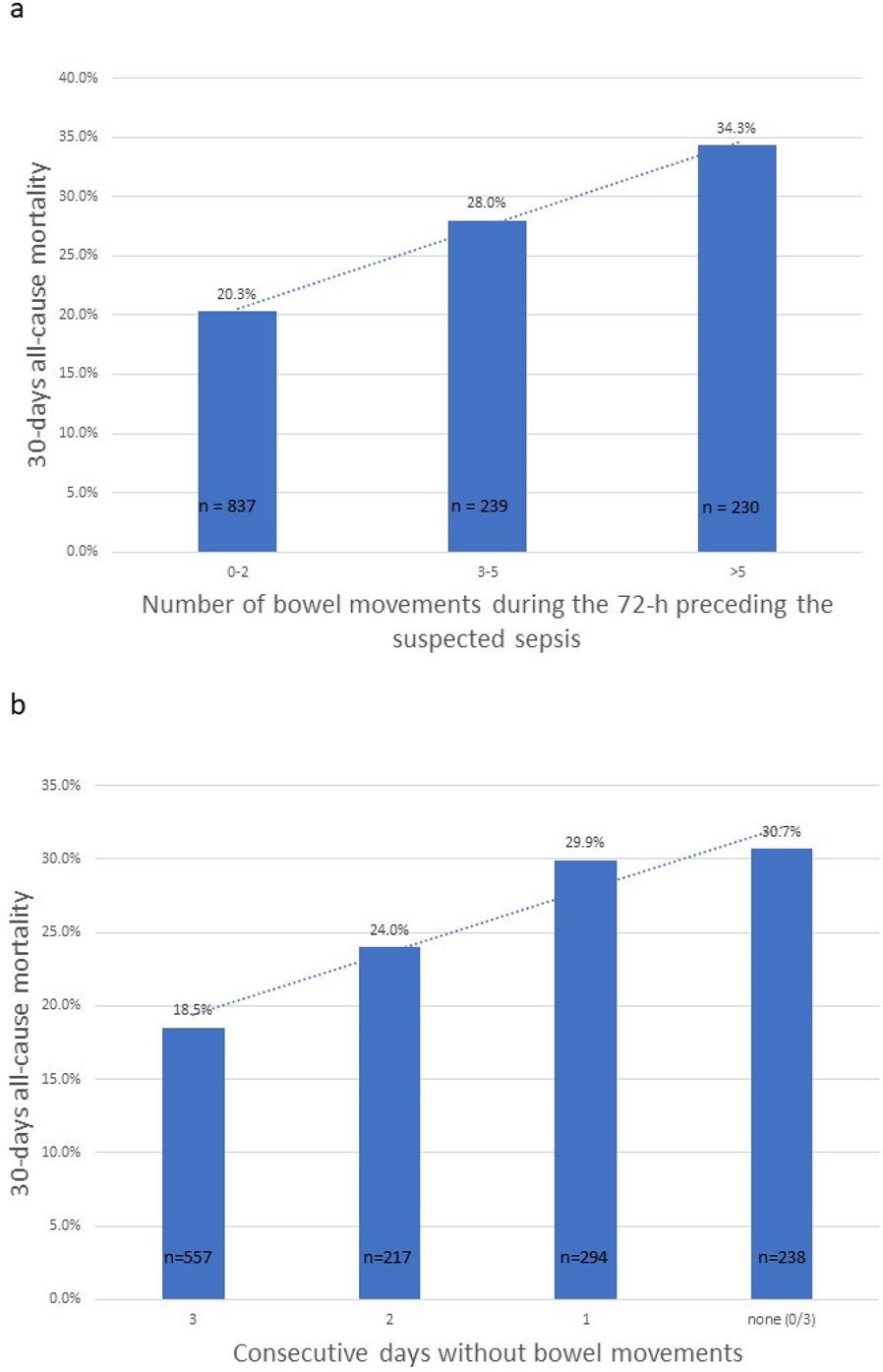
**Fig. 1 (abstract P320)**. The association between gastrointestinal tract function during the 72-h preceding suspected infection and 30-day all-cause mortality

## P321

### The accuracy of pH strip test and pH liquid test comparing with the standard values from pH meter in measuring gastric pH from medical critically ill patients

#### K Phukpattanachai^1^, N Praditseree^1^, S Skjolaas^1^, S Klaychaiya^1^, K Trongtrakul^2^

##### ^1^Faculty of Medicine Chiang Mai University, Chiang Mai, Thailand, ^2^Faculty of Medicine Chiang Mai University, Department of Internal Medicine, Chiang Mai, Thailand

*Critical Care* 2023, **27(S1)**: P321

**Introduction:** Gastric pH measurement can be utilized to confirm the position of nasogastric (NG) tube insertion. Thus, our study aimed to determine the accuracy of more convenient methods by the pH strip test (pHS) and pH liquid test (pHL), compared to the standard pH meter (pHM) in measuring gastric pH values in critically ill patients.

**Methods:** A prospective observational study was conducted on patients who were admitted to medical ICUs of the Faculty of Medicine, Chiang Mai University (MedCMU), Chiang Mai, Thailand, between June and July 2022. Gastric samples were measured by pHM, pHS, and pHL techniques and interpreted by four independent raters. The reliability of pHS and pHL readings reported by intraclass correlation coefficients (ICC) were 0.93 (0.90, 0.96) and 0.94 (0.90, 0.96), respectively.

**Results:** A total of 113 gastric samples were collected from 27 ICU patients, which were 71 (IQR 56, 81) year-old, male 48% (13/27 cases), sepsis/septic shock 67% (18/27 cases), SOFA score 5 (IQR 2.9), and serum lactate 3.7 (IQR 2.2, 5.6) mmol/l. The pH values measured from pHM, pHS, and pHL were 5.83 (IQR 5.12, 6.61), 5.50 (IQR 4.88, 6.00), and 5.75 (IQR 5.25, 6.38), respectively, *p* < 0.01. The pH value from the pHS method was slightly lower than the pHM, with a difference of −0.25 (−0.54, 0.03), *p* < 0.001. While the pH value from pHL was slightly higher than the pHM, with a difference of 0.02 (−0.36, 0.53), *p* = 0.04. The accuracy of pH measurement from pHS had a good correlation with pHM, with Spearman’s correlation (rho) of 0.91 (0.86 to 0.96) and the linear regression equation Y = 0.95 X + 0.56 (Fig. 1A (abstract P321)). On the other hand, there was an optimal correlation between pHL and pHM, with rho = 0.75 (0.60 –0.89) and the equation Y = 1.09 X −0.72 (Fig. 1B).

**Conclusions:** The use of the pHS has a better correlation with the standard pHM measurement than the pHL. The utilized pHS in measuring gastric pH might be an option to ensure the tip of the NG tube position in critically ill patients.

**Figure Figea:**
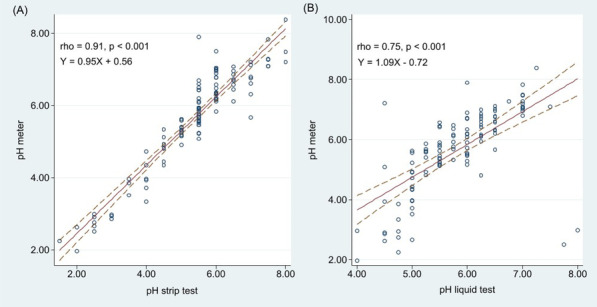
**Fig. 1 (abstract P321)**. The Spearman’s correlation and linear regression equation of (**A**) pH strip test vs. pH meter and (**B**) pH liquid test versus pH meter

## P322

### Observational study: the risk of malnutrition in post-ICU COVID-19 patients in Dubai, UAE

#### W Ayesh^1^, F Dhinda^2^, S Noufal^2^, N Murad^2^

##### ^1^Al Tadawi General Hospital and Medical Center, Dubai, United Arab Emirates, ^2^Dubai Health Authority (DHA), Clinical Nutrition Department, Dubai, United Arab Emirates

*Critical Care* 2023, **27(S1)**: P322

**Introduction:** Due to the impact of the severe acute respiratory syndrome coronavirus 2 (SARS-CoV-2) on the humoral immune system, gastrointestinal, and metabolic activities, malnutrition in COVID-19 is inevitable. This study aimed to assess the prevalence, identify COVID-19 patients at risk of malnutrition, and determine the nutritional risk profile of COVID-19 patients and the need for ongoing nutritional support after ICU stay.

**Methods:** A monocentric observational study based on data collected from 200 COVID-19 patients at hospital discharge in Dubai, UAE. Male and female residents and citizens (≥ 18 years) who tested positive for COVID-19 upon ICU admission and who were ready for discharge were included. The ‘MUST’ malnutrition screening was performed to identify patients at high risk of malnutrition who required ONS and other treatments.

**Results:** The present study included two hundred patients where male participants constituted 68% compared to females (32%). The included population was neither acutely ill nor had nutritional intake for more than 5 days. 45% of COVID-19 patients experienced a reduced dietary intake at hospital, and 58% lost weight during ICU/hospital stays. About 25% received enteral nutrition in the ICU, whereas (2%) required ongoing homecare nutritional support after hospital discharge. Almost 80% were advised to follow up with a dietitian and 96% were provided additional dietary counseling. Regarding the COVID-19 patients' post-ICU stay nutritional support, the adjusted odds ratio of follow-up consultation with dietitian significantly decreased by 66% among patients aged from 18 to 49 years, compared to older patients (ORa = 0.34, 95% CI 0.12–0.86, *p* = 0.032).

**Conclusions:** Close assessment, evaluation, and monitoring of malnutrition are critical in severely ill COVID-19 patients post-ICU. ONS is highly recommended for high-risk patients to provide support against muscle loss during ICU stay and improve the recovery of the patients at discharge.

## P323

### Ultrasound assessment of muscle mass loss in the burned patient and its association with prognosis

#### RI Prado^1^, MT Tanita^2^, LTQ Cardoso^3^, CMC Grion^3^

##### ^1^University Hospital – Londrina State University, Critical Care, Londrina, Brazil, ^2^University Hospital – Londrina State University, Intensivist physician, Londrina, Brazil, ^3^University Hospital – Londrina State University, Department of Internal Medicine, Londrina State University, Londrina, Brazil

*Critical Care* 2023, **27(S1)**: P323

**Introduction:** Loss of muscle mass may be a predictor of mortality in major burn patients, since patients who develop skeletal muscle tissue degradation have longer hospital stays, prolonged mechanical ventilation. The mortality rate in patients with severe burns is between 1.4 and 18% [1]. The aim of this study was to evaluate the loss of lean mass in burn patients through ultrasound of the quadriceps muscle of the thigh in burn patients admitted to a specialized bed at a university hospital and to correlate the results with the prognosis.

**Methods:** Prospective longitudinal study performed using ultrasound of the quadriceps muscle of the thigh to assess the change in thickness in millimeters on day 1, day 3, and day 7 after study enrollment, in 45 burn patients admitted to a burn treatment center (BTC) of a University Hospital, between April 2020 and September 2021. Patients with burns on the thighs, which prevented the performance of the exams, were excluded.

**Results:** Correlation was observed between the variation in muscle thickness in the interval from Day 1 to Day 3 and age (*p* = 0.035). Loss of muscle thickness was observed in all patients between Day 1 and Day 7. The median thickness for all patients on Day 1 was 24.50 mm (ITQ 21.22–30.85) and on Day 7 it was 18.80 (ITQ 16.07–23.62), with *p* = 0.0001. The median quadriceps muscle thickness of the patients admitted to the ward on Day 1 was 27.35 mm (ITQ 21.00–35.00) and on Day 7 it was 22.35 mm (ITQ 15.70–27.90), with *p* = 0.038. The median thickness of patients admitted to the ICU on Day 1 was 23.40 mm (ITQ 21.30–27.00) and on Day 7 it was 18.00 mm (ITQ 16.67–22.02), with *p* = 0.0005.

**Conclusions:** Muscle loss occurred early and rapidly within the first seven days of hospitalization. An association was observed between loss of muscle thickness and age, but no association was observed with the extent of the burn, hospital stay, occurrence of healthcare-associated infection, or mortality.


**Reference**
Brusselaers N et al. Crit Care 2010;14:R188


## P324

### The role of sarcopenia in the mortality of critically ill patients in the ICU

#### F Moniati^1^, C Chatzimatthaiou^2^

##### ^1^Barts and The London, School of Medicine, London, UK, ^2^Barts and The London, London, UK

*Critical Care* 2023, **27(S1)**: P324

**Introduction:** First proposed by Dr. Rosenberg, sarcopenia, the loss of skeletal muscle mass, involves a decline in clinical performance, increased mortality and morbidity. In the critically ill patients in ICU, mortality is still a challenge, particularly for elderly patients with functional decline. The relationship between mortality in ICU critically ill patients and sarcopenia is inconsistent throughout literature, impeding our ability to quantify the magnitude of the problem. Our primary objective is to evaluate the role of sarcopenia in the mortality of the critically ill patients in ICU through a systematic review.

**Methods:** This systematic review was conducted using the Cochrane, Embase and PubMed databases to examine the studies available for the association between sarcopenia and mortality in the critically ill patients.

**Results:** Overall, eleven retrospective cohort studies totalling 2029 patients, all aged 65 years of age and older, were included in our review with the primary outcome of all studies being mortality. As far as the methods of skeletal muscle measurement using Computerized Tomography (CT) are concerned, six studies used skeletal muscle index (SMI) (n = 832), three studies used skeletal muscle area (SMA) (n = 711) and two studies used total psoas muscle area (TPA) (n = 486). The use of SMI demonstrated an increased risk of mortality in critically ill patients with sarcopenia with 95% confidence interval (CI) = 1.75–2.82. Similar results were also shown using SMA (CI = 2.73–4.47) or TPA (CI = 1.85–6.30).

**Conclusions:** Evidently, these results illustrate that critically ill patients in ICU with sarcopenia hold a greater risk of mortality compared with patients without the condition. Effective intervention programmes such as early mobilization and high protein diet can help reduce the mortality risk and provide beneficial outcomes.

## P325

### The association of sarcopenia defined by a simpler screening tool and long-term outcomes

#### T Yuyen, C Thanakiattiwibun, O Chaiwat

##### Department of Anesthesiology, Faculty of Medicine, Siriraj Hospital, Mahidol University, Department of Anesthesiology, Bangkok, Thailand

*Critical Care* 2023, **27(S1)**: P325

**Introduction:** Sarcopenia and frailty are common in older adult patients and associated with poor postoperative outcomes. Low muscle mass and function are main indicators of sarcopenia. The simpler screening tool without muscle mass measurement, C3 formula, demonstrated high predictive power for diagnosing sarcopenia [1]. The aim of this study was to identify the association of sarcopenia defined by C3 formula and long-term outcomes in older adult cancer patients undergoing surgery.

**Methods:** Cancerous surgical patients aged older than 60 years were enrolled. The sarcopenia was defined by C3 formula, consisted of low muscle strength and/or abnormal physical performance, plus malnutrition/risk of malnutrition. The muscle strength was measured by handgrip-strength. The physical performance was assessed by the 6-m usual gait speed walk test. Nutritional status was evaluated by the Mini Nutritional Assessment–Short Form (MNA-SF). After sarcopenia screening, the long-term outcomes were collected including the Barthel Index of Activities of Daily Living (ADL) scores, any infections in the hospital, length of hospital stay, and mortality at 3 months and 1 year.

**Results:** 251 patients were enrolled. 130 patients were sarcopenic defined by C3 formula. Sarcopenic patients had greater number of moderate to severe disability (ADL score ≤ 70) at 3 months after hospital discharge (19.5% vs 5.2%, *P* = 0.001) and higher mortality rate at 1 year after sarcopenia screening (29.5% vs 14.9%, *P* = 0.006). There were no differences regarding the infection rate, length of hospital stay and in-hospital mortality rate. The independent risk factors for one year mortality were distant organ metastasis (HR: 3.99, 95% CI 2.25–7.07) and sarcopenia defined by C3 formula (HR: 1.78, 95% CI 1.01–3.15).

**Conclusions:** Sarcopenia defined by a simpler screening tool was associated with higher moderate to severe disability at 3 months after discharge and increased mortality rate at 1 year after screening.


**Reference**
Chaiwat O et al. PLoS One. 2021;16:e0257672


## P326

### Effectiveness and safety of early enteral feeding in critically ill patients with sepsis: a systematic review and meta-analysis

#### RE Ko^1^, SJ Moon^2^, CM Park^1^, GY Suh^1^, CR Chung^1^

##### ^1^Samsung Medical Center, Critical Care Medicine, Seoul, South Korea, ^2^Samsung Medical Center, Medicine, Seoul, South Korea

*Critical Care* 2023, **27(S1)**: P326

**Introduction:** Although early enteral nutrition (EN) may prevent muscle catabolism, it is concerning in sepsis because of increased risks of intestinal ischemia. We aimed to redefine the evidence regarding the effectiveness and timing of early EN using a systematic review that focused on critically ill patients with sepsis.

**Methods:** We searched MEDLINE, Embase, Cumulative Index to Nursing and Allied Health Literature, Cochrane Central Register of Controlled Trials, ClinicalTrials.gov, and ICTRP databases on February 23, 2022. Our systematic review included studies published after 2004 that compared outcomes of early EN with those of delayed EN in critically ill patients with sepsis aged ≥ 18 years. Two authors independently reviewed the title, abstract, and full text and selected relevant studies according to the inclusion criteria. Two authors independently extracted patient information and reports on effectiveness, adverse effects, and clinical outcomes.

**Results:** Ten studies were included, and the sample size was 31–2410. The pooled risk ratio of mortality obtained using three randomized controlled trials was 1.02 (95% confidence interval [CI] 0.85–1.24; I^2^ = 3.15%, *p* = 0.26), which was not significant. The pooled mean difference of length of stay in the intensive care unit obtained using two randomized controlled trials was -1.52 days (95% CI − 3.18 to 0.15; I^2^ = 47.62%, *p* = 0.17), which was not significant. The pooled risk ratio of intestinal ischemia obtained using two randomized controlled trials was 3.53 (95% CI 1.36–9.15; I^2^ = 0.00%, *p* = 0.53); this was significantly predominant for early EN. Other outcome measurement indices and adverse effect reports had mixed results.

**Conclusions:** Early EN intervention did not significantly improve intensive care unit-related mortality but increased intestinal ischemia risks in critically ill patients with sepsis. Heterogeneous characteristics and outcome measurements should be improved for a tailored approach to early EN in critically ill patients with sepsis.

## P327

### Early initiation of fluid intake (sipping) after extubation versus current recommendation, effect of feeling of thirst

#### V Nigrovičová, Š Sedláčková, M Durila

##### University Hospital in Motol, Department of Anaesthesiology, Resuscitation and Intensive Care medicine, 2nd Medical Faculty, Charles University, Prague 5, Czech Republic

*Critical Care* 2023, **27(S1)**: P327

**Introduction:** Safe extubation is only possible if the patient is conscious and cooperative, able to cough, swallow and has preserved protective reflexes of the respiratory and swallowing tract. Despite that, according to the current recommendation, after extubation they do not receive any liquid orally for 2 h and therefore they feel thirsty. However, there is a lack of evidence in the literature about the validity of this recommendation. The aim of study was to investigate the incidence of thirst and occurrence of complications between patients receiving sipping immediately after extubation and patients who did not drink any fluid within 2 h after extubation.

**Methods:** In randomized study we assigned 40 patients after short intubation and who were extubated within 48 h. We defined 2 groups with 20 patients in every group. 1st group—early initiation of oral fluid intake in a form of sipping within 2 h, with maximal amount of water 100 ml per hour. 2nd group—oral fluid intake postponed up to 2 h after extubation. Exclusion criteria: patients after upper digestive tract surgery, trachea surgery and with possible neurological deficiency. At defined time points (0, 60 and 120 min after extubation) we evaluated thirst and occurrence of complications (nausea, vomiting, aspiration) between both groups.

**Results:** Despite the fact we did not find a significant difference in the feeling of thirst at time points—*p* > 0.05 (probably because of low number of patients, but tendency is obvious), we found, that in the 1st group where patients received sipping, we saw a significant thirst relief in every time point—0, 60, 120 min after extubation—*p* = 0.0009, 0.0088 and 0.0003. There were no differences in occurrence of complications between both groups.

**Conclusions:** The study shows that early initiation of fluid intake after extubation, in a form of sipping, seems to be beneficial and safe for patients and decreases incidence of thirst after extubation without any complications.

## P328

### Amino acids effect on muscle and gut functionality in critically ill patients: a proof-of-concept randomized controlled trial

#### R Bounab^1^, N Heming^1^, R Carlier^2^, H Prigent^3^, V Maxime^1^, B Guenoud^4^, M Hartweg^4^, C Roessle^4^, D Breuille^4^, D Annane^1^

##### ^1^Raymond Poincaré hospital, critical care unit, Garches, France, ^2^ Raymond Poincaré hospital, Medical Imaging, Garches, France, ^3^Raymond Poincaré hospital, Department of Physiology, Garches, France, ^4^Société de Produits Nestlé, Nestlé Health Science, Lausanne, Switzerland

*Critical Care* 2023, **27(S1)**: P328

**Introduction:** A fraction of critically ill patients require prolonged intensive care unit (ICU) stays [1]. This population is at greater risk of muscle mass and muscle strength loss. Supplementation using a tailored mix of amino acids may strengthen the innate gut defence, promote intestinal mucosa repair and limit muscle loss [2].

**Methods:** We performed a single-centre, double-blind randomized placebo-controlled study testing a specific combination of five amino acids or placebo mixed with enteral feeding for up to 21 days. Markers of renal function, gut barrier structure and muscle functionality were collected at baseline and 1, 2, 3 and 8 weeks after randomization. Muscle structure and function were assessed by means of MRI measurements of the anterior quadriceps volume and by twitch airway pressure. Data were compared between groups relative to the baseline.

**Results:** We randomized 35 critically ill patients with sepsis or ARDS. The amino acid blend did not impair urine output, blood creatinine levels or creatinine clearance. Plasma citrulline levels increased significantly during the treatment period in the amino acid group (difference in means [95% CI] 5.86 [1.72; 10.00] nmol/ml, *p* = 0.007). Alanine aminotransferase and alkaline phosphatase concentrations were lower in the amino acid group than in the placebo group at one week (ratio of means 0.5 [0.29; 0.86] (*p* = 0.015) and 0.73 [0.57; 0.94] (*p* = 0.015), respectively). Twitch airway pressure and volume of the anterior quadriceps were greater in the amino acid group than in the placebo group 3 weeks after randomization (difference in means 10.6 [0.99; 20.20] cmH20 (*p* = 0.035) and 3.12 [0.5; 5.73] cm^3^/kg (*p* = 0.022), respectively) (Fig. 1).

**Conclusions:** The5-amino acid blend supplementation increased plasma citrulline levels, reduced alanine aminotransferase and alkaline phosphatase levels, and improved twitch airway pressure and anterior quadriceps volume.


**References**
Nelson JE et al. Am J Respir Crit Care Med 2010;182:446–54.Plank LD et al. Ann Surg 1998;228:146–58.


**Figure Figeb:**
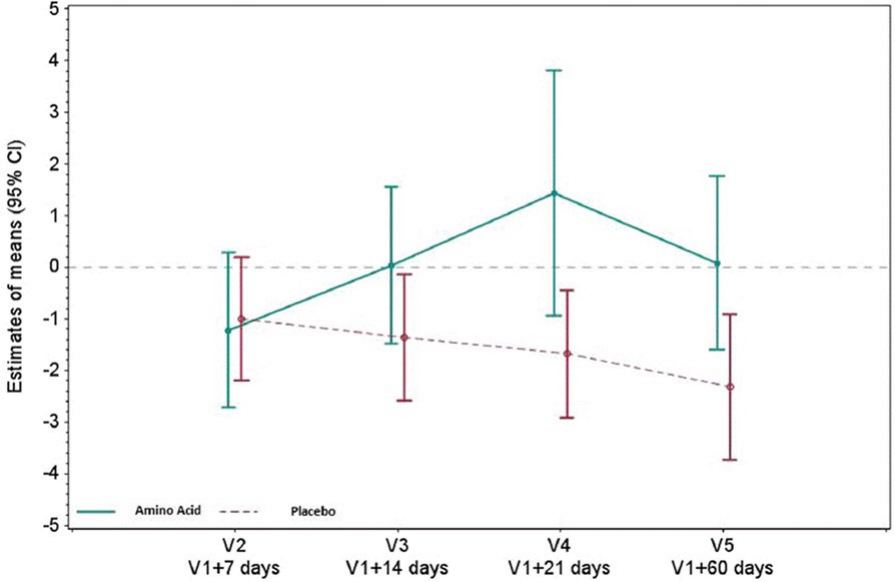
**Fig. 1 (abstract P328)**. Indexed muscle volume of the anterior compartment of the quadriceps (cc/kg) in the amino acid and placebo groups. Anterior quadriceps volume was measured semi-automatically using isometric T1-weighted 3D images

## P329

### The impact of proning on nutritional therapy in mechanically ventilated COVID-19 patients

#### N Steinfort, K Dams, R Jacobs, P Jorens

##### Antwerp University Hospital, ICU, Edegem, Belgium

*Critical Care* 2023, **27(S1)**: P329

**Introduction:** Critically ill COVID-19-patients are at high risk of developing ICU-related malnutrition. This study aimed to examine the impact of proning on providing nutritional therapy for mechanically ventilated COVID-19-patients by comparing the achievement of nutritional goals and possible complications in patients who were proned vs. those who were not.

**Methods:** This is a single-center retrospective cohort study. We included all adult COVID-19 patients admitted to the ICU from 01/03/2020 until 31/05/2020 who required invasive mechanical ventilation (IMV), excluding those referred for ECMO. Data were extracted from electronic patient files. Weight-based nutrition targets were set in agreement with ESPEN guidelines [1].

**Results:** 32 patients were included (prone n = 16). Both groups were comparable in age, sex, comorbidities, biochemical markers and Nutrition Risk Screening on admission. Time on IMV was longer in the prone group (*p* = 0.032). The total time in prone position ranged from 19.5 h to 13.16 d. All patients received a NG tube, 1 proned patient received a jejunal tube. 6 received TPN (*p* = 0.654). Metoclopramide was used more often in the prone group (*p* = 0.028). The prevalence of vomiting (n = 4 vs. n = 5), large gastric residuals (n = 0 vs. n = 3) and VAP (n = 11 vs. n = 10) were comparable for the non-prone vs. prone group, resp. Table 1 shows the percentage of targets reached. These were lower in the prone group, though not statistically significant. However, when correcting for SAPS III-score, the impact of proning declined.

**Conclusions:** These limited data suggest there is no significant difference in feeding COVID-19 patients on IMV that need proning vs. those who do not, except for metoclopramide use. Overall, reaching nutrition targets in these patients is challenging. This model suggests that disease impact is a greater influence on reaching nutritional goals than proning itself.


**Reference**
Singer P et al. Clin Nutr 2019;38:48–79.

**Table Tabdv:** **Table 1 (abstract P329)**. Percentage of targets reached

Achieving targets	Non-prone	Prone	*p* value
Kcal at 24 h (% of target reached)(median, min;max)	11.74 (0.00; 65.70)	11.15 (0.00; 92.17)	0.669
Prot at 24 h (% of target reached)(median, min:max)	0.00 (0.00; 80.00)	0.00 (0.00; 50.00)	0.254
Kcal at 48 h (% of target reached)(median, min;max)	33.68 (0.38; 122.6)	22.83 (0.00; 127.27)	0.557
Prot at 48 h (% of target reached)(median, min;max)	33.95 (0.00; 161.11)	19.27 (0.00; 168.35)	0.305
Kcal at d7 (% of target reached)(median, min;max)	71.12 (32.59; 94.42)	57.14 (10.95; 83.25)	0.136
Prot at d7 (% of target reached)(median, min;max)	77.98 (23.34; 138.22	58.05 (14.78; 144.66)	0.273

*Kcal* kilocalories, *Prot* protein, *min* minimum value, *max* maximum value

## P330

### Comparative evaluation of different respiratory rehabilitation methods in the early postoperative period of cardiac surgery patients

#### DV Fomina, AA Eremenko, TP Zyulyaeva, MS Grekova, AP Alferova, OO Grin, SS Dmitrieva

##### Petrovsky National Research Centre of Surgery, intensive care unit, Moscow, Russian Federation

*Critical Care* 2023, **27(S1)**: P330

**Introduction:** The frequency of postoperative respiratory failure due to ineffective coughing and evacuation of bronchial mucus is 17%-22%. The objective was to evaluate the effectiveness of different cough stimulation methods in the early postoperative period of cardiac surgery patients.

**Methods:** In randomized prospective comparative study (ClinicalTrials.gov—NCT05159401) four methods were evaluated. There were 20 patients in each group. 1st group—oscillatory PEP-therapy (Acapella DH Green, Portex), group 2—external chest oscillatory massage combined with intermittent pressure mask ventilation Comfort Cough Plus, Seoil Pacific Corporation, (CC), group 3—vibro-acoustic lung massage, BARK VibroLUNG, (VL). The 4th control group included patients with manual chest massage (MCM). Procedures were performed 10–12 h after tracheal extubation 3 times a day for 3 days. Before the procedure and 20 min after it, the effectiveness of sputum discharge, gas exchange and maximum inspiratory lung capacity (MILC) were evaluated.

**Results:** The largest number of patients with productive cough was fixed in groups 1, 2 and 4 after fourth session, in group VL during 4–6 session. The increase in SpO_2_ on room air after 3 days of treatment with PEP-therapy and MCM was 4%, in others—5% (Table 1). The maximum increase in MILC was registered in the patients with PEP-therapy, the minimum—in the MCM group. Mean MILC exceeded more than 2000 ml in group 1 by session 7, in group 2 by session 4, in group 3 by session 9, in group 4 by session 8. No significant effect on PaCO_2_ was noted.

**Conclusions:** The effects of the applied methods of mechanical stimulation of cough were directed to improve the passage of mucus and oxygenating lung function. After 3 days of treatment the most significant increase of MILC was registered in PEP-therapy and CC group. No undesirable effects of the procedures were noted.

**Table Tabdl:** **Table 1 (abstract P330)**. Results

Parameter/p	PEP-therapy	CC	VL	MCM
∆SpO_2,_ %	4 ± 3	5 ± 4	5 ± 3	4 ± 2
MILC, 1st session,ml	1000 [650;1500]	1000 [800;1625]	1210 ± 777	1233 ± 771
MILC, 9th session,ml p1–3 = 0.02; p2–3 = 0.04; p3–4 = 0,	2460 ± 1174	2233 ± 900	2155 ± 838	2388 ± 1118
∆ MILC, ml p1–3 = 0.03; p1–4 = 0.03	1255 ± 998	1140 ± 966	950 [450;1100]	925 [300;1500]

*CC* Comfort Cough, *VL* VibroLUNG, *MCM* manual chest massage, *MILC* maximum inspiratory lung capacity, M ± SD, Me[25;75]

## P331

### The effect of physical rehabilitation on ICU adults

#### C Chatzimatthaiou^1^, F Moniati^2^

##### ^1^Barts and The London, School of Medicine, London, UK, ^2^Barts and The London, London, UK

*Critical Care* 2023, **27(S1)**: P331

**Introduction:** A large percentage of critical illness survivors suffer due to loss of muscle mass, polyneuropathy and myopathy which occur during the early phase of ICU admission and are correlated with increased ICU and hospital stays. In order to manage this physical sequela of critical illness, a specific approach has been adopted: physical rehabilitation. In the ICU, rehabilitation is used to reverse muscle catabolism, reduce the effects of immobility and improve physical functioning. Our main outcome is to investigate the effect of physical rehabilitation in ICU specifically on the outcomes of patients, the dose–response profile and the impact it has on task-specific training.

**Methods:** We conducted a systematic review using the Ovid Medline, Cochrane and Embase databases to explore the current studies available on physical rehabilitation in the ICU. We included all randomized controlled trials which investigated physical rehabilitation in the ICU in adults. Physical function, muscle strength, hospital and ICU stays, health-related quality of life and mortality comprised our outcomes.

**Results:** We included sixty-five trials, with a total of 5658 participants. Results showed that physical rehabilitation decreased hospital length stay by 1.77 days (mean difference, 1.77 d: 95% CI 3.05–0.48 d), ICU length of stay by 0.7 days (mean difference, 0.70 d: 95% CI 1.39 TO 0.25 d) and physical function at the hospital discharge has been improved (mean difference, 0.24; 95% CI0.00–0.46). In trials which investigated functional exercises and low-dose physical rehabilitation, the intervention was more effectual compared to the control. Lastly, there was no impact on the other outcomes caused by physical rehabilitation.

**Conclusions:** We concluded that physical rehabilitation in the ICU overall decreased the hospital and ICU length of stay and improved physical function.

## P332

### Impact of early rehabilitation on the functional quality of life of the critically ill at one year

#### ARA Rodríguez Alcaraz^1^, BLV Lobo Valbuena^1^, LMC Manglano-Cerrato^2^, AA Alcina^2^, RML Molina Lobo^1^, PB Benavides^2^, JJ Juárez^2^, RCV Castañeda Vozmediano^3^, FG Gordo^1^

##### ^1^Hospital del Henares, Servicio de Medicina Intensiva, Coslada, Madrid, Spain, ^2^Hospital del Henares, Servicio de Medicina Física y Rehabilitación, Coslada, Madrid, Spain, ^3^Facultad de Ciencias de la Salud, Universidad Francisco de Vitoria, Grupo de Investigación en Patología Crítica, Pozuelo de Alarcón, Madrid, Spain

*Critical Care* 2023, **27(S1)**: P332

**Introduction:** ICU-acquired weakness (ICUAW) is a long-recognised phenomenon, featuring a prevalence of 25–80%. Early mobilisation is anaccepted intervention that may attenuate ICUAW and improve outcomes [1, 2].

**Methods:** Prospective observational study in polyvalent ICU analysing the effect of early rehabilitation (eRHB) on quality of life one year after discharge (D/C).Patients who required invasive mechanical ventilation > 24 h and survived SARS-CoV2 respiratory infection between 5/3/2020 and 12/01/2022 were included. Patients were classified into two groups: eRHB or not eRHB. Demographic and clinical data were collected, and a telephone survey was conducted one year after D/C. Clinical Frailty Scale at ICU admission (T1) and one year after D/C (T5); Medical Research Council (MRC) at the start of rehabilitation (T2) and hospital D/C (T4); Barthel Index at ICU D/C (T3), T4 and T5; and the SF-36 health questionnaire at T5 were also collected. Statistical analysis was performed between subgroups: Pearson's Chi-square test or Mann–Whitney *U* test to find significant differences. ART-ANOVA was used to analyse the survey results.

**Results:** Of 99 patients, 64.6% belonged to the eRHB group. There were no statistically significant differences in the analysis of clinic-demographic variables. We observed a significant improvement of the MRC, a better Barthel Index in the eRHB group, and a statistically significant positive impact on several components of the SF-36 in the eRHB group (physical functioning, vitality, social functioning, bodily pain, general health, and self-reported health transition).

**Conclusions:** Patients who received eRHB had better physical functioning and higher vitality recovery. In addition, they suffered less impact on their social life, had better pain control, and reported improved general health. All this emphasises the need for eRHB protocols in the ICU, promoting multidisciplinary care of our patients.


**References**
Needham DM. JAMA 2008;300:1685–1690.Lang JK et al. Crit Care Med. 2020;48:e1121–e1128.


## P333

### Effect of early mobilization within 72 h of admission or intubation of critically ill ICU patients on length of ICU stay: a systematic review with meta-analysis

#### N Daum^1^, N Drewniok^1^, A Buyukli^1^, B Ulm^2^, J Grunow^1^, S Schaller^1^

##### ^1^Charité – Universitätsmedizin Berlin, Department of Anesthesiology and Operative Intensive Care Medicine (CVK, CCM), Berlin, Germany, ^2^Technical University of Munich, Munich, Germany

*Critical Care* 2023, **27(S1)**: P333

**Introduction:** Previous systematic reviews showed a positive effect of early mobilization on ICU length of stay (LOS). There is data showing that an early start as recommended, i.e., within 72 h after ICU admission or intubation in critically ill patients, is essential for positive effects on ICU LOS [1, 2]. Therefore, we aimed to investigate the effectiveness of early mobilization within 72 h of ICU admission or intubation.

**Methods:** We conducted a systematic review and meta-analysis examining the effect of early mobilization within 72 h compared with standard care in terms of ICU length of stay. Here, randomized controlled trials were included from inception to October 8, 2022. Early mobilization was defined, based on Bein et al., as any patient intervention that initiates and/or supports passive or active range of motion exercises and aims to promote and/or maintain range of motion [1].

**Results:** Of a total of 29,680 studies screened, 18 studies with a total of 1833 patients met the inclusion criteria and were included in the statistical analysis. In the meta-analysis, the random effects model showed a positive effect in ICU LOS in the group after admission in favor of early mobilization compared with standard care (WMD (weighted mean difference): −0.57 days, 95% CI [−1.26, 0.13]; SMD (standardized mean difference): −0.44, 95% CI [−0.82; −0.06]; 16 studies with 1567 patients; see Fig. 1). The effect after intubation proved to be nonsignificant (WMD: −0.65 days, 95% CI [−1.92, 0.63]; SMD: −0.37, 95% CI [−1.11; 0.38]; 2 studies with 266 patients). When all studies were considered together, there was a positive effect in favor of early mobilization compared with standard care with a significant SMD (WMD: −0.58 days, 95% CI [−1.19, 0.03]; SMD: −0.43, 95% CI [−0.78; −0.09]).

**Conclusions:** Early mobilization within 72 h of ICU admission or after intubation had a positive albeit small effect on LOS in the ICU.


**Reference**
Bein T et al. Anaesthesist 2015;64:596–611.

**Figure Figec:**
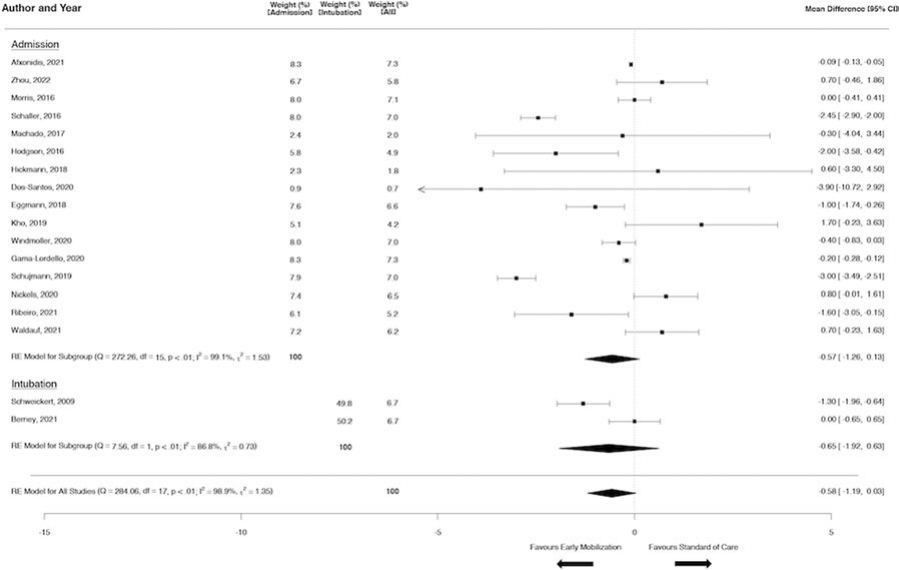
**Fig. 1 (abstract P333)**. Weighted mean difference of ICU LOS in relation to starting early mobilization within 72 h of ICU admission or intubation

## P334

### Improving patient outcomes-a challenging approach in discharge planning from intensive care to rehabilitation

#### A Goldenberg, Y Polishook, S Guetta

##### Kaplan Medical Center, General Intensive Care Unit, Rehovot, Israel

*Critical Care* 2023, **27(S1)**: P334

**Introduction:** By improving the discharge process from intensive care to rehabilitation facility it is possible to improve patient outcomes while maintaining continuity of quality care [1]. Planning discharge early saves hospital beds, shortens patients overall hospitalization, and increases patient-family satisfaction.

**Methods:** A team discussion based on patients individual requirements.Involvement of multi-disciplinary team. Development of discharge checklist to include:all the relevant information required to guarantee continuity of treatment, contacting facility to coordinate discharge,checking and meeting all pre-admission criteria to facility. Obtaining relevant authorization from Health Care Authority. Early involvement of family. Preparation of treatment summaries, transferring all relevant information.recording NEWS 2 score. After discharge follow up call.

**Results:** 48 intensive care patients that were either transferred out of ICU to a ward, or discharged to a rehabilitation facility over a two year period from 21.11.2019 until 7.6.2022. All rehabilitation facilities reported increased satisfaction with the discharge process. Average time from decision to discharge to actual discharge, being shortened from 5 to 2 days.

**Conclusions:** By utilizing planned discharge we were able to successfully discharge our patients from intensive care unit to rehabilitation facility, thereby improving patient outcomes. We prevented readmissions, shortened hospital stay, led to a reduction in overall ICU stay and increased satisfaction on the part of both rehabilitation facility and family.


**Reference**
Mcneill H et al. JMIR Perioper Med 2020;3:e13782.


## P335

### Acute stress disorder and post traumatic stress disorder after ICU admission for acute respiratory failure due to COVID-19

#### G Salve^1^, G Ceschin^2^, C Sonnati^2^, M Moscariello^2^, M Forte^2^, DM Piovera^1^, M Mangiagalli^1^, S Mongodi^1^, F Mojoli^1^

##### ^1^Policlinico San Matteo, Rianimazione I, Pavia, Italy, ^2^Policlinico San Matteo, Scuola di specializzazione Anestesia, Rianimazione, Pavia, Italy

*Critical Care* 2023, **27(S1)**: P335

**Introduction:** ICU survivors may present symptoms of acute stress disorder(ASD) or post traumatic stress disorder (PTSD) [1], as defined by the Diagnostic and Statistical Manual of Mental Disorders. Prevalence of PTSD in ARDS affects 20% of survivors but is higher in SARS/MERS patients [2]; risk factors are delirium, ICU sedation, altered memories or ICU amnesia [3].

**Methods:** We enrolled patients discharged from our ICU (Pavia-Italy) after admission for acute respiratory failure due to COVID-19. Written consent was collected. Inclusion criteria: age ≥ 18 years, ICU admission between February 2020 and July 2021 for severe COVID-19, SARS-CoV-2 infection confirmed by positive nasal swab/tracheal aspirate, available follow-up visit. Exclusion criteria: refuse to attend the follow up visit, refuse to perform the psychological tests. To assess ASD/PTSD we used the IES-R: a total mean score ≥ 33 was the cut-off value for ASD/PTSD; subscale scores for avoidance, intrusion, hyperarousal were calculated. Quantitative and categorical variables are expressed as median [IQR] and number (%).

**Results:** Of the 491 patients admitted to ICU, 113 patients were included (males 84(74.3%), age 61.0[52.0–66.0] years, BMI 28.2[25.8–31.6] kg/m^2^, SAPS2 31.0[26.0–41.5], ICU stay 17.0[8.0–28.0] days, invasive mechanical ventilation 61(54.0%). They were assessed in median 107.0 [82.0–150.0] days after ICU discharge. 37 patients (32.7%) had ASD/PTSD, higher than the expected 20% in general ARDS population (*p* < 0.001). Both intrusion 11.0[5.0–17.0] and avoidance 7.0[3.0–13.0] median scores were higher than hyperarousal 5.0[2.0–9.0].

**Conclusions:** ASD/PTSD is frequent after discharge to ICU for severe COVID-19, this prevalence is higher than other ARDS population, maybe for stigma, difficulties with quarantine and isolation. Patients have higher intrusion and avoidance scores, maybe because during the pandemic isolation was unavoidable.


**References**
Brown SM et al. Ann Am Thorac Soc 2019;16:947–56.Ahmed H et al. J Rehabil Med. 2020;52:jrm00063.Righy C et al. Crit Care 2019;23:213.


## P336

### Analysis of mortality and long-term prognosis in ventilated patients with COVID-19: a multicenter prospective observational study

#### K Nakamura^1^, J Hatakeyama^2^

##### ^1^Teikyo University Hospital, Department of Emergency and Critical Care Medicine, Tokyo, Japan, ^2^Osaka Medical and Pharmaceutical University, Department of Emergency and Critical Care Medicine, Osaka, Japan

*Critical Care* 2023, **27(S1)**: P336

**Introduction:** Long-term prognosis, especially for post-intensive care syndrome (PICS) is an emerging problem in critically ill patients. Prevalence and risk factors are unclear in patients with severe coronavirus disease 2019 (COVID-19). We aimed to investigate the prevalence and risks of mortality and PICS in ventilated patients with COVID-19.

**Methods:** A multicenter prospective study was conducted on ventilated patients with COVID-19 infection. The questionnaire for PICS evaluation was mailed within a median of 6 mo after hospital discharge, concerning Barthel Index, Short-Memory Questionnaire, and Hospital Anxiety and Depression Scale scores.

**Results:** 251 patients completed the PICS questionnaires with a prevalence of PICS of 58.6%, along with the highest percentages of cognitive impairment. Delirium (OR 2.34, *p* = 0.03) and the duration of mechanical ventilation (OR 1.29, *p* = 0.02) were identified as independent risks for PICS. In 297 patients who received mechanicalventilation for 7 day or longer, protein and energy delivery in day 4–7, especially for protein delivery, were independently and monotonically associated with in-hospital mortality, but not with PICS occurence.

**Conclusions:** 60% of the ventilated patients with COVID-19 suffered from PICS. Delirium and longer mechanical ventilation were identified as risks for PICS. In the patents requiring longer mechanical ventilation, nutrition delivery in the late period of the acute phase might be imprtant to survive COVID-19.

## P337

### A retrospective review of the functional status of patients post-severe COVID-19 disease

#### S Angelov, S O’Keefe, C Sheehan, Z Aslam, B O’Brien

##### Cork University Hospital, Anaesthesiology & Intensive Care Medicine, Cork, Ireland

*Critical Care* 2023, **27(S1)**: P337

**Introduction:** The purpose of this study was to evaluate the functional status and ongoing respiratory symptoms of patients who have been discharged home, who were admitted to the intensive care unit (ICU) with severe COVID-19 disease. Long term complications post critical illness, such as post intensive care syndrome (PICS), are well-recognised [1], however, little data exists specific to COVID-19 ICU survivors.

**Methods:** Local ethics committee approval was secured. Participants were recruited using the electronic intensive care chart database. All patients, from March 2020 to November 2021, who survived Covid-19 were included. Researchers conducted phone interviews with participants and completed three questionnaires; Katz Index of Independent Living Questionnaire, Karnofsky Performance Status Scale and the American Thoracic Society (ATS) Respiratory Disease Questionnaire. Data was input and analysed using excel and stored on a password encrypted computer.

**Results:** Database search revealed 61 patients available, 33 of 61 patients were included. 20 (61%) were male, 13 (39%) were female. Mean age was 58, with a range of 25–81. Mean length of ICU stay was 15 days, with a range of 1–74 days. 15 (45%) patients were intubated. Most common Katz score (range 1–6) was the highest score of 6 in 23 (70%) patients. Most common Karnofsky score (range 10–100) was 60 in 11 (33%) patients, with 31 (94%) scoring < 100. The most frequent ATS shortness of breath (SOB) grade (range 0–4) was 3 in 10 (9%) patients, with 23 (70%) patients scoring > 0. 14 (42%) patients had an ongoing cough, 11 (33%) patients had a wheeze and 0 (0%) patients were on home oxygen therapy.

**Conclusions:** This study highlights in the majority of patients who survived severe Covid-19 disease to discharge home, there were serious ongoing limitations to their functional status and exercise tolerance, however, they remained independent of basic activities of daily living.


**Reference**
Rawal G et al. J Transl Int Med 2017;5:90–92.


## P338

### The effect of consecutive COVID-19 waves on the mental wellbeing of ICU nursing staff

#### J Branders^1^, SN Noels^2^, MV Vreven^2^, GDH De Herdt^2^, HVE Vanden Eede^3^

##### ^1^ZNA Middelheim, Intensive care, Antwerp, Belgium, ^2^University Hospital Antwerp, Anesthesiology, Edegem, Belgium, ^3^ AZ Rivierenland, Anesthesiology, Rumst, Belgium

*Critical Care* 2023, **27(S1)**: P338

**Introduction:** Working on an ICU ward is considered a physical as well as mentally challenging profession which has already been objectified by multiple studies in the past. When the Covid-19 pandemic started, medical staff were suddenly exposed to even greater levels of stress not only at work but also due to social restrictions. Our primary aim of this cross-sectional study is to observe the evolution in time of the impact on mental health during as well as after the pandemic.

**Methods:** In this cross-sectional study the mental health of our ICU nursing staff (n = 18) was monitored over a period of 26 months by using validate questionnaires for anxiety, depression, insomnia and post-traumatic stress syndrome (PTSD) such as the Generalized Anxiety Disorder-7 (GAD-7) score, Patient Health Questionnaire-9 (PHQ-9), Insomnia severity index (ISI) and the revised version of the Impact of Event Severity scale (IES-r). In total it entailed 4 surveys during the pandemic and one follow up survey 6 months after.

**Results:** Regarding GAD7 and ISI, ICU nurses did not score significantly at the beginning nor the end of this study. IES scores started off at 18 which is just below significant but 35% had a score associated with developing PTSD. These scores remained relatively high throughout the pandemic but saw a mean downward trend at the end (*p* = 0.338). Mean PHQ-9 scores were initially at the level of “minimal depression” and rose to be classified as “mild depression” at the end of the pandemic. In the follow-up survey scores dropped back to “minimal depression”. In time, the PHQ-9 test was not withheld as significant (*p* = 0.298).

**Conclusions:** It is evident that the results presented in our study show that ICU nurses are prone to developing mental health problems during times of increased workload and stress and in particular the development of PTSD remained high in some subjects. Although there was a slight decrease in our follow-up survey, we believe mental health should be closely followed upon in the future.

## P339

### Potential benefits of automated infusion relay: survey of its possibilities from the material waste and cost perspective

#### E Meijer^1^, M Onrust^1^, W Dieperink^1^, F Doesburg^2^, W Bult^3^, M Nijsten^4^

##### ^1^UMC Groningen, Dpt of Critical Care, Groningen, Netherlands, ^2^UMC Groningen, Dpt of Medical Technology, Groningen, Netherlands, ^3^UMC Groningen, Dpt of Clinical Pharmacy and Pharmacology, Groningen, Netherlands, ^4^UMC Groningen, ICU, Groningen, Netherlands

*Critical Care* 2023, **27(S1)**: P339

**Introduction:** Manipulations with continuous syringe infusions in the ICU are time-consuming and cost-intensive. Nurses often have to exchange syringes at preplanned times, but pre-emptive swapping of syringes may result in considerable unused residual volume, and thus additional waste. During vasopressor infusion, automated infusion relay is usually considered for ensuring a smooth switching between syringe pumps. However, infusion relay also allows the optimal use of drug solutions and could present an opportunity to reduce environmental footprint and lower costs. Consequently, we studied the magnitude of unused drug disposal in our ICU in a context of manual syringe exchange, to evaluate the potential benefit of an automated infusion relay function.

**Methods:** We performed a prospective study over 3 weeks in a 32-bed tertiary university adult ICU. All patients, except strictly isolated patients (e.g. with COVID-19, MRSA or ESBL) were included. All 50 ml syringes that were disposed of before or after use were separately collected per patient and the constituent solution and the remaining volume were recorded. Medication costs, as used internally in our hospital, were identified for the 20 most commonly used drugs or solutions.

**Results:** A total of 2667 syringes from 105 subsequent adult ICU patients were collected and analyzed (Fig. 1). Expired medication or discontinuation of medication accounted for disposal of 179 (7%) completely filled syringes. The 2488 remaining syringes had a mean ± SD residual volume of 12 ± 13 ml (24%). This remaining medication volume had an associated cost of € 5736 or 28% of total cost of fully filled syringes, or at least €100,000 on an annual basis.

**Conclusions:** In our ICU approximately a quarter of syringe solutions are discarded during syringe switching. In terms of waste production, associated nursing time and associated material costs, more efficient syringe exchanges with automated infusion relay may address these three concerns.

**Figure Figed:**
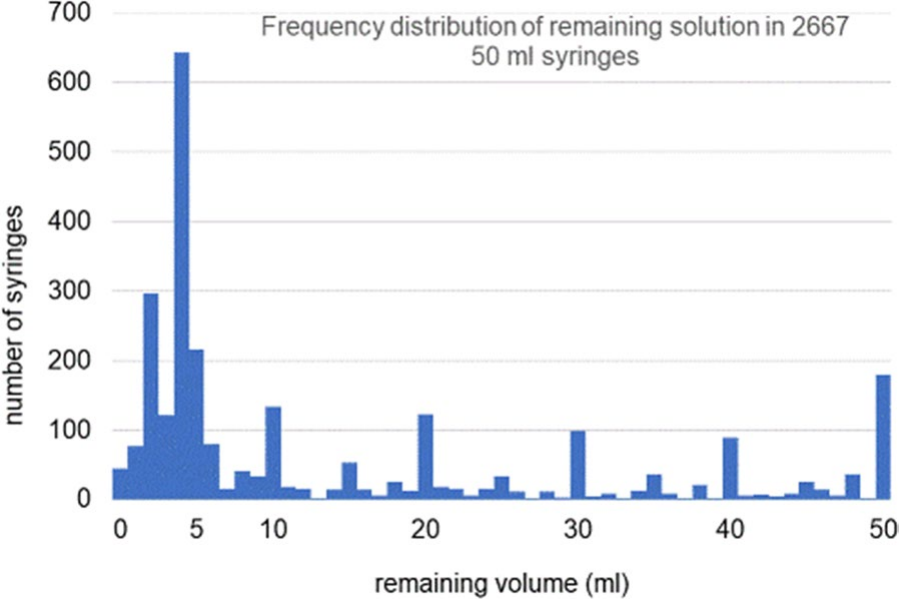
**Fig. 1 (abstract P339)**. Frequency distribution

## P340

### Identifying barriers to a robust second check of medication

#### V Milic, L Cameron, C Jones

##### St Thomas Hospital, Pharmacy, London, UK

*Critical Care* 2023, **27(S1)**: P340

**Introduction:** Medication errors are common in critically ill patients with a reported average of 1.7 medical errors each day [1]. Critically ill patients are at increased risk of medication error and patient harm, due to complex medication regimens with frequent changes, organ dysfunction increasing potential for harm from medication errors, and greater use of intravenous medications are associated with increased errors. Many hospitals have policies mandating an independent second check for high risk medication administration such as intravenous drugs or Controlled Drugs, with the intention of improving safety by identifying and preventing errors reaching the patient. This second checking process has a significant time implications and the efficacy for error prevention is debated with adherence to second checking being documented between 52 and 72% within literature [2, 3]. The aim of this study was to determine the barriers for a robust independent second check of medication within adult critical care.

**Methods:** Four focus groups were conducted with critical care nurses at Guy’s and St Thomas’ Foundation Trust. Thematic analysis was undertaken to identify potential barriers to implementing an effective second check. Themes were then analysed for thematic content by three critical care pharmacists and grouped with evidence to support or refute the themes.

**Results:** Subthemes and themes are described in Table 1. The main themes identified as barriers to an independent second checking were complex processes, IT, routine, personnel and geography.

**Conclusions:** Our study identified a number of common themes associated with failures to implement an effective second check for medication in a critical care. The main barriers identified were personnel, geography and routine. Overall, ethnographic studies are required at ward level as a bigger piece of research to try to further understand second checking.


**References**
Moyen E et al. Crit Care 2008;12:208.Schilp J et al. BMJ Open 2014;4:e005232.Alsulami Z et al., J Adv Nurs 2014;70:1404–13.

**Table Tabsd:** **Table 1 (abstract P340)**. Themes and subthemes identified from 29 nurses across 4 focus groups

Themes	Shortcuts, multiple competing priorities, time
1. Complex process	Timing of medication, co-signing, shortcuts
2. IT	Timing of medication, varying IT systems
3. Routine	Unwritten rules, process, familiarity, culture
4. Personnel	Protecting self, seniority of staff, staff
5. Geography	Time of day, bed location

## P341

### Implementation of clinical pharmacy services in the ICU

#### FD Duchi

##### AZ Groeninge Kortrijk, Hospital Pharmacy, Kortrijk, Belgium

*Critical Care* 2023, **27(S1)**: P341

**Introduction:** Because of the severity of the disease, the use of high-risk medication, the high frequency of changes in medication charts, and polymedication, patients admitted to the ICU are at high risk for the occurrence of clinically relevant drug-related problems (DRPs). One way to reduce the risk of DRPs is the presence of a hospital pharmacist in the ICU. Since Belgian healthcare is organized differently and the presence of a hospital pharmacist in ICU teams is not well established, a study was conducted to implement clinical pharmacy services.

**Methods:** A prospective non-controlled interventional six-week study was conducted in a 31-bed intensive care unit in az groeninge Kortrijk, Belgium. The hospital pharmacist was involved in the evaluation of the medication chart of the patient, attending medical rounds, and answering drug-related questions. Clinical interventions were presented to physicians. The acceptance rate was measured, and potential risk factors associated with the acceptance rate were identified.

**Results:** A total of 204 DRPs were detected. Physicians accepted 158 interventions, resulting in an acceptance rate of 77.5%. Statistical analysis showed that the acceptance rate was significantly associated with the type of proposed intervention. The intervention to change a drug and to pause or stop a drug was implemented more frequently.

**Conclusions:** Providing patient care to critically ill patients by a multidisciplinary team is an emphasis of the modern day ICU because the team approach is invaluable. Through proactive participation in evaluating the appropriateness of the medication chart, identifying and reporting DRPs, and proposing evidence-based interventions, the pharmacist contributed to the multidisciplinary team in the ICU. Quality improvement by the implementation of clinical pharmacy resulted in high acceptance rates of proposed interventions and high appreciation for the presence of a hospital pharmacist from health care providers in the ICU.

## P342

### Adult admissions to an intensive care unit due to adverse drug reactions

#### G Masiliūnienė^1^, E Kaduševičius^2^, E Stankevičius^2^

##### ^1^Kaunas Hospital of the Lithuanian University of Health Sciences, ICU, Kaunas, Lithuania, ^2^Lithuanian University of Health Sciences, Lithuanian University of Health Sciences, Kaunas, Lithuania

*Critical Care* 2023, **27(S1)**: P342

**Introduction:** Adverse drug reactions (ADRs) remain a huge burden on health care, leading to significant morbidity, mortality, and costs. Based on different literature resources, the prevalence of ADR-related hospital admissions ranges from 0.2 to 54.5% [1]. The costs of an ADR have been reported to be substantial with an additional $6000–$9000 for each event [2]. The aim of this study was to determine the frequency, probability, and severity of ADRs and ADR-related costs in patients admitted to the ICU.

**Methods:** This prospective consecutive study was carried out in the ICU of Kaunas Hospital of the Lithuanian University of Health Sciences from September 2021 to November 2022. Adult patients admitted to the ICU because of an ADR were included. The probability of suspected ADRs was evaluated using the Naranjo Scale. The severity of ADRs was assessed by the Modified Hartwig and Siegel Scale.

**Results:** A total of 768 patients were admitted to the ICU during a 15-month period. Of them, 43 (5.6%) admissions were related to ADRs. The mean age of patients admitted due to ADRs was 76.7 years (SD 13.8; range, 33–97). Most of the suspected ADRs (74.5%) were classified as probable; 16.3%, as definite; and 9.3%, as possible. The majority (93%) of ADRs were categorized as severe (of them, 32.5% resulting in death) and the remaining ADRs were moderate. The most commonly reported ADR was bleeding (53.5%) followed by hyponatremia and bradycardia (each 14%), diarrhea (11.6%) and other such as coma, pulmonary edema, etc. (7%). The costs per event ranged from €502 to €11,280. Health care expenditures attributed to ADR-related admissions to the ICU totaled more than €172,000.

**Conclusions:** Our study showed that the overwhelming majority of ADRs were severe and one-third of them led to a fatal outcome. This warrants future studies on preventability and solutions how to reduce the costs of ADR-related ICU admissions.


**References**
Angamo MT et al. Drug Saf 2016;39:847–57.Kane-Gill SL et al. Crit Care Med 2010;38:S83–9.


## P343

### Improving drug preparation during emergencies and rapid sequence induction in the adult intensive care unit

#### I Canisius, A Brown, C Lambert

##### St Mary’s Hospital, Adult Intensive Care, London, UK

*Critical Care* 2023, **27(S1)**: P343

**Introduction:** We aimed to improve efficiency and safety in preparing emergency and rapid sequence induction drugs (ERDs). Critically ill patients are exposed to multiple drugs, and the fast-paced ICU environment is an accepted contributor towards error [1]. Emergencies and intubation involve particularly high-risk injectables, and errors may have devastating effects. Drug preparation may be delegated to doctors of varying experience, causing potential error or delay. We instituted dedicated ERD preparation stations containing drugs, equipment and a quick-reference table. As per European guidance [2], we introduced pre-filled syringes of drugs that would otherwise need dilution, to avoid miscalculation and extra steps.

**Methods:** Efficiency and safety in ERD preparation was assessed pre- and post-intervention. Doctors were surveyed regarding knowledge and confidence levels. Two subsets of 10 doctors completed a practical task pre- and post- intervention to record the time taken to prepare a full set of ERDs. They were each given a drug reference table to mitigate prior knowledge as an influencing variable. Participants completed feedback to identify equipment and process factors that they felt caused delays or risk of error.

**Results:** 20 pre- and 11 post-intervention survey responses were collected. 91% of responders felt that the new stations had improved safety and efficiency. There was a 61% increase (*p* = 0.003) in awareness of drug locations post-intervention. In the practical assessments, the average time taken to prepare a full set of drugs decreased from 20 to 7 min pre- to post-intervention (*p* = 0.00009).

**Conclusions:** Dedicated drug preparation stations have improved efficiency and drug location knowledge. The stations will be extended to other trust ICUs, where we aim to re-audit with a larger sample. Quantifying impact on drug error rate and associated avoidable patient harm requires longer-term study.

**Acknowledgement:** I. Canisius and A. Brown contributed equally to the study as first author.


**References**
Kane-Gill S et al. Crit Care Clin 2006;22:273–290.Whitaker D et al. Eur J Anaesthesiol 2017;34:4–7.


## P344

### Prevalence of frailty in critically ill cancer patients and its impact on acute outcomes and post ICU discharge quality of life

#### SK Sundaramurthy^1^, R Dominic Savio^1^, S Thomas^1^, L Ranganathan^2^, R Venkataraman^2^, N Ramakrishnan^2^, DD Vidyasagar^3^

##### ^1^Apollo Proton Cancer Centre, Critical Care Medicine, Chennai, India, ^2^Apollo Hospital, Critical Care Medicine, Chennai, India, ^3^Apollo Cancer Hospital, Teynampet, Critical Care Medicine, Chennai, India

*Critical Care* 2023, **27(S1)**: P344

**Introduction:** The impact of frailty on critically ill cancer patients remains to be elucidated. We sought to assess its prevalence in this cohort and its impact on acute outcomes and 90-day post hospital discharge health related quality of life (HRQoL).

**Methods:** Single centre, prospective, cohort study conducted in the intensive care unit (ICU) of a tertiary care, cancer hospital. All consecutive adult patients with cancer, admitted to ICU over a 10 months period were included in the study. Patients on end-of-life care were excluded. Frailty was assessed upon ICU admission using the Clinical Frailty Scale (CFS version 2.0) by two independent investigators [1]. Data pertaining to demographics and outcomes were documented. The acute outcomes analysed were requirement for respiratory and vasopressor support, time to mobilization, ICU readmission, ICU length of stay (LOS), hospital LOS and in-hospital mortality. Patients were followed-up at 90 days post hospital discharge with a phone interview to assess their HRQoL using the EuroQol 5D 5L questionnaire [2].

**Results:** A total of 516 patients were included in the study. All of them were diagnosed to have frailty (77.9% mild). There was moderate inter-observer agreement on CFS (0.552 kappa coefficient). Advanced age (*p* = 0.046) and malnutrition (*p* = 0.027) were found to be significant risk factors. All acute outcomes, namely, requirement for respiratory (*p* < 0.01) and vasopressor (*p* < 0.01) support, time to mobilization (*p* = 0.01), ICU readmission (*p* < 0.01), ICU LOS (*p* < 0.01), hospital LOS (*p* < 0.01) and in-hospital mortality (*p* = 0.01) were adversely affected by frailty. Patients with higher frailty reported significantly poorer health scores (*p* = 0.01) and had worse performance among four out of the five dimensions in EQ 5D 5L at 90 days.

**Conclusions:** Critically ill cancer patients have a high prevalence of frailty and this adversely affects acute outcomes and 90-day post discharge HRQoL.


**References**
Rockwood K et al. Can Geriatr J. 2020;23:210–215.Janssen MF et al. Qual Life Res 2013;22:1717–1727.


## P345

### Impact of frailty on long-term quality of life in patients undergoing cardiac surgery

#### M Schroyens^1^, SM Bagshaw^1^, CM Norris^2^, DB Rolfson^3^, SR Meyer^4^, HT Stelfox^5^, MA Zibdawi^1^, CL Montgomery^2^

##### ^1^University of Alberta, Department of Critical Care Medicine, Edmonton, Canada, ^2^University of Alberta, Faculty of Nursing, Edmonton, Canada, ^3^University of Alberta, Division of Geriatric Medicine, Department of Medicine, Edmonton, Canada, ^4^University of Alberta, Division of Cardiac Surgery, Department of Surgery, Edmonton, Canada, ^5^University of Calgary, Department of Critical Care Medicine, Cumming School of Medicine, Calgary, Canada

*Critical Care* 2023, **27(S1)**: P345

**Introduction:** Frailty is associated with a risk of worse outcomes and higher health services use in adult patients undergoing cardiac surgery. In this study we assessed the impact of baseline frailty status on health-related quality of life (HRQL) at 12 months following cardiac surgery.

**Methods:** This prospective cohort study included patients aged 50 years and older who underwent non-emergent surgery between November 2011 and March 2014 in two adult cardiac surgery programs in Alberta, Canada. Frailty was defined as a Clinical Frailty Scale (CFS) score of 5 or greater. HRQL was assessed using the EuroQol 5-domain 3-level (EQ-5D) and Short Form-12 (SF-12) surveys in person pre-operatively and by telephone at 6 months and at 12 months after surgery. Patient socio-demographics, comorbidities, surgery details, cardiac surgery risk scores, adverse events and discharge disposition were also collected.

**Results:** The cohort (529 patients) mean (SD) age was 67 (9) years. 25.9% were female and the pre-operative prevalence of frailty was 9.6%. Patients with frailty were older (median [IQR] age 75 [65–80] v. 67 [60–73], *p* < 0.001) and had higher EuroSCORE II (8 [6–9] v. 5 [3–7], *p* < 0.001). SF-12 physical component score increased from baseline to 12-months in frail (27.5 [9.4]–38.2 [11.9], *p* < 0.001) and non-frail patients (37.0 [11.4]–47.3 [9.9], *p* < 0.001). Mental component score in patients with frailty was similar (47.7 [12.0]–52.2 [10.4], *p* = 0.29) and increased in non-frail (51.8 [10.7]–54.9 [8.5], *p* < 0.001). Mean (SD) EQ-5D VAS increased in frail (46.2 [18.9]–60.3 [21.6], *p* < 0.001) and non-frail (60.2 [20.2]–76.6 [15.4], *p* < 0.001).

**Conclusions:** HRQL was more impaired in patients with frailty at baseline although incremental improvement in HRQL was reported in frail and non-frail patients over 12-months post-operatively. Recognizing the risk to HRQL among patients with frailty pre-operatively requires further exploration to identify potential interventions to optimize outcomes.

## P346

### Validity of physician-perceived frailty in the intensive care unit

#### Y Angel, O Marudi, A Nini, D Stavi, A Nevo, D Aviram, Y Lichter, N Adi, I Matot, A Gal-Oz

##### Tel Aviv Sourasky Medical Center, Division of Anesthesia, Pain Management and Intensive Care, Tel Aviv, Israel

*Critical Care* 2023, **27(S1)**: P346

**Introduction:** Frailty is a valuable predictor for outcome in elderly intensive care unit (ICU) patients. Physicians in ICU often describe patients as “frail” in reference to their pre-morbid condition and possibly their chances of recovery following ICU admission. Several validated scores to assess frailty have been developed, such as the Clinical Frailty Scale (CFS) and the Modified Frailty Index (MFI). These scores correlate well with post-operative and post-ICU mortality [1–3]. Nonetheless, in our clinical experience, ICU physicians more often opt to use their clinical judgement, or “eyeball assessment”, to estimate the degree of frailty.

**Methods:** A retrospective study of patients admitted to a single center ICU in a large academic medical center in Tel Aviv during March and April 2019. Physician-perceived frailty, as reported on admission on an ordinal scale of no frailty, mild, moderate, or severe frailty, was compared with results of validated scores such as CFS and MFI calculated retrospectively from patient records.

**Results:** 100 consecutive patients were included; 60 [60%] were male, mean age 58 ± 18 years. The Spearman Correlation test measured a correlation of 0.31 (*p* = 0.002) between physician-perceived frailty and CFS and 0.1 (*p* = 0.326) between physician-perceived frailty and MFI. On multivariate logistic regression, physician-perceived frailty was found to be independently associated with 30-day mortality (OR 2.16, *p* < 0.001; Fig. 1) but not with the duration of mechanical ventilation, ICU or hospital stay.

**Conclusions:** In this single-center, retrospective study, no correlation was found between physician-perceived frailty and CFS or MFI scores. Nevertheless, physician-perceived level of frailty was significantly associated with 30-day mortality. Larger and prospective studies are needed to ascertain these results.


**References**
Chen X et al. Clin Interv Aging. 2014;9:433–41.Dasgupta M et al. Arch Gerontol Geriatr. 2009;48:78–83.Le Maguet P et al. Intensive Care Med. 2014;40:674–82.

**Figure Figee:**
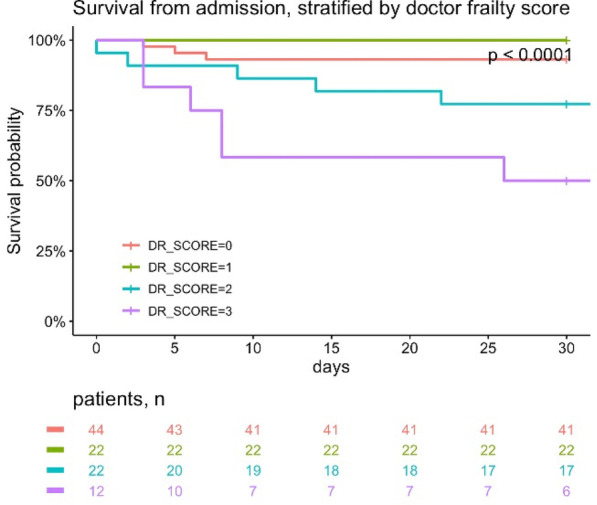
**Fig. 1 (abstract P346)**. Kaplan-Meier curve showing 30 day survival stratified by physician-perceived frailty score on admission

## P347

### Frailty among critically ill adults in India and association with patient-centered outcomes: a registry embedded cohort study

#### BK Tirupakuzhi Vijayaraghavan, IRIS Frailty Investigators

##### Apollo Main Hospital, Department of Critical Care Medicine, Chennai, India

*Critical Care* 2023, **27(S1)**: P347

**Introduction:** There are limited data on frailty and outcomes for critically ill patients in low/middle income settings. We studied the prevalence of frailty at admission and evaluated association with patient-centred outcomes.

**Methods:** Prospective registry-embedded cohort study conducted in 7 Indian ICUs (4 hospitals). Adult patients anticipated to stay for ≥ 48 h were included. Exposure of interest was frailty as defined by the Clinical Frailty Scale (> 5) and the primary outcome was ICU mortality. Additional outcomes were in-hospital mortality, receipt of invasive or non-invasive ventilation or kidney replacement therapy, stage 2/3 acute kidney injury (AKI), and length of ICU stay. Categorical outcomes were compared using Chi-square and continuous outcomes using Mann–Whitney test. For analyses of ICU and hospital mortality, we employed generalized linear models; associations are reported as odds ratios (OR) and 95% confidence intervals (CI).

**Results:** 838 patients were included. Median (IQR) age was 57 (42.68) years and 65.0% were male. The prevalence of frailty was 166/838 (20.0%). The median (IQR) APACHE II among frail patients was 15(10–20) as compared to 11(7–16) among the non-frail and median (IQR) Charlson comorbidity score was 3(1–4) among frail patients as compared to 1(0–3) among the non-frail. Frailty was associated with ICU mortality (adjusted OR1.91, 95% CI 1.30–3.11), hospital mortality (adjusted OR 2.22, 1.44–3.39), receipt of non-invasive ventilation (unadjusted OR 2.68, 1.77–4.03), receipt of kidney replacement therapy (unadjusted OR 3.15, 1.90–5.17), receipt of vasopressors (unadjusted OR 1.47, 1.04–2.07), and stage 2/3 AKI (unadjusted OR 2.35, 1.60–3.43) (Fig. 1). There was no association with invasive ventilation (unadjusted OR 1.11, 0.71–1.57). Frail patients stayed longer in the ICU (median 6 days vs. 4 days, *p* < 0.001).

**Conclusions:** Frailty is common among critically ill patients in India and associated with worse outcomes.

**Figure Figef:**
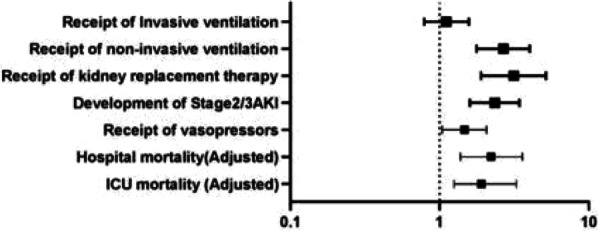
**Fig. 1 (abstract P347)**. Forest plot of outcomes for frail vs. non-frail critically ill adults

## P348

### Prognosis of chronic critical illness (CCI)/persistentinflammation immunosuppression and catabolism (PICS)

#### Q Zhou, J Liu

##### The Affiliated Suzhou Hospital of Nanjing Medical University, Suzhou Municipal Hospital, Suzhou Clinical Medical Center of Critical Care Medicine, Department of Critical Care Medicine, Suzhou, China

*Critical Care* 2023, **27(S1)**: P348

**Introduction:** To investigate and compare the clinical features and prognosis of chronic critical illness (CCI)/persistent inflammation immunosuppression and catabolism (PICS).

**Methods:** This is a prospective observational clinical study. During this study period, we collect ICU patients’ data from Suzhou Municipal Hospital and Suzhou Ninth People’s Hospital. All patients older than 18 years of age were included and according to the corresponding exclusion and diagnostic criteria, they were divided into four groups: PICS group, CCI group, CCI and PICS group (CCI + PICS), nor CCI and nor PICS group (NCCI + NPICS), collected and recorded age, sex, hospital time, hospital diagnosis, acute physiological and chronic health status score II (APACHE II), sequential organ failure detection score(SOFA), c-reactive protein(CRP), absolute value lymphocyte count (L),serum albumin (Alb), white cells count (WBC), absolute value neutrophils count (N), secondary infection and 28-days case fatality rate separately.

**Results:** A total of 687 patients were admitted to the ICU during the study period. The hospitalization time less than 14 days were excluded and 168 patients were eventually included. There are 17 in the PICS group,71 in the CCI group,50 in the CCI + PICS group and 30 in the NCCI + NPICS group. Baseline characteristics showed statistically significant differences in SOFA, length of hospital stay, 28-day mortality among four groups. Baseline, main indicators and multiple comparisons showed that the CCI + PICS group had longer hospital stay,worse prognosis and more adverse outcomes. Multivariate analysis showed that final age, CRP on day 14 and 21, serum albumin on day 1 and 21 had an impact on the prognosis (*p* < 0.05).

**Conclusions:** The clinical prognosis of the four groups decreased in order of NCCI + NPICS, CCI, PICS, CCI + PICS. Our finding of clinically isolated PICS may indicate that PICS acts as a trigger or independent factor to worsen the prognosis of CCI.

## P349

### New-onset mental illness and long-term survival in survivors of critical illness: a population-based cohort study in South Korea

#### HY Jo, TK Oh, JW Hwang

##### Seoul National University Bundang Hospital, Anesthesiology and Pain Medicine, Seongnamsi, South Korea

*Critical Care* 2023, **27(S1)**: P349

**Introduction:** Intensive care unit (ICU) survivors are at higher risk of developing mental illness. We aimed to examine the prevalence and associated factors of newly developed mental illness among ICU survivors of critical illness. Moreover, we examined the association between newly developed mental illness and the 2-years all-cause mortality.

**Methods:** All adult patients (≥ 20 years) who were admitted to the ICU during hospitalization between 2010 and 2018 and survived for 1-year were defined as ICU survivors and were included in this nationwide population-based cohort study. ICU survivors with a history of mental illness before ICU admission were excluded from the study. Depression, anxiety disorder, bipolar disorder, insomnia disorder, and substance abuse were considered as mental illnesses.

**Results:** A total of 1,316,776 ICU survivors were included in the analysis, and 21,274 survivors (1.6%) had newly developed mental illness within 1-year of ICU admission. Old age, longer ICU stay, increased Charlson comorbidity index, hospital admission through the emergency room, increased total cost of hospitalization, mechanical ventilator support, extracorporeal membrane oxygenation support, and continuous renal replacement therapy were associated with an increased prevalence of newly developed mental illness. Moreover, the newly developed mental illness group showed a 1.71-fold higher 2-years all-cause mortality rate than the no mental illness group (hazard ratio: 1.71; 95% confidence interval: 1.65–1.77; *p* < 0.001).

**Conclusions:** In South Korea, 1.6% of ICU survivors had newly developed mental illness within 1-year of ICU admission. Moreover, newly developed mental illness was associated with an increased 2-year all-cause mortality.

## P350

### Use of healthcare before and after sepsis: a case control study

#### J Dahlberg^1^, A Linder^1^, L Mellhammar^2^

##### ^1^Lund University, Lund, Sweden, ^2^Lund University, Department of Clinical Sciences, Lund, Sweden

*Critical Care* 2023, **27(S1)**: P350

**Introduction:** The aim of this study was to compare prevalence of readmissions and death between sepsis and non-sepsis hospitalizations the first year after discharge, and to investigate what diagnoses sepsis-patients present with at readmission. The aim was also to evaluate to what degree patients hospitalized for sepsis seek medical attention prior to hospitalization.

**Methods:** This was a retrospective case–control study with data validated through clinical chart review. A disproportionate stratified sampling model was used to include a relatively larger number of sepsis hospitalizations. All eight public hospitals in region Scania, Sweden were included (January 1 to December 31, 2019). 447 patients hospitalized for sepsis (cases), and 541 patients hospitalized for other causes (control) were identified through clinical chart review. Logistic regression was used to analyze healthcare the week prior to hospitalization, and cox regression was used to analyze death and readmission the year after discharge.

**Results:** 48% of patients that survived a sepsis hospitalization were readmitted the year after discharge, compared to 39% for non-sepsis patients (HR 1.50, 95% CI 1.03–2.19), *p* = 0.04 (Fig. 1). 32% of readmissions were caused by infection, and 18% by cardiovascular disease. 52% of readmissions occurred within 90 days and 75% within 180 days. Finally, 34% of sepsis patients had sought prehospital contact with a physician the week before hospitalization, compared to 22% for non-sepsis patients (OR 1.80, 95% CI 1.06–3.04), *p* = 0.03.

**Conclusions:** Patients hospitalized for sepsis had higher risk of readmission the year after discharge compared to non-sepsis patients. The most common diagnoses at readmission were infection followed by cardiovascular disease. With better follow up, some of these readmissions could potentially be prevented. Patients hospitalized for sepsis had sought prehospital contact the week prior to hospitalization to a greater extent than non-sepsis patients.

**Figure Figeg:**
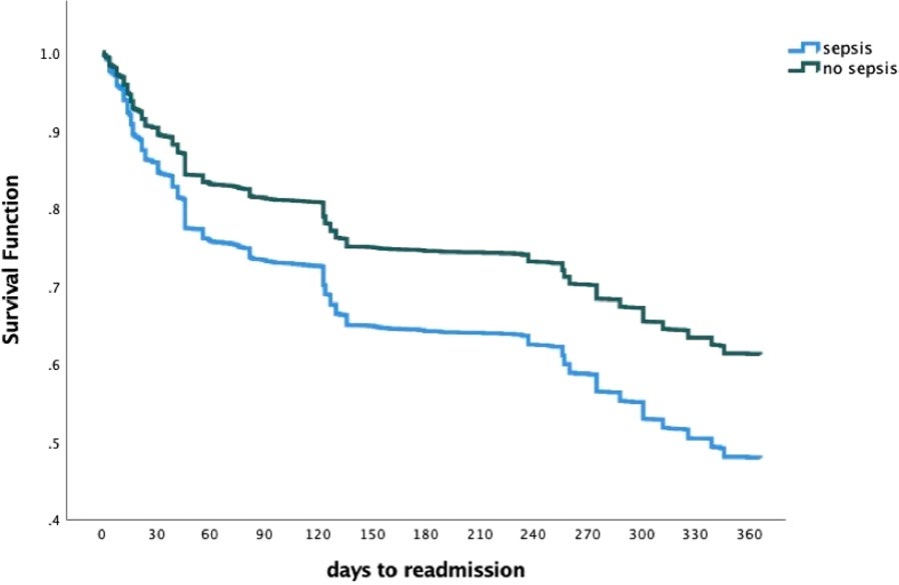
**Fig. 1 (abstract P350)**. Unadjusted survival function for readmissions the year after discharge, with separate lines for sepsis and non-sepsis hospitalizations

## P351

### Resident-led simulation-based course increases first-year medical residents’ confidence in managing common critical care scenarios

#### M Vanden Eede^1^, H Vanden Eede^2^, D De Blick^3^, N Van Regenmortel^4^, K Monsieurs^3^, P Mertens^5^

##### ^1^Universitair Ziekenhuis Antwerpen, Intensive Care, Edegem, Belgium, ^2^AZ Rivierland, Anesthesiologie, Reet, Belgium, ^3^Universitair Ziekenhuis Antwerpen, Emergency Department, Edegem, Belgium, ^4^ZNA Stuivenberg, Intensive Care, Antwerpen, Belgium, ^5^Universitair Ziekenhuis Antwerpen, Anesthesiologie, Edegem, Belgium

*Critical Care* 2023, **27(S1)**: P351

**Introduction:** Simulation-based education (SBE) allows First-Year Residents (FYR) to practice their clinical, cognitive, and behavioral skills without having to deal with actual patients. This creates a secure learning environment where mistakes can be made safely [1]. SBE is commonly organized by medical faculties and includes feedback from experienced educators. The main disadvantage is that medical simulations are resource intensive, posing significant organizational challenges due to limited staff availability, compromising training opportunities [2]. In order to improve SBE delivery possibilities, senior residents could be engaged as teachers. We evaluated the effect of a resident-led SBE program on the confidence level of FYR to manage common critical care scenarios.

**Methods:** This study included FYR from internal medicine, anesthesiology and emergency medicine. The course was voluntary and encompassed an online module, followed by 4-h simulation training containing eight common critical care scenarios. Senior residents (third year or higher) who facilitated the training received a one-day training on didactic principles. FYR self-confidence was measured before and after the course and rated on a scale from 0 to 100% using a validated questionnaire [3].

**Results:** Forty FYR participated in the course. Survey data indicated a significant improvement in the confidence to lead resuscitations and to take care of common critical situations as depicted in Table 1 below.

**Conclusions:** A resident-led course that consisted of an online module and four hours of simulation training significantly improved FYR confidence in leading resuscitations and handling common critical care scenarios.


**References**
Issenberg SB et al. JAMA. 1999;282(9):861–6.Seethamraju RR et al. Simul Healthc. 2022;17:e113–121.Arnold JJ et al. Clin Sim Nurs. 2009;5:e35–34.

**Table Tabdw:** **Table 1 (abstract P351)**. Wilcoxon signed-rank test comparison of median confidence of first-year residents in leading resuscitations and taking care of common critical care scenarios before and after a resident-led simulation course

Ability	Median pre-course confidence % (IQR)	Median post-course confidence % (IQR)	*p* value
Leading a resuscitation	65 (20–70)	70 (55–80)	< 0.001
Managing myocardial infarction	60 (52–75)	75 (70–82)	0.005
Managing gastro-intestinal bleeding	60 (50–70)	70 (60–80)	0.006
Managing hyperkalemia	55 (42–65)	75 (67–80)	0.005
Managing respiratory distress	60 (40–70)	70 (65–81)	< 0.001
Managing septic shock	60 (45–70)	70 (67–81)	< 0.001
Managing anaphylaxis	65 (45–70)	75 (70–85)	0.002

## P352

### Simulation-based clinical procedure education in the ICU

#### M Akatsuka, H Tatsumi, Y Masuda

##### Sapporo Medical University School of Medicine, Department of Intensive Care Medicine, Sapporo, Japan

*Critical Care* 2023, **27(S1)**: P352

**Introduction:** The recent emphasis on hands-on teaching and training to develop professionalism for medical students and residents has placed significant demands on educational institutions for medicine. The traditional method of transmitting specialized knowledge through role modeling when educating students on clinical procedures has given rise to issues such as inadequate understanding and difficulty in maintaining motivation. Therefore, we focus on simulation-based education and group discussion. The aim of this research was to evaluate the state of training and clarify future issues.

**Methods:** Simulation-based education was performed for medical students and residents and a questionnaire survey was conducted to determine the impact the training had on them. The research was performed between January 2022 and October 2022. We have the following four steps: classroom learning; simulation-based education giving the roles of surgeon, caregiver and instructor; group discussions; and summary with the supervising physician.

**Results:** A total of 75 medical students and 5 residents participated, and all of the participants reported that the simulation exercise was useful. When residents performed the procedure on an actual patient after the simulation education, 80% of the residents were successful in their first attempt. Most of the post-procedure comments were positive, such as participants having “more confidence in the procedure” and “a clear understanding of the points of procedure”. However, there were some negative comments about anxiety due to the difference between the simulator and a patient.

**Conclusions:** The results suggest the need for future clinical procedure education to be conducted comprehensively through classroom lectures, simulation education, and practice. A faculty development programme designed to support the teaching and evaluation for faculty members would be helpful for medical students and residents to gain a deeper understanding of clinical procedures and acquire practical skills for patients.

## P353

### Implementing peer support in our intensive care unit to improve the mental wellbeing of trainees

#### M Quance, B Gay, E Howe, E Harlow, S Jarvis

##### Royal Cornwall Hospital, Critical Care, Truro, UK

*Critical Care* 2023, **27(S1)**: P353

**Introduction:** Working on the intensive care unit (ICU) exposes staff to the suffering of patients and their families as a consequence of devastating illnesses and injuries, often leading to psychological trauma. The COVID-19 pandemic has amplified pressures on healthcare staff, resulting in deteriorating mental health and increased incidence of stress and burn out. The Intensive Care Society (ICS) has placed emphasis on peer support to combat this; recognising that working in a ‘psychologically safe’ environment improves wellbeing, enables safe and compassionate care, and reduces mistakes [1]. The aim of peer support is to improve wellbeing by offering a systematic, strategic approach to sustain staff who are coping well and provide initial support to those who are struggling.

**Methods:** Staff who have undertaken ICS training, under the supervision of a clinical psychologist, facilitate fortnightly meetings open to all ICU trainees. They also act as an initial contact to provide advice and guidance on addressing the psychological difficulties highlighted above. An anonymous screening questionnaire at the start of each session allows a moment of reflection for trainees prior to discussion. This, in addition to anonymous feedback, provides qualitative data to both audit the programme and inform improvement.

**Results:** Participation in peer support is voluntary, nonetheless attendance at meetings has been excellent. Initial qualitative results indicate that most trainees feel ‘nervous, anxious or on edge’, ‘question their capability at work’ and are ‘unable to stop thinking about work/a significant event at work’ at least a few days per week. Feedback from trainees regarding the programme has been intensely positive.

**Conclusions:** Anxiety and stress are high amongst ICU trainees which can lead to psychological harm if not addressed. Engagement from trainees in our programme has been good and feedback positive, emphasising the need for peer support in the ICU.


**Reference**
Williams R et al. ICS peer support framework, 2020.


## P354

### Experience of early postgraduate exposure to intensive care medicine: a phenomenological study

#### D Enright^1^, R Colgan^2^, E O’Connor^3^

##### ^1^University of Galway, Galway, Ireland, ^2^Mater Misericordiae University Hospital, Dublin, Ireland, ^3^St James’s Hospital, Intensive Care, Dublin, Ireland

*Critical Care* 2023, **27(S1)**: P354

**Introduction:** While clinical role transition is known to pose a challenge to doctors in training, and the intensive care unit (ICU) noted to be a demanding workplace environment, relatively little is known about the experience of doctors as they transition to intensive care medicine. Thus, this study aimed to explore the experience of registrar-grade doctors undergoing the transition to intensive care medicine (ICM), and to examine the interplay between personal and professional life during this transition.

**Methods:** This qualitative study was guided by transcendental phenomenology and multiple and multimodal transitions theory (MMT). Data were collected via eleven semi-structured interviews, which were analysed using a six-step thematic analysis. Peer debriefing, audit trail and a reflexive diary were used.

**Results:** The experience of doctors undergoing the transition to ICM was found to be influenced by the clinical environment of the ICU, which was noted to be a demanding and potentially stressful workplace, and one in which the support of colleagues was greatly valued (Fig. 1). The individual characteristics of the doctor undergoing the transition impacted upon this transition, as their previous experience and the impact of the transition on their professional development and identity were observed to be influential. In keeping with MMT, the interplay between personal and professional life was significant, as participants outlined the impact of anxiety before a work shift, the emotional and cognitive burden of a shift, and the effect of the transition to ICM on their personal relationships.

**Conclusions:** This study observes the ICU to be an extremely challenging workplace environment, with a notable influence on the personal lives of those working therein. Nevertheless, ICM offers invaluable opportunity for learning and both personal and professional growth.

**Figure Figeh:**
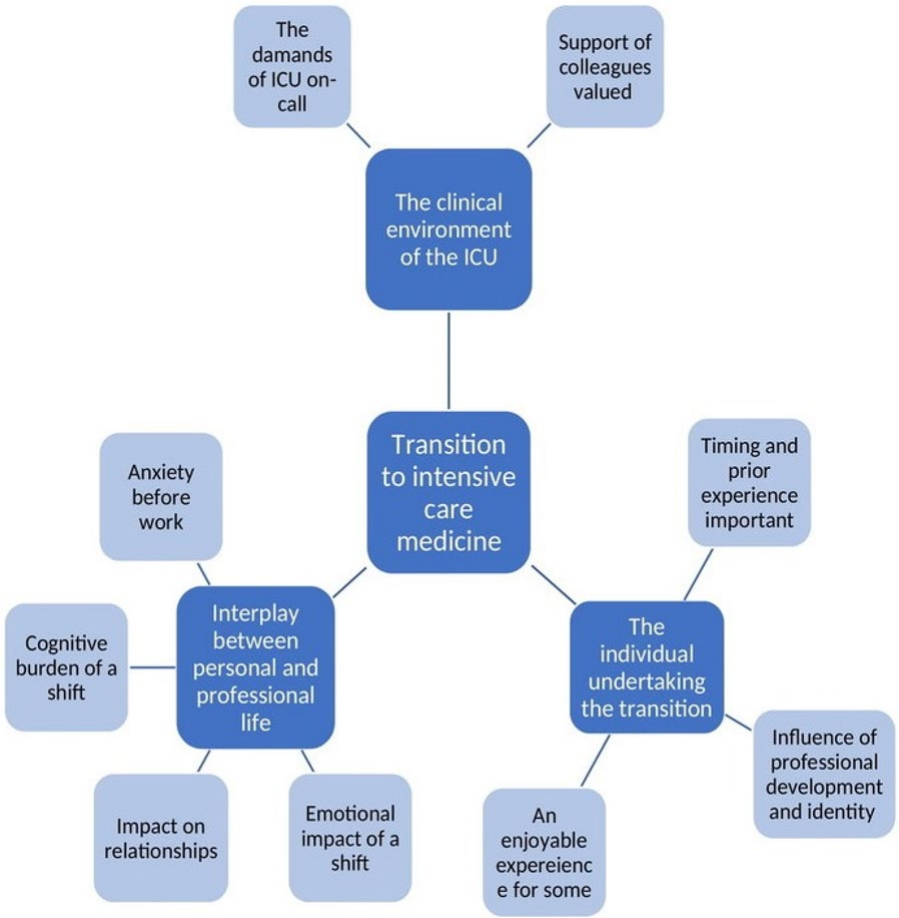
**Fig. 1 (abstract P354)**. The key themes (dark blue boxes, white font) and subthemes (light blue boxes, black font) identified

## P355

### A novel approach to collecting feedback about the intensive care setting

#### L Jackson^1^, FJ Lamb^2^, H Leaman^2^

##### ^1^Surrey and Sussex Healthcare NHS Trust, Department of Medicine, Redhill, UK, ^2^Surrey and Sussex Healthcare NHS Trust, Department of Anaesthetics, Redhill, UK

*Critical Care* 2023, **27(S1)**: P355

**Introduction:** Obtaining feedback regarding patient care in the intensive care unit (ICU) from family members is challenging. The aim of this study was to investigate if the data collected by Medical Examiners (MEs) could provide insight into relative’s ICU experiences. MEs started in England & Wales in 2020 to enable an independent senior doctor to scrutinise care quality and outcomes for in-hospital deaths [1]. MEs contact Next of Kin (NOK) for feedback about hospital care. At Surrey & Sussex Healthcare NHS Trust (SASH), this narrative feedback is recorded in a bespoke database [2].

**Methods:** Anonymised ME data from all deceased ICU patients between 1 October 2020 to 31 March 2022 were analysed. Qualitative data analysis was carried out using an inductive thematic approach. Two researchers (LJ & FJL) analysed the data, and a coding framework and themes were identified. A third researcher (HL) checked the analysis. Permission to use the data was gained from SASH.

**Results:** Analysis included data collected from NOK of 165 deceased ICU patients, including 66 (40%) grown-up children aged > 18 years and 65 (39%) spouses or partner. Of these 102 (50%) were present at moment of death. The four main themes emerged: Communication; Care and treatment; Visiting and Perceptions and understanding (Fig. 1). Across themes, communication was intrinsically linked to all aspects of a NOK’s experience but mostly with patient care. Several clinical learning points were identified including NOK’s involvement in decision making and timing and delivery of complex information.

**Conclusions:** Using ME collected data has proven a valuable source of insightful feedback. These results can strengthen staff’s knowledge of NOK’s expectations and aid learning to improve patient care.


**References**
NHS National Medical Examiners Office. 2020. https://www.england.nhs.uk/establishing-medical-examiner-system-nhs/.M Mackenzie. Bereavement board. FileMaker Server Installation and database.

**Figure Figei:**
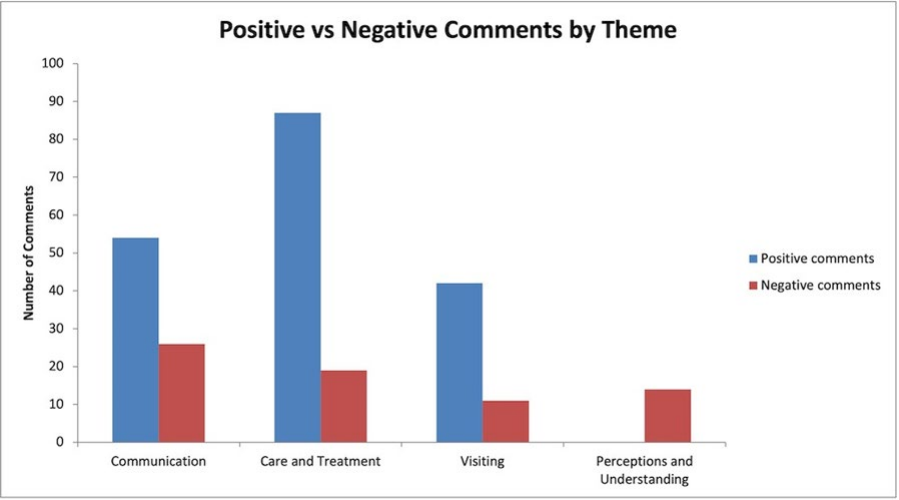
**Fig. 1 (abstract P355)**. Positive and negative comments grouped by theme

## P356

### The experience of critical care clinicians in breaking bad news to < 18 year-old relatives

#### A Molokhia^1^, G Richmond^2^, A Rochester^2^, R Breeze^2^

##### ^1^Hamad Medical Corporation, Medical Intensive Care, Doha, Qatar, Lewisham and Greenwich NHS Trust, Anaesthetics and Intensive Care, London, UK, ^2^Lewisham and Greenwich NHS Trust, London, UK

*Critical Care* 2023, **27(S1)**: P356

**Introduction:** Given that guidance, literature and training on breaking bad news to < 18-year-old relatives (< 18 YOR) in an adult ICU is very limited, we surveyed critical care (CC) physicians’ current practice and confidence levels surrounding this.

**Methods:** A Google Form investigating current practice, confidence levels, areas for development and appetite for training was disseminated to CC physicians worldwide.

**Results:** A total of 194 CC physicians (65% consultants) from 17 countries responded. Of these, 92% reported no training in breaking bad news to < 18YOR and 97% were not aware of any formal guidelines surrounding this. Only 8% felt comfortable breaking bad news to < 18YOR and 97% said that they would be interested in formal training. Roughly 25% of all UK Critical Care (CC) admissions are < 50 years of age [1] and many will have dependents < 18 years old. Despite this, 42% of physicians described varying age restrictions for visiting CC and 7% allow only adults. When breaking bad news to < 18YOR, 79% would seek prior advice from a close adult relative and 41% would like the support of a child psychologist, yet only 11% had access to one.

**Conclusions:** Our data shows there is a worldwide unmet need for greater guidance and training for physicians when communicating with < 18YOR in CC. We plan to liaise with senior CC physicians and psychology services to develop formal guidelines on breaking bad news to < 18YOR with a view to implementing interactive training sessions to improve clinician confidence.


**Reference**
Secondary Care Analysis. NHS Digital 1–28 2017. https://digital.nhs.uk/data-and-information/publications/statistical/hospital-adult-critical-care-activity/2015-16.


## P357

### Agreement between family members and physician’s view in the ICU environment; the personal experience as a factor in-fluencing the attitude towards corresponding hypothetical situations

#### P Stamou^1^, D Tsartsalis^2^, G Papathanakos^1^, E Dragioti^3^, I Andrianopoulos^1^, M Gouva^4^, V Koulouras^1^

##### ^1^University Hospital of Ioannina, Intensive Care Unit, Ioannina, Greece, ^2^” Hippokration” Hospital, Department of Emergency Medicine, Athens, Greece, ^3^University of Ioannina, Laboratory of Psychology of Patients, Families & Health Professionals, School of Health Sciences, Department of Nursing, Ioannina, Greece, ^4^ University of Ioannina, Laboratory of Psychology of Patients, Families & Health Professionals, School of Health Sciences, Department of Nursing, Ioannina, Greece

*Critical Care* 2023, **27(S1)**: P357

**Introduction:** It is not known whether intensive care unit (ICU) patients’ family members realis-tically assess patient's health status. The aim was to investigate agreement between family and intensivists’ assessment concerning patient’s evolution of health, focusing on family members’ resilience and their perceptions about decision-making.

**Methods:** For each ICU patient, withdrawal criteria were assessed by intensivists while family members assessed the development of patient's health and completed Connor-Davidson Mental Endurance- and Self-Compassion-Scale. Six months after ICU discharge, a follow-up contact was established, and family members responded about two hypothetical scenarios.

**Results:** 162 ICU patients and 189 family members were recruited. Intensivists decision about whether a patient met the withdrawal criteria had 75.9% accuracy for prediction of survival, while family’s assessments was statistically independent of intensivist’s opinion; resilience had a significant positive effect on probability of agreement with intensivists. Six months after discharge, family members whose relatives were still alive were significantly more likely to consider family or patient himself as those who should be involved in decision-making.

**Conclusions:** Resilience is related to an enhanced probability of agreement of family with intensivists’ perception of patient’s health evolution. Family attitudes in hypothetical scenarios are significantly biased on the patient's actual health evolution.

## P358

### Communicate: family-oriented communication in the intensive care unit

#### RH Kochen^1^, ASY Kraaijkamp^1^, E Pragt^1^, PWH Oyen^2^, EDJM Perree^2^, MHJ Van den Beuken-van Everdingen^2^, WNKA Van Mook^1^

##### ^1^MUMC, Intensive Care, Maastricht, Netherlands, ^2^MUMC, Palliative Care Center, Maastricht, Netherlands

*Critical Care* 2023, **27(S1)**: P358

**Introduction:** The goal of this prospective observational study was to gain more insight in the current conversation techniques and topics intensivists address during bad news conversations and whether there is room for improvement. The following hypothesis was formed: intensivists can learn from the perspective of palliative care nurses (PCN) with regard to subjects of conversation during end-of-life (EoL) conversations.

**Methods:** The goal of this prospective observational study was to gain more insight in the current conversation techniques and topics intensivists address during bad news conversations and whether there is room for improvement. The following hypothesis was formed: intensivists can learn from the perspective of palliative care nurses (PCN) with regard to subjects of conversation during end-of-life (EoL) conversations.

**Results:** The results (n = 11) are shown in Fig. 1. The feedback of the PCN was that the physicians were empathic and genuinely involved and interested in the relatives and patients; they delivered the message professionally and were clear and understanding in their communication. Points of improvement were to more frequently and in more depth clarify the family relationships, to provide more room for the relatives’ emotions, to discuss spirituality broader than from the perspective of religion only and to have the conversations in more suitable rooms. The survey among relatives (n = 9) revealed that the relatives’ valued discussing the medical context, the patient’s goals and wishes, the patient’s and relatives’ emotions and feelings, meaning of life and spirituality.

**Conclusions:** The PCN’s feedback was valuable and the communication can be improved with regard to the subjects of family relationships, spirituality and providing more room for relatives’ emotions.

**Figure Figej:**
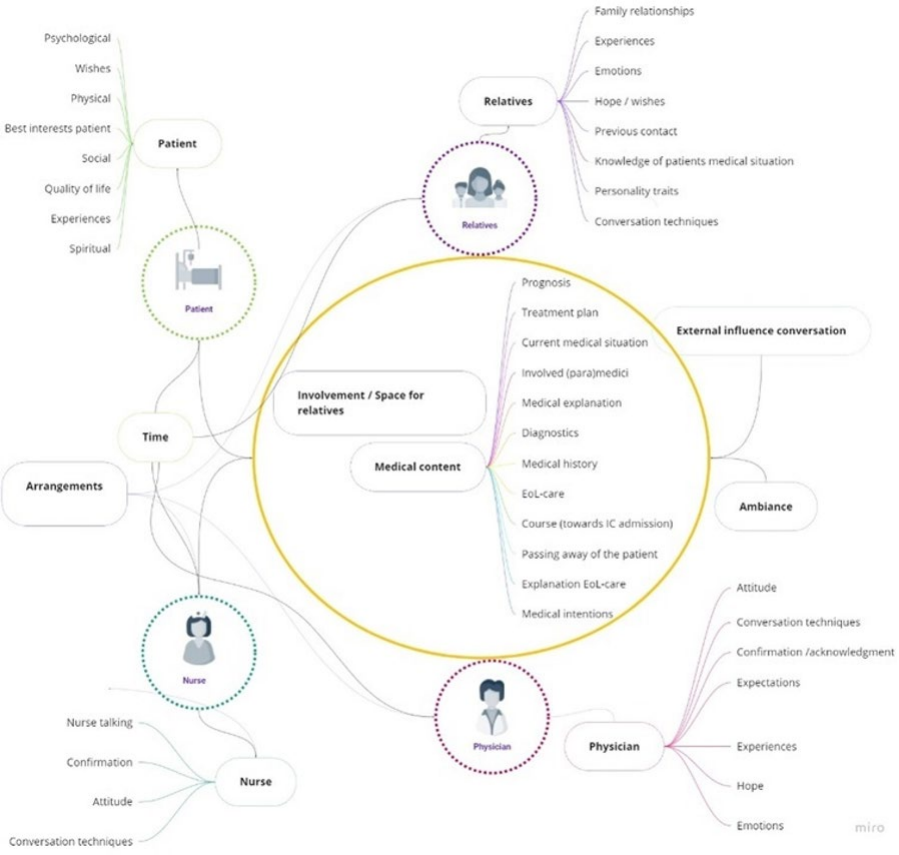
**Fig. 1 (abstract P358)**. An overview of actors involved in the EoL conversations and themes resulting from the qualitative analysis. The themes were divided into actors (physician, nurse, relatives and the patient), medical contet and other (arrangements time, ambiance and external influence conversation). The figure shows how the themes are interacted and the most important subjects in the themes

## P359

### Family satisfaction with ICU communication during the COVID-19 pandemic: a prospective multi-centre Australian study

#### M Reddy^1^, U Kadam^2^, JD Lee^3^, C Chua^4^, W Wang^5^, T McPhail^6^, J Lee^7^, N Yarwood^8^, M Majumdar^2^, A Subramaniam^1^

##### ^1^Frankston Hospital, Department of Intensive Care Medicine, Frankston, Australia, ^2^Werribee Mercy Hospital, Werribee, Department of Intensive Care Medicine, Melbourne, Australia, ^3^Monash Health Casey Hospital, Berwick, Department of Intensive Care Medicine, Melbourne, Australia, ^4^Monash University, Clayton, Faculty of Medicine Nursing and Health Sciences, Melbourne, Australia, ^5^Monash University, Clayton, School of Public Health and Preventive Medicine, Melbourne, Australia, ^6^Werribee Mercy Hospital, Werribee, Department of Social Work, Melbourne, Australia, ^7^Monash Health Casey Hospital, Berwick, Department of Social Work, Melbourne, Australia, ^8^Epworth Hospital Geelong, Waurn Ponds, Department of Intensive Care Medicine, Geelong, Australia

*Critical Care* 2023, **27(S1)**: P359

**Introduction:** Virtual communication has become common practice during the COVID-19 pandemic due to visitation restriction. We aimed to evaluate overall family satisfaction in intensive care unit (FS-ICU) with virtual communication strategies during the COVID-19 pandemic period.

**Methods:** In this prospective multi-centre study involving three metropolitan hospitals in Melbourne, Australia, the next of kin (NOK) of all the eligible ICU patients between 07/01/2022 and 10/31/2020 were required to complete an adopted version of FS-ICU 24-Questionnaire.Group comparisons were analysed for family satisfaction scores: ICU/care (satisfaction with care), FS-ICU/dm (satisfaction with information/decision-making) and FS-ICU total (overall satisfaction with the ICU) were calculated. The essential predictors that influence family satisfaction were identified using quantitative and qualitative analyses.

**Results:** Seventy-three out of the 227 patients' NOK who initially agreed, completed the FS-ICU questionnaire (response rate 32.2%). The mean (SD) FS-ICU/total was 63.9 (30.8). The mean score for satisfaction with FS-ICU/dm was lower than the FS-ICU/care (62.1 [30.30 vs. 65.4 [31.4]; *p* 0.001) (Fig. 1). There was no difference in mean FS-ICU/total scores between survivors (n = 65; 89%) and non-survivors (n = 8, 11%). Higher patient APACHE-III score, female NOK and the patient dying in ICU were independent predictors for FS-ICU/total score while a telephone call at least once a day by an ICU doctor was related to higher family satisfaction for FS-ICU/dm.

**Conclusions:** There was low overall family satisfaction with ICU care and virtual communication strategies adopted during the COVID-19 pandemic. Effort should be targeted for improving factors with virtual communication that cause low family satisfaction during the COVID-19 pandemic.

**Figure Figek:**
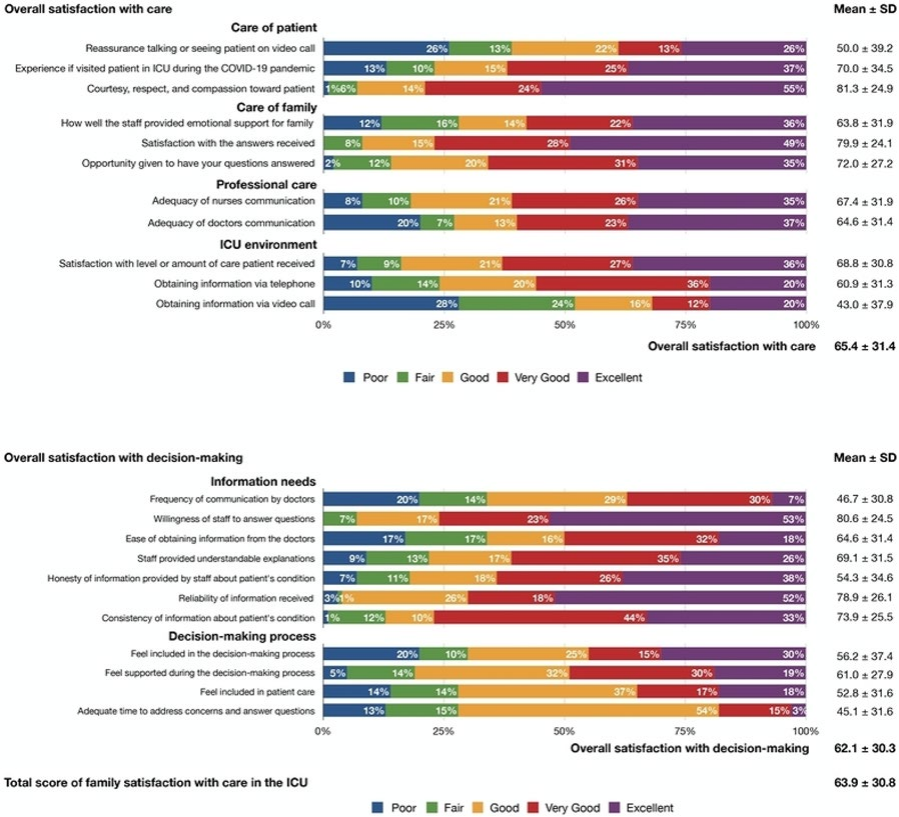
**Fig. 1 (abstract P359)**. Scores for family satisfaction with care (FS-ICU/care) and decision-making (FS-ICU/dm)

## P360

### Family satisfaction with care in the ICU

#### RI Ilan^1^, HK Khalilia^2^

##### ^1^Rambam Health Care Campus, Critical Care Medicine, Haifa, Israel, ^2^Rambam Health Care Campus, School of Medicine, Technion Institute of Technology, Haifa, Israel

*Critical Care* 2023, **27(S1)**: P360

**Introduction:** Admission to the ICU is a significant source of stress for patients' families, and may induce anxiety, post-traumatic stress, and depression [1]. We aimed to assess the satisfaction of family members with the quality of care (QOC), communication with clinical staff, and decision-making processes at our ICU. To our knowledge such evaluation has not been performed in Israel before.

**Methods:** Prospective observational study in the general ICU at the Rambam Health Care Campus in Haifa, Israel. Family members of patients who were admitted for at least 72 h were given a previously validated questionnaire, which included 26 questions concerning QOC, communication and decision-making processes [2].

**Results:** Of 70 family members that were approached, 45 (64%) responded. Overall satisfaction with QOC was 77% (Table 1). Thirty-eight (84%) of participants were completely or very satisfied with the QOC and communication with ICU staff. No statistically significant differences were found between family members of patients who survived and those who did not. Our findings were similar to those of previously reported studies [3].

**Conclusions:** In this first of its kind study in Israel, the majority of family members of patients admitted to the ICU expressed a high level of satisfaction with the QOC, communication, and decision-making processes. However, there is place for improvement on various aspects. Further research is required to better understand the reasons for dissatisfaction regarding certain domains, and to then intervene and improve the experience of family members. The results of the present study will allow us to monitor progress over time and test the effectiveness of various interventions to improve the experience of family members in our ICU.


**References**
Schaefer KG et al. Curr Opin Crit Care 2009;6:569–77.Wall RJ et al. Crit Care Med 2007;35:271–279.Heyland DK et al. Crit Care Med 2002;30:1413–8.

**Table Tabea:** **Table 1 (abstract P360)**: Satisfaction with care and decision making process

	Satisfaction with care	Satisfaction with decision making process	Global satisfaction
Completely satisfied, n (%)	26 (57)	18 (40)	27 (60)
Very satisfied, n (%)	11 (24)	18 (40)	11 (24)
Mostly satisfied, n (%)	5 (11)	3 (7)	7 (15)
Slightly dissatisfied, n (%)	2 (4)	5 (11)	0 (0)
Very dissatisfied, n (%)	1 (2)	1 (2)	0 (0)
Overall satisfaction, %	80	73	77

## P361

### Quantifying caregiver workload during the COVID-19 pandemic using ambient intelligence

#### N Choudhury^1^, A Mizrahi^1^, HA Clark^2^, T Morris^3^, L Boyd^3^, P Chan^1^

##### ^1^Eastern Health, Intensive Care Department, Box Hill, Australia, ^2^Black.AI, Melbourne, Australia, ^3^Eastern Health, Box Hill, Australia

*Critical Care* 2023, **27(S1)**: P361

**Introduction:** The COVID-19 pandemic has increased caregiver workload [1]. It is unclear how this workload is distributed across patients with varying presentations. Ambient intelligence (AmI) utilizes neural networks to monitor multiple data points in video feeds, and automatically tracks various aspects of human movement [2]. AmI was used to examine the workload of healthcare staff in relation to temporal and patient characteristics on a COVID ward at a major metropolitan hospital.

**Methods:** Sensors were deployed in patient rooms on a COVID ward to detect caregiver visits at 5-min intervals. Electronic medical records were used to identify variables hypothesized to contribute to visits.

**Results:** 5514 h across 55 patients (mean age 72, range 17–98) were analyzed. The primary reason for admission was medical in 45 cases (81.8%), psychiatric in 8 cases (14.5%) and surgical in 2 cases (3.6%). Medical emergency (MET) calls occurred in 21 (38.2%) cases. As summarized in Fig. 1, visitation was lowest between 0000 and 0400 (27.3 ± 1.1 min/hour (min/hr)) and highest between 1200 and 1600 (65.5 ± 1.3 min/hr). The mean ± SE visitation spent with medical, psychiatric, and surgical patients was 51.7 ± 0.6, 38.8 ± 1.3, and 33.7 ± 3.9 min/hr respectively (*p* < 0.005). Overall lowest visitation was in surgical patients between 0400 and 0800 (4.5 ± 4.5 min/hr). Mean ± SE visitation were 66.7 ± 4.6 min/hr in the three hours preceding and following MET calls compared to 50.2 ± 0.5 min/hr in periods without MET calls (*p* < 0.005). There was no difference in visitation time between patients with respiratory symptoms and those without (50.7 ± 0.9 vs 48.8 ± 0.7 min/hr, *p* = 0.1).

**Conclusions:** AmI can help quantify patient workload, potentially improving staff planning. Further studies comparing healthcare attendance between patients on COVID wards and non-COVID wards may provide insight into the impact of unique factors associated with the pandemic.


**References**
Knobf MT et al. Oncol Nurs Forum. 2022;49:491–499.Chan PY et al. Aust Crit Care. 2023;36:92–98.

**Figure Figel:**
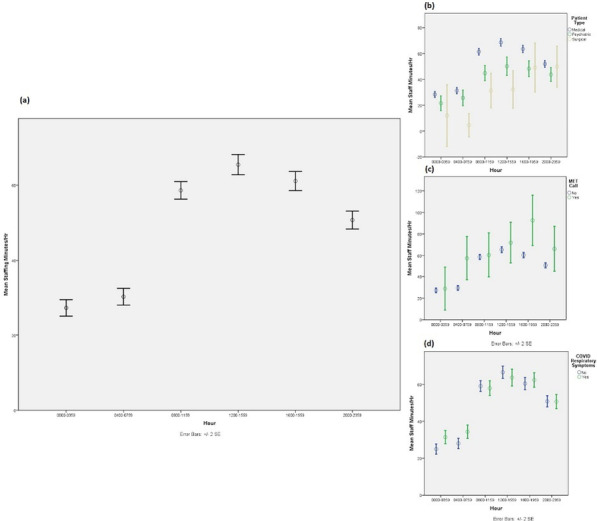
**Fig. 1 (abstract P361)**. Caregiver visitation in staffing minutes/hour with respect to time of day (**a**), and stratified by patient category—medical, psychiatric or surgical (**b**), whether or not a MET call was activated (**c**) and whether the patient had COVID-19 related respiratory symptoms (**d**)

## P362

### Impact of standard versus enhanced personal protective equipment onto team performance during simulated emergency in ICU: randomised controlled trial (PPE-TEAM trial)

#### M Renza^1^, RS Sýkora^1^, DP Peřan^2^, VZ Zvoníček^1^, PW Waldauf^1^, MS Stern^1^, FD Duška^1^

##### ^1^Charles University, Third Faculty of Medicine and FNKV University Hospital, Prague, Czech Republic, Department of Anaesthesia and Intensive Care Medicine, Prague, Czech Republic, ^2^Emergency Medical Services of the Karlovy Vary Region, Karlovy Vary, Czech Republic, Emergency Medical Services of the Karlovy Vary Region, Karlovy Vary, Czech Republic, Karlovy Vary, Czech Republic

*Critical Care* 2023, **27(S1)**: P362

**Introduction:** During the recent COVID-19 pandemics, hospital staff often used personal protective equipment (PPE) beyond the standards recommended by the public health authorities. The aim of the study is to investigate the impact of the additional PPE compared to the standard PPE onto team performance when dealing with a medical emergency.

**Methods:** Single centre, prospective, randomized, controlled, open-label, study compared 22 teams, consisting of a doctor and 2 nurses, randomized in a 1:1 ratio to use standard PPE or Super-Safe Setup in a simulated medical crisis. Before randomisation, all teams underwent a standardised mock scenario without the use of PPE was used to assess the performance at baseline. The primary outcome was Team Emergency Assessment Measure (TEAM), secondary outcomes included the timing and quality parameters of CPR and physiological changes the rescuers.

**Results:** As compared to standard PPE recommended by WHO [1], the use of additional PPE that included hooded coverall, significantly reduced non-technical skills of teams (TEAM score 30.6 ± 5.7 vs. 36.2 ± 5.0, *p* = 0.015) (Table 1), mainly due to reducing performance in complex tasks such as planning of future actions, prioritisation, and following the guidelines. This was also reflected in participants self-assessment score (Table 1). Nonetheless, there was no significant difference in the quality parameters and timing of milestones during a simulated CPR. There were no differences in rescuers physiological responses.

**Conclusions:** During a simulated crisis, extended PPE compared to the PPE recommended by the World Health Organisation degraded non-technical team skills. This did not translate into measurable deterioration of patient-centred outcomes, such as quality or timing of CPR.


**Reference**
World Health Organization. Technical specifications of personal protective equipment for COVID-19. *Interim Guidance*.

**Table Tabeg:** **Table 1 (abstract P362)**. Primary Outcomes—TEAM Score

TEAM item	WHO	SSS	*p*
Q1–Q11 TEAM score			NS
Q12—global score	7.7 ± 1.1	6.5 ± 1.4	0.022
Total TEAM score	36.2 ± 5.0	30.6 ± 5.6	0.015
Self-assessment score	7.9 ± 1.0	7.7 ± 1.3	0.173

## P363

### Real-time remote audio-visual system versus standard buddy system to monitor the doffing of personal protective equipment during COVID-19 pandemic: an observational study

#### BN Naik, MS Lazar, V Ganesh, A Singh, GD Puri

##### PGIMER, Department of Anaesthesia and Intensive Care, Chandigarh, India

*Critical Care* 2023, **27(S1)**: P363

**Introduction:** Literature states a higher self-contamination rate among healthcare workers (HCWs) while doffing personal protective equipment (PPE). During the COVID-19 pandemic, onsite trained observers were not always available to monitor PPE compliance. The remote audio-visual doffing surveillance (RADS) system has the potential to overcome this limitation [1, 2]. We aimed to compare the efficacy of this real-time RADS system against the onsite buddy system for monitoring the doffing of PPE.

**Methods:** This prospective, observational study was conducted at our tertiary care centre in northern India. Study was registered in the clinical trial registry India (CTRI/2020/11/038172). 200 HCWs who cared for COVID-19 patients in the intensive care units were included. Group A included HCWs who performed doffing with the help of an onsite trained observer and group B included HCWs who performed doffing with the RADS system. An independent observer noted the error at any step using the CDC doffing checklist, in both groups. An online questionnaire to analyse the level of satisfaction post-doffing was also surveyed.

**Results:** The proportion of errors committed during doffing was significantly lower in group B compared to group A with a low relative risk of 0.34 (95% CI 0.22–0.51) (*p* < 0.001) (Fig. 1). In both groups, there was no difference in HCWs feedback regarding the ease of the system and fear of committing an error. Though the perceived quality of monitoring was felt better with onsite buddy, the overall confidence rating of being safe after doffing was better with the RADS system.

**Conclusions:** Real-time RADS system may be more effective than the onsite buddy system for ensuring the safety of HCWs during doffing PPE. HCWs level of satisfaction related to the ease and anxiety with the monitoring systems were comparable. RADS system can reduce reliance on HCW resources and can integrate well into existing healthcare systems.


**References**
Singh A et al. Anesth Analg. 2020;131:e112–3.Naik BN et al. Cureus 2021;13:e18071.

**Figure Figem:**
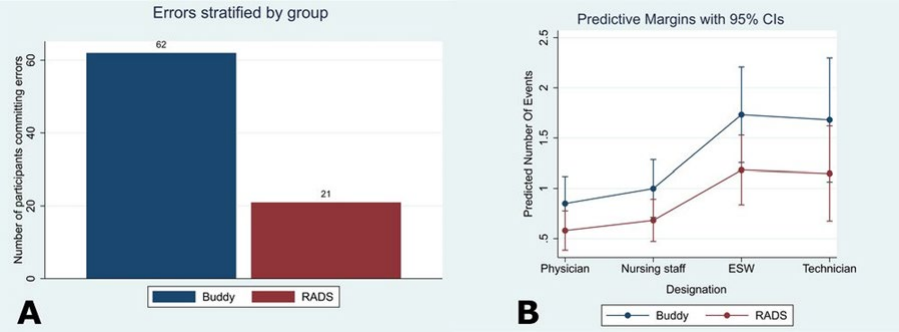
**Fig. 1 (abstract P363)**. **A** Barplot showing the number of participants committing errors in each group; **B** Point plot showing the predicted margins (incidence rate ratios, with 95% CIs) of committing a greater number of errors/events across occupation designation classes during doffing stratified by experimental groups. (ESW = Environmental Sanitation Workers, RADS = Remote Audio-visual Doffing Surveillance System)

## P364

### Diurnal patterns of noise levels observed in the Whittington Hospital intensive care unit

#### NP Ivin^1^, H Montgomery^2^, S Weerakkody^2^

##### ^1^Princess Royal University Hospital, Anaesthetics Department, London, UK, ^2^University College London, UCL Centre for Human Health and Performance, London, UK

*Critical Care* 2023, **27(S1)**: P364

**Introduction:** Delirium and sleep disturbance have deleterious physiological and psychological effects in the critically ill, with associated adverse outcomes including longer length of stay and increased mortality [1]. A contributing factor is the ICU environment; in particular the noise which patients experience. We aimed to describe the characteristics of sound pressure levels (SPLs) that patients and staff are exposed to in an 11 bed bay of a district general hospital in order to target noise limiting interventions.

**Methods:** SPLs (LA_eq_ 1 min and 1 h; A-weighted continuous equivalent SPL, LAF_max_; A-weighted fast constant maximum SPL, and L_peak_; peak SPL) were monitored continuously over a 116 day period using a SoundEar3 (NoiseMeters Ltd) noise activated warning device. Data were extracted and diurnal median and interquartile LA_eq_ values were plotted. Diurnal rates of LAF_max_ values above different thresholds were plotted. Median numbers of extreme L_peak_ values per 24 h period were calculated.

**Results:** Overall median LA_eq_ was 65.3 dB (interquartile range 62.6–67.5 dB). LA_eq_ 1 min and 1 h plots showed diurnal variation consistent with the timings of key events such as ward rounds and handovers, with daytime LA_eq_ values observed to be lower at the weekend (Fig. 1). Although LAF_max_ values were much lower at night, at trough levels 5 min per hour had LAF_max_ > 80 dB. Mean numbers of L_peak_ values per 24 h period were 0.67 > 120 dB, 3.15 > 115 dB, 12.0 > 110 dB, and 45.7 > 105 dB.

**Conclusions:** Our results show a wide diurnal variation in both the ambient and peak noise levels that patients and staff are exposed to, similar to that observed in other centres. Our results show higher noise levels than those measured in comparable centres and higher levels than those that have been recorded in smaller bays, however direct comparisons are difficult due to technical aspects of acoustics.


**Reference**
Salluh JIF et al. BMJ 2015;350:h2538.

**Figure Figen:**
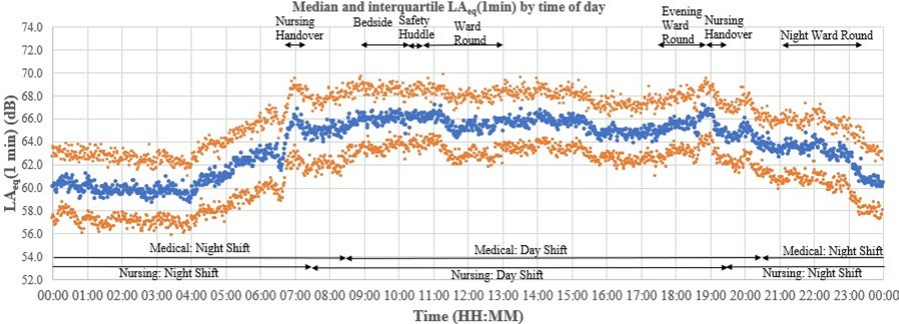
**Fig. 1 (abstract P364)**: Median and interquartile Laeq (1 min) values by time of day. Arrows and annotations indicate the timing and duration of key events each day, and timing of day and night shifts for medical and nursing teams

## P365

### A systematic review investigating what factors influence clinical decision-making for patients in critical care units regarding the initiation, delivery or titration of organ support

#### K Matsumoto, H Malcolm, W Thomson, J Prowle, Z Puthucheary, T Stephens

##### Critical Care and Perioperative Medicine Research Group, William Harvey Research Group, Queen Mary University of London, London, UK

*Critical Care* 2023, **27(S1)**: P365

**Introduction:** The critical care unit is a dynamic environment that necessitates daily clinical decision-making. Despite this, the processes and influences on decision-making have been poorly defined. Our aim was to synthesise existing research to identify factors influencing clinicians’ decisions surrounding organ support.

**Methods:** We conducted a systematic search on three databases (PubMed, Embase and CINAHL) to find relevant papers exploring human factors that influenced decisions made by critical care clinicians. A meta-ethnography was then completed on included papers. The data were collated into a common format and cross-compared. This generated distinct themes/subthemes that were synthesised to develop a higher order interpretation.

**Results:** 33 studies (from 8647 citations) met the inclusion criteria. 21 of these only included nurses, 7 only doctors and 5 were interprofessional. 11 factors that influenced a clinician’s decision-making were identified: experience; professional and personal risk; uncertainty; characteristics of individuals; senior support; team hierarchy; decision making by colleagues; protocols, guidelines and evidence; time and workload; hospital structure; and clinical condition. These were grouped into four themes: human, team, system and patient factors. From our interpretation of the data, we developed a model of how these themes interact to influence clinicians’ decision-making (Fig. 1).

**Conclusions:** While decision-making surrounding organ support is complex and dynamic we identified recurring themes that influenced these decisions across different professions and environments. Further research to improve individual decision-making could be structured around addressing these themes.

**Figure Figeo:**
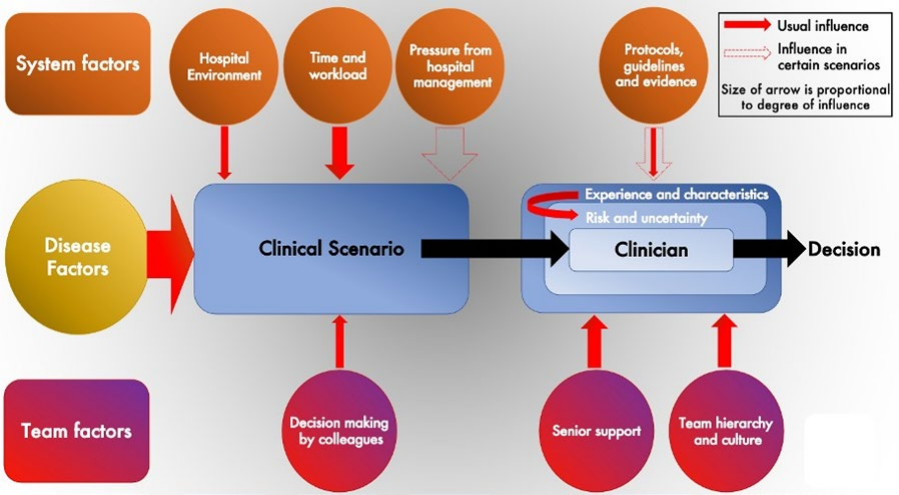
**Fig. 1 (abstract P365)**. Model for clinical decision making. A clinician interprets a clinical scenario and makes a decision. However there are multiple factors that influence either the clinical scenario or the clinician, and in turn the final decision

## P366

### Critical care unit-to-unit capacity transfers have an increased risk of dying compared to non-transferred patients

#### F Parenmark^1^, M Fredrikson^2^, SM Walther^3^

##### ^1^Centre for Research and Development, Uppsala University, Region Gävleborg, Dept. Anaesthesia & Intensive Care, Gävle, Sweden, ^2^Department of Biomedical and Clinical Sciences, Faculty of Medicine, Linköping University, Linköping, Sweden, ^3^Department of Cardiothoracic Anaesthesia and Intensive Care, Linköping University Hospital, Linköping, Sweden

*Critical Care* 2023, **27(S1)**: P366

**Introduction:** To preserve access to the intensive care unit (ICU)—even in times of a shortage of critical care beds—ICUs adopt temporary measures to make room for new admissions. One measure is to transfer a patient to another ICU; a so-called ‘capacity transfer’. Understanding the impact of capacity transfers on patient outcomes is of utmost importance since these transfers are not carried for the benefit of the patient. The purpose of the current study was to examine the idea that capacity transfer is associated with increased mortality compared to non-transferred patients who either died in the ICU or were discharged alive to a regular hospital ward.

**Methods:** In this multicentre, case-controlled, matched observational cohort study, we collected data between January 2016 and December 2019 from 68 ICUs. Capacity transferred patients were matched 1:10 using controls from the same ICU, with a stay as long or longer to avoid immortal time bias in the non-exposed population. We compared adjusted 30- and 90-day mortality rates in patients exposed to capacity transfer with non-transferred patients using potential outcomes modelling and mixed effects logistic regression modelling with propensity score adjustment.

**Results:** 95,909 individuals from 68 ICUs were eligible for inclusion. The main analysis group included 13,670 patients of which 1243 had been transferred due to capacity reasons. Capacity transfer was associated with a risk increase in 30-day mortality of 4.7% (95% CI 2.0–7.4; *p* = 0.001) compared to non-transferred patients when analysed using a potential outcomes framework (Table 1). The average risk increase in 90-day mortality was 4.9% (95% CI 2.1–7.8: *p* = 0.001).

**Conclusions:** This study demonstrates that patients undergoing capacity transfers are exposed to an increased mortality risk with about one additional death for every 20 transferred patients, which was neither caused by illness severity on admission nor by additional factors in the referring ICU.

**Table Tabeh:** **Table 1 (abstract P366)**. Relationships between capacity transfer and mortality risk 30-, and 90 days after transfer

	Mortality 30 days	Mortality 30 days	Mortality 90 days	Mortality 90 days
	Absolute risk difference (95% CI)	Odds ratio (95% CI)	Absolute risk difference (95% CI)	Odds ratio (95% CI)
Model				
Average effect on transferred patient	4.7 (2.0–7.4)	1.30 (1.23–1.38)	4.9 (2.1–7.8)	1.29 (1.21–1.35)
Mixed effects multivariable model	NA	1.66 (1.39–1.98)	NA	1.47 (1.25–1.73)
Propensity score adjusted mixed effects multivariariable model	NA	1.38 (1.18–1.61)	NA	1.32 (1.14–1.54)

## P367

### Impact of the pandemic on critical care staff safety climate assessed using safety attitudes questionnaire (SAQ) in all hospitals within the East of England

#### M Blunt^1^, P Young^1^, M Hijjeh^2^, K Cotton^3^, M Wright^3^

##### ^1^Queen Elizabeth Hospital, Critical Care Centre, King’s Lynn, UK, ^2^Mid and South Essex NHS FT, Critical Care, Basildon, UK, ^3^NHE East of England, East of England Critical Care Network, Cambridge, UK

*Critical Care* 2023, **27(S1)**: P367

**Introduction:** As part of the last two peer review processes the East of England Critical Care Network have undertaken SAQ [1] involving staff in the 18 hospitals in the region. The questionnaire is divided into 7 scales and allows a wide assessment of safety culture which has been associated with improved outcomes and reduced adverse incidents [2]. The two surveys straddle the Covid-19 pandemic which has had a recognised impact on staff welfare. We postulated that this is likely to be identifiable within the survey and wished to understand the effect on different staff groups and levels of experience.

**Methods:** The survey was distributed using an online platform and analysed in R.

**Results:** 1647 questionnaires were completed in 2019 (753) and 2022 (894). Demographic data was collected for role (nursing, medical, AHP, support) and critical care experience (short: < 4 years medium: 4–10 years, long: > 10 years). For all staff Climate scores fell significantly in all scales except stress recognition, most noticeably in job satisfaction and working conditions. This was also the case within the nursing subgroup; however, it was not seen in medical staff (though there was a less pronounced trend). AHPs reported a marked reduction in job satisfaction. Support staff also demonstrated big reductions in all scales including teamworking and safety climate. The fall in job satisfaction and working condition scales were also most profound in staff with 4–10 year’s critical care experience.

**Conclusions:** The pressures of the pandemic have had a lasting effect on most staff groups, particularly in job satisfaction and working conditions. This appears to have particularly impacted nursing and support staff. Those of moderate experience may have been the staff most directly involved with increased patient care and expectations to undertake managerial roles with reduced training. The impact of this appears to be ongoing despite efforts to support staff well-being.


**References**
Sexton J. BMC Health Serv Res 2006;6:44–54.Colla JB. Qual Saf Health Care 2005;14:364–6.


## P368

### Use of a shirt front mounted video camera (vest-cam) to monitor hand hygiene

#### E Ben-chetrit^1^, D Belman^2^, PD Levin^2^

##### ^1^Shaare Zedek Medical Center, Infectious Diseases, Jerusalem, Israel, ^2^Shaare Zedek Medical Center, Intensive Care Unit, Jerusalem, Israel

*Critical Care* 2023, **27(S1)**: P368

**Introduction:** Hand hygiene observations are usually made by an observer during "office hours". Hand hygiene compliance at night/weekends might not be comparable. Using a vest-cam (a camera attached to the shirt front) potentially allows hand hygiene compliance to be monitored at all hours of the day by retrospective analysis of the video footage. In this pilot study we present a comparison of hand hygiene observations made by an observer, versus analysis of vest-cam video taken in parallel.

**Methods:** Following ethics approval, 20 min observation periods were performed on ICU staff wearing a vest-cam. A human observer recorded hand hygiene opportunities (entrance and exit from the patient areas), and hand hygiene performance. The vest-cam video was analyzed by an independent observer according to the same parameters for hand hygiene opportunities and performance. The two records were then compared.

**Results:** Twelve observation periods were analyzed. One was excluded due to camera misplacement that resulted in the caregiver's hands not being visible. Hand hygiene performance was identified by the vest cam on 68/81 (84%) occasions versus 58/81 (72%) by the observer (*p* = 0.09). Mean hand hygiene performance time was 10 ± 8 vs 11 ± 21 s (*p* = 0.74). Not all hand hygiene performance was associated with defined hand hygiene opportunities. Hand hygiene events were identified on 82 occasions. All events were identified from the vest-cam (82/82, 100%), while (67/82, 82%) were identified by the observer (*p* < 0.001). Calculated hand hygiene compliance was similar: vest cam vs observer 51/82 (62%) versus 43/67 (64%, *p* = ns).

**Conclusions:** Analysis of video footageobtained from a shirt front mounted video camera was able to identify hand hygiene opportunities and performance at least as well as an observer. While this technique may be associated with ethical dilemmas, potentially it allows monitoring of hand hygiene compliance outside usual office hours.

## P369

### Exploring intensive care unit (ICU) consultations with a focus on treatment limitations: a critical incident analysis

#### ASY Kraaijkamp^1^, RH Kochen^2^, BPA Spaetgens^3^, L Schultinge^2^, CMH Pinxt^2^, MHJ Van den Beuken-van Everdingen^4^, WNKA Van Mook^2^

##### ^1^Maastricht University Hospital, Intensive Care Department, Palliative Care Department, Maastricht, Netherlands, ^2^Maastricht University Hospital, Intensive Care Department, Maastricht, Netherlands, ^3^Maastricht University Hospital, Geriatric Care department, Maastricht, Netherlands, ^4^Maastricht University Hospital, Palliative Care Department, Maastricht, Netherlands

*Critical Care* 2023, **27(S1)**: P369

**Introduction:** The ICU is frequently consulted when a (frail) patient critically deteriorates on a general ward. However, considering patient-tailored care, this potentially leads to the escalation of care disproportionately to the patient's preferences, pre-existing quality of life, and functioning. Alternatively, other medical specialists, such as the palliative team, can be consulted to discuss less-invasive, comfort-based treatment options. The aim was to gain better insight into the underlying motives behind ICU consultations on general wards with a focus on (prior) documented treatment limitations (such as do-not-resuscitate, do-not-intubate, comfort-only preferences) or for whom intensivists deem ICU treatment too invasive. Concomitantly, to explore the potential role of an earlier involvement of the palliative team in these consultations.

**Methods:** A qualitative study using a critical incident technique and thematic analysis.

**Results:** In total, 36 individual interviews were conducted between April and July 2022, with 18 ICU nurses and intensivists(in-training) consulted by general wards of a tertiary academic centre. The palliative team was involved in 6% at the time of consultation, 84% of the patients were estimated to have a life expectancy of fewer than 12 months, and 58% died within 2 months. The themes frequently discussed were awareness of the burdens of ICU treatment, establishing conformity about supervision, and timely communication about treatment limitations. Participants acknowledged the underutilization of the palliative care team due to invisibility (outside office hours) but saw a potentially valuable role in the earlier consultation of this team.

**Conclusions:** Although intensivists are watchful for inordinate invasive care and share responsibility for counselling or discussing treatment limitations, earlier consultation of potential other actors such as the palliative team might contribute to more patient-tailored care and more efficient use of ICU resources.

## P370

### TEPology: reconsidering treatment escalation planning on ICU

#### E Harlow, W Woodward

##### Royal Cornwall Hospital, Critical Care, Cornwall, UK

*Critical Care* 2023, **27(S1)**: P370

**Introduction:** Treatment escalation planning (TEP) is a dynamic and nuanced process, but for effective communication and handover is often summarised into a binary decision of whether or not to attempt resuscitation. Do Not Attempt Resuscitation (DNAR) status is recognised in 22 European countries [1] and in the UK, where a TEP proforma is frequently used in patient notes. The UK Guidelines for the Provision of Intensive Care Services (GPICS) states that all patients should have a treatment escalation plan documented [2], but provides little further guidance as to when it should be completed or what form it should take.

**Methods:** We audited TEP proforma completion on a general ICU in Cornwall, UK, and compared results to patient records that included either a proforma or narrative indices of treatment escalation planning.

**Results:** 21% of the patient sample had a TEP proforma. 30% had either a TEP proforma or narrative indices of a treatment escalation plan.

**Conclusions:** Documentation of TEP completion in this patient sample is poor and does not meet GPICS standards. Whilst narrative indices recorded in the Consultant notes were more common than TEP proformas, and likely better represent the complexity of TEP decisions while on ICU, the use of standardised proformas at time of ICU discharge contributes to more effective handover, which is crucial for ongoing care and reduction of readmission rates. Use of the proforma should serve to prompt discussion within the MDT, and with patients and their relatives to inform shared decision making. We plan to make TEP proformas mandatory at time of discharge, and reaudit in 3 months. We also anticipate the soon to be published European Society of Intensive Care Medicine’s Guidelines on End of Life care in ICU for further guidance on this important topic.


**References**
The Faculty of Intensive Care Medicine. *GPICSv2*. https://www.ficm.ac.uk/.European Resuscitation Council. https://ercguidelines.elsevierresource.com.


## P371

### Hospital mortality after discharge from intensive care – the reality of a tertiary center

#### JE Mateus, J Rua, AR Nogueira, E Louro, G Almeida, M Simões, P Martins

##### Centro Hospitalar e Universitário de Coimbra, Intensive Care Unit, Coimbra, Portugal

*Critical Care* 2023, **27(S1)**: P371

**Introduction****:** The discharge of patients from the intensive care unit (ICU) to a ward is a high risk transition of care [1]. The aim of this study was to identify the incidence and characteristics of patients who died in general wards after discharge from ICU.

**Methods:** Retrospective study of all patients discharged from a tertiary center ICU from April 2021 to October 2022. Demographic data, severity scores and hospital mortality were evaluated. Statistical analysis was performed by χ^2^ and t Student tests.

**Results:** In this period, 715 out of 962 patients were discharged from our ICU and 55 (8%) died in the wards: 33% in the first 5 days, 18% from 6th to 10th day and 49% from 11th day on. Cause of death was: sepsis (47%), irreversible neurological injury (17%), cardiovascular disease (12%) and tracheostomy obstruction (10%). Comparing to the patients who were discharged home alive, the patients who died in the wards were older (average age 66.6 vs 58.0 years), had shorter ICU length of stay (mean 15.5 vs 23.4 days), had a higher percentage of tracheostomies (34.5 vs 14.2%), higher Charlson Comorbidity Index (3.6 vs 2.7), higher APACHE II (23.9 vs 18.9) and SAPS-II (52.8 vs 43.3) at ICU admission, higher SOFA at ICU admission (8.9 vs 7.1) and discharge (4.8 vs 2.9) and higher standardized mortality (53% vs 37%). There were no statistically significant differences concerning cause of ICU admission.

**Conclusions:** ICU mortality underestimates the true mortality of critically ill patients as the mortality in the first days after ICU discharge can be substantial. Though some deaths are inevitable, other are unexpected and might have been prevented by discharge to high dependence units (lacking in our hospital). In our study, deaths occurred in patients who were more likely to die: older patients with more comorbidities and higher severity scores.


**Reference**
Moreira HE et al. Rev Bras Ter Intensiva 2017;29:63–9.


## P372

### Managing the end-of-life transition in the ICU

#### K Williams, H Omara, K Russell, M Fathy Elkhonezy, V Metaxa

##### Kings College Hospital, Intensive Care, London, UK

*Critical Care* 2023, **27(S1)**: P372

**Introduction:** Approximately 15–20% of ICU patients will die during their hospital admission, frequently after limitation of treatment. Quality of end-of-life (EoL) care focuses on patient/family support, symptom management, minimising distress, and withdrawing/withholding inappropriate treatment [1]. Following withdrawal of life-sustaining therapy, death can be rapid and potentially distressing, hence a smooth transition is paramount with good communication and adequate symptom control [2].

**Methods:** Service evaluation of EoL practices in a large, tertiary London hospital. Data collected from 1/5 to 16/6/22 on ICU patients transitioning to EoL care.

**Results:** 43 EoL patients (Table 1). Treatment limitations were placed for 27 (44%), prior to EoL transition. Treatment withdrawal in 30 (70%) patients: 12 (28%) palliative extubated, 7 (16%) remained intubated but other support withdrawn. 7 (16%) remained on full support. EoL medications were delivered as an infusion in 26 (60%) patients in 24 h before death: 8 (19%) subcutaneously and 18 (42%) intravenously. 8 (19%) remained on high doses with ongoing support, 16 (37%) had reduced infusion rates for comfort only. The most common infused drugs were fentanyl (50–400 mcg/hr), midazolam (20 mg/24 h-10 mg/hr), morphine (15–150 mg/24 h) and glycopyrronium (600–1200 mcg/24 h). Sedation was deep prior to death (RASS-3.8, 24 h and -3.6, 48 h). Reasons for low RASS were severe brain injury, continued full support and agitation. Mean time from EoL transition to death was 1.25 days.

**Conclusions:** Death in ICU can be challenging, due to the highly technical environment, co-existence of multiple organ failure and inability to communicate with the patient. Despite national guidance, the practicalities of transitioning from curative to EoL care vary, even in a single institution. The more distinct variability was observed around the use of EoL medication, with doses exceeding those used in palliative care settings.


**References**
Faculty of Intensive Care Medicine. Care at the end of life, 2019.Efstathiou N et al. Palliat Med 2020;34:1140–1164.

**Table Tabel:** **Table 1 (abstract P372)**. Patient characteristics

Variables (N = 43)	n (%)
Age (mean—years)	65
Sex (female)	16 (37)
Neurosciences, medicine, cardiology, T&O, other	20 (46), 7 (16), 6 (14), 4 (9), 6 (14)
Ref. to external teams: pall care, psychosocial	18 (42), 14 (33)
Documentation of discussion: patient, family	5 (11), 41 (95)
Presence of an advance directive	8 (19)
Died: ICU, ward	34 (79), 9 (21)

## P373

### Implementation of the Triggers tool at the critical care unit at the Royal Marsden Hospital

#### J Gandhi^1^, N Kapoor^1^, P Srivastava^1^, H May-Miller^1^, A Halley^2^, J Droney^2^, P Gruber^3^, A Tweedle^2^

##### ^1^The Royal Marsden Hospital, Palliative/Critical Care, London, UK, ^2^The Royal Marsden Hospital, Palliative Care, London, UK, ^3^The Royal Marsden Hospital, Critical Care, London, UK

*Critical Care* 2023, **27(S1)**: P373

**Introduction:** Mortality rates for cancer patients admitted to Critical Care Units (CCU) are improving but for certain subgroups remain high. It is recognised that early palliative care input improves symptom control, family support, length of CCU stay and the use of clinical interventions towards the end of life. Triggers is a tool to help identify patients that may benefit from early palliative care intervention [1]. The tool was adapted by the Royal Marsden Hospital to identify emergency admissions onto CCU that may benefit from early palliative care review.

**Methods:** A local, multi-cycle retrospective review was conducted with data collected through the electronic patient record system. We collected three data sets—one prior to and two (short and long term) following the implementation of the Triggers tool.

**Results:** Prior to implementation of the tool, data collected between April 2018–April 2019 showed that 149 patients died on CCU or within 30 days of admission. Of these patients, only 56% were referred to the symptom control and palliative care team (SCPCT) however 97% of these patients would have flagged as Triggers positive. Data between February 2020 and January 2021, after implementation of the Triggers tool, showed that 36% of CCU emergency admissions had a Triggers tool completed. Figure 1 shows the results of this sample. 44% of emergency admissions during this time would have flagged as Triggers positive. Overall, 23% of emergency admissions were referred to the SCPCT, of which 46% of these were due to the tool. Data from January to September 2022, showed a Triggers completion rate of 33%. 100% of emergency admissions would have flagged as Triggers positive. Overall, 33% of emergency admissions were referred to SCPCT due to the tool.

**Conclusions:** The use of this novel bespoke Triggers tool is now a standard part of the admission process to CCU. This complex intervention requires ongoing training and education to ensure continued compliance.


**Reference**
Nadkarni Y et al. Support Care Cancer 2022;30:2173-2181.

**Figure Figep:**
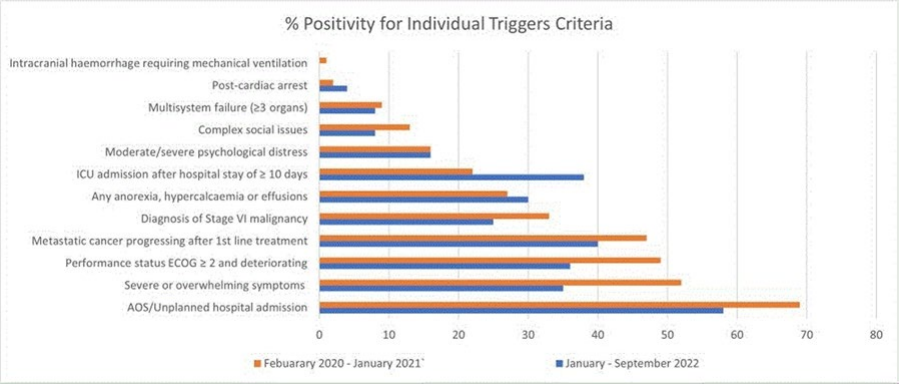
**Fig. 1 (abstract P373)**. Percentage positivity of individual Triggers criteria

## P374

### Are doctors ready to accept Bayesian adaptive trials? The results of a randomized study of intensivists

#### BE Malley^1^ , J Levin^2^ , A Althouse^3^ , L Bukowski^1^, J Kahn^1^, DT Huang^1^

##### ^1^University of Pittsburgh, Department of Critical Care Medicine, Pittsburgh, USA, ^2^University of Pittsburgh, Organizational Behavior Program, Pittsburgh, USA, ^3^University of Pittsburgh, Division of General Internal Medicine, Pittsburgh, USA

*Critical Care* 2023, **27(S1)**: P374

**Introduction:** We evaluated intensivists’ acceptance and understanding of Bayesian adaptive trials and explored the association between acceptance and understanding. Randomized clinical trials (RCTs) are a mainstay of medical research but have multiple drawbacks including cost and inefficiency. Bayesian adaptive trials may address these drawbacks, but are more complex and potentially less accepted by the medical community.

**Methods:** We surveyed United States intensivists identified from an American Medical Association email list. Participants were randomized to view an abstract describing a hypothetical Bayesian adaptive or traditional frequentist trial. The abstracts were otherwise similar in format and content with results derived from the same simulated data. Participants were then asked about self-perceived and measured understanding as well as acceptance of the trial findings. Survey results were analyzed using proportional odds logistic regression.

**Results:** There were 273 responses to 592 surveys (46.1%) and the groups were well balanced. The frequentist group had significantly better self-perceived (*p *< 0.01 and *p *< 0.01) and measured understanding (*p *< 0.01, *p* = 0.08, *p *< 0.01, and *p* = 0.04). There was no significant difference in acceptance between groups (*p* = 0.47 and *p* = 0.10). Acceptance was significantly correlated with both self-perceived and measured understanding (Table 1). These results did not change substantially when adjusting for age, practice setting, clinical time, and subspecialty. Opinions on the increasing use of Bayesian methods were mixed: 49.5% positive, 39.2% neutral or undecided, and 11.4% negative.

**Conclusions:** Despite lower understanding of Bayesian adaptive trials compared to frequentist trials among intensivists we found no difference in acceptance of results. Understanding and acceptance were correlated and a high proportion of intensivists were neutral or undecided about Bayesian adaptive trials so our findings suggest further efforts to educate clinicians on these methods are warranted.

**Table Tabdu:** **Table 1 (abstract P374)**. Physician acceptance of Bayesian adaptive trials is associated with level of understanding

Understanding question category	Question	Association with acceptance Question 1 OR (95% CI)	Association with acceptance Question 2 OR (95% CI)
Self-perceived	Question 3	5.08 (1.88–13.69)	3.38 (1.39- 8.19)
Self-perceived	Question 4	3.74 (1.29–10.85)	6.49 (2.15–19.61)
Measured	Question 5	2.67 (1.08–6.62)	2.56 (1.09–6.02)
Measured	Question 6	3.10 (1.32–7.24)	2.88 (1.36–6.11)
Measured	Question 7	1.54 (0.77–3.10)*	1.72 (0.88–3.36)*
Measured	Question 8	3.61 (1.60–8.15)	3.50 (1.63–7.52)

Odds ratios are of an acceptance response one Likert level higher for a positive/correct understanding question response. All results are significant at an alpha level of 0.05 except those with a *

## P375

### Community intensive care unit recruitment to improve research efficiency

#### N Quigley, SM Bagshaw, VI Lau

##### University of Alberta, Department of Critical Care, Edmonton, Canada

*Critical Care* 2023, **27(S1)**: P375

**Introduction:** In Canada, critical care research has traditionally been conducted in academic hospital intensive care units (ICUs). Increasing community ICUs participation (which represents two-thirds of ICU capacity in Canada) in multi-center trials may enhance recruitment, shorten time to completion and improve generalizability and quality of evidence. We aimed to identify community ICU patients that could be screened for and enrolled in large multi-center trials and quantify the potential opportunity associated with greater community ICU participation.

**Methods:** We first chose 10 landmark critical care trials suitable for community ICUs and extracted data on patient recruitment and study completion. We then retrospectively analyzed all patients admitted to ICU in 2018 in the province of Alberta, Canada and identified those who could have been considered for the chosen landmark trials based on their clinical characteristics. We finally performed a decision tree analysis to describe the number of patients, both in community and academic settings, that could have been eligible for participation in landmark trials. We further estimated the differences in time to completion with inclusion of community ICU patients.

**Results:** 10,157 adult patients were admitted to Alberta ICUs in 2018. 5650 (55.6%) were in academic centers and 4507 (44.4%) were in community hospitals. The inclusion of community ICU patients in the selected landmark critical care trials would have led on average to a 64% increase in the number of patients potentially enrolled. Furthermore, it would have decreased the mean time to completion of trials from an estimated 2.8 years with academic ICU patients only to 1.6 years (43% reduction in duration).

**Conclusions:** The inclusion of eligible patients admitted to community ICUs in multi-center clinical trials can accelerate recruitment and reduce time to trial completion.

